# Multicomponent Synthesis of α-Branched Amines via a Zinc-Mediated Carbonyl Alkylative Amination Reaction

**DOI:** 10.1021/jacs.3c14037

**Published:** 2024-03-15

**Authors:** Joseph M. Phelps, Roopender Kumar, James D. Robinson, John C. K. Chu, Nils J. Flodén, Sarah Beaton, Matthew J. Gaunt

**Affiliations:** Yusuf Hamied Department of Chemistry, University of Cambridge, Cambridge CB2 1EW, United Kingdom

## Abstract

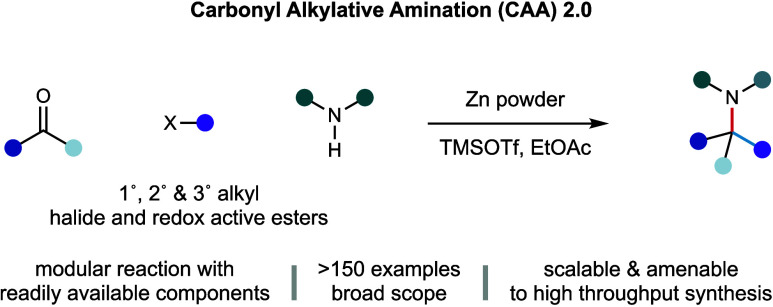

Methods for the synthesis of α-branched alkylamines are important due to their ubiquity in biologically active molecules. Despite the development of many methods for amine preparation, C(sp^3^)-rich nitrogen-containing compounds continue to pose challenges for synthesis. While carbonyl reductive amination (CRA) between ketones and alkylamines is the cornerstone method for α-branched alkylamine synthesis, it is sometimes limited by the sterically demanding condensation step between dialkyl ketones and amines and the more restricted availability of ketones compared to aldehydes. We recently reported a “higher-order” variant of this transformation, carbonyl alkylative amination (CAA), which utilized a halogen atom transfer (XAT)-mediated radical mechanism, enabling the streamlined synthesis of complex α-branched alkylamines. Despite the efficacy of this visible-light-driven approach, it displayed scalability issues, and competitive reductive amination was a problem for certain substrate classes, limiting applicability. Here, we report a change in the reaction regime that expands the CAA platform through the realization of an extremely broad zinc-mediated CAA reaction. This new strategy enabled elimination of competitive CRA, simplified purification, and improved reaction scope. Furthermore, this new reaction harnessed carboxylic acid derivatives as alkyl donors and facilitated the synthesis of α-trialkyl tertiary amines, which cannot be accessed via CRA. This Zn-mediated CAA reaction can be carried out at a variety of scales, from a 10 μmol setup in microtiter plates enabling high-throughput experimentation, to the gram-scale synthesis of medicinally-relevant compounds. We believe that this transformation enables robust, efficient, and economical access to α-branched alkylamines and provides a viable alternative to the current benchmark methods.

## Introduction

**Figure 1:**
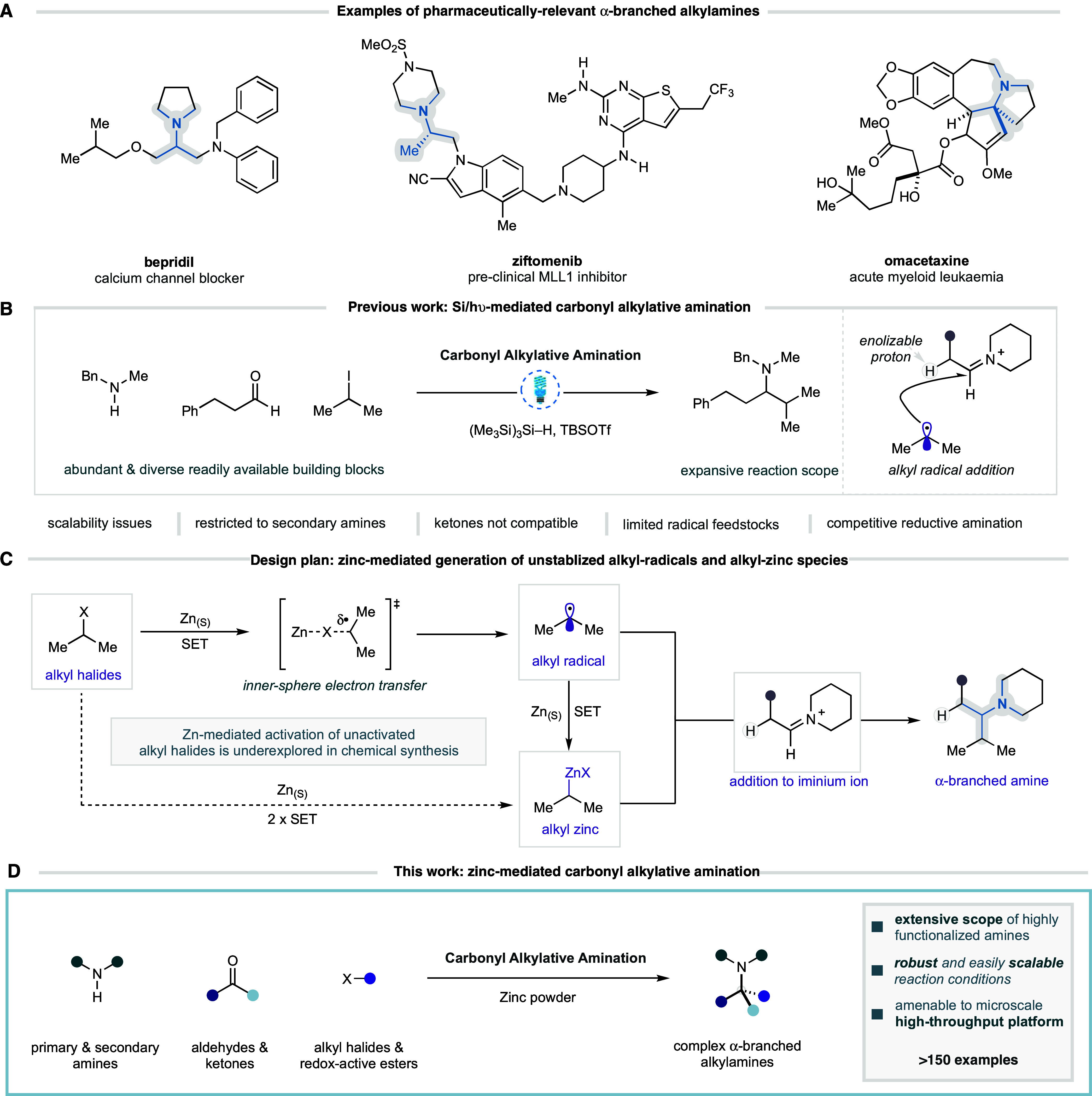
(A) Selected pharmaceutical compounds containing the α-branched alkylamine motif. (B) First-generation silane/visible-light-mediated carbonyl alkylative amination (CAA) and its limitations. (C) Design plan second-generation CAA: zinc-mediated carbonyl alkylative amination via capture of unactivated alkyl radical or alkyl zinc species. (D) Zinc-mediated carbonyl alkylative amination: a robust and general method for the efficient synthesis of complex alkylamines enabling an extensive reaction scope.

The ability of α-branched amine motifs to modulate key biological interactions and regulate the physiochemical properties of small molecules has rendered them as ubiquitous structural features among pharmaceutical agents, agrochemicals, and natural products (Figure 1A).^1^ The continuing need for the synthesis of novel biologically active α-branched amines has meant that the development of robust and general methods for their synthesis, based on modular processes that draw from diverse and abundant feedstocks, remains an important challenge to chemical synthesis.^2−8^

For many years, carbonyl reductive amination (CRA) has been the benchmark method for the synthesis of complex alkylamines and is particularly effective when there is limited α-branching in the respective amine and carbonyl components.^3^ However, poor reactivity is often observed when sterically encumbered ketones are used due to the energetically unfavorable nature of such a condensation step.^9^ Furthermore, the limited availability of dialkyl ketones, relative to aldehydes, often necessitates multistep sequences as part of the overall preparative process for α-branched alkylamines. Critically, the intrinsic dependence of this process on a hydride component makes the synthesis of α-tertiary amines—a class of amine that has become established as an important structural unit in biologically active molecules—inaccessible via this methodology.^10,11^ Therefore, a robust and general higher-order variant of CRA, in which an alkyl group could be directly added to an imine or iminium ion, derived from either alkyl aldehydes or ketones, has the potential to circumvent these limitations while unlocking greater chemical complexity through the utilization of three distinct components.

Conceptually, general higher-order CRAs exist in the form of processes such as the Strecker reaction (cyanide nucleophile), Mannich and Reformatsky reactions (enolate or equivalent nucleophiles), or Barbier reactions (allyl- or benzyl-metal nucleophiles) and their related transformations.^12−15^ However, although these reactions involve the addition of carbon nucleophiles to enolizable imines or iminium ions, they represent special cases involving species of attenuated basicity. In contrast, a general method for the addition of nonstabilized organometallic reagents (derived *in situ* from nonactivated alkyl fragments) to alkyl imines or iminium ions has remained an, essentially, unsolved synthetic challenge, with a few notable exceptions (vide infra).^16^ Due to their high reactivity, deleterious reactions are often observed between nonstabilized alkyl metal reagents and the native carbonyl and, therefore, often require preformation of alkyl-substituted imines and iminium ions, which itself is problematic due to their intrinsic instability. Moreover, the basicity of nonstabilized alkyl metal reagents results in competitive deprotonation adjacent to the carbon–nitrogen double bond, which generally reduces the scope of nascent processes to nonenolizable imines and iminium ions.

To circumvent the problems associated with the addition of classical alkyl metal nucleophiles to iminium ions, our group recently introduced a multicomponent carbonyl alkylative amination (CAA) reaction.^17−19^ This transformation leveraged the addition of neutral, but nucleophilic, alkyl radicals to iminium ion electrophiles. The alkyl radicals were generated via a halogen atom transfer (XAT)-mediated radical chain process dependent on tris(trimethylsilyl)silane [(Me_3_Si)_3_Si–H] and visible light (Figure 1B), initiated by light-driven activation of a putative ternary electron donor–acceptor (EDA) complex. The key step in realizing this transformation was the role of a rapid radical termination step through hydrogen atom transfer (HAT) between the aminium radical cation (resulting from the addition of an alkyl radical to the iminium ion) and the silane reagent. The reaction efficiently coupled a wide range of secondary amines with enolizable aldehydes and nonactivated alkyl iodides, leading to the streamlined synthesis of complex tertiary alkylamines displaying a remarkably broad range of functionally and structurally diverse substituents.

Despite the efficacy and broad scope of this process, the generation of alkyl radicals under our silane/visible-light (Si/hυ)-mediated process resulted in some limitations on the reaction that could not be resolved. The use of (Me_3_Si)_3_Si–H often led to substantial levels of reductive amination-derived side products, particularly with the use of anilines or amines containing a proximal electron-withdrawing group, which resulted in lower reaction yields and challenging purifications. Alkyl radicals generated from alkyl iodides displaying α- or β-electron-withdrawing substituents were polarity-mismatched to the highly electrophilic iminium ions, which resulted in a slower rate of addition. As a result, these highly reactive radicals rapidly engaged the hydridic (Me_3_Si)_3_Si–H reagent, which led to substantial amounts of competitive hydro-deiodination and nonproductive reactions. The dependence on XAT-derived alkyl radicals also restricted the radical precursors to alkyl halides. This mechanistic paradigm precluded the use of several classes of alkyl fragments, such as those containing α-heteroatoms, due to the inherent instability or intractable nature of the required reagents. Furthermore, while the CAA reaction was an effective solution for small-scale amine synthesis, it relied on the use of an expensive silane reagent and visible light, which, in connection with the reliance on dichloromethane as the reaction solvent, imposed limitations on the reaction’s scalability and its overall sustainability. While the (Si/hυ)-mediated process enabled excellent scope in the secondary amine, aldehyde, and alkyl halide component, significantly lower yields were obtained when using primary amines, unless the imine was derived from activated α-ketoesters.^18^ This prevented direct access to complex secondary alkylamines, which are also privileged motifs in biologically active molecules. Finally, one obvious conceptual advantage of a CAA platform over conventional CRA is the potential synthesis of complex α-tertiary amines. However, under our Si/hυ-mediated regime, ketones were always found to be incompetent substrates, unless highly activated. Taken together, these limitations have prevented the full realization of CAA as a truly general platform for amine synthesis.

We questioned whether many of these limitations could be overcome by harnessing a new method of free radical generation. We hypothesized that removing the reaction’s dependence on the hydridic (Me_3_Si)_3_Si–H reductant could potentially alleviate the issues associated with the deleterious pathways of reductive amination and hydro-deiodination. It is well-established that zinc, as well as other metal reductants, can generate alkyl radicals via single-electron transfer (SET) prior to the generation of relevant organometallic species.^20^ In 1998, Rieke and co-workers demonstrated that the formation of alkyl zinc species from alkyl bromides proceeded through two distinct SET events.^20a^ First, activated zinc mediates single-electron reduction of the alkyl bromide via inner-sphere electron transfer to generate an alkyl radical, which can then either dissociate from this complex or recombine with zinc to form the alkyl zinc species via a second SET event. It has been established that both alkyl radicals and alkyl zinc species are potentially competent nucleophiles when coupled with iminium ions.^13−19,21,22^ Therefore, we questioned whether leveraging this divergent activation mode would enable us to access a dynamic platform capable of addressing the limitations imposed by our first-generation Si/hυ-mediated transformation (Figure 1C) and expand the capabilities of the CAA process toward the general and practical synthesis of alkylamines.

Here, we report the successful realization of this idea of a second-generation CAA reaction, mediated by zinc powder (Figure 1D). In contrast to our photochemical process, no reductive amination side products are observed, which enables an operationally simple, efficient, and scalable synthesis of complex alkylamines. Overall, the reaction displays a notably broad substrate scope, which extends to the productive coupling of primary amines and unactivated ketones (previously found to be incompatible substrates under our Si/hυ-mediated conditions), enabling efficient construction of a range of amine scaffolds highly relevant in biologically active molecules. Moreover, the development of a microscale high-throughput platform was shown to be effective at enabling rapid optimization of reactions involving specific classes of substrate. We also found that carboxylic acid-derived redox-active esters (RAEs) were effective precursors for the alkyl nucleophiles, which enhance the pool of alkyl fragments and extend the structural diversity attainable through the CAA platform.

## Optimization Studies

Optimization studies began using *N*-methylbenzylamine (**1a**, 1 equiv), hydrocinnamaldehyde (**2a**, 2 equiv), and isopropyl iodide (**3a**, 3 equiv) as representative coupling partners, through which to assess the competence of a range of metal reductants (Table 1, entries 1–3) and consistent with our Si/hυ-mediated CAA reaction. A silyl triflate was used routinely in our optimization experiments because of its important role in the original Si/hυ-mediated CAA reaction.^17^ While promising activity was observed for both indium (28%) and zinc (43%), optimization was pursued using higher-yield zinc dust. Further exploration of reaction conditions revealed that increasing the amount of TMSOTf resulted in near-quantitative yields using dichloromethane (CH_2_Cl_2_) as the solvent. Unlike our first-generation CAA work, which was restricted to CH_2_Cl_2_, the Zn-mediated process showed productive yields with a broad range of solvents (see the Supporting Information for full details). EtOAc was selected as the optimal solvent due to its enhanced safety profile and lower environmental impact. The Zn-mediated CAA also worked with other silicon-based Lewis acids (entries 6–8), including the more economical TMSCl, with only slightly reduced yields. The amine·HCl salt of *N*-methylbenzylamine (**1a**) could be used in place of the free amine, with the reaction requiring a reduced loading of TMSOTf and leading to only a small reduction in yield (entries 9–10).

**Table 1:**
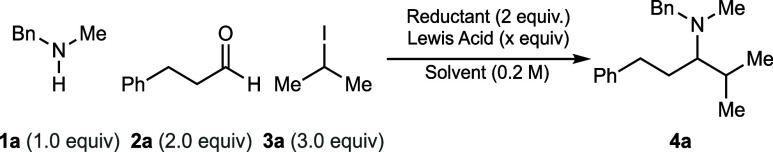
Selected Optimization for the Zinc-Mediated CAA Reaction

entry	reductant	Lewis acid (equiv)	solvent	yield (%)^a^
1	Mn	TMSOTf (1)	CH_2_Cl_2_	2
2	In	TMSOTf (1)	CH_2_Cl_2_	28
3	Zn	TMSOTf (1)	CH_2_Cl_2_	43
4	Zn	TMSOTf (2)	CH_2_Cl_2_	99
5	Zn	TMSOTf (2)	EtOAc	100
6	Zn	TBSOTf (2)	EtOAc	87
7	Zn	TMSCl (2)	EtOAc	93
8	Zn	TFA (2)	EtOAc	45
9^b^	Zn	TMSOTf (2)	EtOAc	65
10^b^	Zn	TMSOTf (1)	EtOAc	84

a Yields of **4a** were determined by ^1^H NMR using 1,1,2,2 tetrachloroethane as an internal standard.

b Amine hydrochloride salt used.

To validate the new Zn-mediated CAA reaction against the Si/hυ-mediated process, we compared reactions under both conditions where the first-generation conditions had resulted in the formation of substantial quantities of a reductive amination side product (Table 2). Under Si/hυ-mediated conditions, amines with a proximal electron-withdrawing functional group, such as *N*-benzylglycine derivative **1b**, gave the desired α-branched tertiary amine **4b** in 54% yield, alongside 23% of the reductive amination side product **5b**. Under the Zn-mediated CAA reaction conditions, we were pleased to observe that the desired product (**4b**) was formed in 78% yield, with no evidence of reductive amination. Interestingly, other amines that had similar problems under the Si/hυ-mediated conditions but were not classified as classically electronically biased also showed dramatic improvement under the Zn-mediated CAA reaction conditions. 3-(Benzylamino)propionitrile (**1c**) gave 40% of the desired product under the Si/hυ-mediated conditions accompanied by 28% of the reductive amination product, whereas the Zn-mediated CAA reaction conditions gave 64% of **4c** as the exclusive product.

**Table 2:**
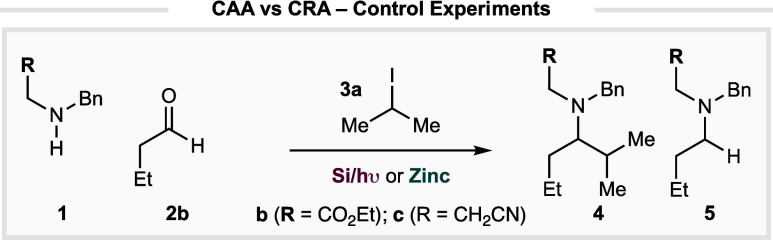
Probing Alkylation vs Reduction Pathways^a^,^b^

entry	conditions	R	**4** (%)	**5** (%)
1	Si/hυ	CO_2_Et	54	23
2	zinc	CO_2_Et	78	0
3	Si/hυ	CH_2_CN	40	28
4	zinc	CH_2_CN	64	0

a Si/hυ CAA conditions: **2b** (2 equiv), **3a** (3 equiv), (Me3Si)_3_Si–H (2 equiv), TBSOTf (2 equiv) EtOAc.

b Zn-mediated CAA conditions: **2b** (2 equiv), **3a** (3 equiv), Zn (2 equiv), TMSOTf (2 equiv), EtOAc. All yields determined by ^1^H NMR using 1,1,2,2 tetrachloroethane.

Further observations showed that the Zn-mediated CAA reaction could be performed using reduced equivalencies of both the aldehyde and alkyl iodide, compared to the Si/hυ-mediated process, if the reaction concentration was increased. Although the reaction is sensitive to air (giving 25% yield when run without an inert atmosphere), it can be carried out effectively by sparging the reaction vessel with nitrogen gas for 10 min and enabling near-quantitative (>99%) conversion to the amine product. The reaction performs well with a range of zinc powders with minimal variance in reaction yield, tolerating a range of particle sizes from 10 to 400 μm (see the Supporting Information for full details of these experiments).

**Figure 2:**
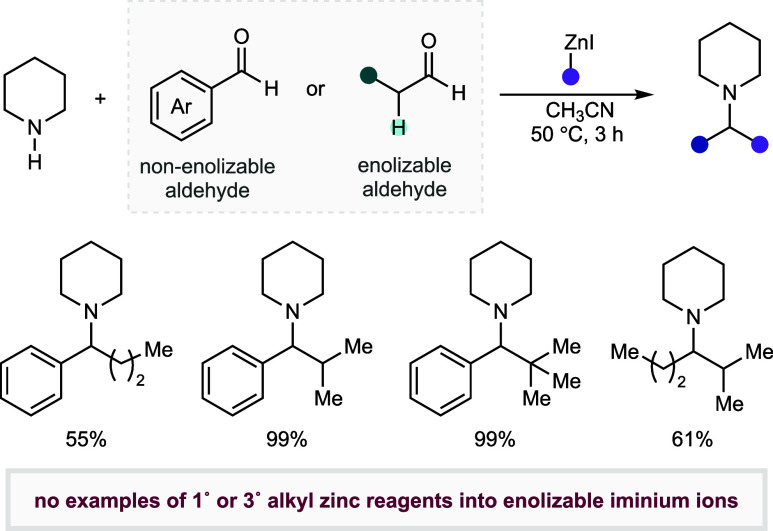
Le Gall and co-workers process for the addition of nonstabilized alkyl zinc reagents to in situ-generated iminium ions.

It is important to note that, during the course of our work, Le Gall and co-workers reported a similar process, wherein preformed, nonstabilized alkyl zinc reagents were productively added to in situ-generated iminium ions.^21g^ In the main part, this elegant method enabled the efficient construction of α-branched amines, derived from in situ-generated (nonenolizable) iminium ions and nonactivated alkyl zincs (Figure 2). Although this reaction validated that a narrow range of alkyl zincs would add to selected iminium ions, the platform displayed several limitations that could limit its potential applicability: the reaction accommodated limited functionality, falling short of the functional group tolerance necessary for the reaction to be considered a viable alternative to reductive amination in a general sense; neither primary amines nor ketones were successfully demonstrated as components of the imine/iminium electrophile; only secondary alkyl zinc reagents were shown to react productively with iminium-ions derived from enolizable aldehydes, and no results were provided for the reaction of primary- or tertiary alkyl zincs. Accordingly, we hoped our initial results would develop into a practical multicomponent platform that would lead to the development of a general amine synthesis reaction.

## Results and Discussion

**Figure 3:**
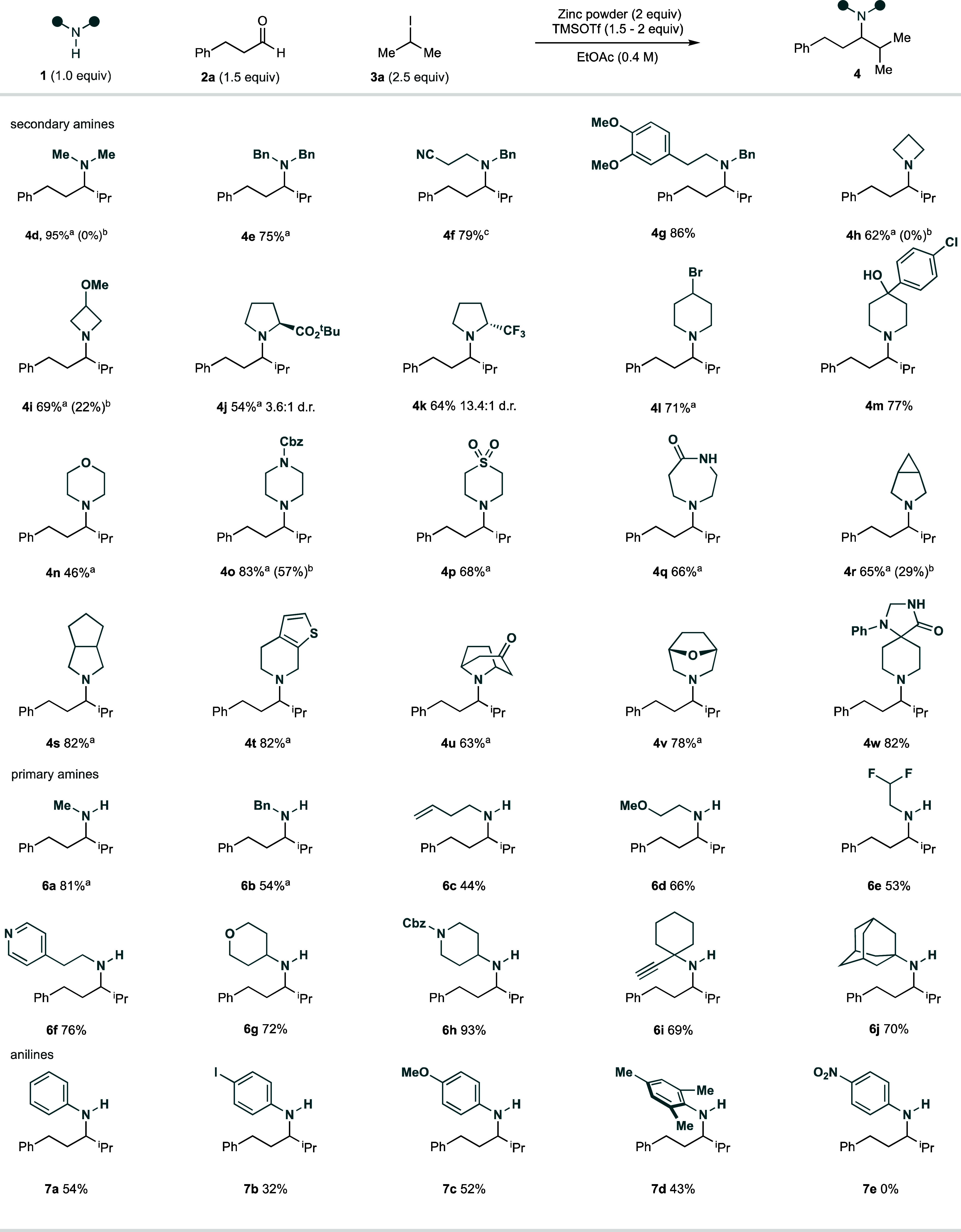
Amine scope of zinc-mediated carbonyl alkylative amination reaction. ^a^Amine hydrochloride salt.

Equipped with a set of optimized conditions, the scope of the zinc-mediated CAA reaction was explored by varying the amine component while retaining hydrocinnamaldehyde **2a** and isopropyl iodide **3a**. The performance of different secondary amines with various electronic and steric properties was assessed under our reaction conditions. A remarkably broad range of amines were able to react efficiently to give α-branched amine products **4c**–**w** in good yields (Figure 3). In comparison to our Si/hυ-mediated CAA reaction, we frequently observed superior yields with many secondary amines when using the Zn-mediated conditions: **4i** (zinc—69%; Si/hυ—22%), **4o** (zinc—83%; Si/hυ—57%), and **4r** (zinc—65%; Si/hυ—29%). Pleasingly, we were also able to access amine products that were shown to be unproductive in our previous Si/hυ-mediated CAA reactions to form amines **4e** and **4h**. No reductive amination side products were observed in any reactions performed under zinc conditions. In contrast to previous reports for the addition of discrete alkyl zinc reagents to imines or iminium ions, our optimized conditions were shown to tolerate a broad range of relevant functional groups, including esters, free alcohols, sulfones, amides, ketones, alkenes, alkynes, and aromatic heterocycles.

Under Si/hυ-mediated reaction conditions, primary amines have been unproductive coupling partners, restricting direct access complex secondary amines via this methodology. We were pleased to observe, under our optimized Zn-mediated reaction conditions, primary amines could be employed as effective coupling partners, enabling the synthesis of secondary alkylamines **6a**–**j** in good yields. The ubiquity of secondary amines in biologically active molecules, as well as the expanded capability for further downstream functionalization through the free (NH) motif, makes the successful realization of this variation of the CAA reaction an important advance toward a general amine synthesis process. Primary anilines also proved to be competent substrates for the transformation, facilitating the synthesis of secondary amines **7a**–**d**, with slightly decreased yields relative to the primary alkylamine substrates. Unfortunately, nitroaniline (**7e**) appeared not to be tolerated under our reaction conditions. Contrary to the findings of our optimization studies, the comparable use of free amine or the respective HCl salt was not general for all amines; **4n**, **4o**, and **7a**–**d** gave lower yields when the amine HCl salt is used and, in such situations, we recommend assessment with both the free amine and the HCl salt in order to obtain optimum yields.

**Figure 4:**
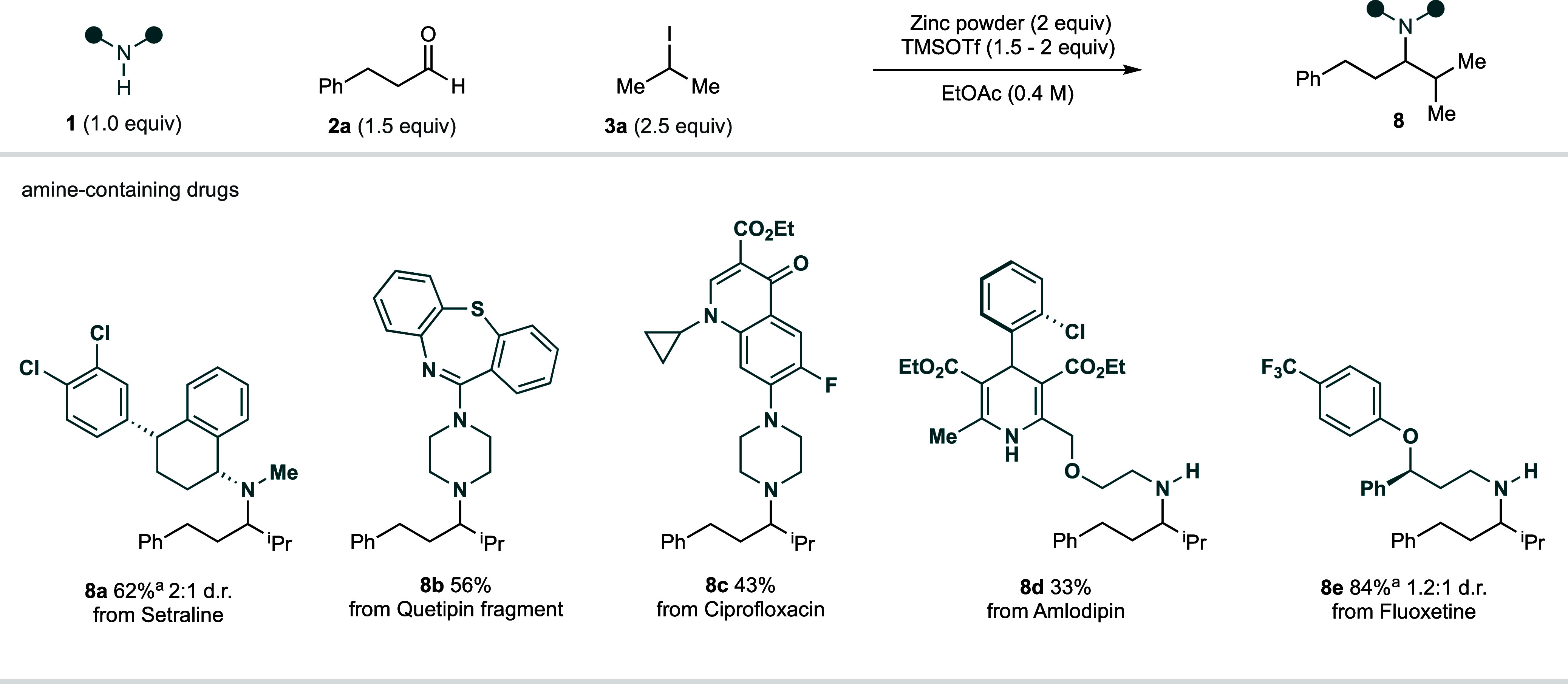
Use of amine-containing drugs in the zinc-mediated carbonyl alkylative amination reaction. ^a^Amine hydrochloride salt was used.

A key aim of this work was to develop a transformation that would tolerate the types of complex functionality frequently encountered in pharmaceutical candidates or other biologically relevant molecules. Accordingly, several amine-containing drugs were subjected to the standard Zn-mediated CAA reaction conditions: amines **8a**–**e** could be prepared in synthetically useful yields (Figure 4) and highlight the reactions’ efficacy for the synthesis of complex alkylamine architectures.

**Figure 5:**
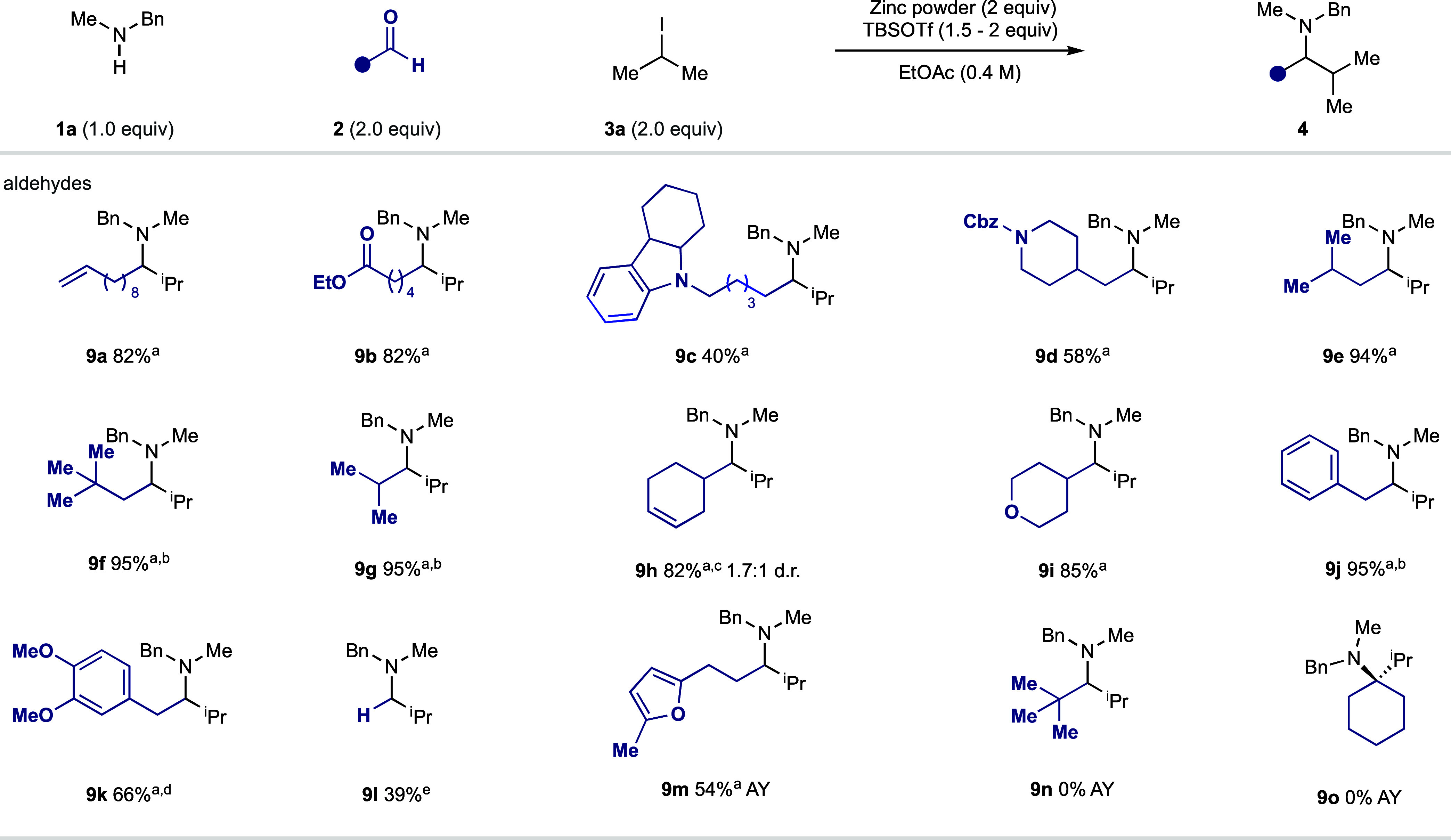
Aldehyde scope of the zinc-mediated carbonyl alkylative amination reaction. ^a^Amine hydrochloride salt. ^b^Zinc (4 equiv), **3a** (4 equiv). ^c^TMSCl (1 equiv). ^d^Zinc (3 equiv), **3a** (3 equiv), and TESCl (1 equiv). ^e^*n*BuOAc (0.4 M).

A range of linear (to form **9a**–**c**) and branched (to form **9d**–**i**) aldehydes were good substrates in the Zn-mediated CAA reaction, producing the corresponding amine products in good yields. It is notable that even aldehydes with an acidic α-proton reacted effectively to give amines **9j**–**k** (Figure 5). When the aldehyde component contained an electron-rich π-system (**9h** and **9k**), the use of a weaker Lewis acid (TMSCl or TESCl) was required to achieve optimal yields. Paraformaldehyde was also a competent coupling partner, enabling the synthesis of unbranched amine **9l**. Unfortunately, α-tertiary aldehydes and ketones (**9n**–**o**) were unsuitable reaction partners under the current reaction conditions, presumably due to a slow condensation and the lower reactivity of the corresponding sterically hindered and less-electrophilic iminium ions. Contrary to previously established examples of organometallic addition to benzaldehyde-derived imines or iminium ions, these substrates were found to be capricious under our reaction conditions (see the Supporting Information for full details).

**Figure 6:**
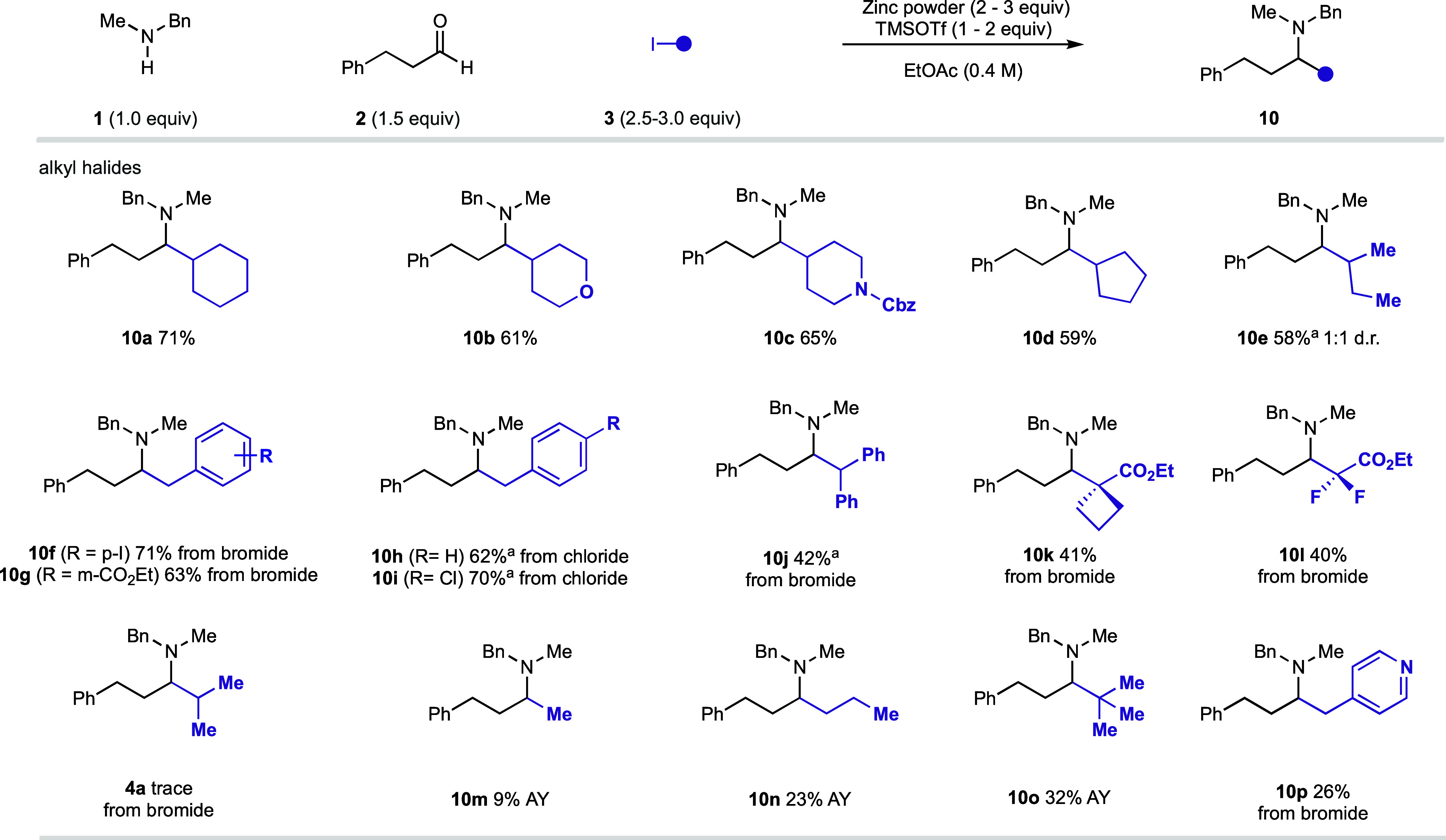
Alkyl iodide scope of the zinc-mediated carbonyl alkylative amination reaction. ^a^Amine hydrochloride salt.

Next, attention turned to the compatibility of alkyl iodides, and we were pleased to observe that a range of cyclic and linear nonactivated secondary alkyl iodides reacted well and produced the amine products **10a**–**e** in good yields (Figure 6). A range of benzyl bromides and benzyl chlorides were also coupled effectively under our optimized reaction conditions, enabling efficient access to β-arylethyl amines **10f**–**j**. In addition, alkyl halides bearing proximal electron-withdrawing groups (that were prone to rapid proto-deiodination under our previous Si/hυ-mediated conditions) were also shown to react effectively to give amines **10k**–**l**.

It is important to note the addition of stabilized alkyl halide species has previously been reported to enolizable imines and iminium ions, under Barbier- or Reformatsky-type regimes.^14,15,21^ However, there are benefits to having one reaction platform that can exploit the different classes of alkyl halides to access the diverse amine architectures. The reaction shows an intrinsic selectivity for nonactivated alkyl iodides over the corresponding alkyl bromides; a reaction of 2-bromopropane failed to yield amine **4a**. Unfortunately, heterobenzylic bromides showed poor but synthetically useful yields under our optimized reaction conditions (**10p**).

In contrast to the Si/hυ-mediated CAA reaction, nonactivated primary and tertiary alkyl iodides were shown to be low-yielding under the optimized Zn-mediated reaction conditions (exemplified by the results for amines **10m**–**o**). As the Si/hυ-mediated CAA reaction was productive with primary, secondary, and tertiary alkyl radicals, we questioned whether the low yields in the zinc-mediated reaction could be attributed to the poor reactivity of the alkyl zinc species. To explore this, we preformed alkyl zinc species formed from nonstabilized 2-iodopropane and 2-methyl-2-iodopropane and showed they were competent nucleophiles under our standard reaction conditions, giving yields of 79% (**4a**) and 91% (**10o**), respectively. Surprisingly, the preformed primary alkyl zinc (derived from *n*-propyl iodide) remained low-yielding (20%, **10n**); see the Supporting Information for full details. Considering these observations, we hypothesized that the low yields observed for the reaction of tertiary alkyl iodides, under our reaction conditions, could be attributed to deleterious S_N_1- or E1-type processes under acidic reaction conditions. However, as the reaction of the primary alkyl zinc species suffered from inherently low reactivity and knowing that primary radical addition to iminium ions proceeds at a diminished rate relative to the other iodide classes,^23^ we looked to explore the effect of additives to improve the efficiency of this challenging class of coupling reactions.

Catalytic amounts of transition-metal additives have been well-established to modulate the reactivity of alkyl zinc species. Copper(I) salts have been shown to substantially enhance the reactivity of alkyl zinc species in Barbier-type reactions.^ ^21c,21e^^ It has been suggested that these catalysts intercept and stabilize the associated carbon-centered radical during zinc-mediated radical formation from alkyl iodides. However, it is also known that copper(I) salts can catalyze halogen–zinc exchange processes, leading to some mechanistic ambiguity surrounding the role of copper iodide in these processes. However, considering these reports, we set about exploring its ability to improve the coupling efficiency of nonactivated primary alkyl iodides. Given the increasing number of components in the Zn-mediated CAA process, we elected to explore this latest iteration of the reaction using a high-throughput experimentation platform.

## High-Throughput Experimentation (HTE)

The efficient optimization of complex multicomponent reactions remains an important challenge in organic chemistry. The vast area of “reagent space”, encompassed by a process with many optimizable parameters, can render this scale of optimization unfeasible using traditional synthetic approaches. To address this challenge and attempt to efficiently evaluate the utility of a copper(I) catalyst, we sought to develop a high-throughput system capable of performing rapid reaction optimization.^24^ We hoped the realization of this platform would enable us to unlock productive reactivity for primary alkyl iodides through undertaking a multiparameter optimization on microscale format.

To translate our reaction from a 0.4 mmol reaction down to a 10–20 μmol system, we had to overcome four key challenges: (1) achieving effective mixing of the heterogeneous reaction mixtures; (2) the requirement that the reaction be performed under inert conditions; (3) the use of corrosive activating agents such as TMSOTf, which often are incompatible with methods of sealing reactions conducted in plate format; and (4) the need to effectively remove excess zinc byproducts from the reaction mixture to enable accurate quantitative analysis. Critically, we aimed to base our HTE-optimization platform on a traditional microplate format that would allow for its integration into automated workflows.^24,25^

During our initial attempts toward establishing this microscale high-throughput platform, it became clear that ethyl acetate would not be a suitable solvent for this system due to its relatively high volatility that resulted in a lack of reproducibility.^24,25^ To combat this, we screened a variety of solvents with boiling points above ethyl acetate, focusing on solvents with similar properties (see the Supporting Information for full details). Pleasingly, we discovered that *n*-butyl acetate (*n*BuOAc) enabled improved reaction performance and effective suppression of solvent loss due to evaporation, while still being able to be easily removed through exposure to high vacuum.

In its fully optimized form, our microscale platform utilized polypropylene 384-well microtiter plates charged with stir bars in each well. During the reaction, the microtiter plates are enclosed in a commercial “nano-nest” reactor,^26^ which was modified with additional silicon sheets to provide effective compression of a PFA sheet onto the reaction well (see the Supporting Information for full details). This effective compression enables gastight sealing within each well, enabling effective heating on a standard hot plate under an internal inert atmosphere, circumventing the need for a glovebox. During the optimization of this platform, it was found that heating the reactions to 50 °C was essential to allow effective stirring due to the high viscosity of the reaction mixtures.^24^ The utilization of a basic resin quench (Amberlite IRA 96) allowed for the simultaneous removal of all metal and reactive species in a simple filter plate format, avoiding the need for complex plate-based liquid–liquid separation techniques. In addition, this platform could also be combined with scavenging resins to enable the effective removal of aldehyde (tris(2-aminoethyl) or hydrazine resin) or secondary amine (isocyanate resin) impurities, enabling the generation of higher-purity α-branched amine samples. Although the reaction mixtures could be effectively analyzed by liquid chromatography-mass spectrometry (LC-MS), we chose to integrate microscale ^1^H NMR, which would enable the generation of accurate yield data during the array synthesis of structurally diverse, complex alkylamines and circumvent the need for a previously attained calibrated analytical standard.

**Figure 7:**
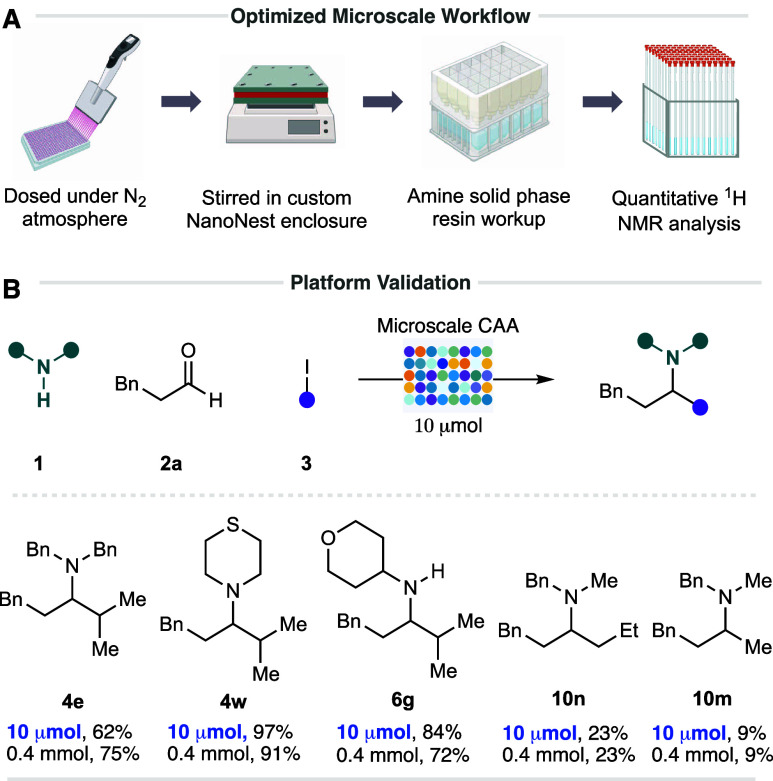
(A) Workflow for the microscale zinc-mediated CAA reaction. (B) Validation of the zinc-mediated CAA reaction; yields were determined by ^1^H NMR using 1,1,2,2 tetrachloroethane; 10 μmol conditions: amine (1 equiv), aldehyde (1.5 equiv), alkyl iodide (3 equiv), zinc (2 equiv), TMSOTf (2 equiv), *n*BuOAc (0.2 M), 50 °C, 16 h; 0.4 mmol conditions: amine (1 equiv), aldehyde (1.5 equiv), alkyl iodide (3 equiv), zinc (2 equiv), TMSOTf (2 equiv), *n*BuOAc (0.4 M), room temperature (rt), 16 h.

To validate our HTE platform for the microscale synthesis of α-branched amines, we tested the synthesis of a range of amines (**4e**, **4w**, and **6g**) using our optimized microscale, plate-based workflow (Figure 7). We were pleased to find that our HTE platform was able to effectively replicate the yields of the batch-scale reactions with a comparable efficacy. It is important to note that a slightly reduced concentration was employed (0.2 M) and elevated temperature (50 °C) was employed to ensure sufficient mixing within the microplate; however, this was observed to have no noticeable effect on the reaction yield (see the Supporting Information for full details). In addition, our microscale platform was also able to effectively replicate the yields of the poorly performing primary alkyl iodides in the synthesis of amine products **10m**–**n**.

**Figure 8:**
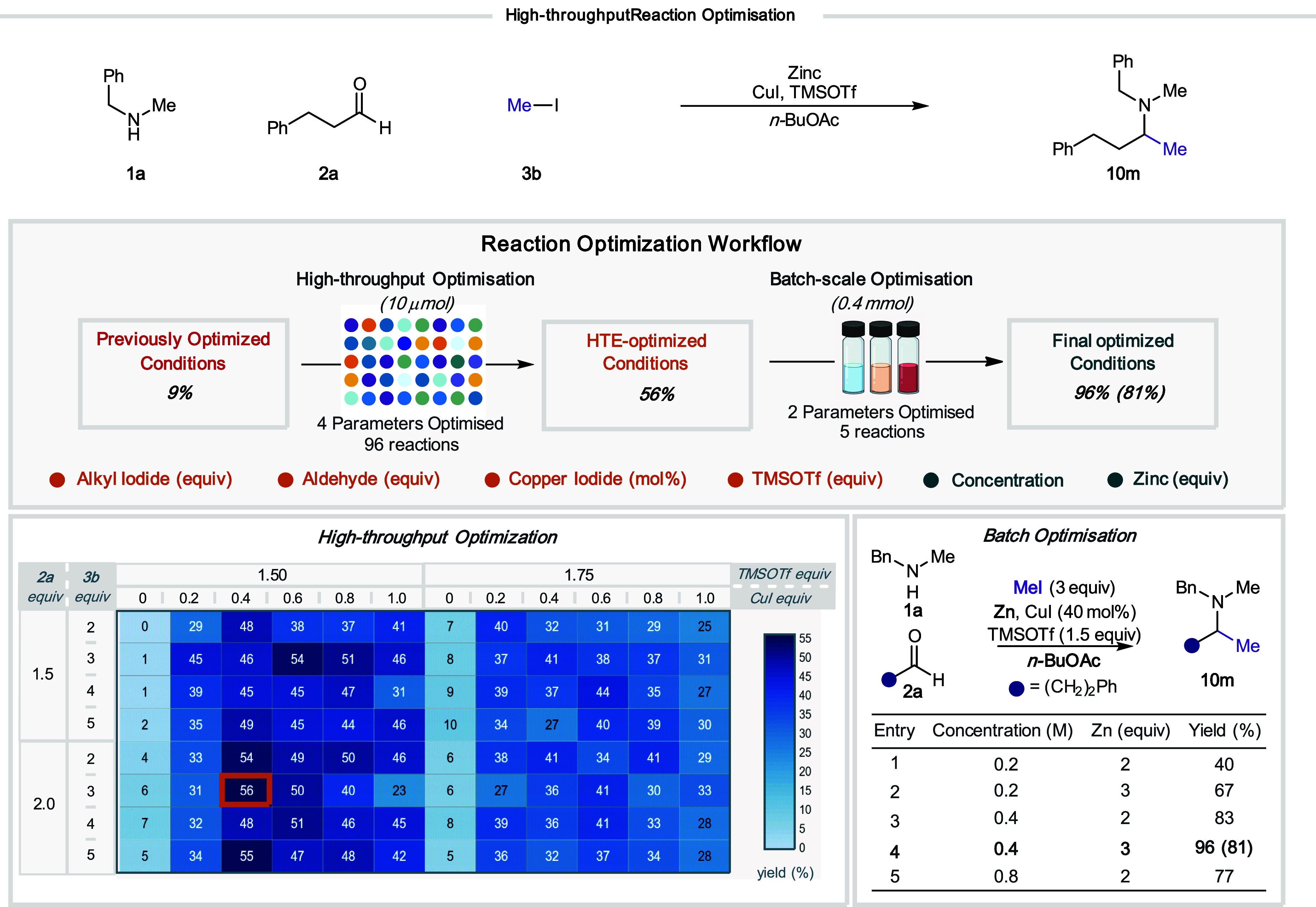
Workflow for the optimization of primary alkyl iodides using a bespoke high-throughput screening platform.

**Figure 9:**
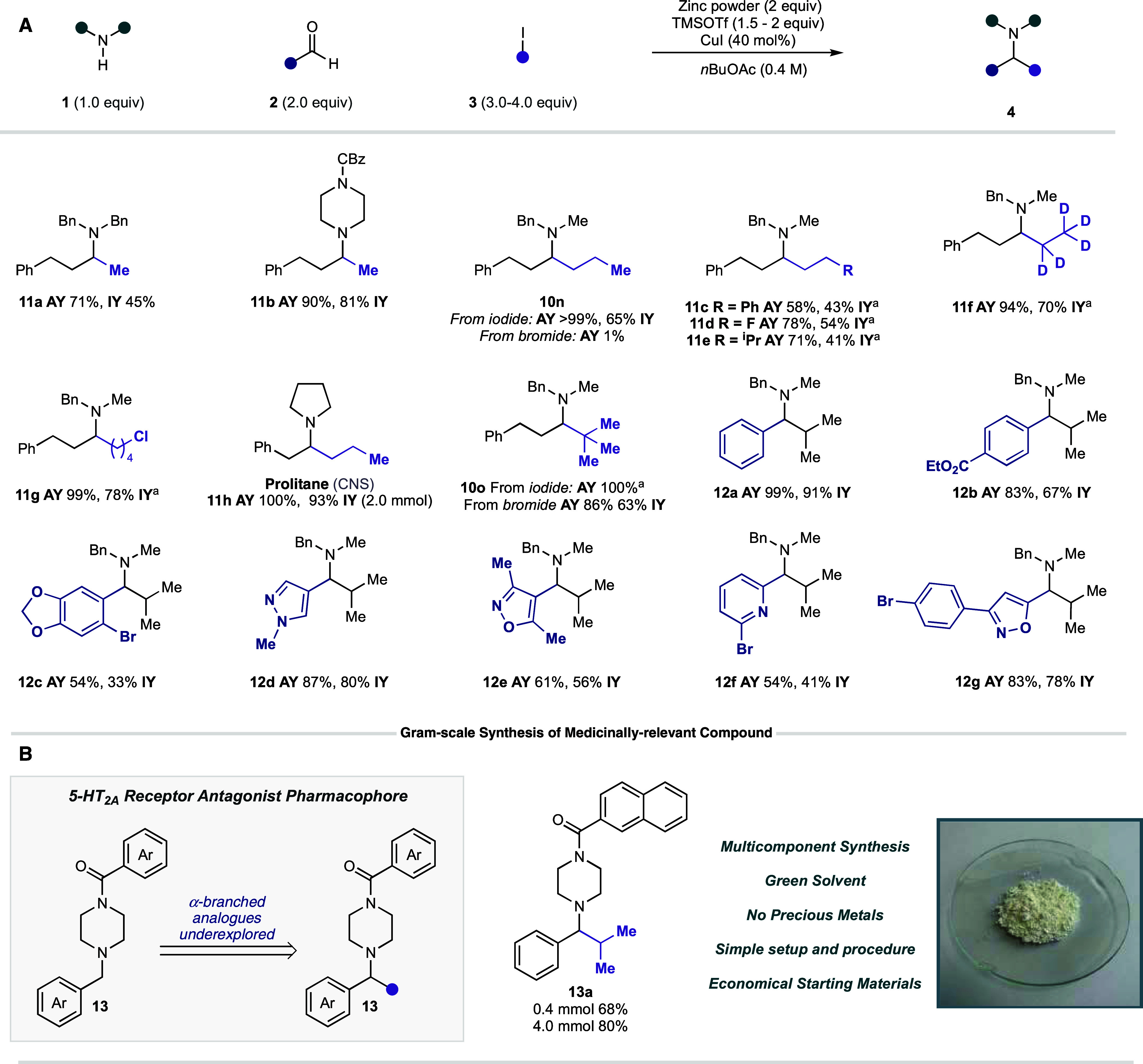
(A) Reaction scope for copper iodide-assisted Zn CAA reaction. (B) Gram-scale synthesis of biologically active α-branched alkylamine.

We next set out to utilize our microscale HTE platform to re-evaluate the use of methyl iodide as an alkylating feedstock, which was shown to perform poorly (**10m**, 9%) under the standard reaction conditions (Figure 7). The simultaneous and systematic optimization of four parameters (equivalents of methyl iodide, aldehyde, copper iodide, and TMSOTf) was performed on a single microtiter plate on a 10 μmol scale (Figure 8). We observed that the addition of 20 mol % copper iodide was essential for yields above 10% and a lower loading of TMSOTf was also beneficial to the reaction. Pleasingly, we were able to identify an improved yield of 56% after 96 reactions using our microscale HTE platform. This reaction required the addition of 40 mol % copper iodide, in combination with 2 equiv of hydrocinnamaldehyde (**2a**), 3 equiv of methyl iodide (**3b**), and 1.5 equiv of TMSOTf, in *n*BuOAc (0.2 M). However, subsequent optimization of zinc loading and concentration was performed in 0.4 mmol batch reaction due to the increased viscosity of the mixture that caused problems in the microtiter plate format. This workflow enabled the identification of optimal reaction conditions in only five further reactions through increasing zinc loading to 3 equiv and the reaction concentration to 0.4 M to give amine **10m** in near-quantitative assay yield (AY—96%, IY—81%). With these new conditions, we observed that the high yields were conserved when iodomethane was used across reactions using different amines (Figure 9, **11a**–**b**). Importantly, we found that a range of other primary iodides also worked under these conditions (**10n**, **11c**–**g**).

This new method was also shown to be applicable to the synthesis of norepinephrine-dopamine reuptake inhibitor prolintane **11h**. This synthesis was performed in a single step, from commercially available starting materials, and generated the tertiary alkylamine product in quantitative yield (IY—93%). Importantly, this procedure circumvents the multistep construction of the ketone precursor—which limited previously published methods toward the synthesis of prolintane using carbonyl reductive amination.^27^

Next, we looked to reinvestigate the contradictory reactivity observed by tertiary alkyl iodides under our reaction conditions. Working with our hypothesis that the lower yields observed under our reaction conditions could be attributed to deleterious S_N_1-type reactivity, associated with tertiary alkyl iodides under our acidic reaction conditions, we first looked at modulating the TMSOTf loading of the reaction. After further optimization (see the Supporting Information for details), we were able to unlock the reactivity of tertiary alkyl halides; reducing the TMSOTf loading from 1.5 to 0.1 equiv produced amine **10o** in good yields, from both the iodide (AY—100%) and bromide (AY—86%, IY—63%) precursors.

Furthermore, benzaldehyde had previously been found to be capricious under the original reaction conditions. However, using these newly optimized conditions, a range of electron-deficient, neutral, and electron-rich benzaldehydes gave good-to-moderate yields (**12a**–**c**). This provided a wider variety of functional groups compared to the XAT-mediated CAA reaction, which could only tolerate the use of electron-deficient benzaldehydes. Heterobenzylic aldehydes also gave more good-to-moderate yields with a variety of heteroaryl groups (**12d**–**g**).

Finally, we sought to demonstrate the scalability of our reaction as part of a potential downstream application. The 5-HT_2A_ receptor antagonist pharmacophore (**13**) is a common biologically active motif (Figure 9B) implicated in treating a range of disorders from Alzheimer’s to schizophrenia.^28^ However, larger α-branched analogues of this motif have remained underexplored.^29^ Utilizing our copper-mediated conditions, we were able to perform the synthesis of the α-branched amine at 0.4 mmol (68%) and gram-scale (4.0 mmol, 80%) to give excellent yields of the desired amine product **13a**, demonstrating the potential of this method for the rapid production of 5-HT_2A_ receptor antagonist analogues.

## Redox-Active Esters as Alkyl Donors

Thus far, the development of the CAA reaction platform has focused on the use of alkyl halides as alkylating agents. However, the low commercial availability, frequent toxicity, and limited stability of alkyl halides remain potentially limiting factors of the reaction. As zinc is known to initiate radical generation through single-electron reduction, we hypothesized that other reductively activated alkylating feedstocks may be amenable to use in the CAA transformation.

Alkyl carboxylic acids are chemically stable and widely available feedstocks.^30^ Consequently, the ability to harness this functional handle within a CAA reaction could substantially expand the chemical space accessible via this transformation. In recent years, the activation of carboxylic acids via the formation of a RAE has enabled access to a myriad of transformations.^31^ It has been demonstrated that upon single-electron reduction mediated by zinc, RAEs can fragment to give alkyl radicals.^32^ Moreover, there is also limited evidence that RAEs can be transformed into discrete alkyl zinc reagents in a similar fashion to alkyl halides.^33^ Consequently, we hypothesized that RAEs would be able to act as pseudohalides, thus enabling the efficient alkylation of *in situ*-generated iminium ions.

**Figure 10:**
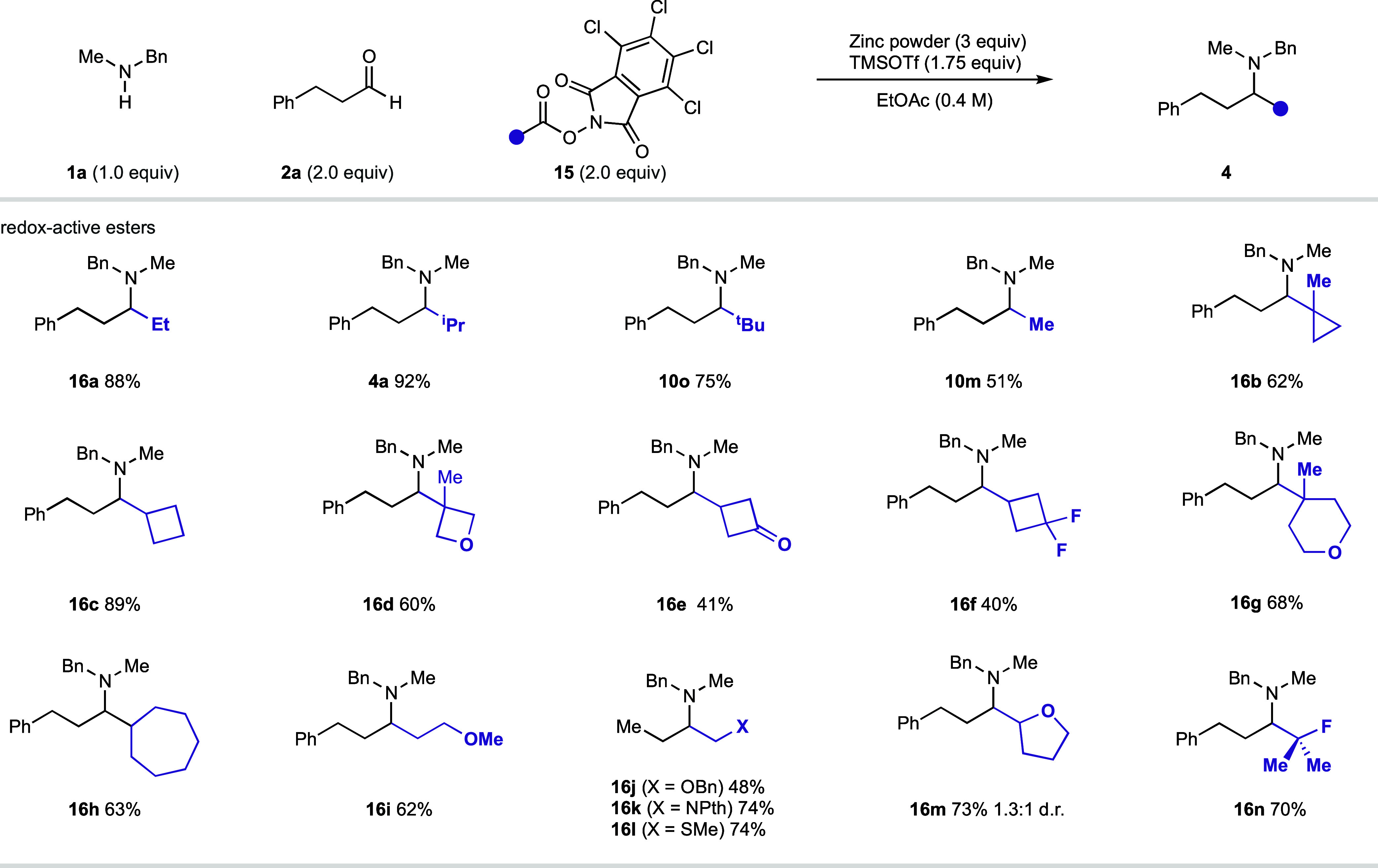
Redox-active ester scope for the zinc-mediated carbonyl alkylative amination reaction.

We were pleased to observe that *N*-hydroxytetrachlorophthalimide-derived RAEs (**11**) are indeed a viable reaction partner (Figure 10), furnishing the desired amine products from a broad range of RAEs. Happily, primary, secondary, and tertiary RAEs were able to react productively without the need for an additional copper additive. Methylation was also possible using this methodology, enabling the synthesis of amine **10m** in modest yield (51%). Critically, the increased stability of many RAEs, compared to alkyl iodides, enabled access to a greater variety of structural diversity, previously inaccessible via Si/hυ-mediated radical generation. RAEs with nearby electron-withdrawing groups (**14e**–**f**) were shown to react effectively under our optimized reaction conditions. In addition, RAEs containing α-heteroatoms (**14j**–**m**) were shown to react in good yields to produce a variety of functionalized amines. It is important to note that these amine products would be difficult to produce via reductive amination and would be inaccessible via our previous Si/hυ-mediated CAA platform due to the reaction’s dependence on alkyl iodides.

## α-Tertiary Amine Synthesis

Molecules containing the α-tertiary amine motif have shown promise across several prominent disease areas. The distinctively high level of topological complexity provided by the incorporation of three functionalized alkyl components, around a central nitrogen atom, renders them highly effective at mediating key biological interactions and exquisitely tunable in terms of their physiochemical properties.^11^ However, this class of amines are notoriously challenging to access via traditional synthetic methods, especially with all-alkyl α-tertiary amines.^10^

While catalytic methods are emerging for their synthesis, most prominently proceeding through the Giese-type functionalization of an α-amino radical, these methods are fundamentally limited in terms of accessible chemical space due to the incorporation of the structural signature associated with the alkene Giese acceptor.^34^ Arguably, one of the best-established strategies toward the synthesis of α-tertiary amines is the 1,2-addition of carbon-centered nucleophiles.^35−37^ However, these examples are invariably limited to activated, preformed imines. While unquestionably a powerful strategy, there are notably few examples of addition to nonactivated dialkyl imines, likely due to competitive α-deprotonation resulting from the basic nature of the organometallic nucleophile. Therefore, a CAA platform capable of synthesizing unbiased α-tertiary amines has the potential to dramatically improve the modularity of these processes and expediate access to this structural motif. Despite our continued efforts, under our previously reported Si/hυ-mediated regime, ketones were found to be low-yielding substrates unless the imine was formed using activated α-ketoesters. We hoped our newly optimized zinc-mediated activation mode would now allow us access to this previously inaccessible class of alkylamines. To realize this vision, we identified two key challenges in unlocking ketones as competent substrates on our Zn-mediated CAA platform. The sterically challenging condensation between the amine and the relevant ketone component would likely necessitate more forcing reaction conditions. Furthermore, the higher electron density of ketiminium renders this intermediate a less competent electrophile.

To assess this hypothesis, we evaluated the coupling between *p*-anisidine (**1b**), acetone (**2b**), and isopropyl iodide (**3a**) (Table 3). As a primary amine, we reasoned that not only does *p*-anisidine provide a sterically less hindered amine for the condensation but the resulting imine may also impart greater stability leading to a higher effective concentration of the active electrophile. The intrinsically high concentrations required for the reactions with ketimines prevented us from using our HTE system due to poor stirring that arose from the viscosity of the reaction mixture.

**Table 3:**
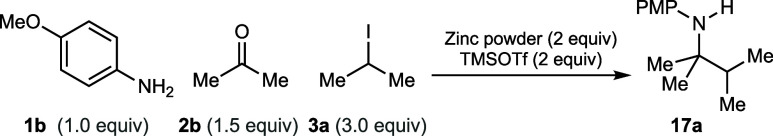
Selected Optimization for the Zinc-Mediated CAA Reaction

entry^a^	Zn (equiv)	solvent	additive	yield (%)^a^
1	2.0	EtOAc (0.4 M)	-	9
2	2.0	CH_2_Cl_2_ (0.4 M)	-	53
3	2.0	toluene (0.4 M)	-	14
4	2.0	EtOAc (0.4 M)	CuI (10 mol %)	41
5	2.0	CH_2_Cl_2_ (0.4 M)	CuI (10 mol %)	59
6	3.0	toluene (0.4 M)	CuI (10 mol %)	51
7	3.0	CH_2_Cl_2_ (0.8 M)	CuI (10 mol %)	51
8	3.0	toluene (1.6 M)	CuI (10 mol %)	61
9	3.0	toluene (1.6 M)	CuI (10 mol %), H_2_O (1.5 equiv)	73
10	3.0	CH_2_Cl_2_ (1.6 M)	CuI (10 mol %), H_2_O (1.5 equiv)	72
11	3.0	toluene (1.6 M)	CuI (10 mol %), H_2_O (1.5 equiv)	93
12	3.0	toluene (1.6 M)	CuI (10 mol %), H_2_O (1.5 equiv)	100

a Yields of **17a** were determined by ^1^H NMR using 1,1,2,2 tetrachloroethane as an internal standard.

After further extensive optimization, it was found that the presence of a copper iodide additive, in tandem with a higher reaction concentration, was essential to bring about optimal yields for the desired transformation. In addition, somewhat counterintuitively, considering the aqueous instability of the ketimine intermediate, the addition of water was found to be important for attaining high yields. This can likely be attributed to the *in situ* generation of triflic acid that facilitates more forcing conditions, generating a higher concentration of the ketimine in solution.

**Figure 11:**
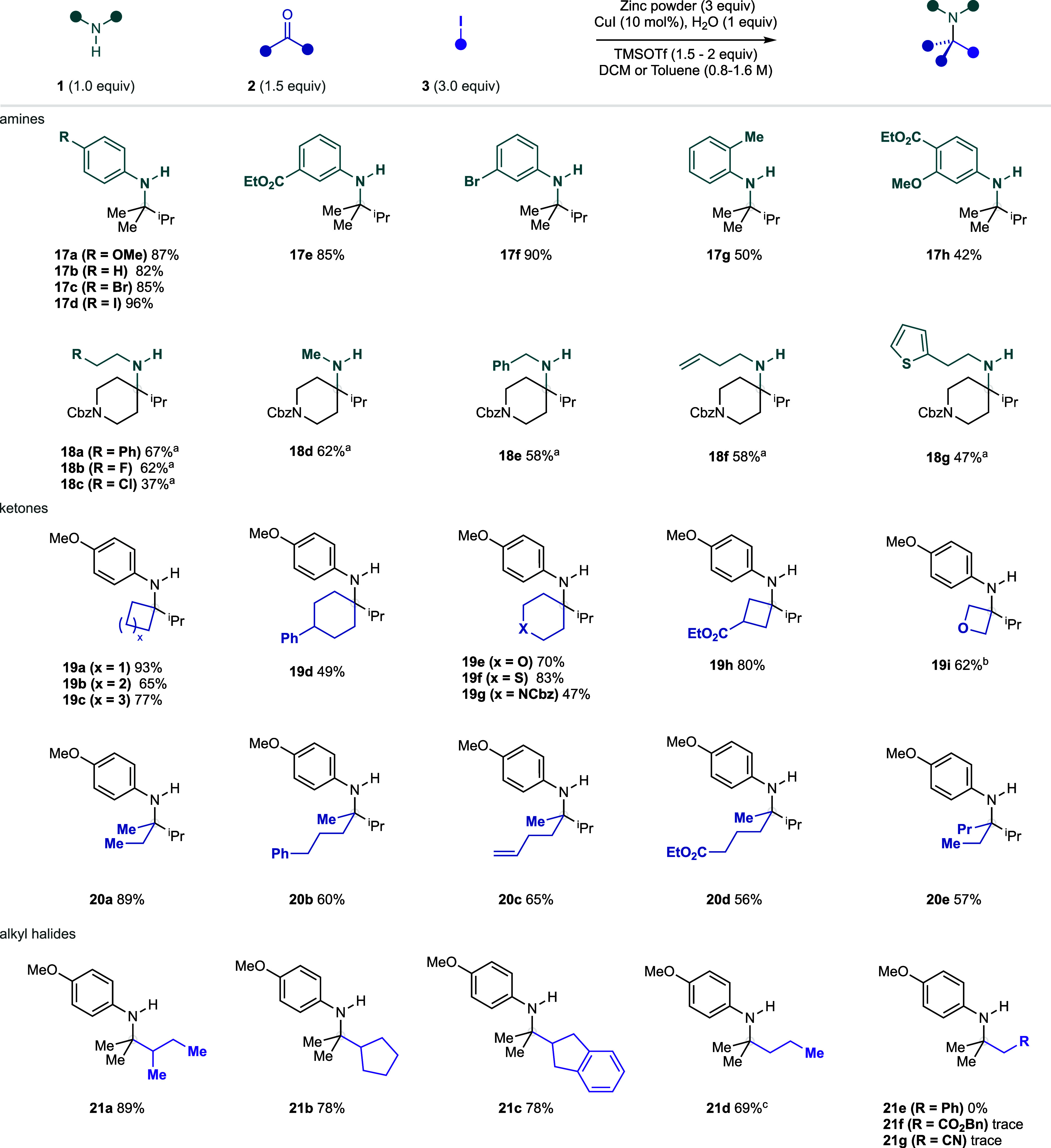
α-Tertiary amine scope for the zinc-mediated carbonyl alkylative amination reaction. ^a^Amine hydrochloride salt. ^b^Copper iodide (50 mol %), ketone (2.5 equiv), alkyl halide (4 equiv), DCM/HFIP (5:1, 0.67 M). ^c^Zinc (8 equiv), alkyl halide (5 equiv).

With optimized conditions in hand, we sought to evaluate the scope of this new method toward the synthesis of α-tertiary amines (Figure 11). We first examined a range of anilines bearing electronically diverse substituents over a range of substitution patterns. We were pleased to observe that a variety of electron-rich (**17a**, 87%), electronically neutral (**17b**, 82%), and electron poor (**17e**, 85%) anilines all reacted effectively under our reaction conditions. In addition, both aryl bromides (**17c**, 85%; **17f**, 90%) and aryl iodides (**17d**, 96%) were preserved under the reaction conditions, enabling the possibility of further product diversification. Both ortho substitution (**17g**, 50%) and disubstituted anilines (**17h**, 42%) were also shown to be tolerated under the reaction conditions. Despite the reduced electrophilicity of alkylamine-derived iminiums, a range of primary aliphatic amines were shown to react productively with 1-Cbz-4-piperidone under our reaction conditions to give amines **18a**–**g**. Notably, alkyl chlorides (**18c**, 37%), alkenes (**18f**, 58%), and an aromatic heterocycle (**18g**, 47%) were all shown to be tolerated under our reaction conditions.

We next turned our attention to the ketone scope of the transformation. A range of structurally diverse cyclic ketones were found to be successful substrates under our reaction conditions. Four- to six-membered cyclic ketones were found to react efficiently to give amines **19a**–**i** in good yields. A variety of functionalized six-membered cyclic ketones, containing O-, S-, and N-heteroatoms, were also found to be competent substrates (**19e**–**d**). Oxetanone was also compatible as the ketone component, delivering the amino-oxetane product in reasonable yield (**19i**) under slightly modified reaction conditions (lower loading of TMSOTf and addition of HFIP) that were required to suppress a decomposition pathway (see the Supporting Information for full details).^38^ Furthermore, a range of linear cyclic ketones was also shown to react proficiently under our reaction conditions (**20a**–**e**).

Finally, we examined the alkyl halide scope for this system. We were pleased to see that both secondary (**21a**–**c**) and primary alkyl iodides (**21d**) were competent nucleophiles. However, activated alkyl iodides (**21e**–**g**) were found to be unsuitable coupling partners under our reaction conditions as competitive alkylation of the primary amine often outcompeted the desired alkylation of the relevant iminium ion.

Although this CAA process was found to enable the synthesis of a diverse range of α-tertiary amine products, certain limitations were identified: ketones bearing acidic α-protons were shown to be low-yielding substrates, secondary amines, and aryl ketones were found to be incompetent substrates (likely due to the sterically challenging condensation step), and the forcing conditions necessary to mediate the condensation slightly reduced the observed functional group tolerance relative to the standard reaction (see the Supporting Information for full details). Current efforts are focused on further bespoke optimization of the Zn-mediated CAA toward the synthesis of α-tertiary amines and our studies will be reported in due course. However, despite these limitations, we believe the high levels of modularity, scalability, affordability, and operational simplicity render this method highly attractive for the synthesis of this highly privileged class of amines.

## Mechanistic Considerations

Understanding the mechanism of this Zn-mediated CAA reaction poses an extensive challenge due to potentially divergent mechanistic pathways operational at several steps in the process. In the first instance, a Rieke-type formation of alkyl zincs is proposed to proceed through the radical intermediate en route to an organozinc intermediate.^20a^ This gives rise to a system wherein both radical and polar mechanisms can exist in equilibrium (for example, the pathway to an organozinc) or in parallel (reaction of an alkyl radical or organozinc with the iminium ion). Furthermore, such a dynamic mechanistic network could be substrate-dependent and related to both the nature of the alkyl nucleophile and the iminium ion intermediate. This means that a problem with many of the possible experiments becomes the difficulty in dissecting the radical and polar pathways, which means that a more detailed and systematic mechanistic study is extremely complex and beyond the scope of this work. That said, we conducted several exploratory experiments to elucidate some fundamental mechanistic parameters.

**Figure 12:**
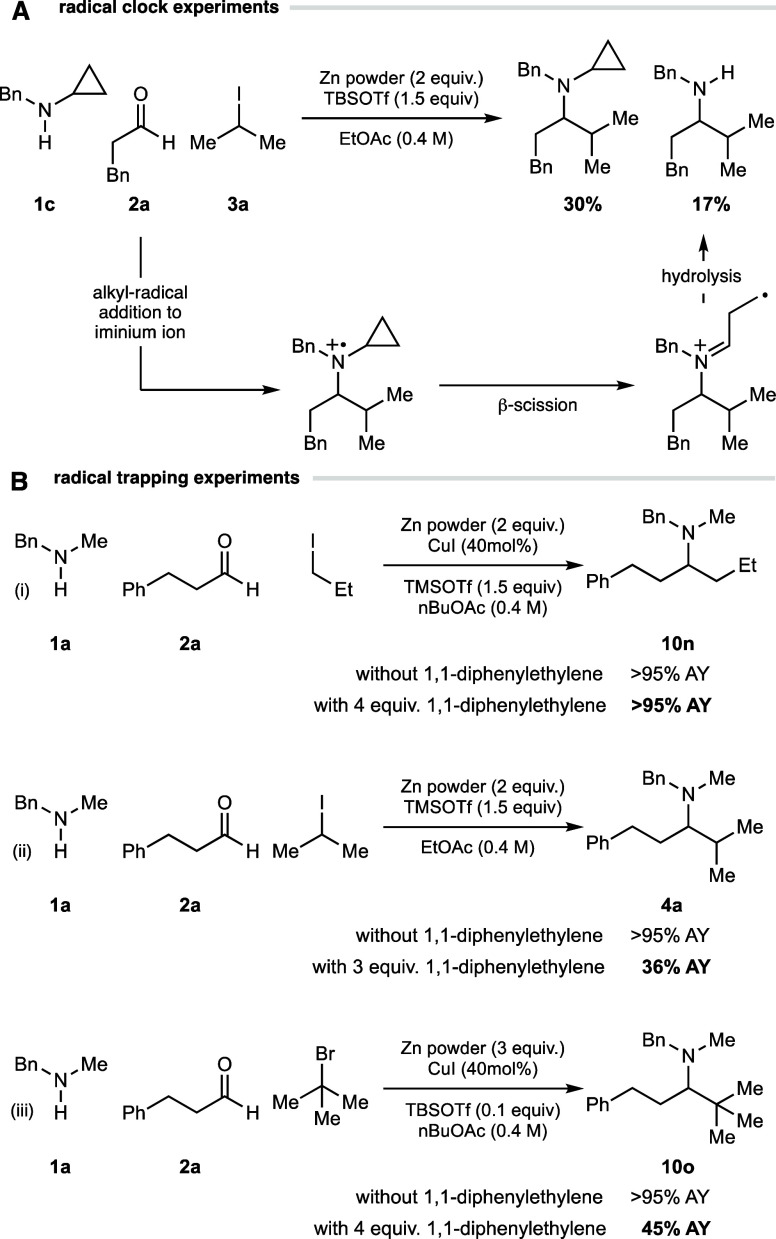
Preliminary mechanistic considerations.

The primary focus of investigations was the use of a cyclopropylamine substrate as a possible trap for an aminium radical cation (Figure 12A). Observation of ring opened (and hence hydrolyzed product) has been used before as a probe for aminyl radical cations (Section S10.5).^39^ Our experiments detected products that were devoid of a cyclopropane ring and the desired product with the cyclopropylamine motif intact. This suggests that a radical addition to an iminium ion is certainly viable under our reaction conditions. However, a control reaction revealed that there is a competing decomposition pathway for the starting cyclopropylamine and the desired cyclopropane product, which means that the loss of the cyclopropane motif may not be solely the result of a radical addition pathway. Therefore, a radical mechanism may still be most likely, but interpretation of these results should be treated with caution. A series of experiments were conducted in the presence of 1,1-diphenylethylene as a radical trap, under optimized conditions developed for primary, secondary, and tertiary alkyl iodides. On the premise that the heteroleptic alkyl zinc reagents generated in this process do not react with 1,1-diphenylethylene, these reactions may provide some information on the operation of a polar or radical pathway. For the reaction of the primary iodides, the addition of this alkene did not affect the observed yield of the desired amine product **10n** (Figure 12Bi), suggesting a radical pathway may not be operational. A note of caution needs to be applied to this result; the result does not prove the passage of a polar pathway because the addition of primary radicals to 1,1-diphenylethylene has been shown to be slow, and so it may be that the iminium ion reacts with the primary alkyl radical in preference to 1,1-diphenylethylene. However, a clearer outcome was observed for reactions with secondary and tertiary alkyl iodides that were doped with 1,1-diphenylethylene. Here, a substantial reduction in the assay yield of **4a** and **10o** (compared to a reaction in the absence of 1,1-diphenylethylene) was observed in both cases, implicating a radical pathway as being competitively operational (Figure 12Bi,ii). No product was observed for the corresponding reaction using redox-active esters in the presence of 1,1-diphenylethylene. While not conclusive, these results and further experiments shown in Section S10 suggest that both polar (primary) and radical (secondary and tertiary) pathways could be operational and are dependent on the structural nature of the alkyl nucleophile, which contrasts previous studies (Sections S10.1–S10.2).^13−^16,21g^^

## Conclusions

In recent years, the mechanistic versatility afforded by transition-metal catalysis has continued to provide impactful solutions to many problems associated with the synthesis of complex amines.^2^ In fact, the intricate design of novel ligands and catalyst systems has enabled the innovative design of novel strategies toward the synthesis of complex amines. Hence, it is important to ask why in this era of catalytic methodology this synthetic platform utilizing a stoichiometric metal reductant remains a viable solution to the synthesis of complex alkylamines? Critically, general, multicomponent, catalytic methods toward the synthesis of complex but unbiased amines remain an, essentially, unsolved synthetic challenge. The successful coupling of an *in situ*-generated imine with a transition-metal center, which would open the door to multicomponent asymmetric, amine synthesis, is yet to be demonstrated.^40^ Importantly, those catalytic methods that have been demonstrated often rely on the use of stoichiometric reductants to turn the relevant catalytic cycle.^41^ In fact, in the absence of asymmetric catalysis, the ability to perform such transformations without the need for financially and environmentally expensive transition-metal catalysts offers a more synthetically efficient solution to the synthesis of complex alkylamines. Therefore, although the future of amine synthesis undoubtably lies in the development of general catalytic regimes, in their absence, the development of cost-effective, robust, and general methods toward the synthesis of amines remains a critically important challenge of organic synthesis.

To this end, we have engineered a second-generation CAA platform for an economical, robust, and scalable strategy for the synthesis of complex alkylamines. The removal of the hydridic silane reagent [(Me_3_Si)_3_Si–H] was shown to eliminate the deleterious reductive amination pathway that limited the yields and led to challenging purifications for numerous amines under our previously disclosed Si/hυ-mediated CAA platform. Critically, this new Zn-mediated activation mode enabled the dramatic expansion of the chemical space accessible via the CAA reaction through unlocking previously incompatible substrate classes. Notably, this new reaction platform was able to facilitate modular access to secondary amines and α-tertiary amines, which were previously inaccessible under the Si/hυ-mediated CAA reaction. The utilization of activated alkyl carboxylic acids as an alkylating feedstock dramatically expanded the chemical diversity available for this component of the reaction. This expansion in scope was underpinned by the development of a microscale high-throughput platform capable of performing rapid optimization of bespoke substrates as well as the array synthesis of complex amines. We believe that the generality and operational simplicity of this transformation will provide a viable alternative to reductive amination, enabling more expedient access to complex amines in medicinal chemistry.

## Supporting Information Available

All experimental procedures, mechanistic discussion, and compound characterization (including ^1^H and ^13^C NMR spectra, IR, and HRMS data) (PDF)
