# Supplementary material for: Multicomponent Synthesis of α-Branched Amines via a Zinc-Mediated Carbonyl Alkylative Amination Reaction
Source: J Am Chem Soc. 2024 Mar 15;146(13):9045–62. doi: 10.1021/jacs.3c14037 (PMC10996026; doi:10.1021/jacs.3c14037)

Multicomponent Synthesis of α-Branched Amines via a Zinc-Mediated Carbonyl Alkylative Amination Reaction

Joseph M. Phelps, Roopender Kumar, James D. Robinson, John C. K. Chu, Nils J. Flodén, Sarah Beaton and Matthew J. Gaunt*

Yusuf Hamied Department of Chemistry, University of Cambridge, Lensfield Road, Cambridge CB2 1EW

Email: mjg32@cam.ac.uk

Contents

1. General Experimental…………………………………………………………………3
    1.1. Solvents and reagents ……………………………………………………..……3
    1.2. Chromatography and Data Analysis……………………………………….………..3
2. Reaction Optimization ………………………………………………………….…....4
    2.1. Optimization reactions procedure ……………………………………………,……4
    2.2. Optimization summary ………………………………………………………….…..4
    2.3. Zinc source optimization general procedure ……………………………………….6
3. Reductive Amination Control Experiments…………………………………..………7
    3.1. Procedure A: Zinc-mediated carbonyl alkylative amination……………….………7
    3.2. Procedure B: Si/hυ-mediated carbonyl alkylative amination…………….…..……7
    3.3. Reductive amination control experiments …………………………………………8
    3.4. Synthesis of reductive amination standards…………………………………………9
4. Reaction procedures …………………………………………………………….…...11
    4.1. **General procedure A** (amine hydrochloride salts) ………………………………11
    4.2. **General procedure B** (free amine) ………………………………………….…11
    4.3. **General procedure C** (aldehyde scope) ………………………………………….11
    4.4. **Acid-Base wash (ABW) procedure**……………………..………………….……12
    4.5. General notes on zinc-mediated carbonyl alkylative amination………………..……12
5. Reaction scope ………………………………………………………………...……13
    5.1. Amine scope ………………………………………………………………..…..13
    5.2. Aldehyde scope…………………………………………………………………….37
    5.3. Alkyl halide scope ………………………………………………………...………47
6. High-throughput experimentation (HTE) ………………………………………..…….59
    6.1. HTE general experimental …………………………………………..…………….59
    6.2. Solvent screening ……………………………………………………...……………..60
    6.3. Zinc scavenging optimization ……………………………………………………..64
    6.4. General procedure D (Modified NanoNest design and plate sealing method) ……..…64
    6.5. Multi-amine array validation experiment …………………………………...……….70
    6.6. Multi-alkyl halide array validation experiment …………………………………...…71
    6.7. Scavenger resin purification optimization …………………………………..……72
    6.8. High-throughput reaction screening (primary iodide optimization) ……………………77

7. CuI assisted Zn-mediated carbonyl alkylative amination……………………………………79
7.1. **General procedure E** (primary alkyl iodides & benzaldehydes) ……………………...79
7.2. Expanded scope ……………………………………………………………………...80
7.3. Tertiary alkyl halide optimization ……………………………………..……92
8. Redox active esters as alkylating feedstocks ………………………………………………..96
8.1. **General procedure F** (synthesis of redox active esters) ………………………………96
8.2. Redox active ester characterisation ……………………………………………………96
8.3. **General procedure G** (redox active esters) ……………………...……………..…103
8.4. Redox active ester reaction scope ……………………………………………………..104
9. Synthesis of α-tertiary amines ……………………………………………...……...113
9.1. Reaction optimization: α-tertiary amine synthesis…………………………...…..113
9.2. Reaction optimization: amino-oxetane synthesis ……………………………………..114
9.3. **General procedures H-J** (α-tertiary amines) …………………………...…………118
9.4. α-Tertiary amines reaction scope……………………………………………………119
10. Mechanistic investigations and control experiments ………………………………………138
10.1. **General procedure K-M** (Synthesis of alkyl-zinc reagents) …………………138
10.2. Reactivity of alkyl-zinc experiments……………………………………….……..140
10.3. Formation of alkyl-zinc species under standard reaction conditions …………..143
10.4. Radical trapping experiments …………………………………………….………144
10.5. Radical clock experiments ……………………………………………………...…153
10.6. Radical cyclisation radical aminyl cation trapping experiments ……..……….157
11. Additional Scope……………………………………………………………………………174
12. References …………………………………………………………………………………177
13. NMR Spectra ………………………………………………………………………………180

1. General Experimental

1.1 Solvents and Reagents

All reactions were run under an inert atmosphere (N_2) unless otherwise stated, with oven-dried glassware, using standard techniques. Anhydrous solvents were obtained from solvent stills (Et_2O was distilled from sodium triphenylmethane ketyl; THF from Na/benzophenone; MeCN, dichloromethane, and hexane from CaH_2). Anhydrous EtOAc, toluene and 1,4-dioxane were purchased. Aldehydes and ketones were used as supplied if sufficiently pure, otherwise they were purified either by distillation or column chromatography and used immediately. All amines and alkyl halides were used as supplied if sufficiently pure, otherwise they were purified either by distillation or silica column chromatography and used immediately. All commercial reagents were used as supplied unless otherwise stated. Petroleum ether 40˚- 60˚was used in all cases where the Pet. Ether abbreviation is used. TBSOTf and TMSOTf were purchased from Sigma Aldrich and used as supplied. All other commercial reagents were used as supplied unless otherwise stated.

1.2 Chromatography and Data Analysis

Analytical thin-layer chromatography (TLC) was performed on Merck Kieselgel 60 F254 0.20 mm precoated, glass backed silica gel plates. Visualization of the developed chromatogram was performed by UV absorbance (λ_{max} = 254 nm), and/or by aqueous $KMnO_4$. Flash column chromatography was performed using silica gel (Merck Geduran Si 60 [40–63 μm]) with the indicated solvent system or performed on a Teledyne ISCO CombiFlash nextgen 300+ using Redisep silica or Redisep gold silica high performance cartridges unless stated. Reverse phase column chromatography was performed on a Teledyne ISCO CombiFlash nextgen using C18 Redisep RF gold cartridges.

Nuclear magnetic resonance (NMR) spectra were recorded on a Bruker AM 400 (400 MHz) or Avance 500 (500 MHz) spectrometer or Avance 700 (700 MHz). Chemical shifts (δ) for ^{1}H NMR spectra are recorded in ppm from Me_4Si with the solvent resonance as the internal standard ($CDCl_3$ = 7.26 ppm, DMSO-d_6 = 2.50, C_6D_6 = 7.16 ppm, CD_3OD = 3.31 ppm). Data is reported as follows: chemical shift [integration, multiplicity (s = singlet, d = doublet, t = triplet, q = quartet, quint = quintet, sext = sextet, sept = septet, m = multiplet, br = broad), coupling constant and proton count]. ^{13}C NMR spectra are reported in ppm from Me_4Si with the solvent resonance as the internal standard ($CDCl_3$ = 77.16 ppm, DMSO-d_6 = 39.52, C_6D_6 = 128.06 ppm, CD_3OD = 49.00 ppm). ^{19}F NMR spectra are reported in ppm from $CFCl_3$ and are uncorrected.

High-resolution mass spectra (HRMS) were measured on a Micromass Q-TOF or on a Shimadzu QTOF LCMS-9030 spectrometer using ESI (electrospray ionisation) techniques at the Department of Chemistry, University of Cambridge.

Infrared (IR) spectra were recorded on a Perkin Elmer 1FT-IR Spectrometer or a Nicolet Summit PRO FTIR Spectrometer fitted with an ATR sampling accessory as solids or films, either through direct application or deposited in CHCl3, with absorptions reported in wavenumbers (cm^{-1}). Melting points (mp) were recorded using a Gallenkamp melting point apparatus and are reported uncorrected.

2. Reaction optimization

2.1 Optimization reactions procedure

An oven dried vial (Biotage Microwave Process Vial (10 mL), equipped with gas-tight pierceable caps) was charged with a stirring bar, Zn dust and amine hydrochloride salt if used. The vial was sealed, evacuated, and backfilled with N_2. To this was added solvent, followed by the aldehyde and the alkyl iodide. The reaction mixture was stirred for one minute, followed by the addition of TMSOTf. The reaction mixture was stirred overnight. The crude mixture was transferred into a 50 mL round bottom flask and diluted with dichloromethane (approximately 20 mL). NaOH (approximately 15 mL, 10% aq.) was added. The heterogenous mixture was vigorously stirred for 10-15 min. The aqueous layer was extracted with dichloromethane three times. The combined organic layer was dried over anhydrous $MgSO_4$ or Na_2SO_4. Solvent was evaporated under reduced pressure. Yields were then determined by ^1H NMR using 1,1,2,2-tetrachloroethane as internal standard.

2.2 Optimization summary

Table S1. Optimization studies and control reactions (0.4 mmol scale)

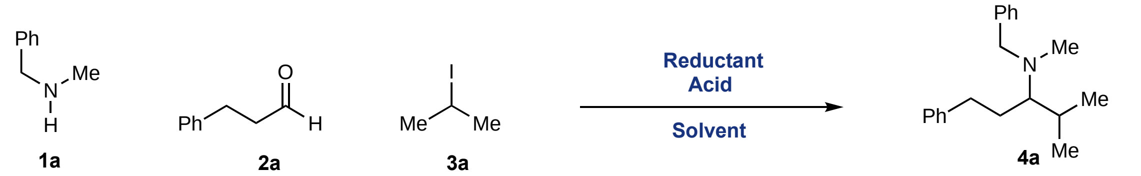

Entry	1a / 2a / 3a (equiv)	Reductant	Acid	Solvent	[Conc]	Yield / %
1	1.0 / 2.0 / 3.0	Mn (2.0 equiv)	TMSOTf (1.0 equiv)	CH_2Cl_2	0.2 M	2
2	1.0 / 2.0 / 3.0	In (2.0 equiv)	TMSOTf (1.0 equiv)	CH_2Cl_2	0.2 M	28
3	1.0 / 2.0 / 3.0	Zn (2.0 equiv)	TMSOTf (1.0 equiv)	CH_2Cl_2	0.2 M	48
4	1.0 / 2.0 / 3.0	Zn (2.0 equiv)	TMSOTf (2.0 equiv)	CH_2Cl_2	0.2 M	>99
5	1.0 / 2.0 / 3.0	Zn (2.0 equiv)	TMSOTf (2.0 equiv)	EtOAc	0.2 M	>99
6	1.0 / 2.0 / 3.0	Zn (2.0 equiv)	TBSOTf (2.0 equiv)	EtOAc	0.2 M	87
7	1.0 / 2.0 / 3.0	Zn (2.0 equiv)	TMSCl (2.0 equiv)	EtOAc	0.2 M	93
8	1.0 / 2.0 / 3.0	Zn (2.0 equiv)	TFA (2.0 equiv)	EtOAc	0.2 M	45
9	1.0 / 2.0 / 3.0	Zn (2.0 equiv)	$BF_3.Et_2O$ (2.0 equiv)	EtOAc	0.2 M	trace
10[b]	1.0 / 2.0 / 3.0	Zn (2.0 equiv)	TMSOTf (2.0 equiv)	EtOAc	0.2 M	65
11[b]	1.0 / 2.0 / 3.0	Zn (2.0 equiv)	TMSOTf (1.0 equiv)	EtOAc	0.2 M	84
12	1.0 / 2.0 / 3.0	Zn (2.0 equiv)	TMSOTf (1.0 equiv)	Dioxane	0.2 M	73
13	1.0 / 2.0 / 3.0	Zn (2.0 equiv)	TMSOTf (1.0 equiv)	MeCN	0.2 M	90
14	1.0 / 1.5 / 3.0	Zn (2.0 equiv)	TMSOTf (2.0 equiv)	EtOAc	0.2 M	65

15	1.0 / 2.0 / 2.5	Zn (2.0 equiv)	TMSOTf (2.0 equiv)	EtOAc	0.2 M	>99
16	1.0 / 1.5 / 2.5	Zn (2.0 equiv)	TMSOTf (2.0 equiv)	EtOAc	0.2 M	25
17	1.0 / 2.0 / 2.5	Zn (1.5 equiv)	TMSOTf (2.0 equiv)	EtOAc	0.2 M	40
18	1.0 / 2.0 / 2.5	Zn (3.0 equiv)	TMSOTf (2.0 equiv)	EtOAc	0.2 M	90
19	1.0 / 2.0 / 3.0	Zn (2.0 equiv)	TMSOTf (1.0 equiv)	EtOAc	0.2 M	84
20	1.0 / 1.5 / 3.0	Zn (2.0 equiv)	TMSOTf (1.0 equiv)	EtOAc	0.4 M	93
21	1.0 / 2.0 / 3.0	Zn (2.0 equiv)	TMSOTf (0.5 equiv)	EtOAc	0.2 M	74
22	1.0 / 1.5 / 3.0	Zn (2.0 equiv)	TMSOTf (0.5 equiv)	EtOAc	0.4 M	99
23	1.0 / 2.0 / 3.0	Zn (2.0 equiv)	TMSOTf (1.0 equiv)	EtOAc	0.4 M	>99
24	1.0 / 1.5 / 3.0	Zn (2.0 equiv)	TMSOTf (2.0 equiv)	EtOAc	0.4 M	>99
25	1.0 / 1.5 / 2.5	Zn (2.0 equiv)	TMSOTf (2.0 equiv)	EtOAc	0.4 M	99
26	1.0 / 2.0 / 3.0	Zn (2.0 equiv)	TMSOTf (2.0 equiv)	THF	0.2 M	50
27	1.0 / 2.0 / 3.0	Zn (2.0 equiv)	TMSOTf (2.0 equiv)	DMF	0.2 M	70
28	1.0 / 2.0 / 3.0	Zn (2.0 equiv)	TMSOTf (2.0 equiv)	DMSO	0.2 M	24

[a] Yields were determined by 1H NMR using 1,1,2,2-tetrachloroethane as internal standard. [b] Amine hydrochloride salt used.

2.3 Zinc source optimization: general procedure

An oven dried vial (Biotage Microwave Process Vial (10 mL), equipped with gas-tight pierceable caps) was charged with a stirring bar and zinc source (0.8 mmol, 2 equiv). The vial was sealed, evacuated, and backfilled with N_2. To this was added EtOAc (1 mL), followed by the N-methyl-benzylamine (52 µL, 0.4 mmol, 1 equiv), hydrocinnamaldehyde (79 µL, 0.6 mmol, 1.5 equiv) and 2-iodopropane (100 µL, 1.2 mmol, 2.5 equiv). The reaction mixture was stirred for one minute, followed by the addition of TMSOTf (144 µL, 0.8 mmol, 2 equiv). The reaction mixture was stirred overnight. The crude mixture was transferred into a 50 ml round bottom flask and diluted with dichloromethane (approximately 20 mL). NaOH (approximately 15 mL, 10% aq.) was added. The heterogenous mixture was vigorously stirred for 10-15 min. The aqueous layer was extracted with dichloromethane three times. The combined organic layer was dried over anhydrous $MgSO_4$. Solvent was evaporated under reduced pressure. Yields were then determined by ^{1}H NMR using 1,1,2,2-tetrachloroethane as internal standard.

Table S2. Optimization studies and control reactions (0.4 mmol scale)

Zinc source	Yield / %
Zinc dust, >=98% purity (<10 µm particle size, Sigma Aldrich)	>99
Nanoparticular zinc, >99% purity (40-60 nm particle size, Sigma Aldrich)	18
Zinc dust, >99.9995% purity (trace metal basis), (<150 µm particle size, Strem Chemicals)	89
Zinc powder, 99.999% purity, (<400 µm particle size, Sigma Aldrich)	93
Zinc-Copper Couple, >= 98% Zn, =<2% Cu, powder (undefined particle size, Thermofischer Scientific)	>99

3. Reductive amination control experiments

3.1 Procedure A: Zinc-mediated carbonyl alkylative amination

An oven dried vial (10 mL), equipped with gas-tight pierceable cap, was charged with a stirring bar and Zn dust (52 mg, 0.8 mmol, 2 equiv). The vial was sealed, evacuated, and backfilled with N_2. To this was added dry EtOAc (2 mL), followed by the amine (0.4 mmol, 1.0 equiv), the hydrocinamaldehyde (0.8 mmol, 2.0 equiv) and the 2-iodopropane (1.2 mmol, 3 equiv). The reaction mixture was stirred for one minute, followed by the addition of TMSOTf (144 µL, 0.4 mmol, 2.0 equiv). The reaction mixture was stirred overnight. The crude mixture was transferred into a 50 mL round bottom flask and diluted with dichloromethane (approximately 20 mL). NaOH (approximately 15 mL, 10% aq.) was added. The heterogenous mixture was vigorously stirred for 10-15 min. The aqueous layer was extracted with dichloromethane three times. The combined organic layer was dried over anhydrous $MgSO_4$ or Na_2SO_4. Solvent was evaporated under reduced pressure. Yield of the product and reductive amination by-product was determined by ^1H NMR using 1,1,2,2-tetrachloroethane as internal standard.

3.2 Procedure B: Si/hυ-mediated carbonyl alkylative amination

An oven dried vial (10 mL), equipped with gas-tight pierceable cap, was charged with a stir bar and 4 Å MS (300 mg). The vial was sealed, evacuated, and backfilled three times with N_2. To this was added dry dichloromethane (0.033 M) followed by addition of amine (0.2 mmol, 1 equiv), hydrocinamaldehyde (0.8 mmol, 2.0 equiv) and the 2-iodopropane (1.2 mmol, 3 equiv), tris(trimethylsilyl)silane (2 equiv) and TBSOTf (1 equiv). The reaction mixture was irradiated using a 40 W blue LED lamp (Kessil A160WE Tuna Blue) with vigorous stirring for 16 h at room temperature. The reaction mixture was filtered and transferred into a 50 mL round bottom flask, 10 mL hexane was added, and the solvent was removed in vacuo resulting in a light yellowish gummy solid which was rinsed with hexane (5 mL x 3). Dichloromethane (15 mL) was added, and the solution was neutralized upon stirring with NaOH (10 mL, 10% aq.) for 30 minutes. The resulting solution was transferred to a separating funnel and organic layer was separated by dichloromethane (15 mL x 3). The organic phase was dried over anhydrous $MgSO_4$, and the solvent removed under reduced pressure. Yield of the product and reductive amination by-product was determined by ^1H NMR using 1,1,2,2- tetrachloroethane as internal standard.

3.3 Reductive amination control experiments

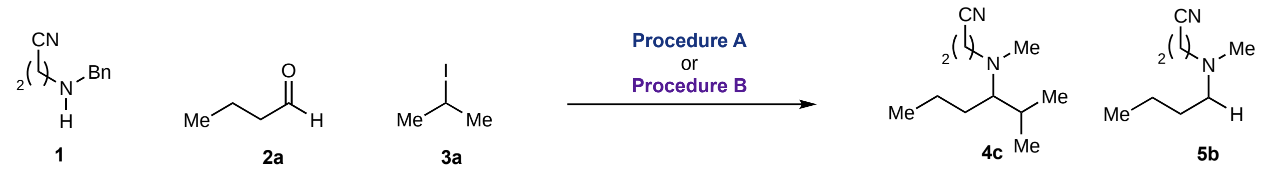

Procedure	4a Yield / %	5a Yield / %
A (Zn-mediated CAA)	64	0
B (Si/hυ-mediated CAA)	40	28

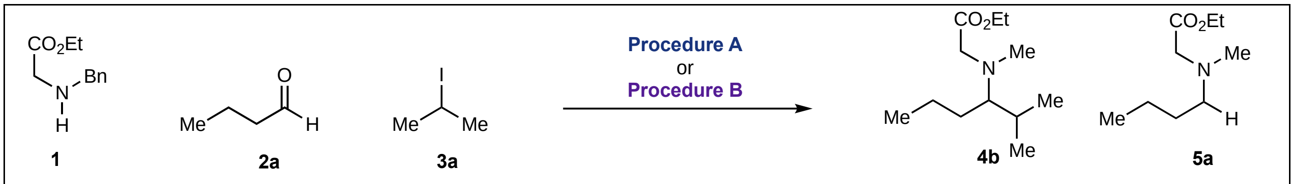

Procedure	4a Yield / %	5a Yield / %
A (Zn-mediated CAA)	78	0
B (Si/hυ-mediated CAA)	54	23

3.4 Synthesis of reductive amination standards

(ethyl N-benzyl-N-butylglycinate) (5a)

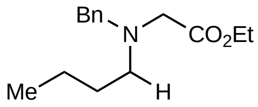

An oven dried vial (Biotage Microwave Process Vial (10 mL), equipped with gas-tight pierceable caps) was charged with a stirring bar, the N-benzylglycine ethyl ester (75 µL, 0.4 mmol, 1 equiv) and butyraldehyde (36 µL, 0.4 mmol, 1 equiv). The vial was sealed, evacuated and backfilled with N_2. To this was added dry DCE (1.0 mL). The vial was uncapped and then sodium triacetoxyborohydride (118 mg, 0.56 mmol, 1.4 equiv) was added. The vial was recapped and placed back under N_2 with vacuum back-refills. The reaction mixture was stirred for 24 h. The crude mixture was transferred into a 50 ml round bottom flask, then NaOH (approximately 15 mL, 10% aq.) and Et_2O (approximately 15 mL) was added. The heterogenous mixture was vigorously stirred for 10-15 min. The aqueous layer was extracted with Et_2O three times. The combined organic layer was dried over anhydrous $MgSO_4$. Solvent was evaporated *in vacuo*. Crude material was purified by column chromatography (0-15% EtOAc in Pet. Ether) yielding the product as a colourless oil (65 mg, 65% yield).

^{1}H NMR (500 MHz, $CDCl_3$) δ 7.36 – 7.28 (m, 4H), 7.27 – 7.21 (m, 1H), 4.17 (d, J = 7.2 Hz, 1H), 4.14 (d, J = 7.2 Hz, 1H), 3.77 (s, 2H), 3.29 (s, 2H), 2.69 – 2.55 (m, 2H), 1.53 – 1.42 (m, 2H), 1.36 – 1.29 (m, 2H), 1.26 (t, J = 7.1 Hz, 3H), 0.96 – 0.81 (m, 4H).

^{13}C NMR (126 MHz, $CDCl_3$) δ 171.6, 139.2, 128.9, 128.2, 127.0, 60.2, 58.2, 54.3, 53.6, 29.7, 20.4, 14.3, 14.0.

IR (film, cm^{-1}): 2956, 2930, 2871, 1743, 1452, 1368, 1646, 1027, 734, 697.

HRMS: m/z calculated for $C_{15}H_{24}NO_2$ $[M+H]^+$ 250.1807; found 250.1804.

(3-(benzyl(butyl)amino)propanenitrile) (5b)

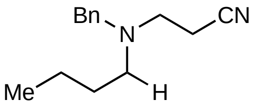

An oven dried vial (Biotage Microwave Process Vial (10 mL), equipped with gas-tight pierceable caps) was charged with a stirring bar, the 3-(benzylamino)propionitirile (62 µL, 0.4 mmol, 1 equiv) and butyraldehyde (36 µL, 0.4 mmol, 1 equiv). The vial was sealed, evacuated and backfilled with N_2. To this was added dry DCE (1.0 ml). The vial was uncapped and then sodium triacetoxyborohydride (118 mg, 0.56 mmol, 1.4 equiv) was added. The vial was recapped and placed back under N_2 with vacuum back-refills. The reaction mixture was stirred for 24 h. The crude mixture was transferred into a 50 ml round bottom flask, then NaOH (approximately 15 ml, 10% aq.) and Et_2O (approximately 15 mL) was added. The heterogenous mixture was vigorously stirred for 10-15 min. The aqueous layer was extracted with Et_2O three times. The combined organic layer was dried over anhydrous $MgSO_4$. Solvent was evaporated *in vacuo*. Crude material was purified by column chromatography (10-25% EtOAc in Pet. Ether) yielding the product as a colourless oil (35 mg, 41% yield).

^{1}H NMR (500 MHz, $CDCl_3$) δ 7.38 – 7.31 (m, 4H), 7.30-7.29 (m, 1H), 3.64 (s, 2H), 2.81 (t, J = 7.0 Hz, 2H), 2.56 – 2.49 (m, 2H), 2.42 (t, J = 7.0 Hz, 2H), 2.20 (s, 2H), 1.54 – 1.47 (m, 2H), 1.41 – 1.31 (m, 2H), 0.92 (t, J = 7.3 Hz, 3H).

^{13}C NMR (126 MHz, $CDCl_3$) δ 139.0, 128.6, 128.3, 127.1, 119.0, 58.4, 53.5, 49.2, 29.3, 20.4, 16.3.

IR (film, cm^{-1}): 2956, 2930, 2861, 1809, 1494, 1452, 1370, 1135, 1073, 736, 698.

HRMS: m/z calculated for $C_{13}H_{19}N_2$ $[M+H]^+$ 217.1705; found 217.170.3

4. Reaction procedures

4.1 General procedure A (amine hydrochloride salts)

An oven dried vial (Biotage Microwave Process Vial (10 mL), equipped with gas-tight pierceable caps) was charged with a stirring bar, the amine hydrochloride salt (0.4 mmol, 1 equiv) and Zn dust (0.8-1.6 mmol, 2-4 equiv). The vial was sealed, evacuated, and backfilled with N_2. To this was added 1.0 mL dry EtOAc, followed by the aldehyde (0.6-0.8 mmol, 1.5-2.0 equiv) and the alkyl iodide (0.8 –1.6 mmol, 2- 4 equiv). The reaction mixture was stirred for one minute, followed by the addition of TMSOTf (0.2- 0.4 mmol, 0.5-1.0 equiv). The reaction mixture was stirred overnight. The crude mixture was transferred into a 50 mL round bottom flask and diluted with dichloromethane (approximately 20 mL). NaOH (approximately 15 mL, 10% aq.) was added. The heterogenous mixture was vigorously stirred for 10-15 min. The aqueous layer was extracted with dichloromethane three times. The combined organic layer was dried over anhydrous $MgSO_4$ or Na_2SO_4. Solvent was evaporated under reduced pressure. If the product is not pure, column chromatography on silica gel is performed.

4.2 General procedure B (free amines)

An oven dried vial (Biotage Microwave Process Vial (10 mL), equipped with gas-tight pierceable caps) was charged with a stirring bar and Zn dust (0.8-1.6 mmol, 2-4 equiv). The vial was sealed, evacuated, and backfilled with N_2. To this was added dry EtOAc (1 mL), followed by the amine (0.4 mmol, 1.0 equiv), the aldehyde (0.6-0.8 mmol, 1.5-2.0 equiv) and the alkyl iodide (0.8 –1.6 mmol, 2-4 equiv). The reaction mixture was stirred for one minute, followed by the addition of TMSOTf (0.3-0.4 mmol, 1.5- 2.0 equiv). The reaction mixture was stirred overnight. The crude mixture was transferred into a 50 mL round bottom flask and diluted with dichloromethane (approximately 20 mL). NaOH (approximately 15 mL, 10% aq.) was added. The heterogenous mixture was vigorously stirred for 10- 15 min. The aqueous layer was extracted with dichloromethane three times. The combined organic layer was dried over anhydrous $MgSO_4$ or Na_2SO_4. Solvent was evaporated under reduced pressure. If the product is not pure, column chromatography on silica gel is performed.

4.2 General procedure C (aldehyde scope)

An oven dried vial (Biotage Microwave Process Vial 10 mL, equipped with gas-tight pierceable caps) was charged with a stirring bar, N-methylbenzylamine hydrochloride (0.2 mmol, 1 equiv) and Zn dust (0.4-0.8 mmol, 2-4 equiv). The vial was sealed, evacuated and backfilled with N_2. To this was added 1.0 ml dry EtOAc, followed by the aldehyde (0.6-0.8 mmol, 1.5-2.0 equiv) and 2-iodopropane (0.8 –1.6 mmol, 2-4 equiv). The reaction mixture was stirred for one minute, followed by the addition of TBSOTf (0.2 mmol, 1.0 equiv), unless stated. The reaction mixture was stirred overnight. The mixture was diluted with dichloromethane and transferred to a round-bottom flask (Note: If any zinc remained, the mixture was filtered). HCl (3M) in Et_2O (0.2 mL) was added. Solvent was removed *in vacuo*. The solid that remained was dissolved by a minimum amount of dichloromethane. The mixture was diluted with Pet. Ether (Note: a precipitate was formed upon addition of Pet. Ether). With careful control of pressure, the dichloromethane, not the Pet. Ether, was removed *in vacuo*. After removing the dichloromethane, time was given for the precipitate to settle. The Pet. Ether then was decanted. While stirring, Pet. Ether was added. Time was given for the precipitate to settle. The Pet. Ether was decanted again. The precipitate was dissolved in dichloromethane. NaOH (10%, aq) was added. The aqueous and organic layers were separated. The aqueous layer was extracted twice with dichloromethane. The combined organic layer was dried over Na_2SO_4 and

filtered. Solvent was removed *in vacuo*. (Note: these steps serve as an alternative to an acid-base wash. However, we occasionally observed some non-basic impurities remained after purification. Therefore, we only adopted this procedure before developing the acid-base wash). If the product is not pure, column chromatography on silica gel is performed.

4.4 Acid-Base wash (ABW) procedure

The mixture was dissolved with a minimum amount of Et_2O or dichloromethane (Note: the protonated amine product is partly soluble in organic solvents, so only a minimum amount of Et_2O or dichloromethane is used). The solution was transferred to a separating funnel. HCl (3 M, aq) and Pet. Ether were added (Note: Do not use more polar organic solvent since the protonated amine product is partly soluble in organic solvents). After very vigorous shaking, the aqueous and organic layers were separated. (Note: the protonated amine product might not be completely soluble in water, any gum that appears is the protonated product. This is fine as they stick to the wall of the separating funnel. Don't drain the organic layer too fast as it might wash the gum out). The organic layer was extracted twice with HCl (3M, aq) (Note: the protonated amine product is partly soluble in organic layer, so multiple extractions are needed). The combined aqueous layer was washed with Pet. Ether. It was basified with aq. NaOH (10%, aq) then dichloromethane was added. After very vigorous shaking, the aqueous and organic layer were separated. (Note: Any protonated amine gum on the wall of the separating funnel should dissolve after shaking) The aqueous layer was extracted with dichloromethane twice. The combined organic layer was dried over Na_2SO_4 or $MgSO_4$. Then the solvent was removed in vacuo.

4.5 General notes on zinc-mediated carbonyl alkylative amination

- The reaction is air-sensitive, and an atmosphere of nitrogen should be maintained.
- The reaction can be conducted in different reaction flasks (round-bottomed flasks and Schleck tube) although microwave vial or schlenk flasks were used in this investigation.
- Commercial aldehydes are generally not pure and should be distilled prior to use.
- The order of addition of different reagents are important should be followed according to the procedures provided.
- When the reaction is clean and consumes all amine starting material, all non-basic impurities can be removed by performing an acid-base wash (see **general procedure ABW**) to afford the pure product.

5. Reaction scope

5.1 Amine scope

N-Benzyl-N,4-dimethyl-1-phenylpentan-3-amine (4a)

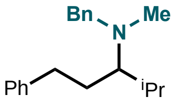

N-Benzyl-N,4-dimethyl-1-phenylpentan-3-amine was prepared according to General Procedure **A** using Zn (52 mg, 0.8 mmol, 2 equiv), N-methyl-1-phenylmethanamine hydrochloride (63 mg, 0.4 mmol, 1 equiv), hydrocinnamaldehyde (79 µL, 0.6 mmol, 1.5 equiv), 2-iodopropane (100 µL, 1.2 mmol, 2.5 equiv), and TMSOTf (72 µL, 0.4 mmol, 1 equiv) in EtOAc (1 mL). Crude material was purified by column chromatography (0-1% MeOH in dichloromethane (with 5% Et_3N)) yielding the product as a colourless oil (94 mg, 84%). Data was in line with previous characterisation.[1]

N,N, 4-trimethyl-1-phenylpentan-3-amine (4d)

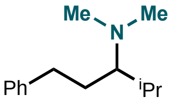

N,N,4-trimethyl-1-phenylpentan-3-amine was prepared according to General Procedure **A** using Zn (52 mg, 0.8 mmol, 2 equiv), dimethylamine hydrochloride (18 mg, 0.4 mmol, 1 equiv), hydrocinnamaldehyde (79 µL, 0.6 mmol, 1.5 equiv), 2-iodopropane (100 µL, 1.2 mmol, 2.5 equiv), and TMSOTf (72 µL, 0.4 mmol, 1 equiv) in EtOAc (1 mL). Crude material was purified by column chromatography (0-10% MeOH in dichloromethane (with 5% Et_3N)) yielding the product as a colourless oil (78 mg, 95%).

^{1}H NMR (400 MHz, $CDCl_3$): δ 7.32 (t, J = 7.7 Hz, 2H), 7.26 – 7.18 (m, 3H), 2.80 – 2.69 (m, 1H), 2.68 – 2.59 (m, 1H), 2.33 (s, 6H), 2.15 – 2.09 (m, 1H), 1.92 – 1.83 (m, 1H), 1.83 – 1.72 (m, 1H), 1.67 – 1.55 (m, 1H), 0.96 (d, J = 6.7 Hz, 3H), 0.96 (d, J = 6.7 Hz, 3H).

^{13}C NMR (101 MHz, $CDCl_3$): δ 143.1, 128.3, 128.2, 125.6, 69.2, 41.3, 35.4, 29.5, 29.1, 21.4, 19.7.

IR (film, cm^{-1}): 2953, 2871, 1453, 1367, 1174, 1140, 1088, 736, 697.

HRMS: m/z calcd for $C_{14}H_{23}N$ $[M+H]^+$ 206.1904; found 206.1907.

N,N-Dibenzyl-4-methyl-1-phenylpentan-3-amine (4e)

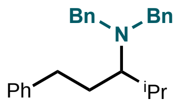

N,N-dibenzyl-4-methyl-1-phenylpentan-3-amine was prepared according to General Procedure **A** using Zn (52 mg, 0.8 mmol, 2 equiv), dibenzylamine hydrochloride (93 mg, 0.4 mmol, 1 equiv), hydrocinnamaldehyde (79 µL, 0.6 mmol, 1.5 equiv), 2-iodopropane (100 µL, 1.2 mmol, 2.5 equiv), and TMSOTf (108 µL, 0.6 mmol, 1.5 equiv) in EtOAc (1 mL). Crude material was purified by column chromatography (50-100% Pet. Ether in dichloromethane (with 5% Et_3N)) yielding the product as a colourless oil (107 mg, 75%).

^{1}H NMR (500 MHz, $CDCl_3$): δ 7.41 – 7.36 (m, 4H), 7.36 – 7.29 (m, 6H), 7.28 – 7.20 (m, 3H), 7.19 – 7.15 (m, 2H), 3.72 (d, J = 13.7 Hz, 2H), 3.65 (d, J = 13.7 Hz, 2H), 2.86 – 2.76 (m, 1H), 2.57 – 2.49 (m, 1H), 2.38 (td, J = 6.4, 5.2 Hz, 1H), 2.04 (hept, J = 6.7 Hz, 1H), 1.99 – 1.89 (m, 1H), 1.70 – 1.59 (m, 1H), 0.99 (d, J = 6.8 Hz, 3H), 0.95 (d, J = 6.7 Hz, 3H).

^{13}C NMR (126 MHz, $CDCl_3$): δ 143.1, 140.6, 129.0, 128.4, 128.3, 128.1, 126.7, 125.6, 62.5, 54.4, 35.0, 29.5, 29.1, 21.7, 20.2.

IR (film, cm^{-1}): 3025, 2952, 2797, 1493, 1452, 744, 696.

HRMS: m/z calcd for $C_{26}H_{31}N$ $[M+H]^+$ 358.2530; found 358.2532.

3-(Benzyl(4-methyl-1-phenylpentan-3-yl)amino)propanenitrile (4f)

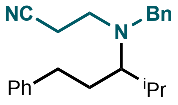

3-(Benzyl(4-methyl-1-phenylpentan-3-yl)amino)propanenitrile was prepared according to General Procedure **B** using Zn (52 mg, 0.8 mmol, 2 equiv), 3-(benzylamino)propanenitrile (65 mg, 0.4 mmol, 1 equiv), hydrocinnamaldehyde (79 µL, 0.6 mmol, 1.5 equiv), 2-iodopropane (100 µL, 1.2 mmol, 2.5 equiv), and TMSOTf (108 µL, 0.6 mmol, 1.5 equiv) in EtOAc (1 mL). Crude material was purified by column chromatography (1% MeOH in dichloromethane (with 5% Et_3N)) yielding the product as a colourless oil (101 mg, 79%).

^{1}H NMR (400 MHz, $CDCl_3$): δ 7.47 – 7.33 (m, 7H), 7.31 – 7.20 (m, 3H), 3.82 (d, J = 13.8 Hz, 1H), 3.75 (d, J = 13.9 Hz, 1H), 3.01 – 2.83 (m, 3H), 2.80 – 2.68 (m, 1H), 2.41 (q, J = 6.4 Hz, 1H), 2.23 (t, J = 7.0 Hz, 2H), 1.96 (app. oct., J = 6.9 Hz, 1H), 1.87 1.90 – 1.81 (m, 2H), 1.10 (d, J = 6.7 Hz, 3H), 1.06 (d, J = 6.6 Hz, 3H).

^{13}C NMR (101 MHz, $CDCl_3$): δ 142.6, 140.1, 128.9, 128.52, 128.48, 127.3, 126.0, 119.1, 66.4, 55.5, 48.0, 34.8, 30.80, 30.76, 21.7, 20.6, 18.3.

IR (film, cm^{-1}): 2954, 2867, 2246, 1494, 1452, 1027, 734, 697.

HRMS: m/z calcd for $C_{22}H_{28}N_2$ $[M+H]^+$ 321.2326; found 321.2319.

N-benzyl-N-(3,4-dimethoxyphenethyl)-4-methyl-1-phenylpentan-3-amine (4g)

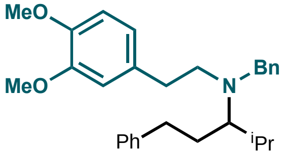

N,N-Dibenzyl-4-methyl-1-phenylpentan-3-amine was prepared according to General Procedure **B** using Zn (52 mg, 0.8 mmol, 2 equiv), *N*-benzyl-2-(3,4-dimethoxyphenyl)ethan-1-amine (108 mg, 0.4 mmol, 1 equiv), hydrocinnamaldehyde (79 μL, 0.6 mmol, 1.5 equiv), 2-iodopropane (100 μL, 1.2 mmol, 2.5 equiv), and TMSOTf (108 μL, 0.6 mmol, 1.5 equiv) in EtOAc (1 mL). Crude material was purified by column chromatography (1% MeOH in dichloromethane (with 5% Et_3N)) yielding the product as a colourless oil (149 mg, 86%).

^{1}H NMR (400 MHz, $CDCl_3$): δ 7.46 – 7.14 (m, 10H), 6.81 (d, J = 8.1 Hz, 1H), 6.67 (dd, J = 8.1, 1.9 Hz, 1H), 6.61 (s, 1H), 3.89 (s, 3H), 3.87 (s, 3H), 3.85 – 3.76 (m, 2H), 2.88 – 2.79 (m, 3H), 2.70 – 2.63 (m, 3H), 2.45 (q, J = 6.5 Hz, 1H), 2.01 – 1.82 (m, 2H), 1.82 – 1.71 (m, 1H), 1.05 – 0.99 (m, 6H).

^{13}C NMR (101 MHz, $CDCl_3$): δ 148.7, 147.1, 143.0, 141.2, 133.4, 128.8, 128.29, 128.27, 128.0, 126.5, 125.6, 120.5, 112.1, 111.2, 65.3, 55.9, 55.7, 55.5, 53.4, 35.8, 35.0, 30.5, 30.4, 21.6, 20.5.

IR (film, cm^{-1}): 2953, 2833, 1514, 1263, 1234, 1140, 1028, 732, 698.

HRMS: m/z calcd for $C_{22}H_{32}NO_2$ $[M+H]^+$ 431.2819; found 431.2814.

1-(4-Methyl-1-phenylpentan-3-yl)azetidine (4h)

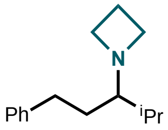

1-(4-Methyl-1-phenylpentan-3-yl)azetidine was prepared according to General Procedure **A** using Zn (52 mg, 0.8 mmol, 2 equiv), azetidine hydrochloride (37 mg, 0.4 mmol, 1 equiv), hydrocinnamaldehyde (79 μL, 0.6 mmol, 1.5 equiv), 2-iodopropane (100 μL, 1.2 mmol, 2.5 equiv), and TMSOTf (72 μL, 0.4 mmol, 1 equiv) in EtOAc (1 mL). Crude material was purified by column chromatography (0-10% MeOH in dichloromethane (with 5% Et_3N)) yielding the product as a colourless oil (54 mg, 62%).

^{1}H NMR (400 MHz, $CDCl_3$): δ 7.28 7.31 – 7.25 (m, 2H) 7.22 – 7.13 (m, 3H), 3.27 – 3.13 (m, 4H), 2.71 – 2.57 (m, 2H), 2.08 – 1.95 (m, 3H), 1.88 – 1.74 (m, 1H), 1.69 – 1.50 (m, 2H), 0.91 (d, J = 6.8 Hz, 3H), 0.89 (d, J = 7.1 Hz, 3H).

^{13}C NMR (101 MHz, $CDCl_3$): δ 142.8, 128.3, 125.7, 72.2, 54.7, 34.4, 30.1, 28.5, 19.6, 17.42, 17.38.

IR (film, cm^{-1}): 2954, 2811, 1495, 1453, 1355, 1196, 1056, 747, 697.

HRMS: m/z calcd for $C_{15}H_{24}N$ $[M+H]^+$ 218.1904; found 218.1907.

3-Methoxy-1-(4-methyl-1-phenylpentan-3-yl)azetidine (4i)

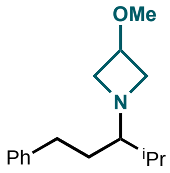

3-Methoxy-1-(4-methyl-1-phenylpentan-3-yl)azetidine was prepared according to General Procedure **A** using Zn (52 mg, 0.8 mmol, 2 equiv), 3-methoxyazetidine hydrochloride (49 mg, 0.4 mmol, 1 equiv), hydrocinnamaldehyde (79 µL, 0.6 mmol, 1.5 equiv), 2-iodopropane (100 µL, 1.2 mmol, 2.5 equiv), and TMSOTf (72 µL, 0.4 mmol, 1 equiv) in EtOAc (1 mL). Crude material was purified by column chromatography (0-10% MeOH in dichloromethane (with 5% Et_3N)) yielding the product as a colourless oil (68 mg, 69%).

^{1}H NMR (400 MHz, $CDCl_3$): δ 7.30 – 7.25 (m, 2H), 7.21 – 7.15 (m, 3H), 4.00 (quint, J = 5.9 Hz, 1H), 3.73 – 3.54 (m, 2H), 3.25 (s, 3H) 2.90 (t, J = 6.5 Hz, 2H), 2.72 – 2.56 (m, 2H), 2.11 – 2.06 (m, 1H), 1.82 (app. oct., J = 7.0, 2.9 Hz, 1H), 1.72 – 1.51 (m, 2H), 0.92 – 0.87 (m, 6H).

^{13}C NMR (101 MHz, $CDCl_3$): δ 142.7, 128.3, 128.2, 125.7, 72.3, 69.5, 60.9, 60.7, 55.9, 34.3, 30.3, 28.8, 19.4, 17.4.

IR (film, cm^{-1}): 3035, 2931, 2823, 1495, 1453, 1366, 1221, 1143, 1064, 749, 697.

HRMS: m/z calcd for $C_{16}H_{26}NO$ $[M+H]^+$ 248.2009; found 248.2010.

2,2-Dimethyl-1-((2*S*)-1-(4-methyl-1-phenylpentan-3-yl)pyrrolidin-2-yl)propan-1-one (4j)

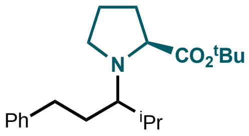

2,2-Dimethyl-1-((2*S*)-1-(4-methyl-1-phenylpentan-3-yl)pyrrolidin-2-yl)propan-1-one was prepared according to General Procedure **A** using Zn (52 mg, 0.8 mmol, 2 equiv), (*S*)-2,2-dimethyl-1-(pyrrolidin-2-yl)propan-1-one hydrochloride (77 mg, 0.4 mmol, 1 equiv), hydrocinnamaldehyde (79 µL, 0.6 mmol, 1.5 equiv), 2-iodopropane (100 µL, 1.2 mmol, 2.5 equiv), and TMSOTf (72 µL, 0.4 mmol, 1 equiv) in EtOAc (1 mL). Crude material was purified by column chromatography (0-1% MeOH in dichloromethane (with 5% Et_3N)) yielding the product as a colourless oil (68 mg, 54%, 3.6:1 d.r.). Data was in line with previous characterisation.[1]

(2*R*)-1-(4-Methyl-1-phenylpentan-3-yl)-2-(trifluoromethyl)pyrrolidine (4k)

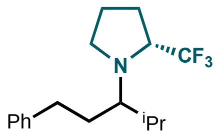

(2*R*)-1-(4-Methyl-1-phenylpentan-3-yl)-2-(trifluoromethyl)pyrrolidine was prepared according to General Procedure **B** using Zn (52 mg, 0.8 mmol, 2 equiv), (*R*)-2-(trifluoromethyl)pyrrolidine (56 mg, 0.4 mmol, 1 equiv), hydrocinnamaldehyde (79 µL, 0.6 mmol, 1.5 equiv), 2-iodopropane (100 µL, 1.2 mmol, 2.5 equiv), and TMSOTf (108 µL, 0.6 mmol, 1.5 equiv) in EtOAc (1 mL). Crude material was purified by column chromatography (0–100% dichloromethane in Pet. Ether (with 5% Et_3N)) yielding the product as a colourless oil (76 mg, 64%, 13.4:1 d.r.). Data was in line with previous characterisation.[1]

4-Bromo-1-(4-methyl-1-phenylpentan-3-yl)piperidine (4l)

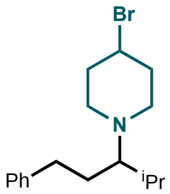

4-Bromo-1-(4-methyl-1-phenylpentan-3-yl)piperidine was prepared according to General Procedure **A** using Zn (52 mg, 0.8 mmol, 2 equiv), 4-bromopiperidine hydrobromide (98 mg, 0.4 mmol, 1 equiv), hydrocinnamaldehyde (79 µL, 0.6 mmol, 1.5 equiv), 2-iodopropane (100 µL, 1.2 mmol, 2.5 equiv), and TMSOTf (72 µL, 0.4 mmol, 1.0 equiv) in EtOAc (1 mL). Crude material was purified by column chromatography (0-1% MeOH in dichloromethane (with 5% Et_3N)) yielding the product as a colourless oil (92 mg, 71%).

^{1}H NMR (400 MHz, $CDCl_3$): δ 7.35 – 7.26 (m, 2H), 7.23 – 7.16 (m, 3H), 4.23 – 4.14 (m, 1H), 2.90 – 2.70 (m, 3H), 2.67 – 2.45 (m, 3H), 2.19 – 2.09 (m, 3H), 2.08 – 1.94 (m, 2H), 1.85 – 1.69 (m, 2H), 1.69 – 1.57 (m, 1H), 0.91 (d, J = 6.7 Hz, 6H).

^{13}C NMR (101 MHz, $CDCl_3$): δ 142.9, 128.33, 128.26, 125.6, 69.5, 51.6, 48.5, 37.53, 37.47, 34.7, 29.9, 29.8, 21.5, 20.3.

IR (film, cm^{-1}): 2950, 1452, 1145, 1104, 1007, 748, 697.

HRMS: m/z calcd for $C_{17}H_{27}BrN$ $[M+H]^+$ 324.1322; found 324.1320.

4-(4-Chlorophenyl)-1-(4-methyl-1-phenylpentan-3-yl)piperidin-4-ol (4m)

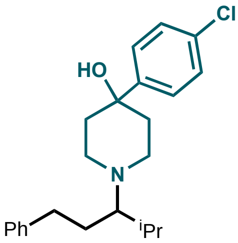

4-(4-Chlorophenyl)-1-(4-methyl-1-phenylpentan-3-yl)piperidin-4-ol was prepared according to General Procedure **B** using Zn (52 mg, 0.8 mmol, 2 equiv), 4-(4-chlorophenyl)piperidin-4-ol (84 mg, 0.4 mmol, 1 equiv), hydrocinnamaldehyde (79 μL, 0.6 mmol, 1.5 equiv), 2-iodopropane (100 μL, 1.2 mmol, 2.5 equiv), and TMSOTf (108 μL, 0.4 mmol, 1.5 equiv) in EtOAc (1 mL). Crude material was purified by column chromatography (0-2% MeOH in dichloromethane (with 5% Et_3N)) yielding the product as a colourless oil (114 mg, 77%).

^{1}H NMR (400 MHz, $CDCl_3$): δ 7.49 (d, J = 8.2 Hz, 2H), 7.39 – 7.30 (m, 4H), 7.29 – 7.21 (m, 3H), 2.99 – 2.79 (m, 3H), 2.77 – 2.62 (m, 3H), 2.30 – 2.21 (m, 1H), 2.07 (qd, J = 12.9, 4.6 Hz, 2H), 1.99 – 1.78 (m, 3H), 1.77 – 1.65 (m, 3H), 0.95 (d, J = 6.8 Hz, 3H), 0.94 (d, J = 7.0 Hz, 3H).

^{13}C NMR (101 MHz, $CDCl_3$): δ 147.3, 143.0, 132.5, 128.3, 128.2, 126.1, 125.6, 71.7, 69.5, 45.3, 45.1, 39.4, 39.2, 34.9, 29.61, 29.57, 21.6, 20.2.

IR (film, cm^{-1}): 2951, 2821, 1264, 1093, 826, 734, 699.

HRMS: m/z calcd for $C_{23}H_{31}ClNO$ $[M+H]^+$ 372.2089; found 372.2084.

4-(4-methyl-1-phenylpentan-3-yl)morpholine (4n)

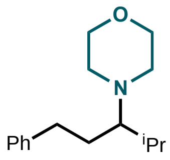

4-(4-methyl-1-phenylpentan-3-yl)morpholine was synthesized according to General Procedure **A** using Zn (52 mg, 0.8 mmol, 2 equiv), morpholine hydrochloride (49 mg, 0.4 mmol, 1 equiv), hydrocinnamaldehyde (78 mL, 0.6 mmol, 1.5 equiv), 2-iodopropane (100 mL, 1 mmol, 2.5 equiv) and TMSOTf (144 mL, 0.8 mmol, 2 equiv) in EtOAc (1 mL). The crude material was subjected to an acid-base workup according to General Procedure **F**, followed by column chromatography (10-20% Et_2O in Pet. Ether) yielding the product as a colourless oil (45 mg, 46%).

Note: The use of morpholine, instead of the hydrochloride salt, gives a significantly lower yield.

^{1}H NMR (400 MHz, $CDCl_3$): δ 7.32 – 7.24 (m, 2H), 7.23 – 7.16 (m, 3H), 3.68 (t, J = 4.7 Hz, 4H), 2.82 – 2.73 (m, 1H), 2.69 – 2.55 (m, 5H), 2.15 – 2.06 (m, 1H), 1.92 – 1.73 (m, 2H), 1.69 – 1.58 (m, 1H), 0.93 (d, J = 6.6 Hz, 3H), 0.92 (d, J = 6.5 Hz, 3H).

^{13}C NMR (101 MHz, $CDCl_3$): δ 142.9, 128.4, 128.3, 125.7, 69.5, 67.8, 49.9, 34.8, 29.4, 29.3, 21.4, 19.9.

IR (film, cm^{-1}): 2952, 2807, 1452, 1115, 1015, 858, 749, 698.

HRMS: m/z calcd for $C_{16}H_{26}NO$ $[M+H]^+$ 248.2009; found 248.2008.

Benzyl 4-(4-methyl-1-phenylpentan-3-yl)piperazine-1-carboxylate (4o)

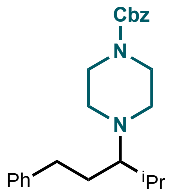

Benzyl 4-(4-methyl-1-phenylpentan-3-yl)piperazine-1-carboxylate was prepared according to General Procedure **A** using Zn (52 mg, 0.8 mmol, 2 equiv), benzyl piperazine-1-carboxylate hydrochloride (103 mg, 0.4 mmol, 1 equiv), hydrocinnamaldehyde (79 µL, 0.6 mmol, 1.5 equiv), 2-iodopropane (100 µL, 1.2 mmol, 2.5 equiv), and TMSOTf (72 µL, 0.4 mmol, 1 equiv) in EtOAc (1 mL). Crude material was purified by column chromatography (0-2% MeOH in dichloromethane (with 5% Et_3N)) yielding the product as a colourless oil (126 mg, 83%).

^{1}H NMR (400 MHz, $CDCl_3$): δ 7.44 – 7.28 (m, 7H), 7.25 – 7.19 (m, 3H), 5.19 (s, 2H), 3.51 (t, J = 5.0 Hz, 4H), 2.82 – 2.73 (m, 1H), 2.71 – 2.52 (m, 5H), 2.20 (td, J = 7.5, 4.8 Hz, 1H), 1.90 – 1.73 (m, 2H), 1.72 – 1.61 (m, 1H), 0.95 (d, J = 6.9 Hz, 6H).

^{13}C NMR (101 MHz, $CDCl_3$): δ 155.2, 142.6, 136.8, 128.3, 128.23, 128.18, 127.8, 127.7, 125.6, 69.3, 66.9, 49.0, 44.6, 34.5, 29.5, 29.3, 21.3, 20.0.

IR (film, cm^{-1}): 2951, 2809, 1697, 1427, 1238, 1119, 1013, 733, 696.

HRMS: m/z calcd for $C_{24}H_{33}N_2O_2$ $[M+H]^+$ 381.2537; found 381.2532.

4-(4-Methyl-1-phenylpentan-3-yl)thiomorpholine 1,1-dioxide (4p)

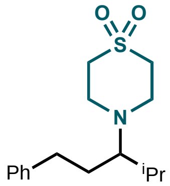

4-(4-Methyl-1-phenylpentan-3-yl)thiomorpholine 1,1-dioxide was prepared according to General Procedure **A** using Zn (52 mg, 0.8 mmol, 2 equiv), thiomorpholine 1,1-dioxide hydrochloride (69 mg, 0.4 mmol, 1 equiv), hydrocinnamaldehyde (79 µL, 0.6 mmol, 1.5 equiv), 2-iodopropane (100 µL, 1.2 mmol, 2.5 equiv), and TMSOTf (72 µL, 0.4 mmol, 1 equiv) in EtOAc (1 mL). Crude material was purified by column chromatography (0-2% MeOH in dichloromethane (with 5% Et_3N)) yielding the product as a colourless oil (80 mg, 68%).

^{1}H NMR (400 MHz, $CDCl_3$): δ 7.35 – 7.27 (m, 2H), 7.24 – 7.16 (m, 3H), 3.18 – 3.06 (m, 4H), 3.05 – 2.92 (m, 4H), 2.79 – 2.60 (m, 2H), 2.29 (td, J = 7.7, 4.7 Hz, 1H), 1.84 – 1.68 (m, 3H), 0.94 (d, J = 6.8 Hz, 6H).

^{13}C NMR (101 MHz, $CDCl_3$): δ 142.1, 128.5, 128.3, 126.0, 70.6, 52.9, 47.5, 34.4, 30.8, 30.4, 21.5, 20.4.

IR (film, cm^{-1}): 2954, 2830, 1300, 1268, 1122, 1035, 860, 733, 699.

HRMS: m/z calcd for $C_{16}H_{26}NO_2S$ $[M+H]^+$ 296.1679; found 296.1683.

1-(4-Methyl-1-phenylpentan-3-yl)-1,4-diazepan-5-one (4q)

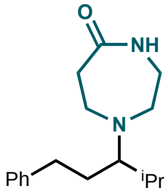

1-(4-Methyl-1-phenylpentan-3-yl)-1,4-diazepan-5-one was prepared according to General Procedure **A** using Zn (52 mg, 0.8 mmol, 2 equiv), 1,4-diazepan-5-one hydrochloride (60 mg, 0.4 mmol, 1 equiv), hydrocinnamaldehyde (79 µL, 0.6 mmol, 1.5 equiv), 2-iodopropane (100 µL, 1.2 mmol, 2.5 equiv), and TMSOTf (72 µL, 0.4 mmol, 1 equiv) in EtOAc (1 mL). Crude material was purified by column chromatography (0-2% MeOH in dichloromethane (with 5% Et_3N)) yielding the product as a colourless oil (72 mg, 66%).

^{1}H NMR (400 MHz, $CDCl_3$): δ 7.34 – 7.26 (m, 2H), 7.24 – 7.14 (m, 3H), 6.81 (s, 1H), 3.31 – 3.19 (m, 1H), 2.87 – 2.70 (m, 5H), 2.67 – 2.55 (m, 3H), 2.29 – 2.07 (m, 1H), 1.84 – 1.61 (m, 3H), 0.93 (d, J = 6.8 Hz, 3H), 0.91 (d, J = 6.8 Hz, 3H).

^{13}C NMR (101 MHz, $CDCl_3$): δ 178.6, 142.6, 128.3, 128.2, 125.7, 71.8, 54.0, 47.8, 44.5, 40.2, 34.4, 30.52, 30.47, 21.6, 20.4.

IR (film, cm^{-1}): 3227, 2952, 2808, 1662, 1354, 732, 697.

HRMS: m/z calcd for $C_{17}H_{27}N_2O$ $[M+H]^+$ 275.2118; found 275.2114.

3-(4-Methyl-1-phenylpentan-3-yl)-3-azabicyclo[3.1.0]hexane (4r)

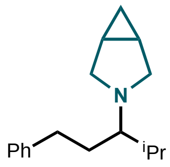

3-(4-Methyl-1-phenylpentan-3-yl)-3-azabicyclo[3.1.0]hexane was prepared according to General Procedure **A** using Zn (52 mg, 0.8 mmol, 2 equiv), 3-azabicyclo[3.1.0]hexane hydrochloride (48 mg, 0.4 mmol, 1 equiv), hydrocinnamaldehyde (79 µL, 0.6 mmol, 1.5 equiv), 2-iodopropane (100 µL, 1.2 mmol, 2.5 equiv), and TMSOTf (72 µL, 0.4 mmol, 1 equiv) in EtOAc (1 mL). Crude material was purified by column chromatography (0-2% MeOH in dichloromethane (with 5% Et_3N)) yielding the product as a colourless oil (63 mg, 65%). Data was in line with previous characterisation.[1]

2-(4-Methyl-1-phenylpentan-3-yl)octahydrocyclopenta[c]pyrrole (4s)

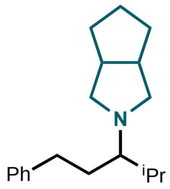

2-(4-Methyl-1-phenylpentan-3-yl)octahydrocyclopenta[c]pyrrole was prepared according to General Procedure **A** using Zn (52 mg, 0.8 mmol, 2 equiv), octahydrocyclopenta[c]pyrrole hydrochloride (60 mg, 0.4 mmol, 1 equiv), hydrocinnamaldehyde (79 µL, 0.6 mmol, 1.5 equiv), 2-iodopropane (100 µL, 1.2 mmol, 2.5 equiv), and TMSOTf (72 µL, 0.4 mmol, 1 equiv) in EtOAc (1 mL). Crude material was purified by column chromatography (0-3% MeOH in dichloromethane (with 5% Et_3N)) yielding the product as a colourless oil (89 mg, 82%).

^{1}H NMR (400 MHz, $CDCl_3$): δ 7.32 – 7.26 (m, 2H), 7.23 – 7.16 (m, 3H), 2.81 (q, J = 8.8 Hz, 2H), 2.77 – 2.68 (m, 1H), 2.67 – 2.58 (m, 1H), 2.58 – 2.47 (m, 2H), 2.22 (dd, J = 8.8, 3.5 Hz, 2H), 2.05 (q, J = 4.8 Hz, 1H), 1.97 – 1.84 (m, 1H), 1.75 – 1.62 (m, 5H), 1.52 – 1.37 (m, 3H), 0.92 (d, J = 6.9 Hz, 3H), 0.91 (d, J = 7.0 Hz, 3H).

^{13}C NMR (101 MHz, $CDCl_3$): δ 143.1, 128.3, 128.2, 125.6, 67.0, 58.8, 58.2, 41.9, 41.8, 35.4, 33.5, 33.4, 30.4, 30.2, 26.3, 20.4, 17.7.

IR (film, cm^{-1}): 2954, 2867, 1452, 1265, 1030, 730, 698.

HRMS: m/z calcd for $C_{19}H_{30}N$ $[M+H]^+$ 272.2373; found 272.2372.

6-(4-Methyl-1-phenylpentan-3-yl)-4,5,6,7-tetrahydrothieno[2,3-c]pyridine (4t)

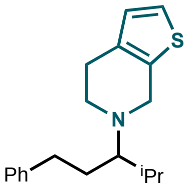

6-(4-Methyl-1-phenylpentan-3-yl)-4,5,6,7-tetrahydrothieno[2,3-c]pyridine was prepared according to General Procedure **A** using Zn (52 mg, 0.8 mmol, 2 equiv), 4,5,6,7-tetrahydrothieno[2,3-c]pyridine hydrochloride (70 mg, 0.4 mmol, 1 equiv), hydrocinnamaldehyde (79 µL, 0.6 mmol, 1.5 equiv), 2-iodopropane (100 µL, 1.2 mmol, 2.5 equiv), and TMSOTf (108 µL, 0.6 mmol, 1.5 equiv) in EtOAc (1 mL). Crude material was purified by column chromatography (0–50% dichloromethane in Pet. Ether (with 5% Et_3N)) yielding the product as a colourless oil (98 mg, 82%).

^{1}H NMR (400 MHz, $CDCl_3$): δ 7.34 – 7.28 (m, 2H), 7.24 – 7.18 (m, 3H), 7.09 (d, J = 5.1 Hz, 1H), 6.77 (d, J = 5.1 Hz, 1H), 3.82 – 3.70 (m, 2H), 2.97 (t, J = 5.4 Hz, 2H), 2.89 – 2.76 (m, 3H), 2.72 – 2.64 (m, 1H), 2.39 (td, J = 7.3, 4.5 Hz, 1H), 2.03 – 1.82 (m, 2H), 1.83 – 1.67 (m, 1H), 0.99 (d, J = 6.7 Hz, 6H).

^{13}C NMR (101 MHz, $CDCl_3$): δ 143.0, 135.0, 133.9, 128.4, 128.3, 125.6, 125.3, 122.1, 69.0, 49.2, 46.9, 34.8, 30.2, 30.1, 26.8, 21.5, 20.3.

IR (film, cm^{-1}): 2961, 2873, 1621, 1583, 1453, 1342, 1068, 732, 698, 594.

HRMS: m/z calcd for $C_{19}H_{26}NS$ $[M+H]^+$ 300.1781; found 300.1776.

8-(4-Methyl-1-phenylpentan-3-yl)-8-azabicyclo[3.2.1]octan-3-one (4u)

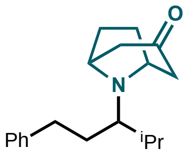

8-(4-Methyl-1-phenylpentan-3-yl)-8-azabicyclo[3.2.1]octan-3-one was prepared according to General Procedure **A** using Zn (52 mg, 0.8 mmol, 2 equiv), 8-azabicyclo[3.2.1]octan-3-one hydrochloride (65 mg, 0.4 mmol, 1 equiv), hydrocinnamaldehyde (79 µL, 0.6 mmol, 1.5 equiv), 2-iodopropane (100 µL, 1.2 mmol, 2.5 equiv), and TMSOTf (72 µL, 0.4 mmol, 1 equiv) in EtOAc (1 mL). Crude material was purified by column chromatography (0-10% MeOH in dichloromethane (with 5% Et_3N)) yielding the product as a colourless oil (72 mg, 63%).

^{1}H NMR (400 MHz, $CDCl_3$): δ 7.33 – 7.25 (m, 2H), 7.23 – 7.15 (m, 3H), 3.75 – 3.67 (m, 2H), 2.71 (t, J = 8.4 Hz, 2H), 2.64 – 2.53 (m, 3H), 2.14 (t, J = 16.3 Hz, 2H), 2.08 – 1.80 (m, 4H), 1.78 – 1.68 (m, 1H), 1.66 – 1.52 (m, 2H), 1.01 – 0.94 (m, 6H).

^{13}C NMR (101 MHz, $CDCl_3$): δ 210.7, 142.4, 128.4, 128.2, 125.9, 60.3, 56.9, 55.4, 46.7, 46.5, 35.1, 31.2, 30.1, 28.9, 28.8, 19.6, 16.6.

IR (film, cm^{-1}): 2954, 1711, 1452, 1346, 1192, 1073, 910, 750, 698.

HRMS: m/z calcd for $C_{19}H_{28}NO$ $[M+H]^+$ 286.2166; found 286.2168.

3-(4-Methyl-1-phenylpentan-3-yl)-8-oxa-3-azabicyclo[3.2.1]octane (4v)

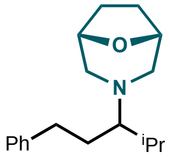

3-(4-Methyl-1-phenylpentan-3-yl)-8-oxa-3-azabicyclo[3.2.1]octane was prepared according to General Procedure **A** using Zn (52 mg, 0.8 mmol, 2 equiv), 8-oxa-3-azabicyclo[3.2.1]octane hydrochloride (60 mg, 0.4 mmol, 1 equiv), hydrocinnamaldehyde (79 µL, 0.6 mmol, 1.5 equiv), 2-iodopropane (100 µL, 1.2 mmol, 2.5 equiv), and TMSOTf (72 µL, 0.4 mmol, 1.0 equiv) in EtOAc (1 mL). Crude material was purified by column chromatography (0-2% MeOH in dichloromethane (with 5% Et_3N)) yielding the product as a colourless oil (85 mg, 78%).

^{1}H NMR (400 MHz, $CDCl_3$): δ 7.34 – 7.28 (m, 2H), 7.25 – 7.18 (m, 3H), 4.33 – 4.26 (m, 2H), 2.88 (t, J = 10.1 Hz, 2H), 2.80 (td, J = 10.6, 9.7, 5.2 Hz, 1H), 2.71 – 2.57 (m, 1H), 2.42 (t, J = 11.5 Hz, 2H), 2.07 – 1.95 (m, 3H), 1.93 – 1.71 (m, 4H), 1.68 – 1.58 (m, 1H), 0.96 (d, J = 6.8 Hz, 3H), 0.93 (d, J = 6.7 Hz, 3H).

^{13}C NMR (101 MHz, $CDCl_3$): δ 142.9, 128.3, 128.3, 125.7, 75.3, 75.2, 68.4, 54.9, 54.4, 34.8, 29.73, 29.69, 28.52, 28.47, 21.3, 20.2.

IR (film, cm^{-1}): 2947, 2868, 2807, 1453, 1066, 993, 873, 749, 697.

HRMS: m/z calcd for $C_{18}H_{28}NO$ $[M+H]^+$ 274.2166; found 274.2164.

8-(4-Methyl-1-phenylpentan-3-yl)-1-phenyl-1,3,8-triazaspiro[4.5]decan-4-one (4w)

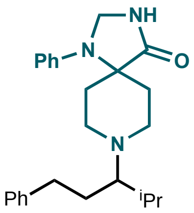

8-(4-Methyl-1-phenylpentan-3-yl)-1-phenyl-1,3,8-triazaspiro[4.5]decan-4-one was prepared according to General Procedure **B** using Zn (52 mg, 0.8 mmol, 2 equiv), 8-azabicyclo[3.2.1]octan-3-ol (93 mg, 0.4 mmol, 1 equiv), hydrocinnamaldehyde (79 µL, 0.6 mmol, 1.5 equiv), 2-iodopropane (100 µL, 1.2 mmol, 2.5 equiv), and TMSOTf (108 µL, 0.6 mmol, 1.5 equiv) in EtOAc (1 mL). Crude material was purified by column chromatography (0-2% MeOH in dichloromethane (with 5% Et_3N)) yielding the product as a white solid (128 mg, 82%).

^{1}H NMR (400 MHz, $CDCl_3$): δ 8.02 (s, 1H), 7.38 – 7.20 (m, 7H), 7.01 (d, J = 8.2 Hz, 2H), 6.87 (t, J = 7.3 Hz, 1H), 4.80 (s, 2H), 3.26 (tt, J = 9.4, 5.3 Hz, 2H), 3.01 – 2.67 (m, 6H), 2.31 (td, J = 7.6, 4.4 Hz, 1H), 2.00 – 1.82 (m, 2H), 1.81 – 1.67 (m, 3H), 1.04 (d, J = 6.8 Hz, 3H), 1.00 (d, J = 6.6 Hz, 3H).

^{13}C NMR (101 MHz, $CDCl_3$): δ 179.1, 143.21, 143.17, 129.1, 128.4, 128.3, 125.6, 118.0, 114.2, 69.6, 59.7, 59.4, 46.11, 46.06, 34.8, 29.80, 29.76, 29.6, 21.6, 20.3.

IR (film, cm^{-1}): 3204, 3025, 2951, 2862, 1704, 1599, 1501, 1368, 1263, 736, 696.

HRMS: m/z calcd for $C_{25}H_{34}N_3O$ $[M+H]^+$ 392.2697; found 392.2694.

N,4-dimethyl-1-phenylpentan-3-amine (6a)

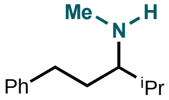

N,4-dimethyl-1-phenylpentan-3-amine was synthesized according to General Procedure **A** using Zn (52 mg, 0.8 mmol, 2 equiv), methylamine hydrochloride (27 mg, 0.4 mmol, 1 equiv), hydrocinnamaldehyde (79 µL, 0.6 mmol, 1.5 equiv), 2-iodopropane (100 µL, 1 mmol, 2.5 equiv) and TMSOTf (72 µL, 0.4 mmol, 1 equiv) in EtOAc (1 mL). Crude material was purified by column chromatography (2-6% MeOH in dichloromethane (with 5% Et_3N)) yielding the product as a colourless oil (62 mg, 81%).

^{1}H NMR (400 MHz, $CDCl_3$) δ 7.35 – 7.27 (m, 2H), 7.26 – 7.18 (m, 3H), 2.80 – 2.69 (m, 1H), 2.69 – 2.60 (m, 1H), 2.44 (s, 3H), 2.32 – 2.22 (m, 1H), 1.96 – 1.85 (m, 1H), 1.81 – 1.70 (m, 1H), 1.69 – 1.57 (m, 1H), 1.36 (brs, 1H), 0.99 – 0.88 (m, 6H).

^{13}C NMR (101 MHz, $CDCl_3$) δ 142.7, 128.27, 128.28, 125.6, 64.6, 34.4, 32.9, 32.1, 29.4, 18.6, 18.0.

IR (film, cm^{-1}): 2953, 2869, 2789, 1453, 747, 697.

HRMS: m/z calcd for $C_{13}H_{22}N$ $[M+H]^+$ 192.1747; found 192.1744.

N-Benzyl-4-methyl-1-phenylpentan-3-amine (6b)

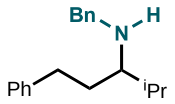

N-Benzyl-4-methyl-1-phenylpentan-3-amine was prepared according to General Procedure **B** using Zn (52 mg, 0.8 mmol, 2 equiv), benzylamine (44 µL, 0.4 mmol, 1 equiv), hydrocinnamaldehyde (79 µL, 0.6 mmol, 1.5 equiv), 2-iodopropane (100 µL, 1.2 mmol, 2.5 equiv), and TMSOTf (108 µL, 0.6 mmol, 1.5 equiv) in EtOAc (1 mL). Crude material was purified by column chromatography (0-5% MeOH in dichloromethane (with 5% Et_3N)) yielding the product as a colourless oil (58 mg, 54%). 56% assay yield by crude 1H NMR analysis using 1,1,2,2-tetrachloroethane.

Note: *Compound is at highest achievable purity, further attempts at purification or use of acidic purification methods leads to degradation.*

^{1}H NMR (400 MHz, $CDCl_3$): δ 7.43 – 7.37 (m, 3H), 7.37 – 7.30 (m, 4H), 7.27 – 7.21 (m, 3H), 3.84 (s, 2H), 2.87 – 2.76 (m, 1H), 2.74 – 2.64 (m, 1H), 2.50 – 2.43 (m, 1H), 2.05 – 1.91 (m, 1H), 1.90 – 1.77 (m, 1H), 1.77 – 1.63 (m, 1H), 1.39 (bs, 1H), 0.98 (d, J = 6.9 Hz, 6H).

^{13}C NMR (101 MHz, $CDCl_3$): δ 142.8, 141.2, 128.34, 128.27, 128.2, 126.8, 125.6, 61.9, 51.8, 32.8, 32.5, 29.8, 18.7, 18.0.

IR (film, cm^{-1}): 3025, 2953, 2868, 1494, 1452, 741, 695.

HRMS: m/z calcd for $C_{19}H_{26}N$ $[M+H]^+$ 268.2060; found 268.2057.

N-(but-3-en-1-yl)-4-methyl-1-phenylpentan-3-amine (6c)

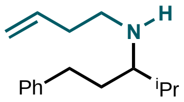

N-(but-3-en-1-yl)-4-methyl-1-phenylpentan-3-amine was prepared according to General Procedure **A** using Zn (52 mg, 0.8 mmol, 2 equiv), but-3-en-1-amine hydrochloride (43 mg, 0.4 mmol, 1 equiv), hydrocinnamaldehyde (79 µL, 0.6 mmol, 1.5 equiv), 2-iodopropane (100 µL, 1.2 mmol, 2.5 equiv), and TMSOTf (72 µL, 0.4 mmol, 1 equiv) in EtOAc (1 mL). Crude material was purified by column chromatography (0-2% MeOH in dichloromethane (with 5% Et_3N)) yielding the product as a colourless oil (40 mg, 44%).

^{1}H NMR (400 MHz, $CDCl_3$): δ 7.33 – 7.25 (m, 2H), 7.23 – 7.15 (m, 3H), 5.82 (ddt, J = 17.0, 10.3, 6.8 Hz, 1H), 5.11 (dt, J = 17.1, 1.7 Hz, 1H), 5.06 (d, J = 10.2 Hz, 1H), 2.79 – 2.55 (m, 4H), 2.34 – 2.29 (m, 1H), 2.25 (q, J = 6.8 Hz, 2H), 1.84 (dq, J = 13.3, 6.7 Hz, 1H), 1.80 – 1.69 (m, 1H), 1.66 – 1.54 (m, 1H), 1.21 (bs, 1H), 0.90 (d, J = 6.8 Hz, 3H), 0.90 (d, J = 6.8 Hz, 3H).

^{13}C NMR (101 MHz, $CDCl_3$): δ 142.8, 136.8, 128.31, 128.27, 125.6, 116.1, 62.7, 46.8, 34.7, 32.9, 32.6, 30.0, 18.7, 18.2.

IR (film, cm^{-1}): 2954, 2927, 1639, 1453, 911, 746, 697.

HRMS: m/z calcd for $C_{16}H_{26}N$ $[M+H]^+$ 232.2060; found 232.2062.

N-(2-Methoxyethyl)-4-methyl-1-phenylpentan-3-amine (6d)

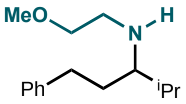

N-(2-Methoxyethyl)-4-methyl-1-phenylpentan-3-amine was prepared according to General Procedure **B** using Zn (52 mg, 0.8 mmol, 2 equiv), 2-methoxyethan-1-amine (35 µL, 0.4 mmol, 1 equiv), hydrocinnamaldehyde (79 µL, 0.6 mmol, 1.5 equiv), 2-iodopropane (100 µL, 1.2 mmol, 2.5 equiv), and TMSOTf (108 µL, 0.6 mmol, 1.5 equiv) in EtOAc (1 mL). Crude material was purified by column chromatography (0-2% MeOH in dichloromethane (with 5% Et_3N)) yielding the product as a colourless oil (62 mg, 66%).

^{1}H NMR (400 MHz, $CDCl_3$): δ 7.35 – 7.27 (m, 2H), 7.26 – 7.17 (m, 3H), 3.52 (t, J = 5.3 Hz, 2H), 3.40 (s, 3H), 2.84 – 2.72 (m, 3H), 2.71 – 2.58 (m, 1H), 2.39 – 2.19 (m, 1H), 1.92 – 1.71 (m, 2H), 1.70 – 1.47 (m, 2H), 0.94 (d, J = 6.8 Hz, 3H), 0.93 (d, J = 6.8 Hz, 3H).

^{13}C NMR (101 MHz, $CDCl_3$): δ 142.8, 128.32, 128.25, 125.6, 72.4, 62.7, 58.7, 47.2, 32.8, 32.7, 30.1, 18.7, 18.1.

IR (film, cm^{-1}): 2953, 2871, 1495, 1453, 1115, 1029, 748, 697.

HRMS: m/z calcd for $C_{15}H_{26}NO$ $[M+H]^+$ 236.2009; found 236.2005.

N-(2,2-Difluoroethyl)-4-methyl-1-phenylpentan-3-amine (6e)

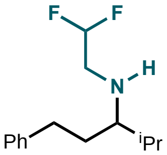

N-(2,2-Difluoroethyl)-4-methyl-1-phenylpentan-3-amine was prepared according to General Procedure **B** using Zn (52 mg, 0.8 mmol, 2 equiv), 2,2-difluoroethan-1-amine (28 µL, 0.4 mmol, 1 equiv), hydrocinnamaldehyde (79 µL, 0.6 mmol, 1.5 equiv), 2-iodopropane (100 µL, 1.2 mmol, 2.5 equiv), and TMSOTf (108 µL, 0.6 mmol, 1.5 equiv) in EtOAc (1 mL). Crude material was purified by column chromatography (50-100% Pet. Ether in dichloromethane (with 5% Et_3N)) yielding the product as a colourless oil (51 mg, 53%).

^{1}H NMR (400 MHz, $CDCl_3$): δ 7.34 – 7.27 (m, 2H), 7.24 – 7.18 (m, 3H), 5.80 (tt, J = 56.8, 4.5 Hz, 1H), 2.96 (tt, J = 14.8, 4.8 Hz, 2H), 2.83 – 2.71 (m, 1H), 2.68 – 2.58 (m, 1H), 2.35 (dt, J = 8.6, 4.5 Hz, 1H), 1.87 – 1.69 (m, 2H), 1.63 – 1.51 (m, 1H), 1.37 – 1.17 (m, 1H), 0.91 (d, J = 7.0 Hz, 6H).

^{13}C NMR (101 MHz, $CDCl_3$): δ 142.4, 128.4, 128.3, 125.8, 116.40 (t, J = 240.2 Hz), 62.6, 49.73 (t, J = 24.7 Hz), 32.7, 32.6, 30.2, 18.5, 17.9.

^{19}F NMR (376 MHz, $CDCl_3$): δ -122.80.

IR (film, cm^{-1}): 2957, 2871, 1453, 1111, 1051, 739, 698.

HRMS: m/z calcd for $C_{14}H_{22}F_2N$ $[M+H]^+$ 242.1715; found 242.1717.

4-Methyl-1-phenyl-*N*-(2-(pyridin-4-yl)ethyl)pentan-3-amine (6f)

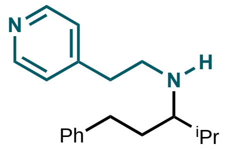

4-Methyl-1-phenyl-*N*-(2-(pyridin-4-yl)ethyl)pentan-3-amine was prepared according to General Procedure **B** using Zn (52 mg, 0.8 mmol, 2 equiv), 4-(2-Aminoethyl)pyridine (47 µL 0.4 mmol, 1 equiv), hydrocinnamaldehyde (79 µL, 0.6 mmol, 1.5 equiv), 2-iodopropane (100 µL, 1.2 mmol, 2.5 equiv), and TMSOTf (144 µL, 0.8 mmol, 2 equiv) in EtOAc (1 mL). Crude material was purified by column chromatography (0-2% MeOH in dichloromethane (with 5% Et_3N)) yielding the product as a pale-yellow oil (86 mg, 76%).

^{1}H NMR (400 MHz, $CDCl_3$): δ 8.48 (dd, J = 11.7, 3.5 Hz, 2H), 7.55 (dd, J = 7.9, 1.8 Hz, 1H), 7.32 – 7.10 (m, 6H), 2.91 – 2.83 (m, 2H), 2.79 – 2.73 (m, 2H), 2.70 – 2.52 (m, 2H), 2.36 – 2.26 (m, 1H), 1.87 – 1.78 (m, 1H), 1.77 – 1.67 (m, 1H), 1.63 – 1.50 (m, 1H), 1.41 (bs, 1H), 0.86 (d, J = 6.1 Hz, 3H), 0.85 (d, J = 6.1 Hz, 3H).

^{13}C NMR (101 MHz, $CDCl_3$): δ 150.2, 147.6, 142.6, 136.1, 135.6, 128.27, 128.26, 125.6, 123.2, 62.6, 48.6, 34.0, 32.8, 32.5, 29.9, 18.5, 18.0.

IR (film, cm^{-1}): 2952, 2867, 1477, 1453, 1106, 1028, 748, 698.

HRMS: m/z calcd for $C_{19}H_{27}N_2$ $[M+H]^+$ 283.2169; found 283.2163.

N-(4-Methyl-1-phenylpentan-3-yl)tetrahydro-2H-pyran-4-amine (6g)

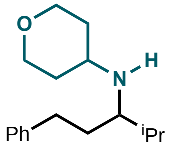

N-(4-Methyl-1-phenylpentan-3-yl)tetrahydro-2H-pyran-4-amine was prepared according to General Procedure **B** using Zn (52 mg, 0.8 mmol, 2 equiv), tetrahydro-2H-pyran-4-amine (41 µL, 0.4 mmol, 1 equiv), hydrocinnamaldehyde (79 µL, 0.6 mmol, 1.5 equiv), 2-iodopropane (100 µL, 1.2 mmol, 2.5 equiv), and TMSOTf (108 µL, 0.6 mmol, 1.5 equiv) in EtOAc (1 mL). Crude material was purified by column chromatography (0-2% MeOH in dichloromethane (with 5% Et_3N)) yielding the product as a colourless oil (75 mg, 72%).

^{1}H NMR (400 MHz, $CDCl_3$): δ 7.34 – 7.28 (m, 2H), 7.26 – 7.16 (m, 3H), 3.98 (dd, J = 11.9, 3.6 Hz, 2H), 3.40 (tq, J = 11.6, 2.2 Hz, 2H), 2.81 – 2.59 (m, 3H), 2.47 (dt, J = 8.2, 4.5 Hz, 1H), 1.91 – 1.71 (m, 4H), 1.63 – 1.50 (m, 1H), 1.38 (dtd, J = 14.1, 11.0, 4.3 Hz, 2H), 0.92 (d, J = 6.8 Hz, 6H).

^{13}C NMR (101 MHz, $CDCl_3$): δ 142.7, 128.30, 128.26, 125.6, 66.8, 66.8, 58.6, 51.4, 34.5, 34.4, 33.1, 32.9, 30.2, 18.5, 17.9.

IR (film, cm^{-1}): 2952, 2841, 1453, 1366, 1140, 1087, 867, 747, 697.

HRMS: m/z calcd for $C_{17}H_{28}NO$ $[M+H]^+$ 262.2166; found 262.2169.

Benzyl 4-((4-methyl-1-phenylpentan-3-yl)amino)piperidine-1-carboxylate (6h)

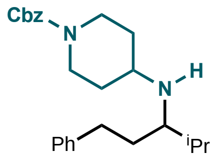

Benzyl 4-((4-methyl-1-phenylpentan-3-yl)amino)piperidine-1-carboxylate was prepared according to the general procedure **B** using Zn (52 mg, 0.8 mmol, 2 equiv), benzyl 4-aminopiperidine-1-carboxylate (94 mg, 0.4 mmol, 1 equiv), hydrocinnamaldehyde (79 µL, 0.6 mmol, 1.5 equiv), 2-iodopropane (100 µL, 1.2 mmol, 2.5 equiv), and TMSOTf (108 µL, 0.6 mmol, 1.5 equiv) in EtOAc (1 mL). Crude material was purified by column chromatography (0-2% MeOH in dichloromethane (with 5% Et_3N)) yielding the product as a colourless oil (146 mg, 93%).

^{1}H NMR (400 MHz, $CDCl_3$): δ 7.41 – 7.36 (m, 4H), 7.36 – 7.28 (m, 3H), 7.24 – 7.17 (m, 3H), 5.16 (bs, 2H), 4.15 – 3.99 (m, 2H), 2.95 (t, J = 12.3 Hz, 2H), 2.80 – 2.56 (m, 3H), 2.44 (dt, J = 8.3, 4.5 Hz, 1H), 1.90 – 1.66 (m, 4H), 1.60 – 1.47 (m, 1H), 1.27 (q, J = 11.7 Hz, 2H), 0.91 (d, J = 6.8 Hz, 6H).

^{13}C NMR (101 MHz, $CDCl_3$): δ 155.1, 142.6, 136.9, 128.3, 128.2, 128.2, 127.8, 127.7, 125.6, 66.9, 58.7, 51.9, 42.57, 42.55, 33.0, 32.8, 30.0, 18.4, 17.8.

IR (film, cm^{-1}): 2941, 2867, 1692, 1429, 1224, 732, 696.

HRMS: m/z calcd for $C_{25}H_{35}N_2O_2$ $[M+H]^+$ 395.2630; found 395.2694.

1-Ethynyl-N-(4-methyl-1-phenylpentan-3-yl)cyclohexan-1-amine (6i)

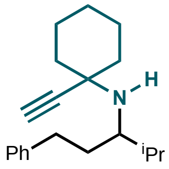

1-Ethynyl-N-(4-methyl-1-phenylpentan-3-yl)cyclohexan-1-amine was prepared according to General Procedure **B** using Zn (52 mg, 0.8 mmol, 2 equiv), 1-ethynylcyclohexan-1-amine (54 µL, 0.4 mmol, 1 equiv), hydrocinnamaldehyde (79 µL, 0.6 mmol, 1.5 equiv), 2-iodopropane (100 µL, 1.2 mmol, 2.5 equiv), and TMSOTf (108 µL, 0.6 mmol, 1.5 equiv) in EtOAc (1 mL). Crude material was purified by column chromatography (0-2% MeOH in dichloromethane (with 5% Et_3N)) yielding the product as a colourless oil (78 mg, 69%).

^{1}H NMR (400 MHz, $CDCl_3$): δ 7.31 – 7.26 (m, 2H), 7.23 – 7.15 (m, 3H), 2.82 – 2.70 (m, 2H), 2.69 – 2.59 (m, 1H), 2.32 (s, 1H), 1.99 – 1.90 (m, 1H), 1.85 – 1.74 (m, 3H), 1.73 – 1.52 (m, 6H), 1.45 – 1.34 (m, 2H), 1.25 – 1.14 (m, 1H), 0.92 (d, J = 6.8 Hz, 3H), 0.90 (d, J = 6.9 Hz, 3H).

^{13}C NMR (101 MHz, $CDCl_3$): δ 143.1, 128.3, 128.2, 125.5, 89.2, 72.0, 57.6, 54.2, 39.42, 39.39, 34.4, 33.1, 31.3, 25.7, 22.82, 22.78, 18.5, 17.9.

IR (film, cm^{-1}): 3302, 3025, 2930, 2855, 1449, 1112, 748, 697, 626, 556.

HRMS: m/z calcd for $C_{20}H_{30}N$ $[M+H]^+$ 284.2373; found 284.2380.

N-(4-Methyl-1-phenylpentan-3-yl)adamantan-1-amine (6j)

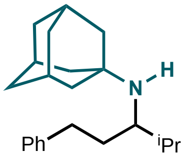

N-(4-Methyl-1-phenylpentan-3-yl)adamantan-1-amine was prepared according to General Procedure **B** using Zn (52 mg, 0.8 mmol, 2 equiv), (3*s*,5*s*,7*s*)-adamantan-1-amine (60 mg, 0.4 mmol, 1 equiv), hydrocinnamaldehyde (79 µL, 0.6 mmol, 1.5 equiv), 2-iodopropane (100 µL, 1.2 mmol, 2.5 equiv), and TMSOTf (108 µL, 0.6 mmol, 1.5 equiv) in EtOAc (1 mL). Crude material was purified by column chromatography (0-2% MeOH in dichloromethane (with 5% Et_3N)) yielding the product as a colourless oil (87 mg, 70%).

^{1}H NMR (400 MHz, $CDCl_3$): δ 7.29 (t, J = 7.5 Hz, 2H), 7.23 – 7.15 (m, 3H), 2.74 – 2.65 (m, 1H), 2.64 – 2.55 (m, 2H), 2.10 – 2.00 (m, 3H), 1.84 – 1.48 (m, 14H), 0.91 (d, J = 6.9 Hz, 3H), 0.89 (d, J = 6.9 Hz, 6H).

^{13}C NMR (101 MHz, $CDCl_3$): δ 143.0, 128.3, 128.2, 125.5, 54.2, 50.7, 44.3, 36.7, 35.6, 33.2, 31.9, 29.7, 18.3, 18.2.

IR (film, cm^{-1}): 2900, 2846, 1451, 1142, 1096, 738, 697.

HRMS: m/z calcd for $C_{22}H_{34}N$ $[M+H]^+$ 312.2686; found 312.2687.

N-(4-Methyl-1-phenylpentan-3-yl)aniline (7a)

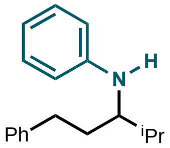

N-(4-Methyl-1-phenylpentan-3-yl)aniline was prepared according to General Procedure **B** using Zn (52 mg, 0.8 mmol, 2 equiv), aniline (36 µL, 0.4 mmol, 1 equiv), hydrocinnamaldehyde (79 µL, 0.6 mmol, 1.5 equiv), 2-iodopropane (100 µL, 1.2 mmol, 2.5 equiv), and TMSOTf (144 µL, 0.8 mmol, 2 equiv) in EtOAc (1 mL). Crude material was purified by column chromatography (0-1% MeOH in dichloromethane (with 5% Et_3N)) yielding the product as a colourless oil (54 mg, 54%).

^{1}H NMR (400 MHz, $CDCl_3$): δ 7.32 – 7.25 (m, 2H), 7.24 – 7.13 (m, 5H), 6.66 (t, J = 7.3 Hz, 1H), 6.58 (d, J = 8.0 Hz, 2H), 3.49 (bs, 1H), 3.28 (dt, J = 8.7, 4.2 Hz, 1H), 2.86 – 2.76 (m, 1H), 2.71 – 2.59 (m, 1H), 2.01 – 1.84 (m, 2H), 1.74 – 1.58 (m, 1H), 0.95 (d, J = 6.9 Hz, 3H), 0.93 (d, J = 6.7 Hz, 3H).

^{13}C NMR (101 MHz, $CDCl_3$): δ 148.5, 142.3, 129.3, 128.4, 128.3, 125.8, 116.5, 112.9, 57.6, 33.5, 32.8, 31.2, 18.4, 18.3.

IR (film, cm^{-1}): 3403, 2955, 2869, 1598, 1503, 1495, 1453, 1319, 1251, 743, 690.

HRMS: m/z calcd for $C_{26}H_{32}N$ $[M+H]^+$ 254.1904; found: 254.1899.

4-Iodo-N-(4-methyl-1-phenylpentan-3-yl)aniline (7b)

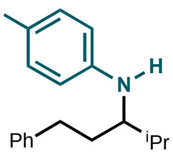

4-Iodo-N-(4-methyl-1-phenylpentan-3-yl)aniline was prepared according to General Procedure **B** using Zn (52 mg, 0.8 mmol, 2 equiv), 4-iodoaniline (88 mg, 0.4 mmol, 1 equiv), hydrocinnamaldehyde (79 µL, 0.6 mmol, 1.5 equiv), 2-iodopropane (100 µL, 1.2 mmol, 2.5 equiv), and TMSOTf (144 µL, 0.8 mmol, 2 equiv) in EtOAc (1 mL). Crude material was purified by column chromatography (0-1% MeOH in dichloromethane (with 5% Et_3N)) yielding the product as a colourless oil (48 mg, 32%).

^{1}H NMR (400 MHz, $CDCl_3$): δ 7.13 (d, J = 8.5 Hz, 2H), 7.05 – 6.99 (m, 2H), 6.97 – 6.87 (m, 3H), 6.08 (d, J = 8.3 Hz, 2H), 3.26 (bs, 1H), 2.94 (dt, J = 8.8, 4.2 Hz, 1H), 2.55 – 2.46 (m, 1H), 2.42 – 2.33 (m, 1H), 1.71 – 1.56 (m, 2H), 1.46 – 1.31 (m, 1H), 0.67 (d, J = 7.2 Hz, 3H), 0.65 (d, J = 7.9 Hz, 3H).

^{13}C NMR (101 MHz, $CDCl_3$): δ 148.1, 142.0, 137.7, 128.38, 128.36, 125.8, 115.1, 57.5, 33.5, 32.7, 31.3, 18.4, 18.2.

IR (film, cm^{-1}): 3404, 3082, 2954, 2868, 1587, 1573, 1492, 1452, 1317, 1292, 806, 745, 697.

HRMS: m/z calcd for $C_{18}H_{23}IN$ $[M+H]^+$ 380.0870; found 380.0869.

4-Methoxy-N-(4-methyl-1-phenylpentan-3-yl)aniline (7c)

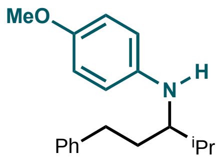

4-Methoxy-N-(4-methyl-1-phenylpentan-3-yl)aniline was prepared according to General Procedure **B** using Zn (52 mg, 0.8 mmol, 2 equiv), 4-methoxyaniline (49 mg, 0.4 mmol, 1 equiv), hydrocinnamaldehyde (79 µL, 0.6 mmol, 1.5 equiv), 2-iodopropane (100 µL, 1.2 mmol, 2.5 equiv), and TMSOTf (144 µL, 0.8 mmol, 2 equiv) in EtOAc (1 mL). Crude material was purified by column chromatography (50-100% Pet. Ether in dichloromethane (with 5% Et_3N)) yielding the product as a colourless oil (58 mg, 52%).

^{1}H NMR (400 MHz, $CDCl_3$): δ 7.37 – 7.28 (m, 2H), 7.27 – 7.18 (m, 3H), 6.84 – 6.78 (m, 2H), 6.57 (d, J = 8.8 Hz, 2H), 3.79 (s, 3H), 3.35 – 3.15 (m, 2H), 2.90 – 2.79 (m, 1H), 2.74 – 2.64 (m, 1H), 2.03 – 1.85 (m, 2H), 1.74 – 1.60 (m, 1H), 0.98 (d, J = 6.9 Hz, 3H), 1.02 – 0.91 (m, 6H).

^{13}C NMR (101 MHz, $CDCl_3$): δ 151.5, 142.8, 142.4, 128.4, 128.3, 125.7, 115.0, 114.2, 58.6, 55.8, 33.4, 32.9, 31.0, 18.4, 18.1.

IR (film, cm^{-1}): 3025, 2953, 2829, 1508, 1453, 1230, 1038, 815, 748, 698.

HRMS: m/z calcd for $C_{19}H_{26}NO$ $[M+H]^+$ 284.2009; found 284.2005.

2,4,6-trimethyl-N-(4-methyl-1-phenylpentan-3-yl)aniline (7d)

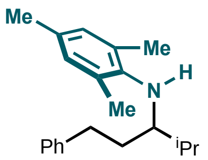

2,4,6-trimethyl-N-(4-methyl-1-phenylpentan-3-yl)aniline was prepared according to General Procedure **B** using Zn (52 mg, 0.8 mmol, 2 equiv), 2,4,6-trimethylaniline (56 µL, 0.4 mmol, 1 equiv), benzyl hydrocinnamaldehyde (79 µL, 0.6 mmol, 1.5 equiv), 2-iodopropane (100 µL, 1.2 mmol, 2.5 equiv), and TMSOTf (144 µL, 0.6 mmol, 2 equiv) in EtOAc (1 mL). Crude material was purified by column chromatography (50–100% Pet. Ether in dichloromethane with (5% Et_3N)) yielding the product as a colourless oil (50 mg, 43%).

^{1}H NMR (400 MHz, $CDCl_3$): δ 7.34 – 7.27 (m, 2H), 7.21 (t, J = 7.3 Hz, 1H), 7.18 – 7.13 (m, 2H), 6.82 (s, 2H), 3.25 (dt, J = 8.0, 4.1 Hz, 1H), 3.01 (bs, 1H), 2.87 – 2.75 (m, 1H), 2.72 – 2.55 (m, 1H), 2.26 (s, 3H), 2.25 (s, 6H), 1.96 – 1.77 (m, 2H), 1.69 – 1.56 (m, 1H), 1.02 (d, J = 6.8 Hz, 3H), 0.98 (d, J = 7.0, 1.3 Hz, 3H).

^{13}C NMR (101 MHz, $CDCl_3$): δ 142.5, 129.8, 129.4, 128.30, 128.25, 127.7, 125.7, 60.0, 33.8, 33.2, 30.7, 20.4, 19.3, 18.1, 18.0.

IR (film, cm^{-1}): 2954, 2868, 1482, 1453, 1264, 854, 734, 697, 561.

HRMS: m/z calcd for $C_{21}H_{30}N$ $[M+H]^+$ 296.2373; found 296.2373.

(1*S*,4*S*)-4-(3,4-Dichlorophenyl)-N-methyl-N-(4-methyl-1-phenylpentan-3-yl)-1,2,3,4 tetrahydronaphthalen-1-amine (8a)

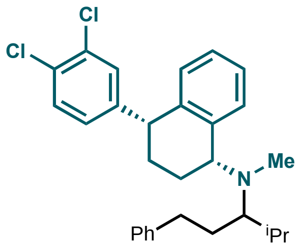

(1*S*,4*S*)-4-(3,4-Dichlorophenyl)-N-methyl-N-(4-methyl-1-phenylpentan-3-yl)-1,2,3,4 tetrahydronaphthalen-1-amine was prepared according to General Procedure **A** using Zn (52 mg, 0.8 mmol, 2 equiv), sertraline hydrochloride (137 mg, 0.4 mmol, 1 equiv), hydrocinnamaldehyde (79 µL, 0.6 mmol, 1.5 equiv), 2-iodopropane (100 µL, 1.2 mmol, 2.5 equiv), and TMSOTf (72 µL, 0.4 mmol, 1 equiv) in EtOAc (1 mL). Crude material was purified by column chromatography (0-2% MeOH in dichloromethane (with 5% Et_3N)) yielding the product as a colourless oil (115 mg, 62%, 2:1 d.r.).

Major isomer:

^{1}H NMR (400 MHz, $CDCl_3$): δ 7.89 (d, J = 7.9 Hz, 1H), 7.33 – 7.25 (m, 4H), 7.21 – 7.11 (m, 5H), 6.89 (d, J = 7.7 Hz, 1H), 6.82 (dt, J = 8.4, 1.5 Hz, 1H), 4.12 (t, J = 4.6 Hz, 1H), 3.93 (dd, J = 10.2, 5.5 Hz, 1H), 2.69 (t, J = 8.4 Hz, 2H), 2.55 (q, J = 5.6 Hz, 1H), 2.23 – 2.08 (m, 4H), 2.04 – 1.70 (m, 5H), 1.66 – 1.52 (m, 1H), 1.07 (d, J = 6.8 Hz, 3H), 1.05 (d, J = 6.9 Hz, 3H).

^{13}C NMR (101 MHz, $CDCl_3$): δ 147.7, 142.8, 140.2, 138.1, 132.1, 130.8, 130.2, 129.9, 129.8, 128.7, 128.33, 128.27, 128.2, 126.9, 126.6, 125.7, 67.1, 63.4, 43.7, 35.3, 32.2, 31.9, 30.7, 30.2, 20.1, 19.7, 18.8.

Minor isomer:

^{1}H NMR (400 MHz, $CDCl_3$) δ 7.79 (d, J = 7.8 Hz, 1H), 7.39 – 7.22 (m, 7H), 7.22 – 7.16 (m, 2H), 6.96 – 6.86 (m, 2H), 4.13 (t, J = 5.3 Hz, 1H), 3.98 (dd, J = 9.2, 5.1 Hz, 1H), 2.99 – 2.86 (m, 1H), 2.83 – 2.70 (m, 1H), 2.65 – 2.55 (m, 1H), 2.22 (s, 3H), 2.20 – 2.01 (m, 3H), 2.02 – 1.86 (m, 2H), 1.82 – 1.69 (m, 1H), 1.73 – 1.61 (m, 1H), 1.01 (d, J = 6.7 Hz, 3H), 0.98 (d, J = 7.0 Hz, 3H).

^{13}C NMR (101 MHz, $CDCl_3$) δ 147.8, 143.1, 140.1, 138.5, 132.1, 130.8, 130.21, 129.9, 129.8, 129.1, 128.4, 128.3, 128.2, 126.8, 126.6, 125.7, 67.1, 61.3, 43.9, 35.2, 31.5, 31.2, 30.6, 29.9, 21.8, 21.2, 19.1.

IR (film, cm^{-1}): 2936, 2866, 1466, 1385, 1130, 1028, 823, 737, 698.

HRMS: m/z calcd for $C_{29}H_{34}Cl_2N$ $[M+H]^+$ 466.2063; found 466.2062.

11-(4-(4-Methyl-1-phenylpentan-3-yl)piperazin-1-yl)dibenzo[b,f][1,4]thiazepine (8b)

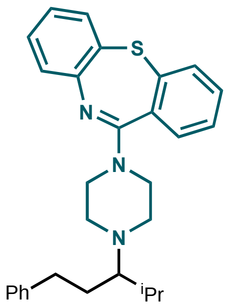

11-(4-(4-Methyl-1-phenylpentan-3-yl)piperazin-1-yl)dibenzo[b,f][1,4]thiazepine was prepared according to General Procedure **B** using Zn (52 mg, 0.8 mmol, 2 equiv), norquetiapine (118 mg, 0.4 mmol, 1 equiv), hydrocinnamaldehyde (79 µL, 0.6 mmol, 1.5 equiv), 2-iodopropane (100 µL, 1.2 mmol, 2.5 equiv), and TMSOTf (108 µL, 0.6 mmol, 1.5 equiv) in EtOAc (1 mL). Crude material was purified by column chromatography (0-1% MeOH in dichloromethane (with 5% Et_3N)) yielding the product as a colourless oil (102 mg, 56%).

^{1}H NMR (500 MHz, $CDCl_3$): δ 7.57 (dd, J = 7.4, 2.2 Hz, 1H), 7.46 (dd, J = 7.8, 3.1 Hz, 1H), 7.38 – 7.32 (m, 5H), 7.29 – 7.22 (m, 4H), 7.16 (t, J = 6.0 Hz, 1H), 6.98 – 6.88 (m, 1H), 3.82 – 3.20 (m, 3H), 3.00 – 2.62 (m, 7H), 2.29 – 2.22 (m, 1H), 2.05 – 1.81 (m, 2H), 1.76 – 1.65 (m, 1H), 1.10 – 0.95 (m, 6H).

^{13}C NMR (126 MHz, $CDCl_3$): δ 161.2, 149.1, 143.0, 140.0, 134.4, 132.2, 132.1, 130.7, 129.2, 129.1, 128.5, 128.4, 128.3, 128.1, 125.7, 125.4, 122.7, 69.31, 69.28, 49.2, 34.8, 34.7, 29.7, 29.6, 21.63, 21.58, 20.2.

IR (film, cm^{-1}): 3035, 2957, 2870, 1588, 1573, 1493, 1467, 1282, 1260, 1032, 746, 696.

HRMS: m/z calcd for $C_{29}H_{34}N_3S$ $[M+H]^+$ 456.2468; found 456.2472.

Ethyl-1-cyclopropyl-6-fluoro-7-(4-(4-methyl-1-phenylpentan-3-yl)piperazin-1-yl)-4-oxo-1,4-dihydroquinoline-3-carboxylate (8c)

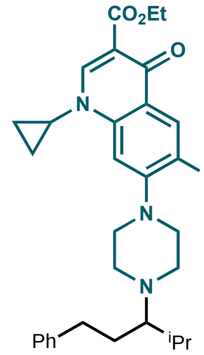

Ethyl-1-cyclopropyl-6-fluoro-7-(4-(4-methyl-1-phenylpentan-3-yl)piperazin-1-yl)-4-oxo-1,4-dihydroquinoline-3-carboxylate was prepared according to General Procedure **B** using Zn (52 mg, 0.8 mmol, 2 equiv), ciprofloxacin ethyl ester (prepared according to *Xu et al.*)[2] (144 mg, 0.4 mmol, 1 equiv), hydrocinnamaldehyde (79 µL, 0.6 mmol, 1.5 equiv), 2-iodopropane (100 µL, 1.2 mmol, 2.5 equiv), and TMSOTf (108 µL, 0.6 mmol, 1.5 equiv) in EtOAc (1 mL). Crude material was purified by column chromatography (0-1% MeOH in dichloromethane (with 5% Et_3N)) yielding the product as a colourless oil (89 mg, 43%).

^{1}H NMR (400 MHz, $CDCl_3$): δ 8.46 (s, 1H), 7.95 (d, J = 13.4 Hz, 1H), 7.30 – 7.15 (m, 6H), 4.36 (q, J = 7.1 Hz, 2H), 3.45 – 3.37 (m, 1H), 3.30 – 3.15 (m, 4H), 2.92 – 2.74 (m, 5H), 2.70 – 2.61 (m, 1H), 2.28 – 2.15 (m, 1H), 1.94 – 1.76 (m, 2H), 1.73 – 1.61 (m, 1H), 1.39 (t, J = 7.1 Hz, 3H), 1.30 (q, J = 6.7 Hz, 2H), 1.17 – 1.09 (m, 2H), 0.96 – 0.92 (m, 6H).

^{13}C NMR (101 MHz, $CDCl_3$): δ 173.0 (d, J = 2.1 Hz), 165.8, 153.3 (d, J = 248.6 Hz), 148.0, 144.8 (d, J = 9.9 Hz), 142.8, 137.9 (d, J = 1.2 Hz) 128.4, 128.3, 125.7, 122.7 (d, J = 7.1 Hz), 113.0 (d, J = 23.1 Hz), 110.2, 104.7, 69.2, 60.8, 50.9, 49.0, 34.8, 34.5, 29.5, 29.4, 21.5, 20.0, 14.4, 8.1.

^{19}F NMR (471 MHz, CDCl3): δ -123.39 (s)

IR (film, cm^{-1}): 2952, 2810, 1705, 1695, 1610, 1495, 1280, 697.

HRMS: m/z calcd for $C_{31}H_{39}FN_3O_3$ $[M+H]^+$ 520.2970; found 520.2965.

Diethyl-4-(6-chlorocyclohexa-1,2,3,5-tetraen-1-yl)-2-methyl-6-((2-((4-methyl-1-phenylpentan-3-yl)amino)ethoxy)methyl)-1,4-dihydropyridine-3,5-dicarboxylate (8d)

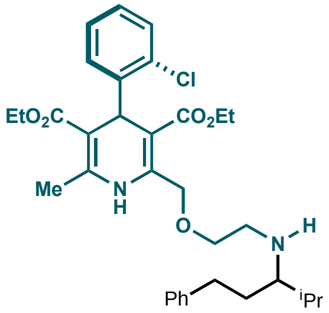

Diethyl-4-(6-chlorocyclohexa-1,2,3,5-tetraen-1-yl)-2-methyl-6-((2-((4-methyl-1-phenylpentan-3-yl)amino)ethoxy)methyl)-1,4-dihydropyridine-3,5-dicarboxylate was prepared according to General Procedure **B** using Zn (26 mg, 0.4 mmol, 2 equiv), amlodipine (82 mg, 0.2 mmol, 1 equiv), hydrocinnamaldehyde (40 µL, 0.3 mmol, 1.5 equiv), 2-iodopropane (50 µL, 0.6 mmol, 2.5 equiv), and TMSOTf (54 µL, 0.3 mmol, 1.5 equiv) in EtOAc (1 mL). Crude material was purified by column chromatography (0-2% MeOH in dichloromethane (with 5% Et_3N)) yielding the product as a colourless oil (38 mg, 33%).

^{1}H NMR (500 MHz, $CDCl_3$): δ 7.44 (s, 1H), 7.39 (dd, J = 7.7, 1.7 Hz, 1H), 7.33 – 7.27 (m, 2H), 7.26 – 7.18 (m, 4H), 7.16 – 7.10 (m, 1H), 7.08 – 7.03 (m, 1H), 5.44 (s, 1H), 4.89 – 4.77 (m, 1H), 4.71 (d, J = 16.1 Hz, 1H), 4.15 – 4.02 (m, 2H), 3.71 – 3.58 (m, 5H), 2.92 – 2.85 (m, 2H), 2.83 – 2.73 (m, 1H), 2.72 – 2.61 (m, 1H), 2.45 – 2.30 (m, 4H), 1.95 – 1.75 (m, 2H), 1.72 – 1.58 (m, 1H), 1.21 (t, J = 7.1 Hz, 3H), 1.02 – 0.90 (m, 6H).

^{13}C NMR (126 MHz, $CDCl_3$): δ 168.1, 167.2, 145.8, 145.6, 144.1, 142.4, 132.3, 131.5, 129.2, 128.4, 128.3, 127.3, 126.9, 125.9, 103.9, 101.4, 67.9, 62.9, 59.8, 50.8, 46.7, 37.2, 32.9, 32.5, 30.1, 19.4, 18.9, 18.3, 14.3.

IR (film, cm^{-1}): 3403, 3060, 2868, 1686, 1477, 1431, 1277, 1204, 1092, 1030, 733, 698.

HRMS: m/z calcd for $C_{33}H_{42}ClN_2O_5$ $[M+H]^+$ 581.2777; found 581.2776.

N,4-dimethyl-1-phenyl-*N*-((*R*)-3-phenyl-3-(4-(trifluoromethyl)phenoxy)propyl)pentan-3-amine (8e)

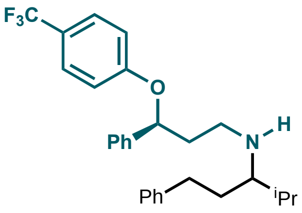

N,4-dimethyl-1-phenyl-*N*-((*R*)-3-phenyl-3-(4-(trifluoromethyl)phenoxy)propyl)pentan-3-amine was prepared according to General Procedure **A** using Zn (52 mg, 0.8 mmol, 2 equiv), fluoxetine hydrochloride (138 mg, 0.4 mmol, 1 equiv), hydrocinnamaldehyde (79 µL, 0.6 mmol, 1.5 equiv), 2-iodopropane (100 µL, 1.2 mmol, 2.5 equiv), and TMSOTf (72 µL, 0.4 mmol, 1 equiv) in EtOAc (1 mL). Crude material was purified by column chromatography (0-2% MeOH in dichloromethane (with 5% Et_3N)) yielding the product as a colourless oil (156 mg, 84%, 1:1.1 d.r.). Data was in line with previous characterisation.[1]

5.2 Aldehyde scope

N-benzyl-N,2-dimethyltridec-12-en-3-amine (9a)

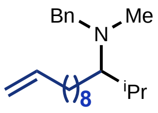

N-benzyl-N,2-dimethyltridec-12-en-3-amine was prepared according to General Procedure **C** using Zn (26 mg, 0.4 mmol, 2 equiv), N-methylbenzylamine hydrochloride (32 mg, 0.2 mmol, 1 equiv), undec-10-enal (80 µL, 0.4 mmol, 2 equiv), 2-iodopropane (68 µL, 0.4 mmol, 2 equiv) and TBSOTf (45 µL, 0.2 mmol, 1 equiv) in EtOAc (1 mL). The product was obtained as a yellow oil (51 mg, 82%).

^{1}H NMR (400 MHz, $CDCl_3$): δ 7.37 - 7.27 (m, 4H), 7.25 - 7.19 (m, 1H), 5.89 - 5.78 (m, 1H), 5.04 - 4.97 (m, 1H), 4.97 - 4.92 (m, 1H), 3.67 (dq, J = 13.8, 1.7 Hz, 1H), 3.63 (dquint, J = 13.8, 1.0 Hz, 1H), 2.23 - 2.15 (m, 4H), 2.09 - 2.02 (m, 2H), 1.88 - 1.78 (m, 1H), 1.46 - 1.24 (m, 14H), 1.00 (d, J = 6.8 Hz, 3H), 0.93 (d, J = 6.7 Hz, 3H).

^{13}C NMR (101 MHz, $CDCl_3$): δ 141.3, 139.4, 128.6, 128.2, 126.6, 114.2, 69.4, 59.3, 37.2, 34.0, 30.6, 30.3, 29.8, 29.7, 29.3, 29.1, 29.0, 27.9, 21.7, 20.6.

IR (film, cm^{-1}): 3075, 3026, 2924, 2852, 2786, 1620, 1494, 1465, 1452, 1362, 1026, 992, 908, 732.

HRMS: m/z calcd for $C_{22}H_{37}N$ $[M+H]^+$ 316.2999; found 316.3007.

Ethyl 6-(benzyl(methyl)amino)-7-methyloctanoate (9b)

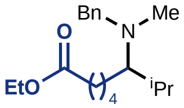

Ethyl 6-(benzyl(methyl)amino)-7-methyloctanoate was prepared according to General Procedure **C** using Zn (26 mg, 0.4 mmol, 2 equiv), N-methylbenzylamine hydrochloride (32 mg, 0.2 mmol, 1 equiv), ethyl 6-oxohexanoate (synthesised using the procedure from Czekelius et al)[3] (48 mg, 0.3 mmol, 1.5 equiv), 2-iodopropane (68 µL, 0.4 mmol, 2 equiv) and TBSOTf (45 µL, 0.2 mmol, 1 equiv) in EtOAc (1 mL) with modifications.

After the reaction, the mixture was diluted with dichloromethane and filtered to remove unreacted zinc. It was then diluted with H_2O. Saturated aq $NaHCO_3$ (0.3 mL) was added. The organic and aqueous layers were separated. The aqueous layer was extracted with dichloromethane twice. 1% NaOH was added to the aqueous layer. The aqueous layer was extracted with dichloromethane twice. The combined organic layer was dried over Na_2SO_4 and filtered. Solvent was removed *in vacuo*. Petroleum ether and H_2O were added. HCl (3M, aq) was added until pH is lower than 4. The organic and aqueous layers were shake vigorously in a separating funnel and separated. Saturated aq $NaHCO_3$ was added to the aqueous layer until pH is higher than 9. Dichloromethane was added. The organic and aqueous layers were shake vigorously in a separating funnel and separated. 1% NaOH was added to the aqueous layer. The aqueous layer was extracted with dichloromethane twice. The combined organic layer was dried over Na_2SO_4 and filtered. The solvent was removed *in vacuo* to afford pure product (43.7 mg, 82%) as a colourless oil.

^{1}H NMR (600 MHz, $CDCl_3$): δ 7.34 - 7.27 (m, 4H), 7.23 - 7.19 (m, 1H), 4.14 (q, J = 7.1 Hz, 2H), 3.65 (d, J = 13.7 Hz, 1H), 3.64 (d, J = 13.7 Hz, 1H), 2.33 (t, J = 7.6 Hz, 2H), 2.21 (td, J = 7.2, 4.4 Hz, 1H), 2.16 (s, 3H), 1.83 (octet, J = 6.7 Hz, 1H), 1.69 - 1.62 (m, 2H), 1.58 - 1.46 (m, 2H), 1.42 - 1.32 (m, 2H), 1.26 (t, J = 7.1 Hz, 3H), 0.98 (d, J = 6.7 Hz, 3H), 0.92 (d, J = 6.7 Hz, 3H).

^{13}C NMR (151 MHz, $CDCl_3$): δ 174.0, 141.1, 128.6, 128.2, 126.7, 69.1, 60.3, 59.3, 37.1, 34.6, 30.5, 28.4, 27.5, 25.6, 21.7, 20.6, 14.4

IR (film, cm^{-1}): 3026, 2934, 2869, 1733, 1453, 1371, 1176, 1027, 733, 698.

HRMS: m/z calcd for $C_{19}H_{31}NO_2$ $[M+H]^+$ 306.2428 ; found 306.2432.

Synthesis of 6-(1,2,3,4-tetrahydro-9H-carbazol-9-yl)hexanal

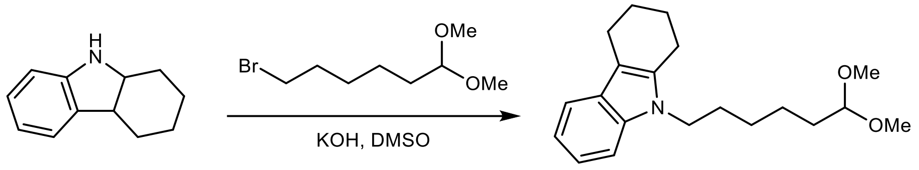

1,2,3,4-tetrahydrocarbazole (617 mg, 3.6 mmol 1 equiv), 6-bromo-1,1-dimethoxyhexane (811 mg, 3.6 mmol, 1 equiv) and KOH (808 mg, 14.4 mmol, 4 equiv) were dissolved in DMSO (9 mL). The mixture was stirred at rt for 48 hours. The reaction was quenched with saturated aqueous NH_4Cl. dichloromethane was added. The aqueous and organic layer were separated. The aqueous layer was extracted with dichloromethane. The combined organic layer was washed with H_2O and brine twice respectively, dried over Na_2SO_4 and filtered. The solvent was removed *in vacuo*. The crude mixture was purified by column chromatography (10-20% Et_2O in Pet. Ether) to afford 9-(6,6-dimethoxyhexyl)-2,3,4,9-tetrahydro-1H-carbazole (1.0 g, 88%) as a colourless oil.

^{1}H NMR (400 MHz, $CDCl_3$): δ 7.47 (d, J = 7.6 Hz, 1H), 7.26 (d, J = 7.8 Hz, 1H), 7.14 (td, J = 7.1, 1.0 Hz, 1H), 7.06 (td, J = 7.5, 0.9, 1H), 4.33 (t, J = 5.7 Hz, 1H), 4.00 (t, J = 7.4 Hz, 2H), 3.31 (s, 6H), 2.77 - 2.68 (m, 4H), 1.99 - 1.82 (m, 4H), 1.75 (quint, J = 7.3 Hz, 2H), 1.63 - 1.53 (m, 2H), 1.44 - 1.34 (m, 4H).

^{13}C NMR (101 MHz, $CDCl_3$): δ 136.2, 135.4, 127.4, 120.6, 118.6, 117.9, 109.4, 108.9, 104.6, 52.9, 43.0, 32.6, 30.5, 27.2, 24.5, 23.5, 23.4, 22.5, 21.3.

IR (film, cm^{-1}): 3062, 2930, 2854, 2833, 1467, 1426, 1371, 1316, 1181, 1130, 1051, 736.

HRMS: m/z calcd for $C_{20}H_{29}NO_2$ $[M+H]^+$ 316.2272, found 316.2269.

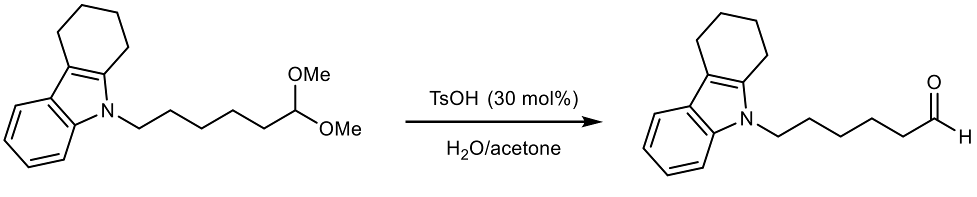

9-(6,6-dimethoxyhexyl)-2,3,4,9-tetrahydro-1H-carbazole (895 mg, 2.84 mmol, 1 equiv) was dissolved in water (9.5 mL) and acetone (47 mL). TsOH monohydrate (162 mg, 0.85 mmol, 30 mol%) was added. The reaction mixture was stirred at 40 °C for 4 h. The mixture was diluted with Et_2O. Saturated aqueous $NaHCO_3$ was added. The organic and aqueous layers were separated. The aqueous layer was extracted twice with Et_2O. The combined organic layer was washed with brine, dried over Na_2SO_4 and filtered. The solvent was removed *in vacuo*. The crude product was purified by column chromatography (10-20% Et_2O in Pet. Ether) to afford 6-(1,2,3,4-tetrahydro-9H-carbazol-9-yl)hexanal (720 mg, 94%) as a colourless oil.

^{1}H NMR (400 MHz, $CDCl_3$): δ 9.77 (t, J = 1.5 Hz, 1H), 7.49 (d, J = 8.1 Hz, 1H), 7.27 (d, J = 8.2 Hz, 1H), 7.16 (td, J = 7.5, 1.2 Hz, 1H), 7.08 (td, J = 7.4, 1.1 Hz, 1H), 4.04 (t, J = 7.3 Hz, 2H), 2.79-2.69 (m, 4H), 2.45 (td, J = 7.2, 1.5 Hz, 2H), 2.01-1.93 (m, 2H), 1.93 - 1.85 (m, 2H), 1.78 (quint, J = 7.6 Hz, 2H), 1.68 (quint, J = 7.6 Hz, 2H), 1.46 - 1.37 (m, 2H).

^{13}C NMR (176 MHz, $CDCl_3$): δ 202.4, 136.1, 135.2, 127.3, 120.5, 118.6, 117.8, 109.4, 108.7, 43.7, 42.6, 30.2, 26.7, 23.4, 23.3, 22.3, 21.8, 21.1.

IR (film, cm^{-1}): 3048, 2930, 2852, 2718, 1722, 1613, 1468, 1444, 1426, 1372, 738.

HRMS: m/z calcd for $C_{20}H_{23}NO$ $[M+H]^+$ 270.1853, found 270.1853.

N-benzyl-N,2-dimethyl-8-(1,2,3,4-tetrahydro-9H-carbazol-9-yl)octan-3-amine (9c)

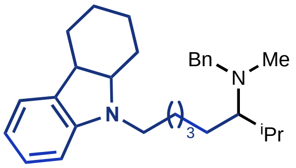

N-benzyl-N,2-dimethyl-8-(1,2,3,4-tetrahydro-9H-carbazol-9-yl)octan-3-amine was prepared according to General Procedure **C** using Zn (26 mg, 0.4 mmol, 2 equiv), N-methylbenzylamine hydrochloride (32 mg, 0.2 mmol, 1 equiv), 6-(1,2,3,4-tetrahydro-9H-carbazol-9-yl)hexanal (108 mg, 0.4 mmol, 2 equiv), 2-iodopropane (68 µL, 0.4 mmol, 2 equiv) and TBSOTf (45 µL, 0.2 mmol, 1 equiv) in EtOAc (1 mL). Crude material was purified by column chromatography (10-12% Et_2O in Pet. Ether) yielding the product as a colourless oil (34 mg, 40%).

^{1}H NMR (500 MHz, $CDCl_3$): δ 7.48 (d, J = 7.7 Hz, 1H), 7.35 - 7.29 (m, 5H), 7.26 - 7.22 (m, 1H), 7.16 - 7.13 (m, 1H), 7.09 - 7.05 (m, 1H), 4.02 (t, J = 7.5 Hz, 2H), 3.65 (d, J = 13.7 Hz, 1H), 3.62 (d, J = 13.7 Hz, 1H), 2.77 - 2.71 (m, 4H), 2.21 - 2.17 (m, 1H), 2.16 (s, 3H), 1.98 - 1.93 (m, 2H), 1.90 - 1.80 (m, 3H), 1.77 (quint, J = 7.4 Hz, 2H), 1.54 - 1.47 (m, 2H), 1.41 - 1.29 (m, 4H), 0.98 (d, J = 6.7 Hz, 3H), 0.91 (d, J = 6.7 Hz, 3H).

^{13}C NMR (100 MHz, $CDCl_3$): δ 141.2, 136.2, 135.4, 128.6, 128.2, 127.4, 126.7, 120.5, 118.6, 117.9, 109.3, 108.9, 69.1, 59.4, 43.1, 37.1, 30.6, 30.4, 28.7, 27.7, 23.5, 23.4, 22.5, 21.8, 21.2, 20.6.

IR (film, cm^{-1}): 3026, 2928, 2853, 2785, 1613, 1584, 1493, 1467, 1426, 1371, 1316, 1233, 1178, 1145, 1014, 734, 697.

HRMS: m/z calcd for $C_{29}H_{40}N_2$ $[M+H]^+$ 417.3265; ; found 417.3268.

Benzyl 4-(2-(benzyl(methyl)amino)-3-methylbutyl)piperidine-1-carboxylate (9d)

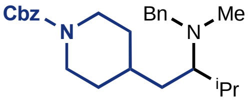

Benzyl 4-(2-(benzyl(methyl)amino)-3-methylbutyl)piperidine-1-carboxylate was prepared according to General Procedure **C** using Zn (26 mg, 0.4 mmol, 2 equiv), N-methylbenzylamine hydrochloride (32 mg, 0.2 mmol, 1 equiv), benzyl 4-(2-oxoethyl)piperidine-1-carboxylate (105 mg, 0.4 mmol, 2 equiv), 2-iodopropane (68 µL, 0.4 mmol, 2 equiv) and TBSOTf (45 µL, 0.2 mmol, 1 equiv) in EtOAc (1 mL). Crude material was purified by column chromatography (15-30% Et_2O in Pet. Ether) yielding the product as a colourless oil (47 mg, 58%).

^{1}H NMR (500 MHz, $CDCl_3$): δ 7.40 - 7.35 (m, 4H), 7.34 - 7.28 (m, 5H), 7.25 - 7.21 (m, 1H), 5.15 (s, 2H), 4.32-4.06 (m, 2H), 3.69 (d, J = 13.6 Hz, 1H), 3.64 (d, J = 13.6, 1H), 2.87 - 2.65 (m, 2H), 2.39-2.33 (m, 1H), 2.20 (s, 3H), 1.90 (octet, J = 6.7 Hz, 1H), 1.79 - 1.57 (m, 3H), 1.52 - 1.46 (m, 1H), 1.19 - 1.11 (m, 2H), 1.07 - 0.97 (m, 4H), 0.92 (d, J = 6.7 Hz, 3H).

^{13}C NMR (101 MHz, $CDCl_3$): δ 155.4, 140.9, 137.2, 128.6, 128.3, 128.0, 127.9, 126.8, 67.0, 65.0, 59.4, 44.6, 44.4, 36.9, 34.8, 33.7, 33.2, 31.9, 29.8, 22.1, 20.4.

IR (film, cm^{-1}): 3062, 3029, 2926, 2869, 2845, 2786, 1696, 1494, 1467, 1276, 1240, 1215, 1073, 1018, 732, 695.

HRMS: m/z calcd for $C_{26}H_{36}N_2O_2$ $[M+H]^+$ 409.2850, found 409.2860.

N-benzyl-N,2,5-trimethylhexan-3-amine (9e)

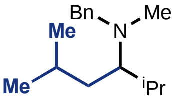

N-benzyl-N,2,5-trimethylhexan-3-amine was prepared according to General Procedure **C** using Zn (26 mg, 0.4 mmol, 2 equiv), N-methylbenzylamine hydrochloride (32 mg, 0.2 mmol, 1 equiv), 3-methylbutanal (43 µL, 0.4 mmol, 2 equiv), 2-iodopropane (68 µL, 0.4 mmol, 2 equiv) and TBSOTf (45 µL, 0.2 mmol, 1 equiv) in EtOAc (1 mL). The pure product was obtained as a yellow oil (44 mg, 94%).

^{1}H NMR (400 MHz, $CDCl_3$): δ 7.39 - 7.21 (m, 4H), 7.28 - 7.22 (m, 1H), 3.70 (s, 2H), 2.39 - 2.34 (m, 1H), 2.20 (s, 3H), 1.92 - 1.77 (m, 2H), 1.54 - 1.47 (m, 1H), 1.18 - 1.11 (m, 1H), 1.04 (d, J = 6.8 Hz, 3H), 0.98 (d, J = 3.0 Hz, 3H), 0.96 (d, J = 3.0 Hz, 3H), 0.93 (d, J = 6.5 Hz, 3H).

^{13}C NMR (101 MHz, $CDCl_3$): δ 141.4, 128.6, 128.2, 126.6, 66.8, 59.2, 37.4, 37.1, 30.6, 26.1, 23.7, 22.6, 21.9, 20.6.

IR (film, cm^{-1}): 3063, 3026, 2952, 2928, 2867, 1494, 1466, 1462, 1383, 1366, 1027, 732.

HRMS: m/z calcd for $C_{16}H_{27}N$ $[M+H]^+$ 234.2217; found 234.2217.

N-benzyl-N,2,5,5-tetramethylhexan-3-amine (9f)

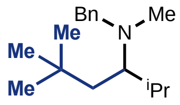

N-benzyl-N,2,5,5-tetramethylhexan-3-amine was prepared according to General Procedure **C** using Zn (52 mg, 0.8 mmol, 4 equiv), N-methylbenzylamine hydrochloride (32 mg, 0.2 mmol, 1 equiv), 3,3-dimethylbutanal (50 µL, 0.4 mmol, 2 equiv), 2-iodopropane (136 µL, 0.8 mmol, 4 equiv) and TBSOTf (45 µL, 0.2 mmol, 1 equiv) in EtOAc (1 mL). The pure product was obtained as a yellow oil (47 mg, 95%)

^{1}H NMR (400 MHz, $CDCl_3$): δ 7.37 - 7.27 (m, 4H), 7.25 - 7.20 (m, 1H), 3.77 (d, J = 13.6 Hz, 1H), 3.62 (d, J = 13.6 Hz, 1H), 2.53 - 2.46 (m, 1H), 2.18 (s, 3H), 2.04 - 1.93 (m, 1H), 1.47 (dd, J = 14.4 Hz, 7.8 Hz, 1H), 1.16 (dd, J = 14.5 Hz, 2.8 Hz, 1H), 0.98 (s, 9H), 0.95 (d, J = 6.8 Hz, 6H).

^{13}C NMR (101 MHz, $CDCl_3$): δ 141.2, 128.7, 128.2, 126.6, 65.9, 59.3, 40.6, 37.6, 30.6, 29.1, 22.2, 20.3.

IR (film, cm^{-1}): 3063, 3027, 2951, 2867, 2786, 1494, 1465, 1463, 1388, 1363, 1244, 1216, 1045, 1020, 909, 723, 697.

HRMS: m/z calcd for $C_{17}H_{29}N$ $[M+H]^+$ 248.2373; found 248.2375.

N-benzyl-N,2,4-trimethylpentan-3-amine (9g)

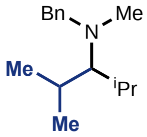

N-benzyl-N,2,4-trimethylpentan-3-amine was prepared according to General Procedure **C** using Zn (52 mg, 0.8 mmol, 4 equiv), N-methylbenzylamine hydrochloride (32 mg, 0.2 mmol, 1 equiv), isobutyraldehyde (37 µL, 0.4 mmol, 2 equiv), 2-iodopropane (136 µL, 0.8 mmol, 4 equiv) and TBSOTf (45 µL, 0.2 mmol, 1 equiv) in EtOAc (1 mL). The pure product was obtained as a yellow oil (44 mg, 95%). Data was in line with previous characterisation.[1]

N-benzyl-1-(cyclohex-3-en-1-yl)-N,2-dimethylpropan-1-amine (9h)

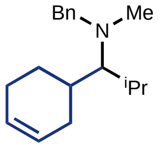

N-benzyl-1-(cyclohex-3-en-1-yl)-N,2-dimethylpropan-1-amine was prepared according to General Procedure **C** using Zn (52 mg, 0.8 mmol, 4 equiv), N-methylbenzylamine hydrochloride (32 mg, 0.2 mmol, 1 equiv), cyclohex-3-ene-1-carbaldehyde (47 µL, 0.4 mmol, 2 equiv), 2-iodopropane (136 µL, 0.8 mmol, 4 equiv) and TMSCl (25 µL, 0.2 mmol, 1 equiv) in EtOAc (1 mL). The pure product was obtained as a colourless oil (43 mg, 1.7:1 d.r., 82%)

Note: TMSCl was used instead of TBSOTf due to electron rich π system.

^{1}H NMR (700 MHz, $CDCl_3$): δ 7.27 - 7.33 (m, 2H), 7.32 - 7.28 (m, 2H), 7.23 - 7.20 (m, 1H); 5.74 - 5.65 (m, 2H), 3.81 (s, 1.28H), 3.74 (s, 0.72H), 2.28 (s, 1.93H), 2.23 - 2.20 (m, 2.08H), 2.19 - 2.14 (m, 0.36H), 2.12 - 1.90 (m, 6.28H), 1.81 - 1.7 (m, 0.36H), 1.47 - 1.33 (m, 1H), 1.03 - 0.99 (m, 6H).

^{13}C NMR (175 MHz, $CDCl_3$): δ 141.4, 141.3, 128.6, 128.5, 128.23, 128.21, 127.5, 127.4, 127.1, 127.0, 126.63, 126.60, 73.4, 72.8, 61.0, 60.6, 39.2, 38.3, 35.4, 34.6, 30.9, 30.8, 28.6, 28.0, 27.4, 27.0, 26.1, 25.8, 21.9, 21.6, 21.3, 21.1.

IR (film, cm^{-1}): 3084, 3062, 3021, 2955, 2908, 2872, 2835, 2785, 1653, 1603, 1494, 1452, 1435, 1386, 1362, 1023, 734, 698, 656.

HRMS: m/z calcd for $C_{18}H_{27}N$ $[M+H]^+$ 258.2217; found 258.2223.

N-benzyl-N,2-dimethyl-1-(tetrahydro-2H-pyran-4-yl)propan-1-amine (9i)

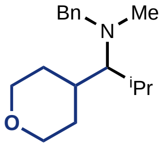

N-benzyl-N,2-dimethyl-1-(tetrahydro-2H-pyran-4-yl)propan-1-amine was prepared according to General Procedure **C** using Zn (26 mg, 0.4 mmol, 2 equiv), N-methylbenzylamine hydrochloride (32 mg, 0.2 mmol, 1 equiv), tetrahydro-2*H*-pyran-4-carbaldehyde (41 µL, 0.4 mmol, 2 equiv), 2-iodopropane (68 µL, 0.4 mmol, 2 equiv) and TBSOTf (45 µL, 0.2 mmol, 1 equiv) in EtOAc (1 mL). The pure product was obtained as a yellow oil (44 mg, 85%). Data was in line with previous characterisation.[1]

N-benzyl-N,3-dimethyl-1-phenylbutan-2-amine (9j)

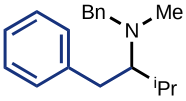

N-benzyl-N,3-dimethyl-1-phenylbutan-2-amine was prepared according to General Procedure **C** using Zn (52 mg, 0.8 mmol, 4 equiv), N-methylbenzylamine hydrochloride (32 mg, 0.2 mmol, 1 equiv), 2-phenylacetaldehyde (46 µL, 0.4 mmol, 2 equiv), 2-iodopropane (136 µL, 0.8 mmol, 4 equiv) and TBSOTf (45 µL, 0.2 mmol, 1 equiv) in EtOAc (1 mL). The pure product was obtained as a colourless oil (51 mg, 95%).

^{1}H NMR (400 MHz, $CDCl_3$): δ 7.34 - 7.14 (m, 10H), 3.61 (s, 2H), 2.92 (dd, J = 14.0, 7.0 Hz, 1H), 2.78 - 2.65 (m, 2H), 2.25 (s, 3H), 1.91 (octet, J = 6.7 Hz, 1H), 1.08 (d, J = 6.6 Hz, 3H), 0.97 (d, J = 6.6 Hz, 3H).

^{13}C NMR (101 MHz, $CDCl_3$): δ 142.6, 141.0, 129.4, 128.6, 128.3, 128.1, 126.6, 125.7, 71.2, 60.0, 37.0, 34.3, 31.4, 21.4, 21.2.

IR (film, cm^{-1}): 3083, 3061, 3025, 2954, 2871, 2843, 2786, 1602, 1494, 1467, 1453, 1363, 1026, 733, 697.

HRMS: m/z calcd for $C_{19}H_{25}N$ $[M+H]^+$ 268.2060; found 268.2072.

N-benzyl-1-(3,4-dimethoxyphenyl)-N,3-dimethylbutan-2-amine (9k)

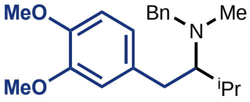

N-benzyl-1-(3,4-dimethoxyphenyl)-N,3-dimethylbutan-2-amine was prepared according to General Procedure **C** using Zn (39 mg, 0.6 mmol, 3 equiv), N-methylbenzylamine hydrochloride (32 mg, 0.2 mmol, 1 equiv), 2-(3,4-dimethoxyphenyl)acetaldehyde (synthesised using the procedure from *Couture et al*)[4] (63 µL, 0.4 mmol, 2 equiv), 2-iodopropane (102 µL, 0.6 mmol, 3 equiv) and TESCl (34 µL, 0.2 mmol, 1 equiv) in EtOAc (1 mL). Crude material was purified by column chromatography (20-50% Et_2O in Pet. Ether) yielding the product as a colourless oil (43 mg, 66%).

Note: It should be noted that TESCl, instead of TBSOTf, was used.

^{1}H NMR (400 MHz, $CDCl_3$): δ 7.26 - 7.16 (m, 5H), 6.82 - 6.70 (m, 3H), 3.88 (s, 3H), 3.83 (s, 3H), 3.59 (s, 2H), 2.87 - 2.79 (m, 1H), 2.69-2.58 (m, 2H), 2.24 (s, 3H), 1.94 - 1.82 (m, 1H), 1.04 (d, J = 6.7 Hz, 3H), 0.94 (d, J = 6.7 Hz, 3H).

^{13}C NMR (101 MHz, $CDCl_3$): δ 148.8, 147.2, 140.9, 135.1, 128.6, 128.1, 126.6, 121.2, 112.7, 111.2, 70.7, 59.9, 56.1, 60.0, 37.0, 33.8, 31.3, 21.4, 21.1.

IR (film, cm^{-1}): 3060, 3025, 2951, 2932, 2870, 2833, 2785, 1589, 1514, 1493, 1463, 1451, 1416, 1260, 1237, 1191, 1154, 1140, 1027, 807, 784, 766, 735, 699.

HRMS: m/z calcd for $C_{21}H_{29}NO_2$ $[M+H]^+$ 328.2272; found 328.2274.

N-Benzyl-N-methyl-1-phenylpropan-3-amine (9l)

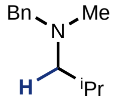

N-Benzyl-N-methyl-1-phenylpropan-3-amine was prepared according to General Procedure **C** with modifications. An oven dried vial (Biotage Microwave Process Vial 10 mL, equipped with gas-tight pierceable caps) was charged with a stir bar, Zn dust (52 mg, 0.8 mmol, 2 equiv) and paraformaldehyde (36 mg, 1.2 mmol, 3 equiv). The vial was sealed, evacuated, and backfilled with N_2. *n*BuOAc (1 mL) and N-methylbenzylamine (52 µL, 0.4 mmol, 1.0 equiv) were added. The mixture was stirred for 10 min. To this was added 2-iodopropane (100 µL, 1.0 mmol, 2.5 equiv). The reaction mixture was stirred for one minute, followed by the addition of TMSOTf (108 µL, 0.3 mmol, 1.5 equiv). The reaction mixture was stirred for 18 h. The crude mixture was worked up according to general procedure **C**. Crude material was purified by column chromatography (5-10% EtOAc in Pet. Ether) yielding the product as a colourless oil (14 mg, 39%).

^{1}H NMR (500MHz, $CDCl_3$) δ 7.35 - 7.29 (m, 4H), 7.26 – 7.21 (m, 1H), 3.46 (s, 2H), 2.16 (s, 3H), 2.12 (d, *J*= 7.4 Hz, 2H), 1.88- 1.76 (m, 1H), 0.91 (d, *J* = 6.6 Hz, 6H).

^{13}C NMR (126 MHz, $CDCl_3$) δ 139.7, 128.9, 128.1, 126.7, 66.1, 62.7, 42.7, 26.2, 20.8.

IR (film, cm^{-1}):2952, 2783, 1452, 1035, 1026, 735, 697.

HRMS: m/z calcd for $C_{12}H_{19}N$ $[M+H]^+$ 178.1590; found 178.1590.

N-benzyl-N,2-dimethyl-6-(5-methylfuran-2-yl)hexan-3-amine (9m)

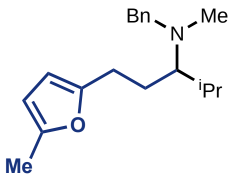

The synthesis of N-benzyl-N,2-dimethyl-6-(5-methylfuran-2-yl)hexan-3-amine was attempted according to General Procedure **C** using Zn (39 mg, 0.6 mmol, 3 equiv), N-methylbenzylamine hydrochloride (32 mg, 0.2 mmol, 1 equiv), 3-(5-Methyl-2-furyl)propionaldehyde (55 mg, 0.4 mmol, 2 equiv), 2-iodopropane (136 µL, 0.8 mmol, 4 equiv) and TMSOTf (36 µL, 0.2 mmol, 0.5 equiv) in EtOAc (1 mL). Yield of the product was determined by ^{1}H NMR using 1,1,2,2-tetrachloroethane as internal standard indicating a 53% yield based on isopropyl peak at 1.05 ppm.

Note: Use of other lewis acids or lewis acid loadings did not improve yields.

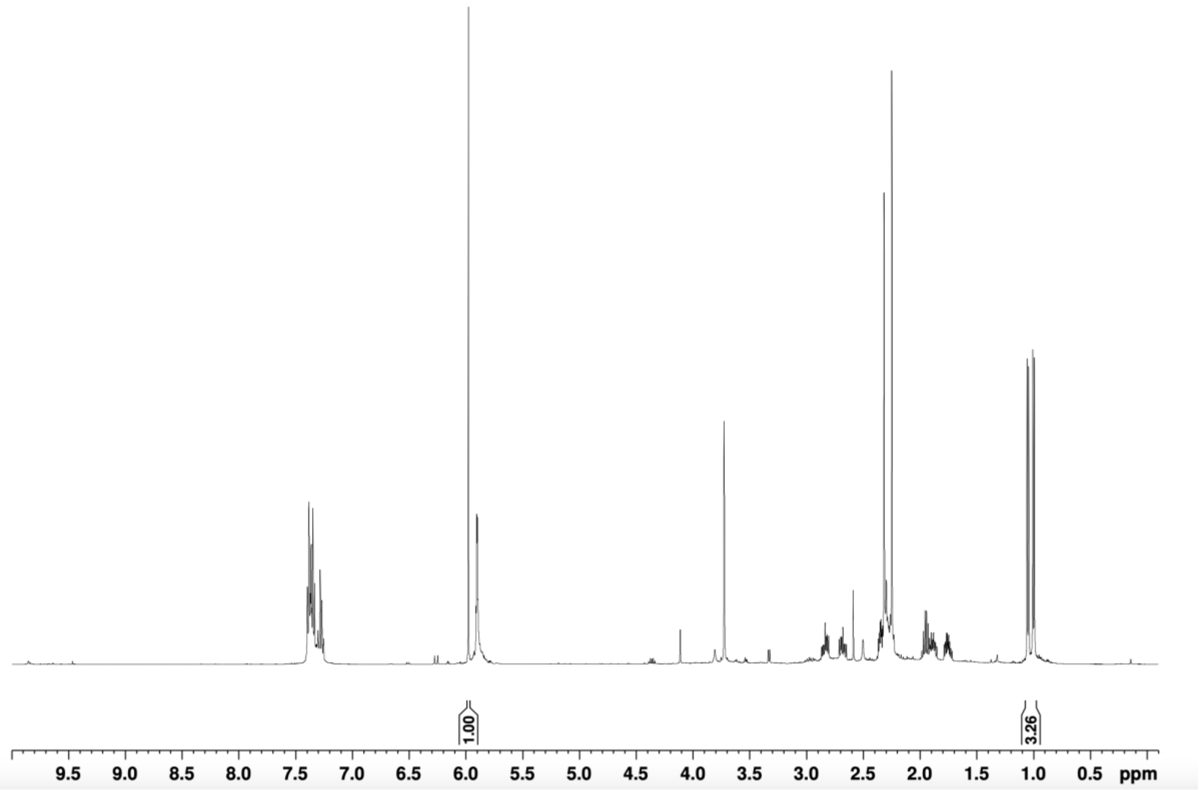

5.3 Alkyl halide scope

N-benzyl-1-cyclohexyl-N-methyl-3-phenylpropan-1-amine (10a)

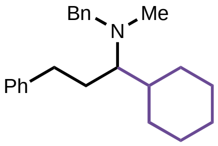

N-benzyl-1-cyclohexyl-N-methyl-3-phenylpropan-1-amine was prepared according to General Procedure **B** using Zn (52 mg, 0.8 mmol, 2 equiv), N-methylbenzylamine (52 µL, 0.4 mmol, 1 equiv), hydrocinnamaldehyde (78 µL, 0.6 mmol, 1.5 equiv), cyclohexyl iodide (129 µL, 1 mmol, 2.5 equiv) and TMSOTf (144 µL, 0.8 mmol, 2 equiv) in EtOAc (1 mL). Crude material was purified by acid-base wash according to general procedure **ABW,** yielding the product as a colourless oil (91 mg, 71%).

^{1}H NMR (400 MHz, $CDCl_3$): δ 7.46 - 7.35 (m, 6H), 7.33-7.25 (m, 4H), 3.76 (s, 2H), 2.94 - 2.85 (m, 1H), 2.78 - 2.69 (m, 1H), 2.47 - 2.70 (m, 1H), 2.30 (s, 3H), 2.06 - 1.71 (m, 7H), 1.69 - 1.59 (m, 1H), 1.42 - 1.05 (m, 5H).

^{13}C NMR (101 MHz, $CDCl_3$): δ 143.4, 141.1, 128.7, 125.53, 128.47, 128.3, 126.7, 125.8, 68.0, 59.3, 40.4, 37.2, 35.3, 31.9, 31.1, 30.2, 26.90, 26.87, 26.8.

IR (film, cm^{-1}): 3061, 2920, 2849, 2785, 1494, 1450, 1027, 1027, 731, 696.

HRMS: m/z calcd for $C_{23}H_{31}N$ $[M+H]^+$ 322.2530; found 322.2545.

N-benzyl-N-methyl-3-phenyl-1-(tetrahydro-2H-pyran-4-yl)propan-1-amine (10b)

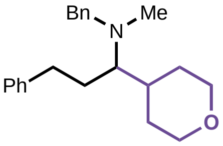

N-benzyl-N-methyl-3-phenyl-1-(tetrahydro-2H-pyran-4-yl)propan-1-amine was prepared according to General Procedure **B** using Zn (52 mg, 0. 8 mmol, 2 equiv), N-methylbenzylamine (52 µL, 0.4 mmol, 1 equiv), hydrocinnamaldehyde (78 µL, 0.6 mmol, 1.5 equiv), 4-iodotetrahydro-2H-pyran (212 mg, 1 mmol, 2.5 equiv) and TMSOTf (144 µL, 0.8 mmol, 2 equiv) in EtOAc (1 mL). Crude material was purified by column chromatography (0-30% Et_2O in Pet. Ether), yielding the product as a colourless oil (79 mg, 61%).

^{1}H NMR (400 MHz, $CDCl_3$): δ 7.37 - 7.30 (m, 6H), 7.29 - 7.20 (m, 4H), 4.07 - 4.00 (m, 2H), 3.74 (d, J = 13.6 Hz, 1H), 3.70 (d, J = 13.7 Hz, 1H), 3.45 - 3.36 (m, 2H), 2.88 - 2.78 (m, 1H), 2.74 - 2.63 (m, 1H), 2.44 - 2.36 (m, 1H), 2.25 (s, 3H), 1.96 - 1.84 (m, 2H), 1.84 - 1.60 (m, 3H), 1.50 - 1.35 (m, 2H).

^{13}C NMR (101 MHz, $CDCl_3$): δ 142.9, 140.7, 128.6, 128.54, 128.47, 128.3, 126.9, 125.9, 68.6, 68.2, 67.4, 59.5, 38.4, 36.8, 35.2, 31.9, 31.4, 30.0.

IR (film, cm^{-1}): 3024, 2946, 2839, 1494, 1453, 1092, 1027, 734, 697.

HRMS: m/z calcd for $C_{22}H_{29}NO$ $[M+H]^+$ 324.2322; found 324.2325.

Benzyl 4-(1-(benzyl(methyl)amino)-3-phenylpropyl)piperidine-1-carboxylate (10c)

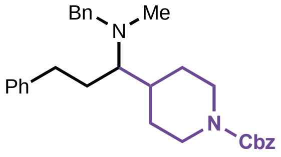

Benzyl 4-(1-(benzyl(methyl)amino)-3-phenylpropyl)piperidine-1-carboxylate (was prepared according to General Procedure **B** using Zn (52 mg, 0.8 mmol, 2 equiv), N-methylbenzylamine (52 µL, 0.4 mmol, 1 equiv), hydrocinnamaldehyde (80 µL, 0.6 mmol, 1.5 equiv), benzyl 4-iodopiperidine-1-carboxylate (345 mg, 1 mmol, 2.5 equiv) and TMSOTf (144 µL, 0.8 mmol, 2 equiv) in EtOAc (1 mL). Crude material was subjected to an acid-base wash according to general procedure **ABW**, followed by column chromatography (30-70% Et_2O in Pet. Ether) yielding the product as a colourless oil (119 mg, 65%). Data was in line with previous characterisation.[1]

N-benzyl-1-cyclopentyl-N-methyl-3-phenylpropan-1-amine (10d)

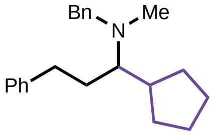

N-benzyl-1-cyclopentyl-N-methyl-3-phenylpropan-1-amine was prepared according to General Procedure **B** using Zn (52 mg, 0. 8 mmol, 2 equiv), N-methylbenzylamine (52 µL, 0.4 mmol, 1 equiv), hydrocinnamaldehyde (93 µL, 0.7 mmol, 1.75 equiv), iodocyclopentane (139 µL, 1.2 mmol, 3 equiv) and TMSOTf (127 µL, 0.7 mmol, 1.75 equiv) in EtOAc (1 mL). Crude material was purified by column chromatography (0-5% EtOAc in Pet. Ether), yielding the product as a colourless oil (73 mg, 59% yield).

^{1}H NMR (500 MHz, $CDCl_3$): δ 7.39 – 7.35 (m, 2H), 7.34 – 7.27 (m, 4H), 7.25 – 7.16 (m, 4H), 3.74 (d, J = 13.7 Hz, 1H), 3.69 (d, J = 13.7 Hz, 1H), 2.90 (ddd, J = 13.6, 10.5, 5.1 Hz, 1H), 2.67 (ddd, J = 13.7, 10.4, 6.4 Hz, 1H), 2.43 (td, J = 8.6, 3.7 Hz, 1H), 2.23 (s, 3H), 2.05 (ddt, J = 17.5, 9.8, 7.6 Hz, 1H), 1.93 – 1.80 (m, 2H), 1.79 –1.57 (m, 4H), 1.56-1.46 (m, 2H), 1.38 – 1.24 (m, 1H), 1.17 (ddt, J = 12.1, 9.7, 8.1 Hz, 1H).

^{13}C NMR (126 MHz, $CDCl_3$): δ 143.4, 141.2, 128.5, 128.5, 128.3, 128.1, 126.6, 125.6, 67.9, 59.1, 43.2, 36.8, 34.5, 32.5, 32.1, 30.8, 25.3, 25.2.

IR (film, cm^{-1}): 2945, 2863, 2784, 1602, 1493, 1451, 1353, 1027, 731, 696, 619.

HRMS: m/z calculated for $C_{22}H_{30}N$ $[M+H]^+$ 308.2378; found 308.2373.

N-benzyl-N-methyl-4-methyl-1-phenylhexan-3-amine (10e)

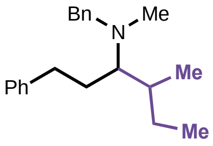

N-benzyl-N-methyl-4-methyl-1-phenylhexan-3-amine was prepared according to General Procedure **A** using Zn (52 mg, 0. 8 mmol, 2 equiv), N-methylbenzylamine hydrochloride (63 mg, 0.4 mmol, 1 equiv), hydrocinnamaldehyde (78 µL, 0.6 mmol,1.5 equiv), 2-iodobutane (139 µL, 1.2 mmol, 3 equiv) and TMSOTf (36 µL, 0.2 mmol, 0.5 equiv) in EtOAc (1 mL). Crude material was purified by column chromatography (0-2% EtOAc in Pet. Ether), yielding the product as a colourless oil (59 mg, 1:1 d.r., 58%).

Diastereomers not distinguishable by ^{1}H NMR.

^{1}H NMR (500 MHz, $CDCl_3$): δ 7.41-7.30 (m, 6H), 7.29-7.21 (m, 4H), 3.77-3.64 (m, 2H), 2.94 - 2.79 (m, 1H), 2.73 - 2.65 (m, 1H), 2.51 -2.44 (m, 1H), 2.26-2.23 (m, 3H), 1.96-1.85 (m, 1H), 1 .78 - 1.51(m, 3H), 1.28-1.13 (m, 1H), 1.03 - 0.87 (m, 6H).

^{13}C NMR (126 MHz, $CDCl_3$): δ 143.3, 143.2, 141.0, 140.9, 128.6, 128.5, 128.44 ,128.40, 128.4, 128.3, 128.2, 128.1, 126.62, 126.59, 125.69, 125.65, 67.1, 66.5, 59.5, 58.9, 37.4, 37.23, 37.19, 35.42, 35.1, 34.9, 30.0, 29.8, 27.9, 26.6, 17.3, 16.7, 11.9, 11.7.

IR (film, cm^{-1}): 2958, 2930, 1494, 1452, 1027, 732, 697.

HRMS: m/z calcd for $C_{21}H_{30}N$ $[M+H]^+$ 296.2373; found 296.2381.

N-Benzyl-1-(4-iodophenyl)-N-methyl-4-phenylbutan-2-amine (10f)

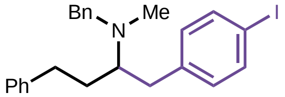

An oven dried vial (Biotage Microwave Process Vial (10 mL), equipped with gas-tight pierceable caps) was charged with a stirring bar was sealed, evacuated and backfilled with N_2. To this was added 1.0 ml dry EtOAc, followed by N-methylbenzylamine (52 µL, 0.4 mmol, 1 equiv) and hydrocinnamaldehyde (78 µL, 0.6 mmol, 1.5 equiv). TMSOTf (72 µL, 0.4 mmol, 1 equiv) was added dropwise. The mixture was stirred for 15 mins. The cap was removed. Zinc (20 mg, 0.3 mmol, 3 equiv) and 4-iodobenzyl bromide (297 mg, 1 mmol, 2.5 equiv) were added in one portion. The microwave vial was sealed, put under vacuum and refilled with N_2. The mixture was stirred overnight. The crude mixture was worked up according to general procedure **A,** then subjected to an acid-base wash according to general procedure **ABW**, followed by column chromatography (10-20% Et_2O in Pet. Ether), yielding the product as a colourless oil (129 mg, 71%).

^{1}H NMR (400 MHz, $CDCl_3$): δ 7.62 (d, J = 7.9 Hz, 2H), 7.39 – 7.27 (m, 7H), 7.22 (t, J = 7.3 Hz, 1H), 7.17 (d, J = 7.5 Hz, 2H), 6.91 (d, J = 7.8 Hz, 2H), 3.77 (d, J = 13.4 Hz, 1H), 3.64 (d, J = 13.5 Hz, 1H), 2.99 (dd, J = 13.3, 5.3 Hz, 1H), 2.94 – 2.79 (m, 2H), 2.59 (ddd, J = 13.9, 10.1, 6.4 Hz, 1H), 2.48 (dd, J = 13.3, 8.5 Hz, 1H), 2.33 (s, 3H), 1.94 – 1.83 (m, 1H), 1.73 – 1.62 (m, 1H).

^{13}C NMR (101 MHz, $CDCl_3$): δ 142.6, 140.6, 140.1, 137.3, 131.4, 128.6, 128.4, 128.34, 128.26, 126.9, 125.7, 90.8, 63.9, 58.2, 36.3, 34.8, 33.3, 32.4.

IR (film, cm^{-1}): 3023, 2931, 2786, 1483, 1452, 1005, 731, 695.

HRMS: m/z calcd for $C_{24}H_{27}IN$ $[M+H]^+$ 456.1183; found 456.1181.

Methyl 3-(2-(benzyl(methyl)amino)-4-phenylbutyl)benzoate (10g)

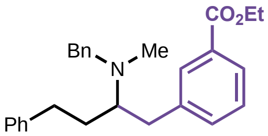

An oven dried vial (Biotage Microwave Process Vial (10 mL), equipped with gas-tight pierceable caps) was charged with a stirring bar was sealed, evacuated and backfilled with N_2. To this was added 1.0 ml dry EtOAc, followed by N-methylbenzylamine (52 µL, 0.4 mmol, 1 equiv) and hydrocinnamaldehyde (78 µL, 0.6 mmol, 1.5 equiv). Then, TMSOTf (72 µL, 0.4 mmol, 1 equiv) was added dropwise. The mixture was stirred for 15 mins. The cap was removed. Zinc (20 mg, 0.3 mmol, 3 equiv) and methyl 3-(bromomethyl)benzoate (229 mg, 1 mmol, 2.5 equiv) were added in one portion. The microwave vial was sealed, put under vacuum and refilled with N_2. The mixture was stirred overnight. The crude mixture was worked up according to general procedure **A,** followed by column chromatography (6-20% Et_2O in Pet. Ether) but the product was still contaminated with impurities. The crude mixture was subjected to an acid-base wash according to general procedure **ABW**, yielding the product as a colourless oil (97 mg, 63%).

^{1}H NMR (400 MHz, $CDCl_3$): δ 7.91 (dd, J = 7.3, 1.7 Hz, 1H), 7.87 – 7.82 (m, 1H), 7.39 – 7.09 (m, 12H), 3.94 (s, 3H), 3.76 (d, J = 13.5 Hz, 1H), 3.64 (d, J = 13.5 Hz, 1H), 3.07 (dd, J = 13.6, 5.3 Hz, 1H), 2.87 (ddd, J = 15.4, 10.2, 5.0 Hz, 2H), 2.64 – 2.49 (m, 2H), 2.32 (s, 3H), 1.95 – 1.79 (m, 1H), 1.64 (ddt, J = 15.1, 11.0, 5.7 Hz, 1H).

^{13}C NMR (101 MHz, $CDCl_3$): δ 167.3, 142.6, 141.3, 140.1, 133.9, 130.3, 130.1, 128.6, 128.4, 128.3, 128.2, 127.1, 126.8, 125.7, 64.1, 58.2, 52.1, 36.3, 35.1, 33.2, 32.3.

IR (film, cm^{-1}): 3025, 2945, 2865, 2788, 1719, 1452, 1279, 1201, 1106, 747, 697.

HRMS: m/z calcd for $C_{26}H_{30}NO_2$ $[M+H]^+$ 388.2272; found 388.2266.

N-benzyl-N-methyl-1,4-diphenylbutan-2-amine (10h)

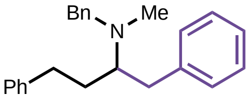

N-benzyl-N-methyl-1,4-diphenylbutan-2-amine was prepared according to General Procedure **A** using Zn (52 mg, 0. 8 mmol, 2 equiv), N-methylbenzylamine hydrochloride (63 mg, 0.4 mmol, 1 equiv), hydrocinnamaldehyde (80 µL, 0.6 mmol,1.5 equiv), benzyl chloride (138 µL, 1.2 mmol, 3 equiv) and TMSOTf (36 µL, 0.2 mmol, 0.5 equiv) in EtOAc (1 mL). The crude product was purified by column chromatography (0-40% Et_2O in Pet. Ether (with 3% NEt3)), yielding the product as a colourless oil (81 mg, 62%).

^{1}H NMR (400 MHz, $CDCl_3$): δ 7.34 – 7.12 (m, 15H), 3.77 (d, J = 13.6 Hz, 1H), 3.63 (d, J = 13.5 Hz, 1H), 3.06 (dd, J = 8.4, 5.0 Hz, 1H), 2.92 – 2.84 (m, 2H), 2.55 – 2.47 (m, 2H), 2.31 (s, 3H) 1.88 – 1.81 (m, 1H), 1.72 – 1.64 (m, 1H).

^{13}C NMR (101 MHz, $CDCl_3$): δ 142.9, 140.9, 140.3, 129.3, 128.6, 128.4, 128.2 (d, J = 5.4 Hz), 126.8, 125.7, 125.6, 64.43 58.1, 36.4, 35.1, 33.2, 32.6.

IR (film, cm^{-1}): 3028, 2917, 2851, 2780, 1594, 1478, 1437, 1354, 1073, 912, 730, 693, 482.

HRMS: m/z calcd for $C_{24}H_{27}N$ $[M+H]^+$ 330.2216; found 330.2217.

N-benzyl-1-(4-chlorophenyl)-N-methyl-4-phenylbutan-2-amine (10i)

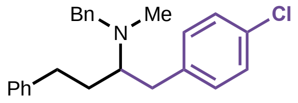

N-benzyl-1-(4-chlorophenyl)-N-methyl-4-phenylbutan-2-amine was prepared according to General Procedure **A** using Zn (52 mg, 0. 8 mmol, 2 equiv), N-methylbenzylamine hydrochloride (63 mg, 0.4 mmol, 1 equiv), hydrocinnamaldehyde (80 µL, 0.6 mmol,1.5 equiv), 4-chlorobenzyl chloride (138 µL, 1.2 mmol, 3 equiv) and TMSOTf (36 µL, 0.2 mmol, 0.5 equiv) in EtOAc (1 mL). The crude product was filtered through an SCX-column (eluting with 5N NH_3 in MeOH) then purified by column chromatography (0-15% EtOAc in Pet. Ether), yielding the product as a colourless oil (102 mg, 70% yield).

^{1}H NMR (500 MHz, $CDCl_3$) δ 7.35 – 7.22 (m, 9H), 7.22 – 7.17 (m, 1H), 7.14 – 7.11 (m, 2H), 7.08 – 7.03 (m, 2H), 3.74 (d, J = 13.5 Hz, 1H), 3.61 (d, J = 13.4 Hz, 1H), 2.98 (dd, J = 13.3, 5.3 Hz, 1H), 2.91 – 2.76 (m, 2H), 2.55 (ddd, J = 13.8, 10.2, 6.3 Hz, 1H), 2.47 (dd, J = 13.3, 8.6 Hz, 1H), 2.29 (s, 3H), 1.85 (dddd, J = 13.9, 10.2, 8.7, 5.2 Hz, 1H), 1.63 (dddd, J = 13.9, 10.2, 6.3, 5.1 Hz, 1H).

^{13}C NMR (126 MHz, $CDCl_3$) δ 142.7, 140.1, 139.4, 131.4, 130.6, 128.6, 128.4, 128.3, 128.3, 128.2, 126.8, 125.7, 63.99, 58.1, 36.3, 34.6, 33.2, 32.3.

IR (film, cm^{-1}): 3024, 2931, 2855, 1787, 1491, 1452, 1089, 1014, 732, 696

HRMS: m/z calculated for $C_{24}H_{27}ClN$ $[M+H]^+$ 364.1832; found 364.1828.

N-benzyl-N-methyl-1,1,4-triphenylbutan-2-amine (10j)

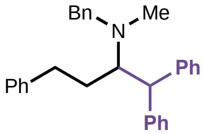

N-benzyl-N-methyl-1,1,4-triphenylbutan-2-amine was prepared according to General Procedure **A** using Zn (52 mg, 0. 8 mmol, 2 equiv), N-methylbenzylamine hydrochloride (63 mg, 0.4 mmol, 1 equiv), hydrocinnamaldehyde (80 µL, 0.6 mmol,1.5 equiv), bromodiphenylmethane (297mg, 1.2 mmol, 3 equiv) and TMSOTf (36 µL, 0.2 mmol, 0.5 equiv) in EtOAc (1 mL). The crude mixture was filtered through an SCX-column (eluting with 5N NH_3 in MeOH) then purified by column chromatography (0-100% (80% Et_2O: 10% Pet. Ether : 3% Et_3N) in Pet. Ether) to afford the product as a colourless oil (68 mg, 42%).

^{1}H NMR (500 MHz, $CDCl_3$) δ 7.39 (dd, J = 7.7, 2.5 Hz, 2H), 7.34 – 7.25 (m, 8H), 7.25 – 7.17 (m, 6H), 7.06 (dd, J = 7.4, 2.8 Hz, 2H), 6.95 (dt, J = 7.2, 2.5 Hz, 2H), 4.10 (dd, J = 11.0, 2.6 Hz, 1H), 3.75 (dd, J = 13.8, 2.6 Hz, 1H), 3.60 (ddt, J = 10.6, 7.0, 3.3 Hz, 1H), 3.50 (dd, J = 13.8, 2.5 Hz, 1H), 2.66 (ddt, J = 16.1, 8.3, 3.9 Hz, 1H), 2.47 – 2.32 (m, 1H), 2.19 (d, J = 2.6 Hz, 3H), 2.02 – 1.86 (m, 1H), 1.78 – 1.62 (m, 1H).

^{13}C NMR (126 MHz, $CDCl_3$) δ 144.5, 143.9, 142.6, 140.6, 128.6, 128.6, 128.4, 128.4, 128.3, 128.3, 128.2, 128.0, 126.5, 126.3, 126.0, 125.7, 66.0, 59.0, 56.9, 35.9, 34.7, 31.9.

IR (film, cm^{-1}): 3023, 2924, 2780, 1599, 1492, 1450, 1353, 1027, 734, 695.

HRMS: m/z calculated for $C_{30}H_{32}N$ $[M+H]^+$ 406.2535; found 406.2542.

Ethyl 1-(1-(benzyl(methyl)amino)-3-phenylpropyl)cyclobutane-1-carboxylate (10k)

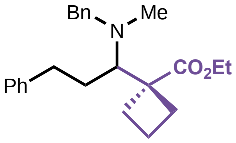

Ethyl 1-(1-(benzyl(methyl)amino)-3-phenylpropyl)cyclobutane-1-carboxylate was prepared according to the general procedure B using zinc (52 mg, 0.8 mmol, 2 equiv), N-methylbenzylamine (52 µL, 0.4 mmol 1 equiv), hydrocinnamaldehyde (79 µL, 0.6 mmol, 1.5 equiv), cyclobutyl bromide ethyl ester (248 µL, 1.2 mmol, 3 equiv) and TMSOTf (108 µL, 0.6 mmol, 1.5 equiv) in EtOAc (1 mL). Crude material was purified by column chromatography (0-15 (10% Et_2O:90% Pet. Ether: NEt_3 3%) in Pet. Ether) to afford the product as a colorless oil (60 mg, 41%).

^{1}H NMR (400 MHz, $CDCl_3$) δ 7.41 – 7.20 (m, 10H), 4.37 – 4.11 (m, 2H), 3.87 – 3.74 (m, 2H), 3.12 (dd, J = 9.2, 3.4 Hz, 1H), 2.97 (ddd, J = 13.5, 10.9, 5.1 Hz, 1H), 2.76 (ddd, J = 13.5, 10.4, 6.2 Hz, 1H), 2.55 – 2.38 (m, 2H), 2.29 – 2.13 (m, 6H), 2.03 – 1.89 (m, 2H), 1.82-1.73 (m, 1H), 1.30 (t, J = 7.1 Hz, 3H).

^{13}C NMR (101 MHz, $CDCl_3$) δ 177.4, 142.6, 140.5, 128.5, 128.39, 128.35, 128.1, 126.7, 125.9, 68.6, 60.3, 59.8, 54.1, 37.4, 35.4, 30.5, 28.5, 28.3, 16.7, 14.2.

IR (film, cm-1): 2976, 2865, 1718, 1452, 1279, 1198, 1105, 1026, 732, 696.

HRMS: m/z calcd for $C_{24}H_{32}NO_2$ $[M+H]^+$ 366.2428; found 366.2434.

Ethyl 3-(benzyl(methyl)amino)-2,2-difluoro-5-phenylpentanoate (10l)

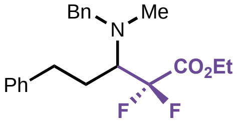

Ethyl 3-(benzyl(methyl)amino)-2,2-difluoro-5-phenylpentanoate was prepared according to the general procedure B using zinc (52 mg, 0.8 mmol, 2 equiv), N-methylbenzylamine (52 µL, 0.4 mmol 1 equiv), hydrocinnamaldehyde (79 µL, 0.6 mmol, 1.5 equiv), ethyl 2-bromo-2,2-difluoroacetate (244 µL, 1.2 mmol, 3 equiv) and TMSOTf (108 µL, 0.6 mmol, 1.5 equiv) in EtOAc (1 mL). Crude material was purified by column chromatography (0-15% (10% Et_2O:90% Pet. Ether: NEt_3 3%) in Pet. Ether) to afford the product as a colorless oil (59 mg, 40%).

^{1}H NMR (400 MHz, $CDCl_3$) δ 7.39 – 7.22 (m, 10H), 4.45 – 4.25 (m, 2H), 3.87 (d, J = 13.4 Hz, 1H), 3.73 (d, J = 13.5 Hz, 1H), 3.49 – 3.32 (m, 1H), 2.94 – 2.74 (m, 2H), 2.33 (s, 3H), 2.18 – 1.98 (m, 2H), 1.32 (t, J = 7.1 Hz, 3H).

^{13}C NMR (126 MHz, $CDCl_3$) δ 164.6 (dd, J = 33.5, 30.7 Hz), 141.5, 139.2, 128.6, 128.5, 128.4, 128.2, 127.1, 126.2, 118.1 (dd, J = 260.9, 256.9 Hz), 64.1 (dd, J = 25.8, 21.1 Hz), 62.5, 59.7, 37.2, 33.4, 25.2, 14.0.

^{19}F NMR (376 MHz, $CDCl_3$) δ -103.8 (d, J = 255.9 Hz), -118.8 (d, J = 255.9 Hz).

IR (film, cm^{-1}): 3061, 2955, 2864, 1758, 1678, 1495, 1063, 748, 698.

HRMS: m/z calcd for $C_{21}H_{26}F_2NO_2$ $[M+H]^+$ 362.1927; found 362.1919.

N-benzyl-*N*-methyl-4-phenylbutan-2-amine (10m)

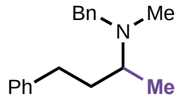

The synthesis of N-benzyl-N-methyl-4-phenylbutan-2-amine was attempted according to General Procedure **B** using Zn (52 mg, 0. 8 mmol, 2 equiv), N-methylbenzylamine (52 µL, 0.4 mmol, 1 equiv), hydrocinnamaldehyde (106 µL, 0.8 mmol, 2 equiv), 2-bromopropane (112 µL, 1.2 mmol, 3 equiv) and TMSOTf (36 µL, 0.2 mmol, 0.5 equiv) in EtOAc (1 mL). Yield of the product was determined by ^{1}H NMR using 1,1,2,2-tetrachloroethane as internal standard indicating a 9% yield based on alpha-methyl peak at 1.10 ppm.

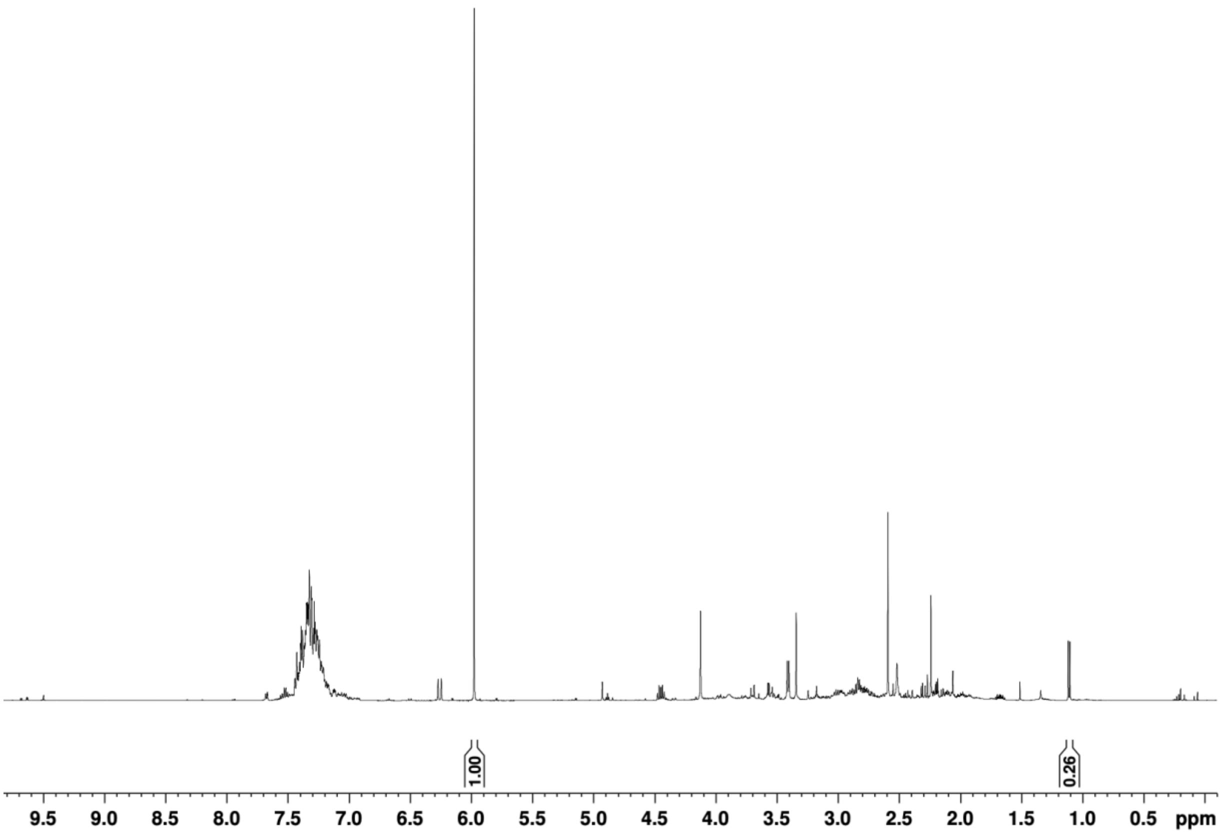

N-Benzyl-N-methyl-1-phenylhexan-3-amine (10n)

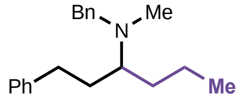

The synthesis of N-Benzyl-N-methyl-1-phenylhexan-3-amine was attempted according to General Procedure **B** using Zn (52 mg, 0. 8 mmol, 2 equiv), N-methylbenzylamine (52 µL, 0.4 mmol, 1 equiv), hydrocinnamaldehyde (106 mL, 0.8 mmol, 2 equiv), 1-iodopropane (117 µL, 1.2 mmol, 3 equiv) and TMSOTf (144 µL, 0.8 mmol, 2 equiv) in EtOAc (1 mL). Yield of the product was determined by ^{1}H NMR using 1,1,2,2-tetrachloroethane as internal standard indicating a 23% yield based on the alpha proton peak at 3.70 ppm.

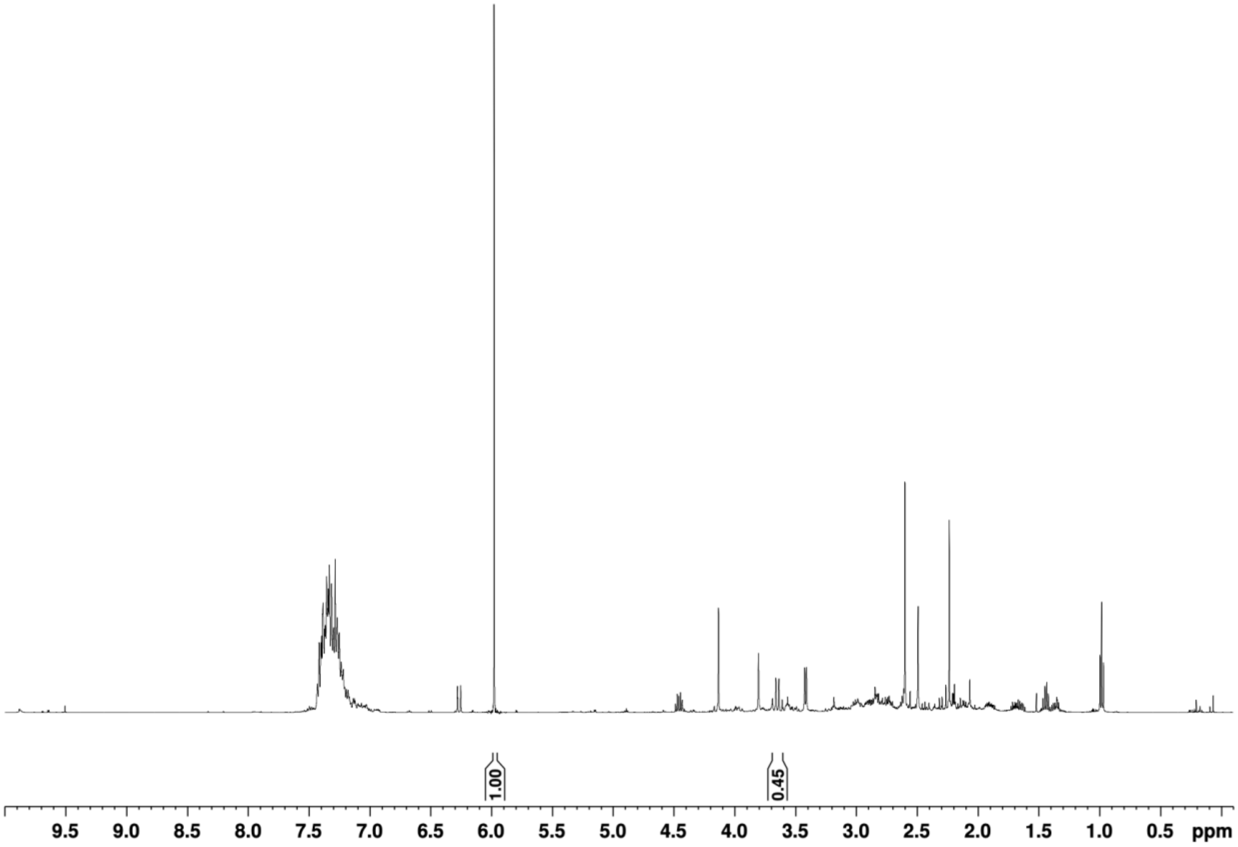

N-benzyl-N,4,4-trimethyl-1-phenylpentan-3-amine (10o)

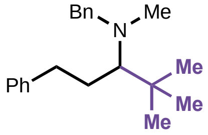

The synthesis of N-benzyl-N,4,4-trimethyl-1-phenylpentan-3-amine was attempted according to General Procedure **B** using Zn (52 mg, 0. 8 mmol, 2 equiv), N-methylbenzylamine (52 µL, 0.4 mmol, 1 equiv), hydrocinnamaldehyde (106 µL, 0.8 mmol, 2 equiv), tertbutyliodide (143 µL, 1.2 mmol, 3 equiv) and TMSOTf (108 µL, 0.6 mmol, 1.5 equiv) in EtOAc (1 mL). Yield of the product was determined by ^{1}H NMR using 1,1,2,2-tetrachloroethane as internal standard indicating a 35% yield based on the diastereotopic benzyl proton peak at 3.95 ppm.

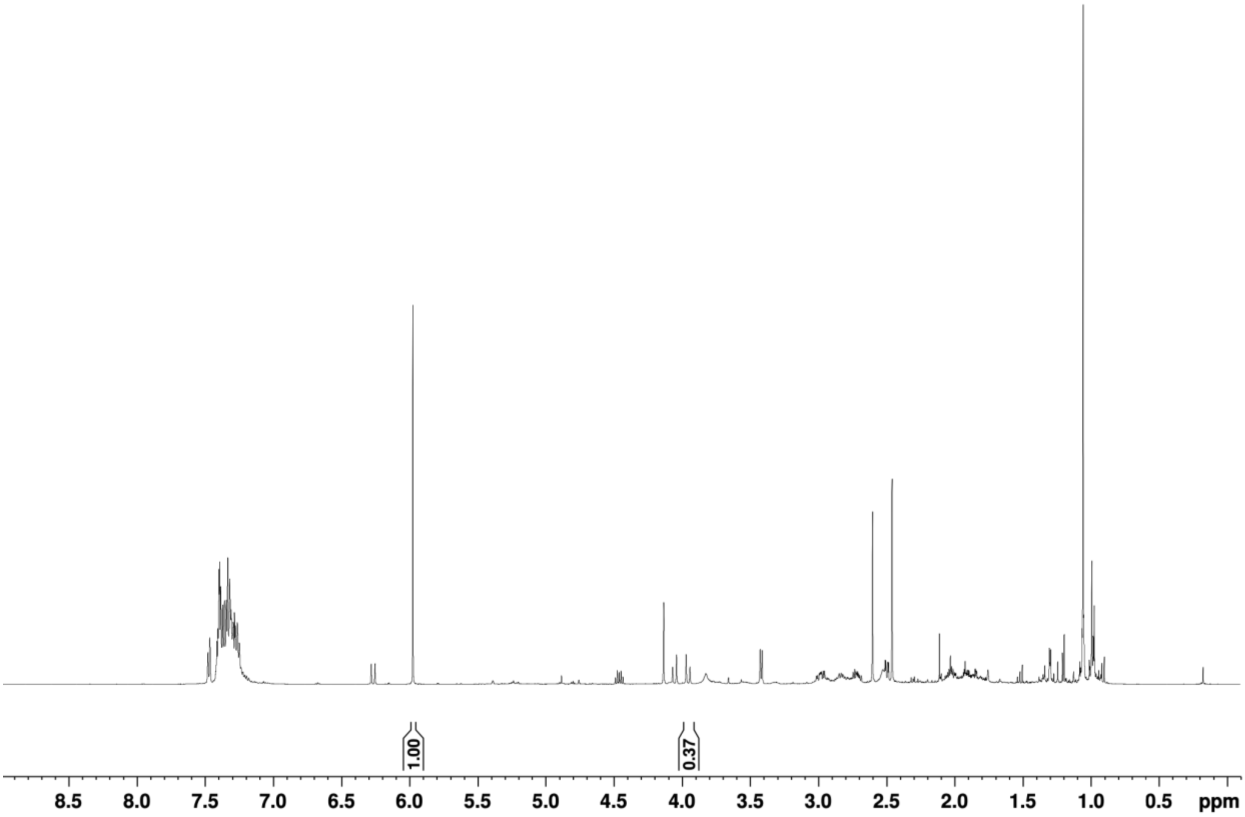

3-(benzyl(methyl)amino)-N,N-dimethyl-5-phenylpentanamide (10p)

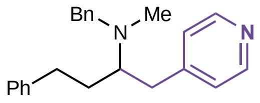

The synthesis of 3-(benzyl(methyl)amino)-N,N-dimethyl-5-phenylpentanamide was attempted according to General Procedure **B** using Zn (52 mg, 0. 8 mmol, 2 equiv), N-methylbenzylamine (52 µL, 0.4 mmol, 1 equiv), hydrocinnamaldehyde (106 µL, 0.6 mmol, 1.5 equiv), 4-(Bromomethyl)-pyridine hydrobromide (304 mg, 1.2 mmol, 3 equiv) and TMSOTf (36 µL, 0.2 mmol, 0.5 equiv) in EtOAc (1 mL). Yield of the product was determined by ^{1}H NMR using 1,1,2,2-tetrachloroethane as internal standard indicating a 26% yield based on the diastereotopic benzyl proton peak at 3.74 ppm.

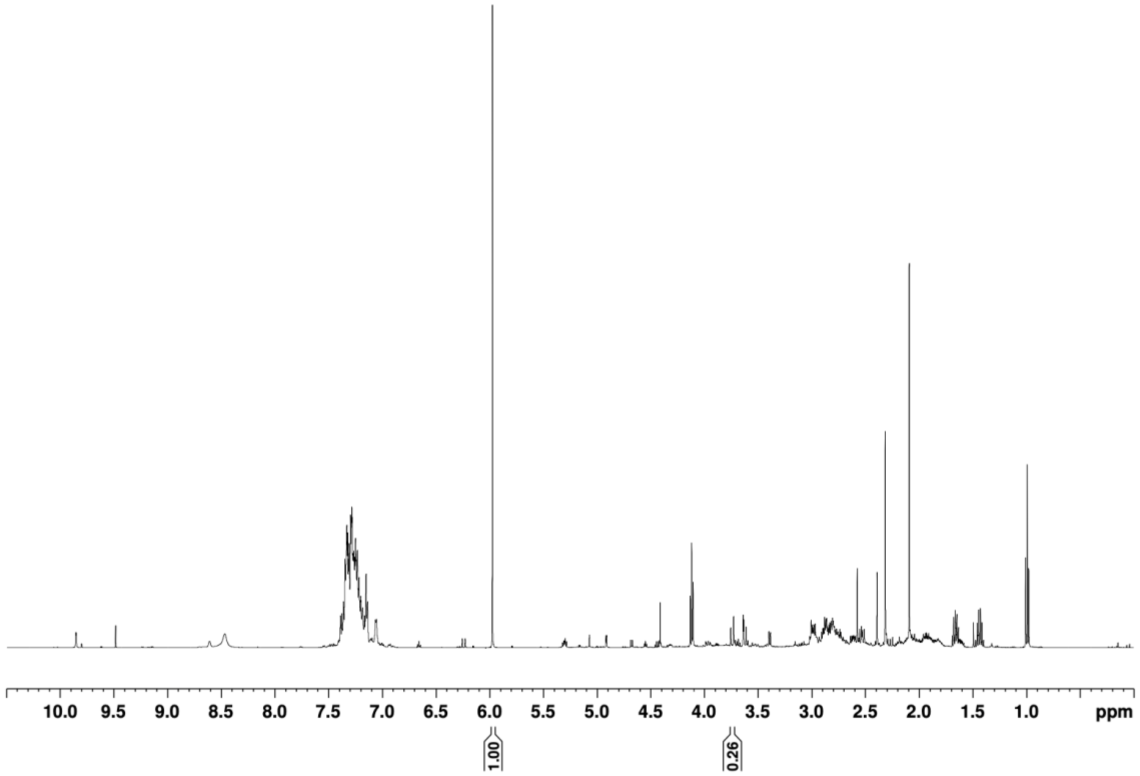

6. High-throughput experimentation (HTE)

6.1 HTE general experimental

Microscale reactions were performed in Axygen 384-well, nonsterile, clear V- bottom 120 μL, polypropylene deep well non-treated plates (catalogue number: AXY-P-384-120SQ-C). Liquid dosing for was performed using IntegraTM single channel VIAFLO II electronic pipettes (300 and 12.5 μL, Cat. No. 4013 and 4011) and IntegraTM 8-channel VIAFLO electronic pipettes (125 μL, Cat. No. 4622) inside a Bel-ArtTM SP ScienceTM sidENTRYTM Clamping Ring Glove Box (Fischersci, Cat. No. 15641814) under a N_2 atmosphere. Liquid plate transfers were performed using an Integra VIAFLO 96 electronic pipette (Cat. No. 6001) using a 5-125 μL 96 pipette head (Cat. No. 6102). Reaction shaking was performed on a Heidolph UK Vibramax 100 (Cat. No. 544-21200-00). Centrifugation and centrifugal evaporation were performed using a SP Genevac EZ-2 Elite. For filtration receiver plates, Greiner Masterblock 96-well 500 μL, non-sterile, v-bottom polypropylene plates (Cat. No. 786201) were used. For microplate filtering, Pall Acroprep Advance 96-well 1mL filter plates (polypropylene, 1μm glass fibre, short tip, Cat. No. 8131) were used.

MP-isocyanate resin was purchased from Biotage, sulfonyl hydrazine resin was purchased from Sigma Aldrich and tris(aminoethylamine) polymer supported resin was purchased from Aldrich. All other resins were purchased from Sigma Aldrich or Fluka. All resins were washed with EtOAc before use. Amberlite IRA-96 was ground using a pestle and mortar into a fine powder then washed and stored under nitrogen to prevent CO_2 adsorption. Stock solutions were prepared in Supleco 4 mL, screw top glass vials (Sigma Aldrich, Cat. No. 854190).

Custom Nanonest reactors were built using commercial Analytical Sales NanoNest reactors (Cat. No. 1626100) with a plastic plate border gasket (Cat. No.1626010) and an acrylic riser (Cat. No. 162611) with two silicone matt bottom inserts (Cat. No. 1626004) as shown in General Procedure **D**.

6.2 Solvent screening

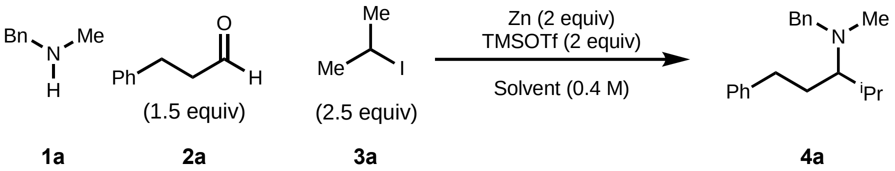

To a 4 mL vial, zinc powder (26 mg, 0.40 mmol, 2 equiv) was added. The vial was sealed, evacuated, and backfilled with N_2. To the vial, *n*BuOAc (500 µL) was added, followed by *N*-methylbenzylamine (25.8 µL, 0.2 mmol, 1 equiv), hydrocinamaldehyde (39.9 µL, 0.3 mmol, 1.5 equiv) and isopropyl iodide (49.9 µL, 0.5 mmol, 2.5 equiv). The mixture was stirred for 1 min then TMSOTf (72.8 µL, 0.4 mmol, 2 equiv) was added. The reaction mixtures were stirred at room temperature for 16 h, then reaction mixture was loaded onto a plug of vacuum-packed resin. After the desired reaction time had passed, the solution was filtered under vacuum and the resin washed with EtOAc (800 µL). The resulting solution was concentrated *in vacuo* to yield the crude reaction mixture.

**Solvent**	**Yield / %**
EtOAc	82
iPrOAc	84
EtOiPr	67
DMSO	39
γ-Valerolactone	13
δ-Valerolactone	10
ε-valerolactone	24
Cyrene	57
Methylbutyrate	68
Propylene carbonate	41
Isobutanitrile	52
*n*PrOAc	86
*n*BuOAc	87
Diethyl carbonate	81
Ethyl propionate	32
Methyl benzoate	86

6.3 Zinc scavenging optimization

6.3.1 Identification of need for zinc scavenging

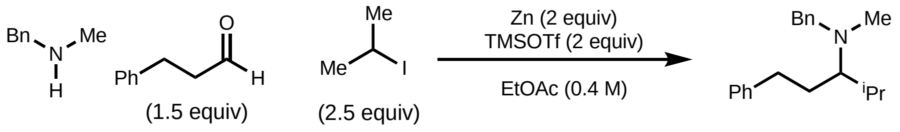

1a **2a** **3a** **4a**

Two samples were prepared according to the general procedure below:

An oven dried vial (Biotage Microwave Process Vial (10 mL), equipped with gas-tight pierceable caps) was charged with a stirring bar, and Zn dust (52 mg, 0.4 mmol, 2 equiv). The vial was sealed, evacuated and backfilled with N_2. To this was added EtOAc (1 mL), followed by N-methylbenzylamine (26 μL, 0.2 mmol, 1.0 equiv), hydrocinnamaldehyde (78 μL, 0.4 mmol, 1.5 equiv) and 2-iodopropane (50 μL, 0.5 mmol, 2.5 equiv). The reaction mixture was stirred for 10s, followed by the addition of TMSOTf (72 μL, 0.4 mmol, 2 equiv). The reaction mixture was stirred for 16 hours.

Sample A – The vial was uncapped and transferred to a 50 mL rbf. Dichloromethane (approx. 20 mL) was used to rinse the vial. The solvent was removed *in vacuo*. The resulting crude was dissolved in $CDCl_3$, and the mixture analysed by ^{1}H NMR using 1,1,2,2-tetrachloroethane as internal standard.

Sample B- The crude mixture was worked up according to General procedure **B**. The resulting crude was dissolved in $CDCl_3$, and the mixture analysed by ^{1}H NMR using 1,1,2,2-tetrachloroethane as internal standard.

Sample A: unquenched reaction mixture ^{1}H NMR

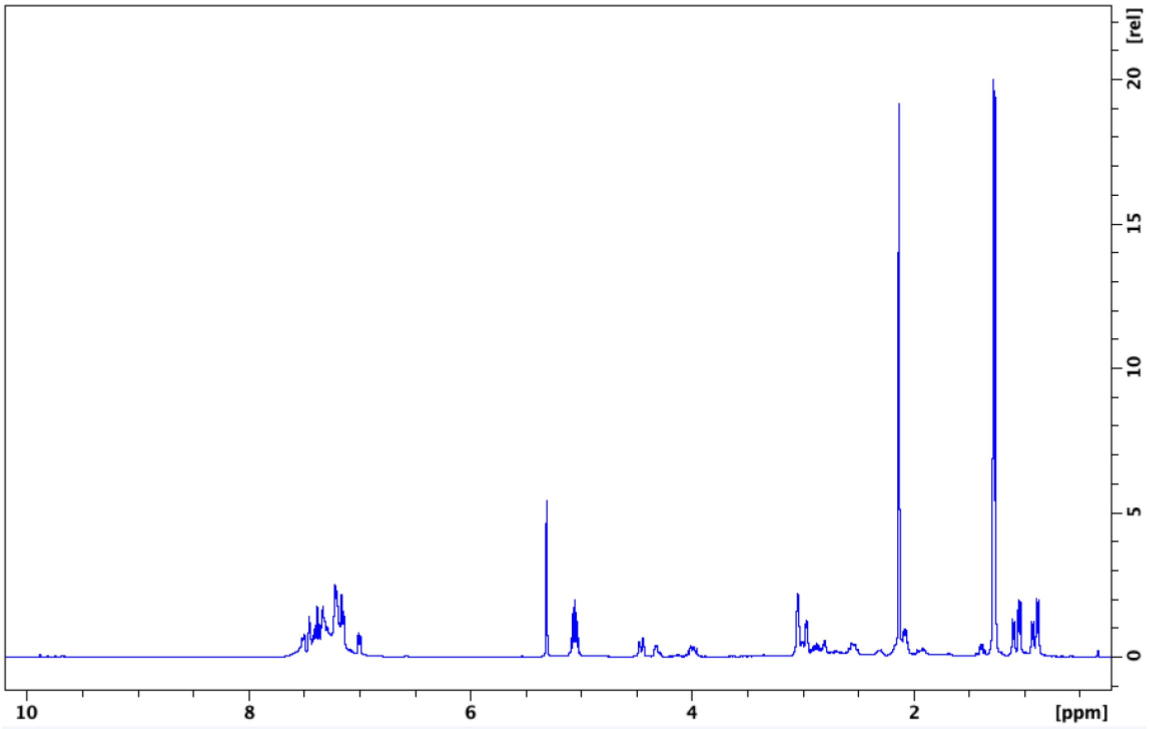

Sample B: quenched reaction mixture ^{1}H NMR

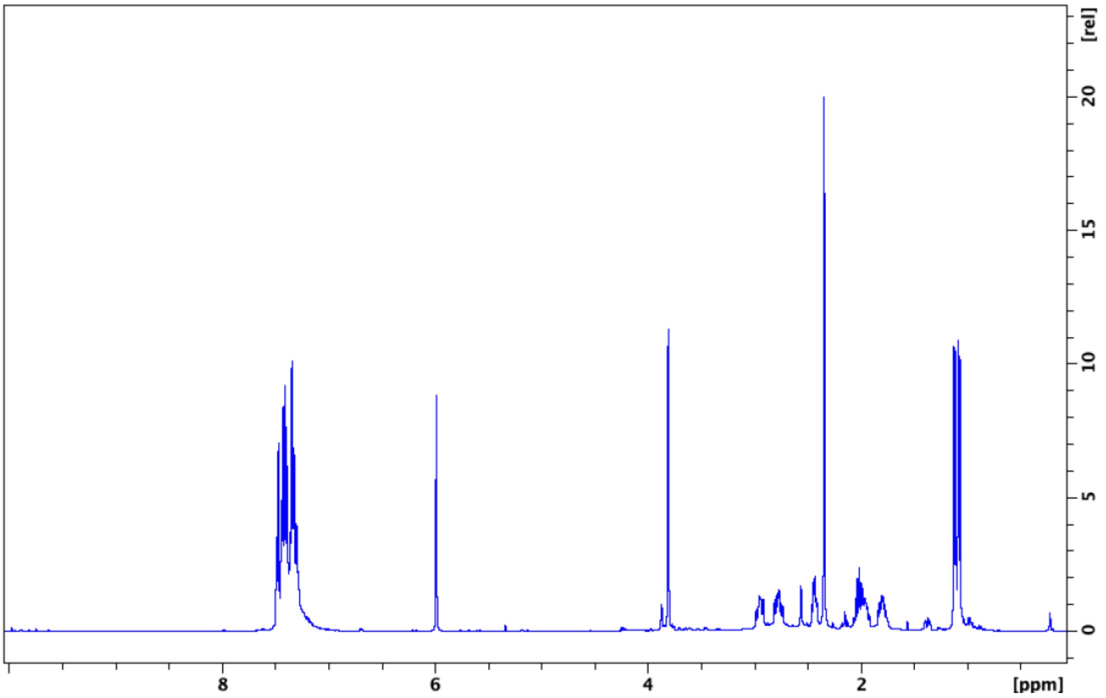

Stacked ^{1}H NMRs of unquenched (red) and quenched (blue) reaction mixtures with diagnostic peaks indicated.

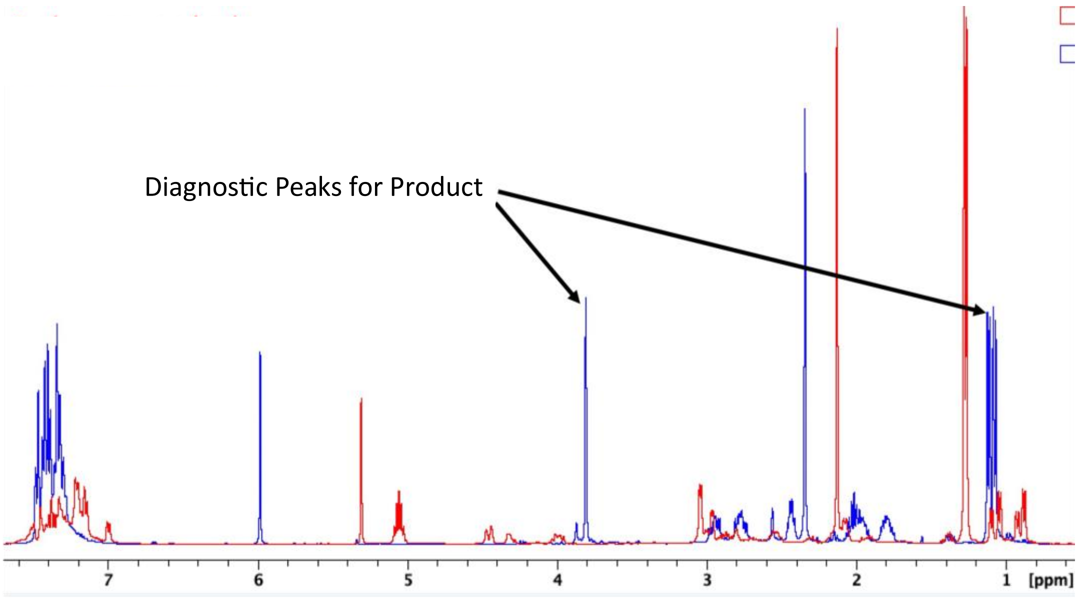

6.3.2 Resin quenching screening

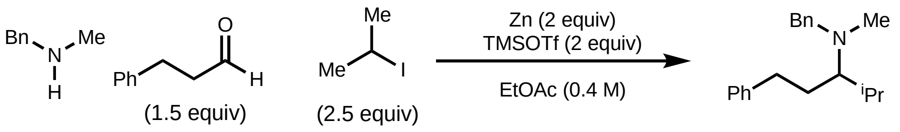

1a **2a** **3a** **4a**

To a 4 mL vial, zinc powder (26 mg, 0.4 mmol, 2 equiv) was added. The vial was sealed, evacuated, and backfilled with N_2. To the vial, *n*BuOAc (500 µL) was added, followed by N-methylbenzylamine (26 µL, 0.2 mmol, 1 equiv), hydrocinamaldehyde (40 µL, 0.3 mmol, 1.5 equiv) and 2-iodopropane (50 µL, 0.5 mmol, 2.5 equiv). The mixture was stirred for 1 min then TMSOTf (73 µL, 0.4 mmol, 2 equiv) was added. The reaction mixtures were stirred at room temperature for 16 h, then reaction mixture was loaded onto a plug of vacuum-packed resin. After the desired reaction time had passed, the solution was filtered under vacuum and the resin washed with EtOAc (800 µL). The resulting solution was concentrated under reduced pressure to yield the crude reaction mixture.

**Quenching Agent**	**Mass / g**	**Time on resin / h**	**Diagnostic peak identified in ^{1}H NMR**
Aminomethyl polystyrene	0.278	0.5	No
Rink amide resin	0.187	1.0	Yes
Basified Amberlite 120-H	0.094	1.0	No
Activated charcoal	0.200	1.0	No
Amino functionalised silica	0.200	1.0	No
Amberlite IRA-400	1.000	2.0	No
Amberlite IRA-96	1.200	2.0	Yes
Amberlite IRA-743	1.200	2.0	Yes
Amberlyst A-21	0.800	2.0	Yes
Amberlite IRA-96	0.400	3.0	Yes
Amberlite IRA-743	0.400	3.0	No
Amberlyst A-21	0.400	3.0	Yes

6.3.3 Alternative reaction quenching strategies screening

Catch and release:

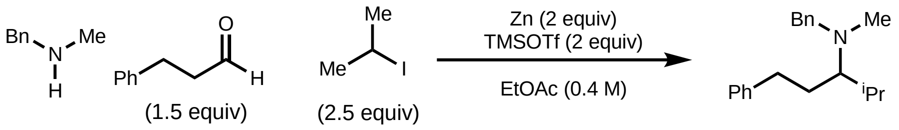

To a 4 mL vial, zinc powder (26 mg, 0.40 mmol, 2 equiv) was added. The vial was sealed, evacuated and backfilled with N_2. To the vial, *n*BuOAc (500 µL) was added, followed by N-methylbenzylamine (26 µL, 0.2 mmol, 1 equiv), hydrocinamaldehyde (40 µL, 0.3 mmol, 1.5 equiv) and 2-iodopropane (50 µL, 0.5 mmol, 2.5 equiv). The mixture was stirred for 1 min then TMSOTf (73 µL, 0.4 mmol, 2 equiv) was added. The reaction mixtures were stirred at room temperature for 16 h. Then a purification method was attempted, the resulting mixtures concentrated *in vacuo*.

Resin	Mass	Time on resin / h	Elution solvent	Diagnostic product peaks seen in ^{1}H NMR
HyperSep SCX cartridge (cat: 60108-420)	100 mg cartridge	1	7 M NH_3 in MeOH	Yes
Amberlite 120-H	400 mg	4	7 M NH_3 in MeOH	No

Zinc complexation and removal:

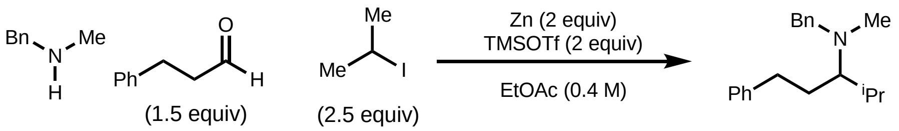

To a 4 mL vial, zinc (26 mg, 0.40 mmol, 2 equiv) was added under inert conditions. To the vial, *n*BuOAc (500 µL) was added, followed by N-methylbenzylamine (26 µL, 0.2 mmol, 1 equiv), 3-phenylpropionaldehyde (40 µL, 0.3 mmol, 1.5 equiv) and 2-iodopropane (50 µL, 0.5 mmol, 2.5 equiv). The mixture was stirred for 1 min then TMSOTf (73 µL, 0.4 mmol, 2 equiv) was added. The reaction mixtures were stirred at room temperature for 16 h. To the reaction mixture, complexation agent was added, and the mixture stirred for time denoted bellow. The resulting mixture was filtered to remove precipitate and concentrated under reduced pressure.

Quenching agent	Mass / mg	Equiv	Time reacting in solution / h	Filtration agent	Diagnostic product peaks seen in ^{1}H NMR
Triphenyl phosphine oxide	278	5	0.5	Celite	No
2,2'-bipyridine	187	6	1	Celite	Yes
2,2'-bipyridine	94	3	1	Celite	No

Aqueous quench:

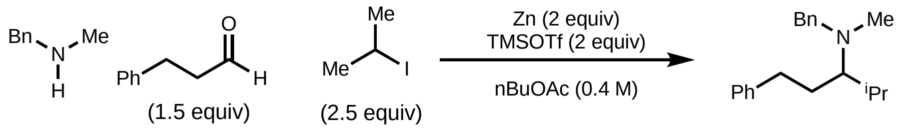

1a **2a** **3a** **4a**

To a 4 mL vial, zinc (26 mg, 0.40 mmol, 2 equiv) was added. The vial was sealed, evacuated and backfilled with N_2. To the vial, *n*BuOAc (500 µL) was added, followed by N-methylbenzylamine (26 µL, 0.2 mmol, 1 equiv), hydrocinamaldehyde (40 µL, 0.3 mmol, 1.5 equiv) and 2-iodopropane (50 µL, 0.5 mmol, 2.5 equiv). The mixture was stirred for 1 min then TMSOTf (73 µL, 0.4 mmol, 2 equiv) was added. The reaction mixtures were stirred at room temperature for at least 16 h to ensure reaction completion. Then a quenching method was attempted, the resulting mixtures concentrated under reduced pressure.

Quenching agent	Amount	Reaction time / h	Diagnostic product peaks seen in ^{1}H NMR
10 % NaOH solution	500 µL	0.5	Yes
10 % NaOH loaded onto celite	2 mL on approx. 1 g celite	2	No

6.3.4 Amberlite IRA-96 quenching optimization

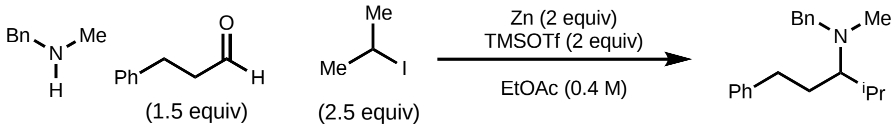

1a **2a** **3a** **4a**

An oven dried vial (Biotage Microwave Process Vial (10 mL), equipped with gas-tight pierceable caps) was charged with a stirring bar, and zinc (52 mg, 0.4 mmol, 2 equiv). The vial was sealed, evacuated and backfilled with N_2. To this was added EtOAc (1 mL), followed by N-methylbenzylamine (26 µL, 0.2 mmol, 1.0 equiv), hydrocinnamaldehyde (78 µL, 0.4 mmol, 1.5 equiv) and 2-iodopropane (50 µL, 0.5 mmol, 2.5 equiv). The reaction mixture was stirred for 10s, followed by the addition of TMSOTf (73 µL, 0.4 mmol, 2 equiv). The reaction mixture was stirred for 16 hours. Then a quenching method was attempted, the resulting mixtures concentrated under reduced pressure.

Time reaction mixture exposed to quenching agent	Amount of quenching agent / mg	Minimum equiv of resin to reactive species	Diagnostic product peaks seen in ^{1}H NMR
5 min	1000	2.28	Yes
5 min	750	1.70	Yes
5 min	600	1.37	Yes
5 min	550	1.25	No
5 min	500	1.14	No
4 h	600	1.37	Yes
4 h	550	1.25	Yes
4 h	500	1.14	Yes
4 h	450	1.02	Yes
4 h	400	0.91	Yes
4 h	350	0.80	No

6.4 Modified Nanonest and Sealing Method (General Procedure D)

6.4.1 Modified NanoNest Design

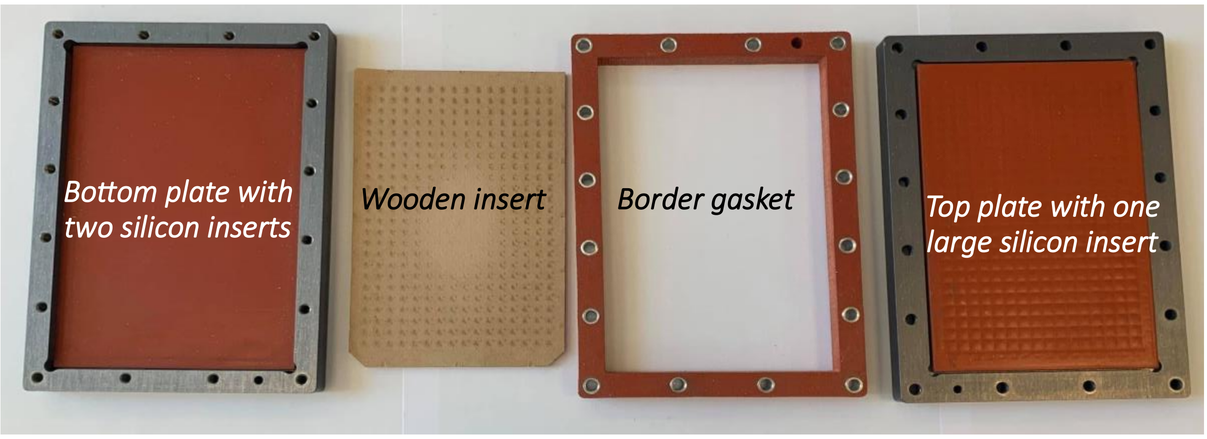

Figure S4: The Nanonest enclosure separated into its composite parts

6.4.2 Plate sealing procedure.

A PFA mat (0.125 mm thickness, FLONFILMTM 600 PFA film) was placed on top of the microplate wells and sealed with electrical tape around the edges (see figure below). The plate was placed onto two silicon matts inside the bottom NanoNest plate. A silicon gasket was placed around the plate and a further PFA sheet and silicon matt placed on top of the microplate. The top aluminium plate was used to compress the apparatus through gradual tightening of 12 screws in a cross wise pattern.

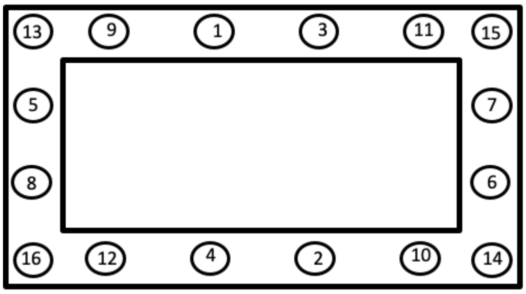

The order the NanoNest enclosure screws were turned.

6.4.2 Demonstration of sealing,

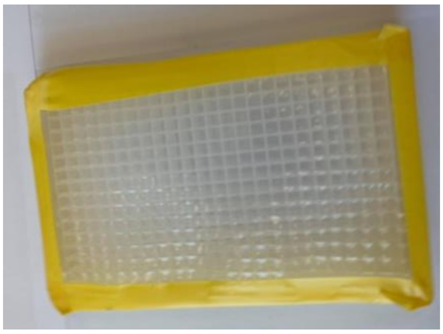

1. The microplate is sealed with a PFA sheet by electrical tape.

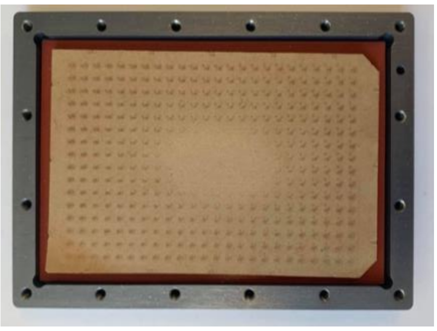

2. The wooden insert is placed inside the bottom plate.

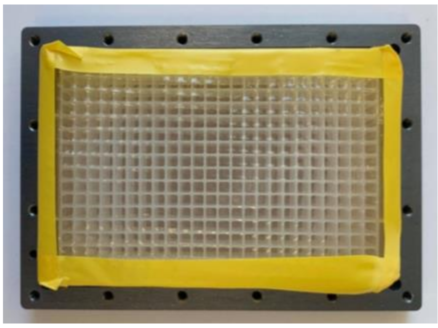

4. The microplate is placed on top of the wooden insert.

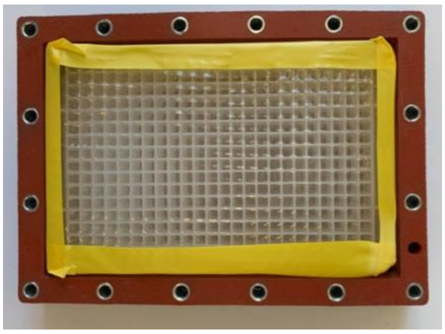

3. The gasket is placed around the microplate.

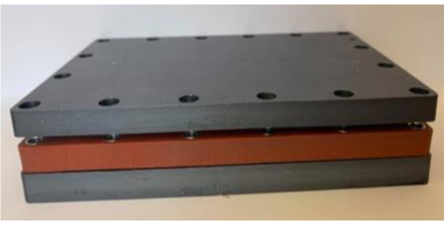

5. A second PFA sheet is placed ontop of the microplate and then the top plate is placed on top.

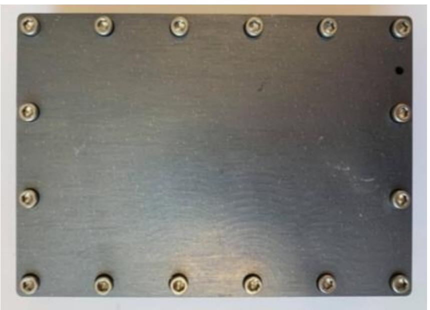

6. The enclosure is secured and compressed with bolts (following General Procedure **D**)

6.5 Multi amine array validation experiment

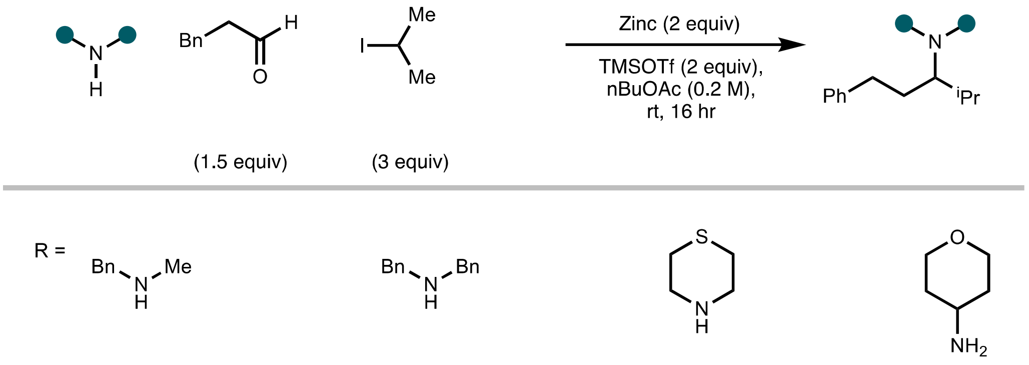

1 **2** **3** **4**

Stock solutions of N-methylbenzylamine (0.8 M), thiomorpholine (0.8 M), dibenzylamine (0.8 M), 4-aminotetrahydrofuran (0.8 M), 2-iodopropane (2.4 M), hydrocinamaldehyde (1.2 M) and TMSOTf (1 M) in *n*BuOAc were prepared. To a 384 well microplate, Zn dust (20 µmol) was dosed into the wells via a zinc:*n*BuOAc slurry (12.5 µL, 10 %w/v) using an electronic pipette and the solvent removed via centrifugal evaporation. Then, stirrer bars (2 mm x 2 mm) were added to each well. The stock solutions and plate were transferred inside of the purge box and placed under a nitrogen atmosphere (5 x vacuum back refill). Amine (12.5 µL) and aldehyde (12.5 µL) stock solutions were dosed into their corresponding wells and then mixed (5 x 17.5 µL mix volume, 20 µL aspirate volume, integra pipette mix function). Then, alkyl halide (12.5 µL) and TMSOTf (12.5 µL) stock solutions were dosed, in order, to their corresponding wells. The plate was sealed according to General Procedure **D** and taken out of the purge box. The Nanonest was placed onto a hot plate and stirred at 1000 rpm.

After 24 h, the Nanonest was left to cool to room temperature, then opened and the PFA sealed plate was centrifuged for 10s. Then the PFA film was removed, and the reaction mixtures were transferred onto Amberlite IRA-96 resin (100 mg) loaded into a 96 well filter plate. The reaction wells were washed with two reaction volumes of EtOAc. The filter plate was placed onto of a 96 well receiver plate and centrifuged at 10,000 rpm for 10 s. The resin was washed with EtOAc (300 µL) and centrifuged again. The resin was washed again with EtOAc (50 µL) and centrifuged for 20s. The solvent was removed using the "high + low boiling point" setting on the Genevac EZ-2 elite with a 40 min 'time to final stage' and a 20 min final stage with a maximum temperature of 40 ˚C. The concentrated reaction mixtures were transferred into the purge box and placed under a nitrogen atmosphere (5 x vacuum back refill).

To each well internal standard (1 µL, 1,1,2,2-tetrachloroethane) was dosed and then $CDCl_3$ (60 µL) were added. All 12 repeat wells were added to the same NMR tube (7 mm). Each well was rinsed with additional $CDCl_3$ (60 µL) and added to the same NMR tube. Yields were determined as an average across all 12 reactions against the internal standard.

**Entry**	**Amine**	**Yield / %**
1	N-methylbenzylamine	80
2	thiomorpholine	*97*
3	dibenzylamine	*62*
4	4-aminotetrahydrofuran	*84*

6.6 Multi halide array validation experiment

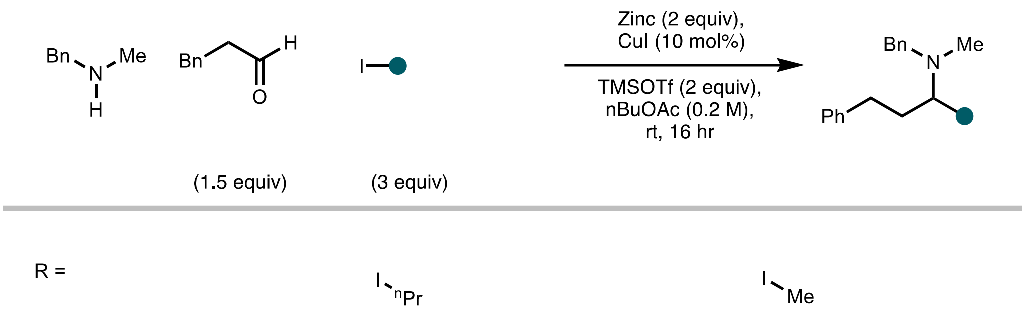

1 2

Stock solutions of N-methylbenzylamine (0.8 M), 1-iodopropane (2.4 M), methyl iodide (2.4 M), hydrocinamaldehyde (1.2 M) and TMSOTf (1 M) in *n*BuOAc were prepared. To a 384 well microplate, Zn dust (20 µmol) was dosed into the wells via a zinc:*n*BuOAc slurry (12.5 µL, 10 %w/v) using an electronic pipette and the solvent removed via centrifugal evaporation. Then, stirrer bars (2 mm x 2 mm) were added to each well. The stock solutions and plate were transferred inside of the purge box and placed under a nitrogen atmosphere (5 x vacuum back refill). Amine (12.5 µL) and aldehyde (12.5 µL) stock solutions were dosed into their corresponding wells and then mixed (5 x 17.5 µL mix volume, 20 µL aspirate volume, integra pipette mix function). Then, alkyl halide (12.5 µL) and TMSOTf (12.5 µL) stock solutions were dosed, in order, to their corresponding wells. Each amine was repeated 12 times. The plate was sealed according to General Procedure **D** and taken out of the purge box. The Nanonest was placed onto a hot plate and stirred at 1000 rpm.

After 24 h, the Nanonest was left to cool to room temperature, then opened and the PFA sealed plate was centrifuged for 10s. Then the PFA film was removed, and the reaction mixtures were transferred onto Amberlite IRA-96 resin (100 mg) loaded into a 96 well filter plate. The reaction wells were washed with two reaction volumes of EtOAc. The filter plate was placed onto of a 96 well receiver plate and centrifuged at 10,000 rpm for 10 s. The resin was washed with EtOAc (300 µL) and centrifuged again. The resin was washed again with EtOAc (50 µL) and centrifuged for 20s. The solvent was removed using the "high + low boiling point" setting on the Genevac EZ-2 elite with a 40 min 'time to final stage' and a 20 min final stage with a maximum temperature of 40 °C. The concentrated reaction mixtures were transferred into the purge box and placed under a nitrogen atmosphere (5 x vacuum back refill).

To each well internal standard (1 µL, 1,1,2,2-tetrachloroethane) was dosed and then $CDCl_3$ (60 µL) were added. All 12 repeat wells were added to the same NMR tube (7 mm). Each well was rinsed with additional $CDCl_3$ (60 µL) and added to the same NMR tube. Yields were determined as an average across all 12 reactions against the internal standard.

**Entry**	**Halide**	**Yield / %**
1	1-iodopropane	23
2	methyl iodide	*9*

6.7 Scavenger resin purification optimization

6.7.1 Secondary amine removal resin optimization.

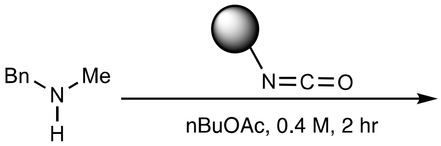

MP-isocyanate resin (x g) was added to a solution of N-methylbenzylamine (26 µL, 0.2 mmol, 1 equiv) in *n*BuOAc (500 µL). After 2 h, the solution was filtered under vacuum and the resin washed with EtOAc (800 µL). The resulting solvent was removed *in vacuo* to yield the residual amine.

Mass of resin /g	Resin equiv	Amine remaining / %
0.40	2.6	3
0.30	1.9	4
0.20	1.3	7

6.7.2 Secondary amine removal resin optimization.

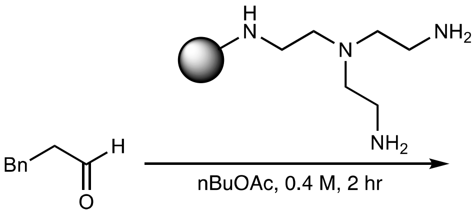

The resin (x g) was added to a solution of hydrocinamaldehyde (1 equiv) in *n*BuOAc (500 µL). After 2 h, the solution was filtered under vacuum and the resin washed with EtOAc (800 µL). The resulting solvent was removed *in vacuo* to yield the residual aldehyde.

Resin	Hydrocinamaldehyde / mmol	Mass of resin /g	Resin equiv	Aldehyde remaining / %
Tris(aminoethylamine) polymer bound	0.30	0.40	2.3	14
Tris(aminoethylamine) polymer bound	0.30	0.20	1.7	72
Tris(aminoethylamine) polymer bound	0.10	0.20	3.5	3
Tris(aminoethylamine) polymer bound	0.10	0.10	1.75	3
Sulfonyl hydrazine resin	0.30	0.40	1.73	5

6.7.3 Multi-resin purification screening:

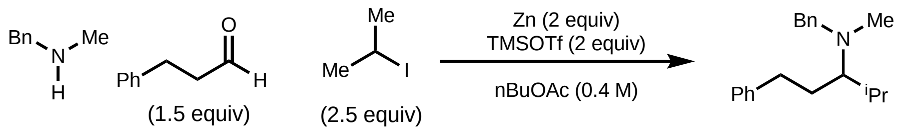

1a **2a** **3a** **4a**

To a 4 mL vial, zinc (26 mg, 0.40 mmol, 2 equiv) was added. The vial was sealed, evacuated and backfilled with N_2. To the vial, *n*BuOAc (500 µL) was added, followed by N-methylbenzylamine (26 µL, 0.2 mmol, 1 equiv), hydrocinamaldehyde (40 µL, 0.3 mmol, 1.5 equiv) and 2-iodopropane (50 µL, 0.5 mmol, 2.5 equiv). The mixture was stirred for 1 min then TMSOTf (73 µL, 0.4 mmol, 2 equiv) was added. The reaction mixtures were stirred at room temperature for at least 16 h to ensure reaction completion. The reaction mixture was then subjected to one of the methods outlined below:

3 resins layered:

A reaction mixture was loaded onto layered (in respective order) Amberlite-IRA96 (600 mg), tris(aminoethyl)amine polymer based (400 mg) and MP-isocyanate resin (200 mg). The mixture was left on the resins for 2 hours before being pulled through with vacuum and the resins were washed with EtOAc (800 µL) before the resulting solvent was removed *in vacuo* to yield the crude product.

4 resins layered:

A reaction mixture was loaded onto a premixed mixture of Amberlite-IRA96 (600 mg), tris(aminoethyl)amine polymer based (400 mg), MP-isocyanate resin (200 mg) and activated charcoal (66 mg). The mixture was left on the resins for 2 hours before being pulled through with vacuum and the resins were washed with EtOAc (800 µL) before the resulting solvent was removed *in vacuo* to yield the crude product.

2 independent filter steps:

A reaction mixture was loaded onto Amberlite-IRA96 (600 mg) for 5 mins and filtered under vacuum. The resin was washed with EtOAc (800 µL), and the resulting solvent removed *in vacuo*. The crude material was redissolved in nBuOAc (500 µL) and loaded onto a mix of tris(aminoethyl)amine polymer based (400 mg) and MP-isocyanate resin (200 mg) and activated charcoal (66 mg). The mixture was left on the resins for 2 hours before being pulled through with vacuum and the resins were washed with EtOAc (800 µL) before the resulting solvent was removed *in vacuo* to yield the crude product.

The resulting crudes were analysed by ^{1}H NMR focusing on the removal of the starting material amine, aldehyde, and a small minority of aldol by-products (shown below).

Stacked ^{1}H NMR (0-4 ppm) highlighting the product (blue) and the staring material amine (beige) after each workup method. Cleanest product is obtained from the multistep quenching and purification workflow.

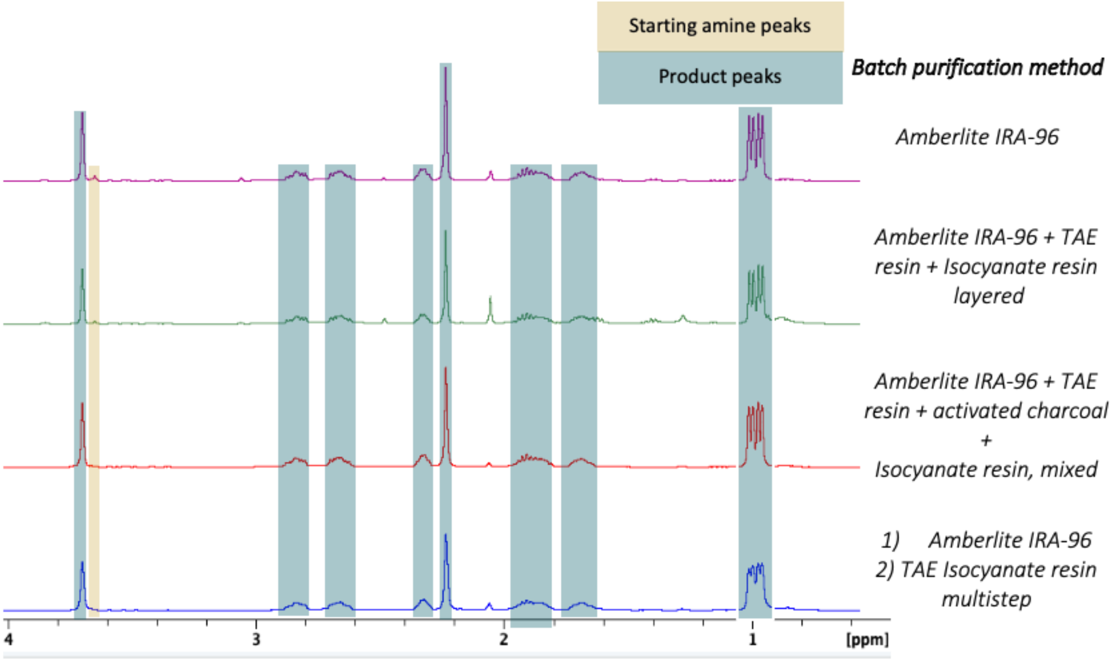

Stacked ^{1}H NMR (10.1-9.3 ppm) highlighting the starting material aldehyde (blue) after each workup method. Cleanest product is obtained from the multistep quenching and purification workflow with removal of the majority of aldol side products.

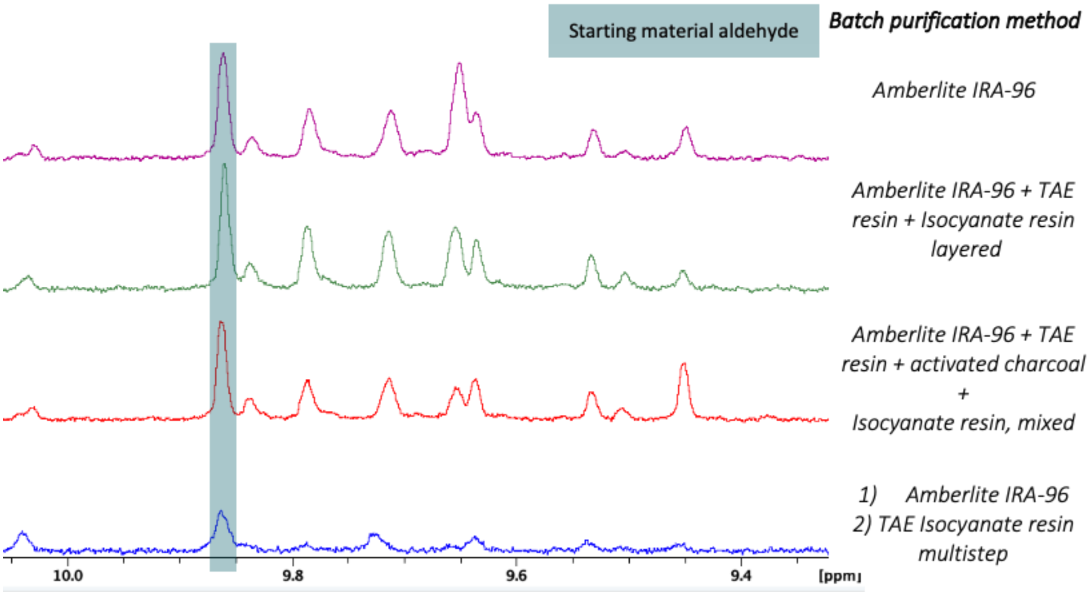

The full ^{1}H NMR for each purification trial outcomes are shown below in $CDCl_3$ using 1,1,2,2-tetrachloroethane as an internal standard.

The ^{1}H NMR of the reaction mixture after quenching using Amberlite IRA-96.

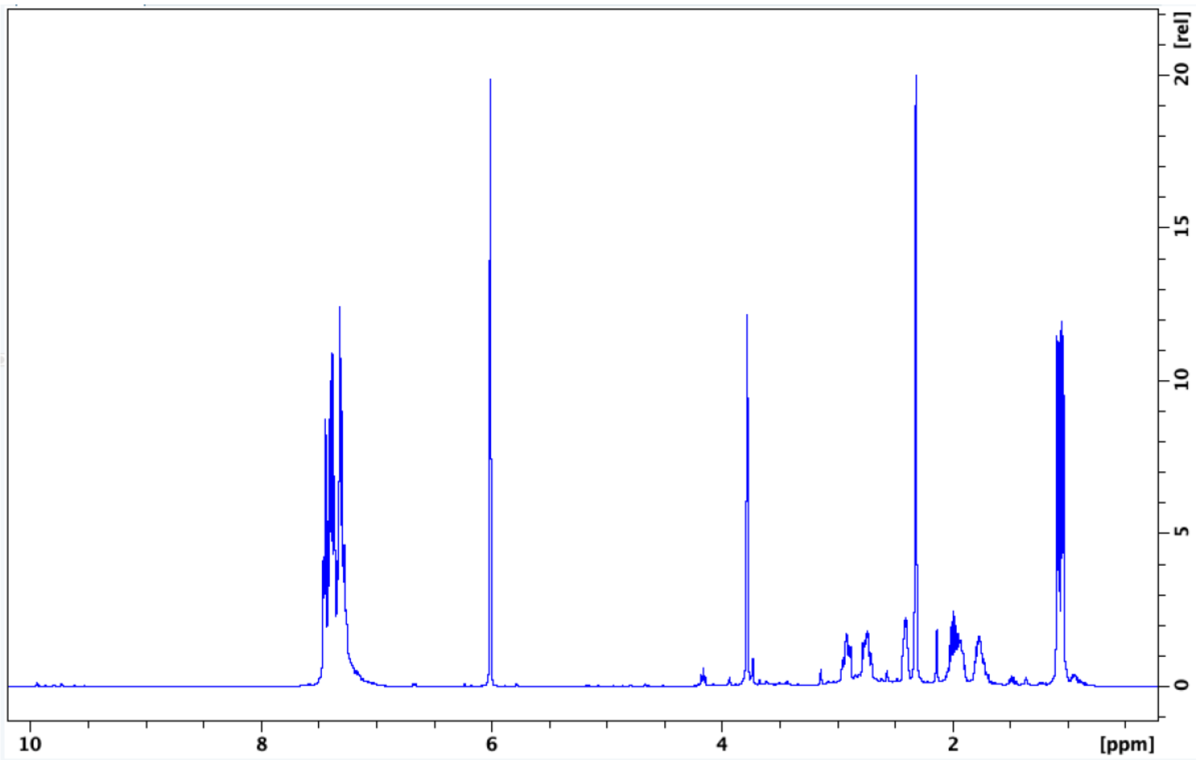

The ^{1}H NMR of the reaction mixture for the 3 layered resins purification trial.

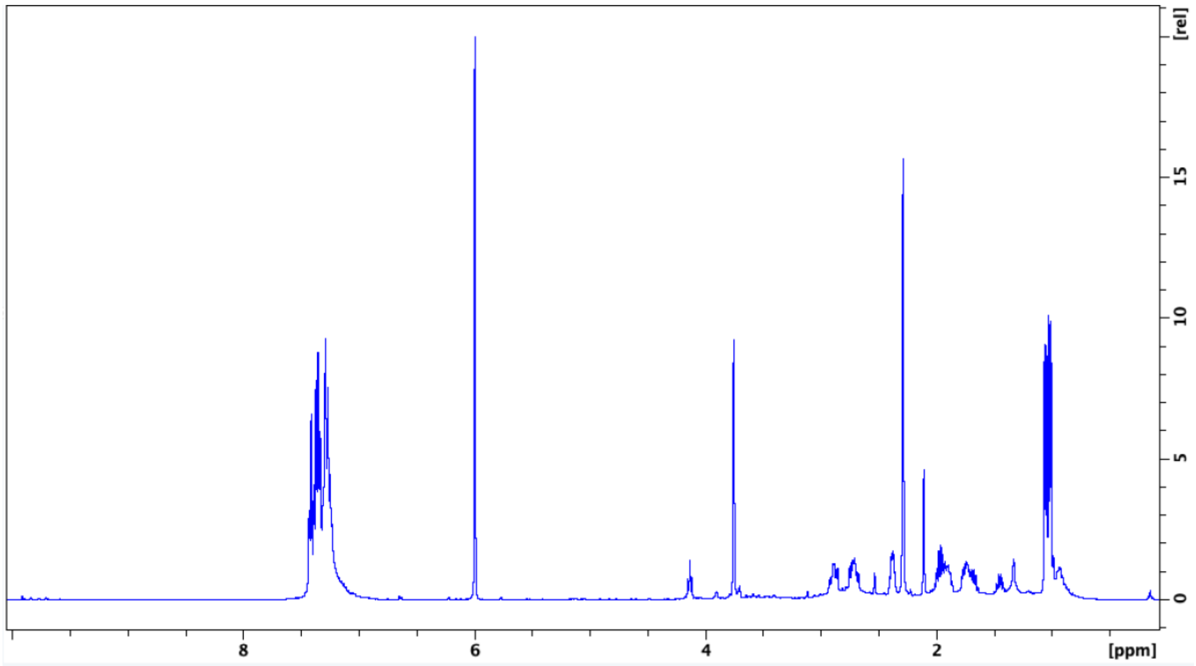

The ^{1}H NMR of the reaction mixture for the 4 resins mixed purification trial.

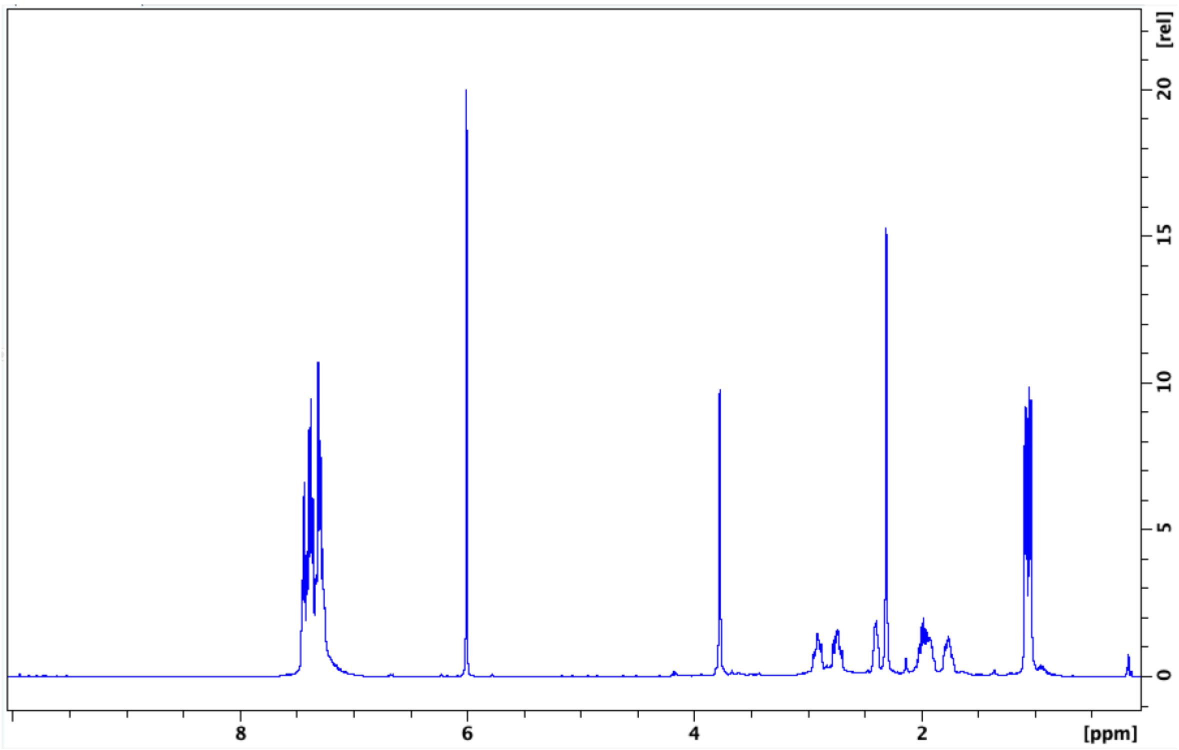

The ^{1}H NMR of the reaction mixture for the 2 step, multi-resin purification trial.

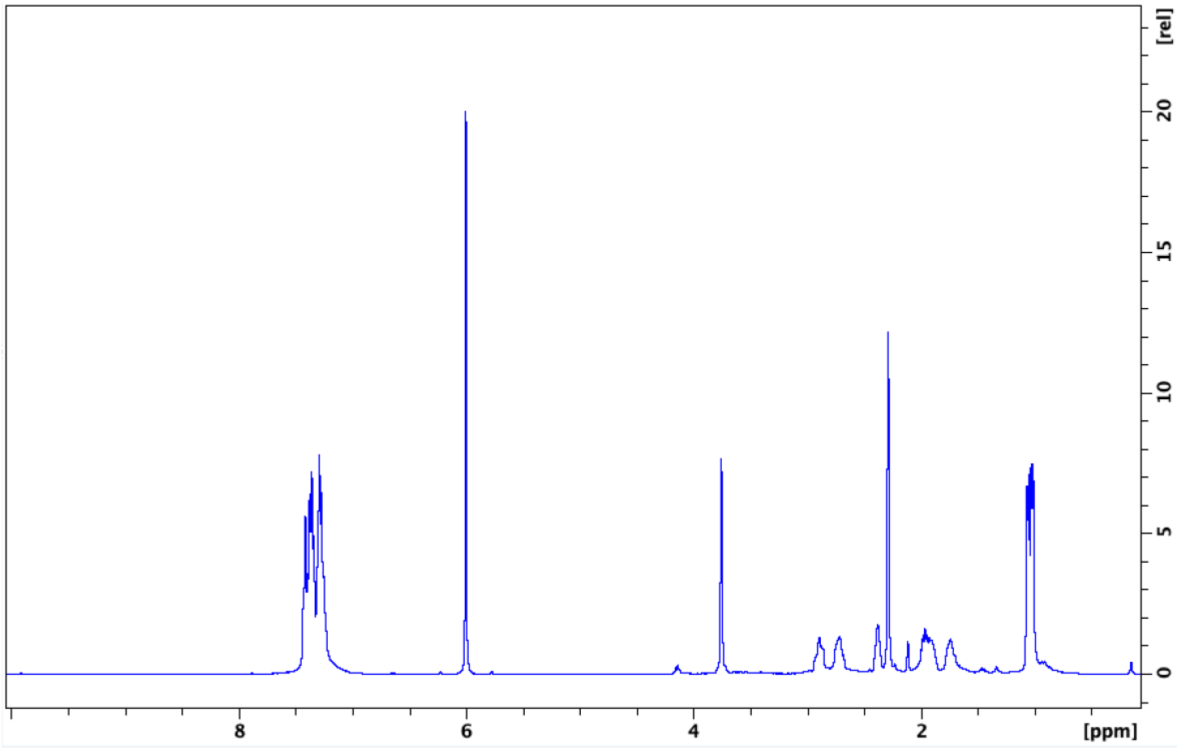

6.8 High-throughput Reaction Screening (primary iodide optimization)

6.8.1 High throughput screening

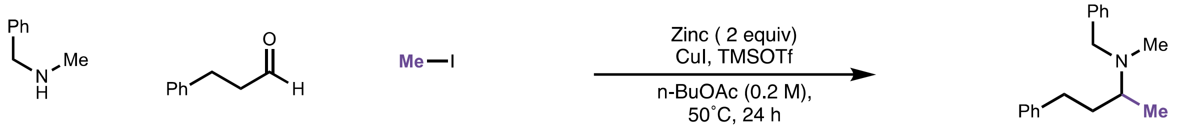

Stock solutions of N-methylbenzylamine (0.8 M), hydrocinnamaldehyde (1.2, 1.6 M), methyl iodide (2.4, 3.2, 4.0, 4.8 M), and TMSOTf (1.2 and 1.4 M) in *n*BuOAc were prepared. To two 384 well plates, Zn dust (20 µmol) was dosed into the central 48 wells (figure 4) via a zinc:*n*BuOAc slurry (12.5 µL, 10 %w/v) using an electronic pipette and the solvent removed via centrifugal evaporation.

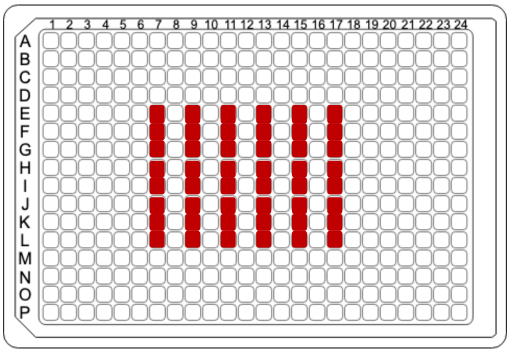

Figure S10: Zinc slurries dosed into central wells

Then, stirrer bars (2 mm x 2 mm) were added to each well. The stock solutions and plate were transferred inside of the purge box and placed under a nitrogen atmosphere (5 x vacuum back refill). Amine (12.5 µL) and aldehyde (12.5 µL) stock solutions were dosed into their corresponding wells and then mixed (5 x 17.5 µL mix volume, 20 µL aspirate volume, integra pipette mix function). Then, alkyl halide (12.5 µL) and TMSOTf (12.5 µL) stock solutions were dosed, in order, to their corresponding wells. The plate was sealed according to General Procedure **D** and taken out of the purge box. The Nanonest was placed onto a hot plate and heated to 50 ˚C and stirred at 1000 rpm (figure 5). Stirring the reaction at 50 ºC, instead of ambident temperature, can ensure uniform mixing for reproducible yields.

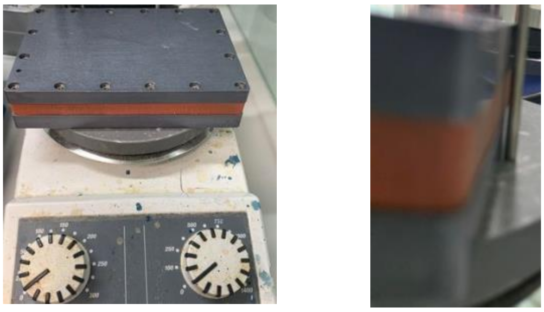

Figure S11: Nanonest enclosure on hotplate (left);
Temperature probe positioned against Nanonest and top of hot plate (right)

After 24 h, the Nanonest was left to cool to room temperature, then opened and the PFA sealed plate was centrifuged for 10s. Then the PFA film was removed, and the reaction mixtures were transferred onto Amberlite IRA-96 resin (100 mg) loaded into a 96 well filter plate. The reaction wells were

washed with two reaction volumes of EtOAc. The filter plate was placed onto of a 96 well receiver plate and centrifuged at 10,000 rpm for 10 s. The resin was washed with EtOAc (300 μL) and centrifuged again. The resin was washed again with EtOAc (50 μL) and centrifuged for 20 s. The solvent was removed using the "high + low boiling point" setting on the Genevac EZ-2 elite with a 40 min 'time to final stage' and a 20 min final stage with a maximum temperature of 40 ˚C. The concentrated reaction mixtures were transferred into the purge box and placed under a nitrogen atmosphere (5 x vacuum back refill). To each well internal standard (1μL, TCE) was dosed and then DMSO-d_6 (200 μL) was added. Each mixture was mixed and then transferred to a 3mm NMR tube (Bruker, Z112272) using a disposable syringe and needle. The NMR tubes were capped with a closed cap (Bruker, Z107163) and placed in a 96 well block (Bruker, Z112272). The NMR samples were placed inside of a Bruker SampleJet Autosampler and a ^{1}H NMR was recorded for each sample (1 scan, 90˚pulse, d1 = 5s, no delay). Yields were determined relative to internal standard based on the methyl group alpha to the amine.

6.8.2 Batch Screening

An oven dried vial (Biotage Microwave Process Vial (10 mL), equipped with gas-tight pierceable caps) was charged with a stirring bar, copper iodide (30 mg, 0.16 mmol, 0.4 equiv) and Zn dust (0.8-1.2 mmol, 2-3 equiv). The vial was sealed, evacuated and backfilled with N_2. To this was added *n*-BuOAc, followed by N-methylbenzylamine (52 μL, 0.4 mmol, 1.0 equiv) and hydrocinnamaldehyde (108 μL, 2 mmol, 2 equiv). The mixture was stirred for 15 min. Iodomethane (100 μL, 1.6 mmol, 4 equiv) was added. The reaction mixture was stirred for 10s, followed by the addition of TMSOTf (108 μL, 0.6 mmol, 1.5 equiv). The reaction mixture was stirred for 24 hours. The reaction mixture was worked up according to general procedure **B** and yields were determined by ^{1}H NMR using 1,1,2,2-tetrachloroethane as internal standard.

Entry	Zn Equiv	Concentration / M	^{1}H NMR yield /%
1	2	0.2	40
2	3	0.2	67
3	2	0.4	83
4	3	0.4	96
5	2	0.8	77

7. CuI assisted Zn-mediated carbonyl alkylative amination

7.1 General procedure E (primary alkyl iodides / benzaldehydes)

An oven dried vial (Biotage Microwave Process Vial (10 mL), equipped with gas-tight pierceable caps) was charged with a stirring bar, Zn dust (1.2 mmol, 3 equiv) and copper iodide (0.16 mmol, 40 mol%) (and amine HCl salt (0.4 mmol, 1 equiv) if used). The vial was sealed, evacuated and backfilled with N_2. To this was added *n*BuOAc (1 mL), followed by the amine (0.4 mmol, 1.0 equiv) (if HCl salt not used), the aldehyde (0.8 mmol, 2.0 equiv). The mixture was stirred for 15 minutes then, whilst stirring, alkyl iodide (1.2 – 1.6 mmol, 3-4 equiv) then TMSOTf (0.3-0.4 mmol, 1.5-2.0 equiv). The reaction mixture was stirred overnight. The crude mixture was transferred into a 50 ml round bottom flask and diluted with dichloromethane (approximately 20 mL). NaOH (approximately 15 mL, 10% aq.) was added. The heterogenous mixture was vigorously stirred for 10-15 min. The aqueous layer was extracted with dichloromethane three times. The combined organic layer was dried over anhydrous $MgSO_4$ or Na_2SO_4. Solvent was evaporated under reduced pressure. If the product is not pure, column chromatography on silica gel is performed.

7.2 Expanded Scope

N-Benzyl-N-methyl-1-phenylbutan-3-amine (10m)

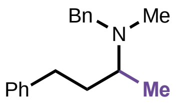

N-Benzyl-N-methyl-1-phenylbutan-3-amine was prepared according to General Procedure **E** using Zn (78 mg, 1.2 mmol, 3 equiv), copper iodide (30 mg, 0.16 mmol, 0.4 equiv), N-methylbenzylamine (52 µL, 0.4 mmol, 1 equiv), hydrocinnamaldehyde (108 µL, 0.8 mmol, 2 equiv), iodomethane (100 µL, 1.26mmol, 4 equiv), and TMSOTf (106 µL, 0.6 mmol, 1.5 equiv) in *n*BuOAc (1 mL). Crude material was purified by an acid-base wash according to general procedure **ABW**, to afford the product as a colourless oil (82 mg, 81%). Data was in line with previous characterisation.[1]

N,N-Dibenzyl-1-phenylbutan-3-amine (11a)

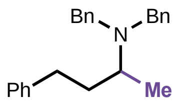

N,N-Dibenzyl-1-phenylbutan-3-amine was prepared according to General Procedure **E** using Zn (78 mg, 1.2 mmol, 3 equiv), copper iodide (30 mg, 0.16 mmol, 0.4 equiv), dibenzylamine (117 µL, 0.4 mmol, 1.0 equiv), hydrocinnamaldehyde (108 µL, 0.8 mmol, 2 equiv), iodomethane (100 µL, 1.26mmol, 4 equiv), and TMSOTf (106 µL, 0.6 mmol, 1.5 equiv) in *n*BuOAc (1 mL). Crude material was purified by column chromatography (4.5% Et_2O in Pet. Ether (with 0.5% NEt_3)) to afford the product as a colourless oil (59 mg, 45%).

^{1}H NMR (500MHz, $CDCl_3$) δ 7.46 (d, J = 7.3 Hz, 4H), 7.37 (t, J = 7.5 Hz, 4H), 7.29 (t, J = 7.5 Hz, 4H), 7.24-7.19 (m, 1H), 7.15 (d, J = 8.2 Hz, 2H), 3.81 (d, J = 13.7, 2H), 3.51 (d, J = 13.8 Hz, 2H), 2.92-2.83 (m, 2H), 2.61 – 2.43 (m, 1H), 2.05-1.95 (m, 1H), 1.67 – 1.55 (m, 1H), 1.12 (d, J = 6.6 Hz, 3H).

^{13}C NMR (126 MHz, $CDCl_3$) δ 143.1, 140.7, 128.8, 128.4, 128.3, 128.2, 126.7, 125.6, 53.4, 52.4, 36.2, 33.4, 13.5.

IR (film, cm^{-1}): 3024,2928,1493, 1452, 1150, 1027, 741, 695, 467.

HRMS: m/z calcd for [M+H]+ 330.2216; found 330.2219.

1-N-Cbz-4-(4-phenylbutan-2-yl)piperazine (11b)

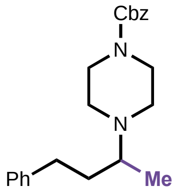

1-N-Cbz-4-(4-phenylbutan-2-yl)piperazine was prepared according to General Procedure **E** using Zn (78 mg, 1.2 mmol, 3 equiv), copper iodide (30 mg, 0.16 mmol, 0.4 equiv), N-Cbz-piperazine hydrochloride (103 mg, 0.4 mmol, 1.0 equiv), hydrocinnamaldehyde (108 µL, 0.8 mmol, 2 equiv), iodomethane (100 µL, 1.26mmol, 4 equiv), and TMSOTf (106 µL, 0.6 mmol, 1.5 equiv) in *n*BuOAc (1 mL). Crude material was purified by column chromatography (15% Et_2O in Pet. Ether), yielding the product as a colourless oil (82 mg, 58%).

^{1}H NMR (500 MHz, $CDCl_3$) δ 7.40 (d, J = 4.5 Hz, 4H), 7.38- 7.28(m, 3H), 7.22 (d, J = 7.4 Hz, 3H), 5.17 (s, 2H), 3.63-3.48 (m, 4H), 2.76-2.60 (m, 3H), 2.67 (brs, 2H), 2.56 (brs, 2H), 1.93-1.83 (m, 1H), 1.65-1.55 (m, 1H), 1.02 (d, J = 6.6 Hz, 3H).

^{13}C NMR (101 MHz, $CDCl_3$) δ 155.3, 142.5, 136.9, 128.5 (d, J = 7.2 Hz), 128.3, 128.0, 127.9, 125.9, 125.7, 67.1, 58.4, 47.9, 44.3, 35.4, 32.9, 13.8.

IR (film, cm^{-1}): 2932, 2859, 1697, 1452, 1427, 1237, 1119, 749, 732, 696.

HRMS: m/z calcd for 353.2224 $[M+H]^+$; found 353.2225.

N-Benzyl-N-methyl-1-phenylhexan-3-amine (10n)

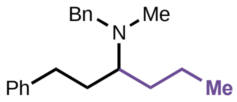

N-Benzyl-N-methyl-1-phenylhexan-3-amine was prepared according to General Procedure **E** using Zn (78 mg, 1.2 mmol, 3 equiv), copper iodide (30 mg, 0.16 mmol, 0.4 equiv), N-methylbenzylamine (52 µL, 0.4 mmol, 1.0 equiv), hydrocinnamaldehyde (108 µL, 0.8 mmol, 2 equiv), 1-iodopropane (156 µL, 1.6 mmol, 4 equiv), and TMSOTf (106 µL, 0.6 mmol, 1.5 equiv) in *n*BuOAc (1 mL). Crude material was purified by an acid-base wash according to general procedure **ABW**, to afford the product as a colourless oil (73 mg, 65%). Data was in line with previous characterisation.[1]

N-benzyl-N-methyl-1,5-diphenylpentan-3-amine (11c)

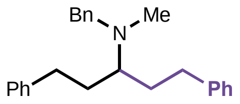

N-benzyl-N-methyl-1,5-diphenylpentan-3-amine was prepared according to General Procedure **E** using Zn (78 mg, 1.2 mmol, 3 equiv), copper iodide (30 mg, 0.16 mmol, 0.4 equiv), N-methylbenzylamine hydrochloride (63 mg , 0.4 mmol, 1.0 equiv), hydrocinnamaldehyde (108 µL, 0.8 mmol, 2 equiv), (2-iodoethyl)benzene (174 µL, 1.2 mmol, 3 equiv) and TMSOTf (36 µL, 0.2 mmol, 0.5 equiv) in *n*BuOAc (1 mL). Crude material was purified by column chromatography (0-15% Et_2O in Pet. Ether (3.0% NEt_3)) to afford a colourless oil (59 mg, 43%). Data was in line with previous characterisation.[1]

N-benzyl-1-fluoro-N-methyl-5-phenylpentan-3-amine (11d)

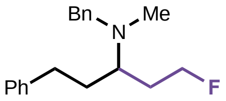

N-benzyl-1-fluoro-N-methyl-5-phenylpentan-3-amine was prepared according to General Procedure **E** using Zn (78 mg, 1.2 mmol, 3 equiv), copper iodide (30 mg, 0.16 mmol, 0.4 equiv), N-methylbenzylamine hydrochloride (63 mg , 0.4 mmol, 1.0 equiv), hydrocinnamaldehyde (108 µL, 0.8 mmol, 2 equiv), 1-fluoro-2-iodoethane (105 µL, 1.2 mmol, 3 equiv) and TMSOTf (36 µL, 0.2 mmol, 0.5 equiv) in *n*BuOAc (1 mL). Crude material was purified by column chromatography (0-20% EtOAc in Pet. ether) to afford the product as a colourless oil (68 mg, 60%).

^{1}H NMR (500 MHz, $CDCl_3$): 7.32 – 7.27 (m, 6H), 7.25 – 7.17 (m, 4H), 4.71 – 4.44 (m, 2H), 3.57 (d, J = 2.2 Hz, 2H), 2.80 – 2.63 (m, 3H), 2.16 (s, 3H), 2.02 – 1.87 (m, 2H), 1.84 – 1.72 (m, 1H), 1.66 – 1.57 (m, 1H).

^{13}C NMR (126 MHz, $CDCl_3$): 142.5, 140.1, 128.6, 128.41, 128.39, 128.2, 126.8, 125.8, 82.6 (d, J = 163.6 Hz), 58.5 (d, J = 5.4 Hz), 58.0, 36.1, 33.4, 31.8, 30.8 (d, J = 19.1 Hz).

^{19}F NMR (376 MHz, $CDCl_3$): δ -219.25.

IR (film, cm^{-1}): 2929, 2854, 2255, 1983, 1495, 1370, 837.

HRMS: m/z calcd for $C_{19}H_{24}NF$ $[M+H]^+$ 286.1966; found 286.1966.

N-Benzyl-*N*-methyl-1-phenylhexan-3-amine (11e)

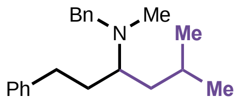

N-Benzyl-N-methyl-1-phenylhexan-3-amine was prepared according to General Procedure **E** using Zn (78 mg, 1.2 mmol, 3 equiv), copper iodide (30 mg, 0.16 mmol, 0.4 equiv), N-methylbenzylamine hydrochloride (63 mg, 0.4 mmol, 1.0 equiv), hydrocinnamaldehyde (108 µL, 0.8 mmol, 2 equiv), 1-Iodo-2-methylpropane (138 µL, 1.2 mmol, 3 equiv), and TMSOTf (36 µL, 0.2 mmol, 0.5 equiv) in *n*BuOAc (1 mL). Crude material was purified by column chromatography (0-10% (10% Et_2O:90% Pet. Ether: NEt_3 3%) in Pet. Ether) afford the product as a colourless oil (47 mg, 40%).

^{1}H NMR (500 MHz, $CDCl_3$) δ 7.39-7.29 (m, 6H), 7.29-7.20 (m, 4H), 3.61 (d, J = 13.7 Hz, 1 H), 3.57 (d, J = 13.7, 2H), 2.82-2.74 (m, 1H), 2.73-2.67 (m, 1H), 2.67-2.60 (m, 1H), 2.18 (s, 3H), 1.92-1.83 (m, 1H), 1.81- 1.70 (m, 1H), 1.65-1.56 (m, 1H), 1.53-1.45 (m, 1H), 1.23-1.15 (m, 1H), 0.89 (dd, J = 4.6, 6.8 Hz, 6H).

^{13}C NMR (101 MHz, $CDCl_3$) δ 143.1, 140.8, 128.7, 128.5, 128.3, 128.1, 126.6, 125.6, 59.8, 57.8, 38.8, 36.3, 33.5, 32.2, 25.1, 23.0, 22.9.

IR (film, cm^{-1}): 2956, 2928, 1456, 731, 698.

HRMS: m/z calcd for $C_{21}H_{29}N$ $[M+H]^+$ 296.2373; found 296.2378.

N-benzyl-N-methyl-1-phenylpentan-3-amine-4,4,5,5,5-*d*$_5$ (11f)

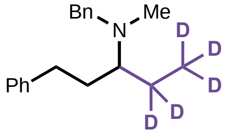

N-benzyl-N-methyl-1-phenylpentan-3-amine-4,4,5,5,5-*d*$_5$ was prepared according to General Procedure **G** using Zn (78 mg, 1.2 mmol, 3 equiv), copper iodide (30 mg, 0.16 mmol, 0.4 equiv), N-methylbenzylamine hydrochloride (63 mg , 0.4 mmol, 1.0 equiv), hydrocinnamaldehyde (108 µL, 0.8 mmol, 2 equiv), iodoethane-*d*$_5$ (96 µL, 1.2 mmol, 3 equiv) and TMSOTf (36 µL, 0.2 mmol, 0.5 equiv) in *n*BuOAc (1 mL). Crude material was purified by column chromatography (0-10% EtOAc in Pet. Ether) afford the product as a colourless oil (61 mg, 72%).

^{1}H NMR (500 MHz, $CDCl_3$) δ 7.41 – 7.26 (m, 6H), 7.20 (sept, J = 7.2 Hz, 4H), 3.61 (d, J = 13.5 Hz, 1H), 3.55 (d, J = 13.5 Hz, 1H), 2.77 (ddd, J = 15.0, 10.3, 5.4 Hz, 1H), 2.65 (ddd, J = 13.7, 10.2, 6.2 Hz, 1H), 2.42 (t, J = 6.8 Hz, 1H), 2.16 (s, 3H), 1.85 – 1.73 (m, 1H), 1.63 (ddt, J = 13.1, 10.2, 6.1 Hz, 1H);

^{13}C NMR (126 MHz, $CDCl_3$) δ 143.2, 140.7, 128.7, 128.5, 128.3, 128.2, 126.7, 125.6, 64.0, 59.5, 57.9, 40.5, 36.4, 33.6, 32.0.

^{2}H NMR (107 MHz, $CDCl_3$) δ 1.58 (s, 1H), 1.30 (s, 1H), 0.88 (s, 3H).

IR (film, cm^{-1}): 3024, 2924, 2853, 2785, 1601, 1493, 1452, 1026, 735, 679

HRMS: m/z calculated for $C_{19}H_{32}D_5N$ $[M+H]^+$ 273.2379; found 273.2376.

N-benzyl-7-chloro-N-methyl-1-phenylheptan-3-amine (11g)

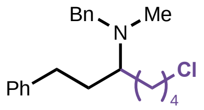

N-benzyl-7-chloro-N-methyl-1-phenylheptan-3-amine was prepared according to General Procedure **E** using Zn (78 mg, 1.2 mmol, 3 equiv), copper iodide (30 mg, 0.16 mmol, 0.4 equiv), N-methylbenzylamine (52 µL, 0.4 mmol, 1.0 equiv), hydrocinnamaldehyde (108 µL, 0.8 mmol, 2 equiv), 1-chloro-4-iodobutane (118 µL, 1.2 mmol, 3 equiv) and TMSOTf (108 µL, 0.6 mmol, 1.5 equiv) in *n*BuOAc (1 mL) with a modification. The reaction mixture was quenched with saturated Na_2CO_3 (approx. 20 ml, aq). Crude material mixture was purified by column chromatography (0-40% (10% Et_2O:90% Pet. Ether: NEt_3 3%) in Pet. Ether), to afford a colourless oil (102 mg, 78%).

NOTE: This product cyclises to the quaternary ammonium in under 1 hour in $CDCl_3$ and continues to convert in solution. Compound should be stored as HCl salt before further use.

^{1}H NMR (400 MHz, $CDCl_3$) δ 7.40 – 7.20 (m, 10H), 3.61 (s, 2H), 3.58 (t, J = 6.7 Hz, 2H), 2.83 – 2.66 (m, 2H), 2.56 (quint, J = 6.6 Hz, 1H), 2.21 (s, 3H), 1.96-1.85 (m, 1H), 1.79 (quint, J = 7.0 Hz, 2H), 1.70- 1.59 (m, 2H), 1.58-1.48 (m, 2H), 1.44-1.33 (m, 1H).

^{13}C NMR (101 MHz, $CDCl_3$) δ 142.9, 140.5, 128.6, 128.42, 128.36, 128.2, 126.7, 125.7, 61.8, 57.9, 45.1, 36.3, 33.5, 32.8, 32.0, 28.8, 24.5.

IR ($CDCl_3$ solution, cm^{-1}):3097, 3040, 2938, 1609, 1500, 1459, 1033, 760, 704, 542.

HRMS: m/z calcd for $C_{21}H_{28}NCl$ $[M+H]^+$ 330.1983; found 330.1987.

1-(1-phenylpentan-2-yl)pyrrolidine (Prolitane) (11h)

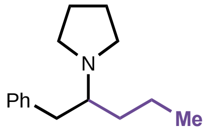

1-(1-phenylpentan-2-yl)pyrrolidine (Prolitane) was prepared according to General Procedure **E** using Zn (392 mg, 6 mmol, 3 equiv), copper iodide (152 mg, 0.8 mmol, 0.4 equiv), pyrrolidine (164 µL, 2 mmol, 1.0 equiv), phenylacetaldehyde (481 mg, 4.0 mmol, 2 equiv), 1-iodopropane (582 µL, 6 mmol, 3 equiv) and TMSOTf (543 µL, 3 mmol, 1.5 equiv) in *n*BuOAc (5 mL). The crude material was filtered through an SCX-column (eluting with 5N NH_3 in MeOH) then purified by reverse phase column chromatography (20-100% MeCN in H_2O) to afford the product as a colourless oil (395 mg, 93%). Characterisation matched previous data.[5]

^{1}H NMR (500 MHz, $CDCl_3$) δ 7.33 – 7.27 (m, 2H), 7.25 – 7.19 (m, 3H), 3.01 (q, J = 8.5 Hz, 1H), 2.77 – 2.68 (m, 2H), 2.68 – 2.54 (m, 4H), 1.88 – 1.72 (m, 4H), 1.47 – 1.38 (m, 3H), 1.38 – 1.26 (m, 1H), 0.85 (t, J = 7.0 Hz, 2H).

^{13}C NMR (126 MHz, $CDCl_3$) δ 140.9, 129.3, 128.2, 125.7, 64.4, 50.6, 37.6, 33.8, 23.6, 18.5, 14.4.

IR (film, cm^{-1}): 2955, 2929, 2870, 1493, 1453, 1376, 1352, 1131, 1030, 735, 698.

HRMS: m/z calculated for $C_{30}H_{32}N$ $[M+H]^+$ 218.1908; found 218.1907.

N-Benzyl-N-methyl-1-phenyl-2methylpropan-1-amine (12a)

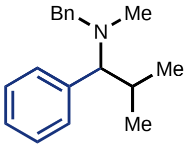

N-Benzyl-N-methyl-1-phenyl-2-methylpropan-1-amine was prepared according to General Procedure **E** using Zn (78 mg, 1.2 mmol, 3 equiv), copper iodide (30 mg, 0.16 mmol, 0.4 equiv), N-methylbenzylamine (52 µL, 0.4 mmol, 1.0 equiv), benzaldehyde (82 µL, 0.8 mmol, 2 equiv), 2-iodopropane (160 mL, 1.6 mmol, 4 equiv) and TMSOTf (108 µL, 0.6 mmol, 1.5 equiv) in *n*BuOAc (1 mL). Crude material was purified by acid-base wash according to General Procedure **ABW** to afford the product as a colourless oil (92 mg, 91%).

^{1}H NMR (400 MHz, $CDCl_3$) δ 7.44-7.39 (m, 4H), 7.39-7.29 (m, 4H), 7.25-7.21 (m, 2H), 3.57 (d, J = 13.5 Hz, 1H), 3.30 (d, J = 13.5 Hz, 1H), 3.22 (d, J = 9.6 Hz, 1H), 2.40 (m, 1H), 2.12 (s, 3H), 1.20 (d, J = 6.5 Hz, 3H), 0.79 (d, J = 6.5 Hz, 3H).

^{13}C NMR (121 MHz, $CDCl_3$) δ 140.4, 137.5, 129.4, 128.7, 128.2, 127.7, 126.8, 126.6, 74.7, 58.6, 37.4, 28.6, 20.8, 20.0.

IR (film, cm^{-1}): 2954, 2789,1493, 1451, 1020, 735, 697.

HRMS: m/z calcd for [M+H]+ 254.1903, found 254.1903.

methyl 4-(1-(benzyl(methyl)amino)-2-methylpropyl)benzoate (12b)

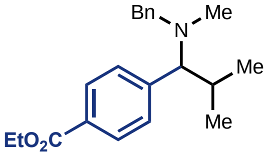

methyl 4-(1-(benzyl(methyl)amino)-2-methylpropyl)benzoate was prepared according to General Procedure **E** using Zn (78 mg, 1.2 mmol, 3 equiv), copper iodide (30 mg, 0.16 mmol, 0.4 equiv), N-methylbenzylamine (52 µL, 0.4 mmol, 1.0 equiv), methyl 4-formylbenzoate (131 mg, 0.8 mmol, 2 equiv), 2-iodopropane (120 mL, 1.2 mmol, 3 equiv) and TMSOTf (108 µL, 0.6 mmol, 1.5 equiv) in nBuOAc (1 mL). Crude material was purified by column chromatography (0-10% (90% Pet. Ether: 10% Et_2O: 3% NEt_3) in Pet. Ether) to give the product as a colourless oil (83%, 67%)

^{1}H NMR (500 MHz, $CDCl_3$) δ 8.07 (d, J = 8.5 Hz, 1H), 7.38- 7.24 (m, 7H), 3.96 (s, 3H), 3.51 (d, J = 13.7 Hz, 1H), 3.28-3.22 (m, 2H), 2.43 -2.33 (m, 1H), 2.09 (s, 3H), 1.16 (d, J = 6.5 Hz, 3H), 0.75 (d, J = 6.5 Hz, 3H)

^{13}C NMR (126 MHz, $CDCl_3$) δ 167.2, 143.1, 139.9, 129.3, 129.0, 128.7, 128.6, 128.2, 126.8, 74.3, 58.7, 52.1, 37.4, 28.5, 20.6, 19.7.

IR (film, cm^{-1}): 2952, 1720, 1435, 1276, 1103, 1018, 713, 698.

HRMS: m/z calcd for $[M+H]^+$ 312.1958, found 312.19610

N-benzyl-1-(6-bromobenzo[d][1,3]dioxol-5-yl)-N,2-dimethylpropan-1-amine (12c)

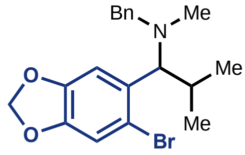

N-benzyl-1-(6-bromobenzo[d][1,3]dioxol-5-yl)-N,2-dimethylpropan-1-amine was prepared according to General Procedure **E** using Zn (78 mg, 1.2 mmol, 3 equiv), copper iodide (30 mg, 0.16 mmol, 0.4 equiv), N-methylbenzylamine (52 μL, 0.4 mmol, 1.0 equiv), 6-bromo-benzo[1,3]dioxole-5-carbaldehyde (183 mg, 0.8 mmol, 2 equiv), 2-iodopropane (120 μL, 1.2 mmol, 3 equiv) and TMSOTf (108 μL, 0.6 mmol, 1.5 equiv) in *n*BuOAc (1 mL). Crude material was purified by column chromatography (0-10% (90% Pet. Ether: 10% Et_2O: 3% NEt_3) in Pet. Ether) to give the product as a colourless oil (50 mg, 33%)

^{1}H NMR (500 MHz, $CDCl_3$) δ 7.37 - 7.22 (m, 5H), 6.89 (s, 1H), 7.09 (s, 1H), 3.95 (d, J = 9.2, 1H), 3.55 (d, J = 18.9 Hz, 1H), 3.50 (d, J = 18.9, 1H), 2.29 - 2.19 (m, 1H), 2.10 (s, 3H), 1.19 (d, J = 6.4 Hz, 3H), 0.81 (d, J = 6.2 Hz, 3H)

^{13}C NMR (126 MHz, $CDCl_3$) δ 147.1, 146.9, 140.4, 131.6, 128.5, 128.2, 126.6, 116.9, 112.6, 108.6, 101.6, 72.2, 58.0, 37.4, 30.1, 20.5, 19.1.

IR (film, cm^{-1}): 2957, 2787, 1473, 1223, 1037, 734, 698.

HRMS: m/z calcd for $[M+H]^+$ 376.0907, found 376.0908.

N-benzyl-N,2-dimethyl-1-(1-methyl-1H-pyrazol-4-yl)propan-1-amine (12d)

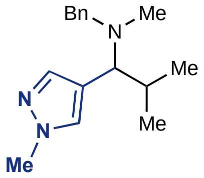

N-benzyl-N,2-dimethyl-1-(1-methyl-1H-pyrazol-4-yl)propan-1-amine was prepared according to General Procedure **E** with modifications. In 4 mL vial capped with a septa, Zn (78 mg, 1.2 mmol, 3 equiv), 2-iodopropane (120 μL, 1.2 mmol, 3 equiv) and TMSOTf (54 μL, 0.3 mmol, 0.75 equiv) in *n*BuOAc (0.5 mL) were stirred for 1 hour. At the same time, copper iodide (30 mg, 0.16 mmol, 0.4 equiv), N-methylbenzylamine (52 μL, 0.4 mmol, 1.0 equiv), 1-methyl-1H-pyrazole-4-carbaldehyde (77 mg, 0.7 mmol, 1.75 equiv), and TMSOTf (54 μL, 0.3 mmol, 0.75 equiv) in *n*BuOAc (0.5 mL) were stirred in a 10 mL microwave vial equipped with a septa cap. After 1 hour, the zinc and alkyl halide mixture was syringed into the other vial with stirring. The rest of the reaction was carried our using General Procedure **E**. Crude material was purified by column chromatography (0-10% (90% Pet. Ether: 10% Et_2O: 3% NEt_3) in Pet. Ether) to give the product as a colourless oil (82 mg, 80%)

^{1}H NMR (500 MHz, $CDCl_3$) δ 7.34 (p, J = 7.21, 5H), 7.27 - 7.22 (m, 1H), 7.17 (s, 1H), 3.94 (s, 3H), 3.51 (d, J = 13.4 Hz, 1H), 3.19 (d, J = 13.9 Hz, 1H), 3.14 (d, J = 10.5 Hz, 1H), 2.15 - 2.07 (m, 1H), 2.03 (s, 3H), 1.15 (d, J = 6.5 Hz, 3H), 0.77 (d, J = 6.7 Hz, 3H)

^{13}C NMR (126 MHz, $CDCl_3$) δ 140.2, 139.6, 129.2, 128.7, 128.2, 126.6, 117.2, 65.4, 58.5, 38.9, 37.1, 30.1, 20.8, 20.6

IR (film, cm^{-1}): 2952, 2789, 1452, 1024, 1014, 985, 732, 697

HRMS: m/z calcd for $[M+H]^+$ 258.1965, found 258.1967.

N-benzyl-1-(3,5-dimethylisoxazol-4-yl)-N,2-dimethylpropan-1-amine (12e)

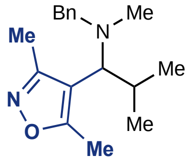

N-benzyl-1-(3,5-dimethylisoxazol-4-yl)-N,2-dimethylpropan-1-amine was prepared according to General Procedure **E** with modifications. In 4 mL vial capped with a septa, Zn (78 mg, 1.2 mmol, 3 equiv), 2-iodopropane (120 µL, 1.2 mmol, 3 equiv) and TMSOTf (54 µL, 0.3 mmol, 0.75 equiv) in *n*BuOAc (0.5 mL) were stirred for 1 hour. At the same time, copper iodide (30 mg, 0.16 mmol, 0.4 equiv), N-methylbenzylamine (52 µL, 0.4 mmol, 1.0 equiv), 3,5-dimethyl-4-isoxazolecarbaldehyde (88 mg, 0.7 mmol, 1.75 equiv), and TMSOTf (54 µL, 0.3 mmol, 0.75 equiv) in *n*BuOAc (0.5 mL) were stirred in a 10 mL microwave vial equipped with a septa cap. After 1 hour, the zinc and alkyl halide mixture was syringed into the other vial with stirring. The rest of the reaction was carried our using General Procedure **E**. Crude material was purified by column chromatography (0-10% (90% Pet. Ether: 10% Et_2O: 3% NEt_3) in Pet. Ether) to give the product as a colourless oil (61 mg, 56%)

^{1}H NMR (400 MHz, $CDCl_3$) δ 7.36 - 7.30 (m, 1H), 7.30 - 7.23 (m, 1H), 3.54 (d, J = 13.8 Hz, 1H), 3.37 (d, J = 13.3 Hz, 1H), 3.15 (d, J = 9.1 Hz, 1H), 2.44 (s, 3H), 2.42 - 2.34 (m, 1H), 2.23 (s, 3H), 2.06 (s, 3H), 1.14 (d, J = 6.5 Hz, 3H), 0.81 (d, J = 6.6 Hz, 3H)

^{13}C NMR (101 MHz, $CDCl_3$) δ 166.7, 160.2, 139.6, 128.5, 128.3, 126.9, 110.6, 65.9, 58.9, 38.2, 28.9, 21.0, 19.4, 12.8, 12.0

IR (film, cm^{-1}): 2958, 2785, 1452, 1416, 1024, 737, 698

HRMS: m/z calcd for $[M+H]^+$ 273.1961, found 273.1964.

N-benzyl-1-(6-bromopyridin-2-yl)-N,2-dimethylpropan-1-amine (12f)

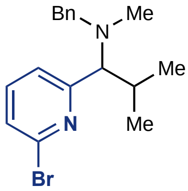

N-benzyl-1-(6-bromopyridin-2-yl)-N,2-dimethylpropan-1-amine was prepared according to General Procedure **E** with modifications. In 4 mL vial capped with a septa, Zn (78 mg, 1.2 mmol, 3 equiv), 2-iodopropane (120 µL, 1.2 mmol, 3 equiv) and TMSOTf (54 µL, 0.3 mmol, 0.75 equiv) in *n*BuOAc (0.5 mL) were stirred for 1 hour. At the same time, copper iodide (30 mg, 0.16 mmol, 0.4 equiv), N-methylbenzylamine (52 µL, 0.4 mmol, 1.0 equiv), 6-bromopyridine-2-carboxaldehyde (149 mg, 0.8 mmol, 1.75 equiv), and TMSOTf (54 µL, 0.3 mmol, 0.75 equiv) in *n*BuOAc (0.5 mL) were stirred in a 10 mL microwave vial equipped with a septa cap. After 1 hour, the zinc and alkyl halide mixture was syringed into the other vial with stirring. The rest of the reaction was carried our using General Procedure **E**. Crude material was purified by column chromatography (0-10% (90% Pet. Ether: 10% Et_2O: 3% NEt_3) in Pet. Ether) to give the product as a colourless oil (55 mg, 41%)

^{1}H NMR (500 MHz, $CDCl_3$) δ 7.53 (t, J = 7.7 Hz, 1H), 7.39 (dd, J = 7.9, 0.9 Hz, 1H), 7.37 - 7.31 (m, 4H), 7.25 (tt, J = 7.0, 1.6 Hz, 1H), 7.10 (dd, J = 7.5, 0.8 Hz, 1H), 3.61 (d, J = 13.7 Hz, 1H), 3.37 - 3.30 (m, 2H), 2.53 - 2.43 (m, 1H), 2.13 (s, 3H), 1.17 (d, J = 6.7 Hz, 3H), 0.73 (d, J = 6.6 Hz, 3H)

^{13}C NMR (126 MHz, $CDCl_3$) δ 160.5, 141.4, 140.1, 137.7, 128.6, 126.7, 126.0, 122.5, 74.9, 58.4, 37.5, 28.2, 20.2, 20.0

IR (film, cm^{-1}): 2956, 2869, 2793, 1575, 1551, 1429, 1108, 1024, 791, 735, 696

HRMS: m/z calcd for $[M+H]^+$ 333.0961, found 333.0967.

N-benzyl-1-(3-(4-bromophenyl)isoxazol-5-yl)-N,2-dimethylpropan-1-amine (12g)

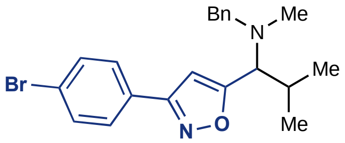

N-benzyl-1-(3-(4-bromophenyl)isoxazol-5-yl)-N,2-dimethylpropan-1-amine was prepared according to General Procedure **E** with modifications. In 4 mL vial capped with a septa, Zn (78 mg, 1.2 mmol, 3 equiv), 2-iodopropane (120 µL, 1.2 mmol, 3 equiv) and TMSOTf (54 µL, 0.3 mmol, 0.75 equiv) in *n*BuOAc (0.5 mL) were stirred for 1 hour. At the same time, copper iodide (30 mg, 0.16 mmol, 0.4 equiv), N-methylbenzylamine (52 µL, 0.4 mmol, 1.0 equiv), 3-(4-bromophenyl)isoxazole-5-carboxaldehyde (202 mg, 0.8 mmol, 1.75 equiv), and TMSOTf (54 µL, 0.3 mmol, 0.75 equiv) in *n*BuOAc (0.5 mL) were stirred in a 10 mL microwave vial equipped with a septa cap. After 1 hour, the zinc and alkyl halide mixture was syringed into the other vial with stirring. The rest of the reaction was carried our using General Procedure **E**. Crude material was purified by column chromatography (0-10% (90% Pet. Ether: 10% Et_2O: 3% NEt_3) in Pet. Ether) to give the product as a colourless oil (125 mg, 78%)

^{1}H NMR (500 MHz, $CDCl_3$) δ 7.77 - 7.74 (m, 2H), 7.65 - 7.62 (m, 2H), 7.41 - 7.33 (m, 4H), 7.31 – 7.26 (m, 1H), 6.36 (s, 1H), 3.68 (d, J = 13.6 Hz, 1H), 3.47 (d, J = 10.7 Hz, 1H), 3.40 (d, J = 13.5 Hz, 1H), 2.40 - 2.29 (m, 1H), 2.20 (s, 3H), 1.21 (d, J = 6.5 Hz, 3H), 0.85 (d, J = 6.5 Hz, 3H)

^{13}C NMR (126 MHz, $CDCl_3$) δ 171.9, 160.9, 139.3, 132.2, 128.4 - 128.3 (m) 127.0, 124.2, 100.6, 66.3, 58.9, 37.5, 28.8, 20.4, 20.1

IR (film, cm^{-1}): 2959, 1426, 1072, 1024, 1011, 833, 736, 697

HRMS: m/z calcd for $[M+H]^+$ 399.1067, found 399.1050.

(4-(2-methyl-1-phenylpropyl)piperazin-1-yl)(naphthalen-2-yl)methanone (14a) 0.4 mmol reaction

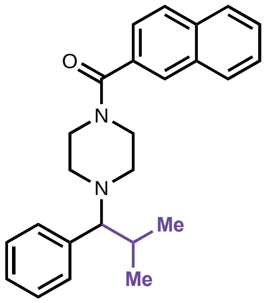

(4-(2-methyl-1-phenylpropyl)piperazin-1-yl)(naphthalen-2-yl)methanone was prepared according to General Procedure **E** using Zn (78 mg, 1.2 mmol, 3 equiv), copper iodide (30 mg, 0.16 mmol, 0.16 equiv), naphthalen-2-yl(piperazin-1-yl)methanone (prepared according to *Waldman et al.)*[6] (96 mg, 0.4 mmol, 1.0 equiv), benzaldehyde (82 µL, 0.8 mmol, 2 equiv), 2-iodopropane (160 µL, 1.6 mmol, 4 equiv) and TMSOTf (108 µL, 0.6 mmol, 1.5 equiv) in *n*BuOAc (1 mL). Crude material was purified by column chromatography (0-10% EtOAc in Pet. Ether) to give the product as a colourless oil (102 mg, 68%).

^{1}H NMR (500 MHz, $CDCl_3$) δ 7.88-7.83 (m, 4H), 7.56-7.51 (m, 2H), 7.43 (dd, J = 8.4, 1.4 Hz, 1H), 7.37-7.38-7.32 (m, 2H), 7.32-7.28 (m, 1H), 7.16-7.12 (m, 1H), 3.83 (*b*d, 2H), 3.47 (*b*s, 2H), 3.08 (d, J = 8.93, 1H), 2.64- 2.21 (m, 5H), 1.04 (d, J = 6.9 Hz, 3H), 0.76 (d, J = 6.5 Hz, 3H).

^{13}C NMR (126 MHz, $CDCl_3$) δ 170.1, 137.2, 133.6, 133.3, 132.7, 129.2, 128.24, 128.2, 127.82, 127.77, 127.1, 127.0, 126.9, 126.6, 124.4, 76.2, 49.3 (t, J = 123.8 Hz), 42.6, 27.8, 20.6, 19.1.

IR (film, cm^{-1}): 2916, 2828, 1621, 1422, 1236, 1006, 998, 756, 712.

HRMS: m/z calcd for $C_{25}H_{28}N_2O$ $[M+H]^+$ 373.2274, found 373.2274.

Scaleup: (4-(2-methyl-1-phenylpropyl)piperazin-1-yl)(naphthalen-2-yl)methanone (14a) 4 mmol reaction

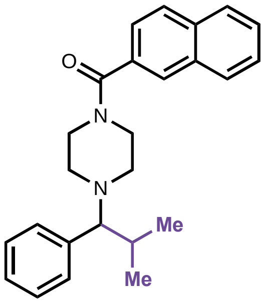

(4-(2-methyl-1-phenylpropyl)piperazin-1-yl)(naphthalen-2-yl)methanone was prepared according to General Procedure **E** using Zn (785 mg, 12 mmol, 3 equiv), copper iodide (305 mg, 1.6 mmol, 0.4 equiv), naphthalen-2-yl(piperazin-1-yl)methanone (prepared according to *Waldman et al.)*[6] (961 mg, 4 mmol, 1.0 equiv), benzaldehyde (816 µL, 8 mmol, 2 equiv), 2-iodopropane (1.6 mL, 16 mmol, 4 equiv) and TMSOTf (1.1 mL, 6 mmol, 1.5 equiv) in *n*BuOAc (10 mL) with modifications. The reaction was performed in 100 mL schlenck flask which was sealed off from nitrogen after vacuum backrefills. The TMSOTf was added dropwise over 2 minutes, avoiding the reaction mixture refluxing. The crude material was purified by column chromatography (0-10% EtOAc in Pet. Ether) to give the product as a colourless oil which solidified upon standing to a white solid (1.2 g, 80%). Data inline with 0.4 mmol reaction.

7.3 Tertiary Alkyl Halide Optimization

7.3.1 Standard reaction condition controls.

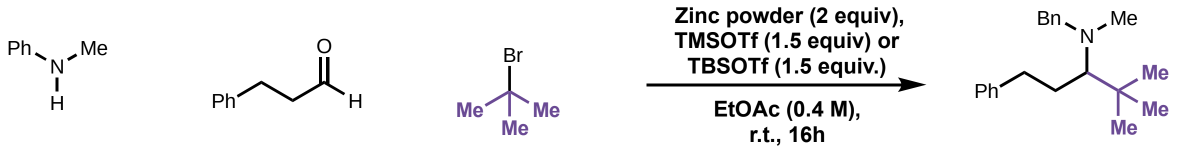

The synthesis of N-benzyl-N,4,4-trimethyl-1-phenylpentan-3-amine was attempted according to General Procedure **B** using Zn (52 mg, 0. 8 mmol, 2 equiv), N-methylbenzylamin*e* (52 µL, 0.4 mmol, 1 equiv), hydrocinnamaldehyde (106 µL, 0.8 mmol, 2 equiv), tertbutyliodide (143 µL, 1.2 mmol, 3 equiv) and TMSOTf (106 µL, 0.6 mmol, 1.5 equiv) or TBSOTf (137 µL, 0.6 mmol, 1.5 equiv) in EtOAc (1 mL). Yield of the product was determined by ^{1}H NMR using 1,1,2,2-tetrachloroethane as internal standard.

Exp	Lewis Acid	Assay Yield / %
**A**	TMSOTf	30
**B**	TBSOTf	34

7.3.2 CuI-assisted reaction condition controls.

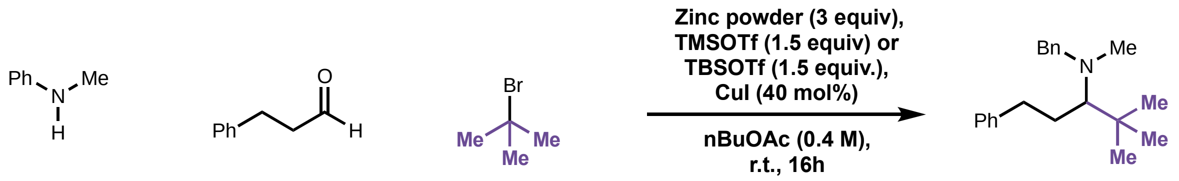

The synthesis of N-benzyl-N,4,4-trimethyl-1-phenylpentan-3-amine was attempted according to General Procedure **E** using Zn (78 mg, 1.2 mmol, 3 equiv), copper iodide (30 mg, 0.16 mmol, 0.4 equiv), N-methylbenzylamine (52 µL, 0.4 mmol, 1 equiv), hydrocinnamaldehyde (108 µL, 0.8 mmol, 2 equiv), tert-butyl bromide (180 µL, 1.26mmol, 4 equiv), and TMSOTf (106 µL, 0.6 mmol, 1.5 equiv) or TBSOTf (137 µL, 0.6 mmol, 1.5 equiv) in *n*BuOAc (1 mL). Yield of the product was determined by ^{1}H NMR using 1,1,2,2-tetrachloroethane as internal standard.

Exp	Lewis Acid	Assay Yield / %
**A**	TMSOTf	38
**B**	TBSOTf	38

7.3.3 TMSOTf and copper iodide optimisation screening

Note: optimisation was performed using tert-butyl bromide as the reagent displayed better stability than tert-butyl iodide.

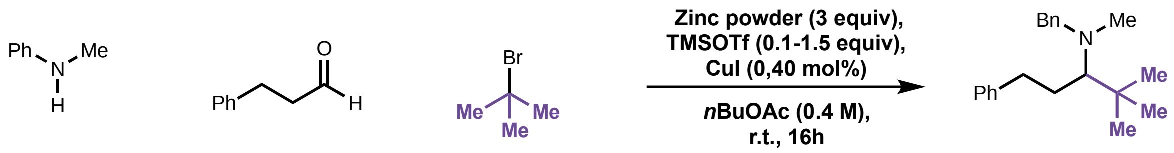

An oven dried vial (Biotage Microwave Process Vial (10 mL), equipped with gas-tight pierceable caps) was charged with a stirring bar, Zn dust (78 mg, 1.2 mmol, 3 equiv) and copper iodide (0-40 mol%). The vial was sealed, evacuated, and backfilled with N_2. To this was added 1.0 ml *n*BuOAc, followed by the N-methylbenzylamine (52 µL, 0.4 mmol, 1.0 equiv), the hydrocinamaldehyde (100 µL, 0.75 mmol, 1.9 equiv). The mixture was stirred for 15 minutes then, whilst stirring, tert-butyl bromide (180 µL, 1.6 mmol, 4 equiv) and TMSOTf (0.1-1.5 equiv) was added. The reaction mixture was stirred overnight. The reaction mixture was worked up according to general procedure **E** and yields were determined by ^{1}H NMR using 1,1,2,2-tetrachloroethane as internal standard.

**Exp**	**TMSOTf loading / equiv**	**Additive**	**Assay Yield /%**
**A**	1.5	copper iodide (40 mol%)	18
**B**	1.0	copper iodide (40 mol%)	21
**C**	0.5	copper iodide (40 mol%)	57
**D**	0.1	copper iodide (40 mol%)	86
**E**	0.1	none	41

From Iodide

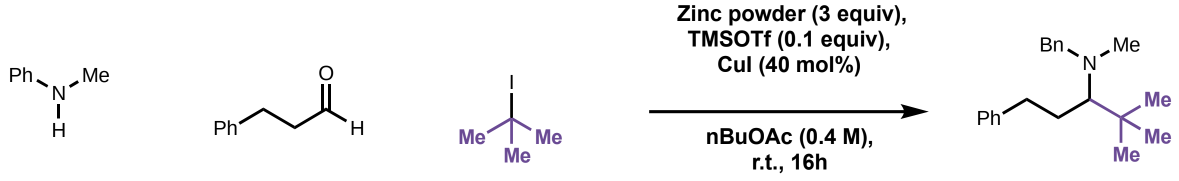

The synthesis of N-benzyl-N,4,4-trimethyl-1-phenylpentan-3-amine was attempted according to General Procedure **E** (with modifications) using zinc (78 mg, 1.2 mmol, 3 equiv), copper iodide (30 mg, 0.16 mmol, 40 mol%), N-methylbenzylamine (52 µL, 0.4 mmol, 1.0 equiv), hydrocinamaldehyde (100 µL, 0.75 mmol, 1.9 equiv), tertbutyliodide (160 µL, 1.6 mmol, 4 equiv) and TMSOTf (7 µL, 0.04 mmol, 10 mol%) in *n*BuOAc (1 mL). The reaction mixture was stirred overnight. The reaction mixture was worked up according to general procedure **E**. Yield of the product was determined by ^{1}H NMR using 1,1,2,2-tetrachloroethane as internal standard indicating a quantitative yield based on the diastereotopic benzyl peak at 3.99 ppm (spectrum shown below).

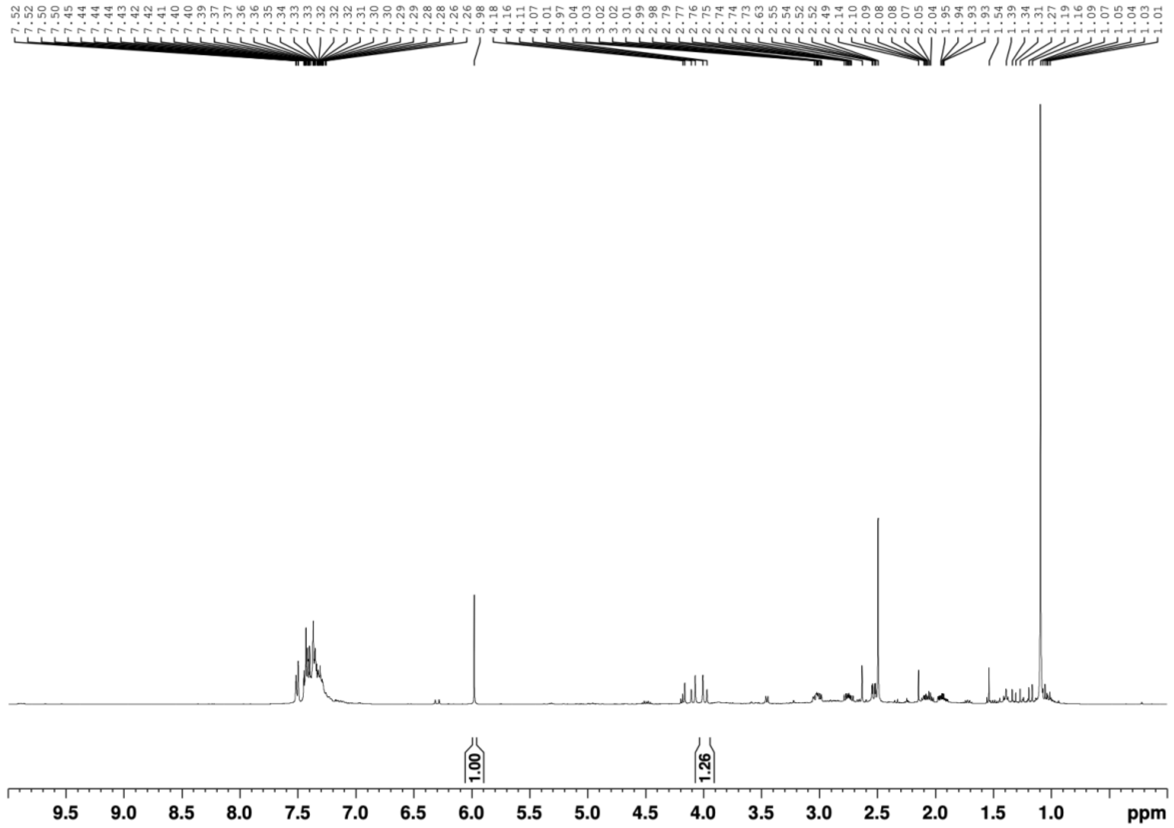

From Bromide - N-benzyl-N,4,4-trimethyl-1-phenylpentan-3-amine (10o)

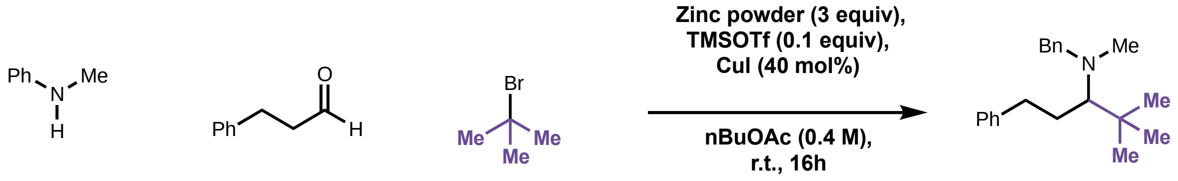

The synthesis of N-benzyl-N,4,4-trimethyl-1-phenylpentan-3-amine was performed according to General Procedure **E** (with modifications) using zinc (78 mg, 1.2 mmol, 3 equiv), copper iodide (30 mg, 0.16 mmol, 40 mol%), N-methylbenzylamine (52 µL, 0.4 mmol, 1.0 equiv), hydrocinamaldehyde (100 µL, 0.75 mmol, 1.9 equiv), tertbutylbromide (180 µL, 1.6 mmol, 4 equiv) and TMSOTf (7 µL, 0.04 mmol, 10 mol%) in *n*BuOAc (1 mL). The reaction mixture was stirred overnight. The reaction mixture was worked up according to general procedure **E**. Crude material was purified by column chromatography (0-10% (10% Et_2O:90% Pet. Ether: NEt_3 3%) in Pet. Ether), yielding the product as a colourless oil (77 mg, 63%). Characterisation agreed with reported data.[1]

From Chloride

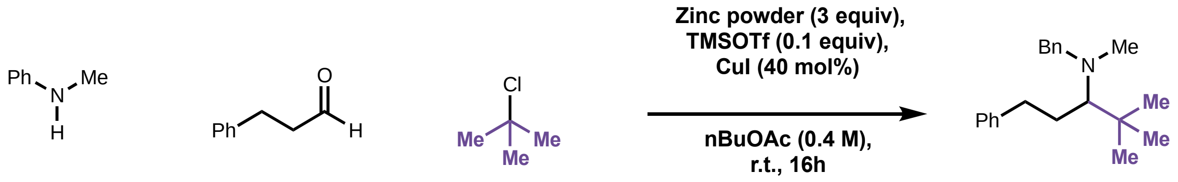

The synthesis of N-benzyl-N,4,4-trimethyl-1-phenylpentan-3-amine was attempted according to General Procedure **E** (with modifications) using zinc (78 mg, 1.2 mmol, 3 equiv), copper iodide (30 mg, 0.16 mmol, 40 mol%), N-methylbenzylamine (52 µL, 0.4 mmol, 1.0 equiv), hydrocinamaldehyde (100 µL, 0.75 mmol, 1.9 equiv), tertbutylchloride (174 µL, 1.6 mmol, 4 equiv) and TMSOTf (7 µL, 0.04 mmol, 10 mol%) in *n*BuOAc (1 mL). The reaction mixture was stirred overnight. The reaction mixture was worked up according to general procedure **B** and analysed by ^{1}H NMR using 1,1,2,2-tetrachloroethane as internal standard indicating a 0% yield.

8. Redox-active esters as alkylating feedstocks

8.1 General procedure F (Synthesis of redox-active esters)

DMAP (0.1 equiv) and N-hydroxytetrachlorophthalimide (1 equiv) were dissolved in dichloromethane (0.2 M). The carboxylic acid (1 equiv) and DCC (1 equiv) or DIC (1 equiv) were added. The reaction was stirred at rt overnight. The solid that was precipitated was filtered through celite. The solvent in the filtrate was removed *in vacuo*. The product was purified by column chromatography.

It should be noted that some of the redox-active esters, especially those bearing an electron-withdrawing group at the α-position, are not stable on silica gel, and they should be eluted from the column as quickly as possible. As redox-active esters are prone to hydrolysis, their molecular weight cannot be detected in LC-MS.

8.2 Redox active ester characterisation

4,5,6,7-tetrachloro-1,3-dioxoisoindolin-2-yl propionate (15a)

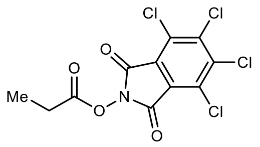

4,5,6,7-tetrachloro-1,3-dioxoisoindolin-2-yl propionate was prepared according to General Procedure **F** using propionic acid (224 µL, 3 mmol, 1 equiv), DMAP (37 mg, 0.3 mmol, 0.1 equiv), *N*-hydroxytetrachlorophthalimide (903 mg, 3 mmol, 1 equiv) and DIC (0.47 mL, 3 mmol, 1 equiv) in dichloromethane (15 mL). Crude material was purified by column chromatography (35-50% dichloromethane in Pet. Ether), yielding the product as a white solid (676 mg, 63%). Data was in line with previous characterisation.[7]

4,5,6,7-tetrachloro-1,3-dioxoisoindolin-2-yl isobutyrate (15b)

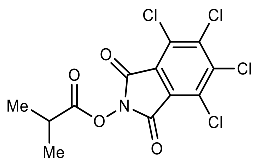

4,5,6,7-tetrachloro-1,3-dioxoisoindolin-2-yl isobutyrate was prepared according to General Procedure **F** using isobutyric acid (556 µL, 6 mmol, 1 equiv), DMAP (73 mg, 0.6 mmol, 0.1 equiv), N-hydroxytetrachlorophthalimide (1.8 g, 6 mmol, 1 equiv), and DIC (0.93 mL, 6 mmol, 1 equiv) in dichloromethane (30 mL). Crude material was purified by column chromatography (35-50% dichloromethane in Pet. Ether), yielding the product as a white solid (1.86 g, 84%). Data was in line with previous characterisation.[8]

4,5,6,7-tetrachloro-1,3-dioxoisoindolin-2-yl pivalate (15c)

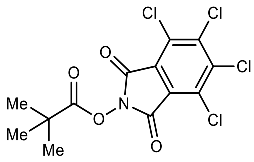

4,5,6,7-tetrachloro-1,3-dioxoisoindolin-2-yl pivalate was prepared according to General Procedure **F** using pivalic acid (303 mg, 3 mmol, 1 equiv), DMAP (37 mg, 0.3 mmol, 0.1 equiv), *N*-hydroxytetrachlorophthalimide (903 mg, 3 mmol, 1 equiv) and DCC (619 mg, 3 mmol, 1 equiv) in dichloromethane (15 mL). Crude material was purified by column chromatography (70-80% dichloromethane in Pet. Ether), yielding the product as a white solid (937 mg, 82%).

mp: 146-152˚C

^{1}H NMR (400 MHz, $CDCl_3$): δ 1.43 (s, 9H).

^{13}C NMR (101 MHz, $CDCl_3$): δ 174.0, 157.9, 141.1, 130.7, 125.0, 38.6, 27.1.

IR (solid, cm^{-1}): 2987, 2976, 1736, 1379, 1364, 1353, 1155, 1053, 1007, 851, 791, 723, 698.

HRMS (ESI-TOF) compound unstable

4,5,6,7-tetrachloro-1,3-dioxoisoindolin-2-yl acetate (15d)

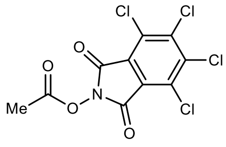

4,5,6,7-tetrachloro-1,3-dioxoisoindolin-2-yl acetate was prepared according to General Procedure **F** using acetic acid (0.17 mL, 3 mmol, 1 equiv), DMAP (37 mg, 0.3 mmol, 0.1 equiv), N-hydroxytetrachlorophthalimide (903 mg, 3 mmol, 1 equiv) and DIC (0.47 mL, 3 mmol, 1 equiv) in dichloromethane (15 mL). Crude material was purified by column chromatography (75-85% dichloromethane in Pet. Ether), yielding the product as a white solid (863 mg, 84%).

mp: 160-168˚C

^{1}H NMR (400 MHz, $CDCl_3$): δ 2.41 (s, 3H).

^{13}C NMR (101 MHz, $CDCl_3$): δ 166.3, 157.7, 141.2, 130.7, 124.9, 17.7.

IR (solid, cm^{-1}): 2945, 1819, 1795, 1745, 1379, 1363, 1300, 1198, 1154, 1036, 730.

HRMS (ESI-TOF) compound unstable

4,5,6,7-tetrachloro-1,3-dioxoisoindolin-2-yl 1-methylcyclopropane-1-carboxylate (15e)

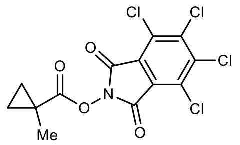

4,5,6,7-tetrachloro-1,3-dioxoisoindolin-2-yl 1-methylcyclopropane-1-carboxylate was prepared according to General Procedure **F** using 1-methylcyclopropanecarboxylic acid (300 mg, 3 mmol, 1 equiv), DMAP (37 mg, 0.3 mmol, 0.1 equiv), N-hydroxytetrachlorophthalimide (903 mg, 3 mmol, 1 equiv), and DIC (0.47 mL, 3 mmol, 1 equiv) in dichloromethane (15 mL). Crude material was purified by column chromatography (30-40% dichloromethane in Pet. Ether), yielding the product as a white solid (937 mg, 84%). Data was in line with previous characterisation.[9]

4,5,6,7-tetrachloro-1,3-dioxoisoindolin-2-yl cyclobutanecarboxylate (15f)

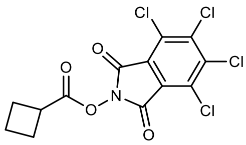

4,5,6,7-tetrachloro-1,3-dioxoisoindolin-2-yl cyclobutanecarboxylate was prepared according to General Procedure **F** using cyclobutanecarboxylic acid (287 µL, 3 mmol, 1 equiv), DMAP (37 mg, 0.3 mmol, 0.1 equiv), N-hydroxytetrachlorophthalimide (903 mg, 3 mmol, 1 equiv) and DIC (0.47 mL, 3 mmol, 1 equiv) in dichloromethane (15 mL). Crude material was purified by column chromatography (30-40% dichloromethane in Pet. Ether) yielding the product as a white solid (906 mg, 79%). Data was in line with previous characterisation.[10]

4,5,6,7-tetrachloro-1,3-dioxoisoindolin-2-yl 3-methyloxetane-3-carboxylate (15g)

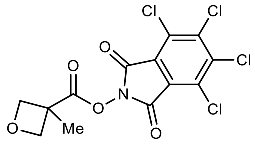

4,5,6,7-tetrachloro-1,3-dioxoisoindolin-2-yl 3-methyloxetane-3-carboxylate. was prepared according to General Procedure **F** using 3-methyloxetane-3-carboxylic acid (348 mg, 3 mmol, 1 equiv), DMAP (37 mg, 0.3 mmol, 0.1 equiv), N-hydroxytetrachlorophthalimide (903 mg, 3 mmol, 1 equiv) and DIC (0.47 mL, 3 mmol, 1 equiv) in dichloromethane (15 mL). Crude material was purified by column chromatography (70-100% dichloromethane in Pet. Ether), yielding the product as a white solid (620 mg, 52%). Data was in line with previous characterisation.[9]

4,5,6,7-tetrachloro-1,3-dioxoisoindolin-2-yl 4-methyltetrahydro-2H-pyran-4-carboxylate (15h)

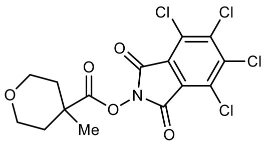

4,5,6,7-tetrachloro-1,3-dioxoisoindolin-2-yl 4-methyltetrahydro-2H-pyran-4-carboxylate was prepared according to General Procedure **F** using 4-methyltetrahydro-2H-pyran-4-carboxylic acid (433 mg, 3 mmol, 1 equiv), DMAP (37 mg, 0.3 mmol, 0.1 equiv), N-hydroxytetrachlorophthalimide (903 mg, 3 mmol, 1 equiv) and DIC (0.47 mL, 3 mmol, 1 equiv) in dichloromethane (15 mL). Crude material was purified by column chromatography (70-95% dichloromethane in Pet. Ether), yielding the product as a while solid (1.1 g, 82%). Data was in line with previous characterisation.[11]

4,5,6,7-tetrachloro-1,3-dioxoisoindolin-2-yl cycloheptanecarboxylate (15i)

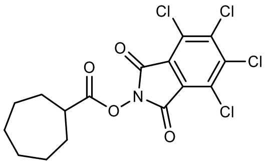

4,5,6,7-tetrachloro-1,3-dioxoisoindolin-2-yl cycloheptanecarboxylate was prepared according to General Procedure **F** using cycloheptanecarboxylic acid (0.41 mL, 3 mmol, 1 equiv), DMAP (37 mg, 0.3 mmol, 0.1 equiv), N-hydroxytetrachlorophthalimide (903 mg, 3 mmol, 1 equiv) and DIC (0.47 mL, 3 mmol, 1 equiv) in dichloromethane (15 mL). The crude product was purified by column chromatography (50% dichloromethane in Pet. Ether), yielding the product as a while solid (1.0 g, 80%).

^{1}H NMR (400 MHz, $CDCl_3$): δ 2.93-2.85 (m, 1H), 2.16 - 2.06 (m, 2H), 1.92-1.74 (m, 4H), 1.66 - 1.50 (m, 6H).

^{13}C NMR (101 MHz, $CDCl_3$): δ 172.5, 158.0, 141.1, 130.6, 125.0, 42.2, 30.8, 28.4, 26.3.

IR (solid, cm^{-1}): 2926, 2857, 1815, 1789, 1743, 1461, 1378, 1366, 1298, 1197, 1154, 1076, 1036, 993, 958, 934, 858, 794, 729, 691.

HRMS: m/z calcd for $C_8H_{14}O_2$ $[M-C_8HCl_4NO_2]^-$ 141.0921; found 141.0915.

4,5,6,7-tetrachloro-1,3-dioxoisoindolin-2-yl 3-oxocyclobutane-1-carboxylate (15j)

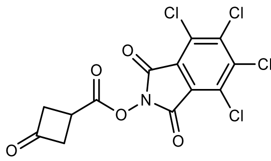

4,5,6,7-tetrachloro-1,3-dioxoisoindolin-2-yl 3-oxocyclobutane-1-carboxylate was prepared according to General Procedure **F** using 3-oxocyclobutane-1-carboxylic acid (342 mg, 3 mmol, 1 equiv), DMAP (37 mg, 0.3 mmol, 0.1 equiv), N-hydroxytetrachlorophthalimide (903 mg, 3 mmol, 1 equiv) and DIC (0.47 mL, 3 mmol, 1 equiv) in dichloromethane (15 mL). After recrystallization in dichloromethane/^{i}PrOH at -20 °C overnight and subsequent column chromatography (dichloromethane), the product was obtained as a while solid (580 mg, 49%).

mp: 168-176°C

^{1}H NMR (400 MHz, $CDCl_3$): δ 3.70 - 3.45 (m, 5H).

^{13}C NMR (101 MHz, $CDCl_3$): δ 201.0, 170.5, 157.6, 141.5, 130.8, 124.8, 52.4, 25.1.

IR (solid, cm^{-1}): 2989, 2972, 2939, 1819, 1780, 1741, 1378, 1300, 1200, 1160, 1143, 1123, 1103, 1085, 1033, 959, 859, 808, 794, 787, 728, 685, 613.

HRMS (ESI-TOF) compound unstable

4,5,6,7-tetrachloro-1,3-dioxoisoindolin-2-yl 3,3-difluorocyclobutane-1-carboxylate (15k)

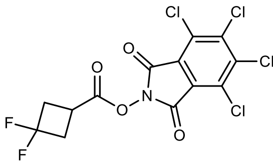

4,5,6,7-tetrachloro-1,3-dioxoisoindolin-2-yl 3,3-difluorocyclobutane-1-carboxylate was prepared according to General Procedure **F** using 3,3-difluoro-cyclobutanecarboxylic acid (408 mg, 3 mmol, 1 equiv), DMAP (37 mg, 0.3 mmol, 0.1 equiv), N-hydroxytetrachlorophthalimide (903 mg, 3 mmol, 1 equiv) and DIC (0.47 mL, 3 mmol, 1 equiv) in dichloromethane (15 mL). Crude material was purified by column chromatography (30-50% dichloromethane in Pet. Ether), yielding the product as a while solid (612 mg, 49%).

mp: 156-160°C

^{1}H NMR (400 MHz, $CDCl_3$): δ 3.38 - 3.31 (m, 1H), 3.13 - 2.99 (m, 4H).

^{13}C NMR (101 MHz, $CDCl_3$): δ 169.6 (t, J = 2.9 Hz), 157.3, 141.3, 130.6, 124.6, 117.9 (dd, J = 283.7, 271.2 Hz), 39.3 (t, 25.5 z), 24.2 (dd, J = 14, 6.0 Hz).

^{19}F NMR (375 MHz, $CDCl_3$): δ -83.3 (d, J = 195.6 Hz), -96.0 (d, J = 195.4 Hz).

IR (solid, cm^{-1}): 2984, 1739, 1300, 1234, 1154, 1105, 1042, 958, 916, 886, 876, 788, 723, 705, 611.

HRMS (ESI-TOF) compound unstable

4,5,6,7-tetrachloro-1,3-dioxoisoindolin-2-yl 3-methoxypropanoate (15l)

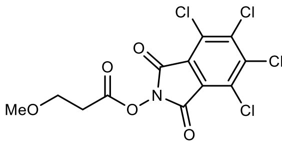

4,5,6,7-tetrachloro-1,3-dioxoisoindolin-2-yl 3-methoxypropanoate was prepared according to General Procedure **F** using 3-methoxypropionic acid (0.28 mL, 3 mmol, 1 equiv), DMAP (37 mg, 0.3 mmol, 0.1 equiv), N-hydroxytetrachlorophthalimide (903 mg, 3 mmol, 1 equiv) and DIC (0.47 mL, 3 mmol, 1 equiv) in dichloromethane (15 mL). Crude material was purified by column chromatography (80-100% dichloromethane in Pet. Ether), yielding the product as a while solid (462 mg, 40%).

^{1}H NMR (400 MHz, $CDCl_3$): δ 3.78 (t, J = 6.3 Hz, 2H), 3.41 (s, 3H), 2.93 (t, J = 6.2 Hz, 2H).

^{13}C NMR (101 MHz, $CDCl_3$): δ 167.4, 157.6, 141.2, 130.7, 124.9, 67.2, 59.2, 32.2.

IR (solid, cm^{-1}): 2977, 2924, 2884, 2846, 2827, 1831, 1796, 1744, 1412, 1397, 1379, 1354, 1300, 1257, 1237, 1188, 1160, 1115, 1084, 1047, 1012, 985, 961, 833, 790, 726, 713, 619.

HRMS: m/z calcd for $C_{12}H_7NO_5Cl_4$ $[M+H]^+$ 407.8971; found 407.8971.

4,5,6,7-tetrachloro-1,3-dioxoisoindolin-2-yl 2-(benzyloxy)acetate (15m)

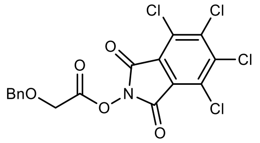

4,5,6,7-tetrachloro-1,3-dioxoisoindolin-2-yl 2-(benzyloxy)acetate was prepared according to General Procedure **F** using benzyloxyacetic acid (0.43 mL, 3 mmol, 1 equiv), DMAP (37 mg, 0.3 mmol, 0.1 equiv), N-hydroxytetrachlorophthalimide (903 mg, 3 mmol, 1 equiv) and DIC (0.47 mL, 3 mmol, 1 equiv) in dichloromethane (15 mL). Crude material was purified by column chromatography (40-70% dichloromethane in Pet. Ether), yielding the product as a while solid (620 mg, 46%). Data was in line with previous characterisation.[8]

4,5,6,7-tetrachloro-1,3-dioxoisoindolin-2-yl tetrahydrofuran-2-carboxylate (15n)

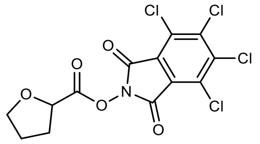

4,5,6,7-tetrachloro-1,3-dioxoisoindolin-2-yl tetrahydrofuran-2-carboxylate was prepared according to General Procedure **F** using tetrahydro-2-furoic acid (287 mL, 3 mmol, 1 equiv), DMAP (37 mg, 0.3 mmol, 0.1 equiv), N-hydroxytetrachlorophthalimide (903 mg, 3 mmol, 1m equiv) and DIC (0.47 mL, 3 mmol, 1 equiv) in dichloromethane (15 mL). Crude material was purified by column chromatography (80-100% dichloromethane in Pet. Ether), yielding the product as a while solid (567 mg, 47%). Data was in line with previous characterisation.[12]

4,5,6,7-Tetrachloro-1,3-dioxoisoindolin-2-yl 2-(methylthio)acetate (15l)

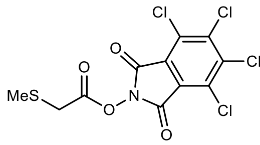

4,5,6,7-tetrachloro-1,3-dioxoisoindolin-2-yl 2-(methylthio)acetate was prepared according to General Procedure **F** using (methylthio)acetic acid (0.26 mL, 3 mmol, 1 equiv), DMAP (37 mg, 0.3 mmol, 0.1 equiv), N-hydroxytetrachlorophthalimide (903 mg, 3 mmol, 1 equiv) and DIC (0.47 mL, 3 mmol, 1 equiv) in dichloromethane (15 mL). Crude material was purified by column chromatography (30-65% dichloromethane in Pet. Ether), yielding the product as a while solid (780 mg, 67%).

mp: 146-150°C

^{1}H NMR (400 MHz, $CDCl_3$): δ 3.50 (s, 2H), 2.34 (s, 3H).

^{13}C NMR (101 MHz, $CDCl_3$): δ 166.3, 157.5, 141.4, 130.8, 124.8, 32.5, 16.4.

IR (solid, cm^{-1}): 2987, 2923, 1814, 1791, 1730, 1367, 1195, 1148, 1072, 1033, 792, 726.

HRMS (ESI-TOF) compound unstable

4,5,6,7-Tetrachloro-1,3-dioxoisoindolin-2-yl 2-(1,3-dioxoisoindolin-2-yl)acetate (15m)

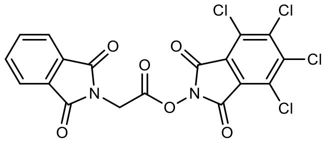

4,5,6,7-Tetrachloro-1,3-dioxoisoindolin-2-yl 2-(1,3-dioxoisoindolin-2-yl)acetate was prepared according to General Procedure **F** using N-phthaloylglycine (616 mg, 3 mmol 1 equiv), DMAP (37 mg, 0.3 mmol, 0.1 equiv), N-hydroxytetrachlorophthalimide (903 mg, 3 mmol, 1 equiv) and DIC (0.47 mL, 3 mmol, 1 equiv) in dichloromethane (15 mL). Crude material was purified by column chromatography (80% dichloromethane in Pet. Ether), yielding the product as a while solid (520 mg, 36%).

^{1}H NMR (400 MHz, $CDCl_3$): δ 7.95 - 7.90 (m, 2H), 7.81 - 7.75 (m, 2H), 4.85 (s, 2H).

^{13}C NMR (101 MHz, $CDCl_3$): δ 166.8, 164.1, 157.0, 141.4, 134.8, 131.9, 130.9, 124.7, 124.2, 36.8.

IR (solid, cm^{-1}): 3030, 3000, 2981, 2950, 1834, 1797, 1776, 1748, 1721, 1379, 1089, 947, 729, 712.

HRMS: m/z calcd for $C_{18}H_6N_2O_6Cl_4$ $[M+H]^+$ 508.8872; found 508.8869.

4,5,6,7-tetrachloro-1,3-dioxoisoindolin-2-yl 2-fluoro-2-methylpropanoate (15n)

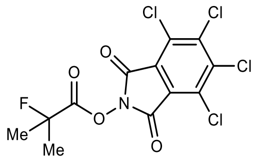

4,5,6,7-tetrachloro-1,3-dioxoisoindolin-2-yl 2-fluoro-2-methylpropanoate was prepared according to General Procedure **F** using 2-fluoro-2-methylpropanoic acid (0.28 mL, 3 mmol, 1 equiv), DMAP (37 mg, 0.3 mmol, 0.1 equiv), N-hydroxytetrachlorophthalimide (903 mg, 3 mmol, 1 equiv) and DIC (0.47 mL, 3 mmol, 1 equiv) in dichloromethane (15 mL). Crude material was purified by column chromatography (50% dichloromethane in Pet. Ether), yielding the product as a while solid (357 mg, 31%). Data was in line with previous characterisation.[9]

8.3 General procedure G (Redox-active esters)

An oven dried vial (Biotage Microwave Process Vial 10 mL, equipped with gas-tight pierceable caps) was charged with a stirring bar. The vial was sealed, evacuated, and backfilled with N_2. To this was added 0.5 ml dry EtOAc, followed by N-methylbenzylamine (13 μL, 0.1 mmol, 1 equiv), hydrocinnamaldehyde (26 μL, 0.2 mmol, 2 equiv) and TMSOTf (32 μL, 0.175 mmol, 1.75 equiv). The mixture was stirred for 15 mins. The cap was removed. Zinc (20 mg, 0.3 mmol, 3 equiv) and the redox active ester (0.2 mmol, 2 equiv) were added in one portion. The microwave vial was sealed, put under vacuum and refilled with N_2. The mixture was stirred overnight. NEt_3 (0.2 mL) was added. The mixture was stirred for 15 mins. The mixture was dilute with Et_2O and stirred for 15 mins. The white precipitate formed was removed by filtration through celite. Solvent was removed *in vacuo*. The white solid that remained was dissolved in a minimum amount of dichloromethane. The mixture was diluted with Pet. Ether until no more precipitate was formed (Note: The precipitate should appear as fine powder. If it appeared as a lump, it was re-dissolved in a minimum amount of dichloromethane and diluted with Pet. Ether. It is because the product can be trapped in the lump) The precipitate was removed by filtered through celite. Solvent was removed *in vacuo*. The resulting oil was purified by an acid-base wash. If necessary, the product was further purified by column chromatography.

For rapid assay yields an alternative workup procedure can be used. The reaction mixture can be poured on to 600 mg of ground amberlite IRA 96 resin. The mixture was then passed through the resin using 50 mL of dichloromethane. The organic solution was then passed through $MgSO_4$ and an assay yield could be attained using ^{1}H NMR using 1,3,5-trimethoxybenzene as an internal standard (*1,1,2,2-tetrachloroethane is not suitable with these reaction mixtures before an acid-base workup is applied*).

8.4 Redox-active esters scope

N-benzyl-N-methyl-1-phenylpentan-3-amine (16a)

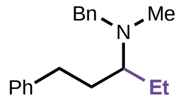

N-benzyl-N-methyl-1-phenylpentan-3-amine was prepared according to General Procedure **G** using Zn (20 mg, 0.3 mmol, 3 equiv), N-methylbenzylamine (13 µL, 0.1 mmol, 1 equiv), hydrocinnamaldehyde (26 µL, 0.2 mmol, 2 equiv), TMSOTf (32 µL, 0.175 mmol, 1.75 equiv) and 4,5,6,7-tetrachloro-1,3-dioxoisoindolin-2-yl propionate (71 mg, 0.2 mmol, 2 equiv) in EtOAc (0.5 mL). The pure product was obtained as a colourless oil (23 mg, 88%).

^{1}H NMR (400 MHz, $CDCl_3$): δ 7.39 - 7.17 (m, 10H), 3.63 (d, J = 13.6 Hz, 1H), 3.57 (d, J = 13.5 Hz, 1H), 2.84 - 2.75 (m, 1H), 2.72 - 2.62 (m, 1H), 2.50-2.41 (m, 1H), 2.18 (s, 3H), 1.88 - 1.77 (m, 1H), 1.70 - 1.62 (m, 2H), 0.95 (t, J = 7.6 Hz, 3H).

^{13}C NMR (101 MHz, $CDCl_3$): δ 143.4, 141.0, 128.9, 128.7, 128.5, 128.4, 126.8, 125.8, 64.3, 58.1, 36.6, 33.8, 32.3, 22.3, 12.2.

IR (film, cm^{-1}): 3025, 2957, 2928, 2872, 2786, 1602, 1494, 1453, 1358, 1073, 1027, 908, 732, 697.

HRMS: m/z calcd for $C_{19}H_{25}N$ $[M+H]^+$ 268.2060; found 268.2069.

N-benzyl-N,4-dimethyl-1-phenylpentan-3-amine (4a)

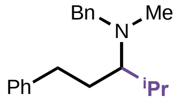

N-benzyl-N,4-dimethyl-1-phenylpentan-3-amine was prepared according to General Procedure **G** using Zn (20 mg, 0.3 mmol, 3 equiv), N-methylbenzylamine (13 µL, 0.1 mmol, 1 equiv), hydrocinnamaldehyde (26 µL, 0.2 mmol, 2 equiv), TMSOTf (32 µL, 0.175 mmol, 1.75 equiv) and 4,5,6,7-tetrachloro-1,3-dioxoisoindolin-2-yl isobutyrate (74 mg, 0.2 mmol, 2 equiv) in EtOAc (0.5 mL). The pure product was obtained as a colourless oil (26 mg, 92%). Data was in line with previous characterisation for **4a**.

N-benzyl-N,4,4-trimethyl-1-phenylpentan-3-amine (10o)

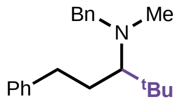

N-benzyl-N,4,4-trimethyl-1-phenylpentan-3-amine was prepared according to General Procedure **G** using Zn (20 mg, 0.3 mmol, 3 equiv), N-methylbenzylamine (13 µL, 0.1 mmol, 1 equiv), hydrocinnamaldehyde (26 µL, 0.2 mmol, 2 equiv), TMSOTf (32 µL, 0.175 mmol, 1.75 equiv) and 4,5,6,7-tetrachloro-1,3-dioxoisoindolin-2-yl pivalate (77 mg, 0.2 mmol, 2 equiv) in EtOAc (0.5 mL). The pure product was obtained as a colourless oil (22 mg, 75%). Data was in line with previous characterisation.[1]

N-benzyl-N-methyl-4-phenylbutan-2-amine (10m)

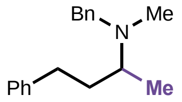

N-benzyl-N-methyl-4-phenylbutan-2-amine was prepared according to General Procedure **G** using Zn (20 mg, 0.3 mmol, 3 equiv), N-methylbenzylamine (13 µL, 0.1 mmol, 1 equiv), hydrocinnamaldehyde (26 µL, 0.2 mmol, 2 equiv), TMSOTf (32 µL, 0.175 mmol, 1.75 equiv) and 4,5,6,7-tetrachloro-1,3-dioxoisoindolin-2-yl acetate (69 mg, 0.2 mmol, 2 equiv) in EtOAc (0.5 mL). Crude material was subjected to an acid-base wash according to general procedure **ABW**, followed by column chromatography (0-7% Et_2O in Pet. Ether) yielding the product as a colourless oil (13 mg, 51%). Data was in line with previous characterisation.[1]

N-benzyl-N-methyl-1-(1-methylcyclopropyl)-3-phenylpropan-1-amine amine (16b)

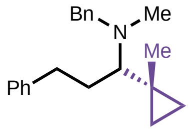

N-benzyl-N-methyl-1-(1-methylcyclopropyl)-3-phenylpropan-1-amine amine was prepared according to General Procedure **G** using Zn (20 mg, 0.3 mmol, 3 equiv), N-methylbenzylamine (13 µL, 0.1 mmol, 1 equiv), hydrocinnamaldehyde (26 µL, 0.2 mmol, 2 equiv), TMSOTf (32 µL, 0.175 mmol, 1.75 equiv) and 4,5,6,7-tetrachloro-1,3-dioxoisoindolin-2-yl 1-methylcyclopropane-1-carboxylate (77 mg, 0.2 mmol, 2 equiv) in EtOAc (0.5 mL) with modifications. The acidic aqueous layer was basified with Na_2CO_3 (saturated, aq), instead of NaOH (10%, aq). The crude product was subjected to an acid-base wash according to general procedure **ABW**, followed by column chromatography (0-15% Et_2O in Pet. Ether) yielding the product as a colourless oil (18 mg, 62%).

^{1}H NMR (400 MHz, $CDCl_3$): δ 7.43 - 7.19 (m, 10H), 3.96 (d, J = 13.6 Hz, 1H), 3.65 (d, J = 13.5 Hz, 1H), 2.86 - 2.71 (m, 2H), 2.32 (s, 3H), 2.15 - 2.02 (m, 1H), 1.95 - 1.83 (m, 2H), 1.17 (s, 3H), 0.60 - 0.52 (m, 1H), 0.48 - 0.41 (m, 1H), 0.28 - 0.18 (m, 2H).

^{13}C NMR (101 MHz, $CDCl_3$): δ 143.2, 141.1, 128.6, 128.52, 128.47, 128.3, 126.7, 125.8, 70.1, 59.1, 39.0, 34.5, 32.7, 21.0, 18.2, 14.5, 12.3.

IR (film, cm^{-1}): 3083, 3063, 3025, 2994, 2951, 2870, 2783, 1603, 1495, 1453, 1028, 736, 698.

HRMS: m/z calcd for $C_{21}H_{27}N$ $[M+H]^+$ 294.2217; found 294.2219.

N-benzyl-1-cyclobutyl-N-methyl-3-phenylpropan-1-amine (16c)

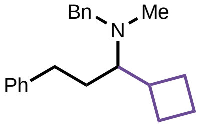

N-benzyl-1-cyclobutyl-N-methyl-3-phenylpropan-1-amine was prepared according to General Procedure **G** using Zn (20 mg, 0.3 mmol, 3 equiv), N-methylbenzylamine (13 µL, 0.1 mmol, 1 equiv), hydrocinnamaldehyde (26 µL, 0.2 mmol, 2 equiv), TMSOTf (32 µL, 0.175 mmol, 1.75 equiv) and 4,5,6,7-tetrachloro-1,3-dioxoisoindolin-2-yl cyclobutanecarboxylate (77 mg, 0.2 mmol, 2 equiv) in EtOAc (0.5 mL). The pure product was obtained as a colourless oil (26 mg, 89%).

^{1}H NMR (500 MHz, $CDCl_3$): δ 7.31 - 7.05 (m, 10H), 3.59 (d, J = 13.6 Hz, 1H), 3.55 (d, J = 13.6 Hz, 1H), 2.81 - 2.73 (m, 1H), 2.61 - 2.40 (m, 3H), 2.10 (s, 3H), 2.02 - 1.92 (m, 1H), 1.90 - 1.72 (m, 3H), 1.69 -1.43 (m, 4H).

^{13}C NMR (176 MHz, $CDCl_3$): δ 143.4, 141.0, 128.49, 128.45, 128.3, 128.2, 126.6, 125.6, 68.1, 58.9, 38.2, 37.1, 33.5, 30.9, 291, 28.0, 19.1.

IR (film, cm^{-1}): 3025, 2933, 2956, 2787, 1494, 1453, 1020, 733, 698.

HRMS: m/z calcd for $C_{21}H_{27}N$ $[M+H]^+$ 294.2217; found 294.2229.

N-benzyl-N-methyl-1-(3-methyloxetan-3-yl)-3-phenylpropan-1-amine (16d)

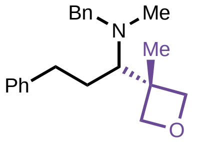

N-benzyl-N-methyl-1-(3-methyloxetan-3-yl)-3-phenylpropan-1-amine was prepared according to General Procedure **G** using Zn (20 mg, 0.3 mmol, 3 equiv), N-methylbenzylamine (13 µL, 0.1 mmol, 1 equiv), hydrocinnamaldehyde (26 µL, 0.2 mmol, 2 equiv), TMSOTf (32 µL, 0.175 mmol, 1.75 equiv) and 4,5,6,7-tetrachloro-1,3-dioxoisoindolin-2-yl 3-methyloxetane-3-carboxylate (80 mg, 0.2 mmol, 2 equiv) in EtOAc (0.5 mL). The pure product was obtained as a colourless oil (19 mg, 60%).

^{1}H NMR (400 MHz, $CDCl_3$): δ 7.38 - 7.31 (m, 6H), 7.30-7.21 (m, 4H), 4.71 (d, J = 5.8 Hz, 1H), 4.53 (d, J = 5.6 Hz, 1H), 4.33 (d, J = 5.8 Hz, 1H), 4.21 (d, J = 5.6 Hz, 1H), 3.83 (d, J = 13.7 Hz, 1H), 3.76 (d, J = 13.8 Hz, 1H), 3.04 (dd, J = 8.4 Hz, 4.0 Hz, 1H), 2.92 - 2.81 (m, 1H), 2.70-2.60 (m, 1H), 2.29 (s, 3H), 2.12 - 1.99 (m, 1H), 1.74 - 1.61 (m, 1H), 1.50 (s, 3H).

^{13}C NMR (101 MHz, $CDCl_3$): δ 142.3, 140.6, 128.7, 128.5, 128.3, 127.0, 126.2, 82.9, 82.2, 68.5, 60.5, 45.0, 39.0, 35.2, 29.1, 21.5.

IR (film, cm^{-1}): 3025, 2932, 2863, 2787, 1602, 1494, 1452, 977, 736, 698.

HRMS: m/z calcd for $C_{21}H_{27}NO$ $[M+H]^+$ 310.2166, found 310.2168.

3-(1-(benzyl(methyl)amino)-3-phenylpropyl)cyclobutan-1-one (16e)

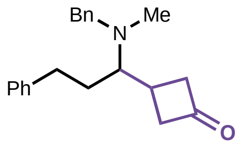

3-(1-(benzyl(methyl)amino)-3-phenylpropyl)cyclobutan-1-one was prepared according to General Procedure **G** using Zn (20 mg, 0.3 mmol, 3 equiv), N-methylbenzylamine (13 µL, 0.1 mmol, 1 equiv), hydrocinnamaldehyde (26 µL, 0.2 mmol, 2 equiv), TMSOTf (32 µL, 0.175 mmol, 1.75 equiv) and 4,5,6,7-tetrachloro-1,3-dioxoisoindolin-2-yl 3-oxocyclobutane-1-carboxylate (79 mg, 0.2 mmol, 2 equiv) with modifications. 0.75 mL of EtOAc, instead of 0.5 mL, was used. Crude material was subjected to an acid-base wash according to general procedure **ABW** followed by column chromatography (0-20% Et_2O in Pet. Ether) yielding the product as a colourless oil (13 mg, 41%).

^{1}H NMR (400 MHz, $CDCl_3$): δ 7.39 - 7.18 (m, 10H), 3.74 (s, 2H), 3.16 - 3.01 (m, 3H), 2.94 - 2.67 (m, 4H), 2.66 - 2.55 (m, 1H), 2.29 (s, 3H), 2.05 - 1.94 (m, 1H), 1.76 - 1.65 (m, 1H).

^{13}C NMR (101 MHz, $CDCl_3$): δ 207.4, 142.5, 140.2, 128.32, 128.58, 128.47 127.1, 126.1, 67.1, 59.1, 52.0, 51.6, 37.0, 33.9, 31.3, 26.6.

IR (film, cm^{-1}): 3060, 3025, 2929, 2858, 2789, 1778, 1742, 1671, 1602, 1495, 1453, 1382, 1178, 1113, 1075, 1028, 748, 699.

HRMS: m/z calcd for $C_{21}H_{25}NO$ $[M+H]^+$ 308.2009; found 308.2012.

N-benzyl-1-(3,3-difluorocyclobutyl)-N-methyl-3-phenylpropan-1-amine (16f)

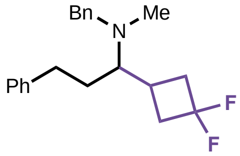

N-benzyl-1-(3,3-difluorocyclobutyl)-N-methyl-3-phenylpropan-1-amine was prepared according to General Procedure **G** using Zn (20 mg, 0.3 mmol, 3 equiv), N-methylbenzylamine (13 µL, 0.1 mmol, 1 equiv), hydrocinnamaldehyde (26 µL, 0.2 mmol, 2 equiv), TMSOTf (32 µL, 0.175 mmol, 1.75 equiv) and 4,5,6,7-tetrachloro-1,3-dioxoisoindolin-2-yl 3,3-difluorocyclobutane-1-carboxylate (84 mg, 0.2 mmol, 2 equiv) in EtOAc (0.5 mL). The crude product was subjected to an acid-base wash according to general procedure **ABW** followed by column chromatography (0-15% Et_2O in Pet. Ether) yielding the product as a colourless oil (13 mg, 40%).

^{1}H NMR (400 MHz, $CDCl_3$): δ 7.33 - 7.19 (m, 10H), 3.66 (s, 2H), 2.83 (ddd, J = 14.0, 9.8, 4.7 Hz, 1H), 2.71 - 2.55 (m, 4H), 2.51 - 2.30 (m, 2H), 2.25 - 2.13 (m, 4H), 1.92-1.80 (m, 1H), 1.63 - 1.54 (m, 1H).

^{13}C NMR (101 MHz, $CDCl_3$): δ 142.6, 140.3, 128.6, 128.53, 128.48, 128.4, 127.0, 126.0, 120.1 (dd, J= 287.2, 269.8 Hz), 67.2 (t, J= 2.3 Hz), 59.0, 40.5 (dd, J= 21.6, 19.3 Hz), 40.1 (dd, J= 21.6, 19.3 Hz), 36.9, 33.7, 31.0, 25.9 (dd, J= 13.8, 3.2 Hz).

IR (film, cm^{-1}): 3026, 2945, 2857, 2790, 1293, 1167, 898, 697.

^{19}F NMR (376 MHz, $CDCl_3$): δ -81.7 (d, J = 191.7 Hz), -101.2 (d, J = 191.6 Hz).

HRMS: m/z calcd for $C_{21}H_{25}F_2N$ $[M+H]^+$ 330.2028; found 330.2035.

N-benzyl-N-methyl-1-(4-methyltetrahydro-2H-pyran-4-yl)-3-phenylpropan-1-amine (16g)

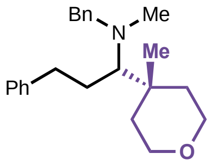

N-benzyl-N-methyl-1-(4-methyltetrahydro-2H-pyran-4-yl)-3-phenylpropan-1-amine was prepared according to General Procedure **G** using Zn (20 mg, 0.3 mmol, 3 equiv), N-methylbenzylamine (13 µL, 0.1 mmol, 1 equiv), hydrocinnamaldehyde (26 µL, 0.2 mmol, 2 equiv), TMSOTf (32 µL, 0.175 mmol, 1.75 equiv) and 4,5,6,7-tetrachloro-1,3-dioxoisoindolin-2-yl 4-methyltetrahydro-2*H*-pyran-4-carboxylate (85 mg, 0.2 mmol, 2 equiv) in EtOAc (0.5 mL). The pure product was obtained as a colourless oil (23 mg, 68%).

^{1}H NMR (400 MHz, $CDCl_3$): δ 7.43 - 7.31 (m, 6H), 7.30 - 7.21 (m, 4H), 4.05 (d, J = 14.0 Hz, 1H), 3.90 (d, J = 13.9 Hz, 1H), 3.86 - 3.72 (m, 2H), 3.66 - 3.55 (m, 2H), 2.98 - 2.88 (m, 1H), 2.72-2.60 (m, 1H), 2.54 - 2.46 (m, 1H), 2.43 (s, 3H), 2.09-1.96 (m, 1H), 1.89 - 1.56 (m, 4H), 1.32 - 1.24 (m, 1H), 1.07 (s, 3H).

^{13}C NMR (101 MHz, $CDCl_3$): δ 142.8, 141.1, 128.6, 128.5, 128.4, 126.8, 126.0, 73.5, 64.4, 63.7, 38.0, 36.8, 36.5, 36.0, 28.4, 18.6.

IR (thin film, cm^{-1}): 3025, 2952, 2848, 2783, 1494, 14522, 1109, 1027, 733, 698.

HRMS: m/z calcd for $C_{23}H_{31}NO$ $[M+H]^+$ 338.2479; found 338.2483.

N-benzyl-1-cycloheptyl-N-methyl-3-phenylpropan-1-amine (16h)

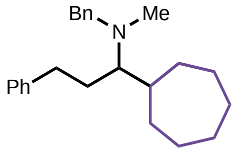

N-benzyl-1-cycloheptyl-N-methyl-3-phenylpropan-1-amine was prepared according to General Procedure **G** using Zn (20 mg, 0.3 mmol, 3 equiv), N-methylbenzylamine (13 µL, 0.1 mmol, 1 equiv), hydrocinnamaldehyde (26 µL, 0.2 mmol, 2 equiv), TMSOTf (32 µL, 0.175 mmol, 1.75 equiv) and 4,5,6,7-tetrachloro-1,3-dioxoisoindolin-2-yl cycloheptanecarboxylate (85 mg, 0.2 mmol, 2 equiv) in EtOAc (0.5 mL). The pure product was obtained as a colourless oil (21 mg, 63%).

^{1}H NMR (400 MHz, $CDCl_3$): δ 7.39 - 7.16 (m, 10H), 3.74 - 3.63 (m, 2H), 2.88 - 2.77 (m, 1H), 2.69 - 2.59 (m, 1H), 2.46 - 2.40 (m, 1H), 2.22 (s, 3H), 1.99 - 1.23 (m, 15H).

^{13}C NMR (101 MHz, $CDCl_3$): δ 143.4, 141.0, 128.7, 128.6, 128.5, 128.3, 126.8, 125.8, 68.2, 59.2, 40.9, 37.5, 35.3, 32.8, 31.2, 30.0, 28.8, 28.4, 27.4, 27.2.

IR (film, cm^{-1}): 3084, 3061, 3025, 2921, 2852, 2785, 1602, 1494, 1453, 1027, 733, 698.

HRMS: m/z calcd for $C_{24}H_{33}N$ $[M+H]^+$ 336.2686; found 336.2694.

N-benzyl-1-methoxy-N-methyl-5-phenylpentan-3-amine (16i)

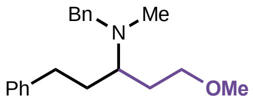

N-benzyl-1-methoxy-N-methyl-5-phenylpentan-3-amine was prepared according to General Procedure **G** using Zn (20 mg, 0.3 mmol, 3 equiv), N-methylbenzylamine (13 µL, 0.1 mmol, 1 equiv), hydrocinnamaldehyde (26 µL, 0.2 mmol, 2 equiv), TMSOTf (32 µL, 0.175 mmol, 1.75 equiv) and 4,5,6,7-tetrachloro-1,3-dioxoisoindolin-2-yl 3-methoxypropanoate (77 mg, 0.2 mmol, 2 equiv) in EtOAc (0.5 mL). The pure product was obtained as a colourless oil (19 mg, 62%).

^{1}H NMR (400 MHz, $CDCl_3$): δ 7.39 - 7.18 (m, 10H), 3.60 (s, 2H), 3.48 (t, J = 6.8 Hz, 2H), 3.35 (s, 3H), 2.82 - 2.63 (m, 3H), 2.19 (s, 3H), 1.97 - 1.84 (m, 2H), 1.71 - 1.57 (m, 2H).

^{13}C NMR (101 MHz, $CDCl_3$): δ 143.0, 140.6, 128.8, 128.54, 128.48, 128.3, 126.84, 125.82, 71.2, 59.5, 58.8, 58.1, 36.3, 33.6, 32.4, 29.9.

IR (film, cm^{-1}): 3061, 3025, 2929, 2860, 2804, 1602, 1494, 1453, 1117, 1026, 733, 698.

HRMS: m/z calcd for $C_{20}H_{27}NO$ $[M+H]^+$ 298.2166; found 298.2170.

N-benzyl-1-(benzyloxy)-N-methylbutan-2-amine (16j)

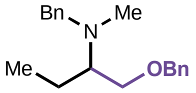

N-benzyl-1-(benzyloxy)-N-methylbutan-2-amine was prepared according to General Procedure **G** using Zn (20 mg, 0.3 mmol, 3 equiv), N-methylbenzylamine (13 µL, 0.1 mmol, 1 equiv), propionaldehyde (14 µL, 0.2 mmol, 2 equiv), TMSOTf (32 µL, 0.175 mmol, 1.75 equiv) and 4,5,6,7-tetrachloro-1,3-dioxoisoindolin-2-yl 2-(benzyloxy)acetate (90 mg, 0.2 mmol, 2 equiv) in EtOAc (0.5 mL). The crude product was subjected to an acid-base wash according to general procedure **ABW** followed by column chromatography (0-40% Et_2O in Pet. Ether) yielding the product as a colourless oil (14 mg, 48%).

^{1}H NMR (400 MHz, $CDCl_3$): δ 7.41 - 7.22 (m, 10H), 4.60 - 4.52 (m, 2H), 3.81-3.66 (m, 3H), 3.54 - 3.46 (m, 1H), 2.85 - 2.73 (m, 1H), 2.28 (s, 3H), 1.66 - 1.49 (m, 2H), 1.01 (t, *J* = 7.4 Hz, 3H).

^{13}C NMR (101 MHz, $CDCl_3$): δ 140.8, 138.8, 128.8, 128.5, 128.6, 127.7, 127.6, 126.8, 73.3, 70.5, 64.1, 58.8, 37.5, 22.0, 11.8.

IR (thin film, cm^{-1}): 3028, 2961, 2930, 2871, 2789, 1494, 1453, 1361, 1101, 1027, 733, 697.

HRMS: m/z calcd for $C_{19}H_{25}NO$ $[M+H]^+$ 284.2009; found 284.2014.

2-(2-(benzyl(methyl)amino)-4-phenylbutyl)isoindoline-1,3-dione (16k)

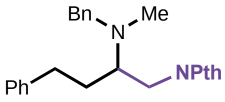

2-(2-(benzyl(methyl)amino)-4-phenylbutyl)isoindoline-1,3-dione was prepared according to General Procedure **G** using Zn (20 mg, 0.3 mmol, 3 equiv), N-methylbenzylamine (13 µL, 0.1 mmol, 1 equiv), propionaldehyde (14 µL, 0.2 mmol, 2 equiv), TMSOTf (32 µL, 0.175 mmol, 1.75 equiv) and 4,5,6,7-tetrachloro-1,3-dioxoisoindolin-2-yl 2-(1,3-dioxoisoindolin-2-yl)acetate (98 mg, 0.2 mmol, 2 equiv) in EtOAc (0.5 mL) with modifications. The acidic aqueous layer was basified with Na_2CO_3 (saturated, aq), instead of NaOH (10%, aq). The pure product was obtained as a colourless oil (29 mg, 74%).

^{1}H NMR (400 MHz, $CDCl_3$): δ 7.88 - 7.82 (m, 2H), 7.77 - 7.71 (m, 2H), 7.33 - 7.26(m, 2H), 7.25 - 7.17 (m, 3H), 7.15 - 7.00 (m, 5H), 3.92 (dd, J = 13.5 Hz, 8.9 Hz, 1H), 3.70 - 3.51 (m, 3H), 3.08 (quint, J = 7.1 Hz, 1H), 2.86 - 2.67 (m, 2H), 2.33 (s, 3H), 2.05 - 1.91 (m, 1H), 1.74 - 1.60 (m, 1H).

^{13}C NMR (101 MHz, $CDCl_3$): δ 168.5, 142.3, 139.9, 133.9, 132.4, 128.63, 128.54, 128.59, 128.1, 126.7, 126.0, 123.2, 59.2, 58.9, 38.5, 35.8, 33.5, 30.1.

IR (film, cm^{-1}): 3084, 6060, 3026, 2938, 2856, 2793, 1770, 1708, 1397, 722, 713, 699;

HRMS: m/z calcd for $C_{26}H_{26}N_2O_2$ $[M+H]^+$ 399.2068, found 399.2068.

N-benzyl-N-methyl-1-(methylthio)-4-phenylbutan-2-amine (16l)

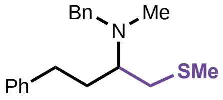

N-benzyl-N-methyl-1-(methylthio)-4-phenylbutan-2-amine was prepared according to General Procedure **G** using Zn (20 mg, 0.3 mmol, 3 equiv), N-methylbenzylamine (13 µL, 0.1 mmol, 1 equiv), propionaldehyde (14 µL, 0.2 mmol, 2 equiv), TMSOTf (32 µL, 0.175 mmol, 1.75 equiv) and 4,5,6,7-tetrachloro-1,3-dioxoisoindolin-2-yl 2-(methylthio)acetate (78 mg, 0.2 mmol, 2 equiv) in EtOAc (0.5 mL). The pure product was obtained as a colourless oil (22 mg, 74%).

^{1}H NMR (400 MHz, $CDCl_3$): δ 7.43 - 7.18 (m, 10H), 3.74 (d, J = 13.4 Hz, 1H), 3.61 (d, J = 13.4 Hz, 1H), 2.93 - 2.77 (m, 3H), 2.77 - 2.65 (m, 1H), 2.50 - 2.39 (m, 1H), 2.25 (s, 3H), 2.08 (s, 3H), 2.00 - 1.82 (m, 2H).

^{13}C NMR (101 MHz, $CDCl_3$): δ 142.7, 140.2, 128.8, 128.6, 128.58, 128.46, 127.0, 125.8, 61.6, 58.0, 36.3, 34.8, 33.3, 32.4, 16.4.

IR (film, cm^{-1}): 3083, 3061, 3025, 2914, 2849, 2787, 1602, 1493, 1453, 1358, 1319, 1124, 1074, 1027, 957, 824, 733, 58.

HRMS: m/z calcd for $C_{19}H_{25}NS$ $[M+H]^+$ 300.1781, found 300.1783.

N-benzyl-N-methyl-3-phenyl-1-(tetrahydrofuran-2-yl)propan-1-amine (16m)

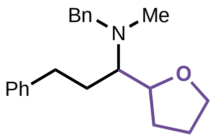

N-benzyl-N-methyl-3-phenyl-1-(tetrahydrofuran-2-yl)propan-1-amine was prepared according to General Procedure **G** using Zn (20 mg, 0.3 mmol, 3 equiv), N-methylbenzylamine (13 µL, 0.1 mmol, 1 equiv), propionaldehyde (14 µL, 0.2 mmol, 2 equiv), TMSOTf (32 µL, 0.175 mmol, 1.75 equiv) and 4,5,6,7-tetrachloro-1,3-dioxoisoindolin-2-yl tetrahydrofuran-2-carboxylate (80 mg, 0.2 mmol, 2 equiv) in EtOAc (0.5 mL). The pure product was obtained as a colourless oil (23 mg, 1.3:1 d.r., 73%)

For characterization, a pure sample of one of the diastereomers can be obtained by column chromatography (0-15% Et_2O in Pet. Ether).

^{1}H NMR (400 MHz, $CDCl_3$): δ 7.39 (d, J = 7.4 Hz, 2H), 7.33 - 7.16 (m, 8H), 4.01 (q, J = 7.1 Hz, 1H), 3.88 - 3.72 (m, 4H), 2.97 - 2.90 (m, 1H), 2.69 (ddd, J = 13.7, 9.6, 6.8 Hz, 1H), 2.62 - 2.57 (m, 1H), 2.32 (s, 3H), 1.93 - 1.71 (m, 4H), 1.64-1.53 (m, 2H).

^{13}C NMR (101 MHz, $CDCl_3$): δ 143.2, 141.4, 128.8, 128.6, 128.4, 128.2, 126.6, 125.8, 80.6, 67.8, 66.0, 59.9, 37.1, 33.6, 30.8, 30.1, 26.1.

IR (thin film, cm^{-1}): 3025, 2947, 2860, 1495, 1453, 1065, 1040, 1028, 734, 698.

HRMS: m/z calcd for $C_{21}H_{27}NO$ $[M+H]^+$ 310.2166; found 310.2171.

^{1}H Spectrum of diastereomeric mixture

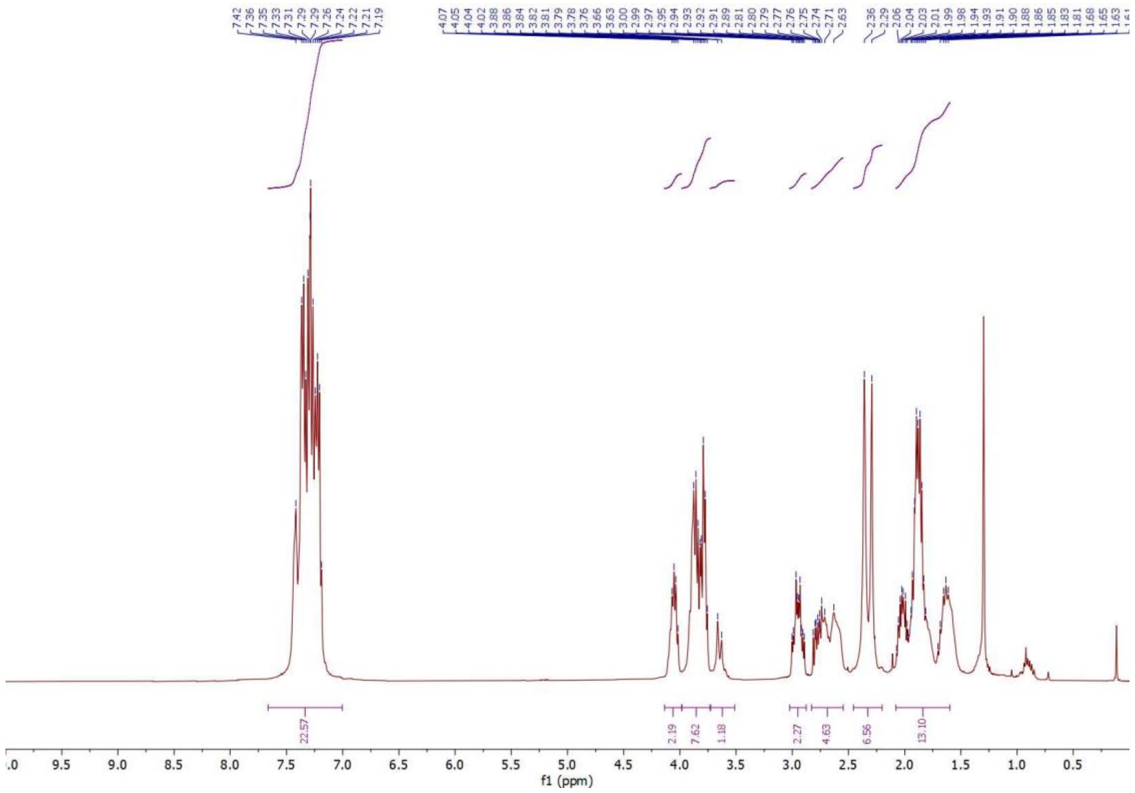

N-benzyl-4-fluoro-N,4-dimethyl-1-phenylpentan-3-amine (16n)

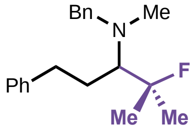

N-benzyl-4-fluoro-N,4-dimethyl-1-phenylpentan-3-amine was prepared according to General Procedure **G** using Zn (20 mg, 0.3 mmol, 3 equiv), N-methylbenzylamine (13 µL, 0.1 mmol, 1 equiv), propionaldehyde (14 µL, 0.2 mmol, 2 equiv), TMSOTf (32 µL, 0.175 mmol, 1.75 equiv) and 4,5,6,7-tetrachloro-1,3-dioxoisoindolin-2-yl 2-fluoro-2-methylpropanoate (78 mg, 0.2 mmol, 2 equiv) in EtOAc (0.5 mL). The pure product was obtained as a colourless oil (21 mg, 70%).

^{1}H NMR (400 MHz, $CDCl_3$): δ 7.35 - 7.28 (m, 6H), 3.89 (d, J = 13.9 Hz, 1H), 3.82 (d, J = 13.9 Hz, 1H), 2.94 - 2.82 (m, 1H), 2.76 - 2.64 (m, 2H), 2.36 (s, 3H), 2.06 - 1.86 (m, 2H), 1.43 (d, J = 2.0, 3H), 1.38 (J = d, 1.2, 3H).

^{13}C NMR (101 MHz, $CDCl_3$): δ 142.7, 140.8, 128.6, 128.4, 126.9, 126.0, 100.0 (d, 170.1.0 Hz), 69.5 (d, 22.4 Hz), 60.9, 38.2, 35.2, 28.3 (d, 2.8 Hz), 26.8 (d, 24.2 Hz), 25.4 (d, 24.8 Hz).

^{19}F NMR (375 MHz, $CDCl_3$): δ -142.0

IR (film, cm^{-1}): 3084, 3062, 3026, 2978, 2935, 2793, 1602, 1495, 1453, 1369, 1028, 734, 698.

HRMS: m/z calcd for $C_{20}H_{26}FN$ $[M+H]^+$ 300.2123; found 300.2128.

9. Synthesis of α-tertiary amines

9.1 Reaction optimization

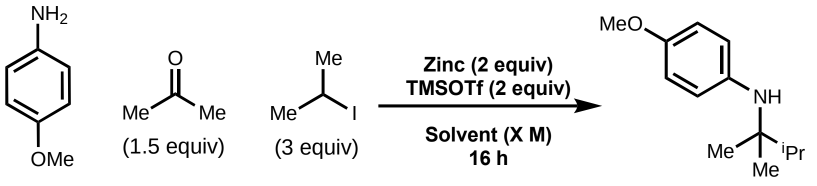

1b 2b 3a 17a

Entry	1b / 2b / 3a (equiv)	Reductant	Additive	Solvent	[Conc]	Yield[a] / %
1	1.0 / 1.5 / 3.0	Zn (2.0 equiv)	-	EtOAc	0.4 M	9
2	1.0 / 1.5 / 3.0	Zn (2.0 equiv)	-	CH_2Cl_2	0.4 M	53
3	1.0 / 1.5 / 3.0	Zn (2.0 equiv)	-	Toluene	0.4 M	14
4	1.0 / 1.5 / 3.0	Zn (2.0 equiv)	CuI (10 mol%)	CH_2Cl_2	0.4 M	41
5	1.0 / 1.5 / 3.0	Zn (2.0 equiv)	CuI (10 mol%)	Toluene	0.4 M	59
6	1.0 / 1.5 / 3.0	Zn (2.0 equiv)	CuI (10 mol%)	CH_2Cl_2	0.8 M	51
7	1.0 / 1.5 / 3.0	Zn (2.0 equiv)	CuI (10 mol%)	Toluene	0.8 M	51
8	1.0 / 1.5 / 3.0	Zn (2.0 equiv)	CuI (10 mol%)	Toluene	1.6 M	71
9	1.0 / 1.5 / 3.0	Zn (3.0 equiv)	CuI (10 mol%)	Toluene	1.6 M	73
10	1.0 / 1.5 / 3.0	Zn (3.0 equiv)	CuI (10 mol%), H_2O (1.5 equiv)	CH_2Cl_2	1.6 M	72
11	1.0 / 1.5 / 3.0	Zn (3.0 equiv)	CuI (10 mol%), H_2O (1.5 equiv)	Toluene	1.6 M	93
12[b]	1.0 / 1.5 / 3.0	Zn (3.0 equiv)	CuI (10 mol%), H_2O (1.5 equiv)	Toluene	1.6 M	100

[a] Each 0.4 mmol reaction crude was worked up according to general procedure A and yields were determined by 1H NMR using 1,1,2,2-tetrachloroethane as internal standard. [b] Reaction run for 24 h.

9.2 Amino-oxetane reaction development

9.2.1 Reaction optimization

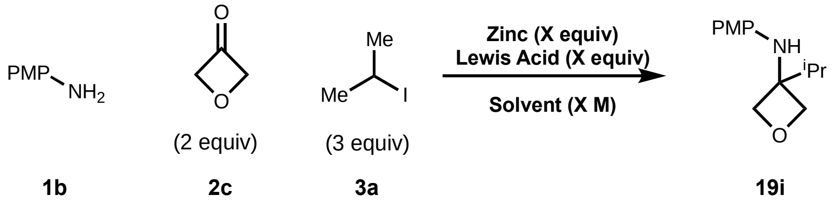

Entry	1b / 2c / 3a (equiv)	Reductant	TMSOTf / equiv	DCM / mL	HFIP / mL	CuI (mol%)	Yield[a] / %
1	1.0 / 1.5 / 3.0	Zn (3.0 equiv)	3.0	0.25	0.00	40	15
2	1.0 / 1.5 / 3.0	Zn (3.0 equiv)	3.0	0.50	0.00	40	44
3	1.0 / 1.5 / 3.0	Zn (3.0 equiv)	3.0	1.00	0.00	40	15
4	1.0 / 1.5 / 3.0	Zn (3.0 equiv)	3.0	0.50	0.05	40	49
5	1.0 / 1.5 / 3.0	Zn (3.0 equiv)	3.0	0.50	0.10	40	58
6	1.0 / 1.5 / 3.0	Zn (3.0 equiv)	3.0	0.50	0.15	40	44
7	1.0 / 2.0 / 4.0	Zn (3.0 equiv)	1.5	0.40	0.20	40	40
8[b]	1.0 / 2.0 / 4.0	Zn (3.0 equiv)	1.5	0.40	0.20	40	26
9	1.0 / 2.0 / 4.0	Zn (3.0 equiv)	1.5	0.20	0.10	40	40
10	1.0 / 2.0 / 4.0	Zn (3.0 equiv)	1.5	0.40	0.20	100	47
11	1.0 / 2.0 / 4.0	Zn (3.0 equiv)	1.5	0.50	0.10	50	73
12	1.0 / 2.0 / 4.0	Zn (3.0 equiv)	1.5	0.25	0.05	50	68
13	1.0 / 2.0 / 3.0	Zn (3.0 equiv)	1.5	0.50	0.10	50	57
14	1.0 / 2.0 / 4.0	Zn (3.0 equiv)	1.5	0.60	0.10	50	37
15	1.0 / 2.0 / 4.0	Zn (3.0 equiv)	1.25	0.50	0.10	50	51
16	1.0 / 2.5 / 4.0	Zn (3.0 equiv)	1.5	0.50	0.10	50	60
17	1.0 / 2.0 / 5.0	Zn (3.0 equiv)	1.5	0.50	0.10	50	44

18	1.0 / 2.5 / 4.0	Zn (3.0 equiv)	2.0	0.50	0.10	50	77
19	1.0 / 2.5 / 3.0	Zn (3.0 equiv)	2.0	0.50	0.10	50	68
20	1.0 / 2.0 / 4.0	Zn (3.0 equiv)	2.0	0.50	0.10	50	64
21	1.0 / 2.0 / 4.0	Zn (3.0 equiv)	2.5	0.50	0.10	50	38
22	1.0 / 2.0 / 4.0	Zn (3.0 equiv)	3.0	0.50	0.10	50	11
23	1.0 / 2.0 / 4.0	Zn (3.0 equiv)	2.0	0.50	0.10	50	36

[a] Each 0.4 mmol reaction crude was worked up according to general procedure A and yields were determined by ^1H NMR using 1,1,2,2-tetrachloroethane as internal standard. [b] HCl salt of amine used.

9.2.2 Amino-oxetane time course study

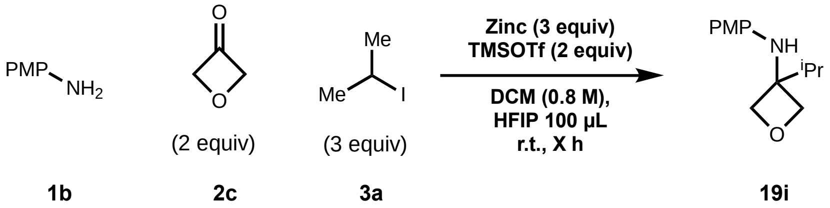

Time studies were set up according to General Procedure **J** using Zn (79 mg, 1.2 mmol, 3 equiv), *p*-anisidine (49 mg, 0.4 mmol, 1 equiv), copper iodide (38 mg, 0.2 mmol, 50 mol%), oxetan-3-one (59 μL, 1.0 mmol, 2.5 equiv), 2-iodopropane (160 μL, 1.6 mmol, 4.0 equiv) and TMSOTf (138 μL, 0.8 mmol, 2.0 equiv). Each 0.4 mmol reaction crude was worked up and yields were determined by ^{1}H NMR using 1,1,2,2-tetrachloroethane as internal standard. Each time point was run in duplicate to give average yields.

Time/ h	Run 1 /%	Run 2 /%	Average /%
1	18	11	14.55
3	31	28	29.5
6	30	31	30.4
8	28	31	29.9
12	44	39	41.1
14	60	62	60.55
16	40	43	41.5
24	33	36	34.85

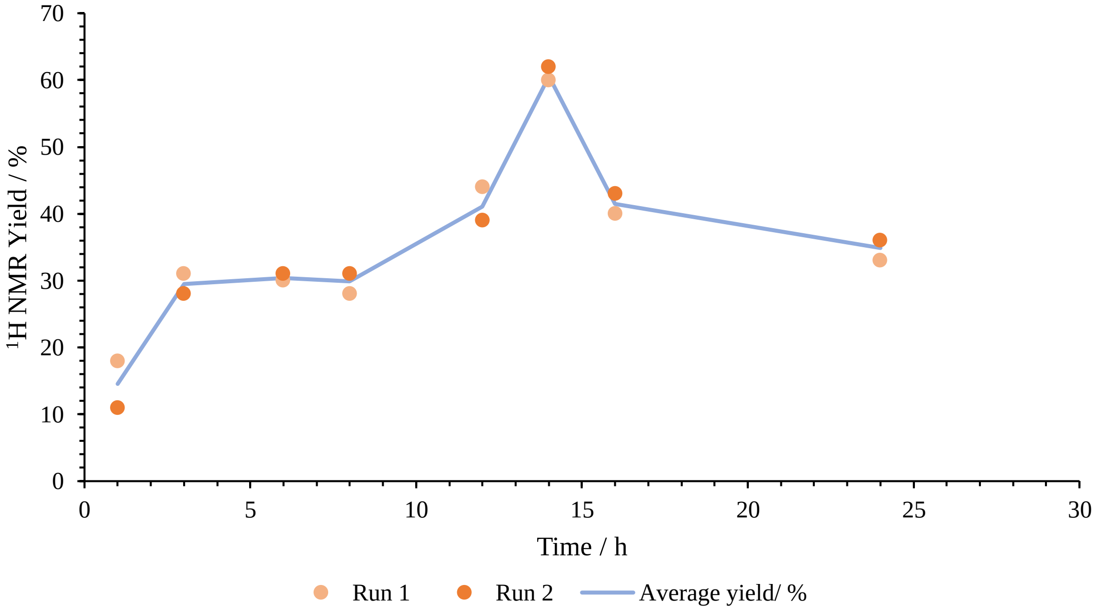

9.2.3 Product degradation study

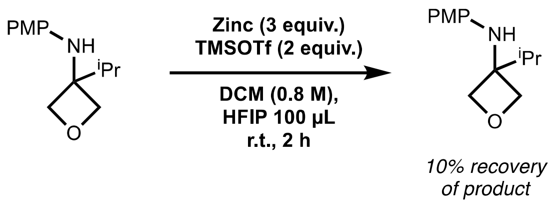

An oven dried vial (Biotage Microwave Process Vial 10 mL, equipped with gas-tight pierceable caps) was charged with a stirring bar, amine (0.4 mmol), Zn dust (79 mg, 1.2 mmol, 3.0 equiv) and copper iodide (38 mg, 0.2 mmol, 50 mol%). The vial was sealed, evacuated, and backfilled with N_2. To this was added 0.5 µl anhydrous dichloromethane. While stirring, HFIP (100 µL) followed by TMSOTf (138 µL, 0.8 mmol, 2.0 equiv) was added. The mixture was stirred for 2 h under a N_2 atm. The mixture was diluted with dichloromethane and basified with NaOH (10%, aq). The heterogenous mixture was stirred vigorously for 15 mins. The organic and aqueous layers were separated. The aqueous layer was extracted with dichloromethane twice. The combined organic layer was dried over Na_2SO_4. Solvent was removed *in vacuo*. Yield of the product remaining was determined by ^{1}H NMR using 1,1,2,2-tetrachloroethane as internal standard indicating a 10% recovery.

9.3 Synthesis of α-tertiary amines: general procedures

9.3.1 General procedure H (Ketone scope-non-alkylamines)

An oven dried vial (Biotage Microwave Process Vial 10 mL, equipped with gas-tight pierceable caps) was charged with a stirring bar, the aniline (0.4 mmol, 1 equiv), Zn dust (79 mg, 1.2 mmol, 3 equiv) and copper iodide (7.5 mg, 0.04 mmol, 10 mol%). The vial was sealed, evacuated and backfilled with N_2. To this was added 0.25 mL dry dichloromethane or toluene. While stirring, the ketone (0.6 mmol, 1.5 equiv) was added dropwise, followed by the alkyl iodide (1.2 mmol, 3 equiv). H_2O (11 µL, 0.6 mmol, 1.5 equiv) and then TMSOTf (109 µL, 0.6 mmol, 1.5 equiv) were added. The mixture was stirred overnight. The mixture was diluted with dichloromethane and basified with NaOH (10%, aq). The heterogenous mixture was stirred vigorously for 15 min. The organic and aqueous layers were separated. The aqueous layer was extracted with dichloromethane twice. The combined organic layer was dried over Na_2SO_4. Solvent was removed under reduced pressure. The crude mixture was purified by column chromatography.

9.3.2 General procedure I (Ketone scope -alkylamines)

An oven dried vial (Biotage Microwave Process Vial 10 mL, equipped with gas-tight pierceable caps) was charged with a stirring bar, the amine HCl salt (0.4 mmol, 1 equiv), Zn dust (79 mg, 1.2 mmol, 3 equiv) and copper iodide (7.5 mg, 0.04 mmol, 10 mol%). The vial was sealed, evacuated and backfilled with N_2. To this was added 0.25 mL dry dichloromethane or toluene. While stirring, the ketone (0.6 mmol, 1.5 equiv) was added dropwise, followed by the alkyl iodide (1.2 mmol, 3 equiv). Then H_2O (11 µL, 0.6 mmol, 1.5 equiv) and TMSOTf (109 µL, 0.6 mmol, 1.5 equiv) were added. The mixture was stirred overnight. The mixture was diluted with dichloromethane and basified with NaOH (10%, aq). The heterogenous mixture was stirred vigorously for 15 mins. The organic and aqueous layers were separated. The aqueous layer was extracted with dichloromethane twice. The combined organic layer was dried over Na_2SO_4. Solvent was removed under reduced pressure. The crude mixture was purified by column chromatography.

9.3.3 General procedure J (Oxetane scope)

An oven dried vial (Biotage Microwave Process Vial 10 mL, equipped with gas-tight pierceable caps) was charged with a stirring bar, the aniline (2 mmol, 1.0 equiv), Zn dust (3 mmol, 3.0 equiv) and copper iodide (1 mmol, 50 mol%). The vial was sealed, evacuated, and backfilled with N_2. To this was added 2.5 mL anhydrous dichloromethane. While stirring, HFIP (500 µL) then oxetan-3-one (5.0 mmol, 2.5 equiv) was added. The reaction mixture was stirred for 30 minutes at 1100 rpm. Alkyl iodide (8 mmol, 4.0 equiv) was added, followed by TMSOTf (4.0 mmol, 2.0 equiv). The mixture was stirred for 14 h under a N_2 atm. The mixture was diluted with dichloromethane and basified with NaOH (10%, aq). The heterogenous mixture was stirred vigorously for 15 mins. The organic and aqueous layers were separated. The aqueous layer was extracted with dichloromethane twice. The combined organic layer was dried over Na_2SO_4. Solvent was removed *in vacuo*. The crude mixture was purified by column chromatography using alumina or reverse phase column chromatography.

9.4 Synthesis of α-tertiary amines: reaction scope

N-(2,3-dimethylbutan-2-yl)-4-methoxyaniline (17a)

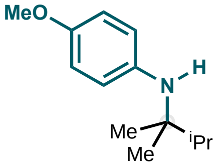

N-(2,3-dimethylbutan-2-yl)-4-methoxyaniline was prepared according to General Procedure **H** using Zn (79 mg, 1.2 mmol, 3 equiv), *p*-anisidine (49 mg, 0.4 mmol, 1 equiv), copper iodide (7.5 mg, 0.04 mmol, 10 mol%), acetone (44 µL, 0.6 mmol, 1.5 equiv) and 2-iodopropane (120 µL, 1.2 mmol, 3 equiv) in toluene (0.25 mL). Crude material was purified by column chromatography (20% dichloromethane in Pet. Ether (with 4% NEt_3)) yielding the product as a brown oil (72 mg, 87%).

^{1}H NMR (400 MHz, $CDCl_3$): δ 6.80 - 6.62 (m, 4H), 3.75 (s, 3H), 2.96 (brs, 1H), 1.91 (sept, J = 6.8 Hz, 1H), 1.15 (s, 6H), 0.94 (d, J = 6.8 Hz, 6H).

^{13}C NMR (100 MHz, $CDCl_3$): δ 153.7, 140.2, 121.6, 114.3, 57.2, 55.7, 36.2, 25.1, 17.8.

IR (film, cm^{-1}): 3403 (br), 2960, 2875, 2832, 1507, 1464, 1236, 1039, 818.

HRMS: m/z calcd for $C_{13}H_{21}NO$ $[M+H]^+$ 208.1696, found 208.1701.

N-(2,3-dimethylbutan-2-yl)aniline (17b)

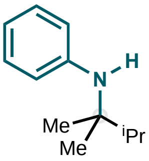

N-(2,3-dimethylbutan-2-yl)aniline was prepared according to General Procedure **H** using Zn (79 mg, 1.2 mmol, 3 equiv), aniline (37 mg, 0.4 mmol, 1 equiv), copper iodide (7.5 mg, 0.04 mmol, 10 mol%), acetone (44 µL, 0.6 mmol, 1.5 equiv) and 2-iodopropane (120 µL, 1.2 mmol, 3 equiv) in toluene (0.25 mL). Crude material was purified by column chromatography (10% dichloromethane in Pet. Ether (with 4% NEt_3)) yielding the product as a brown oil (58 mg, 82%)

^{1}H NMR (400 MHz, $CDCl_3$): δ 7.17 - 7.10 (m, 2H) 7.76 - 7.69 (m, 3H), 3.44 (brs, 1H), 2.13 (sept, J = 6.8 Hz, 1H), 1.26 (s, 6H), 0.94 (d, J = 6.8 Hz, 6H).

^{13}C NMR (100 MHz, $CDCl_3$): δ 146.9, 129.1, 117.9, 117.1, 56.8, 35.7, 25.1, 17.7.

IR (film, cm^{-1}): 3412 (br), 3053, 2970, 2875, 1600, 1496, 1468, 1428, 1391, 1378, 1367, 1332, 1312, 1259, 1196, 1179, 1147, 1105, 1081, 1032, 747, 692.

HRMS: m/z calcd for $C_{12}H_{19}N$ $[M+H]^+$ 178.1591; found 178.1591.

4-bromo-N-(2,3-dimethylbutan-2-yl)aniline (17c)

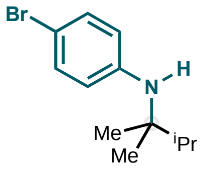

4-bromo-N-(2,3-dimethylbutan-2-yl)aniline was prepared according to General Procedure **H** using Zn (79 mg, 1.2 mmol, 3 equiv), 4-bromoaniline (69 mg, 0.4 mmol, 1 equiv), copper iodide (7.5 mg, 0.04 mmol, 10 mol%), acetone (44 µL, 0.6 mmol, 1.5 equiv) and 2-iodopropane (120 µL, 1.2 mmol, 3 equiv) in toluene (0.25 mL). Crude material was purified by column chromatography (10% dichloromethane in Pet. Ether (4% NEt_3)) yielding the product as a brown oil (95 mg, 93%).

^{1}H NMR (400 MHz, $CDCl_3$): δ 7.20 (d, J = 8.0 Hz, 2H), 6.59 (d, J = 7.9 Hz, 2H), 3.75 - 3.14 (brs, 1H), 2.10 (sept, J = 6.6 Hz, 1H), 1.24 (s, 6H), 0.92 (d, J = 6.6 Hz, 6H).

^{13}C NMR (100 MHz, $CDCl_3$): δ 146.1, 131.8, 118.2, 109.4, 56.8, 35.5, 25.0, 17.7.

IR (film, cm^{-1}): 3416 (br), 2969, 2875, 1589, 1488, 1314, 1177, 810.

HRMS: m/z calcd for $C_{12}H_{18}BrN$ $[M+H]^+$ 256.0696, 258.0675; found 256.0698, 258.0678.

N-(2,3-dimethylbutan-2-yl)-4-iodoaniline (17d)

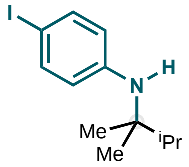

N-(2,3-dimethylbutan-2-yl)-4-iodoaniline was prepared according to General Procedure **H** using Zn (79 mg, 1.2 mmol, 3 equiv), 4-iodoaniline (88 mg, 0.4 mmol, 1 equiv), copper iodide (7.5 mg, 0.04 mmol, 10 mol%), acetone (44 µL, 0.6 mmol, 1.5 equiv) and 2-iodopropane (120 µL, 1.2 mmol, 3 equiv) in toluene (0.25 mL). Crude material was purified by column chromatography (4% dichloromethane in Pet. Ether (with 4% NEt_3)) yielding the product as a brown oil (117 mg, 96%).

^{1}H NMR (400 MHz, $CDCl_3$): δ 7.38 (d, J = 7.8 Hz, 2H), 6.50 (d, J = 7.9 Hz, 2H), 3.70 - 3.24 (brs, 1H), 2.12 (sept, J = 6.8 Hz, 1H), 1.25 (s 6H), 0.93 (d, J = 6.7 Hz, 6H).

^{13}C NMR (100 MHz, $CDCl_3$): δ 146.6, 137.6, 118.5, 78.2, 56.8, 35.3, 24.9, 17.6.

IR (film, cm^{-1}): 3412 (br), 2966, 2874, 1587, 1484, 1392, 1314, 1295, 1256, 1179, 808.

HRMS: m/z calcd for $C_{12}H_{18}IN$ $[M+H]^+$ 304.0557; found 304.0563.

Ethyl 3-((2,3-dimethylbutan-2-yl)amino)benzoate (17e)

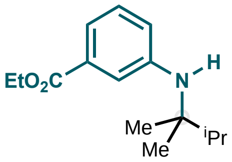

Ethyl 3-((2,3-dimethylbutan-2-yl)amino)benzoate was prepared according to General Procedure **H** using Zn (79 mg, 1.2 mmol, 3 equiv), ethyl 3-aminobenzoate (60 µL, 0.4 mmol, 1 equiv), copper iodide (7.5 mg, 0.04 mmol, 10 mol%), acetone (44 µL, 0.6 mmol, 1.5 equiv) and 2-iodopropane (120 µL, 1.2 mmol, 3 equiv) in toluene (0.25 mL). Crude material was purified by column chromatography (8 % dichloromethane in Pet. Ether (with 4% NEt_3)) yielding the product as a brown oil (85 mg, 85%).

^{1}H NMR (400 MHz, $CDCl_3$): δ 7.39 - 7.32 (m, 2H), 7.17 (t, J = 7.5 Hz, 1H), 6.88 (d, J = 8.3 Hz, 1H), 4.34 (q, J = 6.6 Hz, 2H), 3.70 - 3.58 (brs, 1H), 2.14 (sept, J = 6.8 Hz, 1H), 1.38 (t, J = 6.8 Hz, 3H), 1.27 (s, 6H), 0.93 (d, J = 6.8 Hz, 6H).

^{13}C NMR (100 MHz, $CDCl_3$): δ 167.2, 147.1, 131.2, 128.9, 120.5, 118.6, 117.2, 60.9, 56.8, 35.5, 25.0, 17.7, 14.5.

IR (film, cm^{-1}): 3398 (br), 2973, 2875, 1709, 1603, 1586, 1480, 1430, 1392, 1367, 1342, 1277, 1239, 1198, 1147, 1105, 1081, 1027, 753.

HRMS: m/z calcd for $C_{15}H_{23}NO_2$ $[M+H]^+$ 250.1802; found 250.1806.

3-bromo-N-(2,3-dimethylbutan-2-yl)aniline (17f)

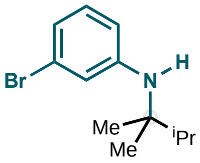

3-bromo-N-(2,3-dimethylbutan-2-yl)aniline was prepared according to General Procedure **H** using Zn (79 mg, 1.2 mmol, 3 equiv), 3-bromoaniline (44 µL, 0.4 mmol, 1 equiv), copper iodide (7.5 mg, 0.04 mmol, 10 mol%), acetone (44 µL, 0.6 mmol, 1.5 equiv) and 2-iodopropane (120 µL, 1.2 mmol, 3 equiv) in toluene (0.25 mL). Crude material was purified by column chromatography (10% dichloromethane in Pet. Ether (with 4% NEt_3)) yielding the product as a brown oil (92 mg, 90%).

^{1}H NMR (700 MHz, $CDCl_3$): δ 6.97 (t, J = 8.0 Hz, 1H), 6.84 (s, 1H), 6.79 (d, J = 7.8 Hz, 1H), 6.60 (d, J = 7.9 Hz, 1H), 3.67-3.48 (brs, 1H), 2.13 (sept, J = 6.8 Hz, 1H), 1.25 (s, 6H), 0.92 (d, J = 6.9 Hz, 6H).

^{13}C NMR (175 MHz, $CDCl_3$): δ 148.4, 130.3, 123.0, 120.1, 118.5, 114.7, 56.8, 35.4, 25.0, 17.7.

IR (film, cm^{-1}): 3416 (br), 2970, 2875, 1591, 1574, 1504, 1478, 1392, 1196, 986, 760, 683.

HRMS: m/z calcd for $C_{12}H_{18}BrN$ $[M+H]^+$ 256.0696, 258.0675; found 256.0697, 258.0677.

N-(2,3-dimethylbutan-2-yl)-2-methylaniline (17g)

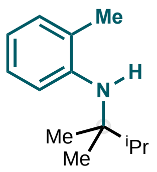

N-(2,3-dimethylbutan-2-yl)-2-methylaniline was prepared according to General Procedure **H** using Zn (79 mg, 1.2 mmol, 3 equiv), toluidine (43 µL, 0.4 mmol, 1 equiv), copper iodide (7.5 mg, 0.04 mmol, 10 mol%), acetone (44 µL, 0.6 mmol, 1.5 equiv) and 2-iodopropane (120 µL, 1.2 mmol, 3 equiv) in toluene (0.25 mL). Crude material was purified by column chromatography (2% dichloromethane in Pet. Ether (with 4% NEt_3)) yielding the product as a brown oil (38 mg, 50%).

^{1}H NMR (400 MHz, $CDCl_3$): δ 7.11 - 7.02 (m, 2H), 6.90 (d, J = 8.2 Hz, 1H), 6.63 (t, J = 7.2 Hz, 1H), 3.62 - 3.16 (brs, 1H), 2.25 (sept, J = 6.7 Hz, 1H), 2.15 (s, 3H), 1.33 (s, 6H), 0.96 (d, J = 6.7 Hz, 6H).

^{13}C NMR (100 MHz, $CDCl_3$): δ 145.0, 130.6, 126.7, 123.1, 116.6, 113.5, 56.4, 35.7, 25.2, 18.3, 17.7.

IR (film, cm^{-1}): 3448 (br), 2962, 2874, 1605, 1586, 1480, 1444, 1391, 1378, 1367, 1338, 1313, 1262, 1201, 1180, 1147, 1052, 907, 742.

HRMS: m/z calcd for $C_{13}H_{21}N$ $[M+H]^+$ 192.1747, found 192.1749.

methyl 4-((2,3-dimethylbutan-2-yl)amino)-2-methoxybenzoate (17h)

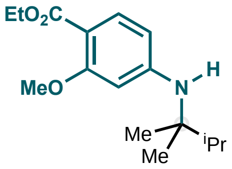

methyl 4-((2,3-dimethylbutan-2-yl)amino)-2-methoxybenzoate was prepared according to General Procedure **H** using Zn (79 mg, 1.2 mmol, 3 equiv), methyl 4-amino-2-methoxybenzoate (73 mg, 0.4 mmol, 1 equiv), copper iodide (7.5 mg, 0.04 mmol, 10 mol%), acetone (44 µL, 0.6 mmol, 1.5 equiv) and 2-iodopropane (120 µL, 1.2 mmol, 3 equiv) in toluene (0.25 mL). Crude material was purified by column chromatography (0-40% Et_2O in Pet. Ether) yielding the product as an amorphous solid (45 mg, 42%).

^{1}H NMR (700 MHz, $CDCl_3$): δ 7.70 (d, J = 8.8 Hz, 1H), 6.23 (dd, J = 8.7, 2.1 Hz, 1H), 6.14 (d, J = 2.3 Hz, 1H), 4.05 (brs, 1H), 3.86 (s, 3H), 3.81 (s, 3H), 2.22 (sept, J = 6.8 Hz, 1H), 1.33 (s, 6H), 0.96 (d, J = 6.6 Hz, 6H).
^{13}C NMR (176 MHz, $CDCl_3$): δ 166.3, 161.5, 152.2, 133.8, 107.1, 106.4, 97.7, 56.84, 55.77, 51.3, 35.4, 24.8, 17.6.
IR (solid, cm^{-1}): 3366, 2920, 2245, 1695, 1608, 1462, 1362, 1258, 1233, 1175, 1104, 1029, 912, 820, 762, 712, 658, 554, 479.

HRMS: m/z calcd for $C_{15}H_{23}NO_3$ $[M+H]^+$ 266.1751, found 266.1753.

Benzyl 4-isopropyl-4-(phenethylamino)piperidine-1-carboxylate (18a)

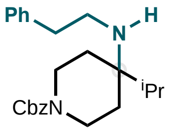

Benzyl 4-isopropyl-4-(phenethylamino)piperidine-1-carboxylate was prepared according to General Procedure **I** using Zn (79 mg, 1.2 mmol, 3 equiv), 2-phenylethan-1-amine hydrochloride (63 mg, 0.4 mmol, 1 equiv), copper iodide (7.5 mg, 0.04 mmol, 10 mol%), benzyl 4-oxopiperidine-1-carboxylate (140 mg, 0.6 mmol, 1.5 equiv) and 2-iodopropane (120 µL, 1.2 mmol, 3 equiv) in dichloromethane (0.25 mL). Crude material was purified column chromatography (1–5% MeOH in dichloromethane (with 5% NEt_3)) yielding the product (102 mg, 67%) as a pale-yellow oil.

^{1}H NMR δ 7.41 – 7.37 (m, 4H), 7.35 – 7.28 (m, 3H), 7.25 – 7.21 (m, 3H), 5.15 (s, 2H), 4.00 – 3.75 (m, 2H), 3.23 – 3.03 (m, 2H), 2.78 – 2.64 (m, 4H), 1.81 (sept, J = 6.8 Hz, 1H), 1.57 – 1.46 (m, 2H), 1.40 – 1.24 (m, 2H), 0.78 (d, J = 6.9 Hz, 6H).

^{13}C NMR (126 MHz, $CDCl_3$): δ 155.4, 140.4, 137.1, 128.8, 128.44, 128.39, 127.9, 127.8, 126.2, 66.8, 41.7, 39.5, 37.4, 32.5, 30.0, 16.6.

IR (film, cm^{-1}): 2955, 2875, 1692, 1426, 1227, 1111, 751, 697.

HRMS: m/z calcd for $C_{24}H_{33}N_2O_2$ $[M+H]^+$ 381.2537; found 381.2536.

Benzyl 4-((2-fluoroethyl)amino)-4-isopropylpiperidine-1-carboxylate (18b)

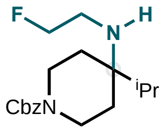

Benzyl 4-isopropyl-4-((2-(thiophen-2-yl)ethyl)amino)piperidine-1-carboxylate was prepared according to General Procedure **I** using Zn (79 mg, 1.2 mmol, 2-fluoroethan-1-amine hydrochloride (40 mg, 0.4 mmol, 1 equiv), copper iodide (7.5 mg, 0.04 mmol, 10 mol%), benzyl 4-oxopiperidine-1-carboxylate (140 mg, 0.6 mmol, 1.5 equiv) and 2-iodopropane (120 µL, 1.2 mmol, 3 equiv) in 0 dichloromethane (0.25 mL). Crude material was purified column chromatography (5–20% EtOAc in Pet. Ether (with 5% NEt_3)) yielding the product as a colourless oil (80 mg, 62%).

^{1}H NMR (500 MHz, $CDCl_3$) δ 7.38 – 7.33 (m, 4H), 7.32 – 7.28 (m, 1H), 5.12 (s, 2H), 4.59 – 4.44 (m, 2H), 3.95 – 3.81 (m, 2H), 3.25 – 3.13 (m, 2H), 2.72 (dt, J = 27.9, 4.8 Hz, 2H), 1.79 (sept, J = 6.9 Hz, 1H), 1.56 (s, 2H), 1.30 (d, J = 19.0 Hz, 2H), 1.13 (s, 1H), 0.85 (d, J = 6.9 Hz, 6H).

^{13}C NMR 126 MHz, $CDCl_3$) δ 155.4, 137.1, 128.5, 127.9, 127.8, 84.6 (d, *J* = 165.3 Hz), 66.9, 54.0, 40.6 (d, *J* = 19.8 Hz), 39.5, 32.7, 29.9, 16.6.

^{19}F NMR (471 MHz, $CDCl_3$) δ -224.2.

IR (film, cm^{-1}): 3340, 2955, 2877, 1691, 1427, 1241, 1111, 1034, 746, 697.

HRMS: m/z calcd for $C_{18}H_{27}FN_2NaO_2$ $[M+Na]^+$ 345.1949; found 345.1948.

Benzyl 4-((2-chloroethyl)amino)-4-isopropylpiperidine-1-carboxylate (18c)

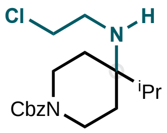

Benzyl 4-isopropyl-4-((2-(thiophen-2-yl)ethyl)amino)piperidine-1-carboxylate was prepared according to General Procedure **I** using Zn (79 mg, 1.2 mmol), 2-chloroethan-1-amine hydrochloride (46 mg, 0.4 mmol, 1 equiv), copper iodide (7.5 mg, 0.04 mmol, 10 mol%), benzyl 4-oxopiperidine-1-carboxylate (140 mg, 0.6 mmol, 1.5 equiv) and 2-iodopropane (120 µL, 1.2 mmol, 3 equiv) in dichloromethane (0.25 mL). Crude material was purified column chromatography (0-30% EtOAc in Pet. Ether), yielding the product as an amorphous solid (50 mg, 37%)

^{1}H NMR (400 MHz, $CDCl_3$): δ 7.41 - 7.30 (m, 5H), 5.15 (s, 2H), 4.00 - 3.84 (m, 2H), 3.65 (t, J = 11.3 Hz, 2H), 3.28 - 3.15 (m, 2H), 2.82 (t, J = 11.3 Hz, 2H), 1.80 (sept, J = 6.9 Hz, 1H), 1.67-1.50 (m, 2H), 1.46 – 1.15 (m, 2H), 0.89 (d, J = 6.9 Hz, 6H).

^{13}C NMR (100 MHz, $CDCl_3$): δ 155.4, 137.1, 128.5 127.9, 127.8, 66.9, 54.2, 46.2, 42.1, 39.5, 32.9, 30.1, 16.6.
IR (solid, cm^{-1}): 3375, 2979, 2833, 1695, 1583, 1525, 1441, 1345, 1295, 1208, 1175, 1075, 1037, 895, 812, 775, 704.

HRMS: m/z calcd for $C_{18}H_{27}ClN_2O_2[M+H]^+$ 339.1834, found 339.1831.

Benzyl 4-isopropyl-4-(methylamino)piperidine-1-carboxylate (18d)

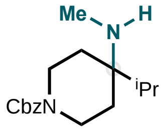

Benzyl 4-isopropyl-4-(phenethylamino)piperidine-1-carboxylate was prepared according to General Procedure **I** using Zn (79 mg, 1.2 mmol, 3 equiv), methylamine hydrochloride (27 mg, 0.4 mmol, 1 equiv), copper iodide (7.5 mg, 0.04 mmol, 10 mol%), benzyl 4-oxopiperidine-1-carboxylate (140 mg, 0.6 mmol, 1.5 equiv) and 2-iodopropane (120 µL, 1.2 mmol, 3 equiv) in dichloromethane (0.25 mL). Crude material was purified column chromatography (1–5% MeOH in dichloromethane (with 5% NEt_3)) yielding the product (72 mg, 62%) as a colourless oil.

^{1}H NMR (500 MHz, CDCl3) δ 7.40 – 7.35 (m, 4H), 7.34 – 7.30 (m, 1H), 5.14 (s, 2H), 4.00 – 3.83 (m, 2H), 3.26 – 3.12 (m, 2H), 2.23 (s, 3H), 1.88 (sept, J = 6.9 Hz, 1H), 1.64 – 1.49 (m, 2H), 1.44 – 1.26 (m, 2H), 1.02 (brs, 1H), 0.86 (d, J = 6.9 Hz, 6H).

^{13}C NMR (126 MHz, $CDCl_3$): δ 155.3, 137.0, 128.5, 127.9, 127.8, 66.9, 54.6, 39.2, 33.6, 30.2, 30.0, 16.6.

IR (film, cm^{-1}): 2954, 1690, 1426, 1277, 1229, 1104, 1028, 731, 696.

HRMS: m/z calcd for $C_{17}H_{26}N_2NaO_2$ $[M+Na]^+$ 313.1887; found 313.1893.

Benzyl 4-(benzylamino)-4-isopropylpiperidine-1-carboxylate (18e)

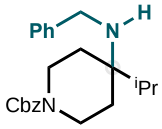

Benzyl 4-(benzylamino)-4-isopropylpiperidine-1-carboxylate was prepared according to General Procedure **I** using Zn (79 mg, 1.2 mmol, 3 equiv), benzylamine hydrochloride (57 mg, 0.4 mmol, 1 equiv), copper iodide (7.5 mg, 0.04 mmol, 10 mol%), benzyl 4-oxopiperidine-1-carboxylate (140 mg, 0.6 mmol, 1.5 equiv) and 2-iodopropane (120 µL, 1.2 mmol, 3 equiv) in dichloromethane (0.25 mL). Crude material was purified by column chromatography (5–20% EtOAc in Pet. Ether (with 5% NEt_3)) yielding the product (85 mg, 58%) as a pale-yellow oil.

^{1}H NMR (500 MHz, $CDCl_3$) δ 7.43 – 7.38 (m, 6H), 7.38 – 7.32 (m, 3H), 7.31 – 7.26 (m, 1H), 5.17 (s, 2H), 4.09 – 3.88 (m, 2H), 3.64 (s, 2H), 3.43 – 3.25 (m, 2H), 2.00 (sept, J = 6.9 Hz, 1H), 1.75 – 1.56 (m, 2H), 1.52 – 1.37 (m, 2H), 0.94 (d, J = 6.9 Hz, 6H).

^{13}C NMR (126 MHz, $CDCl_3$): δ 155.4, 141.4, 137.1, 128.5, 128.4, 128.2, 127.9, 127.8, 126.9, 66.9, 54.6, 44.9, 39.6, 32.6, 30.0, 16.7.

IR (film, cm^{-1}): 2955, 2875, 1692, 1466, 1427, 1278, 1241, 1110, 1086, 1029, 736, 697.

HRMS: m/z calcd for $C_{23}H_{31}N_2O_2$ $[M+H]^+$ 367.2381; found 367.2382.

Benzyl 4-(but-3-en-1-ylamino)-4-isopropylpiperidine-1-carboxylate (18f)

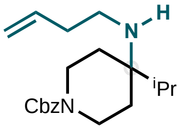

Benzyl 4-(but-3-en-1-ylamino)-4-isopropylpiperidine-1-carboxylate was prepared according to General Procedure **I** using Zn (79 mg, 1.2 mmol, 3 equiv), but-3-en-1-amine hydrochloride (43 mg, 0.4 mmol, 1 equiv), copper iodide (7.5 mg, 0.04 mmol, 10 mol%), benzyl 4-oxopiperidine-1-carboxylate (140 mg, 0.6 mmol, 1.5 equiv) and 2-iodopropane (120 µL, 1.2 mmol, 3 equiv) in dichloromethane (0.25 mL). Crude material was purified column chromatography (5–20% EtOAc in Pet. Ether (with 5% NEt_3)) yielding the product as a colourless oil (41 mg, 31%).

^{1}H NMR (500 MHz, $CDCl_3$) δ 7.38 – 7.33 (m, 3H), 7.32 – 7.28 (m, 1H), 5.80 (ddt, J = 17.1, 10.2, 6.9 Hz, 1H), 5.20 – 4.89 (m, 4H), 4.00 – 3.76 (m, 2H), 3.23 – 3.09 (m, 2H), 2.48 (t, J = 6.7 Hz, 2H), 2.20 (qt, J = 8.2, 2.1 Hz, 2H), 1.81 (sept, J = 6.9 Hz, 1H), 1.56 – 1.48 (m, 2H), 1.38 – 1.15 (m, 2H), 0.83 (d, J = 6.9 Hz, 6H).

^{13}C NMR (126 MHz, $CDCl_3$) δ 155.4, 137.1, 137.0, 128.4, 127.84, 127.79, 116.2, 66.8, 54.1, 39.6, 39.3, 35.3, 32.4, 30.0, 16.7.

IR (film, cm^{-1}): 2955, 2876, 1694, 1428, 1277, 1240, 1108, 1037, 746, 697.

HRMS: m/z calcd for $C_{20}H_{31}N_2O_2$ $[M+H]^+$ 331.2381; found 331.2375.

Benzyl 4-isopropyl-4-((2-(thiophen-2-yl)ethyl)amino)piperidine-1-carboxylate (18g)

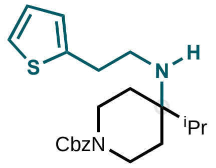

Benzyl 4-isopropyl-4-((2-(thiophen-2-yl)ethyl)amino)piperidine-1-carboxylate was prepared according to General Procedure **I** using Zn (78.5 mg, 1.2 mmol, 3 equiv), 2-(thiophen-2-yl)ethan-1-amine hydrochloride (66 mg, 0.4 mmol, 1 equiv), copper iodide (7.5 mg, 0.04 mmol, 10 mol%), benzyl 4-oxopiperidine-1-carboxylate (140 mg, 0.6 mmol, 1.5 equiv) and 2-iodopropane (120 µL, 1.2 mmol, 3 equiv) in dichloromethane (0.25 mL). Crude material was purified column chromatography (5–20% EtOAc in Pet. Ether (with 5% NEt_3)) yielding the product as a colourless oil (73 mg, 47%).

^{1}H NMR (500 MHz, $CDCl_3$) δ 7.42 – 7.37 (m, 4H), 7.35 – 7.28 (m, 1H), 7.19 – 7.13 (m, 1H), 6.99 – 6.93 (m, 1H), 6.88 – 6.83 (m, 1H), 5.15 (s, 2H), 3.95 – 3.80 (m, 2H), 3.25 – 3.11 (m, 2H), 2.99 (t, J = 6.5 Hz, 2H), 2.74 (t, J = 6.7 Hz, 2H), 1.82 (sept, J = 7.0 Hz, 1H), 1.60 – 1.49 (m, 2H), 1.44 – 1.22 (m, 2H), 0.81 (d, J = 6.9 Hz, 6H).

^{13}C NMR (126 MHz, $CDCl_3$) δ 155.3, 142.9, 137.1, 128.4, 127.8, 127.74, 126.65, 125.0, 123.6, 66.8, 54.2, 41.8, 39.5, 32.5, 31.5, 29.9, 16.6.

IR (film, cm^{-1}): 2955, 2875, 1692, 1427, 1277, 1237, 1108, 1087, 695.

HRMS: m/z calcd for $C_{22}H_{31}N_2O_2S$ $[M+H]^+$ 387.2101; found 387.2102.

N-(1-isopropylcyclobutyl)-4-methoxyaniline (19a)

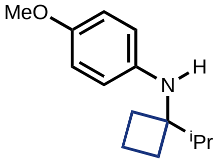

N-(1-isopropylcyclobutyl)-4-methoxyaniline was prepared according to General Procedure **H** using Zn (79 mg, 1.2 mmol, 3 equiv), *p*-anisidine (49 mg, 0.4 mmol, 1 equiv), copper iodide (7.5 mg, 0.04 mmol, 10 mol%), cyclobutanone (45 µL, 0.6 mmol, 1.5 equiv) and 2-iodopropane (120 µL, 1.2 mmol, 3 equiv) in toluene (0.25 mL). Crude material was purified by column chromatography (10-20% dichloromethane in Pet. Ether (with 4% NEt_3)) yielding the product as a brown oil (82 mg, 93%).

^{1}H NMR (400 MHz, $CDCl_3$): δ 6.78 - 6.72 (m, 2H), 6.55 - 6.47 (m, 2H), 3.75 (s, 3H), 3.42 (brs, 1H), 2.30 - 2.20 (m, 3H), 2.11 - 1.91 (m, 3H), 1.80 - 1.69 (m, 1H), 0.94 (d, J = 6.8 Hz, 6H).

^{13}C NMR (100 MHz, $CDCl_3$): δ 151.8, 140.3, 115.9, 114.8, 62.0, 55.9, 30.9, 29.3, 17.1, 14.8.

IR (film, cm^{-1}): 3398 (br), 2954, 2871, 2829, 1506, 1463, 1440, 1231, 1037, 817, 750.

HRMS: m/z calcd for $C_{14}H_{21}NO$ $[M+H]^+$ 220.1696, found 220.1699.

N-(1-isopropylcyclopentyl)-4-methoxyaniline (19b)

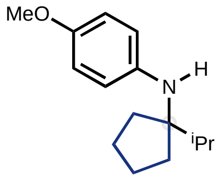

N-(1-isopropylcyclopentyl)-4-methoxyaniline was prepared according to General Procedure **H** using Zn (79 mg, 1.2 mmol, 3 equiv), *p*-anisidine (49 mg, 0.4 mmol, 1 equiv), copper iodide (7.5 mg, 0.04 mmol, 10 mol%), cyclopentanone (53 µL, 0.6 mmol, 1.5 equiv) and 2-iodopropane (120 µL, 1.2 mmol, 3 equiv) in toluene (0.25 mL). Crude material was purified by column chromatography (10-15% dichloromethane in Pet. Ether (with 4% NEt_3)) yielding the product as a brown oil (61 mg, 65%).

^{1}H NMR (400 MHz, $CDCl_3$): δ 6.76 - 6.70 (m, 2H), 6.67 - 6.60 (m, 2H), 3.74 (s, 3H), 3.45-2.5 (brs, 1H), 2.38 (sept, J = 6.8 Hz, 1H), 1.84 - 1.66 (m, 6H), 1.64 - 1.53 (m, 2H), 0.90 (d, J = 6.8 Hz, 6H).

^{13}C NMR (100 MHz, $CDCl_3$): δ 152.0, 140.8, 117.1, 114.5, 68.6, 55.9, 35.6, 31.9, 25.6, 18.2.

IR (film, cm^{-1}): 3402 (br), 2955, 2869, 1508, 1465, 1236, 1041, 818.

HRMS: m/z calcd for $C_{15}H_{23}NO$ $[M+H]^+$ 234.1853; found 234.1854.

N-(1-isopropylcyclohexyl)-4-methoxyaniline (19c)

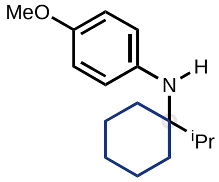

N-(1-isopropylcyclohexyl)-4-methoxyaniline was prepared according to General Procedure **H** using Zn (79 mg, 1.2 mmol, 3 equiv), *p*-anisidine (49 mg, 0.4 mmol, 1 equiv), copper iodide (7.5 mg, 0.04 mmol, 10 mol%), cyclohexanone (62 µL, 0.6 mmol, 1.5 equiv) and 2-iodopropane (120 µL, 1.2 mmol, 3 equiv) in toluene (0.25 mL). Crude material was purified by column chromatography (3-4% dichloromethane in Pet. Ether (with 4% NEt_3)) yielding the product as a brown oil (76 mg, 77%).

^{1}H NMR (400 MHz, $CDCl_3$): δ 6.77 - 6.66 (m, 4H), 3.75 (s, 3H), 3.11 (brs, 1H), 2.11 (sept, J = 6.9 Hz, 1H), 1.76 - 1.67 (m, 2H), 1.68 - 1.42 (m, 7H), 1.27 - 1.14 (m, 1H), 0.90 (d, J = 6.9 Hz, 6H).

^{13}C NMR (100 MHz, $CDCl_3$): δ 152.4, 140.8, 118.9, 114.6, 58.7, 55.7, 34.0, 31.7, 26.3, 21.7, 17.3.

IR (film, cm^{-1}): 3417 (br), 2931, 2855, 2830, 1507, 1463, 1234, 1041, 815, 761.

HRMS: m/z calcd for $C_{16}H_{25}NO$ $[M+H]^+$ 248.2009; found 248.2014.

N-(1-isopropyl-4-phenylcyclohexyl)-4-methoxyaniline (19d)

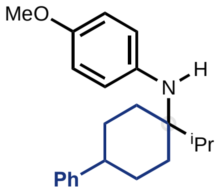

N-(1-isopropyl-4-phenylcyclohexyl)-4-methoxyaniline was prepared according to General Procedure **H** using Zn (79 mg, 1.2 mmol, 3 equiv), *p*-anisidine (49 mg, 0.4 mmol, 1 equiv), copper iodide (7.5 mg, 0.04 mmol, 10 mol%), 4-phenylcyclohexanone (105 mg, 0.6 mmol, 1.5 equiv) and 2-iodopropane (120 µL, 1.2 mmol, 3 equiv) in 0.25 mL toluene. Crude material was purified by column chromatography (0-10% (10% Et_2O: 90% Pet. Ether: 3% NEt_3) in Pet. Ether) yielding the product as a brown oil (63 mg, 49%).

NOTE: AY = 72%,8.5:1 d.r. Major Isomer isolated.

^{1}H NMR (400 MHz, $CDCl_3$): δ 7.34-7.30 (m, 2H), 7.29-7.25 (m,2H) 7.24-7.19 (m,2H), 6.81-6.74 (m, 4H), 3.79 (s, 3H), 3.20 (br s, 1H), 2.60-2.51 (m, 1H), 2.31-2.14 (m, 1H), 1.97-1.87 (m, 4H), 1.81-1.73 (m, 2H), 1.73-1.64 (m, 2H), 0.97 (d, J = 7.0 Hz, 6H).

^{13}C NMR (125 MHz, $CDCl_3$): δ 152.3, 146.4, 140.8, 128.3, 126.9, 126.0, 118.5, 114.6, 58.1, 55.7, 44.5, 34.1, 31.8, 29.2, 17.4.

IR (film, cm^{-1}): 2941, 1506, 1235, 1038, 735, 698.

HRMS: m/z calcd for $C_{22}H_{29}NO$ $[M+H]^+$ 324.2322; found 324.2323

4-isopropyl-N-(4-methoxyphenyl)tetrahydro-2H-pyran-4-amine (19e)

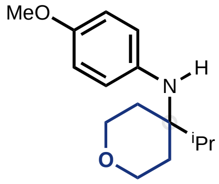

4-isopropyl-N-(4-methoxyphenyl)tetrahydro-2H-pyran-4-amine was prepared according to General Procedure **H** using Zn (79 mg, 1.2 mmol, 3 equiv), *p*-anisidine (49 mg, 0.4 mmol, 1 equiv), copper iodide (7.5 mg, 0.04 mmol, 10 mol%), 4-oxotetrahydropyran (55 µL, 0.6 mmol, 1.5 equiv) and 2-iodopropane (120 mL, 1.2 mmol, 3 equiv) in toluene (0.25 mL). Crude material was purified by column chromatography (30-50% dichloromethane in Pet. Ether (with 4% NEt_3)) yielding the product as a brown oil (70 mg, 70%).

^{1}H NMR (400 MHz, $CDCl_3$): δ 6.76 - 6.72 (m, 2H), 6.71 - 6.65 (m, 2H), 3.85 - 3.75 (m, 4H), 3.74 (s, 3H), 3.31 - 2.95 (brs, 1H), 2.13 (sept, J = 6.9 Hz, 1H), 1.91 - 1.72 (m, 1H), 1.66 - 1.59 (m, 2H), 0.91 (d, J = 6.9 Hz, 6H).

^{13}C NMR (100 MHz, $CDCl_3$): δ 152.9, 140.0, 119.4, 114.6, 63.6, 56.7, 55.7, 33.7, 31.9, 17.0.

IR (film, cm^{-1}): 3379 (br), 2954, 2865, 2832, 1506, 1234, 1102, 1037, 820, 764, 610.

HRMS: m/z calcd for $C_{15}H_{23}NO_2$ $[M+H]^+$ 250.1802; found 250.1806.

4-isopropyl-N-(4-methoxyphenyl)tetrahydro-2H-thiopyran-4-amine (19f)

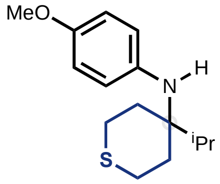

4-isopropyl-N-(4-methoxyphenyl)tetrahydro-2H-thiopyran-4-amine was prepared according to General Procedure **H** using Zn (79 mg, 1.2 mmol, 3 equiv), *p*-anisidine (49 mg, 0.4 mmol, 1 equiv), copper iodide (7.5 mg, 0.04 mmol, 10 mol%), tetrahydro-4*H*-thiopyran-4-one (70 mg, 0.6 mmol, 1.5 equiv) and 2-iodopropane (120 µL, 1.2 mmol, 3 equiv) in dichloromethane (0.25 mL). Crude material was purified by column chromatography (3-9% dichloromethane in Pet. Ether (with 4% NEt_3)) yielding the product as a brown oil (88 mg, 83%).

^{1}H NMR (400 MHz, $CDCl_3$): δ 6.74 (d, J = 8.6 Hz, 2H), 6.67 (d, J = 8.6 Hz, 2H), 3.74 (s, 3H), 3.15 - 2.93 (m, 3H), 2.44 - 2.36 (m, 2H), 2.10 - 1.97 (m, 3H), 1.95 - 1.84 (m, 2H), 0.89 (d, J = 6.9 Hz, 6H).

^{13}C NMR (100 MHz, $CDCl_3$): δ 153.0, 139.8, 119.4, 114.6, 57.5, 55.6, 34.0, 33.4, 23.7, 16.8.

IR (film, cm^{-1}): 3357 (br), 2956, 2831, 1506, 1464, 1440, 1277, 1234, 1206, 1178, 1076, 909, 818, 791, 761, 730, 647.

HRMS: m/z calcd for $C_{15}H_{23}NOS$ $[M+H]^+$ 266.1574; found 266.1578.

Benzyl 4-isopropyl-4-((4-methoxyphenyl)amino)piperidine-1-carboxylate (19g)

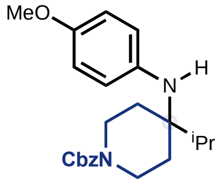

Benzyl 4-isopropyl-4-((4-methoxyphenyl)amino)piperidine-1-carboxylate was prepared according to General Procedure **H** using Zn (79 mg, 1.2 mmol, 3 equiv), 4-methoxyaniline hydrochloride (64 mg, 0.4 mmol, 1 equiv), copper iodide (7.6 mg, 0.04 mmol, 0.1 equiv), benzyl 4-oxopiperidine-1-carboxylate (140 mg, 0.6 mmol, 1.5 equiv) and 2-iodopropane (120 µL, 1.2 mmol, 3 equiv) in dichloromethane (0.25 mL). Crude material was purified by column chromatography (0–5% MeOH in dichloromethane (with 5% Et_3N)) yielding the product (72 mg, 47%) as a pale-yellow oil.

^{1}H NMR (500 MHz, $CDCl_3$): δ 7.39 – 7.34 (m, 4H), 7.34 – 7.30 (m, 1H), 6.77 – 6.72 (m, 2H), 6.72 – 6.65 (m, 2H), 5.14 (s, 2H), 4.06 – 3.91 (m, 2H), 3.74 (s, 3H), 3.31 – 3.21 (m, 2H), 3.09 (brs, 1H), 2.11 (sept, J = 6.9 Hz, 1H), 1.81 – 1.58 (m, 4H), 0.88 (d, J = 6.9 Hz, 6H).

^{13}C NMR (126 MHz, $CDCl_3$): δ 155.4, 153.0, 139.8, 137.0, 128.5, 127.9, 127.8, 119.5, 114.6, 67.0, 57.2, 55.6, 39.7, 33.3, 31.3, 17.0.

IR (film, cm^{-1}): 3360, 2957, 2874, 1687, 1507, 1429, 1279, 1233, 1112, 1037, 821, 697.

HRMS: m/z calcd for $C_{23}H_{30}N_2NaO_3$ $[M+Na]^+$ 405.2149; found 405.2145.

Ethyl 3-isopropyl-3-((4-methoxyphenyl)amino)cyclobutane-1-carboxylate (19h)

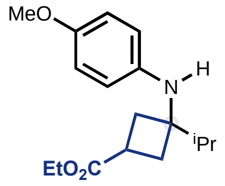

Ethyl 3-isopropyl-3-((4-methoxyphenyl)amino)cyclobutane-1-carboxylate was prepared according to General Procedure **H** using Zn (79 mg, 1.2 mmol, 3 equiv), *p*-anisidine (49 mg, 0.4 mmol, 1 equiv), copper iodide (7.5 mg, 0.04 mmol, 10 mol%), ethyl 3-oxocyclobutanecarboxylate (85 mg, 0.6 mmol, 1.5 equiv) and 2-iodopropane (120 µL, 1.2 mmol, 3 equiv) in toluene (0.25 mL). Crude material was purified by column chromatography (15-30% dichloromethane in Pet. Ether (with 4% NEt_3)) yielding the product as a brown oil (93 mg, 80%, 1.1:1 d.r.).

^{1}H NMR (400 MHz, $CDCl_3$): δ 6.78 - 6.70 (m, 2H), 6.55 - 6.46 (m, 2H), 4.17 - 4.09 (m, 2H), 3.74 - 3.73 (m, 3H), 3.26 - 3.23 (brs, 1H), 3.18 (quint, J = 8.9 Hz, 0.57 H), 2.87 (quint, J = 8.9 Hz, 0.48 H), 2.54-2.43 (m, 2H), 2.41 - 2.32 (m, 1H), 2.30 - 2.15 (m, 2H), 1.27 - 1.21 (m, 3H), 0.95 (d, J = 6.8 Hz, 2.9 H), 0.88 (d, J = 6.8 Hz, 3.1 H).

^{13}C NMR (100 MHz, $CDCl_3$): δ 175.6, 175.3, 152.1, 139.8, 139.6, 116.3, 116.1, 114.8, 114.7, 60.6, 60.5, 60.0, 58.2, 55.83, 55.80, 33.4, 31.8, 31.7, 31.5, 40.0, 31.0, 17.2, 17.0, 14.33, 14.30.

IR (film, cm^{-1}): 3396 (br), 2957, 2905, 2831, 1722, 1617, 1508, 1464, 1234, 1176, 1037, 819, 755.

HRMS: m/z calcd for $C_{17}H_{25}NO_3$ $[M+H]^+$ 292.1908; found 292.1914.

3-isopropyl-N-(4-methoxyphenyl)oxetan-3-amine (19i)

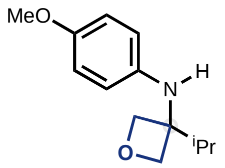

3-isopropyl-N-(4-methoxyphenyl)oxetan-3-amine was prepared according to General Procedure **J** using Zn (118 mg, 1.8 mmol, 3 equiv), *p*-anisidine (74 mg, 0.6 mmol, 1 equiv), copper iodide (57 mg, 0.3 mmol, 50 mol%), oxetan-3-one (88 µL, 1.5 mmol, 2.5 equiv), 2-iodopropane (240 µL, 2.4 mmol, 4.0 equiv) and TMSOTf (207 µL, 1.2 mmol, 2.0 equiv). Crude material was purified by reverse phase column chromatography (0-95% MeCN in H_2O). The product fractions were combined, the MeCN was removed in *vacuo* and extracted with dichloromethane (3 x 20 mL). The solvent was removed in vacuo, yielding the product as a yellow oil (84 mg, 63%).

Note: Not stable on silica

^{1}H NMR (400 MHz, $CDCl_3$): δ 6.80 - 6.75 (m, 2H), 6.52 - 6.47 (m, 2H), 4.75 (d, J = 6.5 Hz, 2H), 4.65 (d, J = 6.3 Hz, 2H), 3.75 (s, 3H), 3.66 (brs, 1H), 2.39 (sept, J = 6.9 Hz, 1H), 1.03 (d, J = 6.9 Hz, 6H).

^{13}C NMR (100 MHz, $CDCl_3$): δ 152.5, 138.9, 115.8, 114.8, 78.2, 61.6, 55.8, 30.7, 16.7.

IR (thin film, cm^{-1}): 2954, 2875, 1741, 1604, 1520, 1425, 1300, 1204, 1116, 1033, 1000, 937, 825, 725, 634, 500.

HRMS: m/z calcd for $C_{13}H_{19}NO_2$ $[M+H]^+$ 222.1489, found 222.1489.

Scaleup: Synthesis of 3-isopropyl-N-(4-methoxyphenyl)oxetan-3-amine on 2 mmol

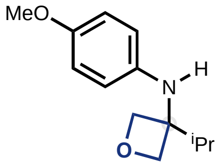

3-isopropyl-N-(4-methoxyphenyl)oxetan-3-amine was prepared according to General Procedure **J** using Zn (392 mg, 6.0 mmol, 3 equiv), p-anisidine (246 mg, 2.0 mmol, 1 equiv), copper iodide (191 mg, 1.0 mmol, 50 mol%), oxetan-3-one (290 µL, 5.0 mmol, 2.5 equiv), 2-iodopropane (800 µL, 8.0 mmol, 4.0 equiv) and TMSOTf (0.69 mL, 4.0 mmol, 2.0 equiv). Crude material was purified by column chromatography using alumina (0-10% EtOAc in Pet. Ether) yielding the product as a yellow oil (217 mg, 49%).

N-(2,3-dimethylhexan-3-yl)-4-methoxyaniline (20a)

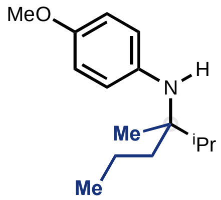

N-(2,3-dimethylhexan-3-yl)-4-methoxyaniline was prepared according to General Procedure **H** using Zn (79 mg, 1.2 mmol, 3 equiv), *p*-anisidine (49 mg, 0.4 mmol, 1 equiv), copper iodide (7.5 mg, 0.04 mmol, 10 mol%), 2-pentanone (64 µL, 0.6 mmol, 1.5 equiv) and 2-iodopropane (120 µL, 1.2 mmol, 3 equiv) in toluene (0.25 mL). Crude material was purified by column chromatography (5-10% dichloromethane in Pet. Ether (with 4% NEt_3)) yielding the product as a brown oil (78 mg, 83%).

^{1}H NMR (400 MHz, $CDCl_3$): δ 6.76 - 6.71 (m, 2H), 6.70 - 6.66 (m, 2H), 3.75 (s, 3H), 3.08 (brs, 1H), 2.38 (sept, *J* = 6.8 Hz, 1H), 1.59 - 1.45 (m, 2H), 1.43 - 1.31 (m, 2H), 1.08 (s, 3H), 0.94 - 0.87 (m, 9H).

^{13}C NMR (100 MHz, $CDCl_3$): δ 152.8, 140.8, 119.5, 114.5, 59.1, 55.7, 40.1, 34.6, 22.1, 17.7, 17.3, 16.9, 14.8.

IR (film, cm^{-1}): 3408 (br), 2956, 2871, 2831, 1507, 1464, 1234, 1179, 1040, 816, 732.

HRMS: m/z calcd for $C_{15}H_{25}NO$ $[M+H]^+$ 236.2009; found 236.2015.

N-(3,4-dimethyl-1-phenylpentan-3-yl)-4-methoxyaniline (20b)

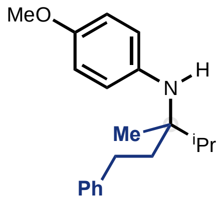

N-(3,4-dimethyl-1-phenylpentan-3-yl)-4-methoxyaniline was prepared according to General Procedure **h** using Zn (79 mg, 1.2 mmol, 3 equiv), *p*-anisidine (49 mg, 0.4 mmol, 1 equiv), copper iodide (7.5 mg, 0.04 mmol, 10 mol%), 4-phenyl-2-butanone (90 µL, 0.6 mmol, 1.5 equiv) and 2-iodopropane (120 µL, 1.2 mmol, 3 equiv) in toluene (0.25 ml). Crude material was purified by column chromatography (2-4% dichloromethane in Pet. Ether (with 4% NEt_3)) yielding the product as a brown oil (71 mg, 60%).

^{1}H NMR (700 MHz, $CDCl_3$): δ 7.28 (t, J = 7.4 Hz, 2H), 7.19 (t, J = 7.3 Hz, 1H), 7.15 (d, J = 7.5 Hz, 2H), 6.81 - 6.74 (m, 4H), 3.78 (s, 3H), 3.35 - 3.16 (brs, 1H), 2.74 - 2.65 (m, 2H), 2.13 (sept, J = 6.8 Hz, 1H), 1.97-1.87 (m, 2H), 1.19 (s, 3H), 0.98 - 0.95 (m, 6H).

^{13}C NMR (100 MHz, $CDCl_3$): δ 152.9, 143.3, 140.6, 128.48, 128.47, 125.7, 119.1, 114.7, 59.1, 55.8, 40.2, 34.5, 30.3, 21.8, 17.7, 17.3.

IR (film, cm^{-1}): 3406 (br), 3024, 2955, 2873, 1602, 1506, 1453, 1235, 1178, 1038, 817, 747, 698.

HRMS: m/z calcd for $C_{20}H_{27}NO$ $[M+H]^+$ 298.2166; found 298.2169.

Scaleup synthesis of N-(3,4-dimethyl-1-phenylpentan-3-yl)-4-methoxyaniline (20b) on 4 mmol

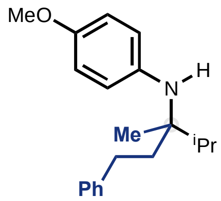

N-(3,4-dimethyl-1-phenylpentan-3-yl)-4-methoxyaniline was synthesised on a 4 mmol scale using *p*-anisidine (493 mg, 4 mmol, 1 equiv), zinc (785 mg, 12 mmol, 3 equiv) and copper iodide (75 mg, 0.4 mmol, 10 mol%) were added to a Biotage Microwave Process Vial (25 mL). The vial was sealed, put under vacuum, and refilled with N_2. toluene (2.5 mL) was added. While stirring, 4-phenylbutan-2-one (0.90 mL, 6 mmol, 1.5 equiv) was added dropwise, followed by 2-iodopropane (1.2 mL, 12 mmol, 3 equiv). Then H_2O (108 µL, 6 mmol, 1.5 equiv) and TMSOTf (1.1 mL, 6 mmol, 1.5 equiv) were added dropwise at 0 °C. The mixture was allowed to warm to rt and stirred overnight. The mixture was worked up according to general procedure **H**. Crude material was purified by column chromatography (1-5% dichloromethane in Pet. Ether (with 4% NEt_3)), which afforded a mixture of the desired product and unreacted 4-phenylbutan-2-one. The mixture was subjected to high vacuum under gentle heating to remove the ketone, affording the product as a brown oil (887 mg, 75%).

Removal of PMP group

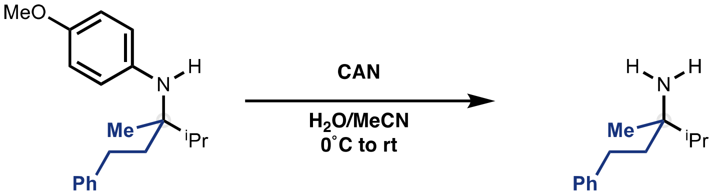

N-(3,4-dimethyl-1-phenylpentan-3-yl)-4-methoxyaniline (119 mg, 0.4 mmol, 1 equiv) was dissolved in MeCN (4 mL). CAN (542 mg, 1.08 mmol, 2.7 equiv) in H_2O (2 mL) was added dropwise at 0 °C. The mixture was stirred at 0 °C for 4h and then at rt for 2 h. $NaHCO_3$ (saturated, aq), Na_2SO_3 (saturated, aq), Rochelle salts (saturated, aq) and EtOAc were added. The aqueous and organic layers were separated. The aqueous layer was extracted with EtOAc three times. The combined organic layer was dried over Na_2SO_4 and filtered. The solvent was removed under vacuum. ^{1}H NMR analysis of the crude product with 1,1,2,2-tetrachloroethane as an internal standard indicated that the product was formed in 89%. Column chromatography (2-8% NEt_3 in EtOAc) afforded 3,4-dimethyl-1-phenylpentan-3-amine as a colourless oil (45 mg, 64%).

^{1}H NMR (400 MHz, $CDCl_3$): δ 7.34 - 7.27 (m, 2H), 7.26 - 7.17 (m, 3H), 2.74 - 2.60 (m, 2H), 1.77 - 1.64 (m, 3H), 1.43 - 1.18 (brs, 2H), 1.09 (s, 3H), 0.98 - 0.92 (m, 6H).

^{13}C NMR (100 MHz, $CDCl_3$): δ 143.2, 128.5, 128.4, 125.8, 53.7, 43.1, 36.8, 30.3, 24.7, 17.5, 17.1.

IR (film, cm^{-1}): 3367, 3303, 3062, 3026, 2960, 2873, 1601, 1496, 1454, 1372, 811, 750, 698.

HRMS: m/z calcd for $C_{13}H_{21}N$ $[M+H]^+$ 192.1747; found 192.1751.

N-(2,3-dimethylhept-6-en-3-yl)-4-methoxyaniline (20c)

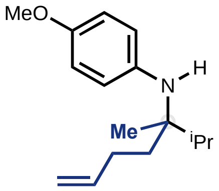

N-(2,3-dimethylhept-6-en-3-yl)-4-methoxyaniline was prepared according to General Procedure **H** using Zn (79 mg, 1.2 mmol, 3 equiv), *p*-anisidine (49 mg, 0.4 mmol, 1 equiv), copper iodide (7.5 mg, 0.04 mmol, 10 mol%), 5-hexen-2-one (70 µL, 0.6 mmol, 1.5 equiv) and 2-iodopropane (120 µL, 1.2 mmol, 3 equiv) in toluene (0.25 mL). Crude material was purified by column chromatography (4-5% dichloromethane in Pet. Ether (with 4% NEt_3)) yielding the product as a brown oil (64 mg, 65%)

^{1}H NMR (400 MHz, $CDCl_3$): δ 6.74 (d, J = 8.3 Hz, 2H), 6.68 (d, J = 8.3 Hz, 2H), 5.90 - 5.76 (m, 1H), 5.00 (d, J = 17.1 Hz, 1H), 4.93 (d, J = 10.1 Hz, 1H), 3.75 (s, 3H), 3.33 - 2.86 (brs, 1H), 2.20 - 1.97 (m, 3H), 1.74 - 1.58 (m, 2H), 1.10 (s, 3H), 0.95 - 0.88 (m, 6H).

^{13}C NMR (100 MHz, $CDCl_3$): δ 152.9, 140.5, 139.5, 119.4, 114.6, 114.2, 59.0, 55.7, 36.9, 34.5, 28.2, 21.9, 17.7, 17.3.

IR (film, cm^{-1}): 3403 (br), 3074, 2958, 2875, 2831, 1639, 1507, 1464, 1388, 1372, 1300, 1236, 1197, 1179, 1114, 1040, 995, 909, 818, 764.

HRMS: m/z calcd for $C_{16}H_{25}NO$ $[M+H]^+$ 248.2009, found 248.2014.

Ethyl 5-((4-methoxyphenyl)amino)-5,6-dimethylheptanoate (20d)

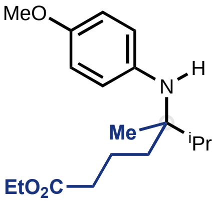

Ethyl 5-((4-methoxyphenyl)amino)-5,6-dimethylheptanoate was prepared according to General Procedure **H** using Zn (78.5 mg, 1.2 mmol, 3 equiv), *p*-anisidine (49 mg, 0.4 mmol, 1 equiv), copper iodide (7.5 mg, 0.04 mmol, 10 mol%), ethyl 4-acetylbutyrate (96 µL, 0.6 mmol, 1.5 equiv) and 2-iodopropane (120 µL, 1.2 mmol, 3 equiv) in toluene (0.25 mL). Crude material was purified by column chromatography (10-18% dichloromethane in Pet. Ether (with 4% NEt_3)) yielding the product as a brown oil (69 mg, 56%).

^{1}H NMR (400 MHz, $CDCl_3$): δ 6.72 (d, J = 8.8 Hz, 2H), 6.66 (d, J = 8.6 Hz, 2H), 4.10 (q, J = 7.1 Hz, 2H), 3.73 (s, 3H), 3.27 - 3.01 (brs, 1H), 2.26 (t, J = 7.1 Hz, 2H), 2.02 (sept, J = 6.8 Hz, 1H), 1.75 - 1.50 (m, 4H), 1.23 (t, J = 7.1, 3H), 1.09 (s, 3H), 0.90 (d, J = 6.8 Hz, 6H).

^{13}C NMR (100 MHz, $CDCl_3$): δ 173.8, 152.8, 140.5, 119.3, 114.5, 60.3, 58.9, 55.7, 37.0, 34.8, 34.5, 21.8, 19.3, 17.6, 17.2, 14.3.

IR (film, cm^{-1}): 3403 (br), 2960, 2875, 2831, 1727, 1507, 1372, 1235, 1179, 1115, 1038, 909, 818, 730.

HRMS: m/z calcd for $C_{18}H_{29}NO_3$ $[M+H]^+$ 308.2221; found 308.2228.

Synthesis of N-(4-methoxyphenyl)heptan-4-imine (Unoptimized)

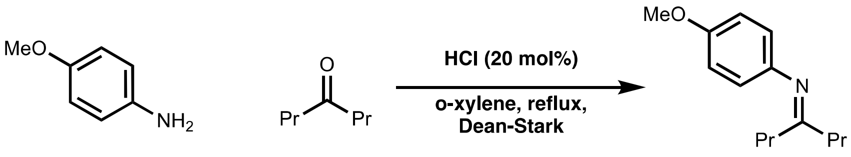

A mixture of *p*-anisidine (6.7 g, 54.7 mmol, 1 equiv), 4-heptanone (9.6 mL, 68.4 mmol, 1.25 equiv) and concentrated HCl (0.91 mL, 10.9 mmol, 20 mol%) in *o*-xylene (3 mL) was refluxed with the Dean-Stark apparatus overnight. The reaction was cooled to rt. The mixture was filtered through celite. Solvent was removed *in vacuo*. The crude mixture was purified by column chromatography (2% Et_2O in Pet. Ether (with 4% Et_3N))) to afford *N*-(4-methoxyphenyl)heptan-4-imine as a slightly yellow oil (2.8 g, 23%).

Notes: This imine hydrolyses easily under ambident conditions, should be stored under nitrogen and used within days after synthesis. A pure ^{13}C NMR spectrum cannot be obtained as the imine hydrolyses during data acquisition. $[M+H]^+$ peak cannot be observed in MS due to rapid hydrolysis of the imine.

^{1}H NMR (400 MHz, $CDCl_3$): δ 6.83 (d, J = 7.0 Hz, 2H)), 6.62 (d, J =6.9 Hz, 2H), 3.79 (s, J = 3H), 2.40 (d, J = 6.7 Hz, 2H), 2.13 (t, J = 6.9 Hz, 2H), 1.69 (sext, J = 7.2 Hz, 2H), 1.48 (sext, J = 7.2 Hz, 2H), 1.00 (t, J = 6.9 Hz, 3H), 0.82 (t, J = 7.0 Hz, 3H).

IR (film, cm^{-1}): 2959, 2872, 2832, 1652, 1607, 1500, 1464, 1440, 1286, 1237, 1207, 1180, 1101, 1035, 836, 759.

N-(4-isopropylheptan-4-yl)-4-methoxyaniline (20e)

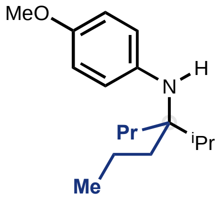

An oven dried vial (Biotage Microwave Process Vial (10 mL), equipped with gas-tight pierceable caps) was charged with a stirring bar, Zn dust (105 mg, 1.6 mmol, 4 equiv) and copper iodide (7.5 mg, 0.04 mmol, 10 mol%). The vial was sealed, evacuated and backfilled with N_2. To this was added dry toluene (0.25 mL), followed by N-(4-methoxyphenyl)heptan-4-imine (88 mg, 0.4 mmol, 1 equiv), 2-iodopropane (160 µL, 1.6 mmol, 4 equiv). H_2O (11 µL, 0.6 mmol, 1.5 equiv) and TMSOTf (109 µL, 0.6 mmol, 1.5 equiv) were subsequently added. The reaction mixture was stirred overnight. The mixture was worked up according to general procedure **H**. Crude material was purified by column chromatography (3-5% dichloromethane in Pet. Ether (with 4% NEt_3)) to afford the product as a brown oil (60 mg, 57%).

^{1}H NMR (400 MHz, $CDCl_3$): δ 6.72 (d, J = 8.0 Hz, 2H), 6.64 (d, J = 8.1 Hz, 2H), 3.74 (s, 3H), 3.12 (brs, 1H), 1.99 (sept, J = 6.8 Hz, 1H), 1.59 - 1.50 (m, 4H), 1.42 - 1.29 (m, 4H), 0.96 - 0.84 (m, 12H).

^{13}C NMR (100 MHz, $CDCl_3$): δ 152.5, 141.3, 119.0, 114.5, 60.9, 55.7, 39.3, 35.1, 17.9, 17.7, 15.1.

IR (film, cm^{-1}): 3403, 2956, 2871, 1507, 1464, 1234, 1178, 1041, 816.

HRMS: m/z calcd for $C_{17}H_{29}NO$ $[M+H]^+$ 264.2322; found 264.2324.

N-(2,3-dimethylpentan-2-yl)-4-methoxyaniline (21a)

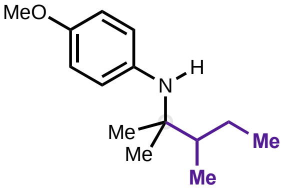

N-(2,3-dimethylpentan-2-yl)-4-methoxyaniline was prepared according to General Procedure **H** using Zn (79 mg, 1.2 mmol, 3 equiv), *p*-anisidine (49 mg, 0.4 mmol, 1 equiv), copper iodide (7.5 mg, 0.04 mmol, 10 mol%), acetone (44 µL, 0.6 mmol, 1.5 equiv) and 2-iodobutane (138 µL, 1.2 mmol, 3 equiv) in toluene (0.25 mL). Crude material was purified by column chromatography (4-10% dichloromethane in Pet. Ether (with 4% NEt_3)) yielding the product as a brown oil (79 mg, 89%).

^{1}H NMR (700 MHz, $CDCl_3$): δ 6.76 - 6.74 (m, 4H), 3.75 (s, 3H), 3.15 - 2.78 (brs, 1H), 1.78 - 1.71 (m, 1H), 1.59 - 1.53 (m, 1H), 1.17 - 1.13 (m, 6H), 1.04 - 0.96 (m, 1H), 0.94 - 0.90 (m, 6H).

^{13}C NMR (100 MHz, $CDCl_3$): δ 153.7, 140.3, 121.5, 114.3, 57.5, 55.7, 43.6, 25.7, 25.2, 24.3, 13.8, 13.1.

IR (film, cm^{-1}): 3402 (br), 2962, 2932, 2874, 1506, 1463, 1235, 1178, 1039, 818.

HRMS: m/z calcd for $C_{14}H_{23}NO$ $[M+H]^+$ 222.1853; found 222.1856.

N-(2-cyclopentylpropan-2-yl)-4-methoxyaniline (21b)

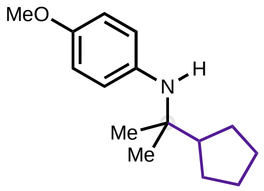

N-(2-cyclopentylpropan-2-yl)-4-methoxyaniline was prepared according to General Procedure **H** using Zn (79 mg, 1.2 mmol, 3 equiv), *p*-anisidine (49 mg, 0.4 mmol, 1 equiv), copper iodide (7.5 mg, 0.04 mmol, 10 mol%), acetone (44 µL, 0.6 mmol, 1.5 equiv) and cyclopentyl iodide (139 µL, 1.2 mmol, 3 equiv) in toluene (0.25 mL). Crude material was purified by column chromatography (5-10% dichloromethane in Pet. Ether (with 4% NEt_3)) yielding the product as a brown oil (81 mg, 87%)

^{1}H NMR (700 MHz, $CDCl_3$): δ 6.80 (d, J = 8.8 Hz, 2H), 6.75 (d, J = 8.9 Hz, 2H), 3.75 (s, 3H), 3.05 - 2.89 (brs, 1H), 2.09 (quint, J = 8.8 Hz, 1H), 1.71 - 1.65 (m, 2H), 1.64 - 1.58 (m, 2H), 1.58 - 1.50 (m, 2H), 1.42-1.35 (m, 2H), 1.16 (s, 6H).

^{13}C NMR (175 MHz, $CDCl_3$): δ 154.2, 140.0, 122.7, 114.2, 56.8, 55.6, 50.1, 27.6, 26.2, 25.8.

IR (film, cm^{-1}): 3370 (br), 2949, 2866, 1500, 1463, 1236, 1178, 1038, 818.

HRMS: m/z calcd for $C_{15}H_{23}NO$ $[M+H]^+$ 234.1853; found 234.1858.

N-(2-(2,3-dihydro-1H-inden-2-yl)propan-2-yl)-4-methoxyaniline (21c)

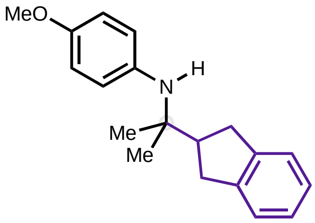

N-(2-(2,3-dihydro-1H-inden-2-yl)propan-2-yl)-4-methoxyaniline was prepared according to General Procedure **H** using Zn (79 mg, 1.2 mmol, 3 equiv), *p*-anisidine (49 mg, 0.4 mmol, 1 equiv), copper iodide (7.5 mg, 0.04 mmol, 10 mol%), acetone (44 µL, 0.6 mmol, 1.5 equiv) and 2-iodo-2,3-dihydro-1*H*-indene[13] (293 mg, 1.2 mmol, 3 equiv) in toluene (0.25 mL). Crude material was purified by column chromatography (1-4% dichloromethane in Pet. Ether (with 4% NEt_3)) yielding the product as a brown oil (72 mg, 64%).

^{1}H NMR (400 MHz, $CDCl_3$): δ 7.23 - 7.18 (m, 2H), 7.18 - 7.13 (m, 2H), 6.90 - 6.83 (m, 2H), 6.81 - 6.75 (m, 2H), 3.77 (s, 3H), 3.03 - 2.89 (m, 4H), 2.80 (quint, J = 8.9 Hz, 1H), 1.26 (s, 6H).

^{13}C NMR (100 MHz, $CDCl_3$): δ 154.5, 143.1, 139.4, 126.4, 124.6, 122.9, 114.3, 56.8, 55.7, 50.0, 34.5, 25.9.

IR (film, cm^{-1}): 3370 (br), 3067, 3021, 2933, 2906, 1507, 1484, 1460, 1238, 1223, 1179, 1038, 822, 743.

HRMS: m/z calcd for $C_{19}H_{23}NO$ $[M+H]^+$ 282.1853, found 282.1855.

4-methoxy-N-(2-methylpentan-2-yl)aniline (21d)

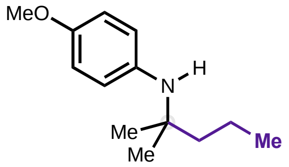

4-methoxy-N-(2-methylpentan-2-yl)aniline was prepared according to General Procedure **H** with modifications using Zn (209 mg, 3.2 mmol, 8 equiv), *p*-anisidine (49 mg, 0.4 mmol, 1 equiv), copper iodide (7.5 mg, 0.04 mmol, 10 mol%), acetone (44 µL, 0.6 mmol, 1.5 equiv) and propyl iodide (195 µL, 2.0 mmol, 5 equiv) in toluene (0.25 mL). The reaction time was 72 h. Crude material was purified by column chromatography (3-8% dichloromethane in Pet. Ether (with 4% NEt_3)) yielding the product as a brown oil (57 mg, 69%).

^{1}H NMR (400 MHz, $CDCl_3$): δ 6.80 - 6.73 (m, 4H), 3.76 (s, 3H), 2.98 (brs, 1H), 1.52 - 1.45 (m, 2H), 1.44 - 1.32 (m, 2H), 1.19 (s, 6H), 0.91 (t, J = 7.1 Hz, 3H).

^{13}C NMR (100 MHz, $CDCl_3$): δ 154.1, 139.7, 122.2, 114.3, 55.6, 54.8, 44.6, 28.2, 17.6, 14.8.

IR (film, cm^{-1}): 3370 (br), 2956, 2870, 1506, 1234, 1178, 1038, 821, 770.

HRMS: m/z calcd for $C_{13}H_{21}NO$ $[M+H]^+$ 208.1696, found 208.1697.

10. Mechanistic investigations and control experiments

10.1 Formation of alkyl-zinc reagents

10.1.1 General procedure K for alkyl-zinc formation[14,15]

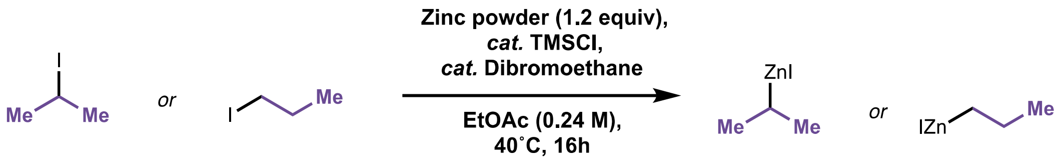

Zinc dust (780 mg, 12 mmol, 1.2 equiv) was added to a dry round bottom flask. Degassed solvent (2.5 mL) was added. 1,2-dibromoethane (43 µL) was added. The mixture was stirred at 65 °C for 3 mins. The mixture was cooled to rt. TMSCl (63 µL) was added. The mixture was stirred at rt for 15 mins. Propyl iodide (975 µL, 10 mmol, 1 equiv) or 2-iodopropane (1 mL, 10 mmol, 1 equiv) was dissolved in solvent (2.5 mL) in another dry round bottom flask. The alkyl iodide solution was added dropwise. Solvent (2.5 mL) was used to rinse the round bottom flask which the alkyl iodide was first dissolved in and was transferred to the round bottom flask containing zinc. The mixture was stirred at 40°C overnight. The mixture was cooled to r.t. and the stirring was stopped to let the remaining zinc settle. The supernatant solution was transferred to a dry round bottom flask. The concentration of alkylzinc iodide was determined by titration against I_2 before use.[16]

10.1.2 General procedure J for alkyl zinc formation[14,15]

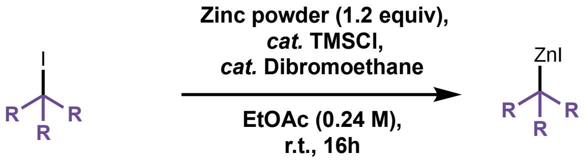

Zinc dust (780 mg, 12 mmol, 1.2 equiv) was added to a dry round bottom flask. Degassed solvent (2.5 mL) was added. 1,2-dibromoethane (43 µL) was added. The mixture was stirred at 65 °C for 3 mins. The mixture was cooled to rt. TMSCl (63 µL) was added. The mixture was stirred at rt for 15 mins. Propyl iodide (975 µL, 10 mmol, 1 equiv), 2-iodopropane (1 mL, 10 mmol, 1 equiv) or tert-butyl iodide (1.2 mL, 10 mmol, 1 equiv) was dissolved in solvent (2.5 mL) in another dry round bottom flask. The alkyl iodide solution was added dropwise. Solvent (2.5 mL) was used to rinse the round bottom flask which the alkyl iodide was first dissolved in and was transferred to the round bottom flask containing zinc. The mixture was stirred at r.t. overnight. The stirring was stopped to let the remaining zinc settle. The concentration of alkylzinc iodide was determined by titration against I_2 before use.[16]

10.1.3 General procedure L for alkyl zinc formation[14,15]

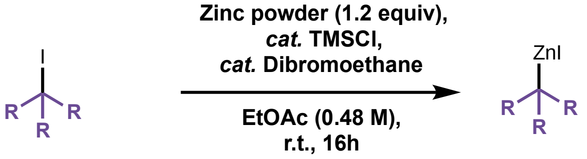

Zinc dust (780 mg, 12 mmol, 1.2 equiv) was added to a dry round bottom flask. Toluene (2.5 mL) was added. 1,2-dibromoethane (43 µL) was added. The mixture was stirred at 65 ºC for 3 mins. The mixture was cooled to rt. TMSCl (63 µL) was added. The mixture was stirred at r.t. for 15 mins. 2-iodopropane (1 mL, 10 mmol, 1 equiv) was added dropwise. The mixture was stirred at rt overnight. The stirring was stopped to let the remaining zinc settle. The concentration of alkylzinc iodide was determined by titration against I_2 before use.[16]

10.2 Reactivity of alkyl-zinc reagents

10.2.1 Primary alkyl-zinc reactivity

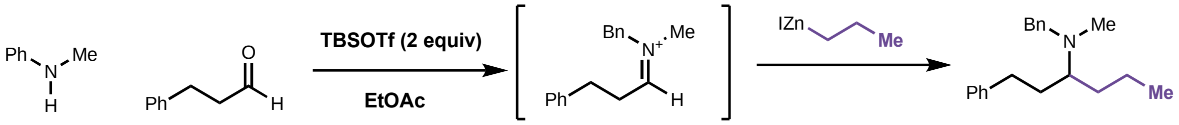

A Biotage Microwave Process microwave vial (10 mL) was sealed, put under vacuum, and refilled with. N-methylbenzylamine (26 mL, 0.2 mmol, 1 equiv) was dissolved in EtOAc (1 mL). Hydrocinnamaldehyde (53 mL, 0.4 mmol, 2 equiv) was added, followed by TBSOTf (92 mL, 0.4 mmol, 2 equiv). The mixture was allowed to stir for 15 mins. A *n*-propyl zinc iodide solution, prepared according to General Procedure **J** (1.4 M, 429 µL, 0.6 mmol, 3 equiv) in EtOAc was added. The mixture was stirred overnight. The mixture was diluted with dichloromethane. NaOH (10%, aq) solution was added. The heterogeneous mixture was stirred for 15 mins vigorously. The organic and aqueous layers were separated. The aqueous layer was extracted with dichloromethane three times. The combined organic layer was dried over Na_2SO_4 and filtered. The solvent was removed *in vacuo*. The expected product was formed in approximately 20% from ^1H NMR spectroscopic analysis of the crude mixture. The second step was repeated using a propyl zinc solution prepared in the presence of 40 mol% copper iodide (1.1 M, 544 µL, 0.6 mmol, 3 equiv). The expected product was formed in 20% from ^1H NMR spectroscopic analysis of the crude mixture.

10.2.2 Secondary alkyl-zinc reactivity

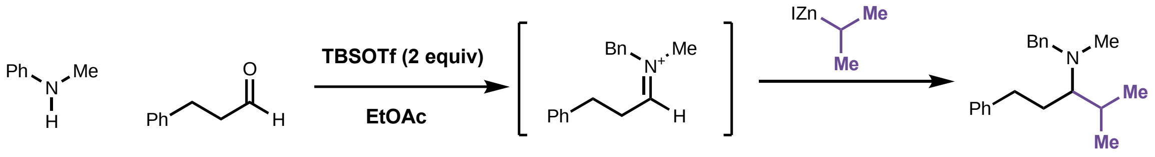

A Biotage Microwave Process microwave vial (10 mL) was sealed, put under vacuum and refilled with N_2. N-methylbenzylamine (26 mL, 0.2 mmol, 1 equiv) was dissolved in EtOAc (1 mL). Hydrocinnamaldehyde (53 mL, 0.4 mmol, 2 equiv) was added, followed by TBSOTf (92 µL, 0.4 mmol, 2 equiv). The mixture was allowed to stir for 15 mins. The isopropylzinc iodide solution, prepared according to General Procedure **K**, (1 M, 0.60 mL, 0.6 mmol, 3 equiv) in EtOAc was added. The mixture was stirred overnight. The mixture was diluted with dichloromethane. NaOH (10%, aq) solution was added. The heterogeneous mixture was stirred for 15 mins vigorously. The organic and aqueous layer were separated. The aqueous layer was extracted with dichloromethane three times. The combined organic layer was dried over Na_2SO_4 and filtered. The expected product was formed in 79% from ^1H NMR spectroscopic analysis of the crude mixture.

10.2.3 Tertiary alkyl-zinc reactivity

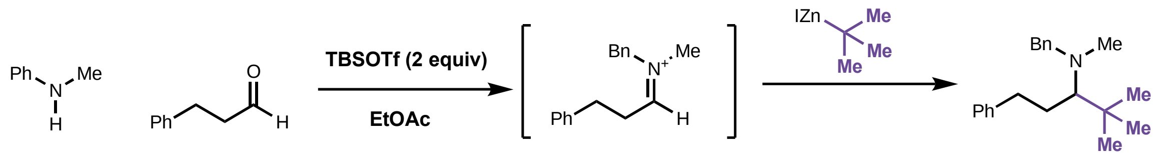

A Biotage Microwave Process microwave vial (10 mL) was sealed, put under vacuum and refilled with N_2. N-methylbenzylamine (26 µL, 0.2 mmol, 1 equiv) was dissolved in EtOAc (1 mL). hydrocinnamaldehyde (53 µL, 0.4 mmol, 2 equiv) was added, followed by TBSOTf (92 µL, 0.4 mmol, 2 equiv). The mixture was allowed to stir for 15 mins. The tertbutylzinc iodide solution, prepared according to General Procedure **J**, (1.1 M, 0.55 mL, 0.6 mmol, 3 equiv) in EtOAc was added. The mixture was stirred overnight. The mixture was diluted with dichloromethane. NaOH (10%, aq) solution was added. The heterogeneous mixture was stirred for 15 mins vigorously. The organic and aqueous layer were separated. The aqueous layer was extracted with dichloromethane three times. The combined organic layer was dried over Na_2SO_4 and filtered. The expected product was formed in 91% from ^{1}H NMR spectroscopic analysis of the crude mixture.

Several follow up experiments with modifications are shown in the summary table below:

Experiment	Modification	Yield /%
**A**	None	91
**B**	No TBSOTf added	82
**C**	copper iodide (30 mg, 0.16 mmol, 0.4 equiv) added to amine mixture	100
**D**	copper iodide (30 mg, 0.16 mmol, 0.4 equiv) added to amine mixture and no TBSOTf was added	61

10.2.4 Attempted preparation of alkylzinc from redox active ester

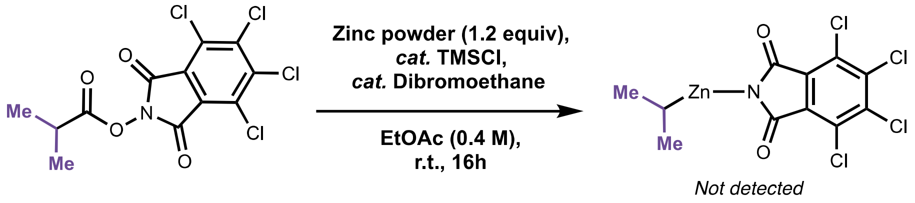

Zinc (26 mg, 0.4 mmol, 1 equiv) was added to a dry round bottom flask. EtOAc (1 mL) was added. 1 drop of dibromoethane was added. The mixture was stirred at 65 °C for 3 min. After cooling to rt, TMSCl (1 drop) was added. The mixture was stirred at r.t. for 15 mins. 4,5,6,7-tetrachloro-1,3-dioxoisoindolin-2-yl isobutyrate (148 mg, 0.4 mmol, 1 equiv) was added. The mixture was stirred at rt overnight. Stirring was stopped to let remaining zinc settle. The supernatant solution was transferred to a dry round bottom flask. I_2 was not discolourised upon addition of this solution, indicating that no alkylzinc was formed. To mimic the conditions of the zinc-mediated CAA, the experiment was repeated with the addition of TMSOTf (217 µL, 0.4 mmol, 1 equiv) and the use of excess zinc (39 mg, 0.6 mmol, 1 equiv). The resulting solution also did not discolorise I_2. Despite this result, this solution was added to the iminium formed from N-methylbenzylamine and hydrocinnamaldehyde as previously described. No CAA product was not detected with ^{1}H NMR spectroscopy after workup.

10.2.5 Reactivity of secondary alkyl-zinc with ketimines

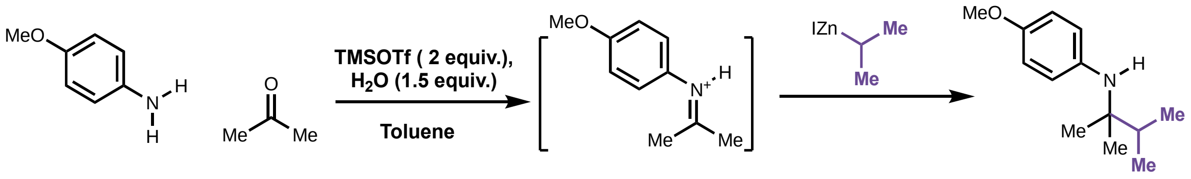

A Biotage Microwave Process microwave vial (10 mL) was sealed, put under vacuum and refilled with N_2. In the vial, p-anisidine (49 mg, 0.4 mmol, 1 equiv) was dissolved in toluene (0.1 mL). Acetone (35 µL, 0.6 mmol, 1.5 equiv) was added, followed by H_2O (11 µL, 0.04 mmol, 1.5 equiv), TMSOTf (109 µL, 0.6 mmol, 1.5 equiv). The mixture was allowed to stir for 15 mins. The isopropylzinc iodide solution, prepared according to General Procedure **L**, (2.4 M, 0.50 mL, 1.2 mmol, 3 equiv) in toluene was added. The mixture was stirred overnight. The mixture was diluted with dichloromethane. NaOH (10%, aq) solution was added. The heterogeneous mixture was stirred for 15 mins vigorously. The organic and aqueous layer were separated. The aqueous layer was extracted with dichloromethane three times. The combined organic layer was dried over Na_2SO_4 and filtered. The expected product was observed formed from ^{1}H NMR spectroscopic analysis of the crude mixture.

ii) The processed was repeated with N-Cbz piperidone (140 mg, 0.6 mmol, 1.5 equiv) as the ketone. The expected product was not observed from ^{1}H NMR spectroscopic analysis of the crude mixture.

10.3 Formation of Alkyl-zincs under standard reaction conditions

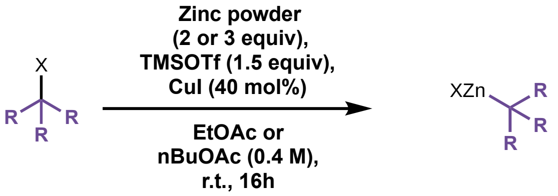

Reactions were conducted according to the corresponding general procedure with the amine and aldehyde removed (with modifications as stated). After the reaction time was completed, the stirring was stopped, and supernatant was used to determine the concentration of organometallic iodometric titration.

Reaction	Iodide or Bromide	General Procedure	Alkyl substituent	copper iodide added	Volume required to quench iodine (iodine scale specified)	Concentration / M
A	Iodide	**E**	primary	No	1.2 mL for 0.2 mmol	0.167
B	Iodide	**E**	primary	Yes	1.0 mL for 0.2 mmol	0.2
C	Iodide	**B**	secondary	No	0.95 mL for 0.2 mmol	0.21
D	Iodide*	**E**	tertiary	No	Full volume did not quench solution (0.2 mmol)	-
E	Iodide*	**E**	tertiary	Yes	Full volume did not quench solution (0.2 mmol)	-
F	Iodide	**E**	tertiary	No	Full volume did not quench solution (0.1 mmol)	-
G	Iodide	**E**	tertiary	Yes	Full volume did not quench solution (0.1 mmol)	-
H	Bromide	**E**	tertiary	Yes	Full volume did not quench solution (0.1 mmol)	-
I	Redox active ester	**G**	secondary	No	Full volume did not quench solution (0.05 mmol)	-
*-TMSOTF (1.5 equiv) added to mixture.						

10.4 Radical trapping experiments

10.4.1 Radical trapping experiments summary

TEMPO, styrene and 1,1-diphenylethylene were used as radical traps. It is important to note:

1. TEMPO is known to react with organozincs to produce the same product as the radical adduct.[17,18,19] Furthermore, it is known to complex alkyl zincs into conglomerate species.[20]

2. Styrene and 1,1-diphenylethylene do not suffer from reactivity with alkyl zinc species unlike many common radical traps (e.g. giese acceptors). However, they show considerably slower reactivity rates with alkyl radicals (in particular primary radicals) which means their reaction rates maybe slower than addition to the iminium.[21,22,23,24]

Table summarising all trapping experiments conducted.

Exp	Iminium type	Feedstock source	Alkyl substitution	Trapping Reagent	Yield /%
**A**	Iminium	Iodide	Primary	TEMPO	Trace
**B**	Iminium	Iodide	Primary	styrene	100
**C**	Iminium	Iodide	Primary	1,1-diphenylethylene	100
**D**	Iminium	Iodide	Secondary	TEMPO	Trace
**E**	Iminium	Iodide	Secondary	styrene	100
**F**	Iminium	Iodide	Secondary	1,1-diphenylethylene	40
**G**	Iminium	Iodide	Tertiary	TEMPO	Trace
**H**	Iminium	Iodide	Tertiary	styrene	36
**I**	Iminium	Iodide	Tertiary	1,1-diphenylethylene	45
**J**	Iminium	RAE	Secondary	TEMPO	0
**K**	Iminium	RAE	Secondary	styrene	0
**L**	Iminium	RAE	Secondary	1,1-diphenylethylene	0
**M**	Ketiminium	Iodide	Secondary	TEMPO	Trace
**N**	Ketiminium	Iodide	Secondary	styrene	74
**O**	Ketiminium	Iodide	Secondary	1,1-diphenylethylene	46

10.4.2 Radical trapping experiments procedures

1° TEMPO

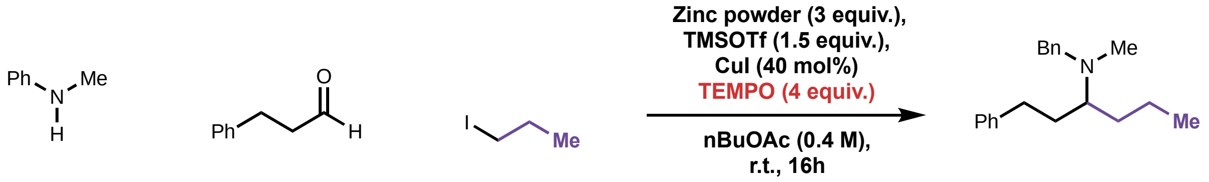

An oven dried vial (Biotage Microwave Process Vial (10 mL), equipped with gas-tight pierceable caps) was charged with a stirring bar, Zn dust (78 mg, 1.2 mmol, 3 equiv), copper iodide (30 mg, 0.16 mmol, 40 mol%) and TEMPO (250 mg, 1.6 mmol, 4 equiv). The vial was sealed, evacuated and backfilled with N_2. To this was added *n*BuOAc (1 mL), followed by the N-methylbenzylamine (52 µL, 0.4 mmol, 1.0 equiv) and hydrocinnamaldehyde (108 µL, 0.8 mmol, 2 equiv). The mixture was stirred for 15 minutes then, whilst stirring, 1-iodopropane (156 µL, 1.6 mmol, 4 equiv), and TMSOTf (106 µL, 0.6 mmol, 1.5 equiv) were added. The reaction mixture was stirred overnight. The crude mixture was transferred into a 50 ml round bottom flask and diluted with dichloromethane (approximately 20 mL). NaOH (approximately 15 ml, 10% aq.) was added. The heterogenous mixture was vigorously stirred for 10-15 min. The aqueous layer was extracted with dichloromethane three times. The combined organic phases were washed with sat. $Na_2S_2O_3$ with 5 drops of 3 M HCl until the solution transformed from orange/red to yellow/colourless. The combined organic layer was dried over anhydrous $MgSO_4$. The solvent was removed *in vacuo* and the reaction was analysed by LCMS and ^{1}H NMR. Trace product was detected by LCMS and ^{1}H NMR. A significant amount of the propyl TEMPO adduct was detected by LCMS.

1° Styrene

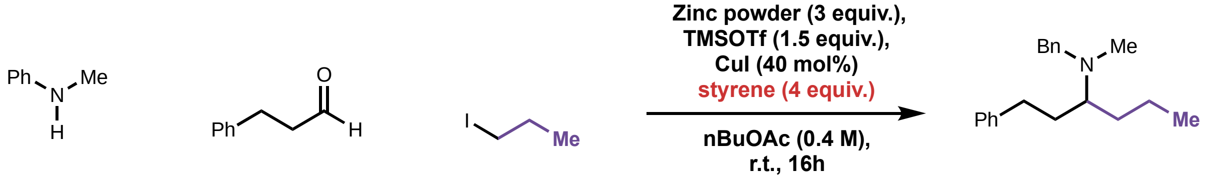

An oven dried vial (Biotage Microwave Process Vial (10 mL), equipped with gas-tight pierceable caps) was charged with a stirring bar, Zn (78 mg, 1.2 mmol, 3 equiv) and copper iodide (30 mg, 0.16 mmol, 40 mol%). The vial was sealed, evacuated and backfilled with N_2. To this was added *n*BuOAc (1 mL), followed by the N-methylbenzylamine (52 µL, 0.4 mmol, 1.0 equiv), hydrocinnamaldehyde (108 µL, 0.8 mmol, 2 equiv) and styrene (183 µL, 1.6 mmol, 4 equiv). The mixture was stirred for 15 minutes then, whilst stirring, 1-iodopropane (156 µL, 1.6 mmol, 4 equiv), and TMSOTf (106 µL, 0.6 mmol, 1.5 equiv) were added. The reaction mixture was stirred overnight. The crude mixture was transferred into a 50 ml round bottom flask and diluted with dichloromethane (approximately 20 ml). NaOH (approximately 15 ml, 10% aq.) was added. The heterogenous mixture was vigorously stirred for 10-15 min. The aqueous layer was extracted with dichloromethane three times. The combined organic layer was dried over anhydrous $MgSO_4$. The solvent was removed *in vacuo* and the reaction was analysed by ^{1}H NMR using 1,1,2,2-tetrachloroethane as an internal standard displaying a quantitative crude yield.

1° 1,1-diphenylethylene

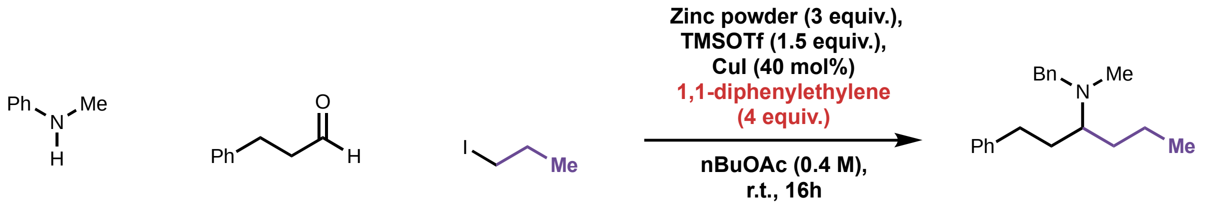

An oven dried vial (Biotage Microwave Process Vial (10 mL), equipped with gas-tight pierceable caps) was charged with a stirring bar, Zn (78 mg, 1.2 mmol, 3 equiv) and copper iodide (30 mg, 0.16 mmol, 40 mol%). The vial was sealed, evacuated and backfilled with N_2. To this was added *n*BuOAc (1 mL), followed by the N-methylbenzylamine (52 µL, 0.4 mmol, 1.0 equiv), hydrocinnamaldehyde (108 µL, 0.8 mmol, 2 equiv) and 1,1-diphenylethylene (283 µL, 1.6 mmol, 4 equiv). The mixture was stirred for 15 minutes then, whilst stirring, 1-iodopropane (156 µL, 1.6 mmol, 4 equiv), and TMSOTf (106 µL, 0.6 mmol, 1.5 equiv) were added. The reaction mixture was stirred overnight. The crude mixture was transferred into a 50 ml round bottom flask and diluted with dichloromethane (approximately 20 ml). NaOH (approximately 15 ml, 10% aq.) was added. The heterogenous mixture was vigorously stirred for 10-15 min. The aqueous layer was extracted with dichloromethane three times. The combined organic layer was dried over anhydrous $MgSO_4$. The solvent was removed *in vacuo* and the reaction was analysed by ^1H NMR using 1,1,2,2-tetrachloroethane as an internal standard displaying a quantitative crude yield.

2° TEMPO

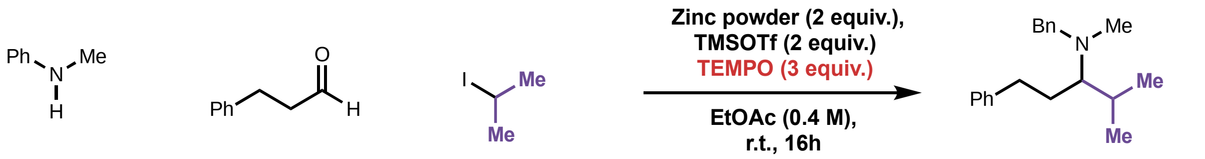

An oven dried vial (Biotage Microwave Process Vial (10 mL), equipped with gas-tight pierceable caps) was charged with a stirring bar and Zn (52 mg, 0.8 mmol, 2 equiv) and TEMPO (188 mg, 1.2 mmol, 3 equiv). The vial was sealed, evacuated and backfilled with N_2. To this was added EtOAc (1 mL), followed by the N-methylbenzylamine (52 µL, 0.4 mmol, 1.0 equiv), hydrocinnamaldehyde (108 µL, 0.8 mmol, 2 equiv) and 2-iodopropane (120 µL, 1.2 mmol, 3 equiv). The reaction mixture was stirred for one minute, followed by the addition of TMSOTf (145 µL, 0.4 mmol, 2.0 equiv). The reaction mixture was stirred overnight. The crude mixture was transferred into a 50 ml round bottom flask and diluted with dichloromethane (approximately 20 ml). NaOH (approximately 15 ml, 10% aq.) was added. The heterogenous mixture was vigorously stirred for 10-15 min. The aqueous layer was extracted with dichloromethane three times. The combined organic phases were washed with sat. $Na_2S_2O_3$ with 5 drops of 3 M HCl until the solution transformed from orange/red to yellow/colourless. The combined organic layer was dried over anhydrous $MgSO_4$. The solvent was removed *in vacuo* and the reaction was analysed by LCMS and ^1H NMR. Trace product was detected by LCMS and ^1H NMR. A significant amount of the isopropyl TEMPO adduct was detected by LCMS.

2° Styrene

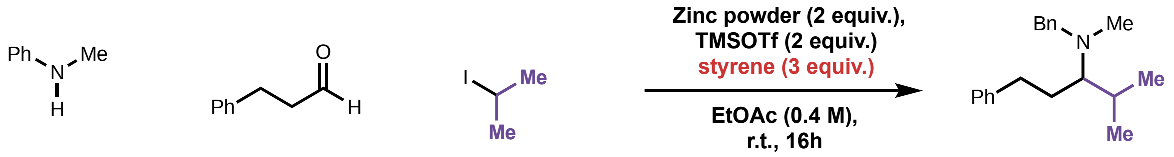

An oven dried vial (Biotage Microwave Process Vial (10 mL), equipped with gas-tight pierceable caps) was charged with a stirring bar and Zn dust (52 mg, 0.8 mmol, 2 equiv) and TEMPO (188 mg, 1.2 mmol, 3 equiv). The vial was sealed, evacuated and backfilled with N_2. To this was added EtOAc (1 mL), followed by the N-methylbenzylamine (52 µL, 0.4 mmol, 1.0 equiv), hydrocinnamaldehyde (108 µL, 0.8 mmol, 2 equiv), styrene (137 µL, 1.2 mmol, 3 equiv) and 2-iodopropane (120 µL, 1.2 mmol, 3 equiv). The reaction mixture was stirred for one minute, followed by the addition of TMSOTf (145 µL, 0.4 mmol, 2.0 equiv). The reaction mixture was stirred overnight. The crude mixture was transferred into a 50 ml round bottom flask and diluted with dichloromethane (approximately 20 ml). NaOH (approximately 15 ml, 10% aq.) was added. The heterogenous mixture was vigorously stirred for 10-15 min. The aqueous layer was extracted with dichloromethane three times. The combined organic layer was dried over anhydrous $MgSO_4$ or Na_2SO_4. The solvent was removed *in vacuo* and the reaction was analysed by ^{1}H NMR using 1,1,2,2-tetrachloroethane as an internal standard displaying a quantitative crude yield.

2° 1,1-diphenylethylene

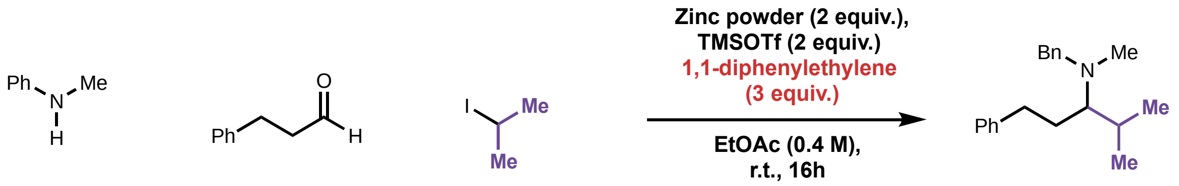

An oven dried vial (Biotage Microwave Process Vial (10 mL), equipped with gas-tight pierceable caps) was charged with a stirring bar and Zn dust (52 mg, 0.8 mmol, 2 equiv) and TEMPO (188 mg, 1.2 mmol, 3 equiv). The vial was sealed, evacuated and backfilled with N_2. To this was added EtOAc (1 mL), followed by the N-methylbenzylamine (52 µL, 0.4 mmol, 1.0 equiv), hydrocinnamaldehyde (108 µL, 0.8 mmol, 2 equiv), 1,1-diphenylethylene (212 µL, 1.2 mmol, 3 equiv) and 2-iodopropane (120 µL, 1.2 mmol, 3 equiv). The reaction mixture was stirred for one minute, followed by the addition of TMSOTf (145 µL, 0.4 mmol, 2.0 equiv). The reaction mixture was stirred overnight. The crude mixture was transferred into a 50 ml round bottom flask and diluted with dichloromethane (approximately 20 ml). NaOH (approximately 15 ml, 10% aq.) was added. The heterogenous mixture was vigorously stirred for 10-15 min. The aqueous layer was extracted with dichloromethane three times. The combined organic layer was dried over anhydrous $MgSO_4$. The solvent was removed *in vacuo* and the reaction was analysed by ^{1}H NMR using 1,1,2,2-tetrachloroethane as an internal standard displaying a 44% crude yield.

3° TEMPO

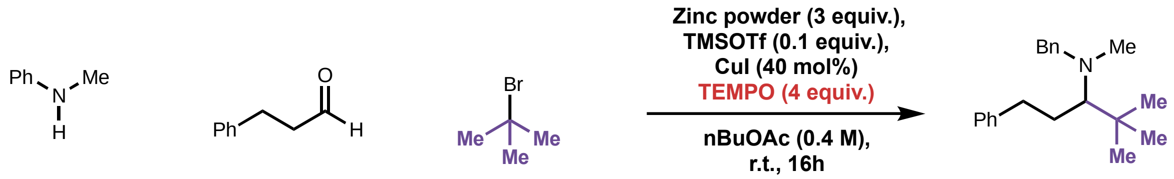

An oven dried vial (Biotage Microwave Process Vial (10 mL), equipped with gas-tight pierceable caps) was charged with a stirring bar, Zn dust (78 mg, 1.2 mmol, 3 equiv), copper iodide (30 mg, 0.16 mmol, 40 mol%) and TEMPO (250 mg, 1.6 mmol, 4 equiv). The vial was sealed, evacuated and backfilled with N_2. To this was added *n*BuOAc (1 mL), followed by the N-methylbenzylamine (52 µL, 0.4 mmol, 1.0 equiv) and hydrocinnamaldehyde (108 µL, 0.8 mmol, 2 equiv). The mixture was stirred for 15 minutes then, whilst stirring, tert-butyl bromide (180 µL, 1.6 mmol, 4 equiv) was added. The reaction mixture was stirred overnight. The crude mixture was transferred into a 50 ml round bottom flask and diluted with dichloromethane (approximately 20 ml). NaOH (approximately 15 ml, 10% aq.) was added. The heterogenous mixture was vigorously stirred for 10-15 min. The aqueous layer was extracted with dichloromethane three times. The combined organic phases were washed with sat. $Na_2S_2O_3$ with 5 drops of 3 M HCl until the solution transformed from orange/red to yellow/colourless. The combined organic layer was dried over anhydrous $MgSO_4$. The solvent was removed *in vacuo* and the reaction was analysed by LCMS and ^{1}H NMR. Trace product was detected by LCMS and ^{1}H NMR. A significant amount of the tert-butyl TEMPO adduct was detected by LCMS.

3° Styrene

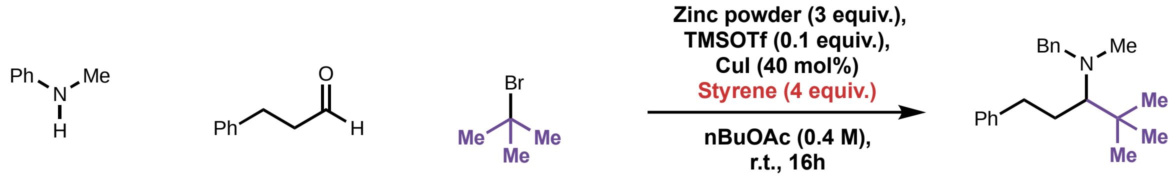

An oven dried vial (Biotage Microwave Process Vial (10 mL), equipped with gas-tight pierceable caps) was charged with a stirring bar, Zn dust (78 mg, 1.2 mmol, 3 equiv) and copper iodide (30 mg, 0.16 mmol, 40 mol%). The vial was sealed, evacuated and backfilled with N_2. To this was added *n*BuOAc (1 mL), followed by the N-methylbenzylamine (52 µL, 0.4 mmol, 1.0 equiv), hydrocinnamaldehyde (108 µL, 0.8 mmol, 2 equiv) and styrene (183 µL, 1.6 mmol, 4 equiv). The mixture was stirred for 15 minutes then, whilst stirring, tert-butyl bromide (180 µL, 1.6 mmol, 4 equiv) was added. The reaction mixture was stirred overnight. The crude mixture was transferred into a 50 ml round bottom flask and diluted with dichloromethane (approximately 20 ml). NaOH (approximately 15 ml, 10% aq.) was added. The heterogenous mixture was vigorously stirred for 10-15 min. The aqueous layer was extracted with dichloromethane three times. The combined organic layer was dried over anhydrous $MgSO_4$. The solvent was removed *in vacuo* and the reaction was analysed by ^{1}H NMR using 1,1,2,2-tetrachloroethane as an internal standard displaying a 36% crude yield.

3° 1,1-diphenylethylene

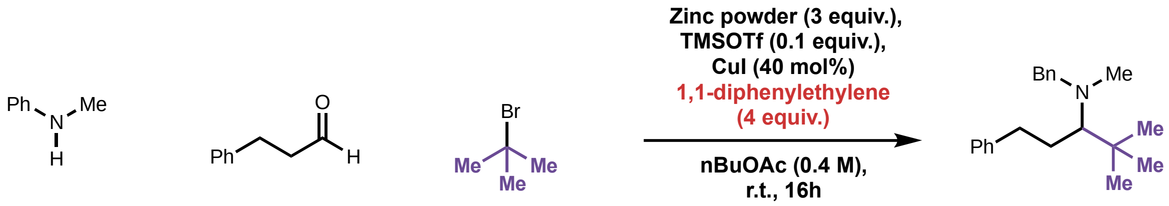

An oven dried vial (Biotage Microwave Process Vial (10 mL), equipped with gas-tight pierceable caps) was charged with a stirring bar, Zn dust (78 mg, 1.2 mmol, 3 equiv) and copper iodide (30 mg, 0.16 mmol, 40 mol%). The vial was sealed, evacuated and backfilled with N_2. To this was added *n*BuOAc (1 mL), followed by the N-methylbenzylamine (52 µL, 0.4 mmol, 1.0 equiv), hydrocinnamaldehyde (108 µL, 0.8 mmol, 2 equiv) and 1,1-diphenylethylene (283 µL, 1.6 mmol, 4 equiv). The mixture was stirred for 15 minutes then, whilst stirring, tert-butyl bromide (180 µL, 1.6 mmol, 4 equiv) was added. The reaction mixture was stirred overnight. The crude mixture was transferred into a 50 ml round bottom flask and diluted with dichloromethane (approximately 20 ml). NaOH (approximately 15 ml, 10% aq.) was added. The heterogenous mixture was vigorously stirred for 10-15 min. The aqueous layer was extracted with dichloromethane three times. The combined organic layer was dried over anhydrous $MgSO_4$. The solvent was removed *in vacuo* and the reaction was analysed by ^1H NMR using 1,1,2,2-tetrachloroethane as an internal standard displaying a 45% crude yield.

RAE TEMPO

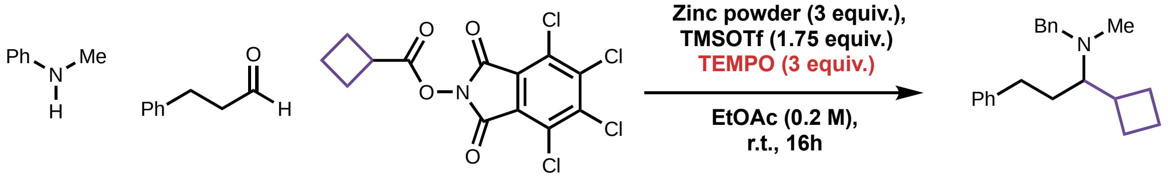

An oven dried vial (Biotage Microwave Process Vial 10 mL, equipped with gas-tight pierceable caps) was charged with a stirring bar. The vial was sealed, evacuated and backfilled with N_2. To this was added EtOAc (0.5 mL), followed by N-methylbenzylamine (13 µL, 0.1 mmol, 1 equiv), hydrocinnamaldehyde (26 µL, 0.2 mmol, 2 equiv), TEMPO (188 mg, 1.2 mmol, 3 equiv) and TMSOTf (32 µL, 0.175 mmol, 1.75 equiv). The mixture was stirred for 15 mins. The cap was removed. Zinc (20 mg, 0.3 mmol, 3 equiv) and the 4,5,6,7-tetrachloro-1,3-dioxoisoindolin-2-yl cyclobutanecarboxylate (77 mg, 0.2 mmol, 2 equiv) were added in one portion. The microwave vial was sealed, put under vacuum and refilled with N_2. The mixture was stirred overnight. The crude mixture was transferred into a 50 ml round bottom flask and diluted with dichloromethane (approximately 20 ml). NaOH (approximately 15 ml, 10% aq.) was added. The heterogenous mixture was vigorously stirred for 10-15 min. The aqueous layer was extracted with dichloromethane three times. The combined organic phases were washed with sat. $Na_2S_2O_3$ with 5 drops of 3 M HCl until the solution transformed from orange/red to yellow/colourless. The combined organic layer was dried over anhydrous $MgSO_4$. The solvent was removed *in vacuo* and the reaction was analysed by LCMS and ^1H NMR. No product was detected by LCMS and ^1H NMR. A significant amount of the cyclobutyl TEMPO adduct was detected by LCMS.

RAE Styrene

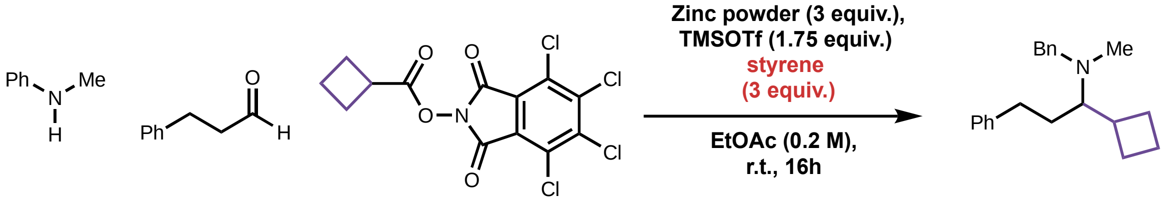

An oven dried vial (Biotage Microwave Process Vial 10 mL, equipped with gas-tight pierceable caps) was charged with a stirring bar. The vial was sealed, evacuated and backfilled with N_2. To this was added EtOAc (0.5 mL), followed by N-methylbenzylamine (13 µL, 0.1 mmol, 1 equiv), hydrocinnamaldehyde (26 µL, 0.2 mmol, 2 equiv), styrene (137 µL, 1.2 mmol, 3 equiv) and TMSOTf (32 µL, 0.175 mmol, 1.75 equiv). The mixture was stirred for 15 mins. The cap was removed. Zinc (20 mg, 0.3 mmol, 3 equiv) and the 4,5,6,7-tetrachloro-1,3-dioxoisoindolin-2-yl cyclobutanecarboxylate (77 mg, 0.2 mmol, 2 equiv) were added in one portion. The microwave vial was sealed, put under vacuum and refilled with N_2. The mixture was stirred overnight. The crude mixture was transferred into a 50 ml round bottom flask and diluted with dichloromethane (approximately 20 ml). NaOH (approximately 15 ml, 10% aq.) was added. The heterogenous mixture was vigorously stirred for 10-15 min. The aqueous layer was extracted with dichloromethane three times. The combined organic layer was dried over anhydrous $MgSO_4$. The solvent was removed *in vacuo* and the reaction was analysed by ^{1}H NMR using 1,1,2,2-tetrachloroethane as an internal standard displaying a 0% crude yield.

RAE 1,1-diphenylethylene

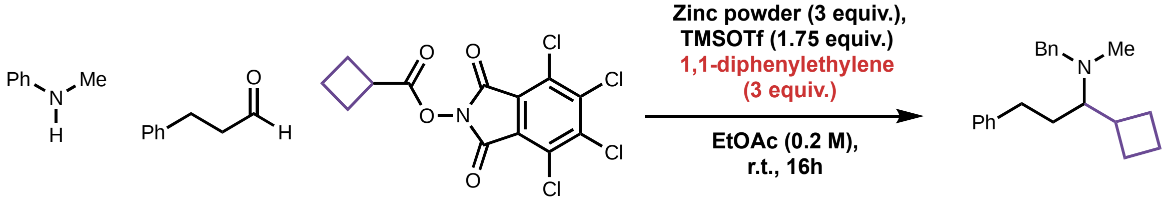

An oven dried vial (Biotage Microwave Process Vial 10 mL, equipped with gas-tight pierceable caps) was charged with a stirring bar. The vial was sealed, evacuated and backfilled with N_2. To this was added EtOAc (0.5 mL), followed by N-methylbenzylamine (13 µL, 0.1 mmol, 1 equiv), hydrocinnamaldehyde (26 µL, 0.2 mmol, 2 equiv), 1,1-diphenylethylene (212 µL, 1.2 mmol, 3 equiv) and TMSOTf (32 µL, 0.175 mmol, 1.75 equiv). The mixture was stirred for 15 mins. The cap was removed. Zinc (20 mg, 0.3 mmol, 3 equiv) and the 4,5,6,7-tetrachloro-1,3-dioxoisoindolin-2-yl cyclobutanecarboxylate (77 mg, 0.2 mmol, 2 equiv) were added in one portion. The microwave vial was sealed, put under vacuum and refilled with N_2. The mixture was stirred overnight. The crude mixture was transferred into a 50 ml round bottom flask and diluted with dichloromethane (approximately 20 ml). NaOH (approximately 15 ml, 10% aq.) was added. The heterogenous mixture was vigorously stirred for 10-15 min. The aqueous layer was extracted with dichloromethane three times. The combined organic layer was dried over anhydrous $MgSO_4$. The solvent was removed *in vacuo* and the reaction was analysed by ^{1}H NMR using 1,1,2,2-tetrachloroethane as an internal standard displaying a 0% crude yield.

Ketone TEMPO

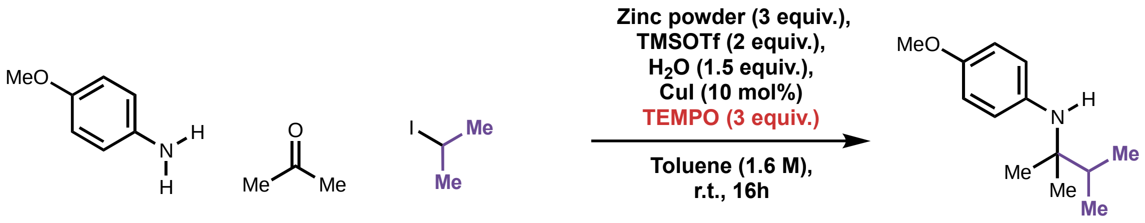

An oven dried vial (Biotage Microwave Process Vial 10 mL, equipped with gas-tight pierceable caps) was charged with a stirring bar, the *p*-anisidine (49 mg, 0.4 mmol, 1 equiv), Zn dust (79 mg, 1.2 mmol, 3 equiv), TEMPO (188 mg, 1.2 mmol, 3 equiv) and copper iodide (7.5 mg, 0.04 mmol, 10 mol%). The vial was sealed, evacuated and backfilled with N_2. To this was added toluene (0.25 mL). While stirring, the acetone (44 µL, 0.6 mmol, 1.5 equiv) was added dropwise, followed by the 2-iodopropane (120 µL, 1.2 mmol, 3 equiv), H_2O (11 µL, 0.6 mmol, 1.5 equiv) and then TMSOTf (109 µL, 0.6 mmol, 1.5 equiv). The mixture was stirred overnight. The mixture was diluted with dichloromethane and basified with NaOH (10%, aq). The heterogenous mixture was stirred vigorously for 15 mins. The organic and aqueous layers were separated. The aqueous layer was extracted with dichloromethane twice. The combined organic layer was dried over anhydrous $MgSO_4$. The solvent was removed *in vacuo* and the reaction was analysed by LCMS and ^{1}H NMR. Trace product was detected by LCMS but not ^{1}H NMR. A significant amount of the isopropylTEMPO adduct was detected by LCMS.

Ketone Styrene

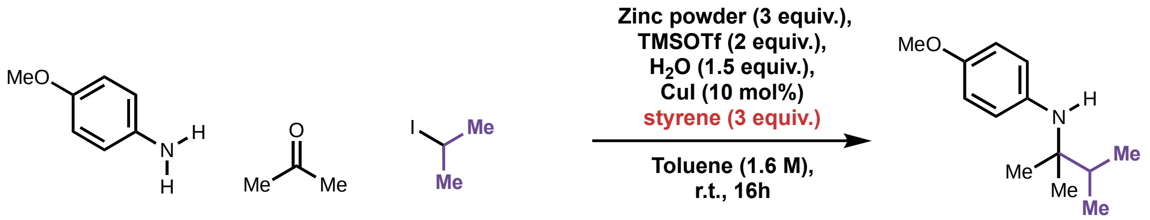

An oven dried vial (Biotage Microwave Process Vial 10 mL, equipped with gas-tight pierceable caps) was charged with a stirring bar, the *p*-anisidine (49 mg, 0.4 mmol, 1 equiv), Zn dust (79 mg, 1.2 mmol, 3 equiv) and copper iodide (7.5 mg, 0.04 mmol, 10 mol%). The vial was sealed, evacuated and backfilled with N_2. To this was added toluene (0.25 mL). While stirring, the acetone (44 µL, 0.6 mmol, 1.5 equiv) was added dropwise, followed by the 2-iodopropane (120 µL, 1.2 mmol, 3 equiv), styrene (137 µL, 1.2 mmol, 3 equiv), H_2O (11 µL, 0.6 mmol, 1.5 equiv) and then TMSOTf (109 µL, 0.6 mmol, 1.5 equiv). The mixture was stirred overnight. The mixture was diluted with dichloromethane and basified with NaOH (10%, aq). The heterogenous mixture was stirred vigorously for 15 mins. The organic and aqueous layers were separated. The aqueous layer was extracted with dichloromethane twice. The combined organic layer was dried over $MgSO_4$. The solvent was removed *in vacuo* and the reaction was analysed by ^{1}H NMR using 1,1,2,2-tetrachloroethane as an internal standard displaying a 74% crude yield.

Ketone 1,1-diphenylethylene

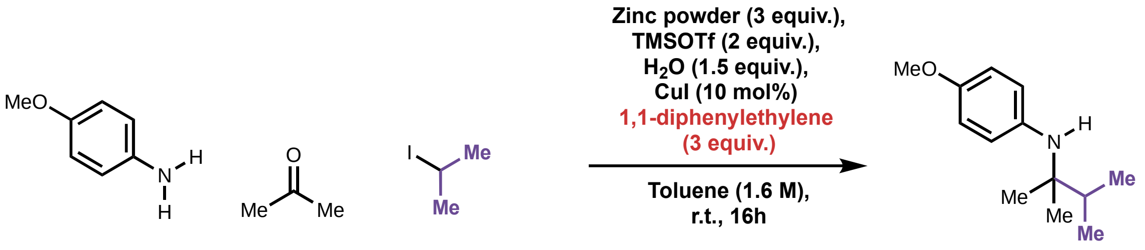

An oven dried vial (Biotage Microwave Process Vial 10 mL, equipped with gas-tight pierceable caps) was charged with a stirring bar, the *p*-anisidine (49 mg, 0.4 mmol, 1 equiv), Zn dust (79 mg, 1.2 mmol, 3 equiv) and copper iodide (7.5 mg, 0.04 mmol, 10 mol%). The vial was sealed, evacuated and backfilled with N_2. To this was added toluene (0.25 mL). While stirring, the acetone (44 µL, 0.6 mmol, 1.5 equiv) was added dropwise, followed by the 2-iodopropane (120 µL, 1.2 mmol, 3 equiv), 1,1-diphenylethylene (212 µL, 1.2 mmol, 3 equiv), H_2O (11 µL, 0.6 mmol, 1.5 equiv) and then TMSOTf (109 µL, 0.6 mmol, 1.5 equiv). The mixture was stirred overnight. The mixture was diluted with dichloromethane and basified with NaOH (10%, aq). The heterogenous mixture was stirred vigorously for 15 mins. The organic and aqueous layers were separated. The aqueous layer was extracted with dichloromethane twice. The combined organic layer was dried over $MgSO_4$. The solvent was removed *in vacuo* and the reaction was analysed by ^{1}H NMR using 1,1,2,2-tetrachloroethane as an internal standard displaying a 46% crude yield.

10.5 Radical clock experiment

Recent approaches to determining alkyl zinc halide addition vs radical addition have used the ring opening of a cyclopropyl by the intermediate radical aminyl cation.[25] We undertook this study and found that mixtures of ring opened and cyclopropyl products were observed. However, the starting material was found to be partially unstable (23% degradation over the reaction time) under the reaction conditions. ***Therefore, the results of these experiments are not accurate.***

10.5.1 Stability of amine under reaction conditions

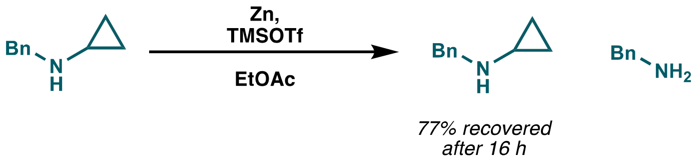

An oven dried vial (Biotage Microwave Process Vial (10 mL), equipped with gas-tight pierceable caps) was charged with a stirring bar and Zn dust (39 mg, 0.6 mmol, 3 equiv). The vial was sealed, evacuated and backfilled with N_2. To this was added EtOAc (1 mL) , followed by *N*-benzylcyclopropylamine (29 mg, 0.2 mmol, 1 equiv) The reaction mixture was stirred for 10s, followed by the addition of TMSOTf (54 µL, 0.3 mmol, 1.5 equiv). The reaction mixture was stirred overnight. The crude mixture was transferred into a 50 ml round bottom flask and diluted with dichloromethane (approximately 20 ml). NaOH (approximately 15 ml, 10% aq.) was added. The heterogenous mixture was vigorously stirred for 10 min.

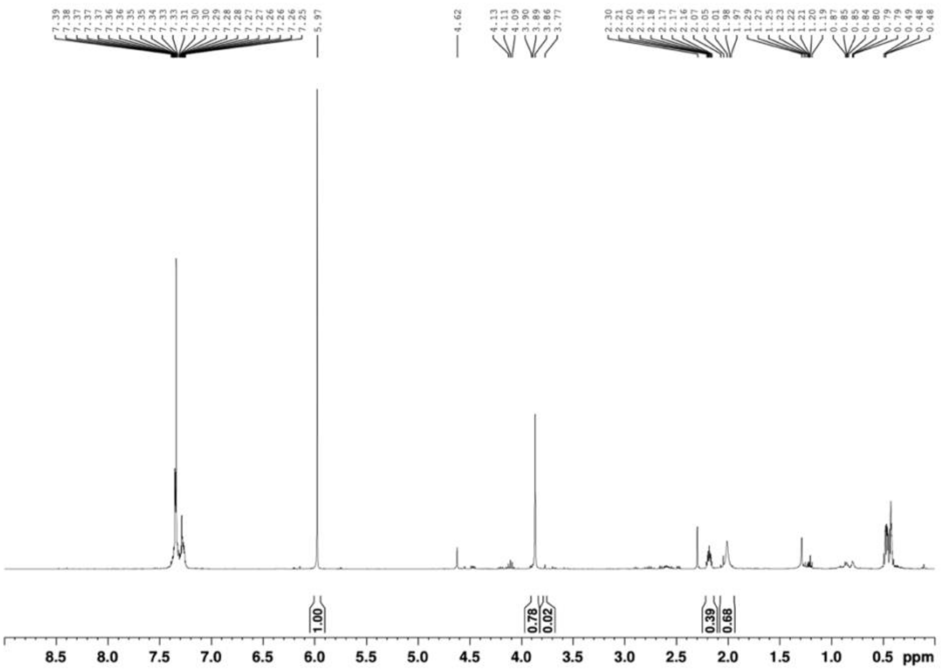

The aqueous layer was extracted with dichloromethane three times. The combined organic layer was dried over anhydrous $MgSO_4$. Solvent was evaporated *in vacuo*. Yield of the recovered product was determined by ^{1}H NMR using 1,1,2,2-tetrachloroethane as internal standard indicating a 72% yield based on the heptet peak at 2.18 ppm (spectrum shown below).

10.5.1 Primary alkyl halide ring opening (*inaccurate due to degradation*)

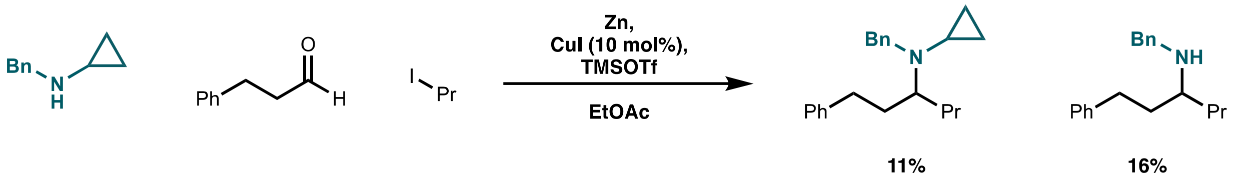

An oven dried vial (Biotage Microwave Process Vial (10 mL), equipped with gas-tight pierceable caps) was charged with a stirring bar, copper iodide (15 mg, 0.08 mmol, 0.4 equiv) and Zn (39 mg, 0.6 mmol, 3 equiv). The vial was sealed, evacuated and backfilled with N_2. To this was added *n*BuOAc (0.25 mL), followed by *N*-benzylcyclopropylamine (29 mg, 0.2 mmol, 1 equiv) and hydrocinnamaldehyde (108 µL, 0.3 mmol, 1.5 equiv). The mixture was stirred for 15 min. 1-iodopropane (156 µL, 1.6 mmol, 4 equiv) was added. The reaction mixture was stirred for 10s, followed by the addition of TMSOTf (54 µL, 0.3 mmol, 1.5 equiv). The reaction mixture was stirred overnight. The crude mixture was transferred into a 50 ml round bottom flask and diluted with dichloromethane (approximately 20 ml). NaOH (approximately 15 ml, 10% aq.) was added. The heterogenous mixture was vigorously stirred for 10 min. The aqueous layer was extracted with dichloromethane three times. The combined organic layer was dried over anhydrous $MgSO_4$. Solvent was evaporated *in vacuo*. The mixture was purified by column chromatography (3%–80% Et_2O in Pet. Ether) to afford *N*-benzyl-*N*-(1-phenylpentan-3-yl)cyclopropanamine (colourless oil, 6.8 mg, 11%) and *N*-benzyl-1-phenylpentan-3-amine (colourless oil, 8.7 mg, 16%).

N-benzyl-N-(1-phenylpentan-3-yl)cyclopropanamine

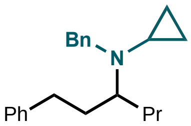

^{1}H NMR (400 MHz, $CDCl_3$): δ 7.32 - 7.14 (m, 10H), 3.77 (d, J = 13.7 Hz, 1H), 3.71 (d, J = 13.7 Hz, 1H), 2.74 (ddd, J = 15.8, 10.4, 5.6 Hz, 1H), 2.63 (quint, J = 6.5 Hz, 1H), 2.53 (ddd, J = 15.8, 10.5, 5.8 Hz, 1H), 2.08 - 2.01 (m, 1H), 1.96 - 1.85 (m, 1H), 1.72 - 1.59 (m, 2H), 1.41 - 1.25 (m, 3H), 0.88 (t, J = 7.1 Hz, 3H), 0.39 - 0.33 (m, 2H), 0.29 - 0.23 (m, 2H).

^{13}C NMR (100 MHz, $CDCl_3$): δ 143.3, 141.6, 129.2, 128.5, 128.4, 128.0, 126.5, 125.7, 61.1, 56.0, 33.9, 33.7, 33.1, 32.8, 20.6, 14.4, 7.6, 7.0.

IR (film, cm^{-1}): 3062, 3026, 2954, 2929, 2869, 1603, 1495, 1453, 1377, 1346, 1018, 749, 715, 697.

HRMS: m/z calcd for $C_{22}H_{29}N$ $[M+H]^+$ 308.2373, found 308.2379.

N-benzyl-1-phenylpentan-3-amine

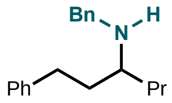

^{1}H NMR (500 MHz, $CDCl_3$): δ 7.34 - 7.31 (m, 4H), 7.30 - 7.23 (m, 3H), 7.20 - 7.16 (m, 3H), 3.76 (s, 2H), 2.69 - 2.59 (m, 3H), 1.79 - 1.72 (m, 2H), 1.53 - 1.30 (m, 5H), 0.90 (t, J = 7.3 Hz, 3H)

^{13}C NMR (125 MHz, $CDCl_3$): δ 142.9, 141.2, 128.49, 128.45, 128.3, 126.9, 125.8, 56.3, 51.2, 36.5, 36.0, 32.1, 19.1, 14.5.

IR (film, cm^{-1}): 3340 (br), 3026, 2955, 2926, 2854, 1602, 1495, 1454, 1072, 1029, 908, 734, 698.

HRMS: m/z calcd for $C_{19}H_{25}N$ $[M+H]^+$ 268.2060, found 268.2065

10.5.1 Secondary alkyl halide ring opening (*inaccurate due to degradation*)

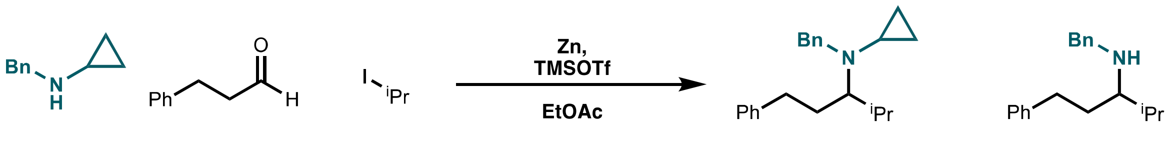

N-benzylcyclopropylamine (29 mg, 0.2 mmol, 1 equiv) and zinc (26 mg, 0.4 mmol, 2 equiv) were added to a Biotage Microwave Process microwave vial (10 mL). The vial was sealed, put under vacuum and refilled with N_2. EtOAc (1 mL) was added. While stirring, hydrocinnamaldehyde (40 µL, 0.3 mmol, 1.5 equiv), 2-iodopropane (40 µL, 0.4 mmol, 2 equiv) and TMSOTf (72 µL, 0.4 mmol, 2 equiv) were added. The mixture was stirred overnight. The mixture was diluted with dichloromethane and transferred to a round-bottom flask. NaOH (10%, aq) was added. The heterogeneous mixture was stirred vigorously for 15 mins. The aqueous and organic layers were separated. The aqueous layer was extracted twice with dichloromethane. The combined organic layer was dried over Na_2SO_4 and filtered. Solvent was removed *in vacuo*. The mixture was purified by column chromatography (5%–70% Et_2O in Pet. Ether) to afford *N*-benzyl-*N*-(4-methyl-1-phenylpentan-3-yl)cyclopropanamine (colourless oil, 19 mg, 30%) and *N*-benzyl-4-methyl-1-phenylpentan-3-amine (colourless oil, 9.2 mg, 17%).

N-benzyl-N-(4-methyl-1-phenylpentan-3-yl)cyclopropanamine

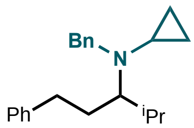

^{1}H NMR (400 MHz, $CDCl_3$) δ 7.35 – 7.30 (m, 6H), 7.28 – 7.17 (m, 4H), 3.88 (s, 2H), 2.93 – 2.83 (m, 1H), 2.62 – 2.46 (m, 2H), 2.25 – 2.16 (m, 1H), 2.11 – 1.91 (m, 2H), 1.81-1.69 (m, 1H), 0.99 – 1.06 (m, 6H), 0.41 – 0.23 (m, 4H).

^{13}C NMR (101 MHz, $CDCl_3$) δ 143.4, 141.6, 129.4, 128.5, 128.4, 127.9, 126.6, 125.7, 67.4, 56.5, 34.99, 34.95, 30.9, 30.0, 22.3, 20.5, 7.8, 7.7.

IR (film, cm^{-1}): 3025, 2927, 2866, 1494, 1452, 1028, 748, 696.

HRMS: m/z calcd for $C_{20}H_{30}N$ $[M+H]^+$ 308.2373; found 308.2373.

10.5 Tether alkene aminyl radical cation traps

10.5.1 Aminyl radical cation experiments

The use of tethered alkenes to undergo a 5-exo radical cyclisation to trap aminyl radical cations has been used to synthesize cyclic amines and as a mechanism probe in the literature.[26,27]

Based on this literature we attempted to use 4-pentenal and pent-4-en-1-amine hydrochloride as independent traps under our standard conditions to investigate this cyclisation.

10.5.2 Initial cyclisation attempts with isopropyl iodide

Initial investigations onto the feasibility of this trap were conducted below with variations using different aldehydes, radical traps and HAT reagents. Unfortunately, none of these improved the reaction profiles in a useful manner and would have clouded the results. Instead, in section 10.5.3, we have provided the results of the standard trap experiments for all structural classes.

2° Aldehyde tethered alkene

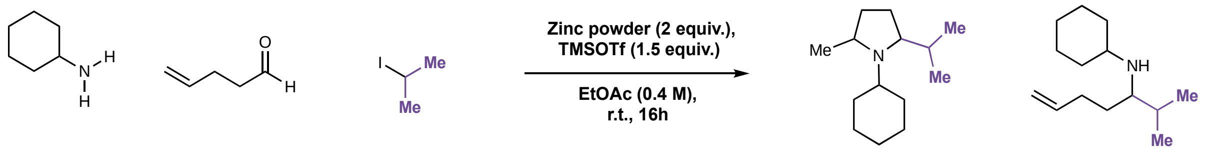

An oven dried vial (Biotage Microwave Process Vial (10 mL), equipped with gas-tight pierceable caps) was charged with a stirring bar and Zn dust (52 mg, 0.8 mmol, 2 equiv) and TEMPO (188 mg, 1.2 mmol, 3 equiv). The vial was sealed, evacuated and backfilled with N_2. To this was added EtOAc (1 mL), followed by the cyclohexylamine (46 µL, 0.4 mmol, 1.0 equiv), 4-pentenal (79 µL, 0.8 mmol, 2 equiv), and 2-iodopropane (120 µL, 1.2 mmol, 3 equiv). The reaction mixture was stirred for one minute, followed by the addition of TMSOTf (106 µL, 0.6 mmol, 1.5 equiv). The reaction mixture was stirred overnight. The crude mixture was transferred into a 50 ml round bottom flask and diluted with dichloromethane (approximately 20 ml). NaOH (approximately 15 ml, 10% aq.) was added. The heterogenous mixture was vigorously stirred for 10-15 min. The aqueous layer was extracted with dichloromethane three times. The combined organic layer was dried over anhydrous $MgSO_4$. The solvent was removed *in vacuo* and the reaction was analysed by ^{1}H NMR using 1,1,2,2-tetrachloroethane as an internal standard and LCMS analysis. ^{1}H NMR analysis showed a diverse mixture was present. LCMS analysis showed a wide variety of products. An extracted ion spectrum showed the presence of 2 major peaks corresponding to the expected products. Additionally, another extracted ion spectrum showed trace peaks indicating traces of the addition of the cyclised alkene radical intermediate to another iminium.

The ^{1}H NMR of the resulting reaction mixture using 1,1,2,2-tetrachloroethane as an internal standard.

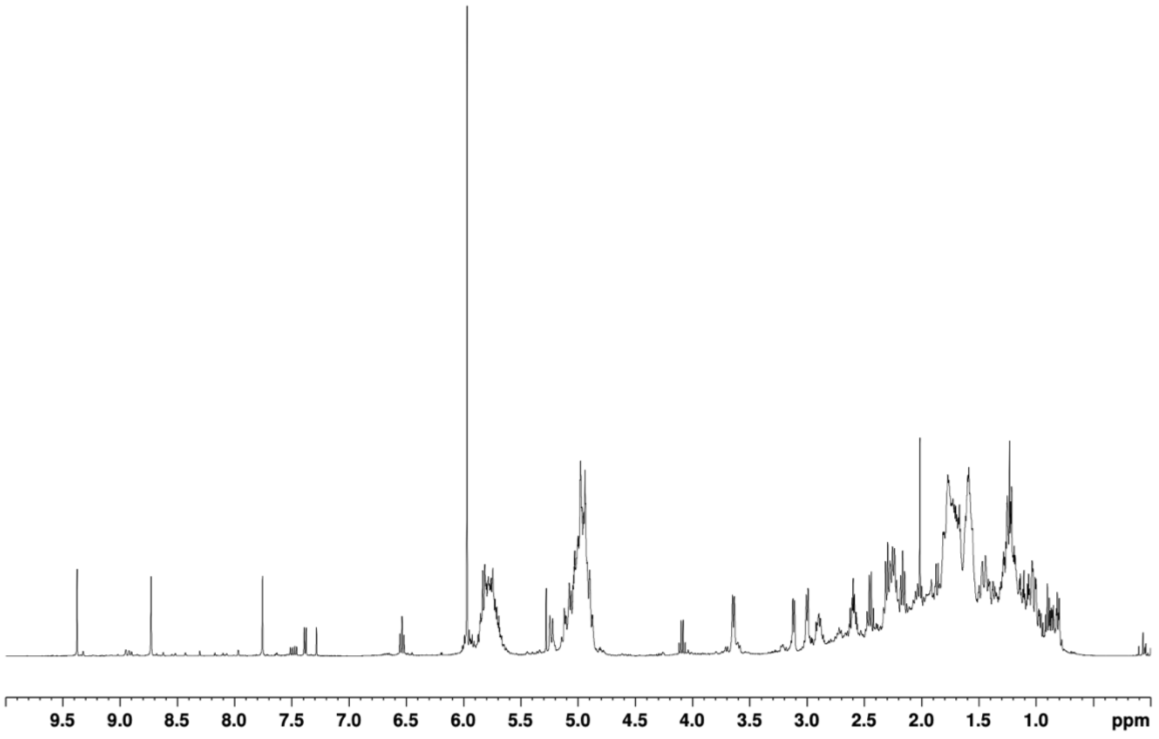

The LCMS TIC (+) and TIC (-) trace of the overall reaction mixture with extracted ion spectrums below

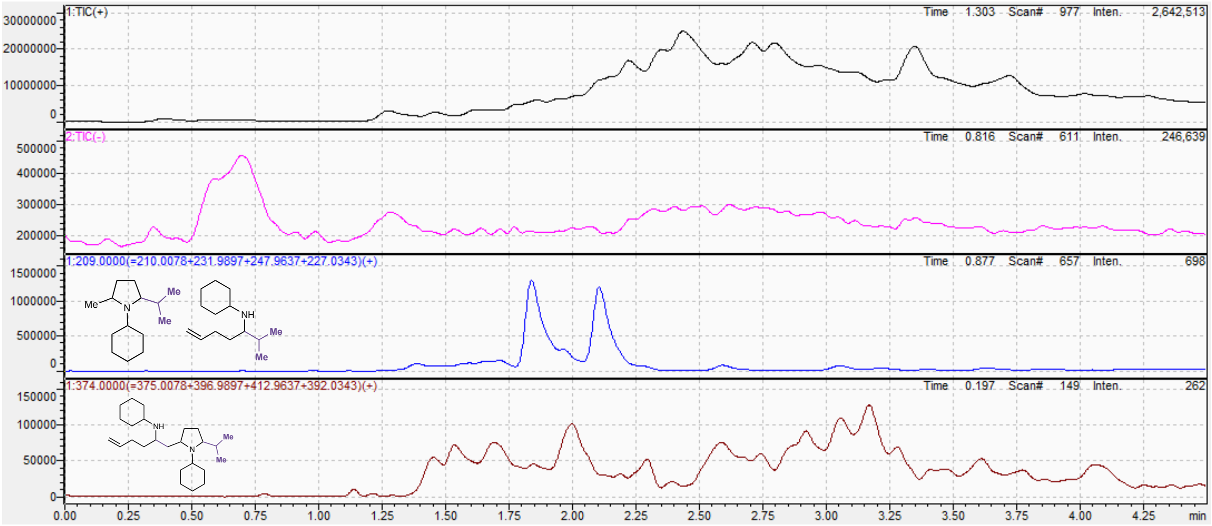

Further to these experiments several variations were attempted, using the same procedure as above, shown in the table below. Corresponding LCMS traces with extracted ion spectrums of the expected products and radical trap adducts are shown.

Experiment	Aldehyde	HAT or Radical Trap added
A	4-Pentenal	Et_3SiH
B	4-Pentenal	Ph_3SiH
C	4-Pentenal	TolS-STol
D	cis-4-Heptenal	None
E	cis-4-Heptenal	PhS-SPh
F	cis-4-Heptenal	I_2
G	cis-4-Heptenal	^{t}BuSH

Experiment A – LCMS TIC and extracted ion spectrums

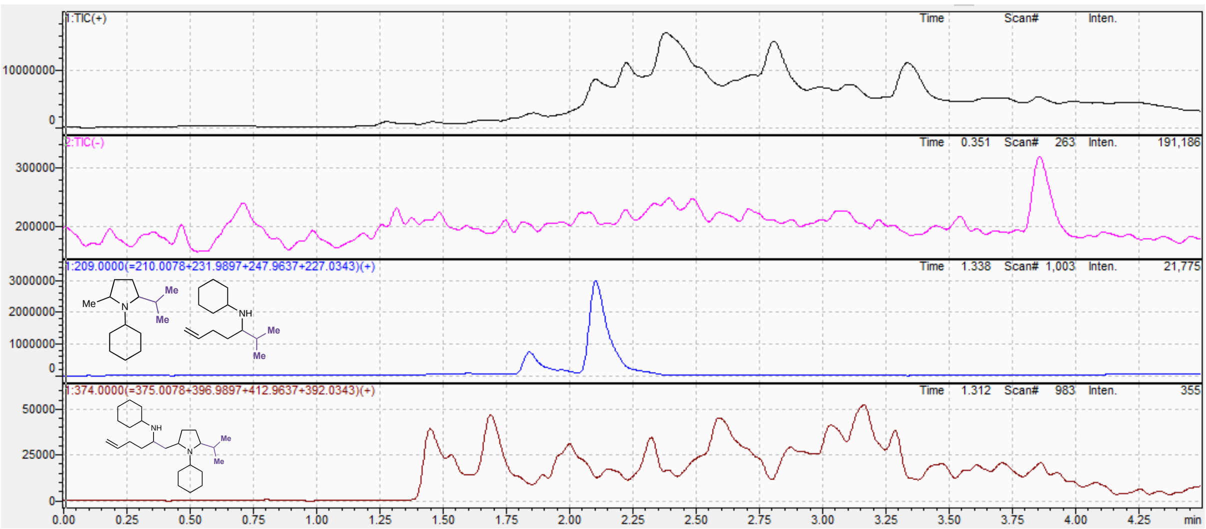

Experiment B – LCMS TIC and extracted ion spectrums

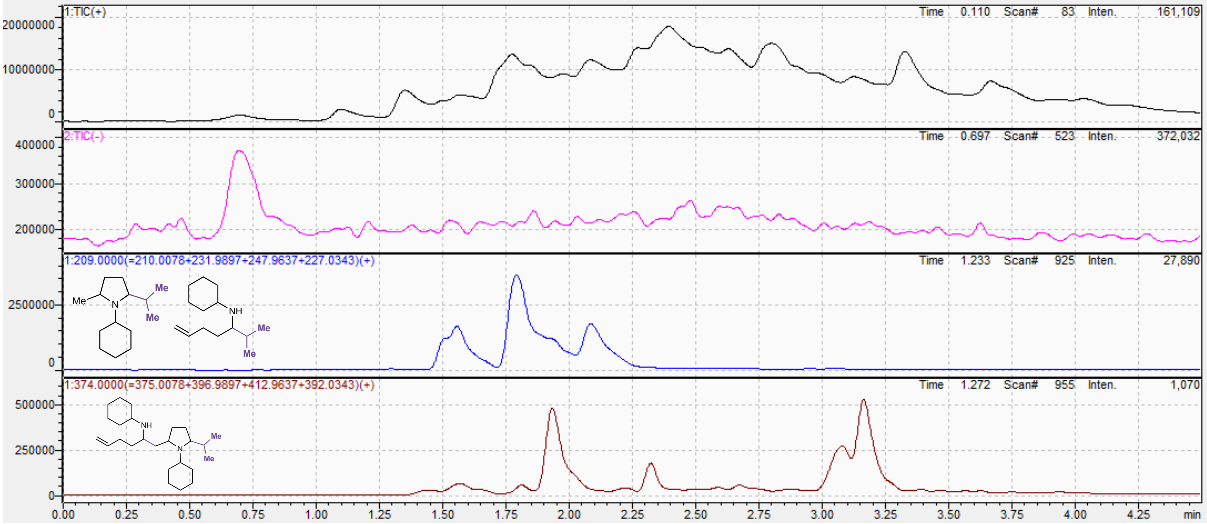

Experiment C – LCMS TIC and extracted ion spectrums

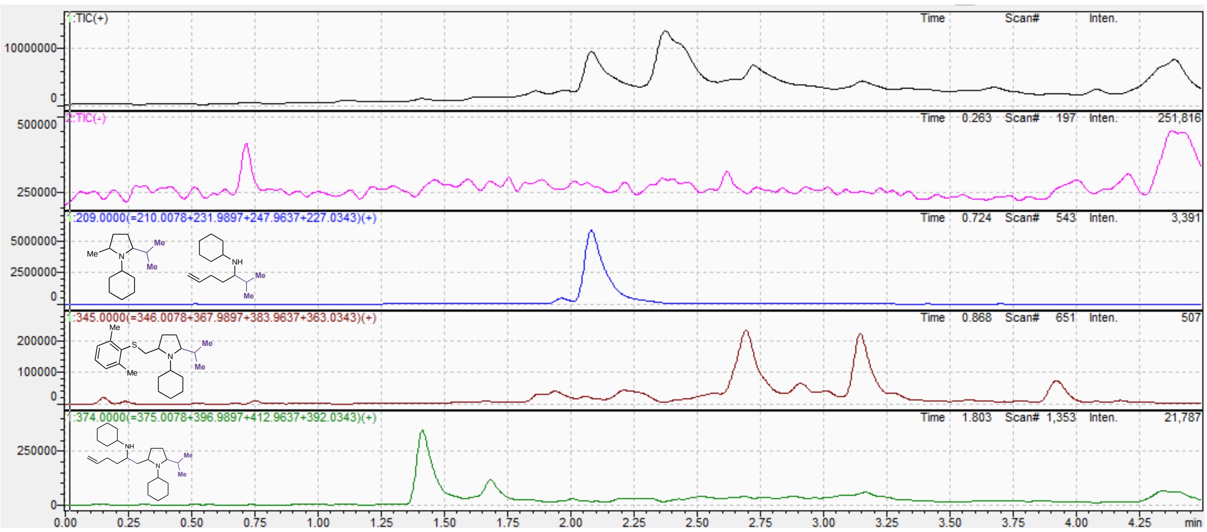

Experiment D – LCMS TIC and extracted ion spectrums

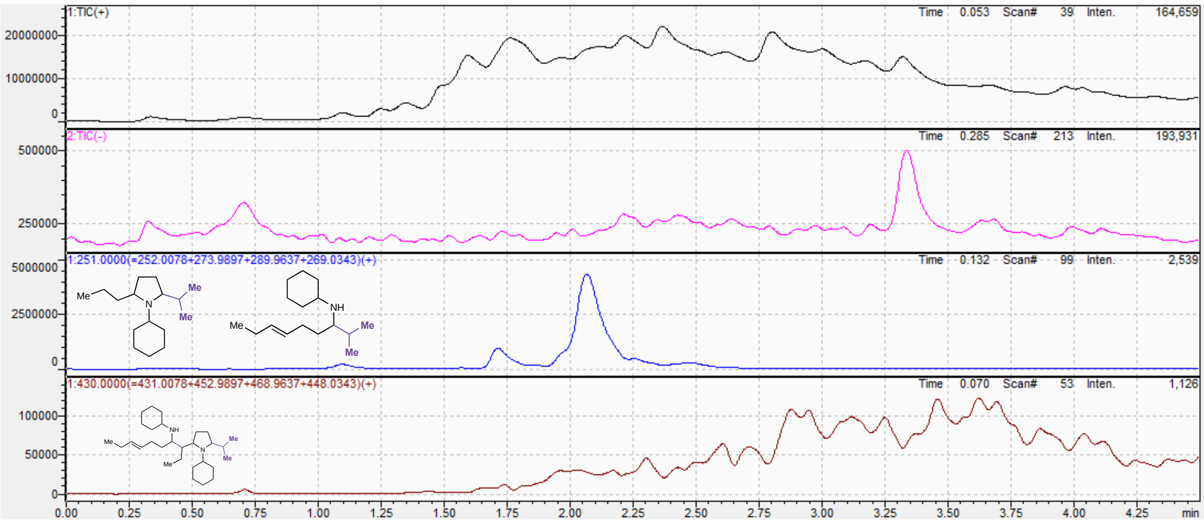

Experiment E – LCMS TIC and extracted ion spectrums

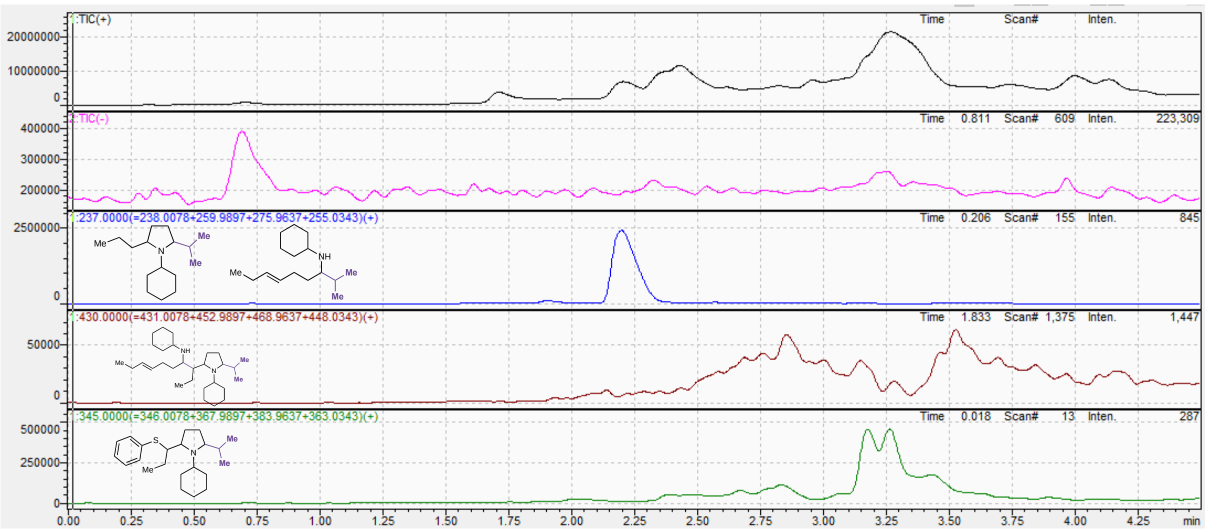

Experiment F – LCMS TIC and extracted ion spectrums

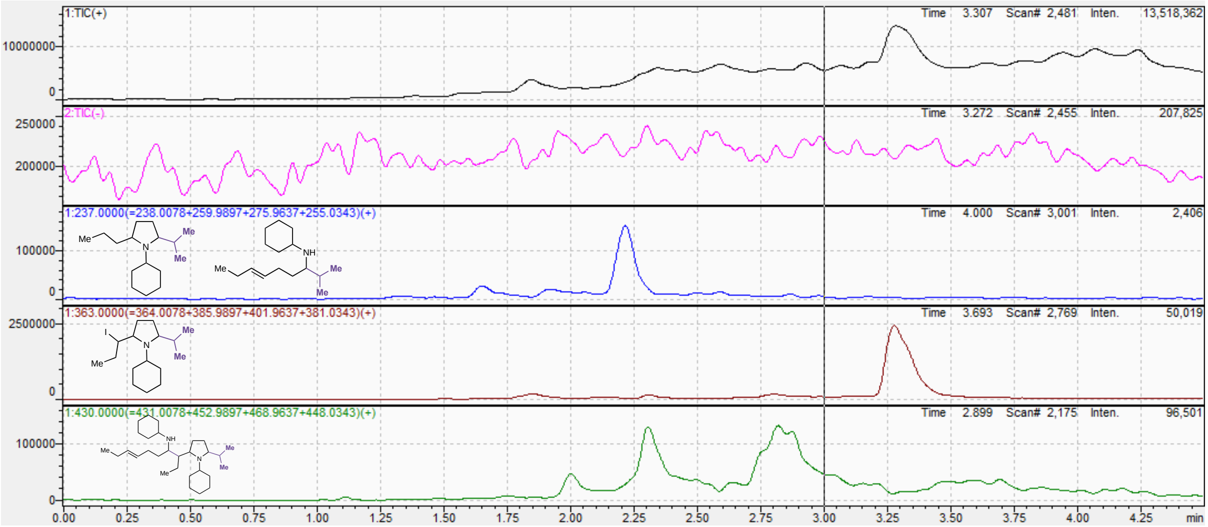

Experiment G – LCMS TIC and extracted ion spectrums

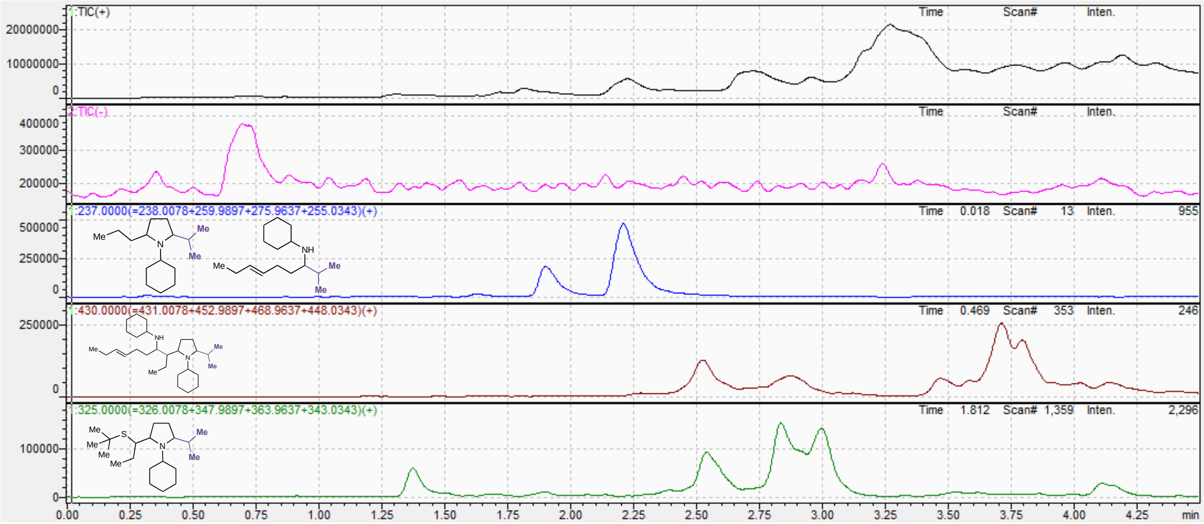

Section 10.5.3 Standardised amine and aldehyde tethered alkene trapping experiments

2° Amine tethered alkene

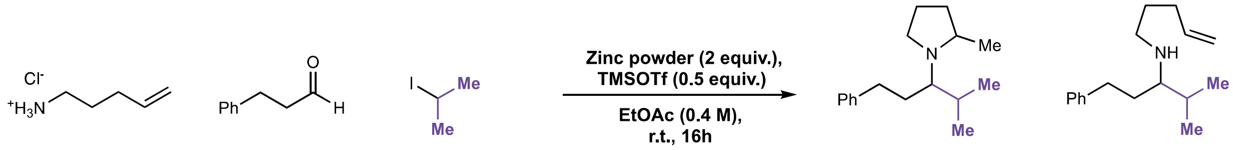

An oven dried vial (Biotage Microwave Process Vial (10 mL), equipped with gas-tight pierceable caps) was charged with a stirring bar, Zn (52 mg, 0.8 mmol, 2 equiv) and pent-4-en-1-amine hydrochloride (49 mg, 0.4 mmol, 1 equiv). The vial was sealed, evacuated and backfilled with N_2. To this was added EtOAc (1 mL), followed by hydrocinnamaldehyde (108 µL, 0.8 mmol, 2 equiv) and 2-iodopropane (120 µL, 1.2 mmol, 3 equiv). The reaction mixture was stirred for one minute, followed by the addition of TMSOTf (36 µL, 0.2 mmol, 0.5 equiv). The reaction mixture was stirred overnight. The crude mixture was transferred into a 50 ml round bottom flask and diluted with dichloromethane (approximately 20 ml). NaOH (approximately 15 ml, 10% aq.) was added. The heterogenous mixture was vigorously stirred for 10-15 min. The aqueous layer was extracted with dichloromethane three times. The combined organic layer was dried over anhydrous $MgSO_4$. The solvent was removed *in vacuo* and the reaction was analysed by ^{1}H NMR using 1,1,2,2-tetrachloroethane as an internal standard and LCMS analysis. ^{1}H NMR analysis showed a diverse mixture was present. LCMS analysis showed a wide variety of products. An extracted ion spectrum showed the presence of 2 major peaks corresponding to the expected products. Additionally, another extracted ion spectrum showed trace peaks showing the presence of the addition of the cyclised alkene radical intermediate to another iminium.

The ^{1}H NMR of the resulting reaction mixture using 1,1,2,2-tetrachloroethane as an internal standard.

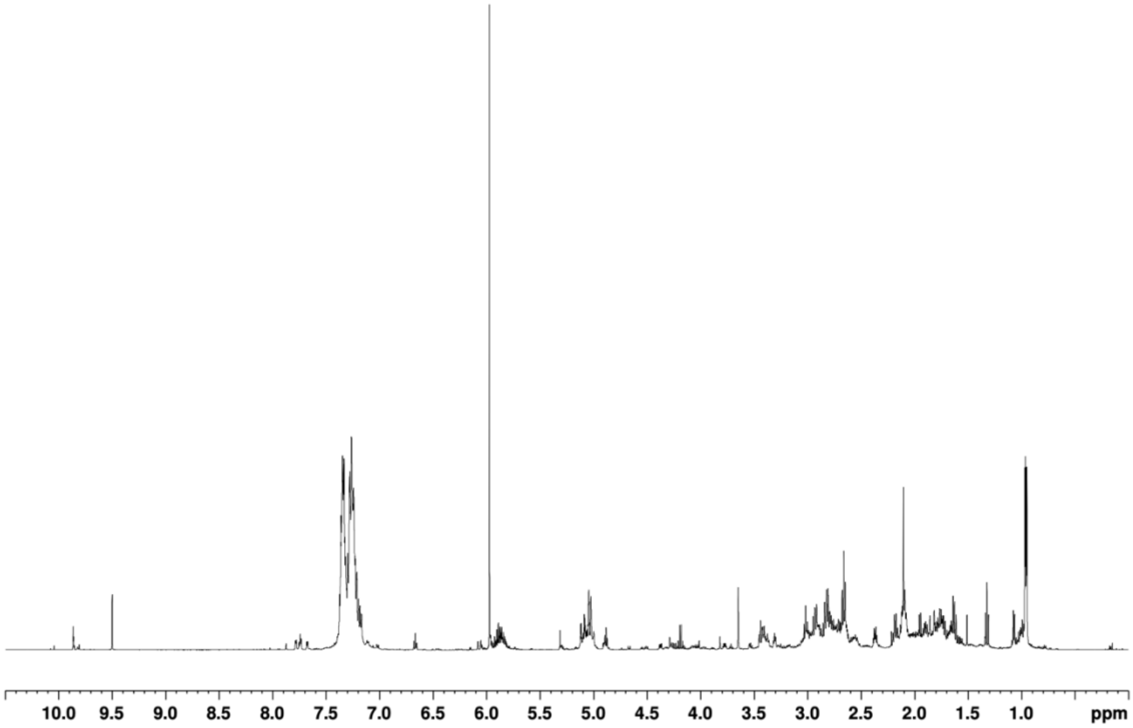

The LCMS TIC (+) and TIC (-) trace of the overall reaction mixture with extracted ion spectrums below

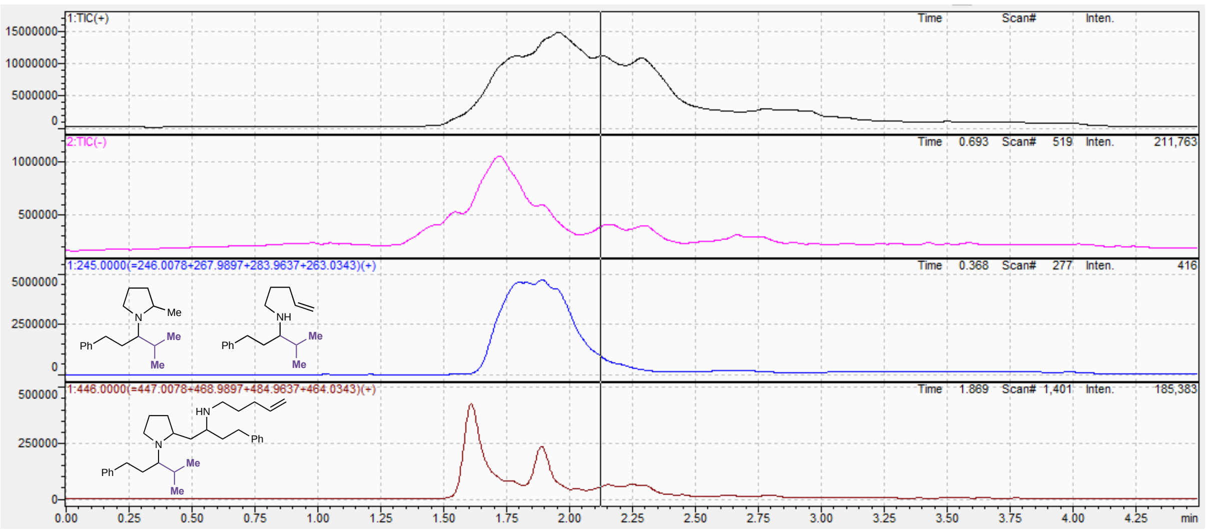

1° Amine tethered alkene

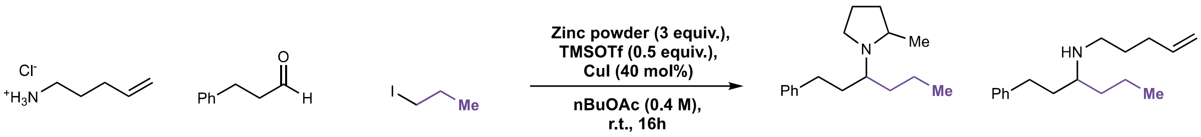

An oven dried vial (Biotage Microwave Process Vial (10 mL), equipped with gas-tight pierceable caps) was charged with a stirring bar, Zn dust (78 mg, 1.2 mmol, 3 equiv), copper iodide (30 mg, 0.16 mmol, 40 mol%) and pent-4-en-1-amine hydrochloride (49 mg, 0.4 mmol, 1 equiv). The vial was sealed, evacuated and backfilled with N_2. To this was added *n*BuOAc (1 mL), followed by and hydrocinnamaldehyde (108 µL, 0.8 mmol, 2 equiv). The mixture was stirred for 15 minutes then, whilst stirring, 1-iodopropane (156 µL, 1.6 mmol, 4 equiv), and TMSOTf (106 µL, 0.6 mmol, 1.5 equiv) were added. The reaction mixture was stirred overnight. The crude mixture was transferred into a 50 ml round bottom flask and diluted with dichloromethane (approximately 20 mL). NaOH (approximately 15 ml, 10% aq.) was added. The heterogenous mixture was vigorously stirred for 10-15 min. The aqueous layer was extracted with dichloromethane three times. The combined organic layer was dried over anhydrous $MgSO_4$. The solvent was removed *in vacuo* and the reaction was analysed by ^{1}H NMR using 1,1,2,2-tetrachloroethane as an internal standard and LCMS analysis. ^{1}H NMR analysis showed a diverse mixture was present. LCMS analysis showed a wide variety of products. An extracted ion spectrum showed the presence of 2 major peaks corresponding to the expected products. Additionally, another extracted ion spectrum showed trace peaks showing the presence of the addition of the cyclised alkene radical intermediate to another iminium.

The ^{1}H NMR of the resulting reaction mixture using 1,1,2,2-tetrachloroethane as an internal standard.

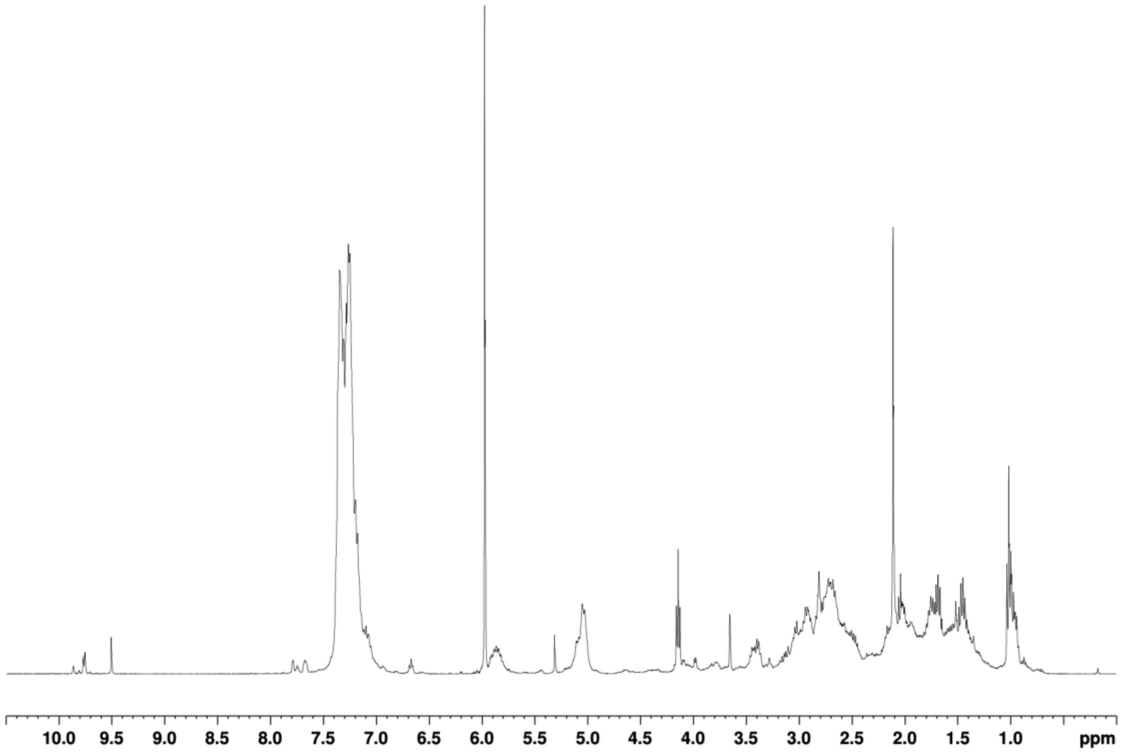

The LCMS TIC (+) and TIC (-) trace of the overall reaction mixture with extracted ion spectrums below

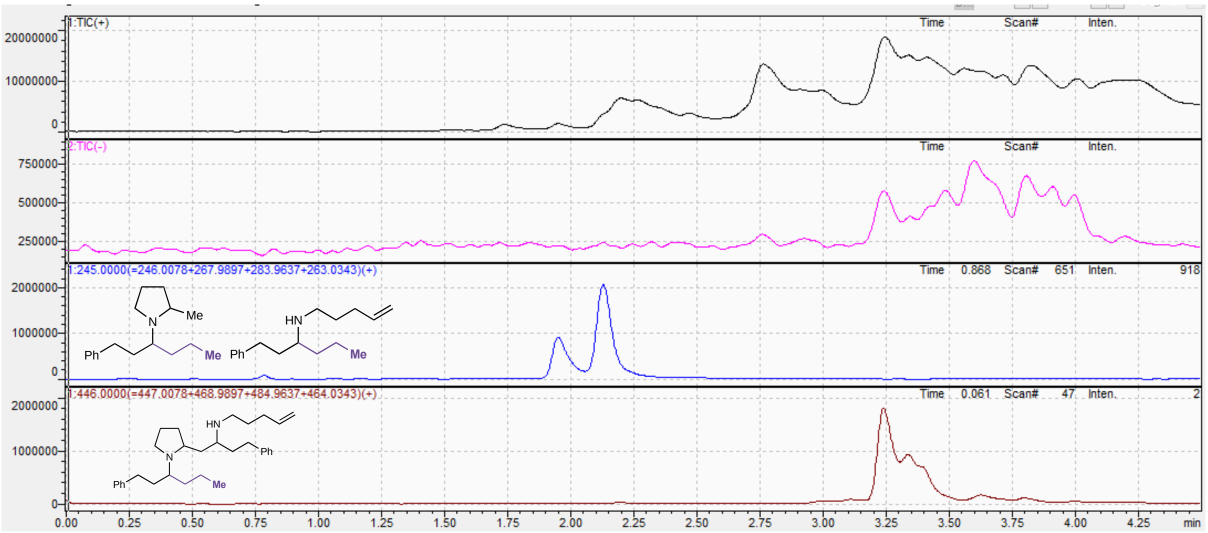

1° Aldehyde tethered alkene

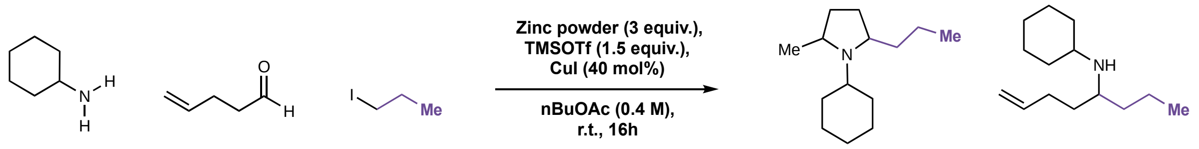

An oven dried vial (Biotage Microwave Process Vial (10 mL), equipped with gas-tight pierceable caps) was charged with a stirring bar, Zn (78 mg, 1.2 mmol, 3 equiv) and copper iodide (30 mg, 0.16 mmol, 40 mol%). The vial was sealed, evacuated and backfilled with N_2. To this was added *n*BuOAc (1 mL), followed by the cyclohexylamine (46 µL, 0.4 mmol, 1.0 equiv) and 4-pentenal (79 µL, 0.8 mmol, 2 equiv). The mixture was stirred for 15 minutes then, whilst stirring, 1-iodopropane (156 µL, 1.6 mmol, 4 equiv), and TMSOTf (106 µL, 0.6 mmol, 1.5 equiv) were added. The reaction mixture was stirred overnight. The crude mixture was transferred into a 50 ml round bottom flask and diluted with dichloromethane (approximately 20 ml). NaOH (approximately 15 ml, 10% aq.) was added. The heterogenous mixture was vigorously stirred for 10-15 min. The aqueous layer was extracted with dichloromethane three times. The combined organic layer was dried over anhydrous $MgSO_4$. The solvent was removed *in vacuo* and the reaction was analysed by ^{1}H NMR using 1,1,2,2-tetrachloroethane as an internal standard and LCMS analysis. ^{1}H NMR analysis showed a diverse mixture was present. LCMS analysis showed a wide variety of products. An extracted ion spectrum showed the presence of 2 major peaks corresponding to the expected products. Additionally, another extracted ion spectrum showed trace peaks showing the presence of the addition of the cyclised alkene radical intermediate to another iminium.

The ^{1}H NMR of the resulting reaction mixture using 1,1,2,2-tetrachloroethane as an internal standard

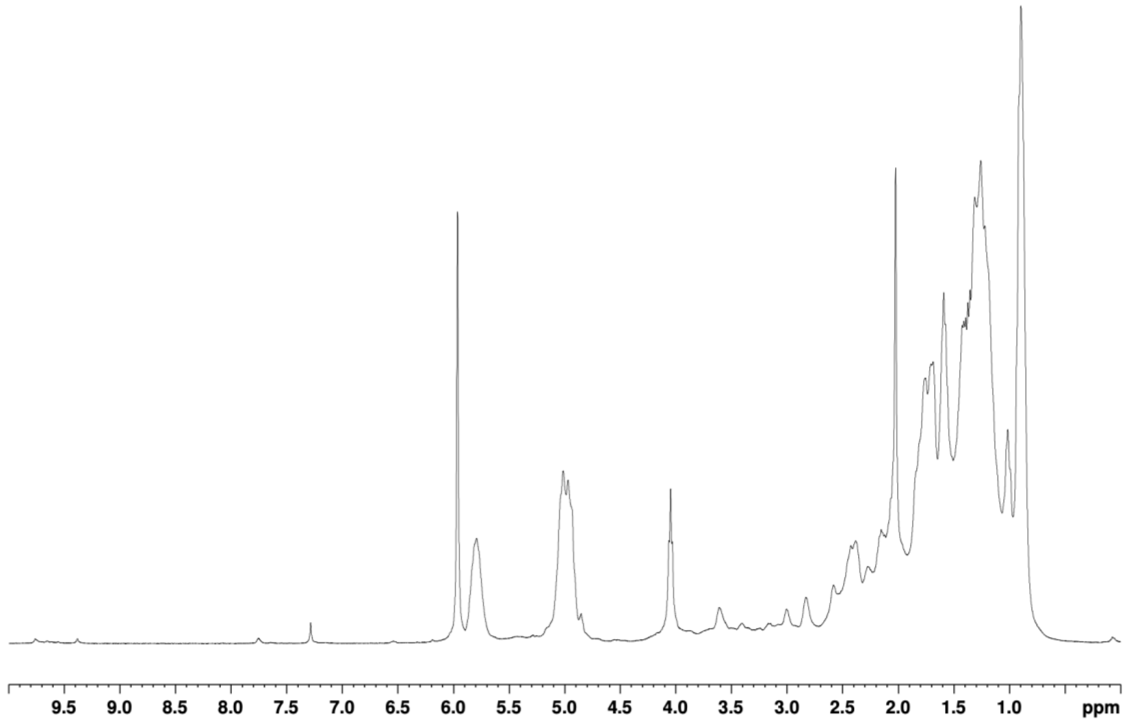

The LCMS TIC (+) and TIC (-) trace of the overall reaction mixture with extracted ion spectrums below

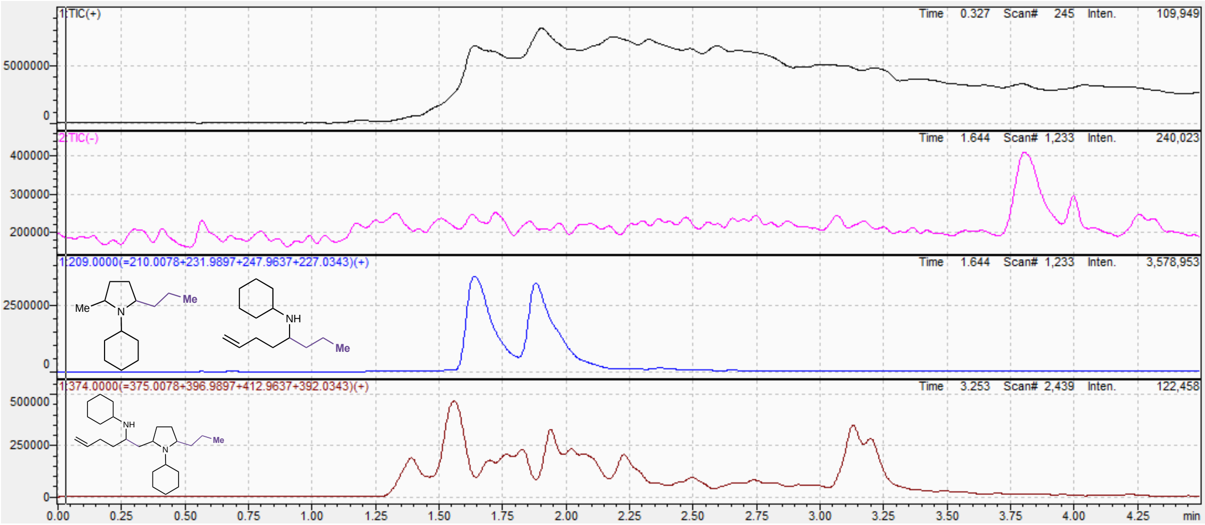

3° Amine tethered alkene

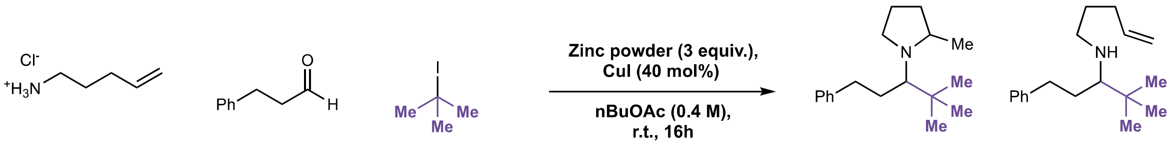

An oven dried vial (Biotage Microwave Process Vial (10 mL), equipped with gas-tight pierceable caps) was charged with a stirring bar, Zn dust (78 mg, 1.2 mmol, 3 equiv), copper iodide (30 mg, 0.16 mmol, 40 mol%) and pent-4-en-1-amine hydrochloride (49 mg, 0.4 mmol, 1 equiv). The vial was sealed, evacuated and backfilled with N_2. To this was added *n*BuOAc (1 mL), followed by hydrocinnamaldehyde (108 µL, 0.8 mmol, 2 equiv). The mixture was stirred for 15 minutes then, whilst stirring, tert-butyl iodide (143 µL, 1.2 mmol, 3 equiv) was added. The reaction mixture was stirred overnight. The crude mixture was transferred into a 50 ml round bottom flask and diluted with dichloromethane (approximately 20 ml). NaOH (approximately 15 ml, 10% aq.) was added. The heterogenous mixture was vigorously stirred for 10-15 min. The aqueous layer was extracted with dichloromethane three times. The combined organic layer was dried over anhydrous $MgSO_4$. The solvent was removed *in vacuo* and the reaction was analysed by ^{1}H NMR using 1,1,2,2-tetrachloroethane as an internal standard and LCMS analysis. LCMS analysis showed a wide variety of products. An extracted ion spectrum showed the presence of 1 major peak corresponding to the expected products. Additionally, another extracted ion spectrum showed trace peaks showing the presence of the addition of the cyclised alkene radical intermediate to another iminium.

The ¹H NMR of the resulting reaction mixture using 1,1,2,2-tetrachloroethane as an internal standard

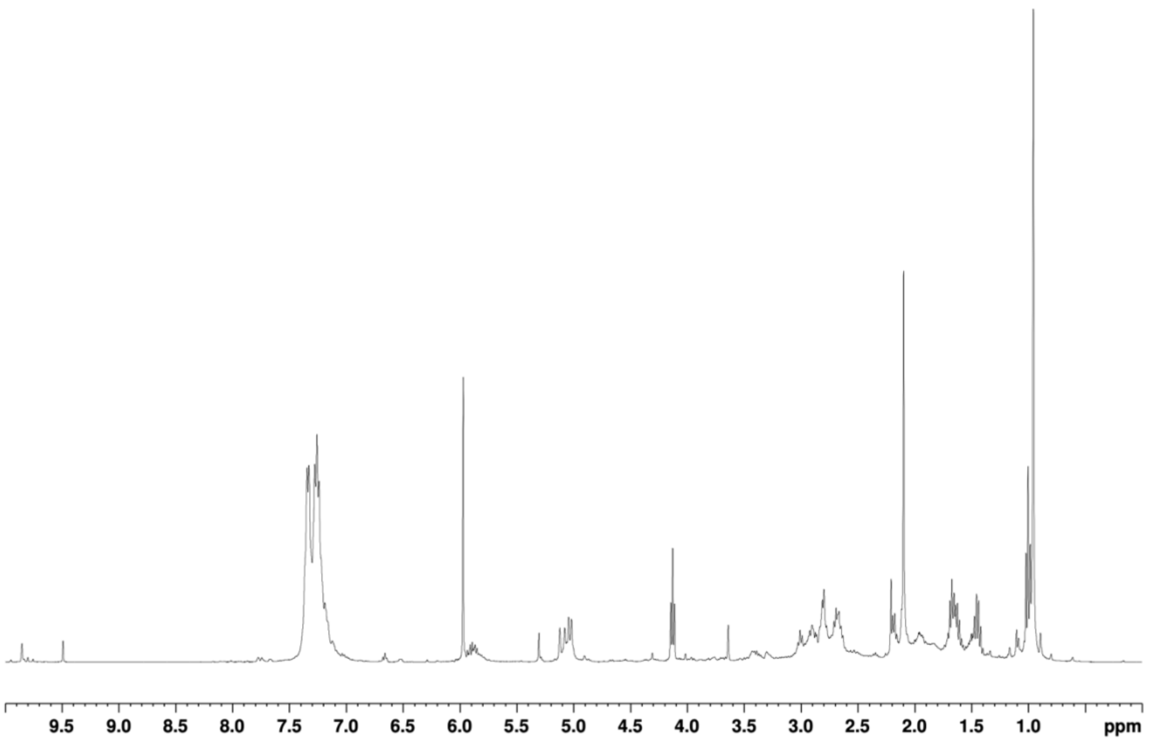

The LCMS TIC (+) and TIC (-) trace of the overall reaction mixture with extracted ion spectrums below

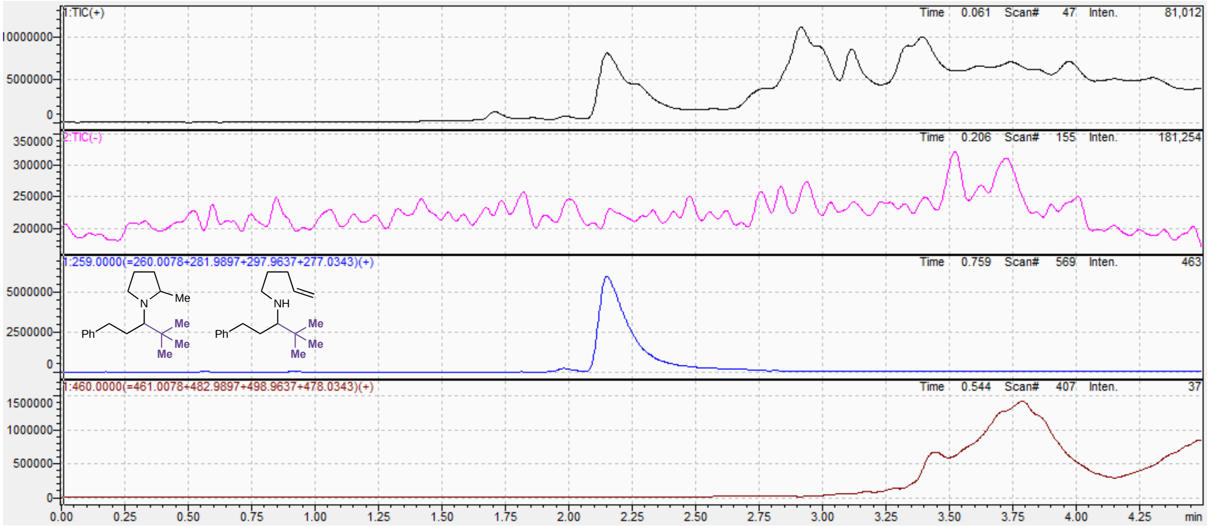

3° Aldehyde tethered alkene

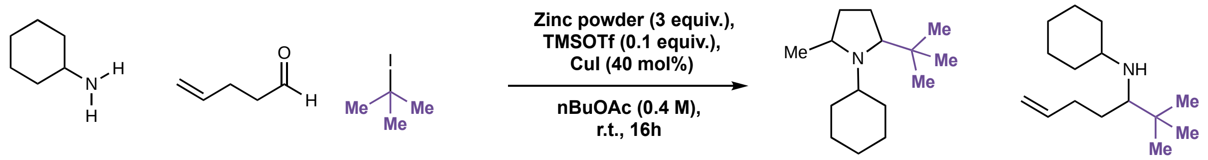

An oven dried vial (Biotage Microwave Process Vial (10 mL), equipped with gas-tight pierceable caps) was charged with a stirring bar, Zn dust (78 mg, 1.2 mmol, 3 equiv) and copper iodide (30 mg, 0.16 mmol, 40 mol%). The vial was sealed, evacuated and backfilled with N_2. To this was added *n*BuOAc (1 mL), followed by cyclohexylamine (46 µL, 0.4 mmol, 1.0 equiv) and 4-pentenal (79 µL, 0.8 mmol, 2 equiv). The mixture was stirred for 15 minutes then, whilst stirring, tert-butyl iodide (143 µL, 1.2 mmol, 3 equiv) was added. The reaction mixture was stirred overnight. The crude mixture was transferred into a 50 ml round bottom flask and diluted with dichloromethane (approximately 20 ml). NaOH (approximately 15 ml, 10% aq.) was added. The heterogenous mixture was vigorously stirred for 10-15 min. The aqueous layer was extracted with dichloromethane three times. The solvent was removed *in vacuo* and the reaction was analysed by ^{1}H NMR using 1,1,2,2-tetrachloroethane as an internal standard and LCMS analysis. LCMS analysis showed a wide variety of products. An extracted ion spectrum showed the presence of 1 major peak corresponding to the expected products. Additionally, another extracted ion spectrum showed trace peaks showing the presence of the addition of the cyclised alkene radical intermediate to another iminium.

The ^{1}H NMR of the resulting reaction mixture using 1,1,2,2-tetrachloroethane as an internal standard

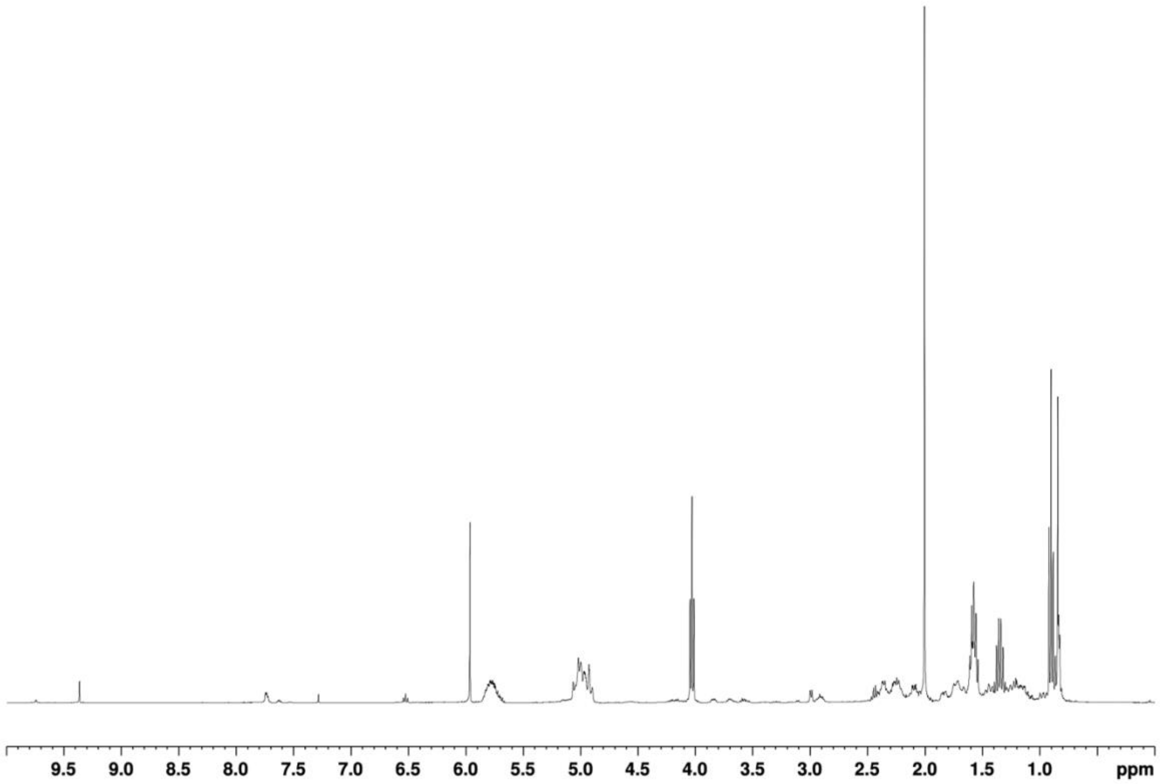

The LCMS TIC (+) and TIC (-) trace of the overall reaction mixture with extracted ion spectrums below

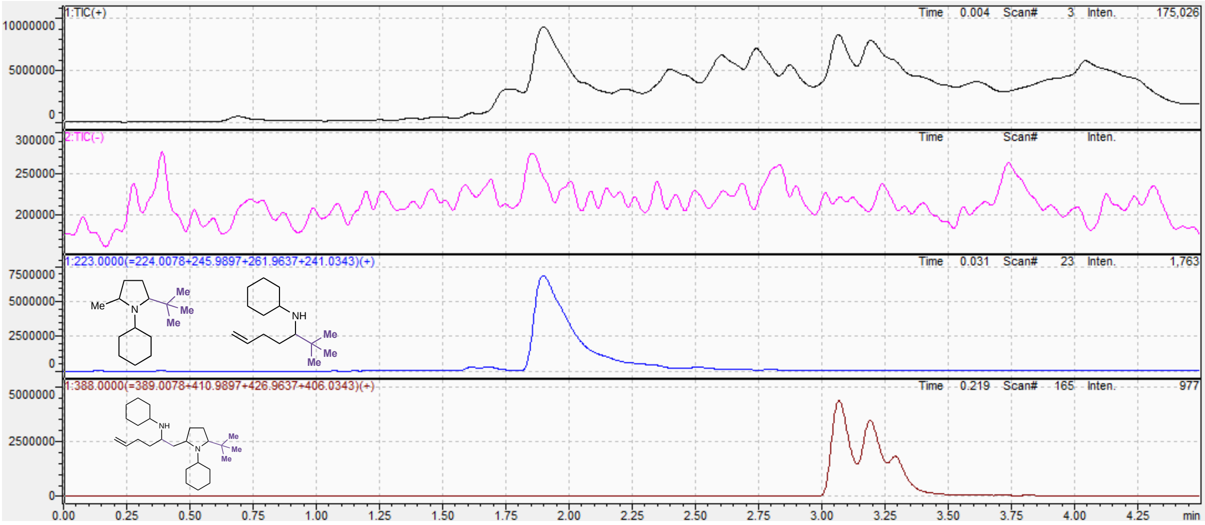

RAE Amine tethered alkene

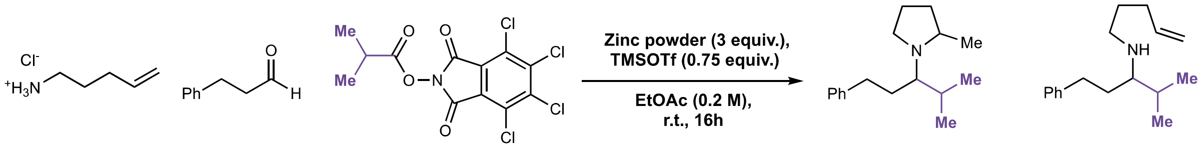

An oven dried vial (Biotage Microwave Process Vial 10 mL, equipped with gas-tight pierceable caps) was charged with a stirring bar and pent-4-en-1-amine hydrochloride (49 mg, 0.4 mmol, 1 equiv). The vial was sealed, evacuated and backfilled with N_2. To this was added EtOAc (1 mL), followed hydrocinnamaldehyde (54 µL, 0.4 mmol, 2 equiv) and TMSOTf (27 µL, 0.15 mmol, 0.75 equiv). The mixture was stirred for 15 mins. The cap was removed. Zinc (39 mg, 0.6 mmol, 3 equiv) and the 4,5,6,7-tetrachloro-1,3-dioxoisoindolin-2-yl isopropylcarboxylate (223 mg, 0.6 mmol, 3 equiv) were added in one portion. The microwave vial was sealed, put under vacuum and refilled with N_2. The mixture was stirred overnight. The crude mixture was transferred into a 50 ml round bottom flask and diluted with dichloromethane (approximately 20 ml). NaOH (approximately 15 ml, 10% aq.) was added. The heterogenous mixture was vigorously stirred for 10-15 min. The aqueous layer was extracted with dichloromethane three times. The combined organic layer was dried over anhydrous $MgSO_4$. The solvent was removed *in vacuo* and the reaction was analysed by ^1H NMR using 1,3,5-trimethoxybenzene as an internal standard and LCMS analysis. LCMS analysis showed a wide variety of products. An extracted ion spectrum showed the presence of 2 major overlapping peaks corresponding to the expected products. Additionally, another extracted ion spectrum showed trace peaks showing the presence of the addition of the cyclised alkene radical intermediate to another iminium.

The ^{1}H NMR of the resulting reaction mixture using 1,3,5-trimethoxybenzene as an internal standard

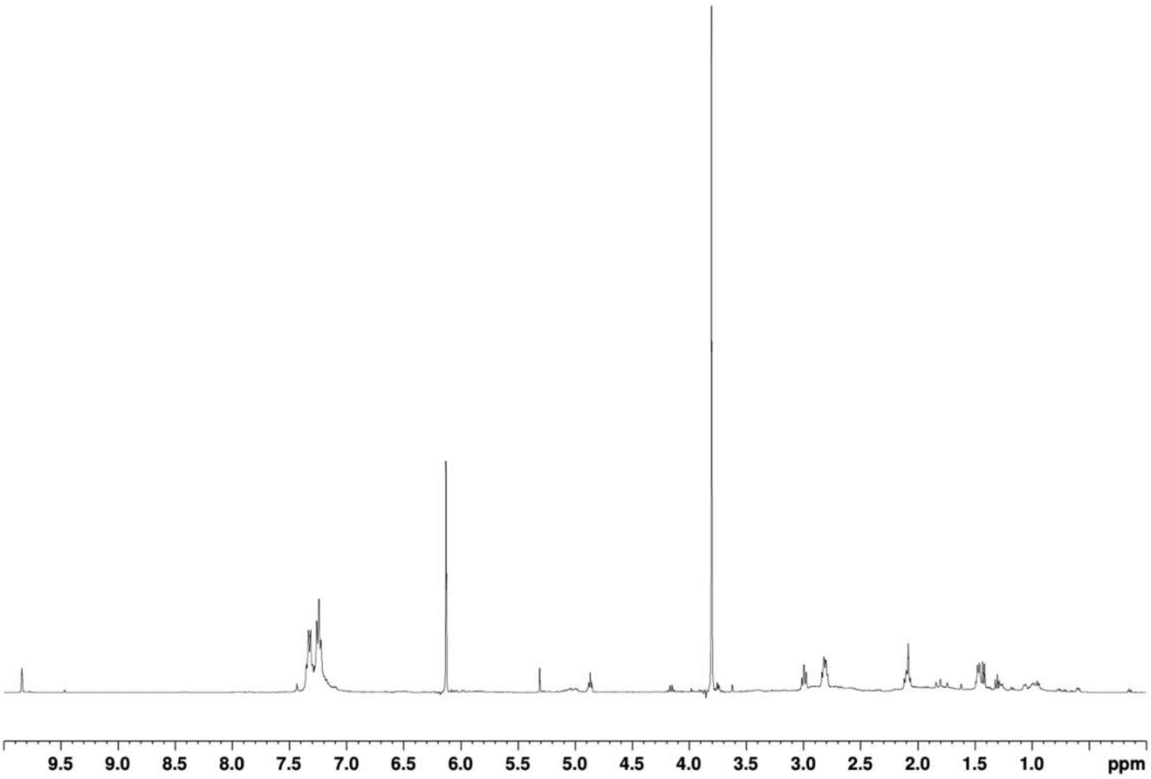

The LCMS TIC (+) and TIC (-) trace of the overall reaction mixture with extracted ion spectrums below

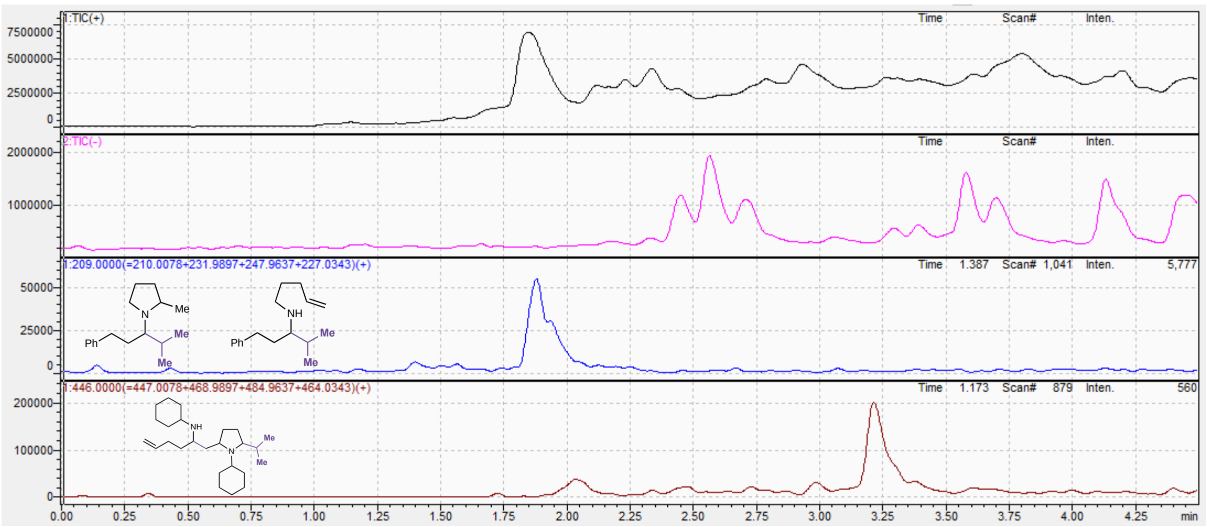

RAE Aldehyde tethered alkene

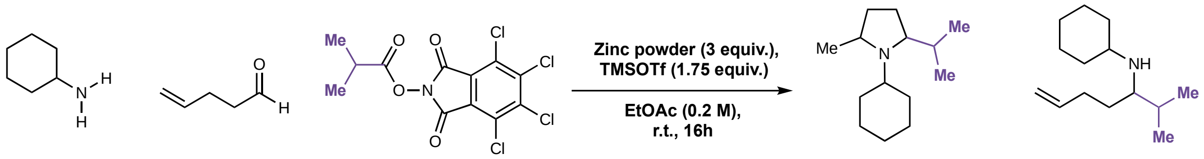

An oven dried vial (Biotage Microwave Process Vial 10 mL, equipped with gas-tight pierceable caps) was charged with a stirring bar. The vial was sealed, evacuated and backfilled with N_2. To this was added EtOAc (1 mL), followed by cyclohexylamine (23 µL, 0.2 mmol, 1.0 equiv) and 4-pentenal (40 µL, 0.4 mmol, 2 equiv) and TMSOTf (63 µL, 0.35 mmol, 1.75 equiv). The mixture was stirred for 15 mins. The cap was removed. Zinc (39 mg, 0.6 mmol, 3 equiv) and the 4,5,6,7-tetrachloro-1,3-dioxoisoindolin-2-yl isopropylcarboxylate (223 mg, 0.6 mmol, 3 equiv) were added in one portion. The microwave vial was sealed, put under vacuum and refilled with N_2. The mixture was stirred overnight. The crude mixture was transferred into a 50 ml round bottom flask and diluted with dichloromethane (approximately 20 ml). NaOH (approximately 15 ml, 10% aq.) was added. The heterogenous mixture was vigorously stirred for 10-15 min. The aqueous layer was extracted with dichloromethane three times. The combined organic layer was dried over anhydrous $MgSO_4$. The solvent was removed *in vacuo* and the reaction was analysed by ^1H NMR using 1,3,5-trimethoxybenzene as an internal standard and LCMS analysis. LCMS analysis showed a wide variety of products. An extracted ion spectrum showed the presence of 1 major peak and 1 minor corresponding to the expected products. Additionally, another extracted ion spectrum showed trace peaks indicating traces of the addition of the cyclised alkene radical intermediate to another iminium.

The ^1H NMR of the resulting reaction mixture using 1,3,5-trimethoxybenzene as an internal standard

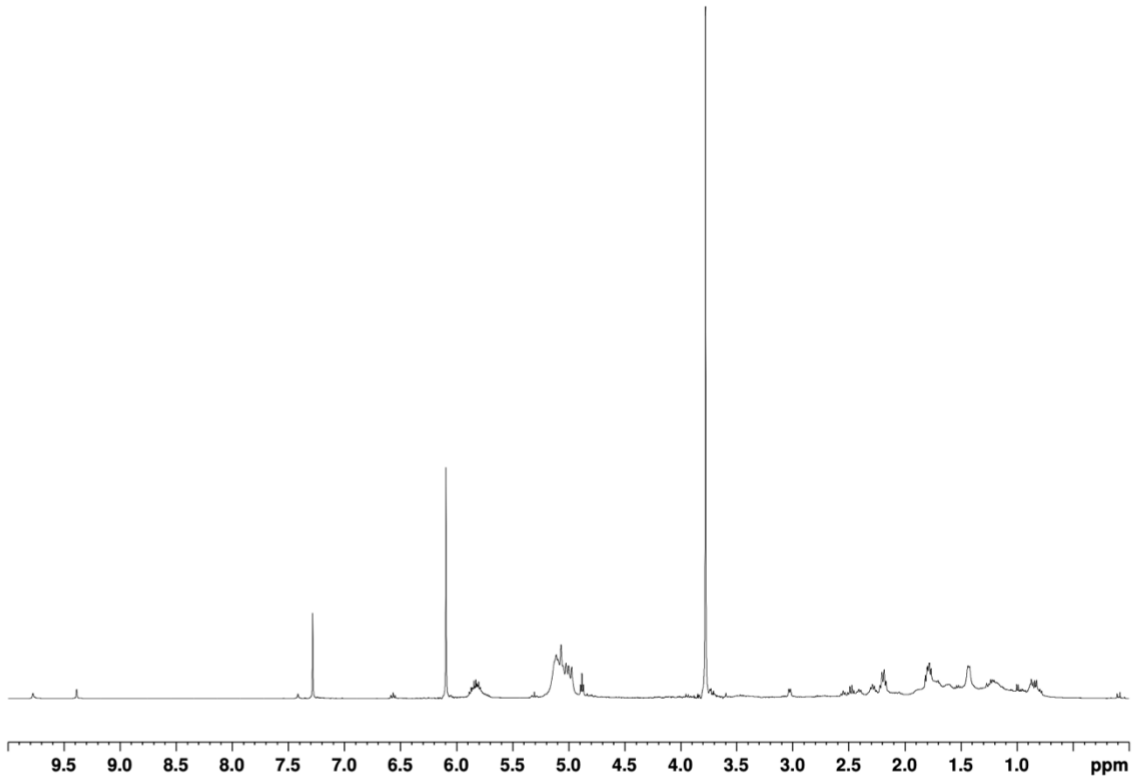

The LCMS TIC (+) and TIC (-) trace of the overall reaction mixture with extracted ion spectrums below

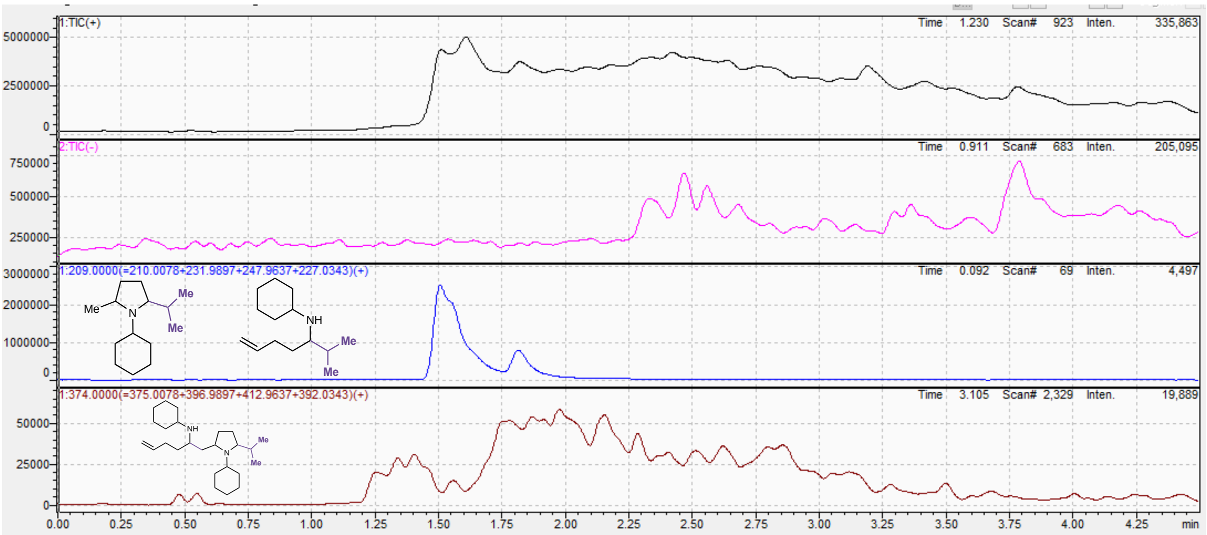

Ketone Amine tethered alkene

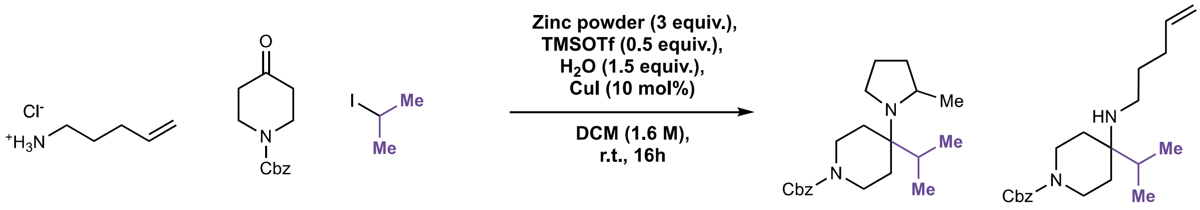

An oven dried vial (Biotage Microwave Process Vial 10 mL, equipped with gas-tight pierceable caps) was charged with a stirring bar, pent-4-en-1-amine hydrochloride (49 mg, 0.4 mmol, 1 equiv), benzyl 4-oxopiperidine-1-carboxylate (140 mg, 0.6 mmol, 1.5 equiv), Zn dust (79 mg, 1.2 mmol, 3 equiv) and copper iodide (7.5 mg, 0.04 mmol, 10 mol%). The vial was sealed, evacuated and backfilled with N_2. To this was added dichloromethane (0.25 mL). While stirring, 2-iodopropane (120 µL, 1.2 mmol, 3 equiv), H_2O (11 µL, 0.6 mmol, 1.5 equiv) and then TMSOTf (36 µL, 0.2 mmol, 0.5 equiv) were added. The mixture was stirred overnight. The mixture was diluted with dichloromethane and basified with NaOH (10%, aq). The heterogenous mixture was stirred vigorously for 15 mins. The organic and aqueous layers were separated. The aqueous layer was extracted with dichloromethane twice. The combined organic layer was dried over anhydrous $MgSO_4$. The solvent was removed *in vacuo* and the reaction was analysed by ^{1}H NMR using 1,1,2,2-tetrachloroethane as an internal standard and LCMS analysis. ^{1}H NMR analysis showed a diverse mixture was present. LCMS analysis showed a wide variety of products. An extracted ion spectrum showed the presence of 2 major peaks corresponding to the expected products. Additionally, another extracted ion spectrum showed trace peaks showing the presence of the addition of the cyclised alkene radical intermediate to another iminium.

The ^{1}H NMR of the resulting reaction mixture using 1,1,2,2-tetrachloroethane as an internal standard

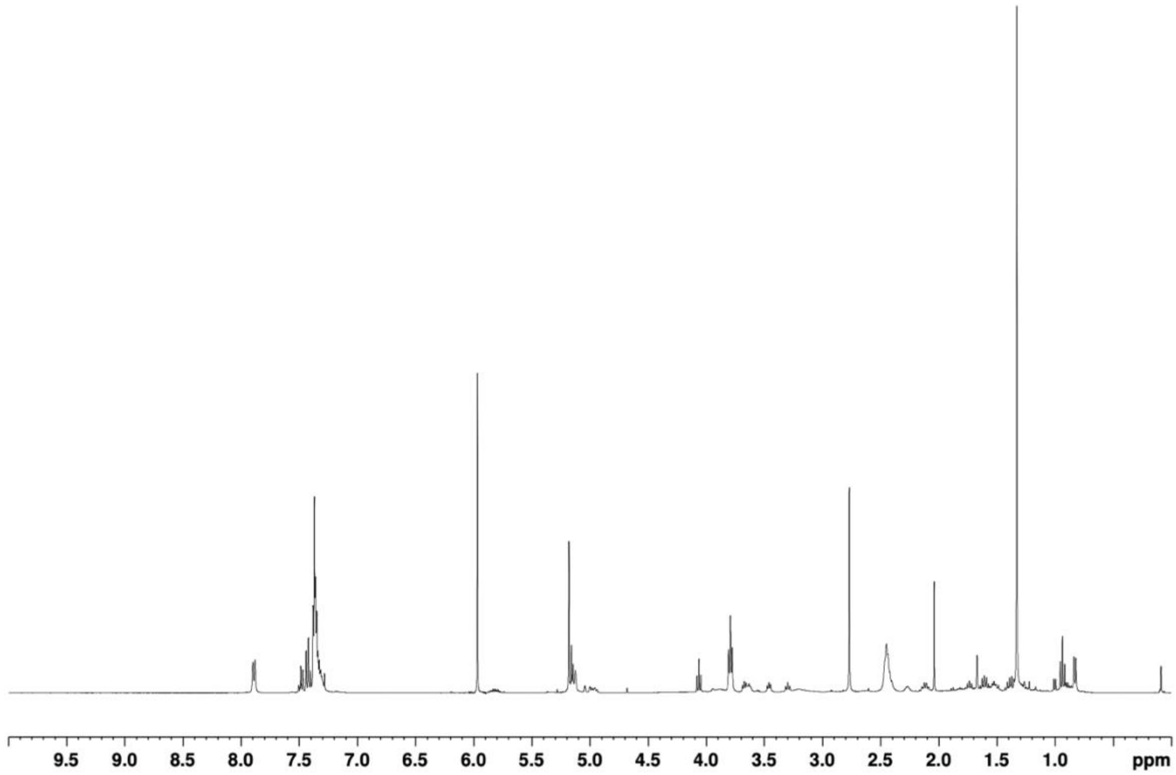

The LCMS TIC (+) and TIC (-) trace of the overall reaction mixture with extracted ion spectrums below

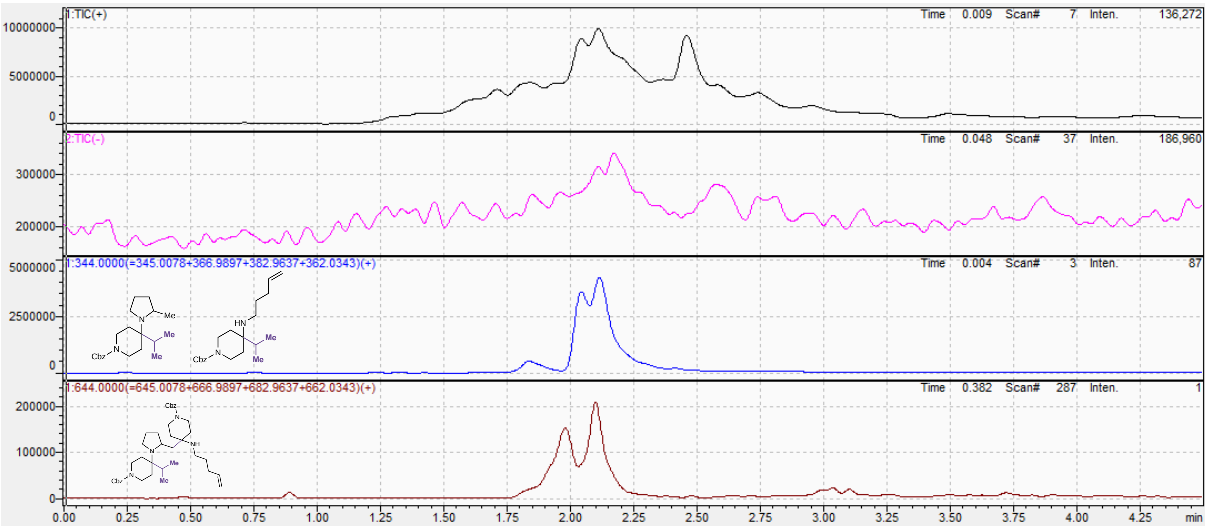

11. Additional Scope

Amines

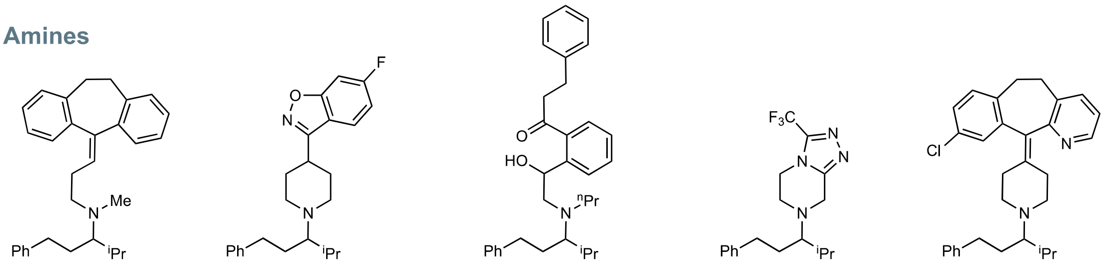

63% IY | 82% IY | 31% IY | 40% AY | 0%

Aldehydes

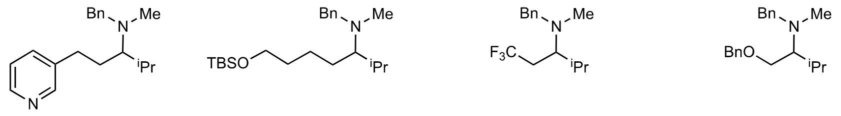

20% IY | 0% | 0% | 0%

Alkyl halides

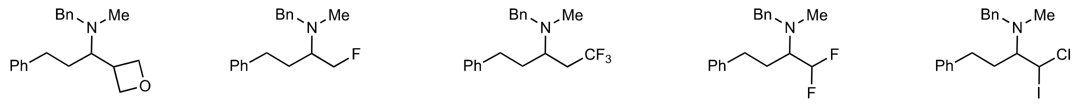

65% AY Decomposed during isolation | 20% AY | 0% | 0% | 0%

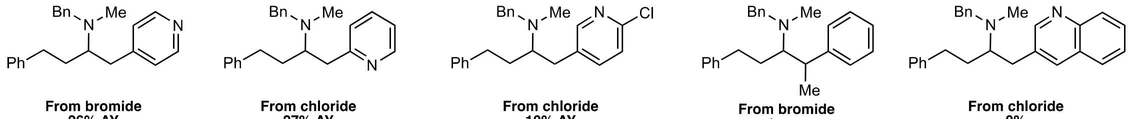

From bromide 26% AY | From chloride 27% AY | From chloride 10% AY | From bromide trace | From chloride 0%

Redox active esters

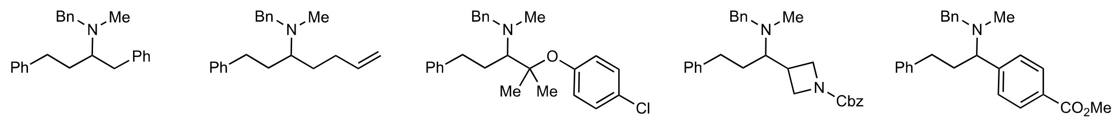

30% AY | 0% | 0% | 0% | 0%

CuI conditions

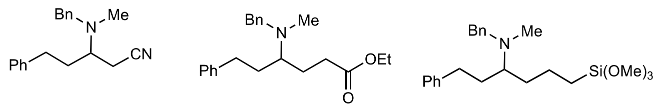

From bromide 80% AY 54% IY | 47% AY | 0% | 0%

α-Tertiary amines

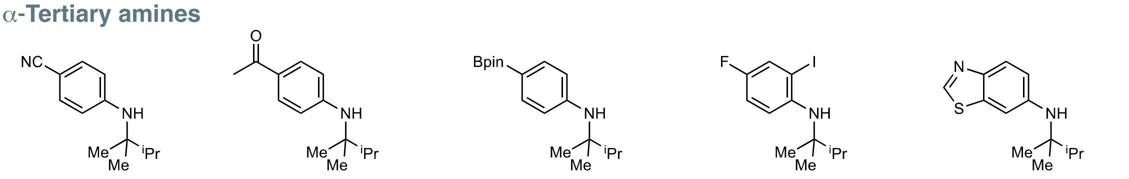

55% AY | 66% AY | 75% AY decomposed | 21% AY | 5% AY

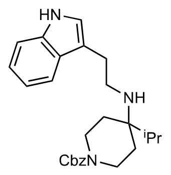

44% AY

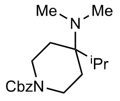

0%

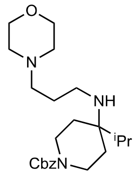

0%

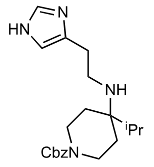

0%

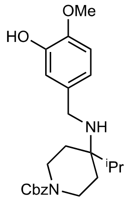

0%

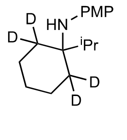

69% IY

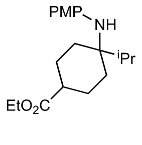

53% AY

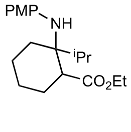

12% AY

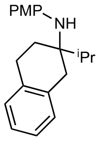

9% AY

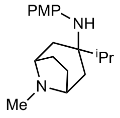

13% AY

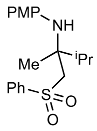

16% AY

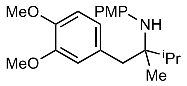

38% AY

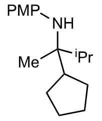

14% AY

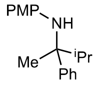

0%

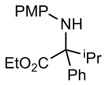

trace

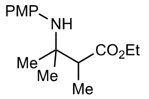

50% AY

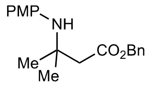

14% AY

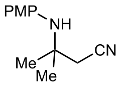

6% AY

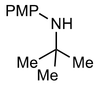

trace

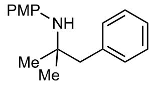

0%

Amino-oxetanes

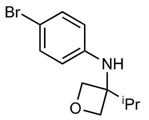

62% AY

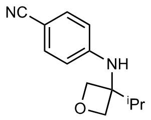

40% AY

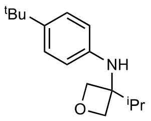

20% AY

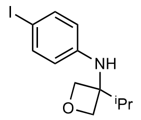

30% AY

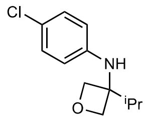

34% AY

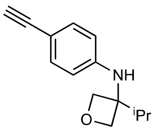

0%

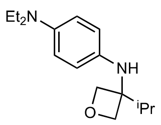

trace

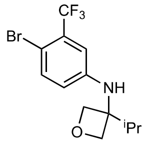

27% AY

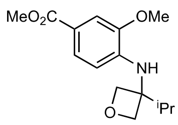

trace

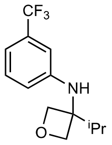

30% AY

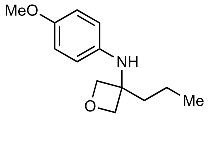

36% AY

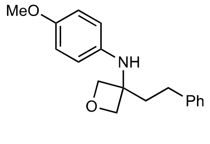

21% AY

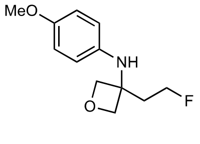

10% AY

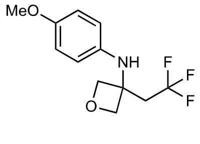

30% AY

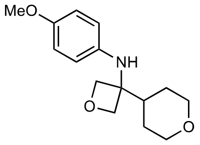

36% AY

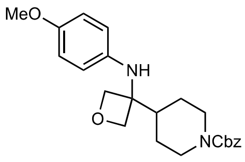

17% AY

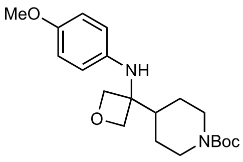

23% AY

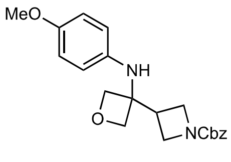

0%

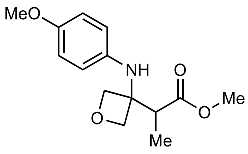

0%

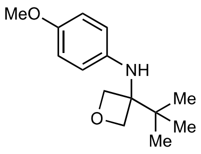

0%

12. References

1. Kumar, R.; Flodén, N. J.; Whitehurst, W. G.; Gaunt, M. J. A General Carbonyl Alkylative Amination for Tertiary Amine Synthesis. *Nature* **2020**, 581, 415–420.

2. Li, X.; Zhang, Y.-K.; Plattner, J. J.; Mao, W.; Alley, M. R. K.; Xia, Y.; Hernandez, V.; Zhou, Y.; Ding, C. Z.; Li, J.; Shao, Z.; Zhang, H.; Xu, M. Synthesis and Antibacterial Evaluation of a Novel Tricyclic Oxaborole-Fused Fluoroquinolone. *Bioorganic & Medicinal Chemistry Letters* **2013**, 23, 963–966.

3. Wulkesch, C.; Czekelius, C. Straightforward Synthesis of Fluorinated Enals via Photocatalytic α-Perfluoroalkenylation of Aldehydes. *J. Org. Chem.* **2021**, 86, 7425–7438.

4. Bezagu, M.; Errico, C.; Chaulot-Talmon, V.; Monti, F.; Tanter, M.; Tabeling, P.; Cossy, J.; Arseniyadis, S.; Couture, O. High Spatiotemporal Control of Spontaneous Reactions Using Ultrasound-Triggered Composite Droplets. *J. Am. Chem. Soc.* **2014**, 136, 7205–7208.

5. Mujahid, M.; Korpe, G. V.; Deshmukh, S. P.; Bhadange, S. G.; Muthukrishnan, M. An Alternative Synthesis of the CNS Stimulant Prolintane. *Arkivoc 2019*, **2019** (5), 292–297.

6. Garivet, G.; Hofer, W.; Konitsiotis, A.; Klein, C.; Kaiser, N.; Mejuch, T.; Fansa, E.; Alsaabi, R.; Wittinghofer, A.; Bastiaens, P. I. H.; Waldmann, H. Small-Molecule Inhibition of the UNC-Src Interaction Impairs Dynamic Src Localization in Cells. *Cell Chemical Biology* **2019**, 26, 842-851.e7.

7. Hsiao, Y.-T.; Beadle, J.; Pascoe, C.; Annadate, R.; Vederas, J. C. Decarboxylative Radical Addition to Methylideneoxazolidinones for Stereocontrolled Synthesis of Selectively Protected Diamino Diacids. *Org. Lett.* **2021**, 23, 7270–7273.

8. Qin, T.; Cornella, J.; Li, C.; Malins, L. R.; Edwards, J. T.; Kawamura, S.; Maxwell, B. D.; Eastgate, M. D.; Baran, P. S. A General Alkyl-Alkyl Cross-Coupling Enabled by Redox-Active Esters and Alkylzinc Reagents. *Science* **2016**, 352 (6287), 801–805.

9. Ni, S.; Garrido-Castro, A. F.; Merchant, R. R.; de Gruyter, J. N.; Schmitt, D. C.; Mousseau, J. J.; Gallego, G. M.; Yang, S.; Collins, M. R.; Qiao, J. X.; Yeung, K.-S.; Langley, D. R.; Poss, M. A.; Scola, P. M.; Qin, T.; Baran, P. S. A General Amino Acid Synthesis Enabled by Innate Radical Cross-Coupling. *Angew. Chem. Int. Ed.* **2018**, 57 (44), 14560–14565.

10. Zhang, Y.; Zhang, D. Cu-Photoredox-Catalyzed C(Sp)–C(Sp3) Coupling of Redox-Active Esters with Terminal Alkynes. *Org. Biomol. Chem.* **2020**, 18, 4479–4483.

11. Chen, T.; Zhang, H.; Mykhailiuk, P. K.; Merchant, R. R.; Smith, C. A.; Qin, T.; Baran, P. S. Quaternary Centers by Nickel-Catalyzed Cross-Coupling of Tertiary Carboxylic Acids and (Hetero)Aryl Zinc Reagents. *Angew. Chem. Int. Ed.* **2019**, 58, 2454–2458.

12. Bosque, I.; Bach, T. 3-Acetoxyquinuclidine as Catalyst in Electron Donor–Acceptor Complex-Mediated Reactions Triggered by Visible Light. *ACS Catal.* **2019**, 9, 9103–9109.

13. Zhang, Z.; Górski, B.; Leonori, D. Merging Halogen-Atom Transfer (XAT) and Copper Catalysis for the Modular Suzuki–Miyaura-Type Cross-Coupling of Alkyl Iodides and Organoborons. *J. Am. Chem. Soc.* **2022**, 144, 1986–1992.

14. Pinaud, M.; Le Gall, E.; Presset, M. Mixed Aliphatic Organozinc Reagents as Nonstabilized C sp 3 -Nucleophiles in the Multicomponent Mannich Reaction. *J. Org. Chem.* **2022**, 87, 4961–4964.

15. Levin, V. V.; Zemtsov, A. A.; Struchkova, M. I.; Dilman, A. D. Reactions of Difluorocarbene with Organozinc Reagents. *Org. Lett.* **2013**, 15, 917–919.

16. Krasovskiy, A.; Knochel, P. Convenient Titration Method for Organometallic Zinc, Magnesium, and Lanthanide- Reagents. *Synthesis 2006*, **2006** (5), 890–891.

17. Vogler, T.; Studer, A. Applications of TEMPO in Synthesis. *Synthesis 2008*, **2008** (13), 1979–1993.

18. Nagashima, T.; Curran, D. P. Reactions of Tempo with Alkylsamarium and Other Organometallic Reagents. *Synlett 1996*, **1996** (4), 330–332.

19. Mancheño, O. G.; Stopka, T. TEMPO Derivatives as Alternative Mild Oxidants in Carbon–Carbon Coupling Reactions. *Synthesis* **2013**, 45, 1602–1611.

20. Budny-Godlewski, K.; Kubicki, D.; Justyniak, I.; Lewiński, J. A New Look at the Reactivity of TEMPO toward Diethylzinc. *Organometallics* **2014**, 33 (19), 5093–5096.

21. Nakamura, T.; Busfield, W. K.; Jenkins, I. D.; Rizzardo, E.; Thang, S. H.; Suyama, S. Reaction of Tert -Alkoxyl and Alkyl Radicals with Styrene Studied by the Nitroxide Radical-Trapping Technique. *J. Org. Chem.* **1997**, 62, 5578–5582.

22. Moad, G.; Rizzardo, E.; Solomon, D. H. Selectivity of the Reaction of Free Radicals with Styrene. *Macromolecules* **1982**, 15, 909–914.

23. Citterio, A.; Arnoldi, A.; Minisci, F. Nucleophilic Character of Alkyl Radicals. 18. Absolute Rate Constants for the Addition of Primary Alkyl Radicals to Conjugated Olefins and 1,4-Benzoquinone. *J. Org. Chem.* **1979**, 44, 2674–2682.

24. Examples of styrene and 1,1-diphenylethylene being used as traps for alkyl radicals: a) Xie, J.; Li, J.; Weingand, V.; Rudolph, M.; Hashmi, A. S. K. Intermolecular Photocatalyzed Heck-like Coupling of Unactivated Alkyl Bromides by a Dinuclear Gold Complex. *Chem. Eur. J.* **2016**, 22, 12646–12650. B) Chen, C.; Wang, Z.-J.; Lu, H.; Zhao, Y.; Shi, Z. Generation of Non-Stabilized Alkyl Radicals from Thianthrenium Salts for C–B and C–C Bond Formation. *Nat Commun* **2021**, 12, 4526.c) Ding, H.; Orazov, M. Anodically-Generated Alkyl Radicals Derived from Carboxylic Acids as Reactive Intermediates for Addition to Alkenes. *ChemElectroChem* **2023**, 10, e202201099.

25. a) Grimm, M. L.; Suleman, N. K.; Hancock, A. N.; Spencer, J. N.; Dudding, T.; Rowshanpour, R.; Castagnoli, N.; Tanko, J. M. Stereoelectronic and Resonance Effects on the Rate of Ring Opening of N -Cyclopropyl-Based Single Electron Transfer Probes. *J. Am. Chem. Soc.* **2020**, 142, 2640–2652. b) Nonhebel, D. C. The Chemistry of Cyclopropylmethyl and Related Radicals. *Chem. Soc. Rev.* **1993**, 22, 347. c) Gladkov, A. A.; Levin, V. V.; Dilman, A. D. Photoredox Promoted Barbier-Type Reaction of Alkyl Iodides with N -Alkyl and N -Aryl Imines. *J. Org. Chem.* **2023**, 88, 1260–1269.

26. a) Heinz, C.; Lutz, J. P.; Simmons, E. M.; Miller, M. M.; Ewing, W. R.; Doyle, A. G. Ni-Catalyzed Carbon–Carbon Bond-Forming Reductive Amination. *J. Am. Chem. Soc.* **2018**, 140, 2292–2300. b) Pratley, C.; Fenner, S.; Murphy, J. A. Nitrogen-Centered Radicals in Functionalization of Sp 2 Systems: Generation, Reactivity, and Applications in Synthesis. *Chem. Rev.* **2022**, 122 (9), 8181–8260. c) Newcomb, Ma.; Burchill, M. T.; Deeb, T. M. A Mechanistic Probe Study of Potential Electron-Transfer Reactions of Lithium Dialkylamides. *J. Am. Chem. Soc.* **1988**, 110, 6528–6535.

27. a) Jackman, M. M.; Cai, Y.; Castle, S. L. Recent Advances in Iminyl Radical Cyclizations. *Synthesis* **2017**, 49, 1785–1795. b) Morris, S. A.; Wang, J.; Zheng, N. The Prowess of Photogenerated Amine Radical Cations in Cascade Reactions: From Carbocycles to Heterocycles. *Acc. Chem. Res.* **2016**, 49, 1957–1968. c) Newcomb, M.; Deeb, T. M.; Marquardt, D. J. N-Hydroxypyridine-2-Thione Carbamates. IV. A Comparison of 5-Exo Cyclizations of an Aminyl Radical and an Aminium Cation Radical. *Tetrahedron* **1990**, 46, 2317–2328. d) Probing the Diastereoselectivity in the Cyclization of Cationic Aminyl Radicals. *Tetrahedron: Asymmetry* **1999**, 10, 4091–4094. e)

Schmittel, M.; Burghart, A. Understanding Reactivity Patterns of Radical Cations. *Angew. Chem. Int. Ed. Engl.* **1997**, 36, 2550–2589. f) Newcomb, M.; Esker, J. L. Facile Production and Cyclizations of Amidyl Radicals. *Tet. Lett.* **1991**, 32, 1035–1038. g) Newcomb, M.; Marquardt, D. J.; Deeb, T. M. N-Hydroxypyridine-2-Thione Carbamates. v. Syntheses of Alkaloid Skeletons by Aminium Cation Radical Cyclizations. *Tetrahedron* **1990**, 46, 2329–2344. h) Horner, J. H.; Martinez, F. N.; Musa, O. M.; Newcomb, M.; Shahin, H. E. Kinetics of Dialkylaminium Cation Radical Reactions: Radical Clocks, Solvent Effects, Acidity Constants, and Rate Constants for Reactions with Hydrogen Atom Donors. *J. Am. Chem. Soc.* **1995**, 117, 11124–11133. i) Wagner, B. D.; Ruel, G.; Lusztyk, J. Absolute Kinetics of Aminium Radical Reactions with Olefins in Acetonitrile Solution 1. *J. Am. Chem. Soc.* **1996**, 118, 13–19.

^{1}H and ^{13}C Spectral Data

ethyl N-benzyl-N-butylglycinate (5a)

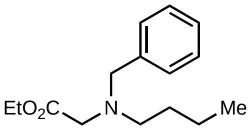

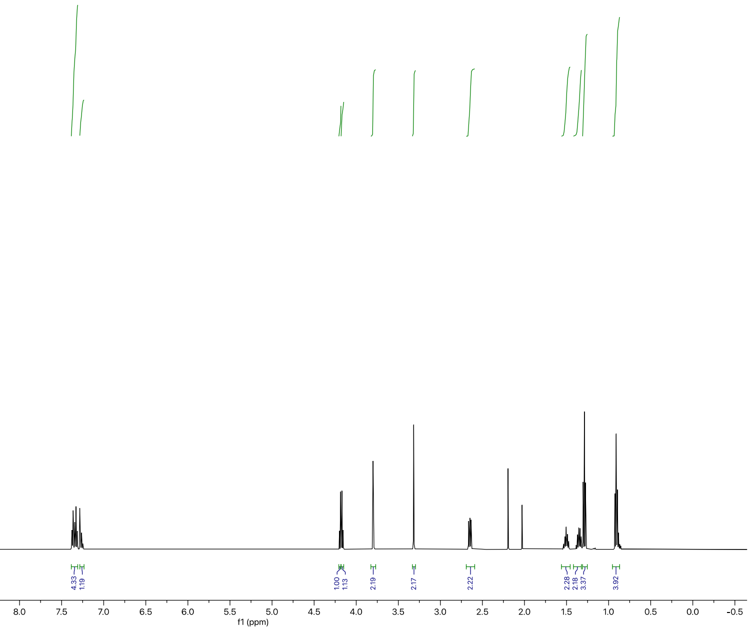

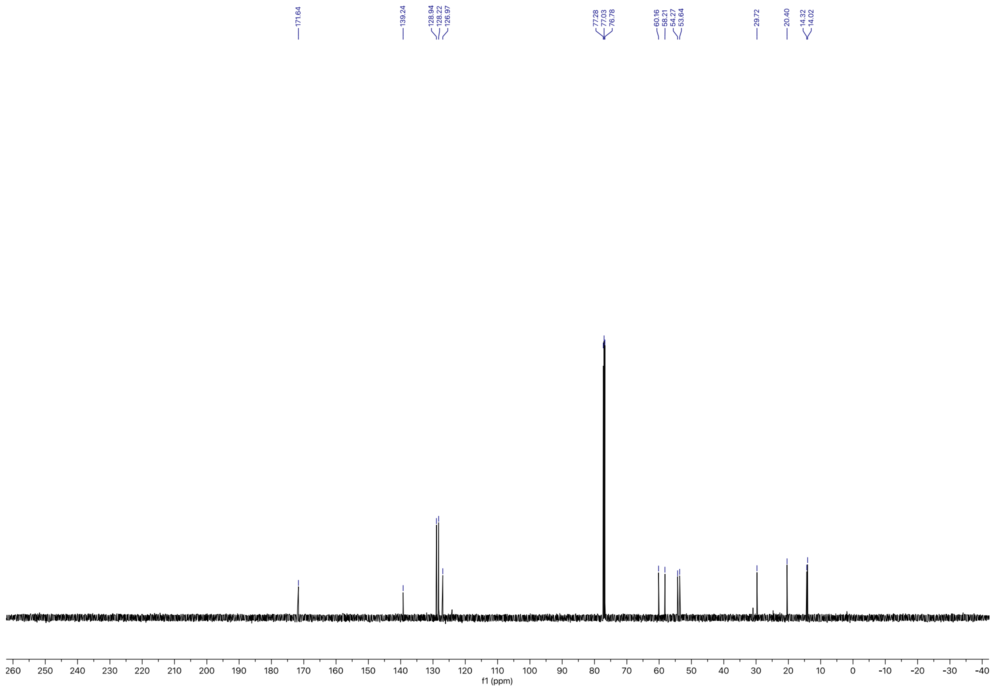

(3-(benzyl(butyl)amino)propanenitrile) (5b)

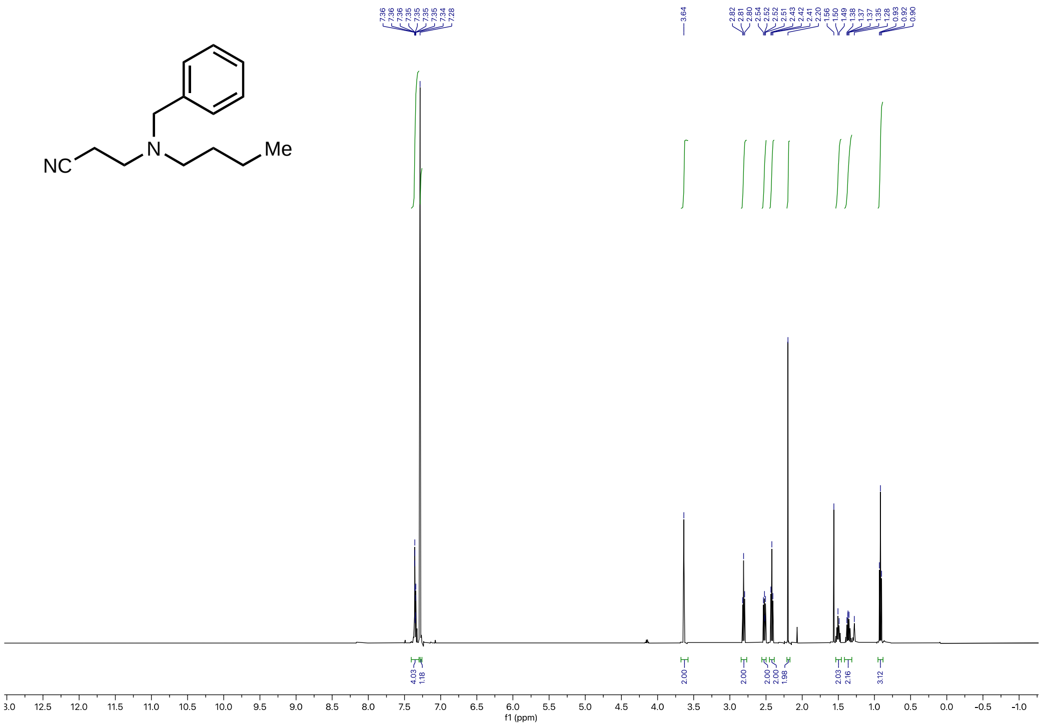

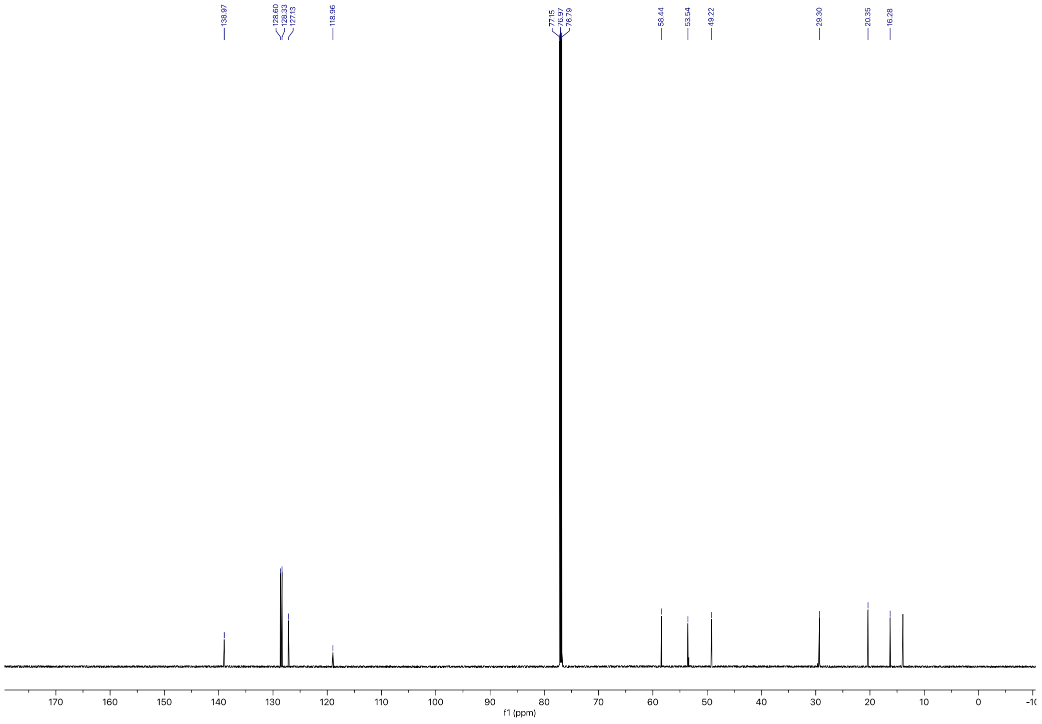

N,N, 4-trimethyl-1-phenylpentan-3-amine (4d)

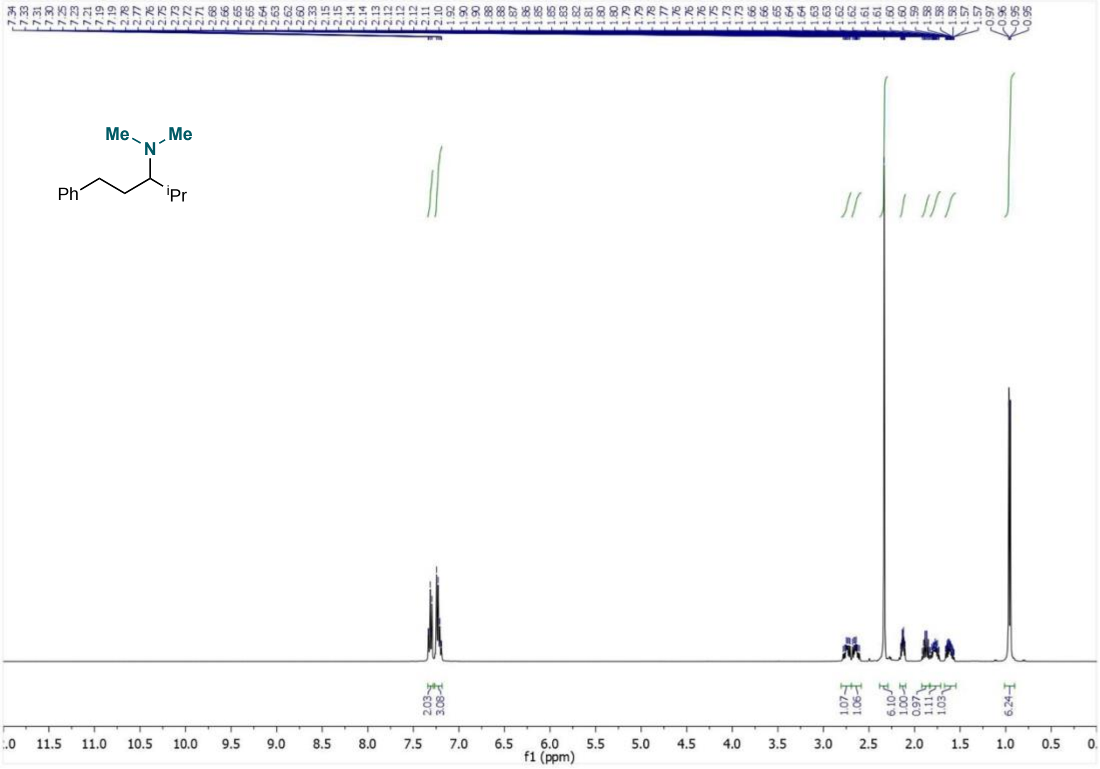

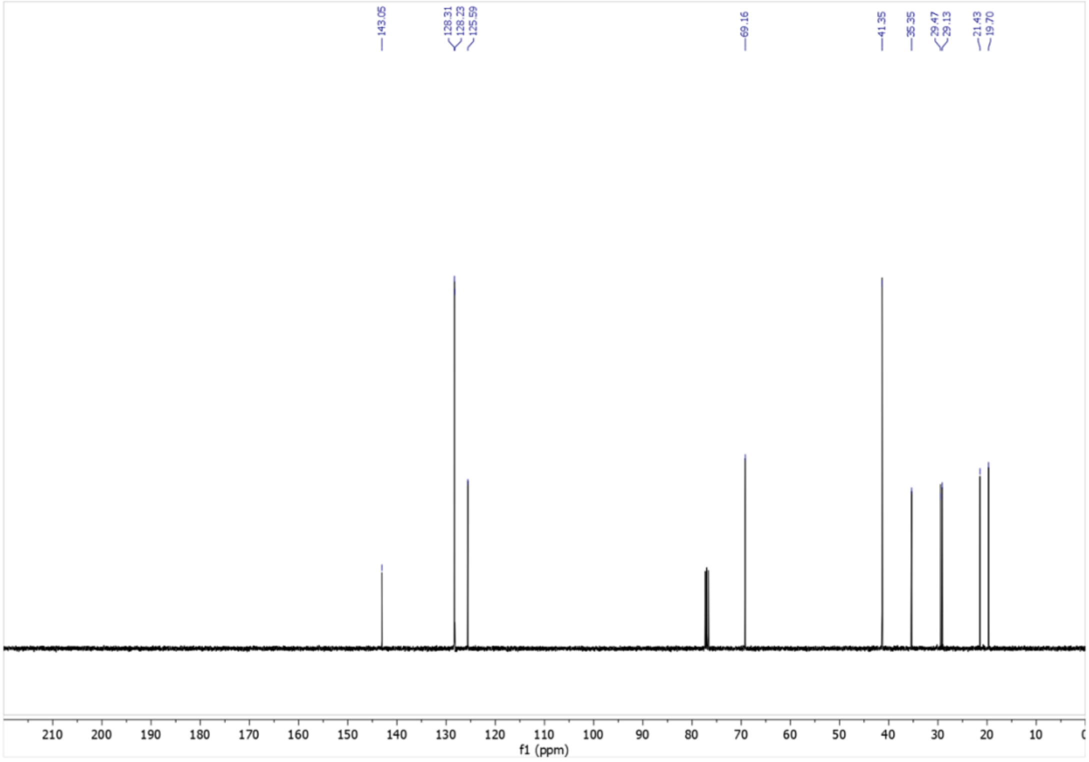

N,N-Dibenzyl-4-methyl-1-phenylpentan-3-amine (4e)

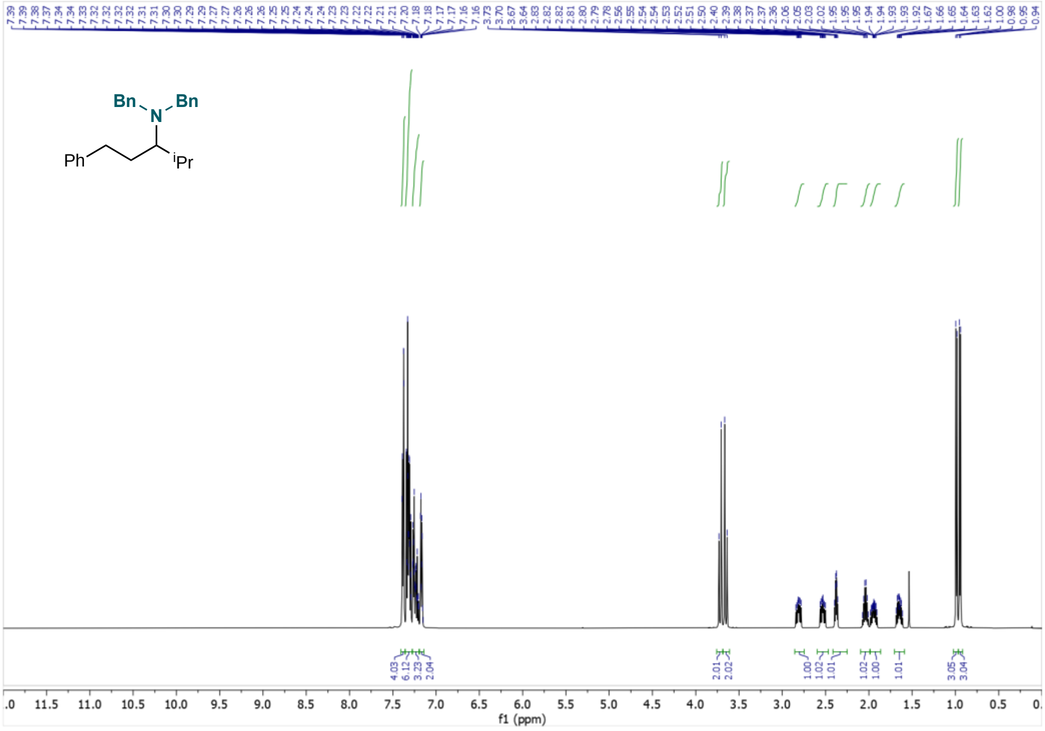

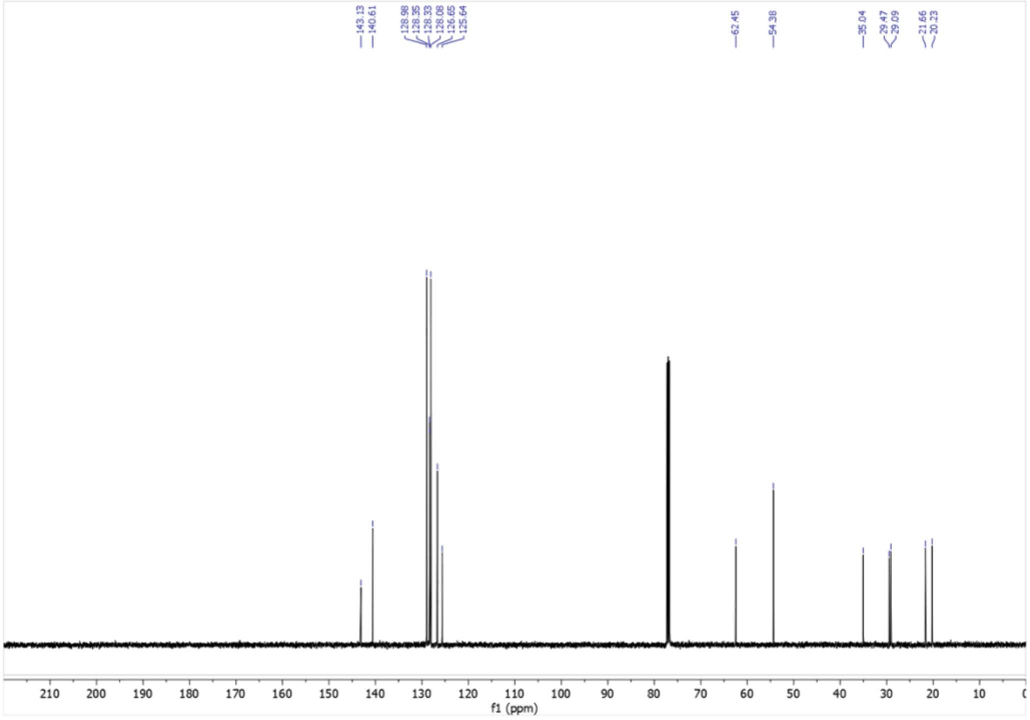

3-(Benzyl(4-methyl-1-phenylpentan-3-yl)amino)propanenitrile (4f)

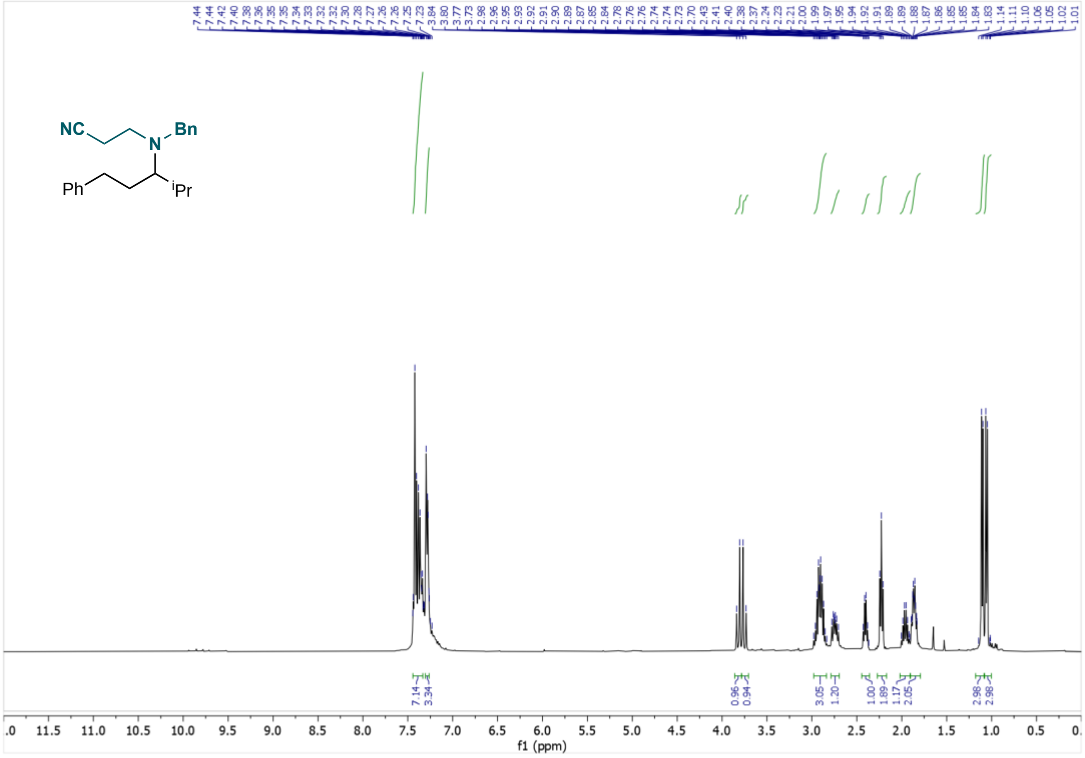

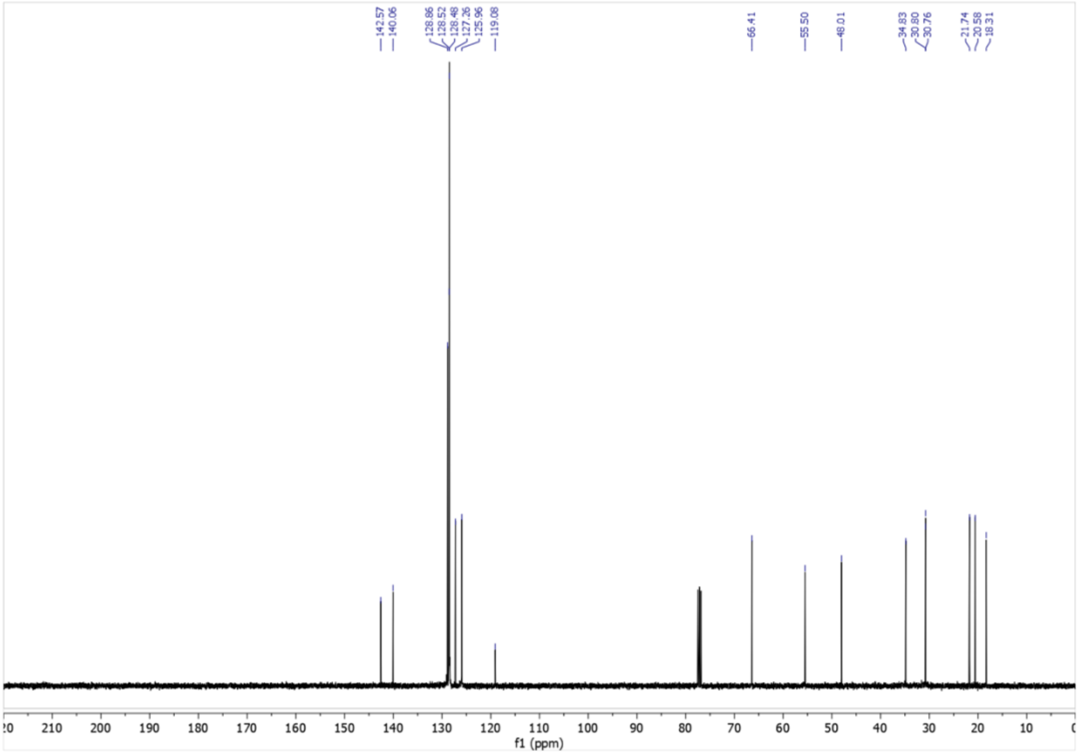

N-benzyl-N-(3,4-dimethoxyphenethyl)-4-methyl-1-phenylpentan-3-amine (4g)

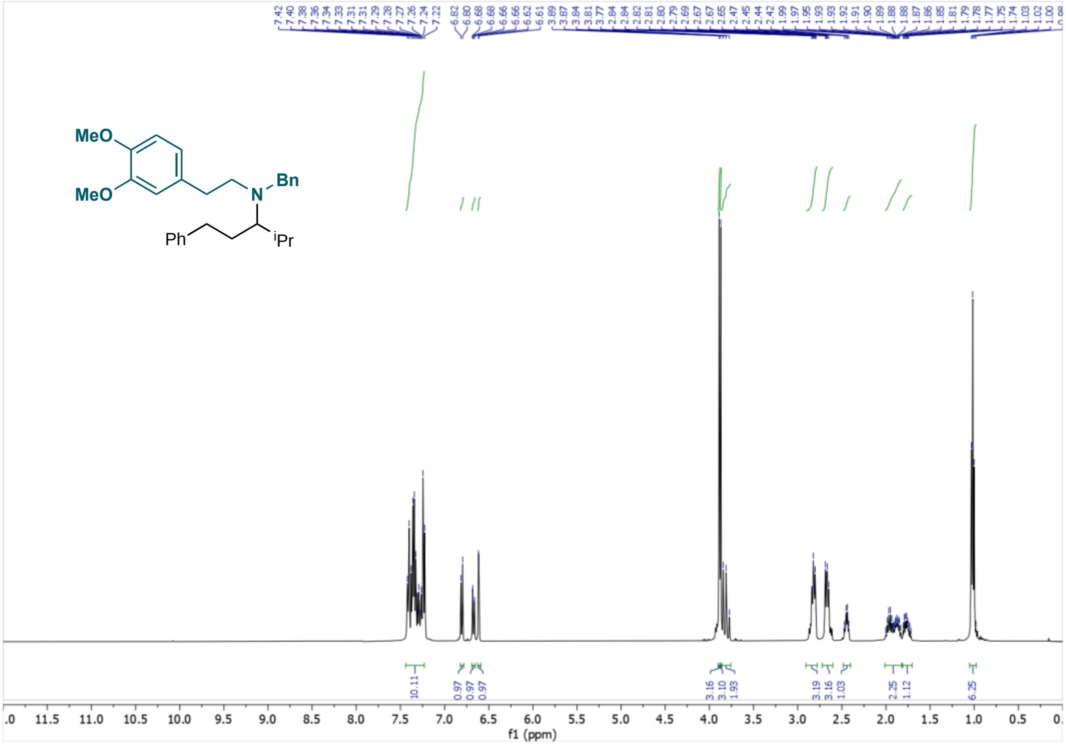

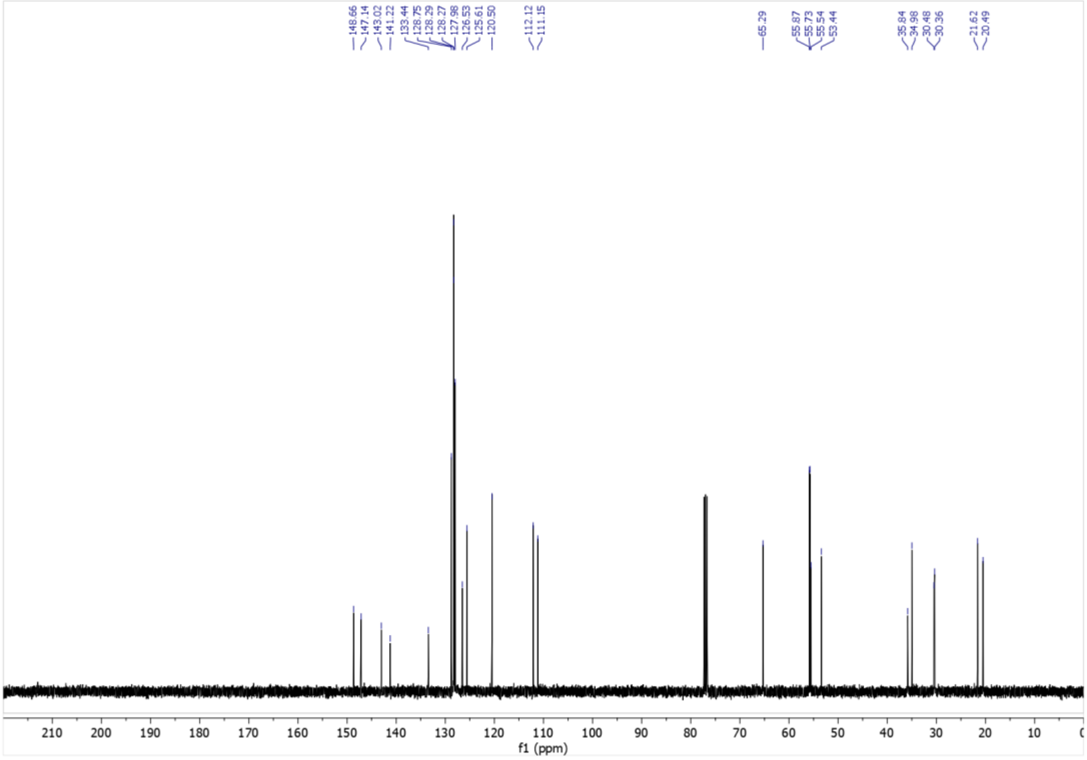

1-(4-Methyl-1-phenylpentan-3-yl)azetidine (4h)

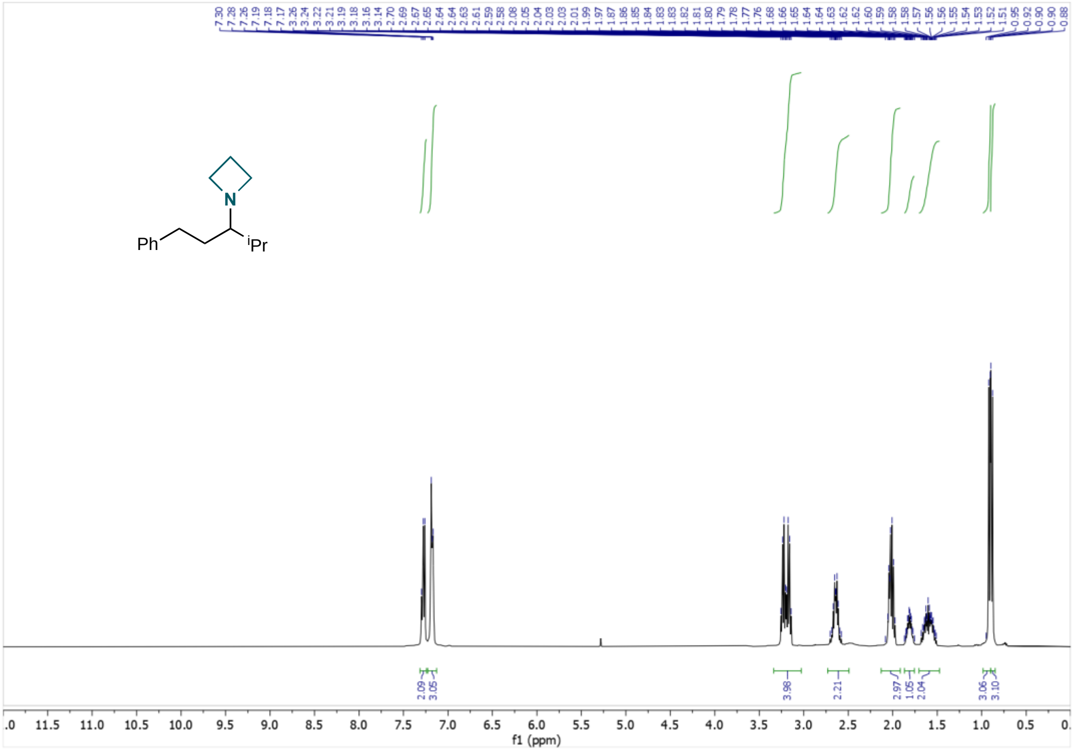

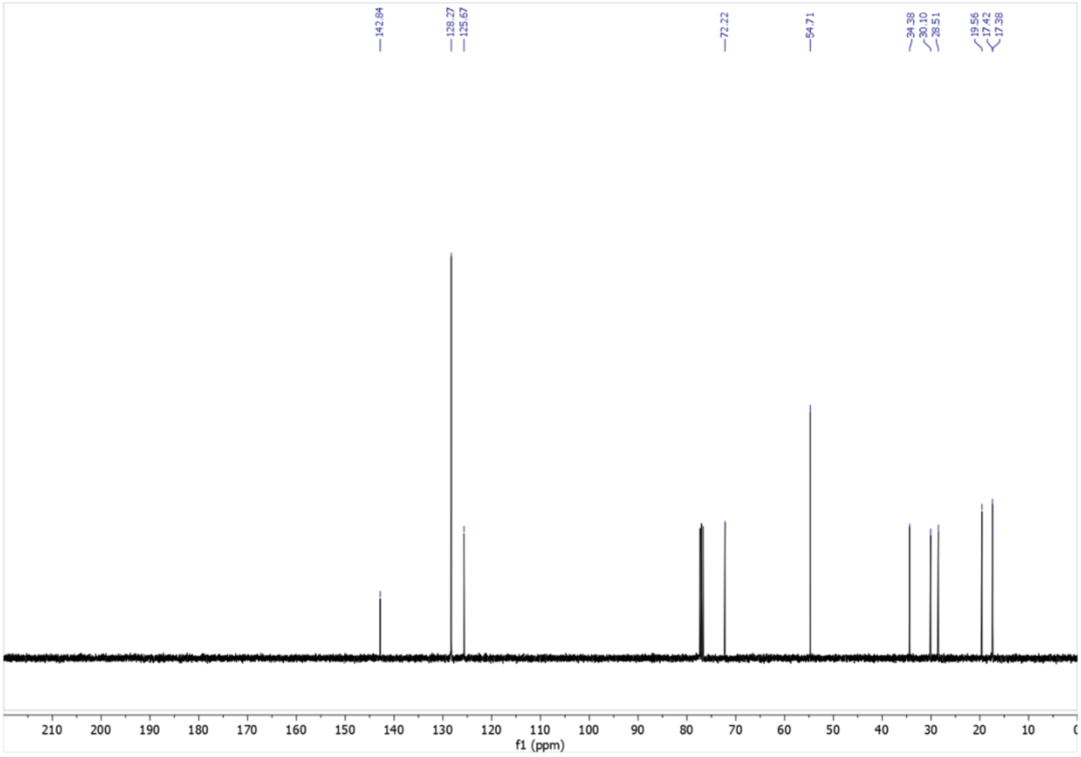

3-Methoxy-1-(4-methyl-1-phenylpentan-3-yl)azetidine (4i)

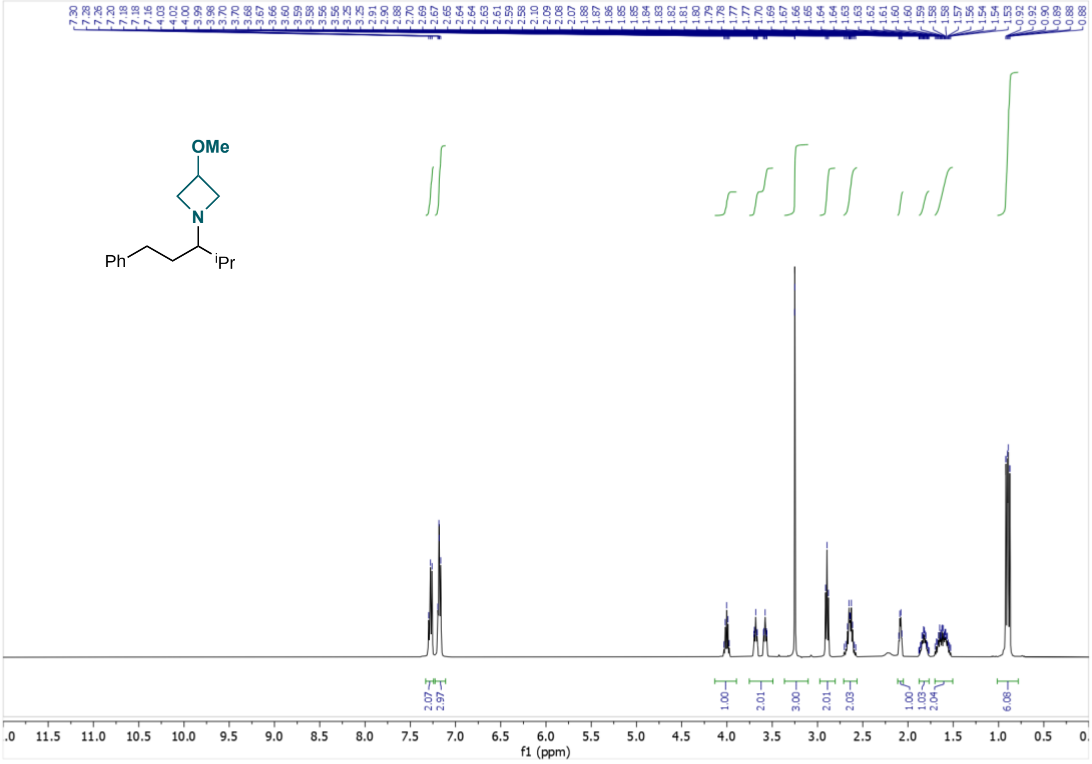

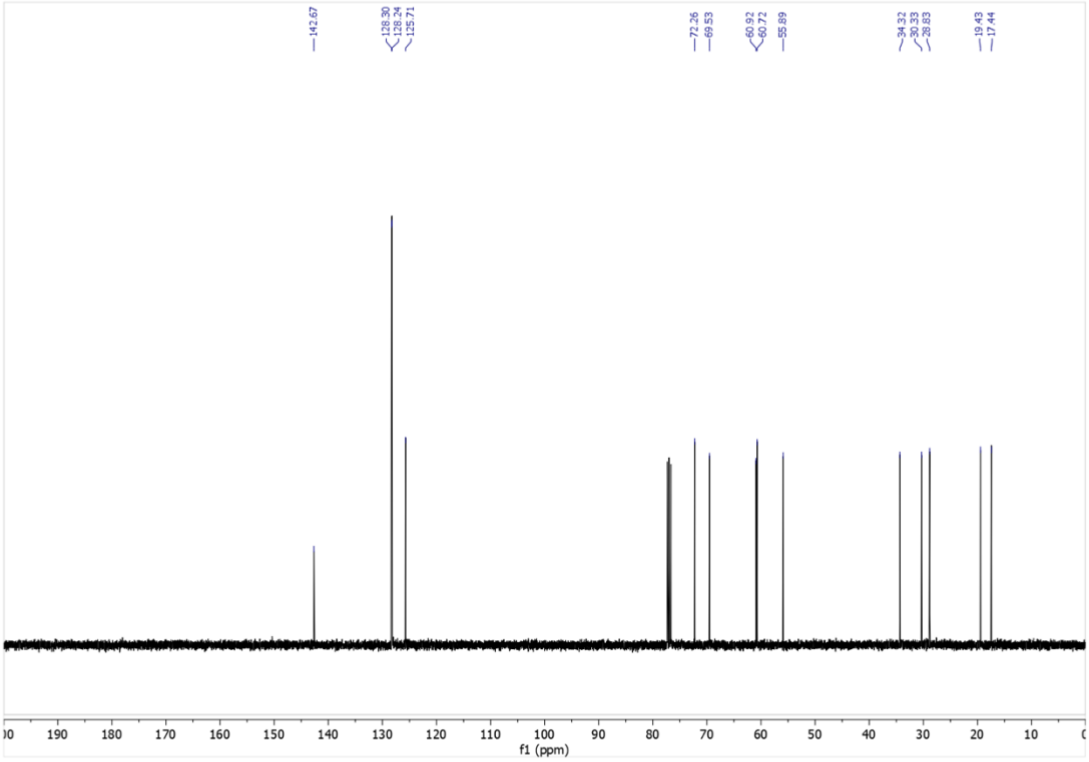

4-Bromo-1-(4-methyl-1-phenylpentan-3-yl)piperidine (4l)

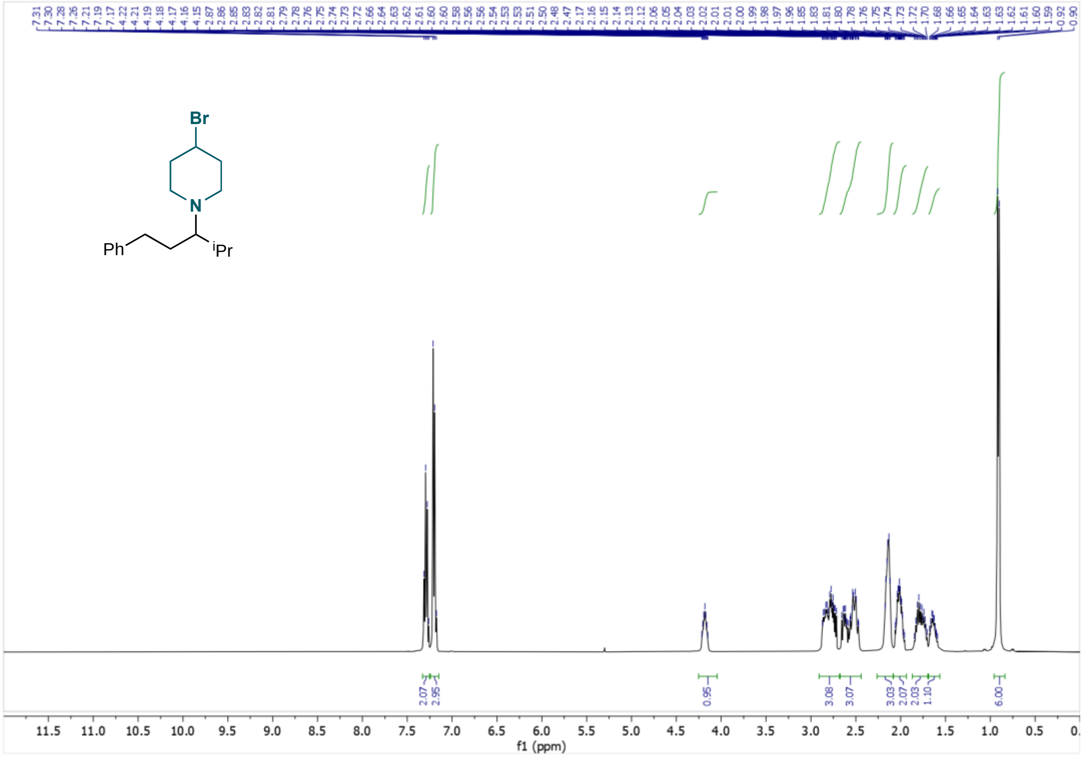

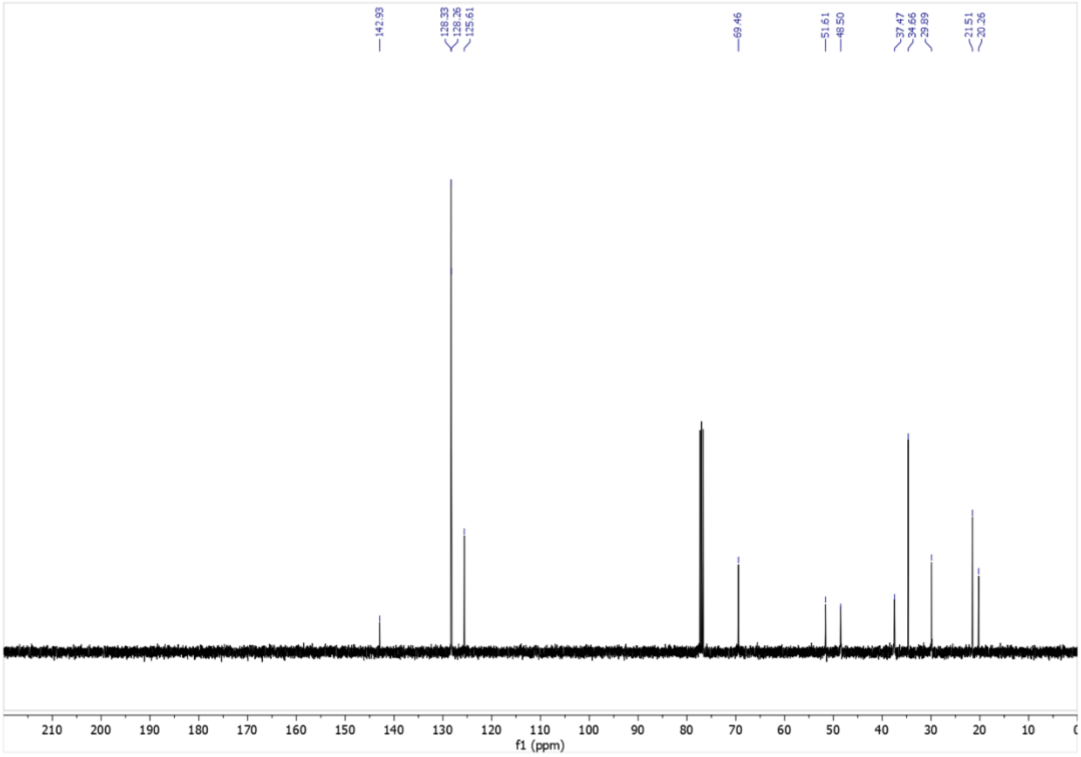

4-(4-Chlorophenyl)-1-(4-methyl-1-phenylpentan-3-yl)piperidin-4-ol (4m)

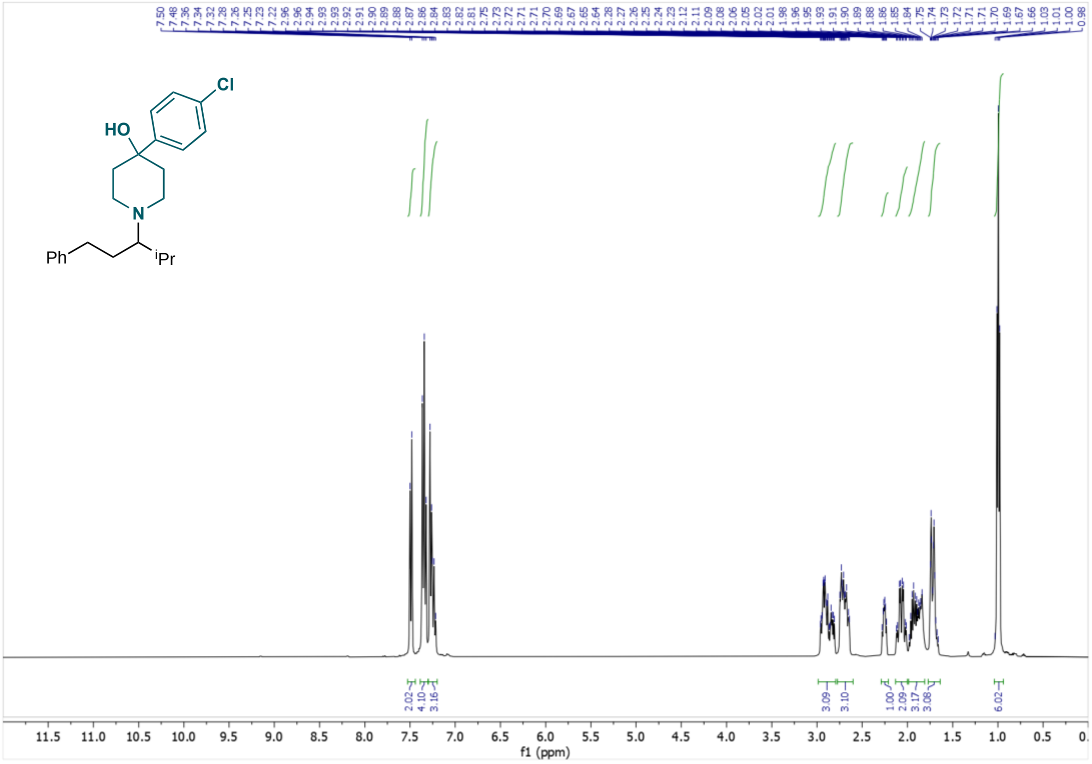

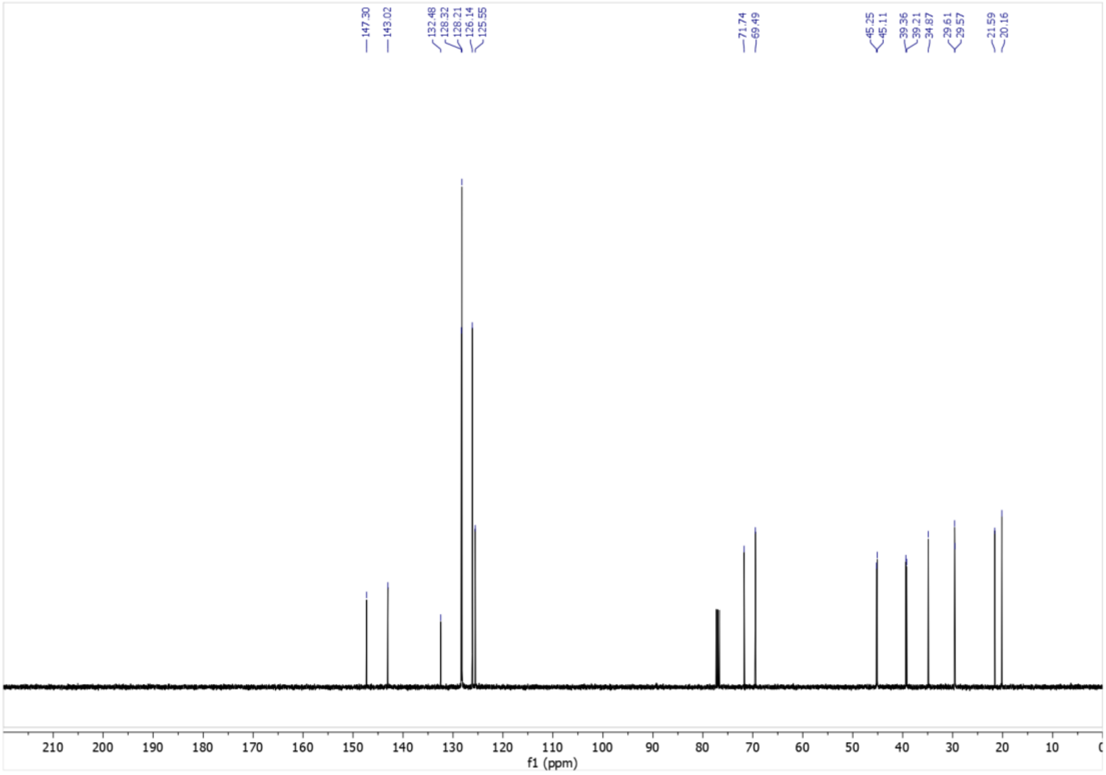

4-(4-methyl-1-phenylpentan-3-yl)morpholine (4n)

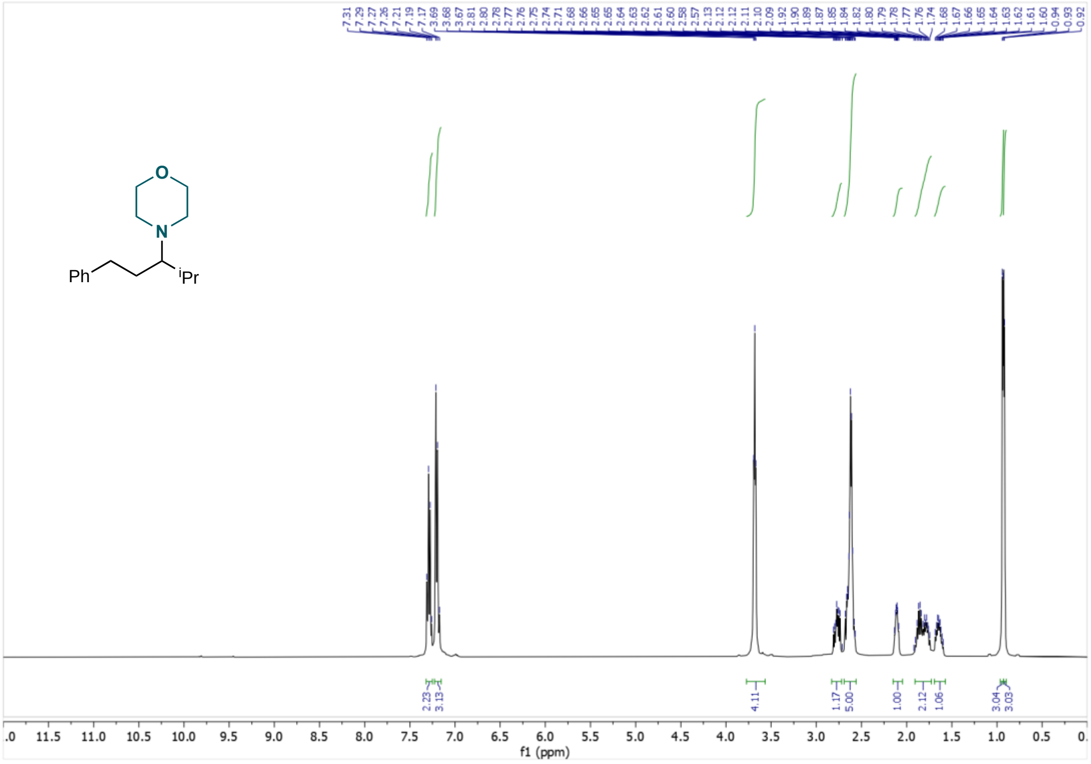

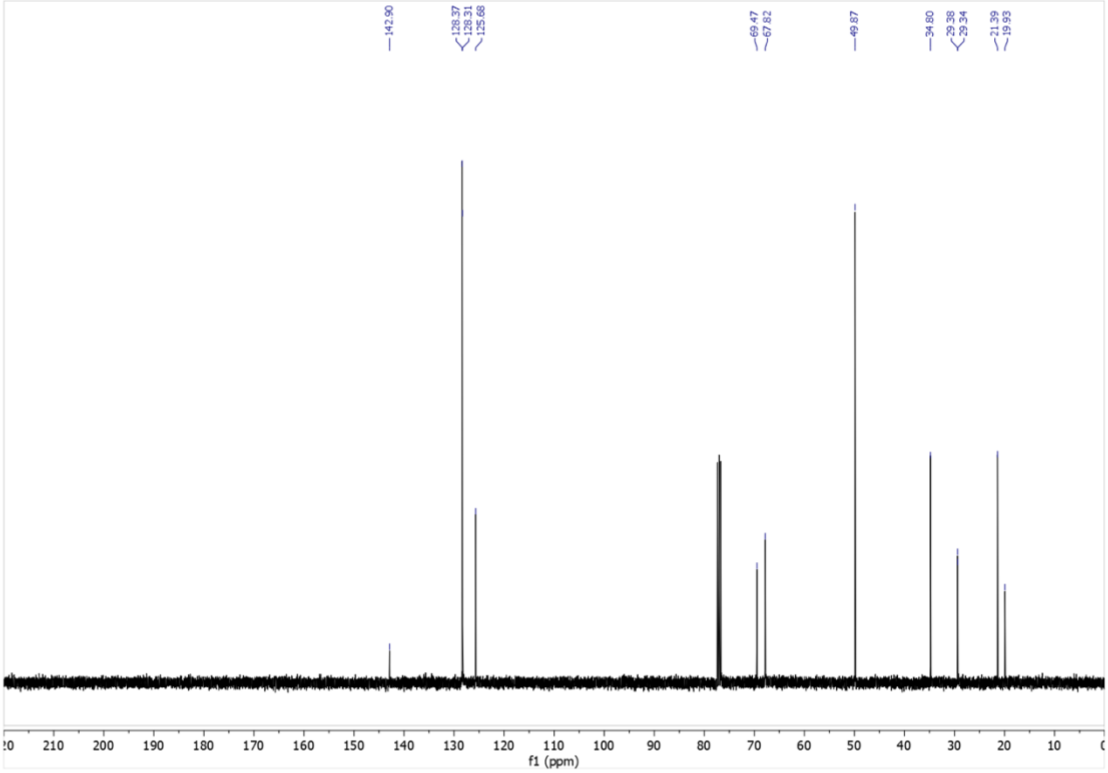

Benzyl 4-(4-methyl-1-phenylpentan-3-yl)piperazine-1-carboxylate (4o)

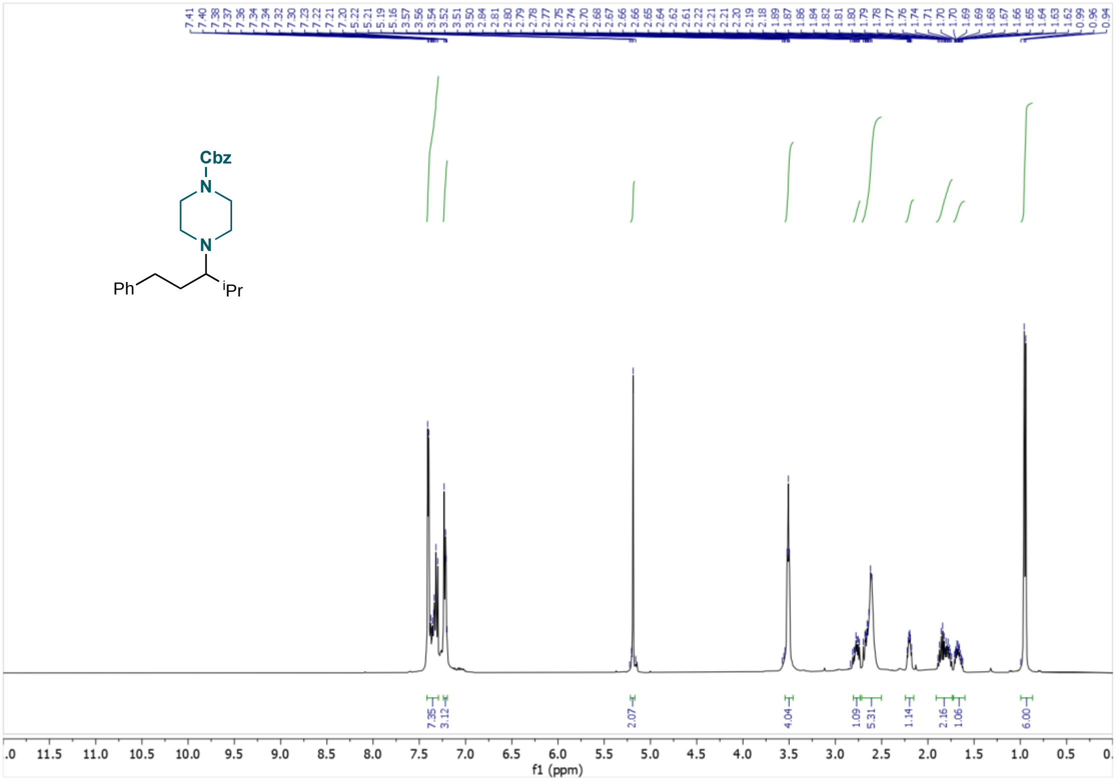

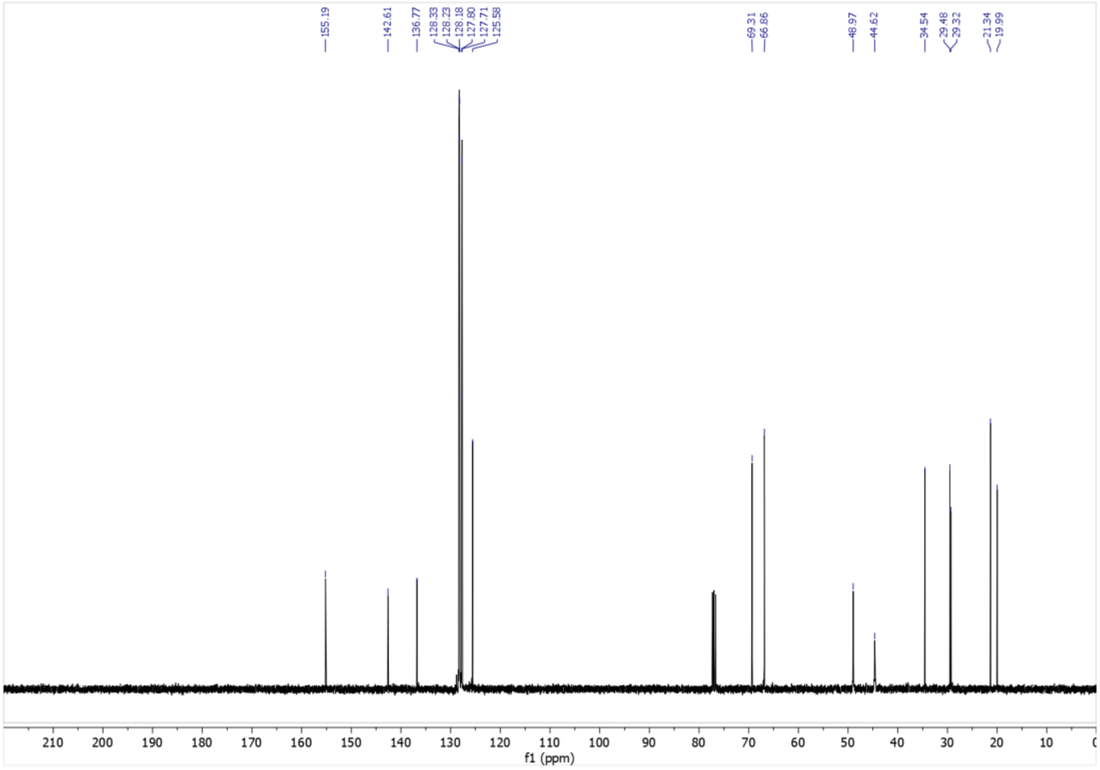

4-(4-Methyl-1-phenylpentan-3-yl)thiomorpholine 1,1-dioxide (4p)

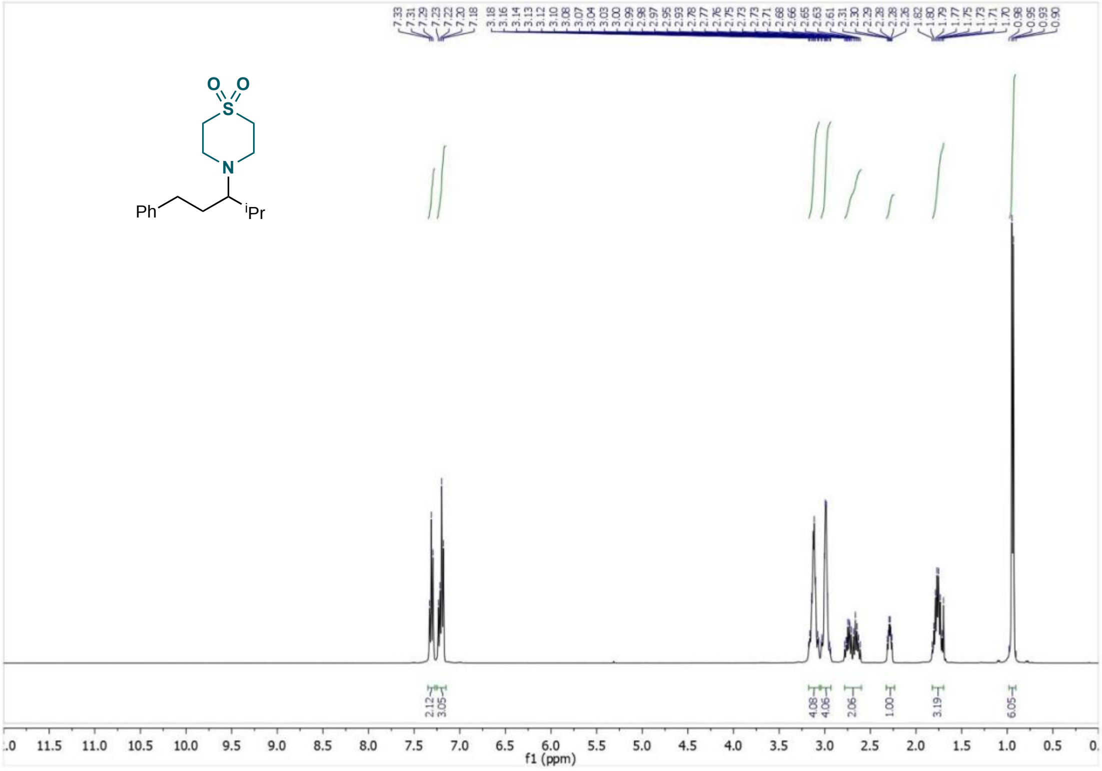

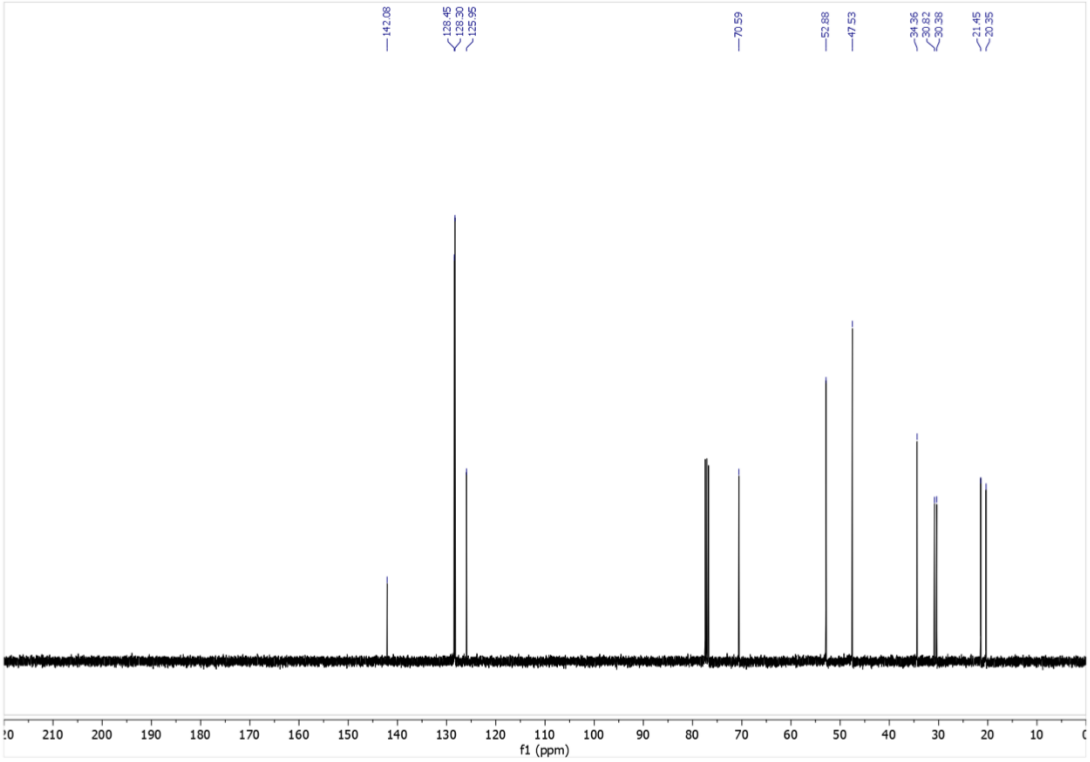

1-(4-Methyl-1-phenylpentan-3-yl)-1,4-diazepan-5-one (4q)

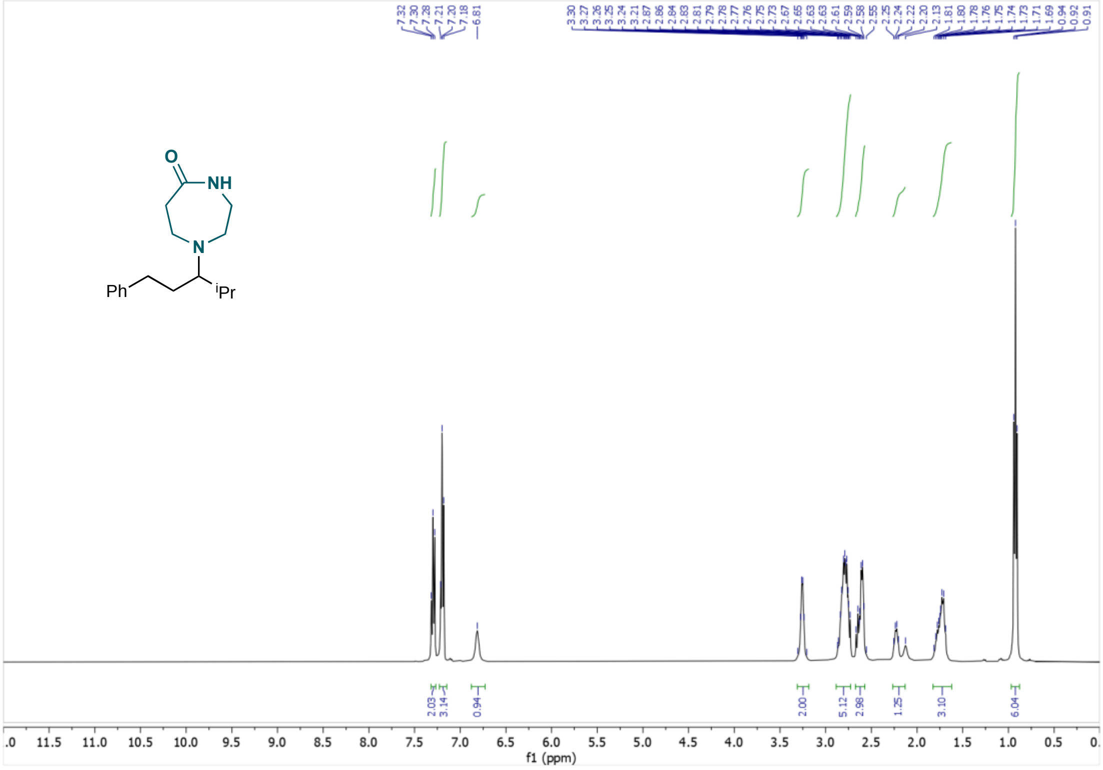

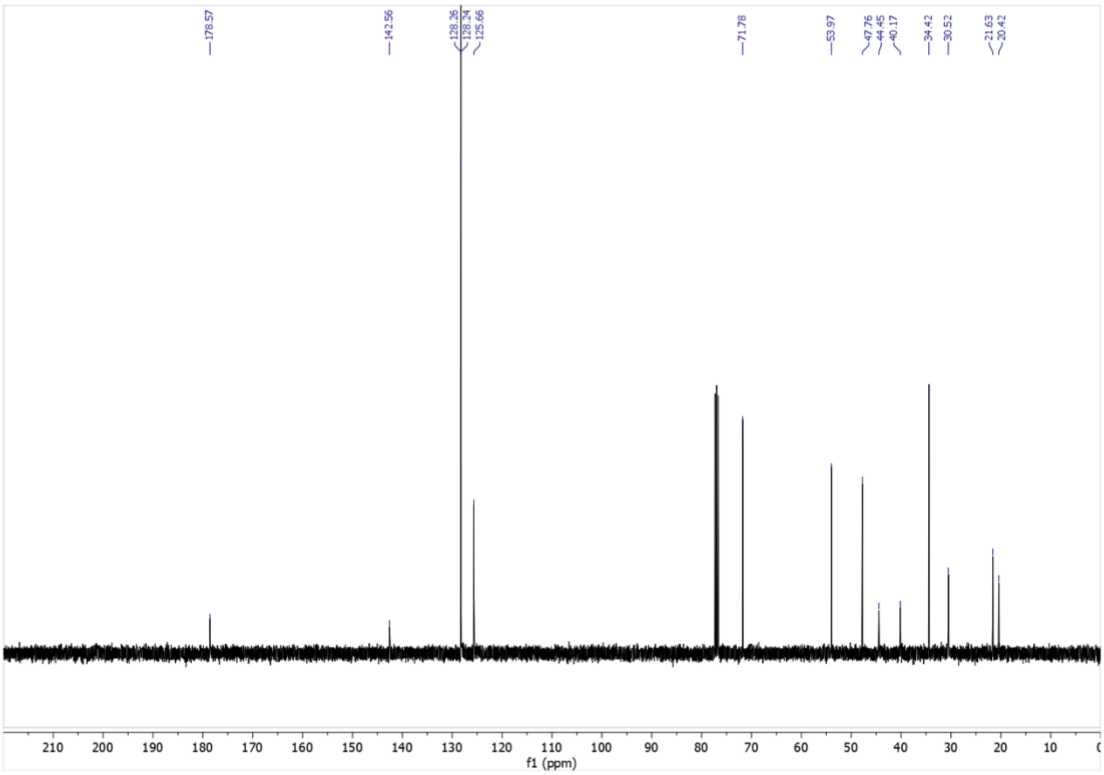

2-(4-Methyl-1-phenylpentan-3-yl)octahydrocyclopenta[c]pyrrole (4s)

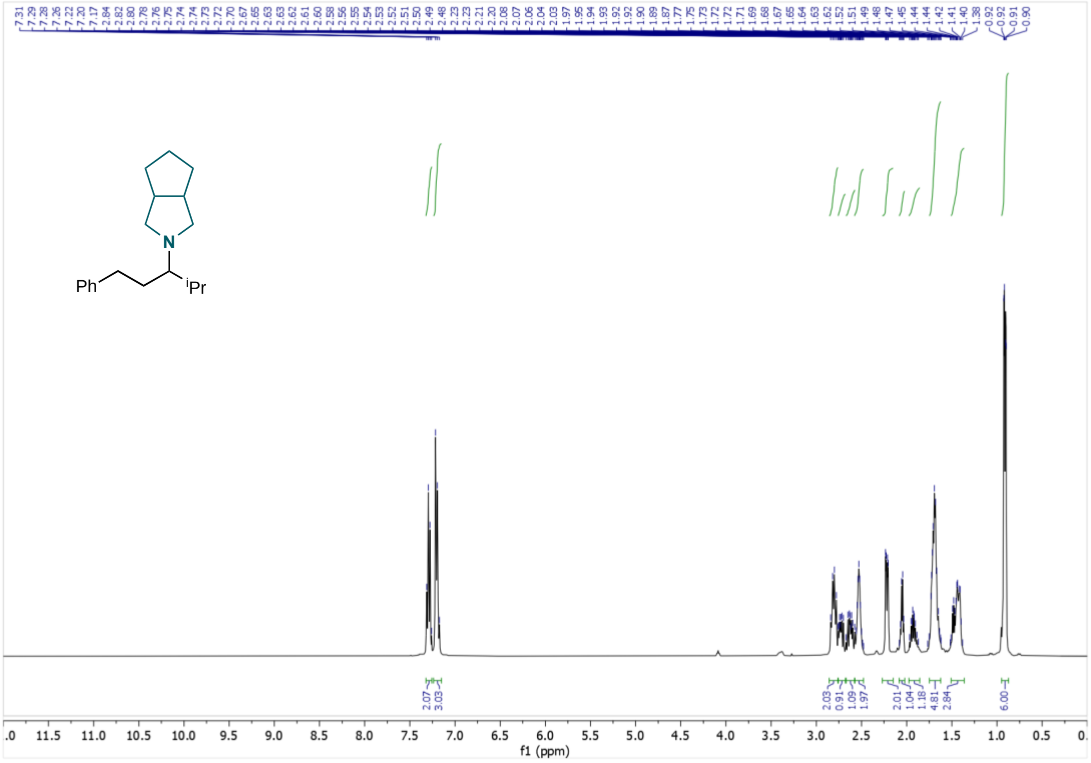

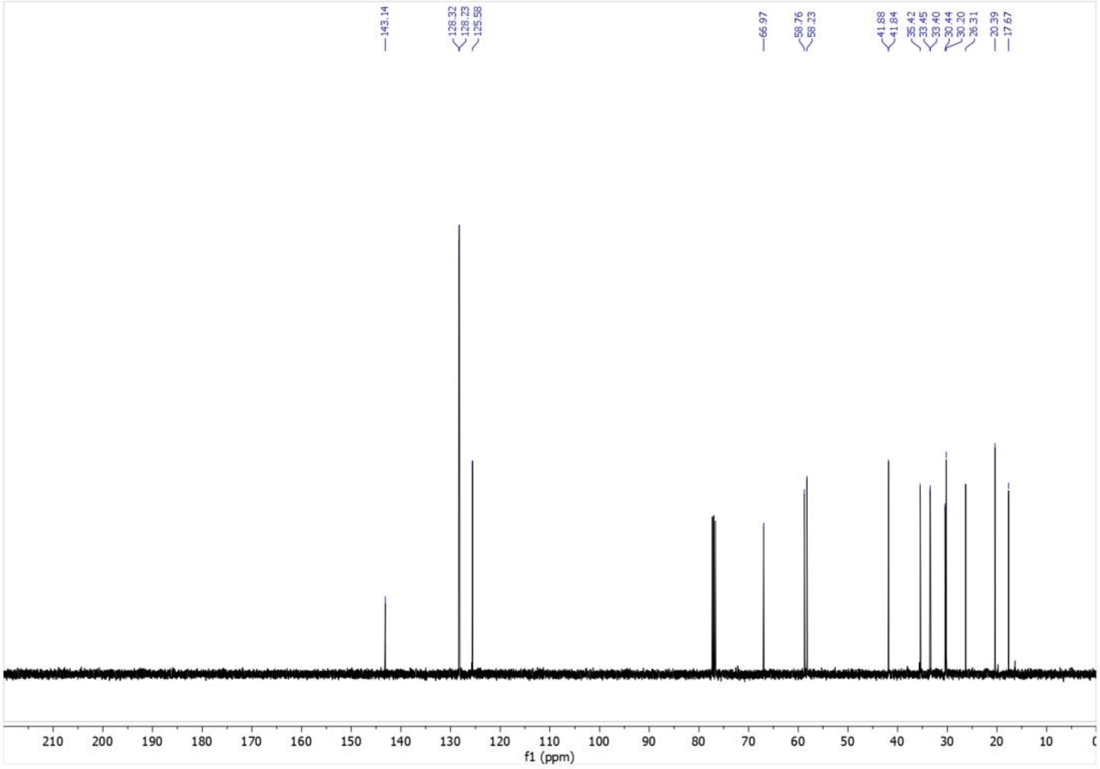

6-(4-Methyl-1-phenylpentan-3-yl)-4,5,6,7-tetrahydrothieno[2,3-c]pyridine (4t)

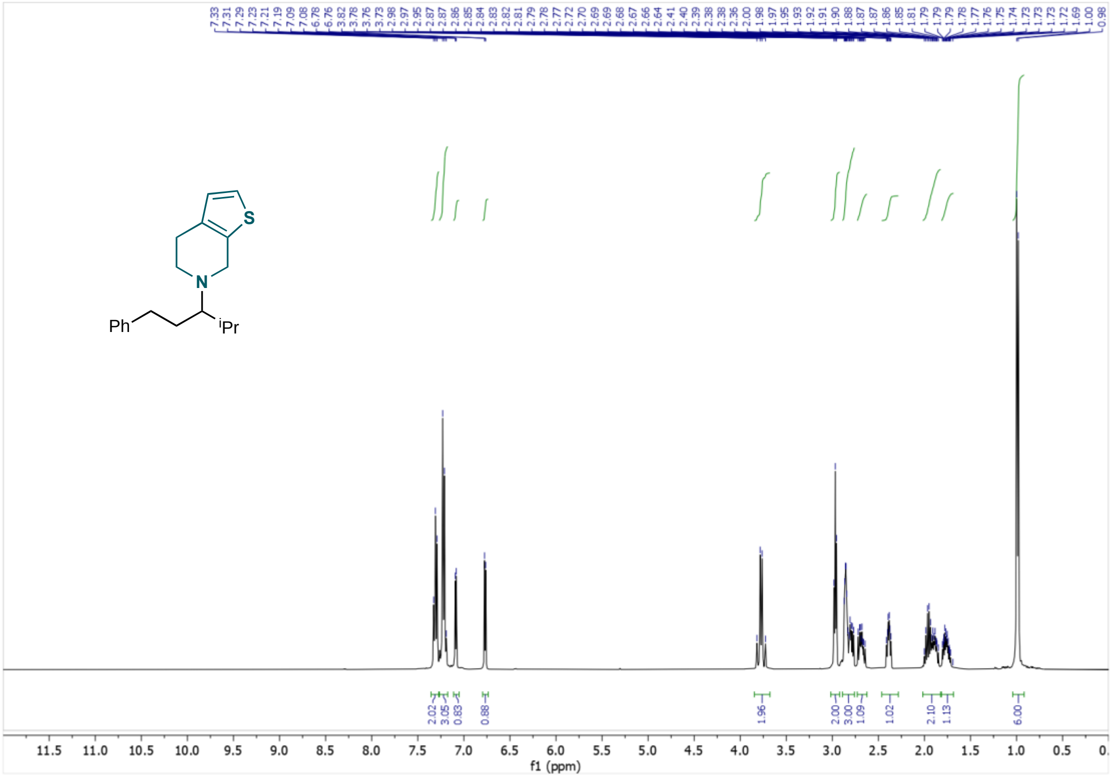

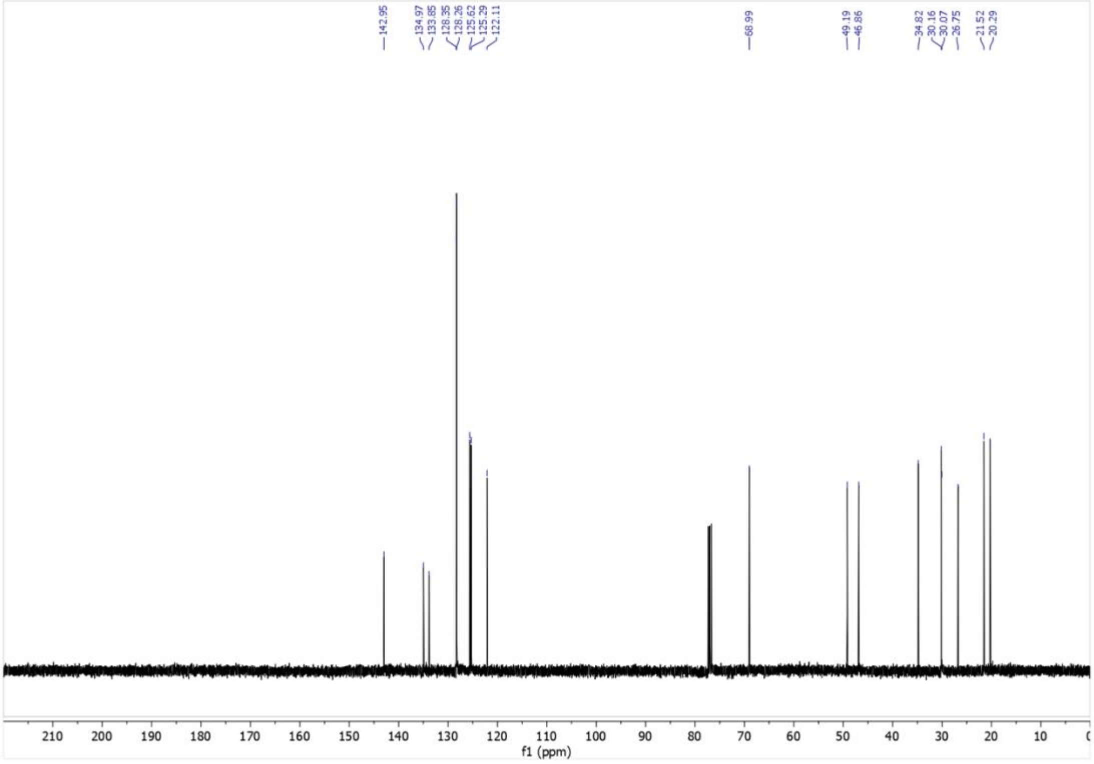

8-(4-Methyl-1-phenylpentan-3-yl)-8-azabicyclo[3.2.1]octan-3-one (4u)

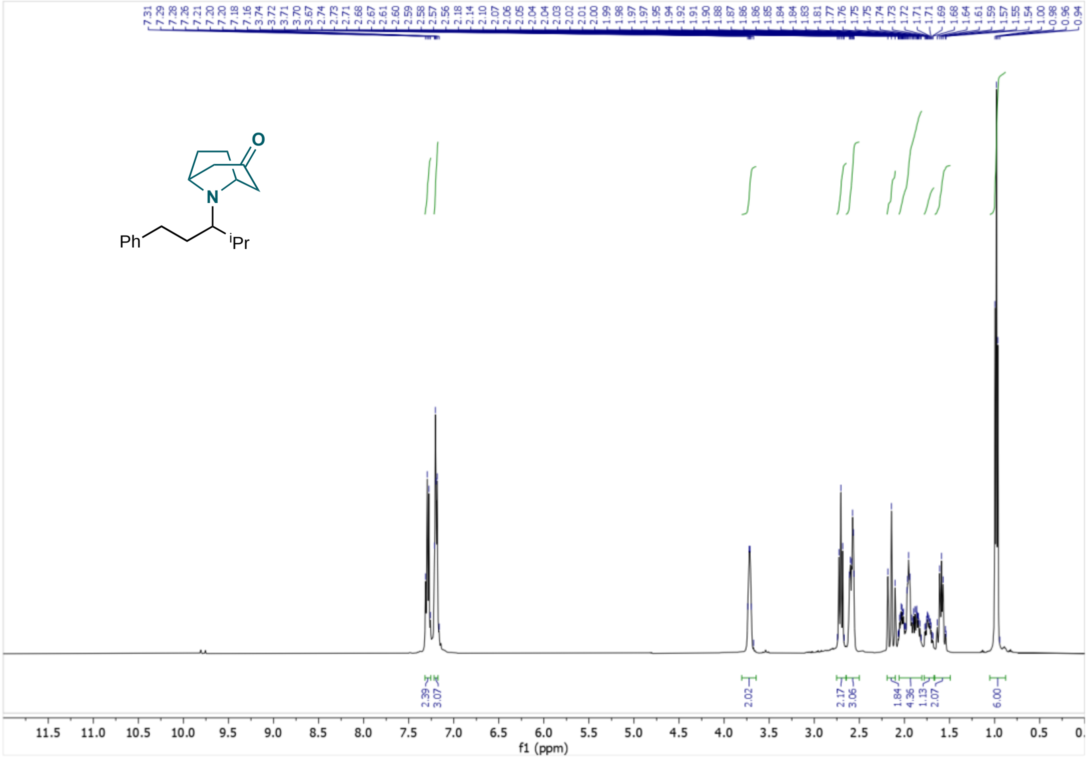

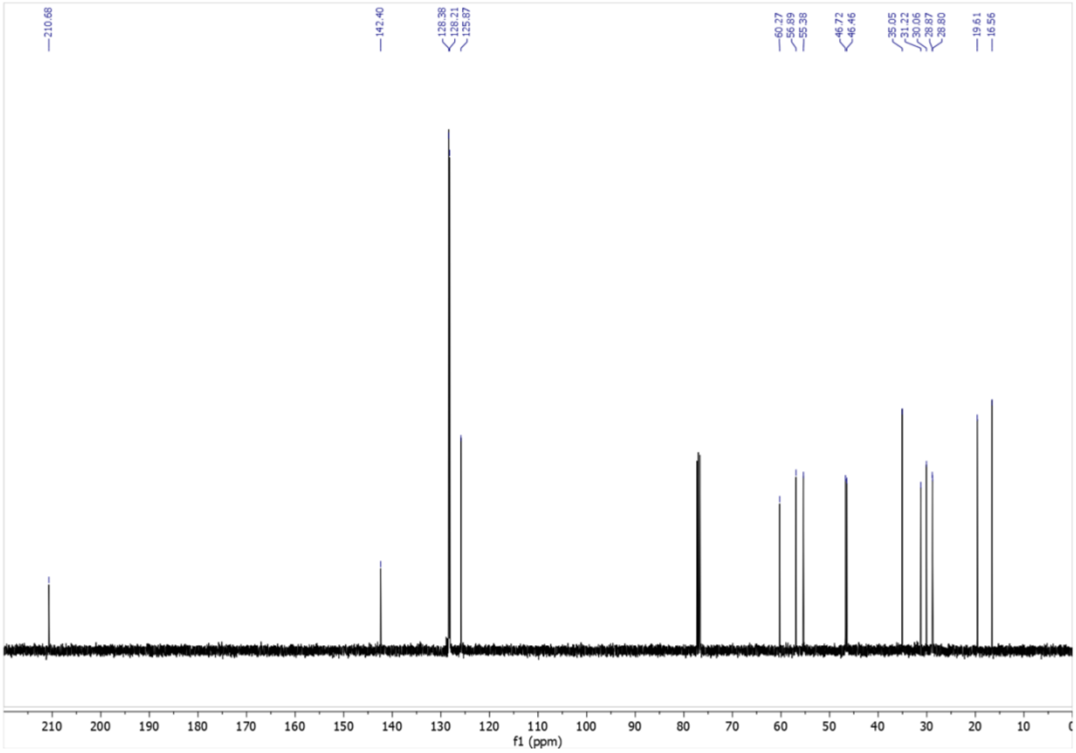

3-(4-Methyl-1-phenylpentan-3-yl)-8-oxa-3-azabicyclo[3.2.1]octane (4v)

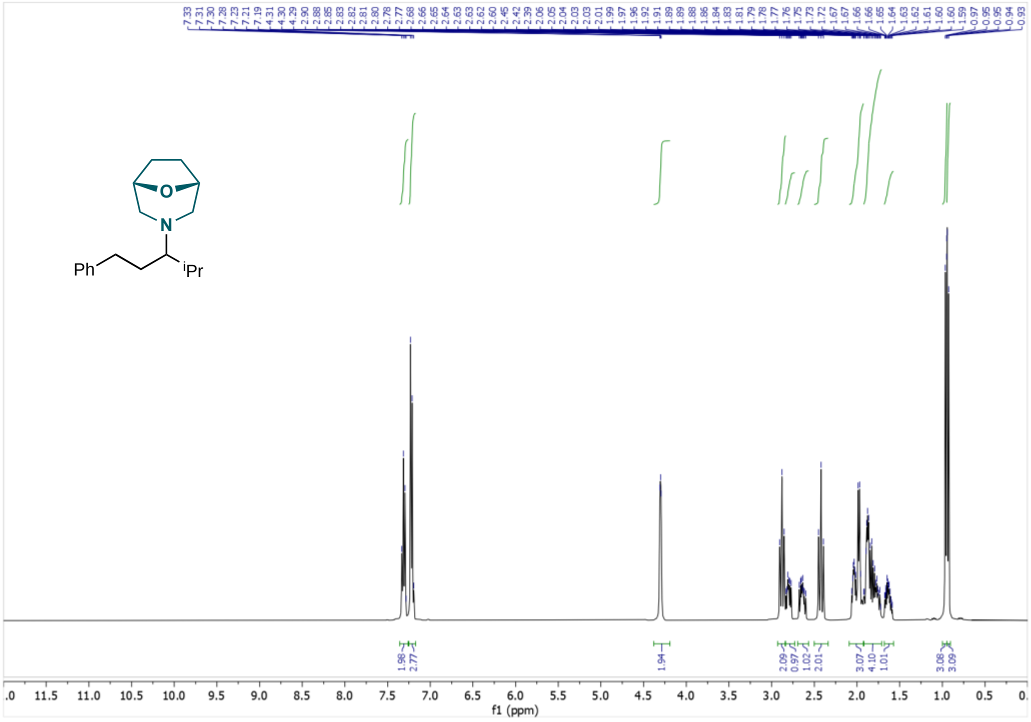

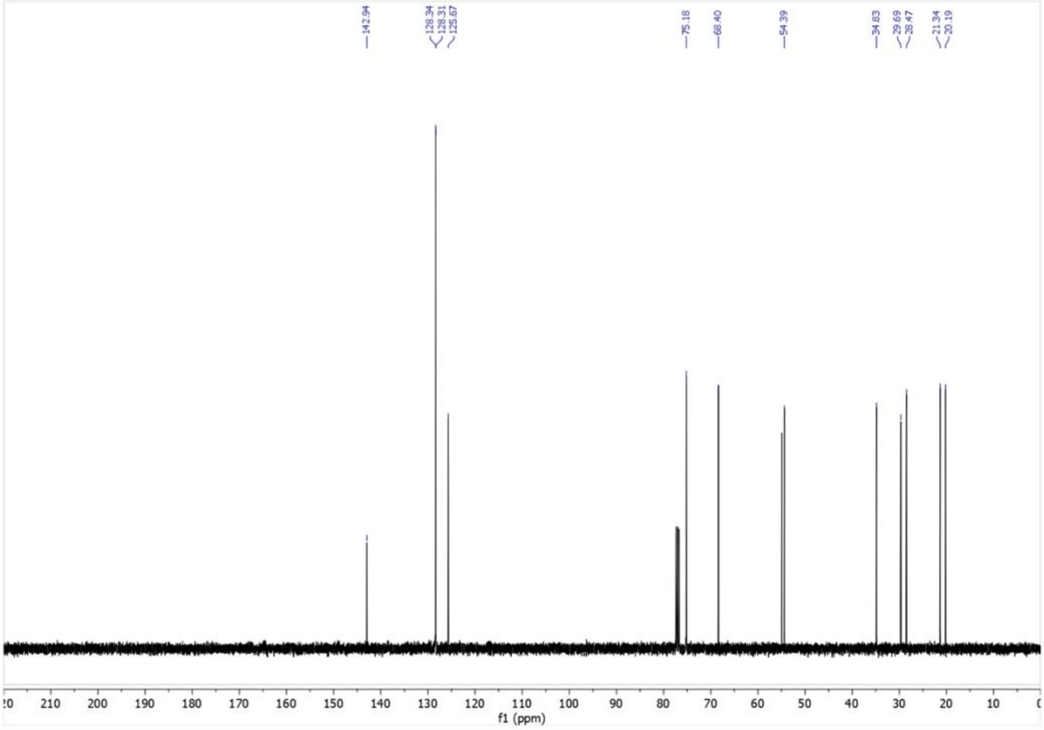

8-(4-Methyl-1-phenylpentan-3-yl)-1-phenyl-1,3,8-triazaspiro[4.5]decan-4-one (4w)

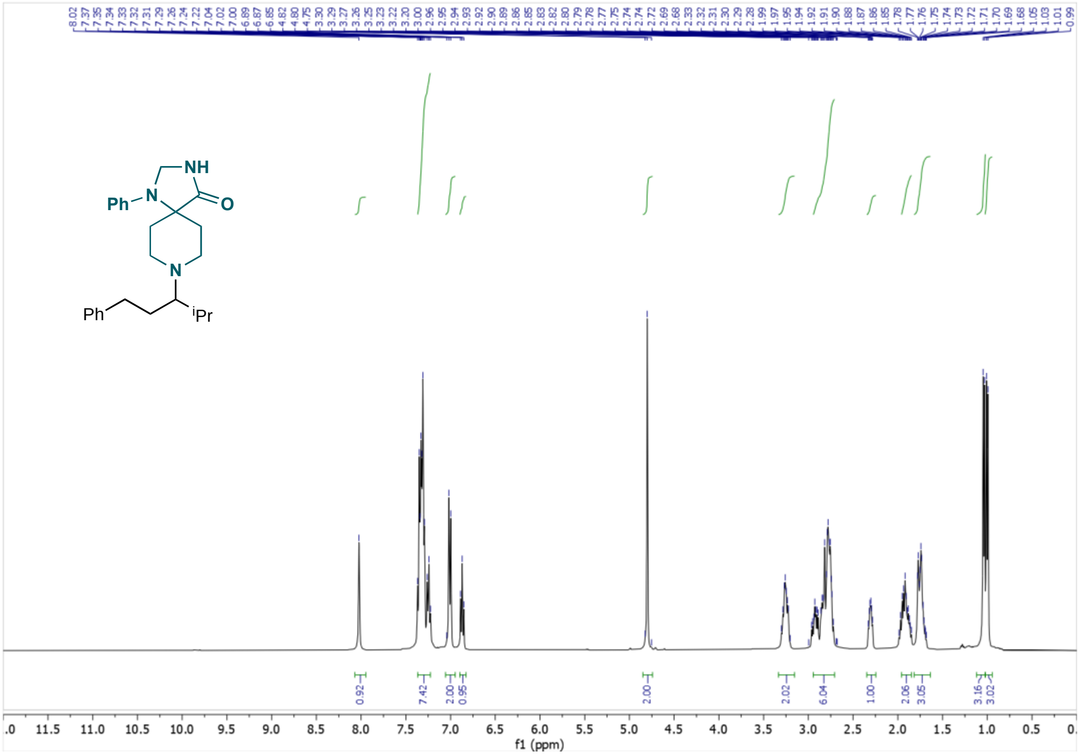

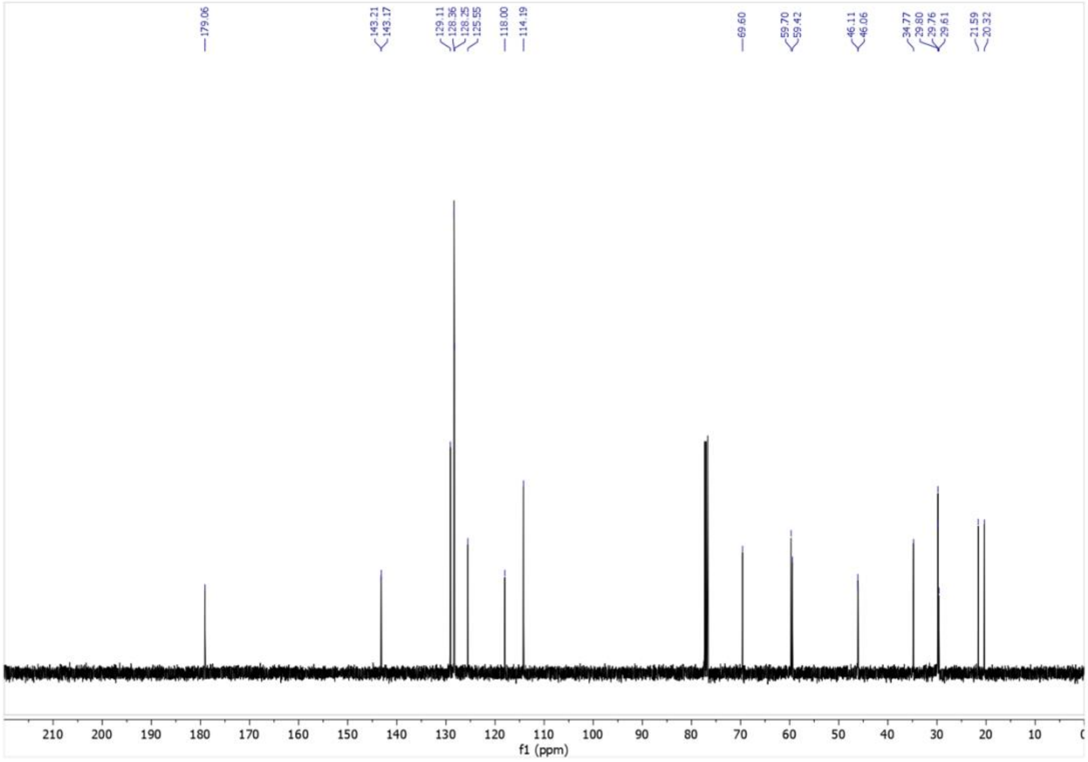

N,4-dimethyl-1-phenylpentan-3-amine (6a)

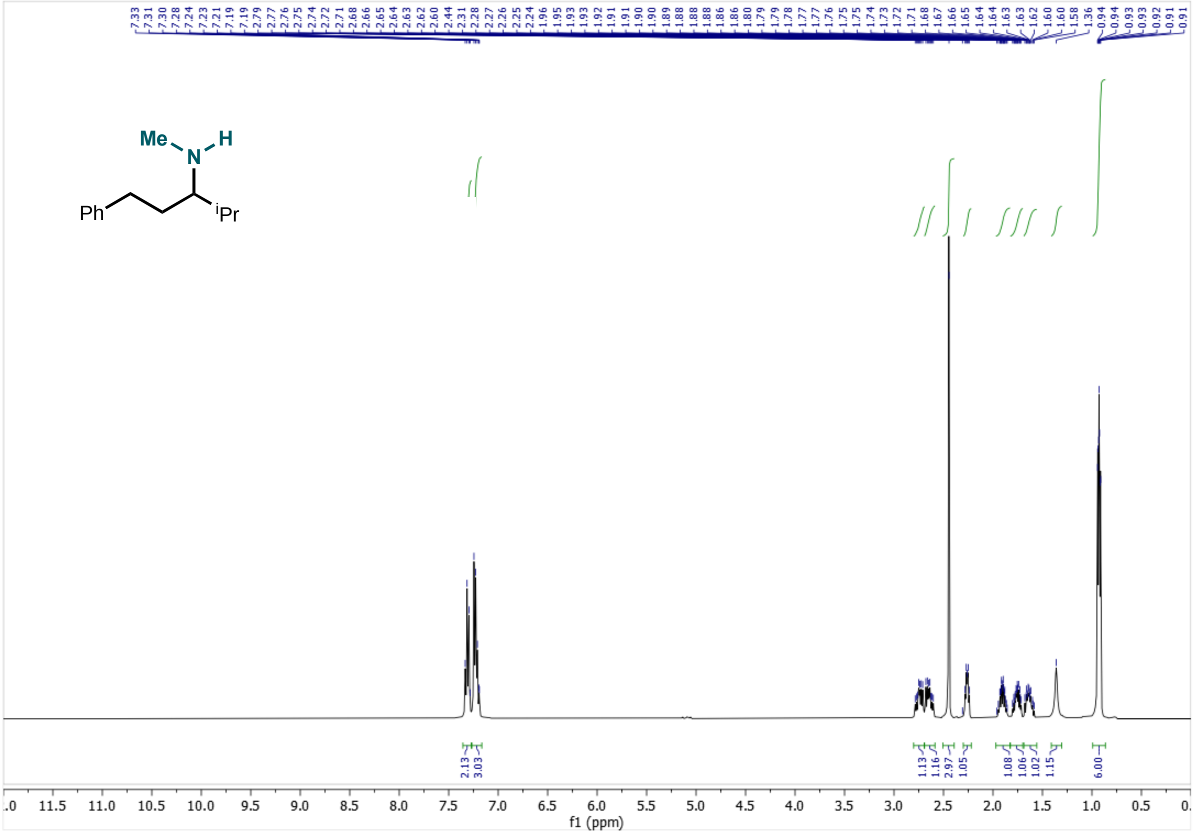

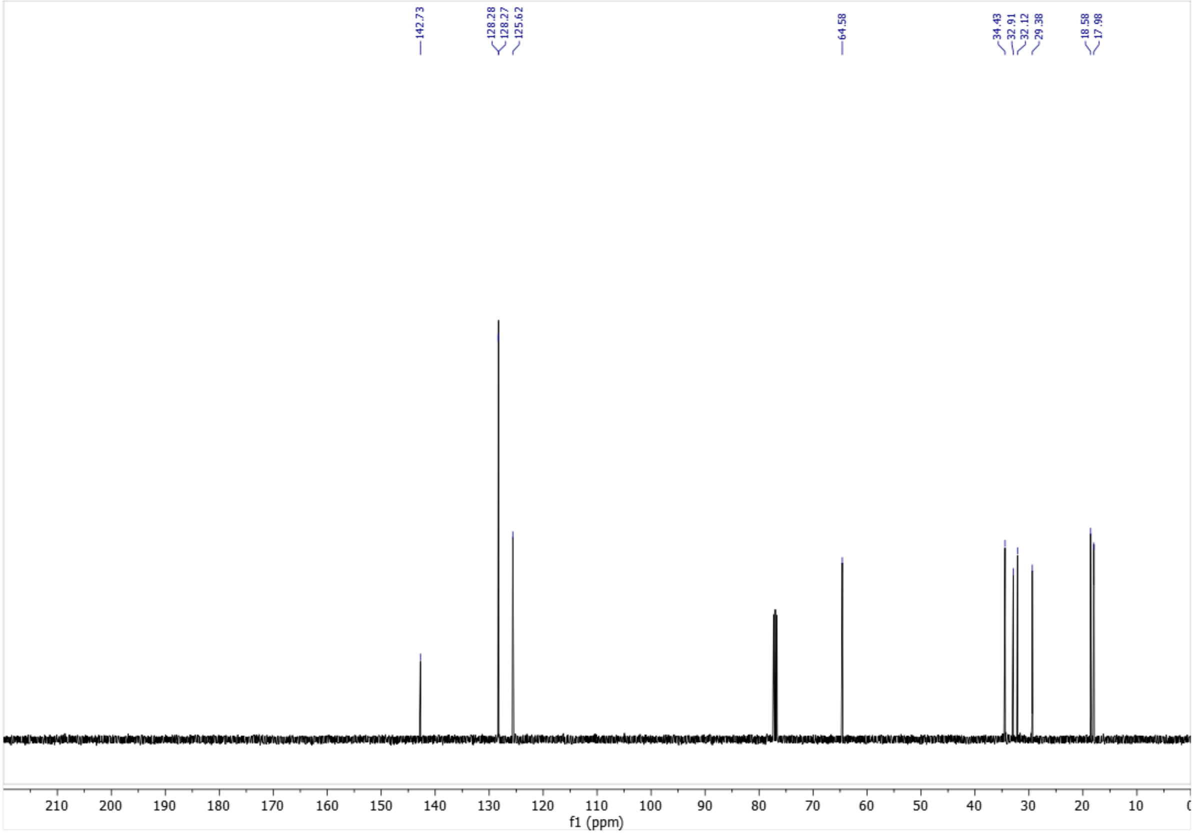

N-Benzyl-4-methyl-1-phenylpentan-3-amine (6b)

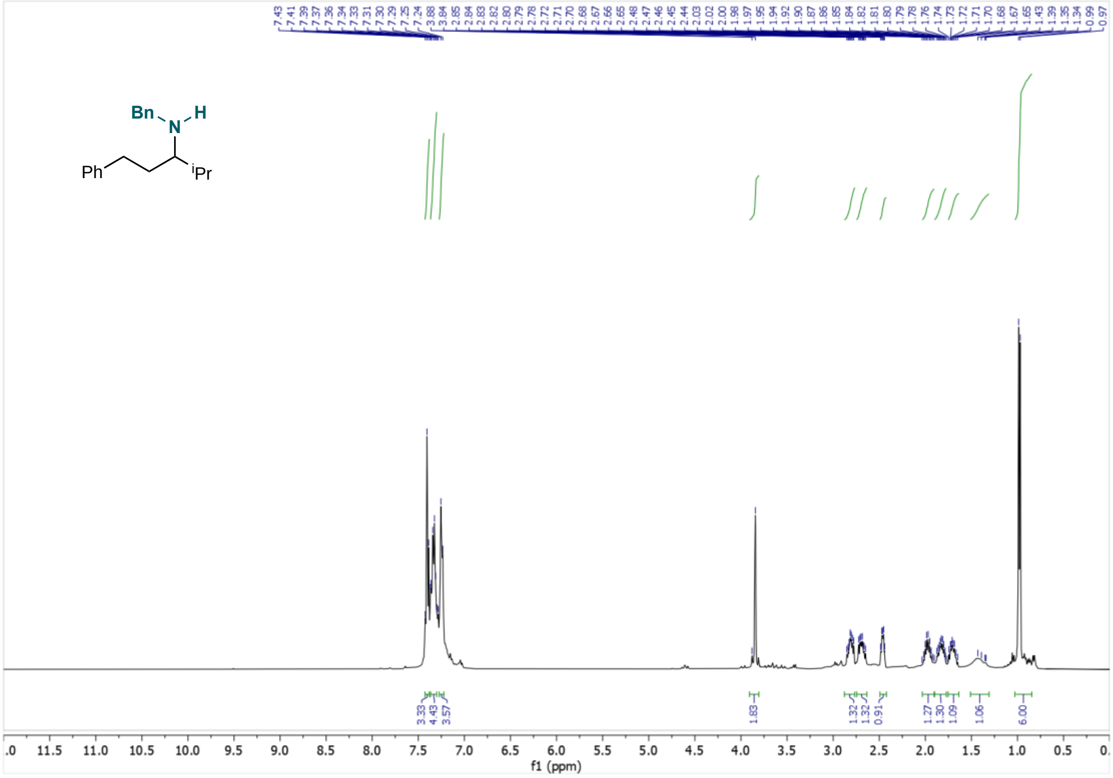

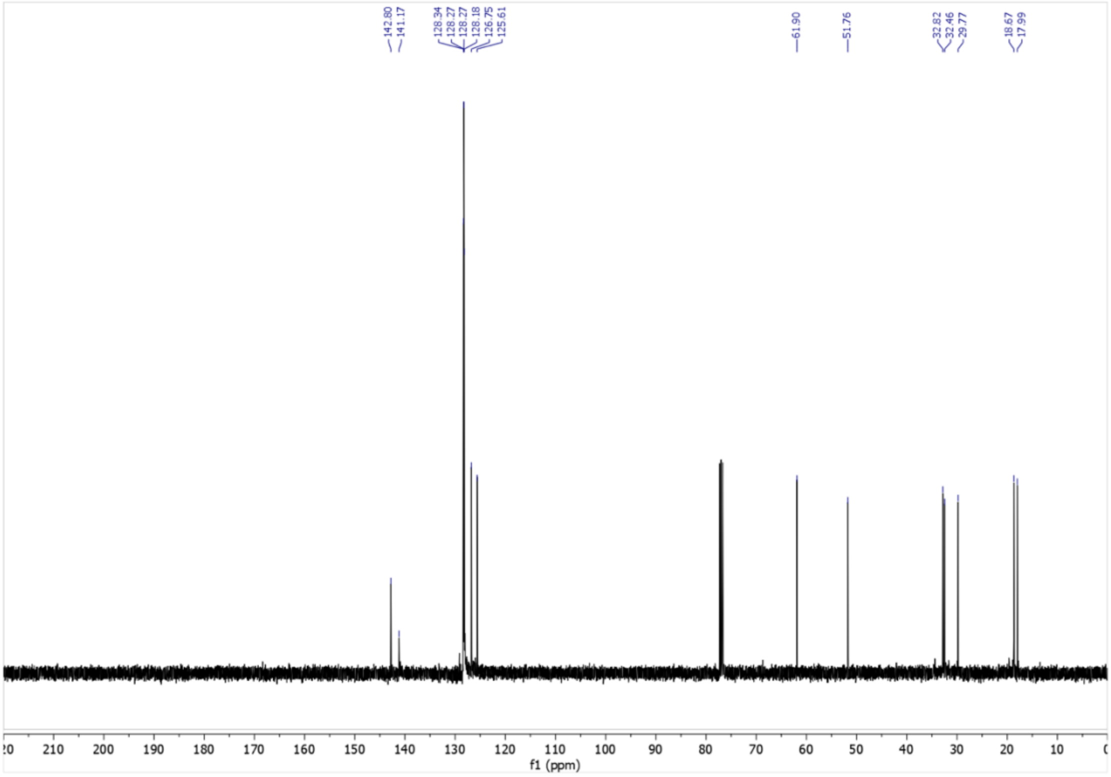

N-(but-3-en-1-yl)-4-methyl-1-phenylpentan-3-amine (6c)

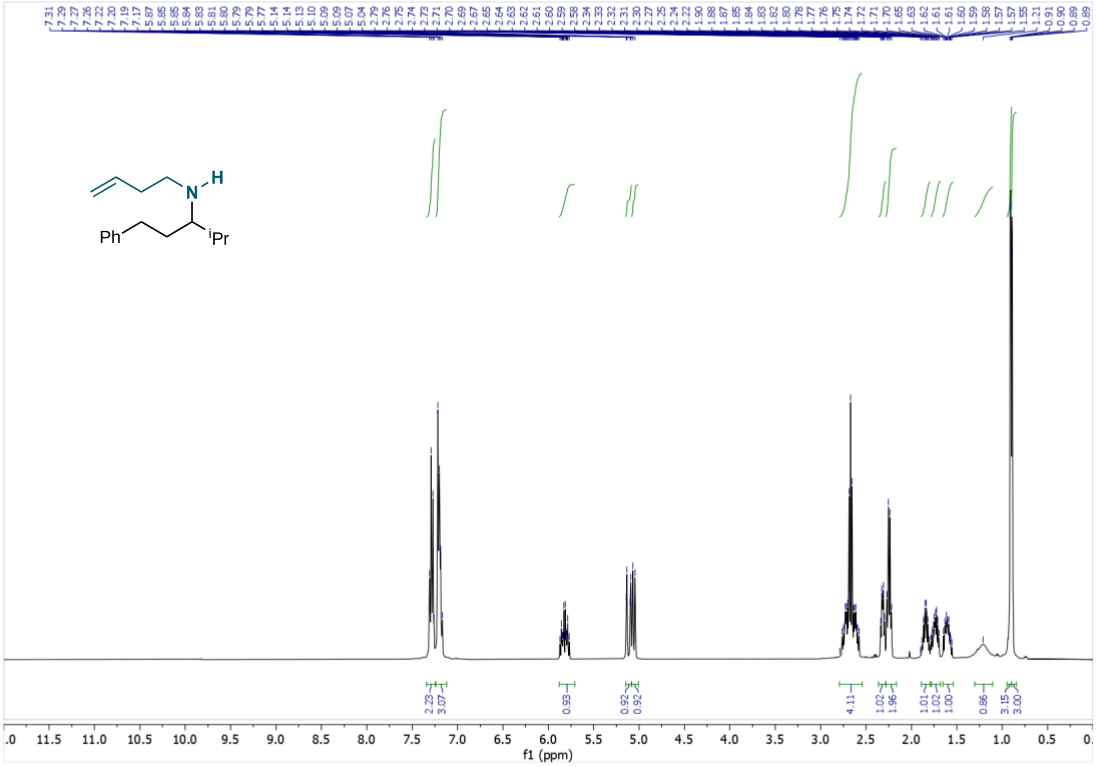

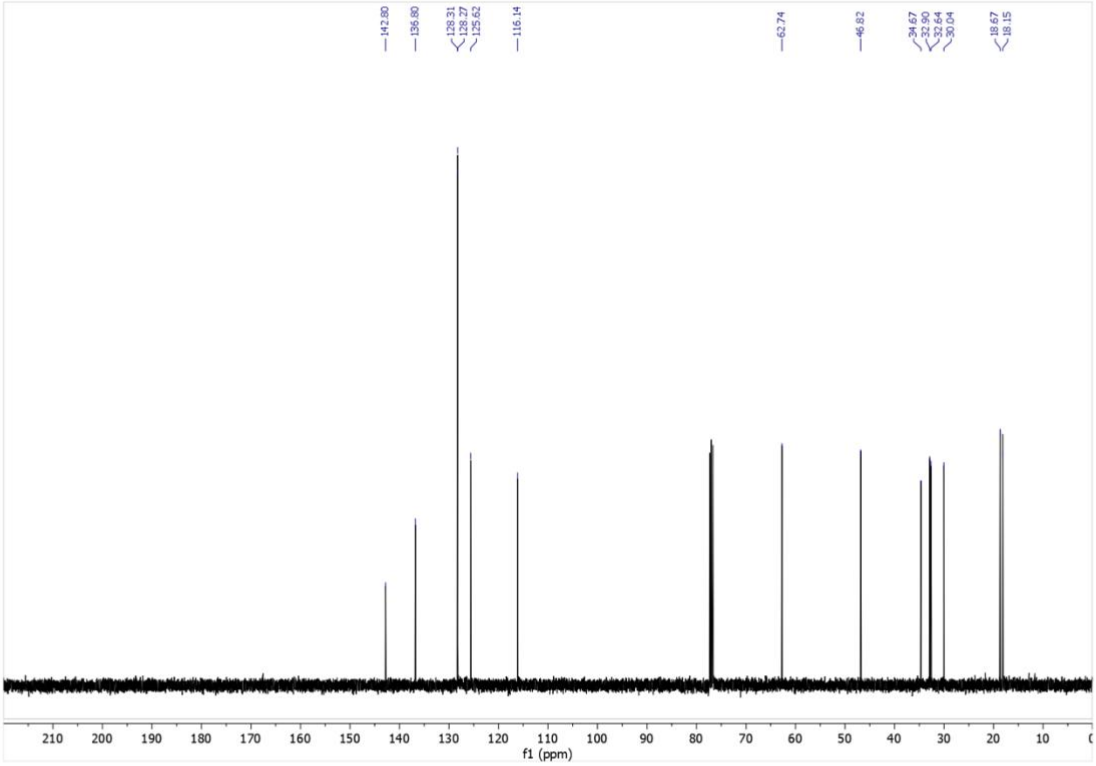

N-(2-Methoxyethyl)-4-methyl-1-phenylpentan-3-amine (6d)

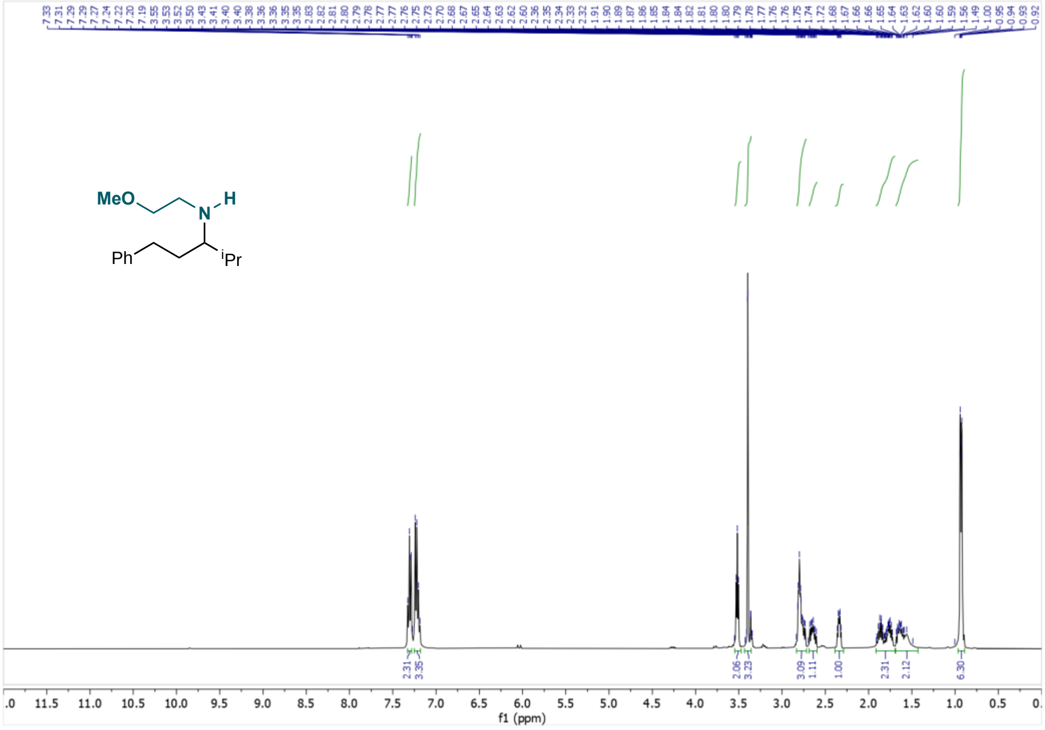

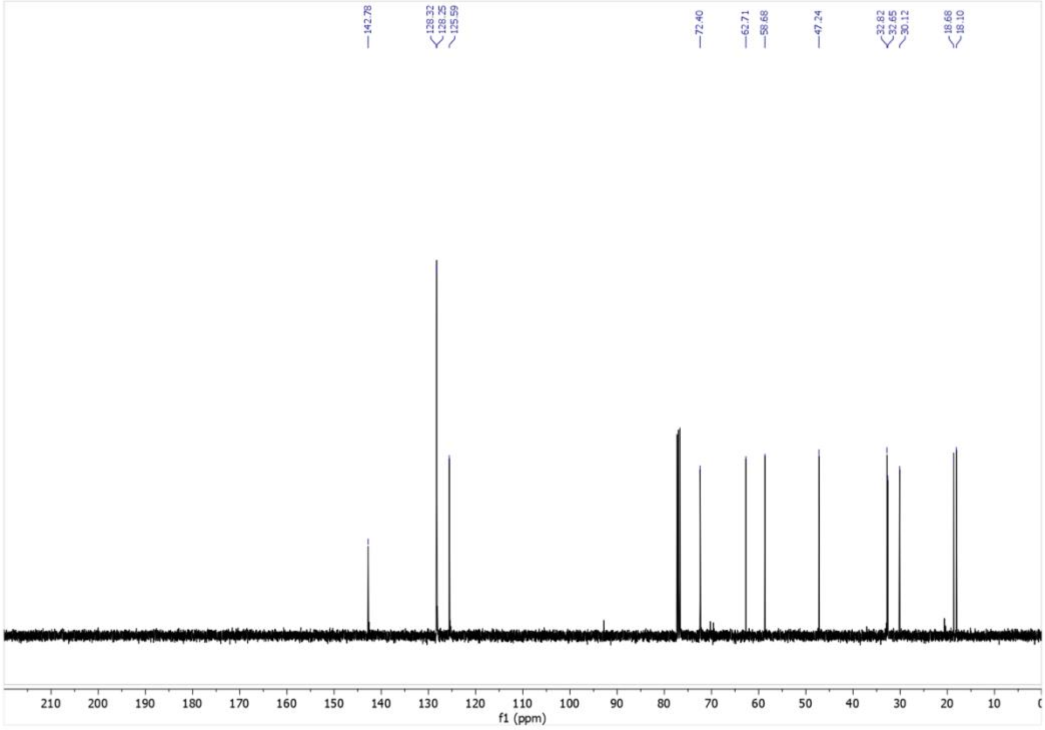

N-(2,2-Difluoroethyl)-4-methyl-1-phenylpentan-3-amine (6e)

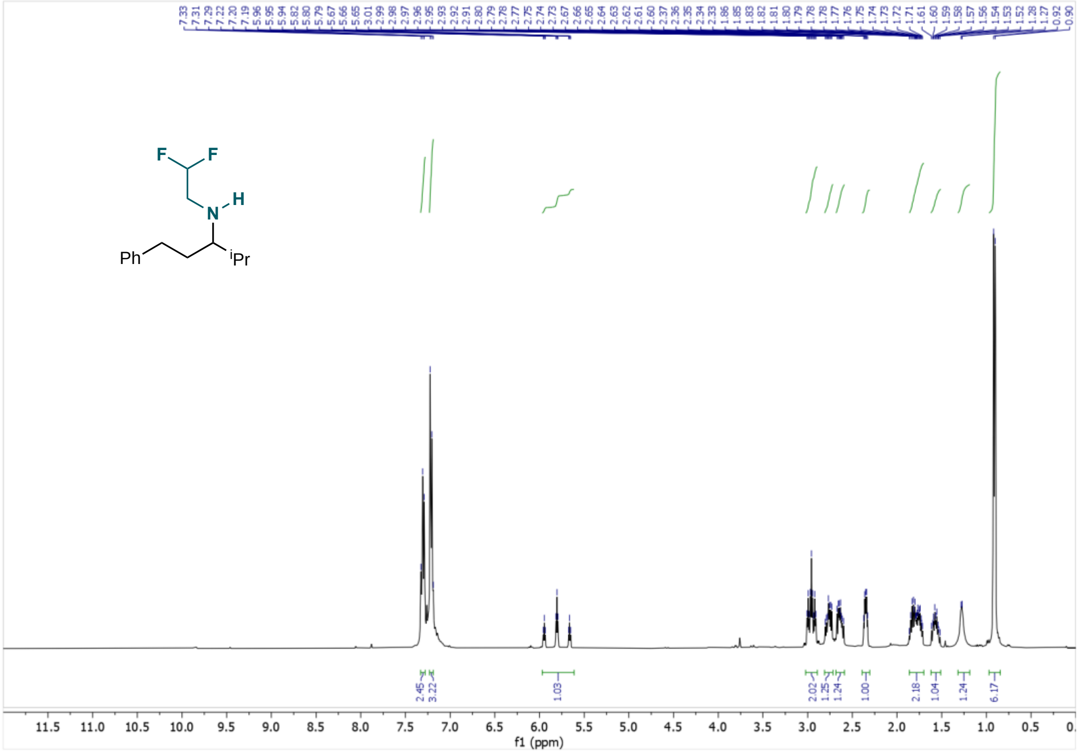

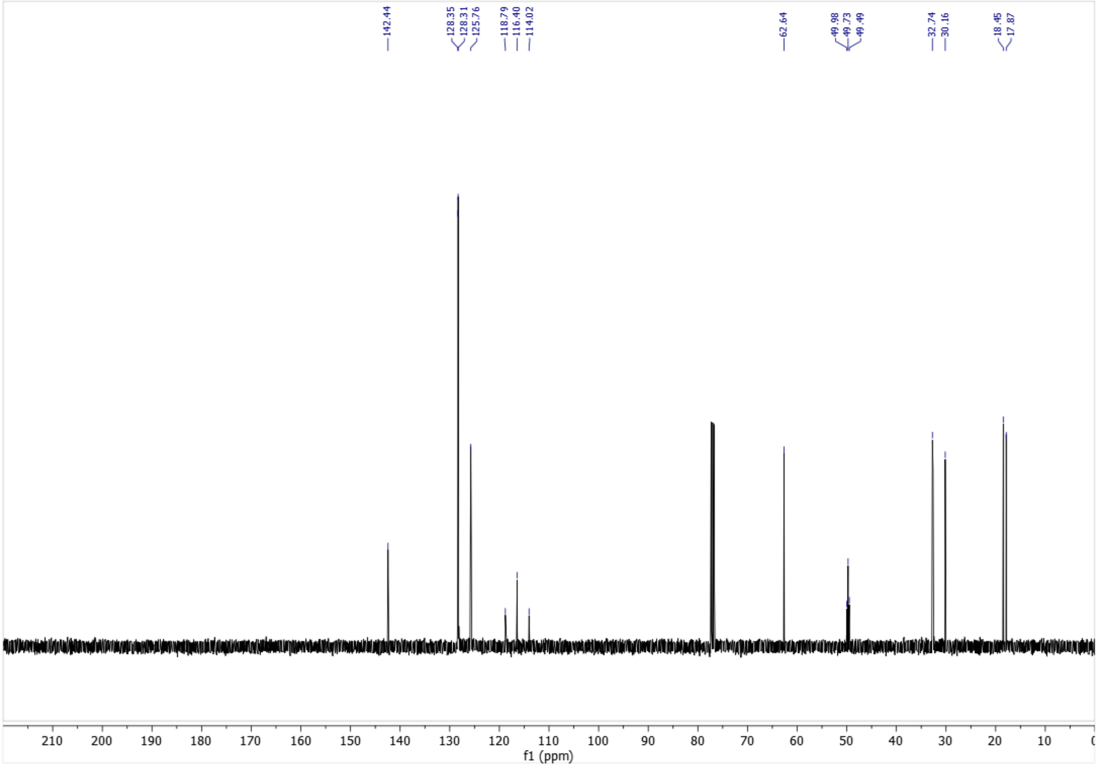

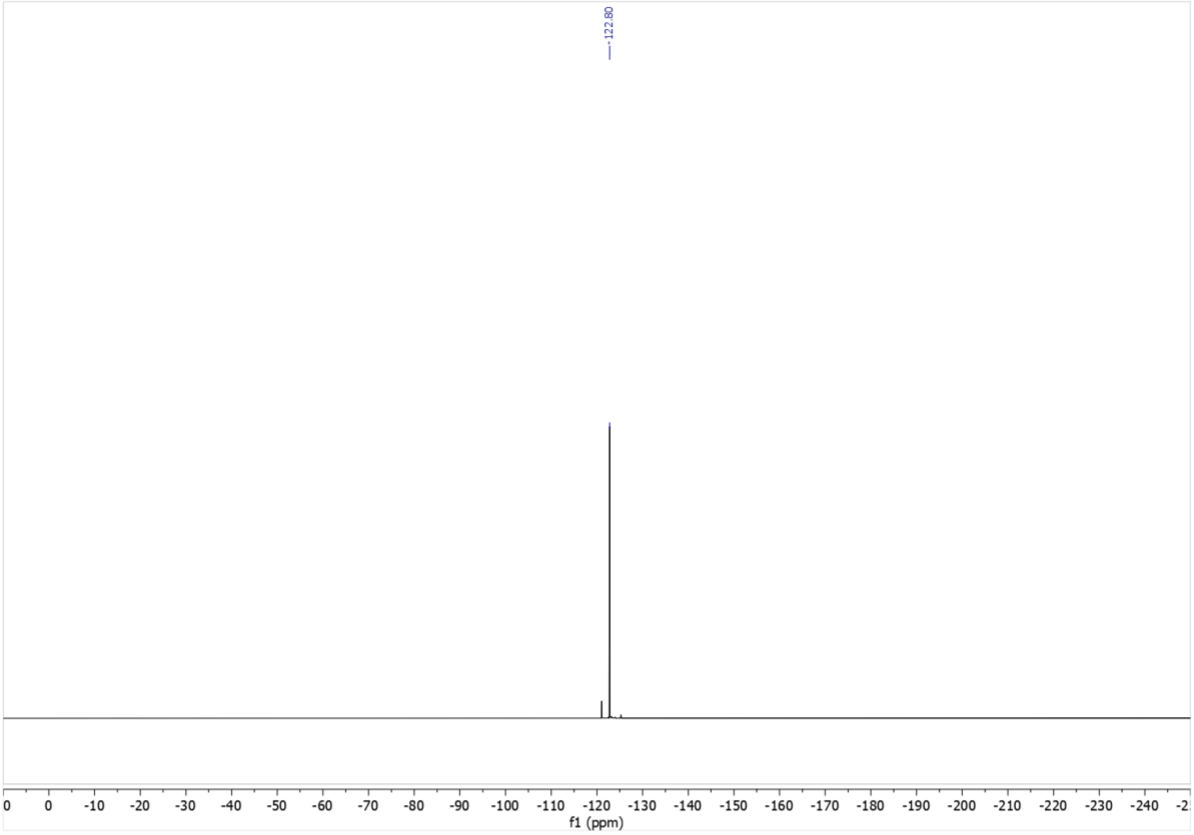

-122.80
0
0
-10
-20
-30
-40
-50
-60
-70
-80
-90
-100
-110
-120
-130
-140
-150
-160
-170
-180
-190
-200
-210
-220
-230
-240
f1 (ppm)

4-Methyl-1-phenyl-N-(2-(pyridin-4-yl)ethyl)pentan-3-amine (6f)

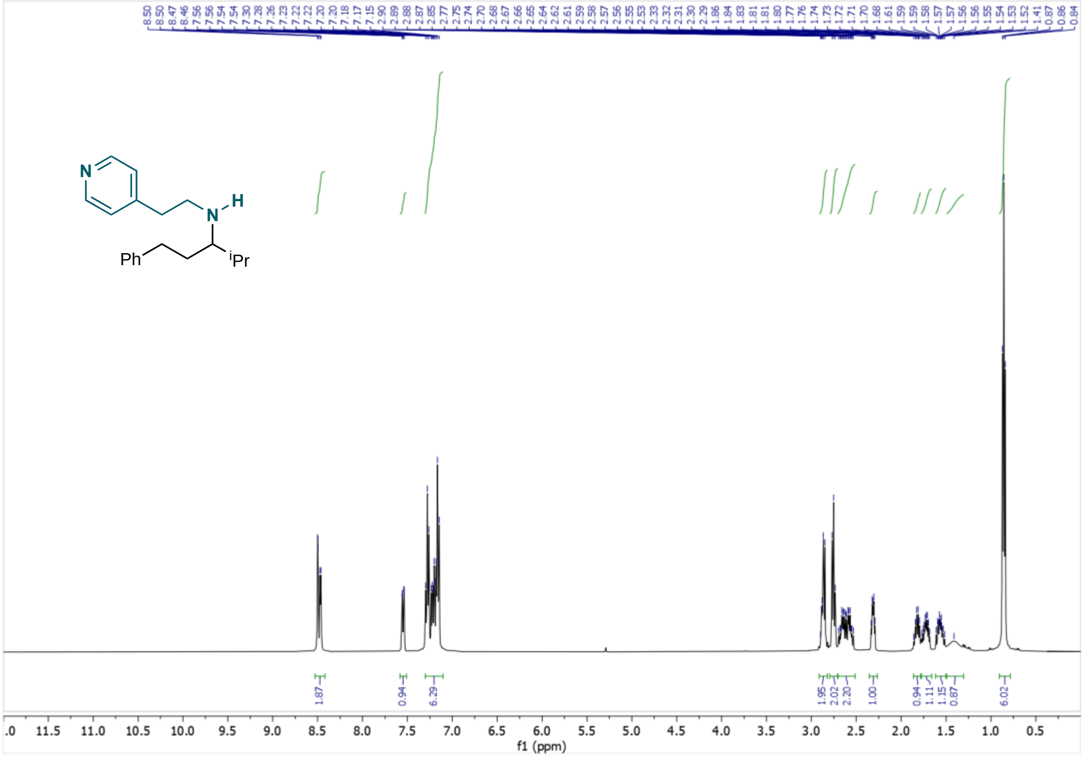

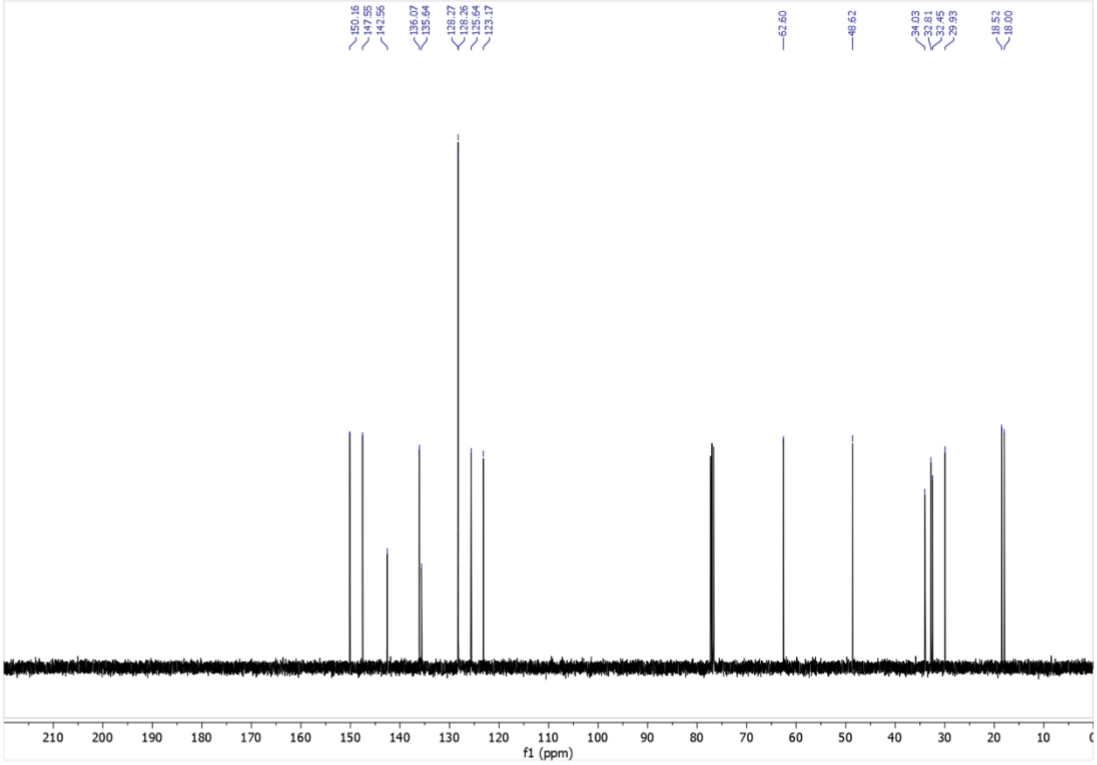

N-(4-Methyl-1-phenylpentan-3-yl)tetrahydro-2H-pyran-4-amine (6g)

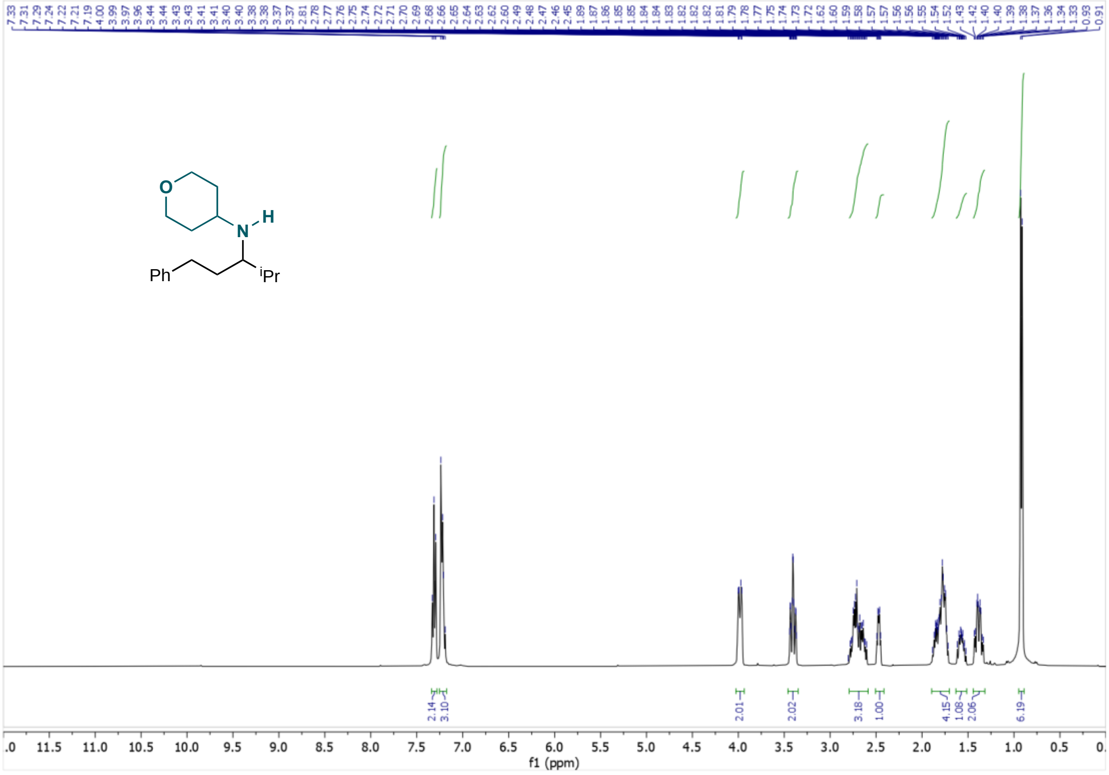

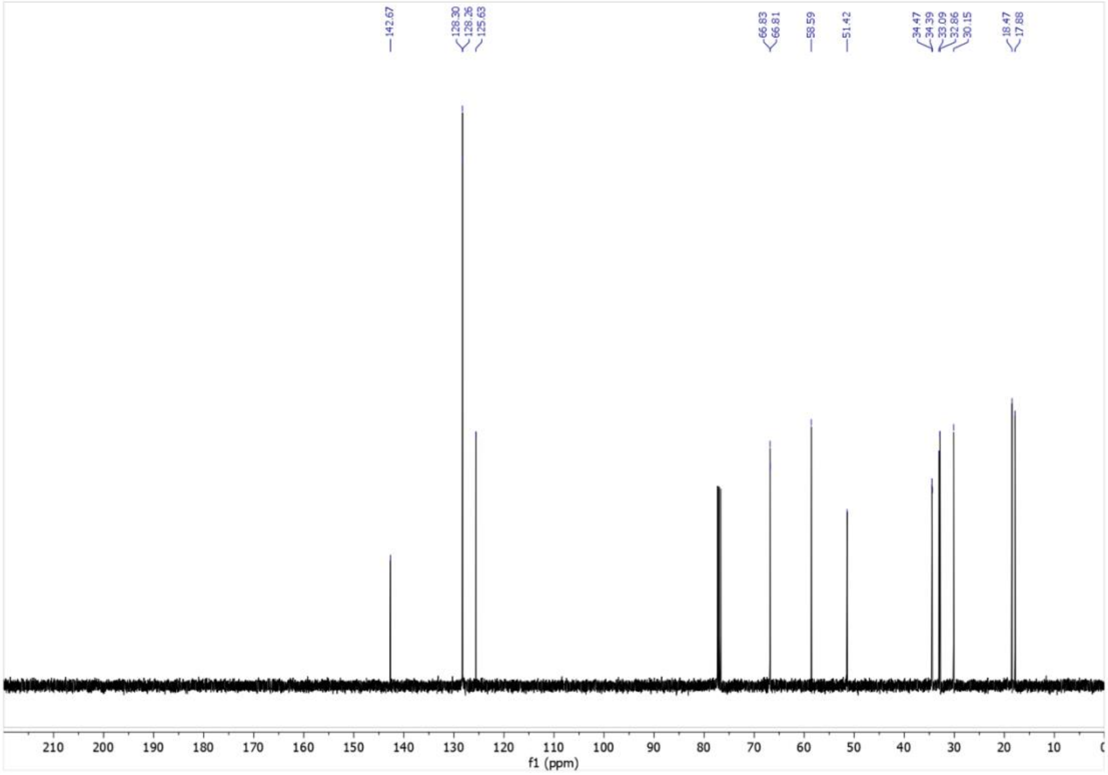

Benzyl 4-((4-methyl-1-phenylpentan-3-yl)amino)piperidine-1-carboxylate (6h)

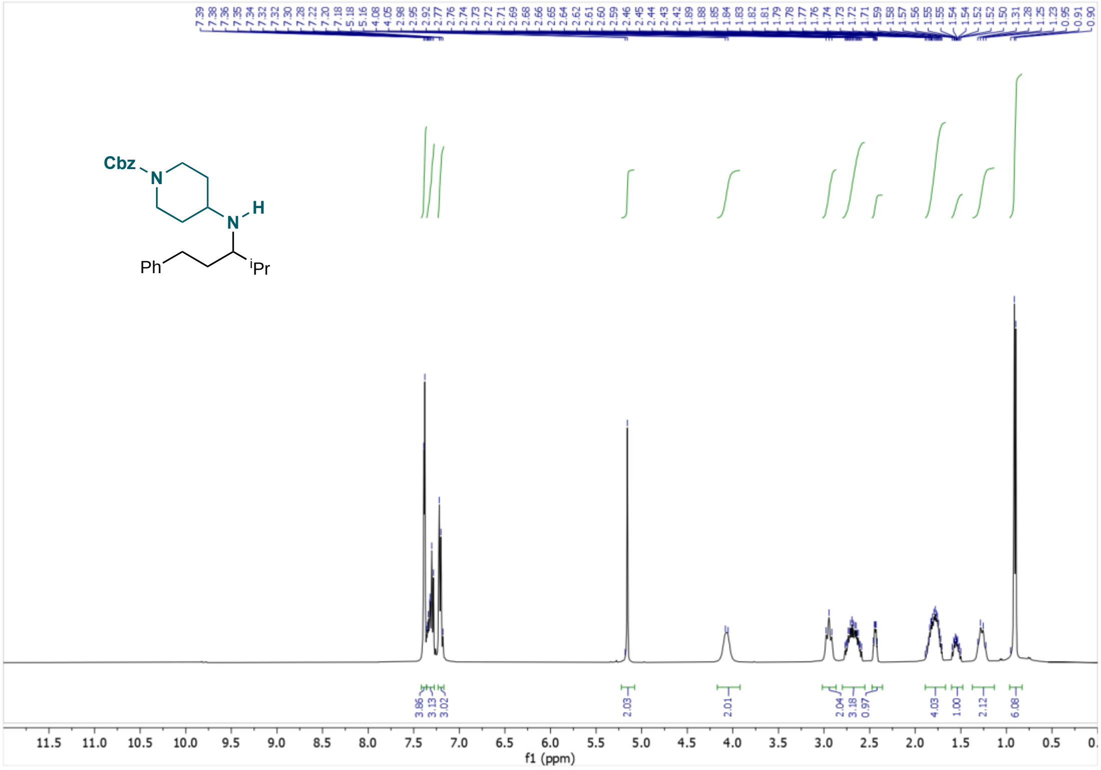

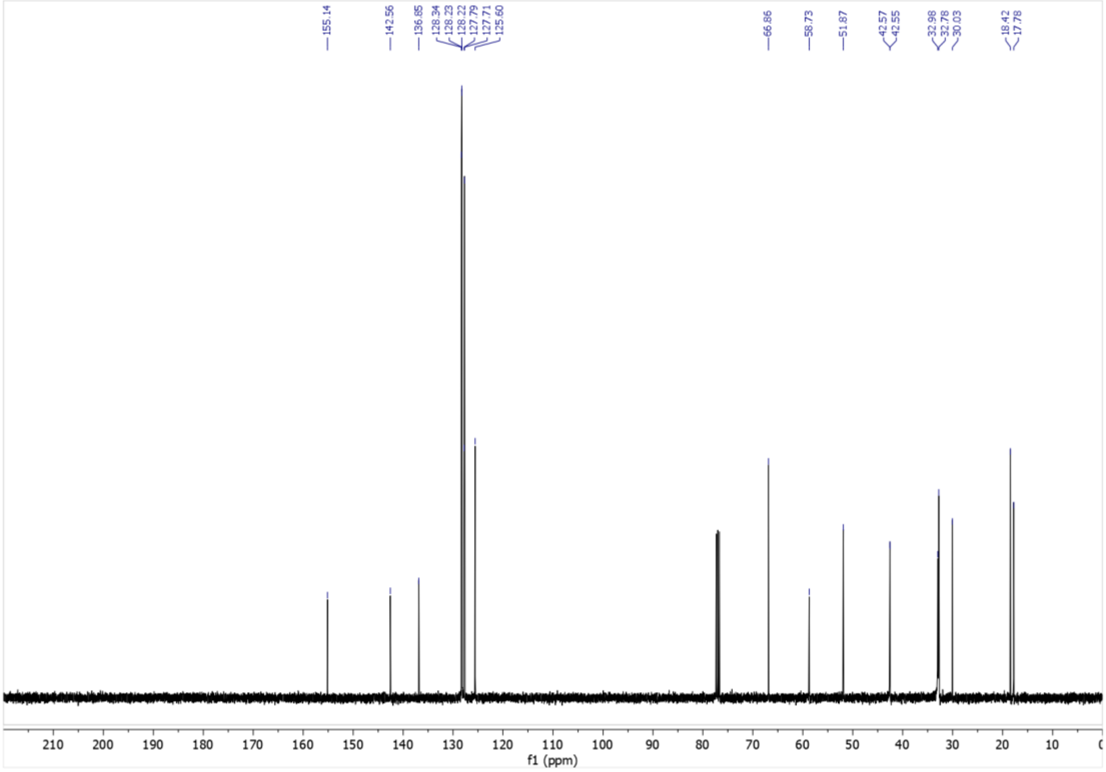

1-Ethynyl-N-(4-methyl-1-phenylpentan-3-yl)cyclohexan-1-amine (6i)

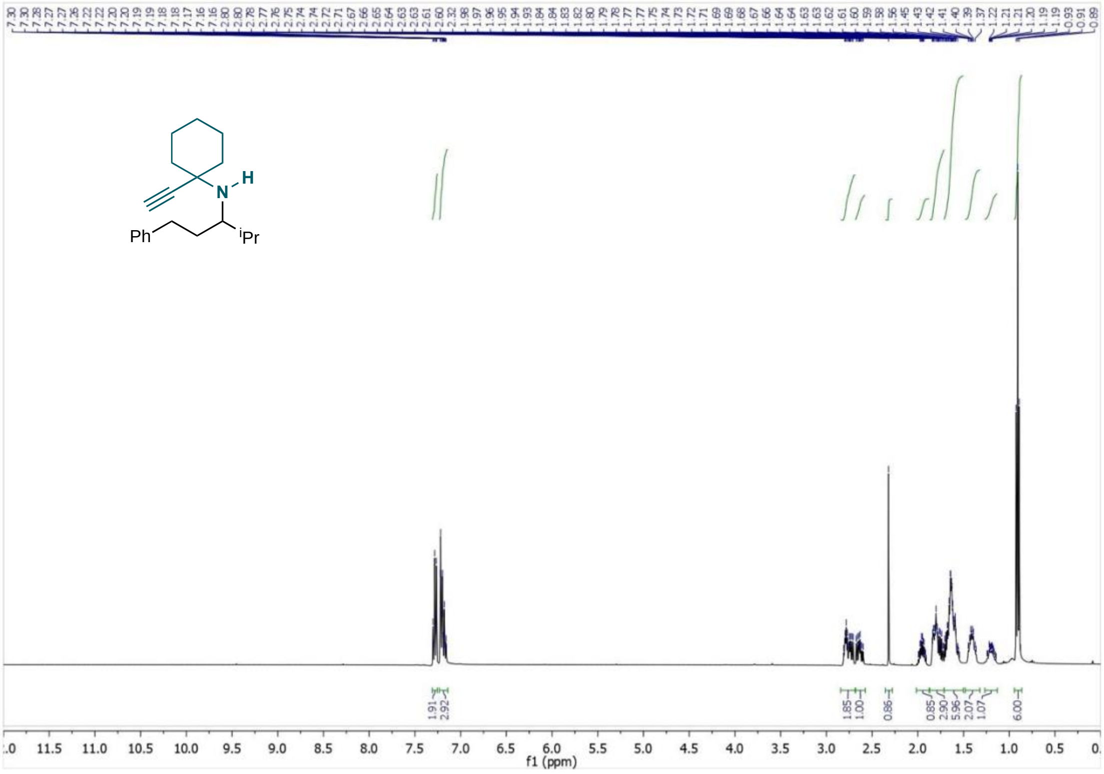

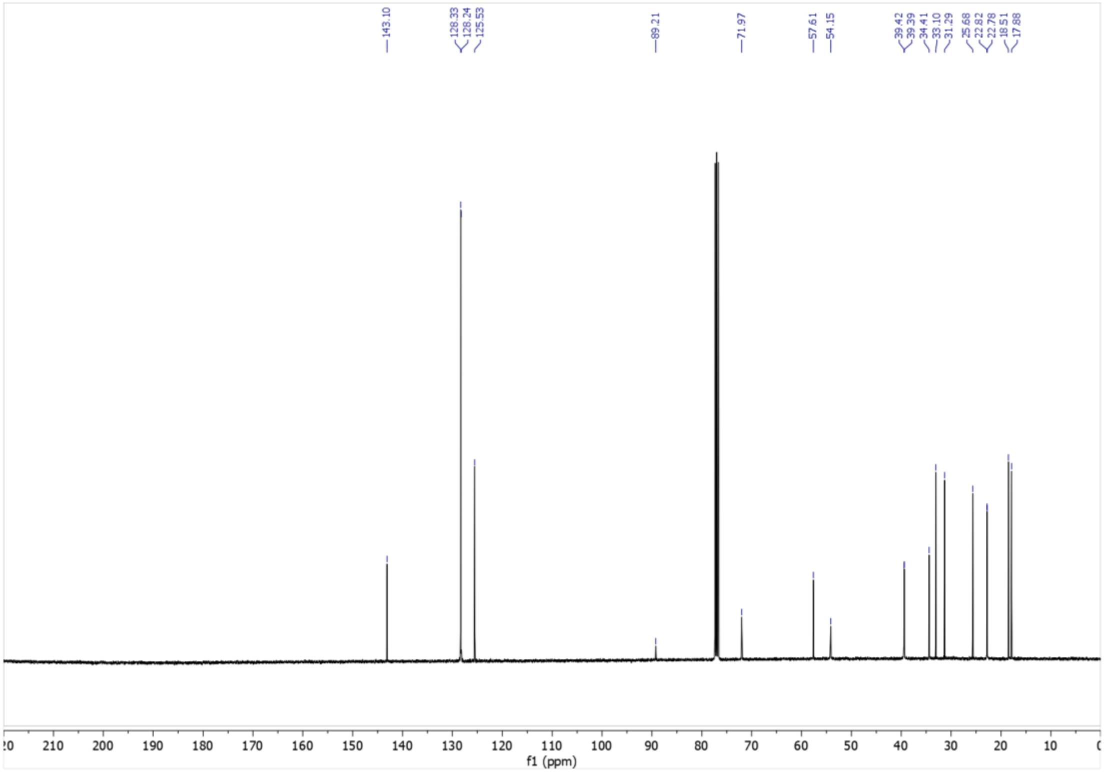

N-(4-Methyl-1-phenylpentan-3-yl)adamantan-1-amine (6j)

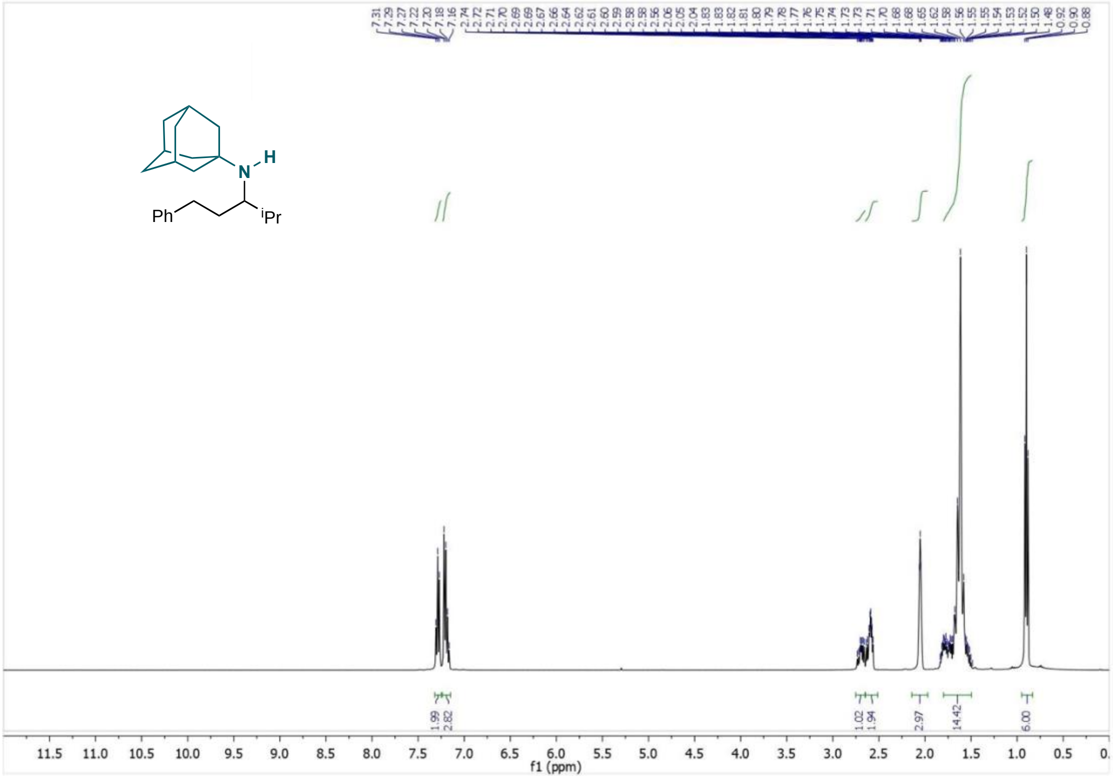

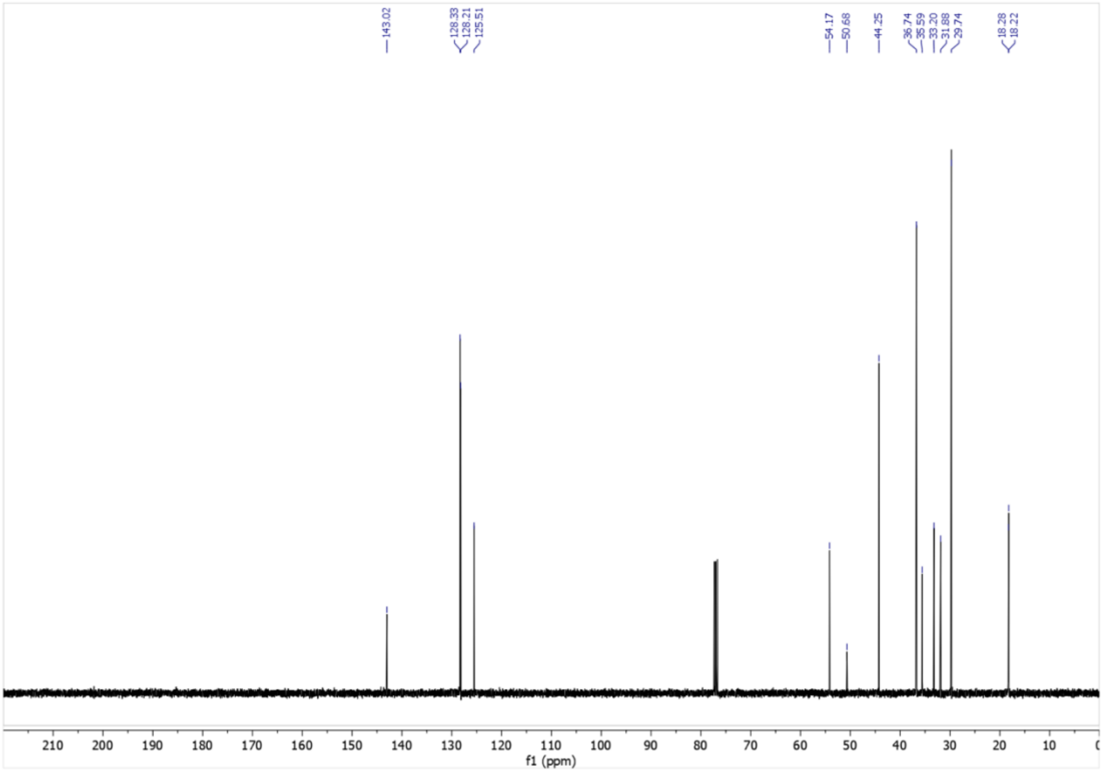

N-(4-Methyl-1-phenylpentan-3-yl)aniline (7a)

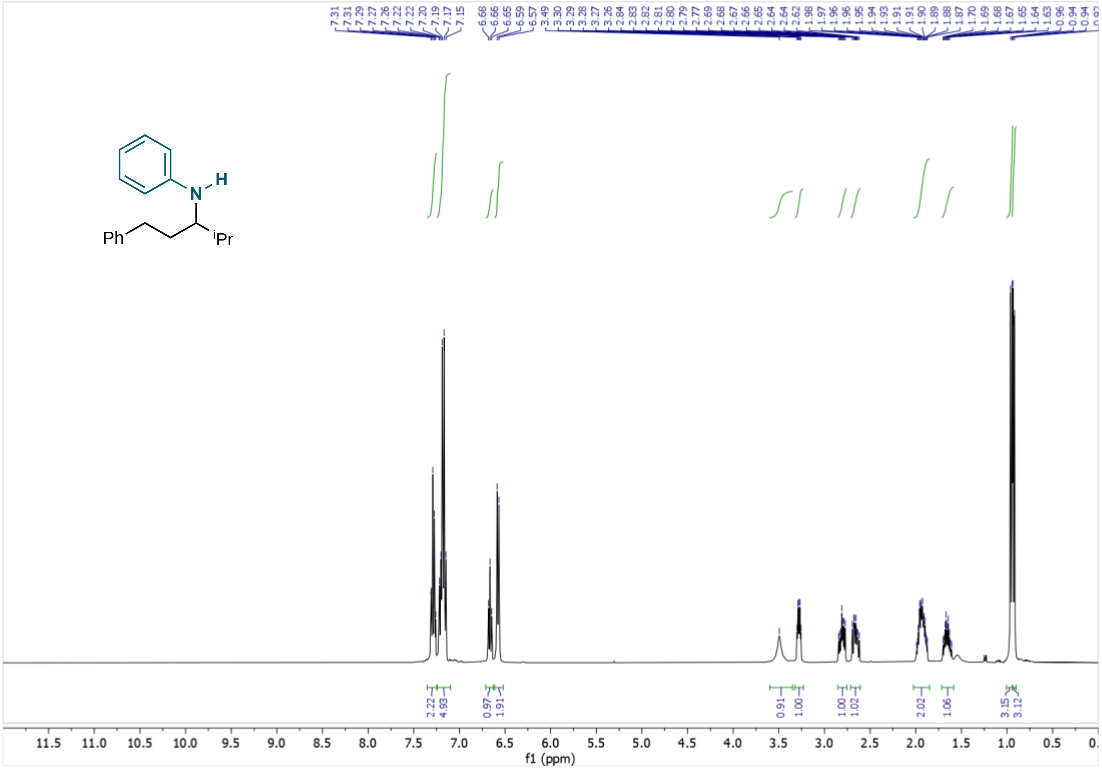

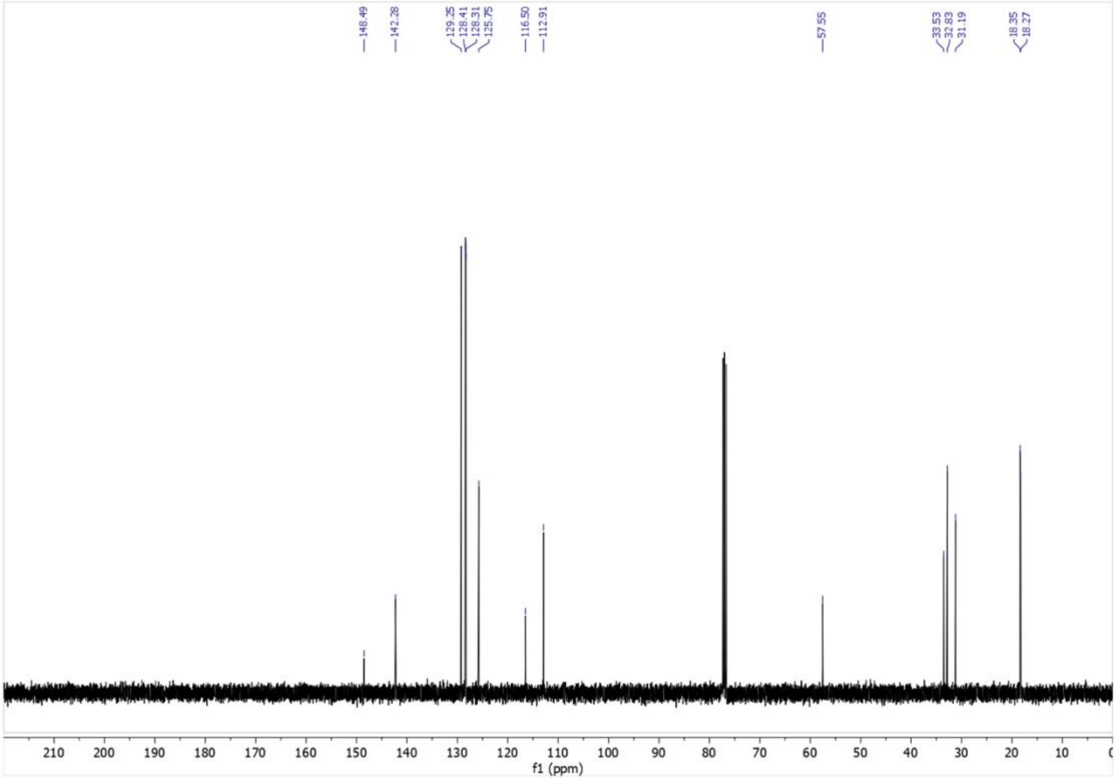

4-Iodo-N-(4-methyl-1-phenylpentan-3-yl)aniline (7b)

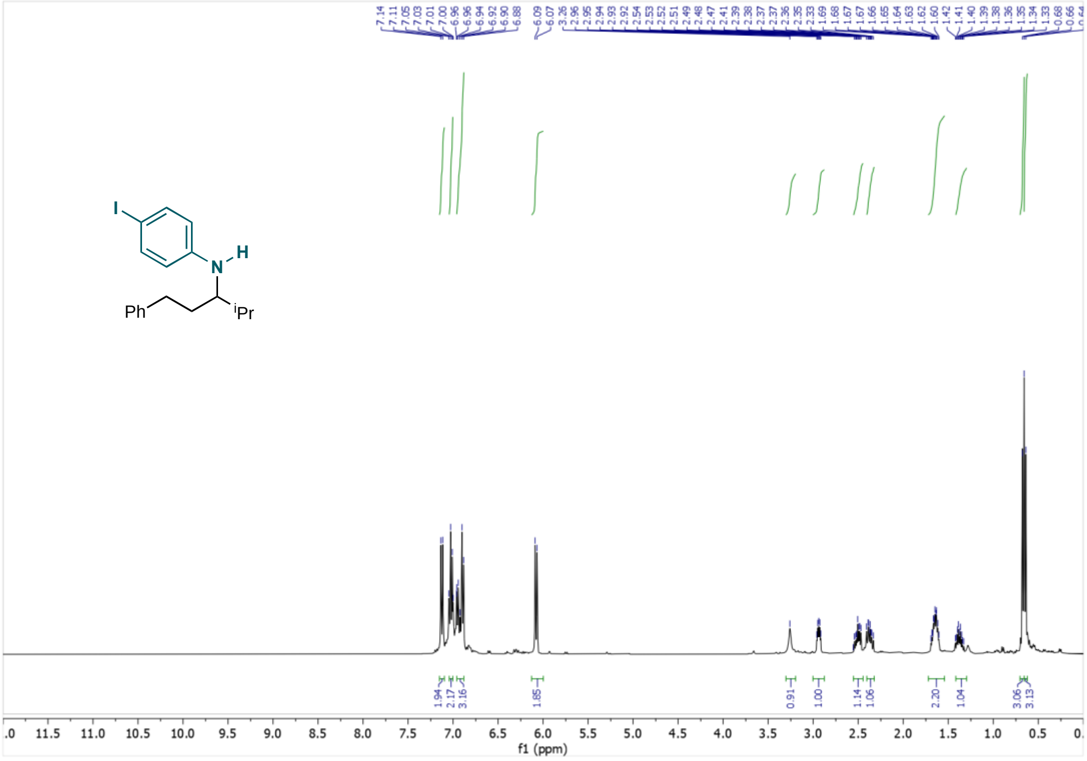

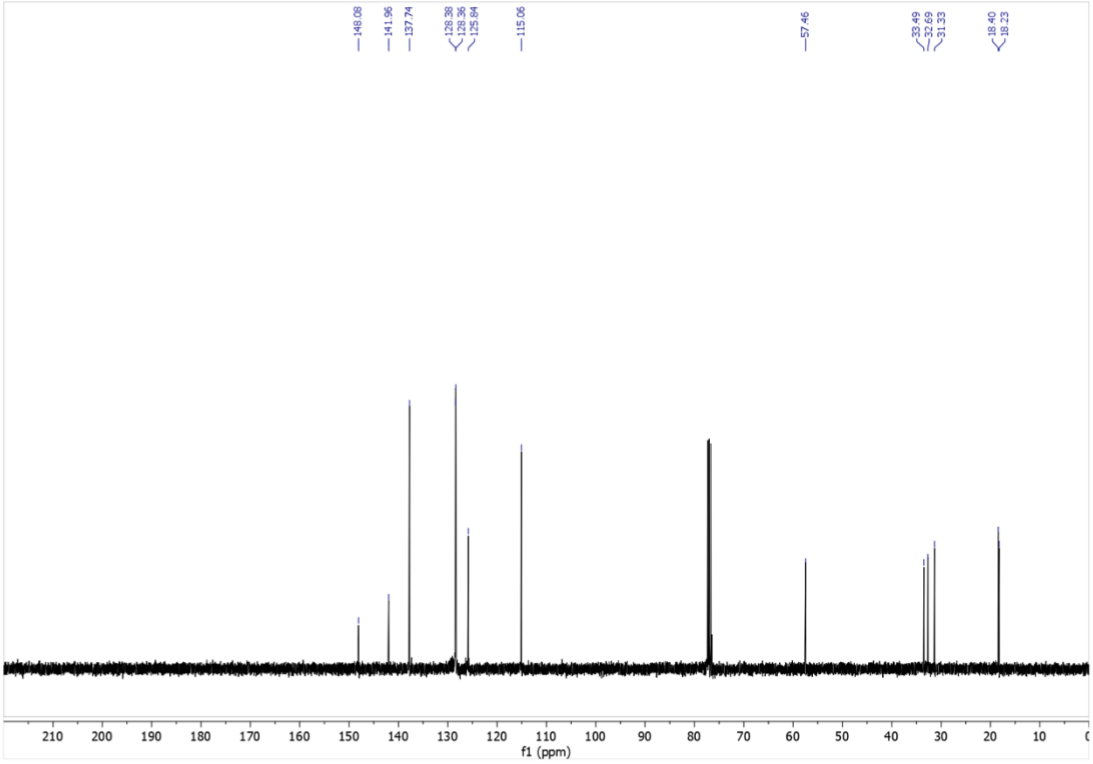

4-Methoxy-N-(4-methyl-1-phenylpentan-3-yl)aniline (7c)

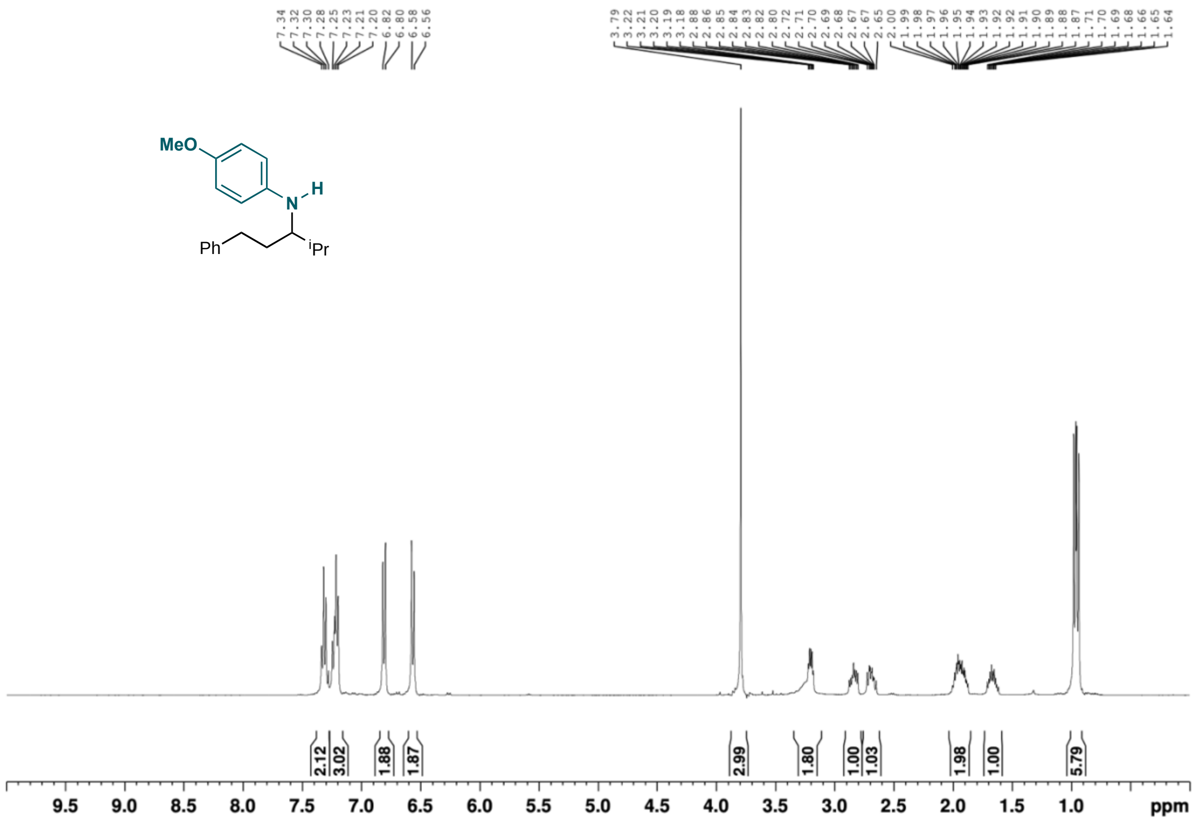

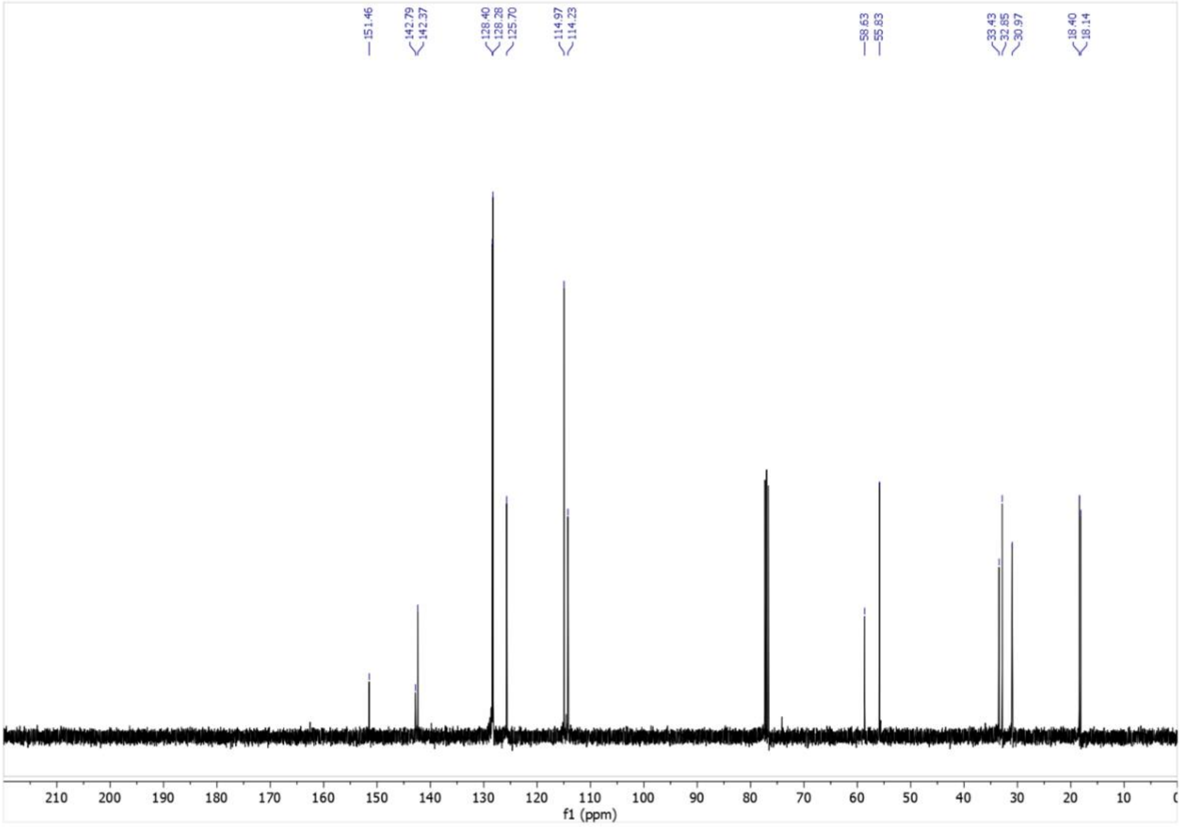

2,4,6-trimethyl-N-(4-methyl-1-phenylpentan-3-yl)aniline (7d)

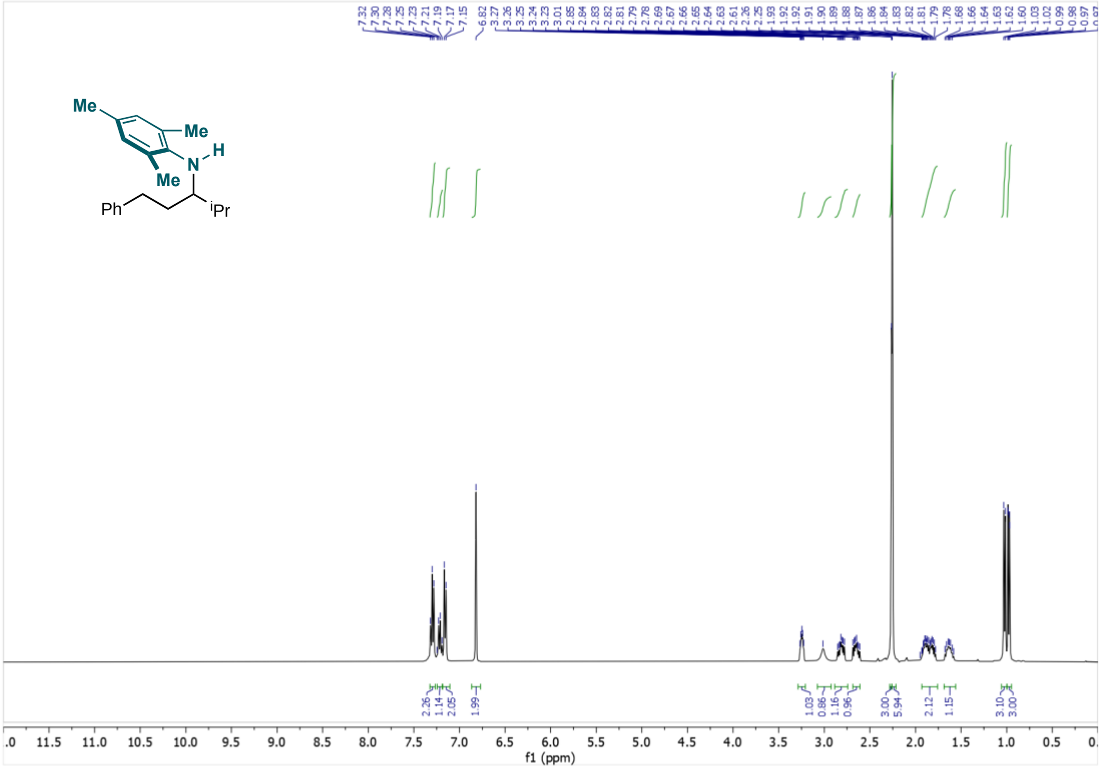

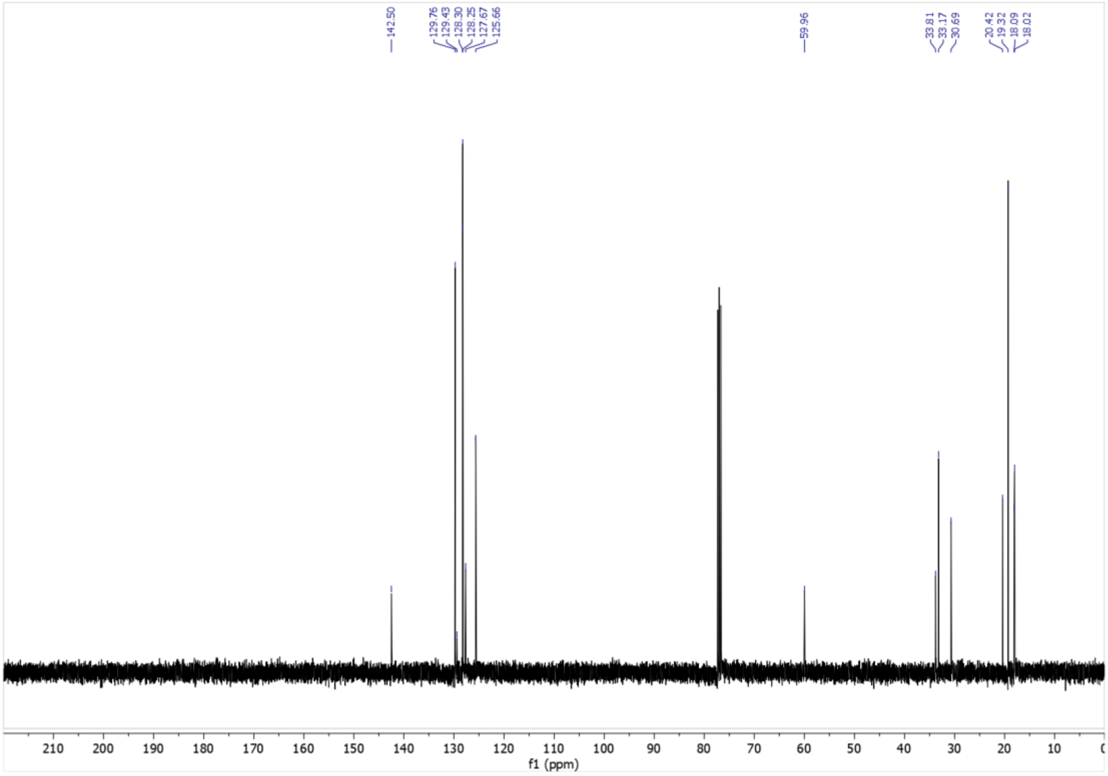

(1S,4S)-4-(3,4-Dichlorophenyl)-N-methyl-N-(4-methyl-1-phenylpentan-3-yl)-1,2,3,4 tetrahydronaphthalen-1-amine (8a) major isomer

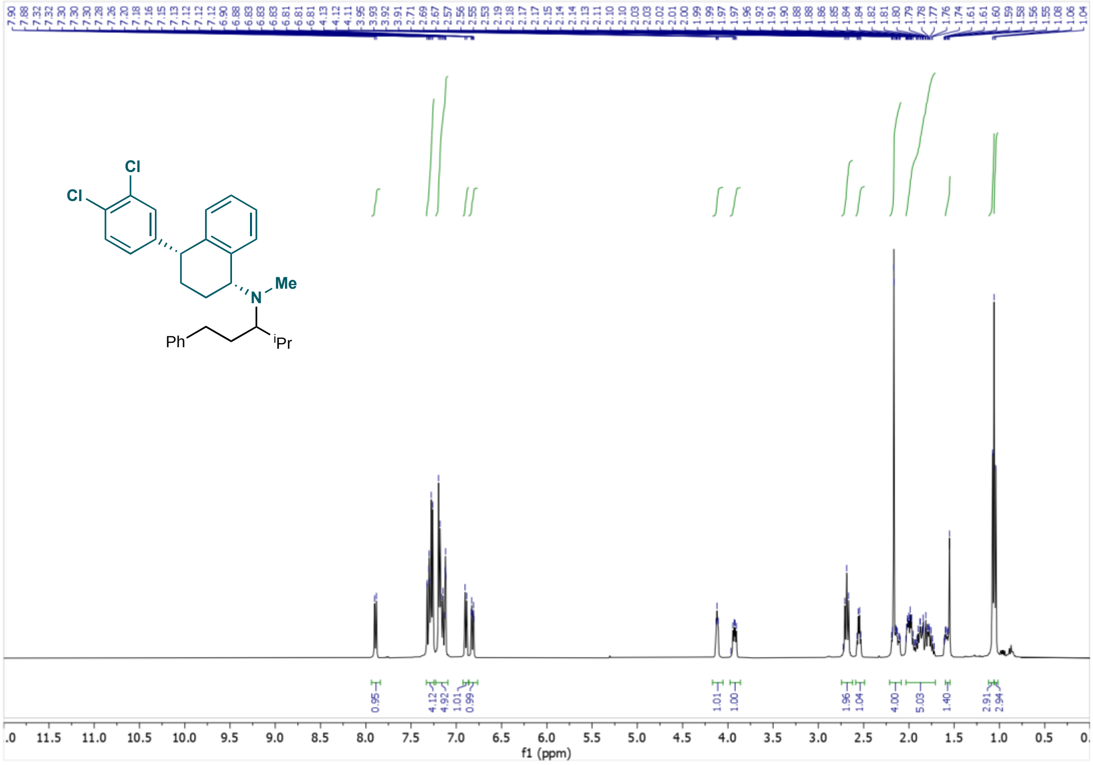

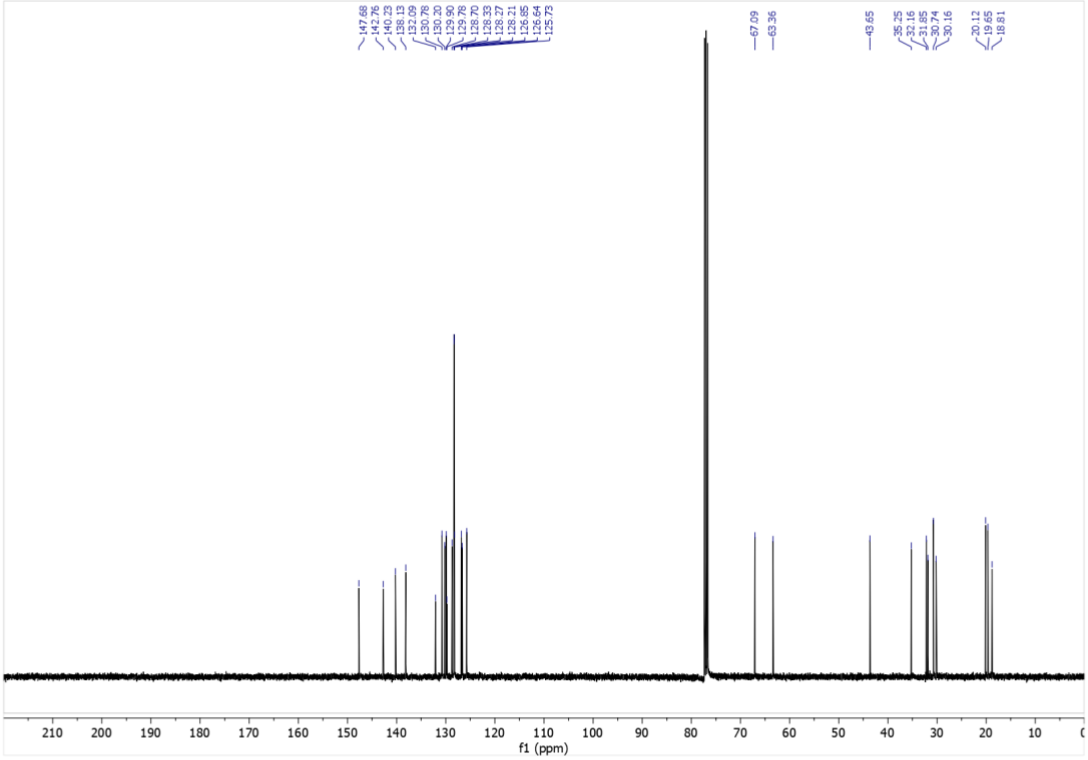

(1S,4S)-4-(3,4-Dichlorophenyl)-N-methyl-N-(4-methyl-1-phenylpentan-3-yl)-1,2,3,4 tetrahydronaphthalen-1-amine (8a) minor isomer

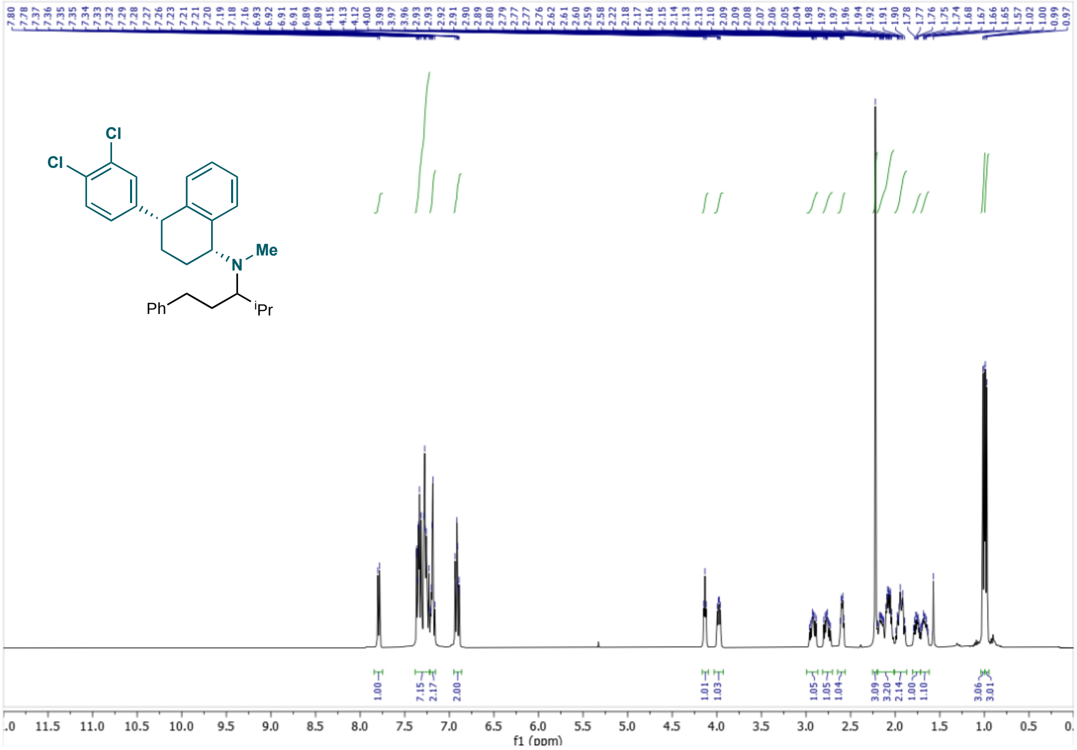

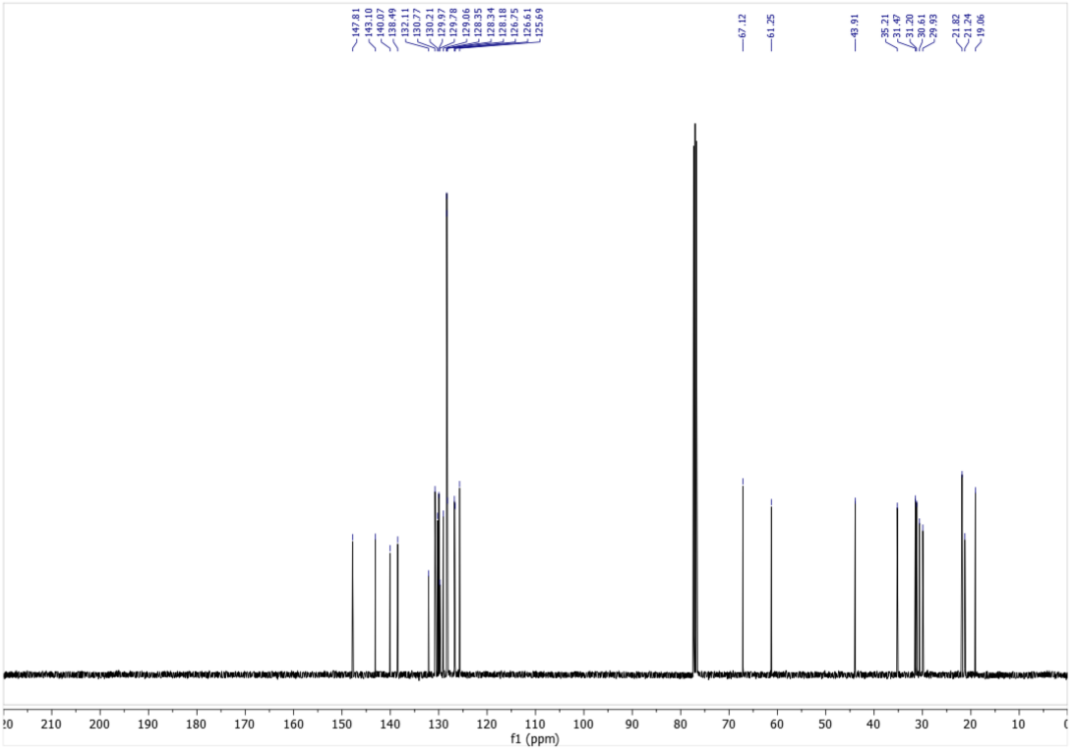

11-(4-(4-Methyl-1-phenylpentan-3-yl)piperazin-1-yl)dibenzo[b,f][1,4]thiazepine (8b)

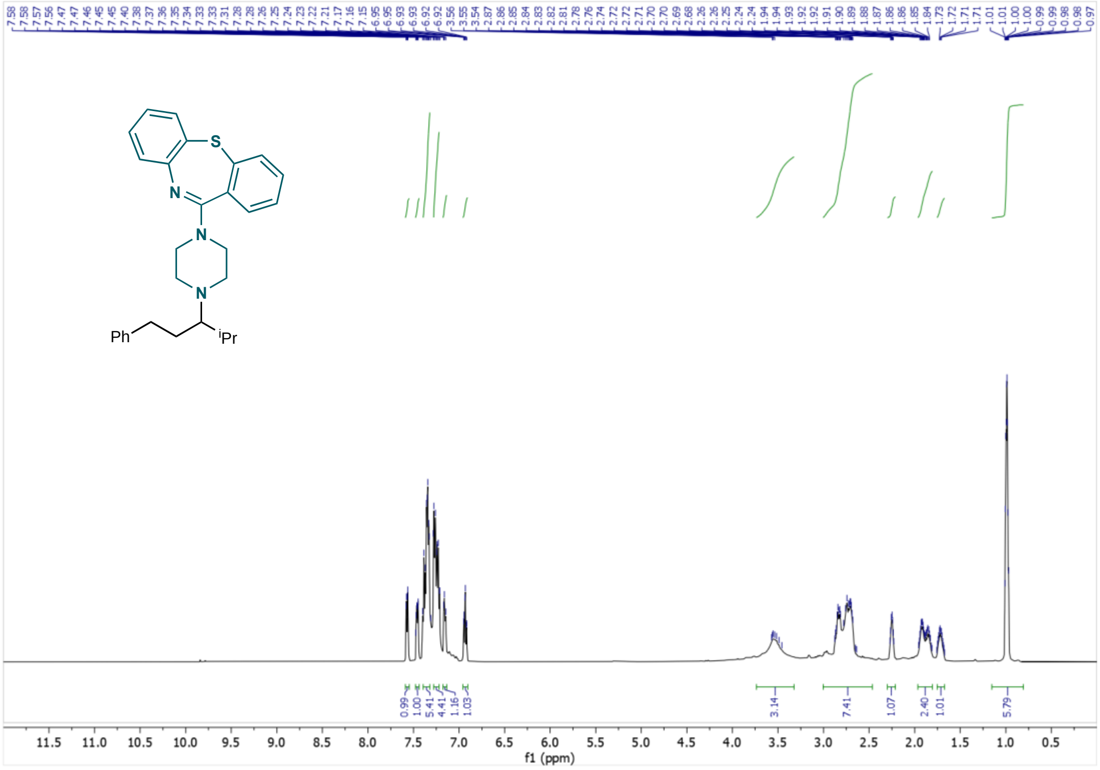

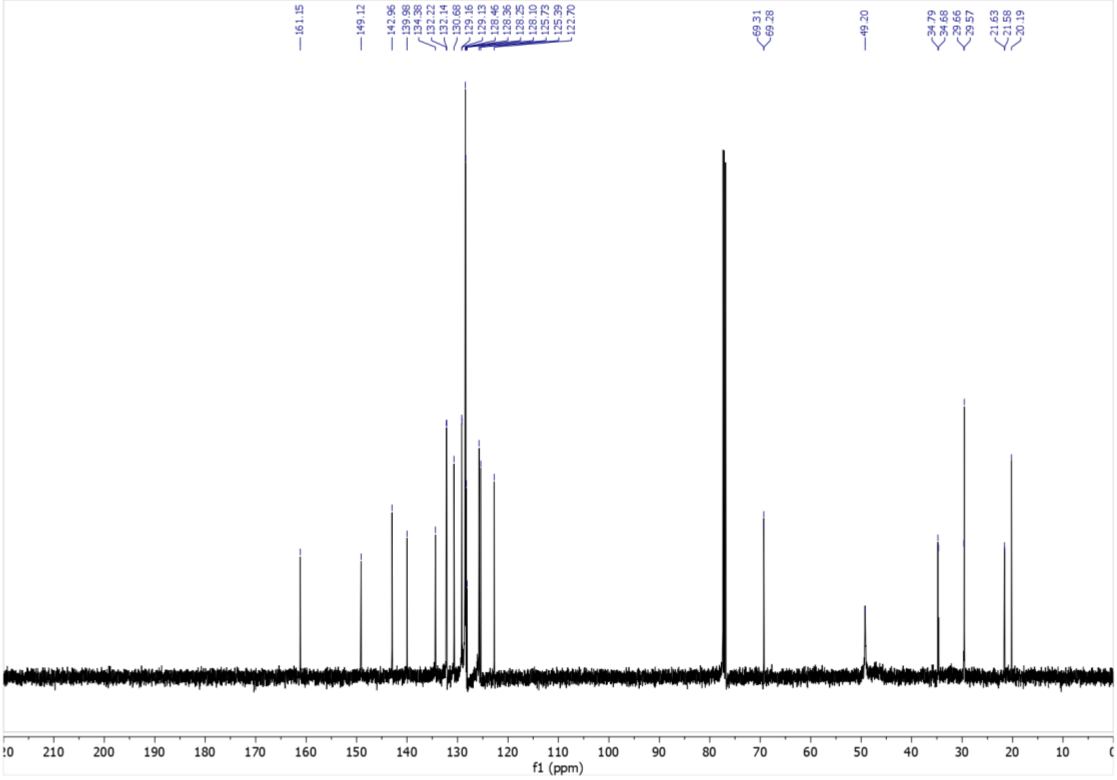

Ethyl 1-cyclopropyl-6-fluoro-7-(4-(4-methyl-1-phenylpentan-3-yl)piperazin-1-yl)-4-oxo-1,4-dihydroquinoline-3-carboxylate (8c)

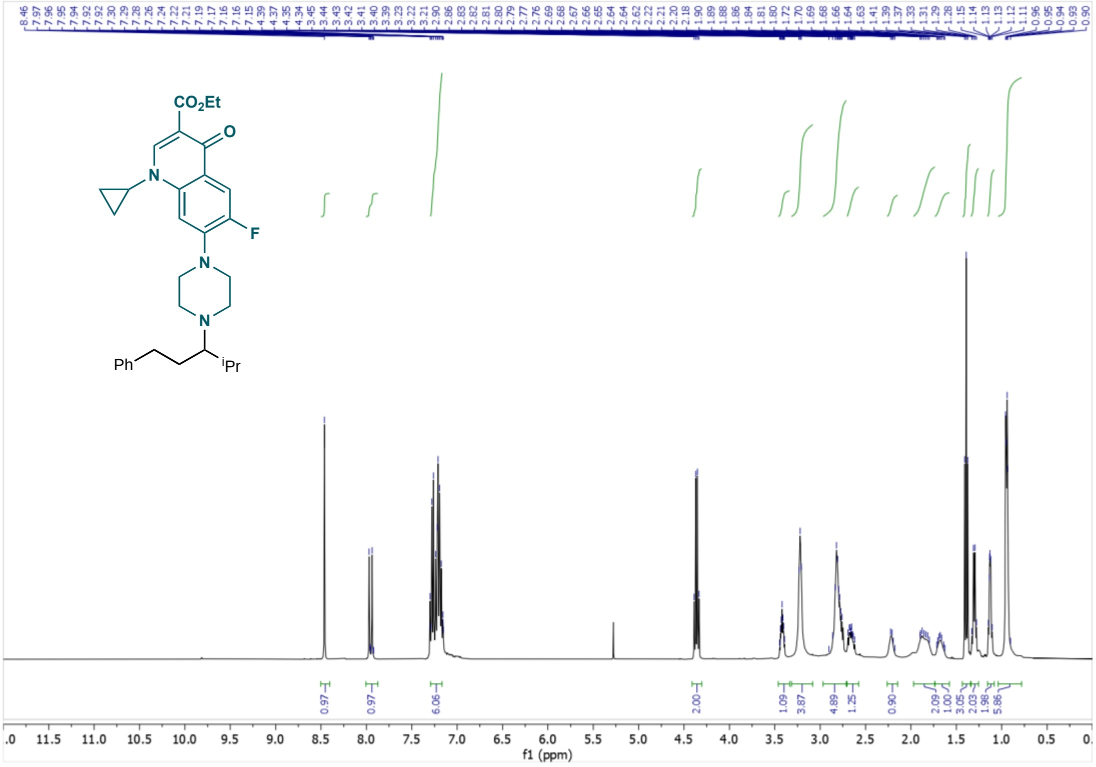

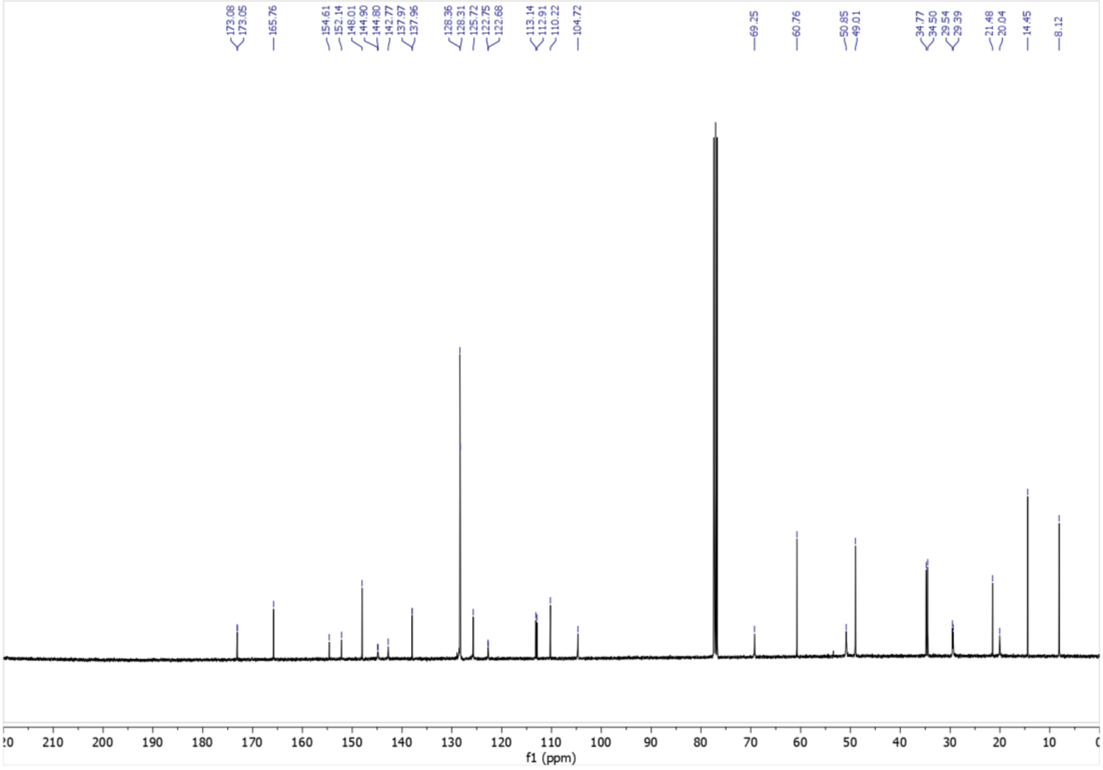

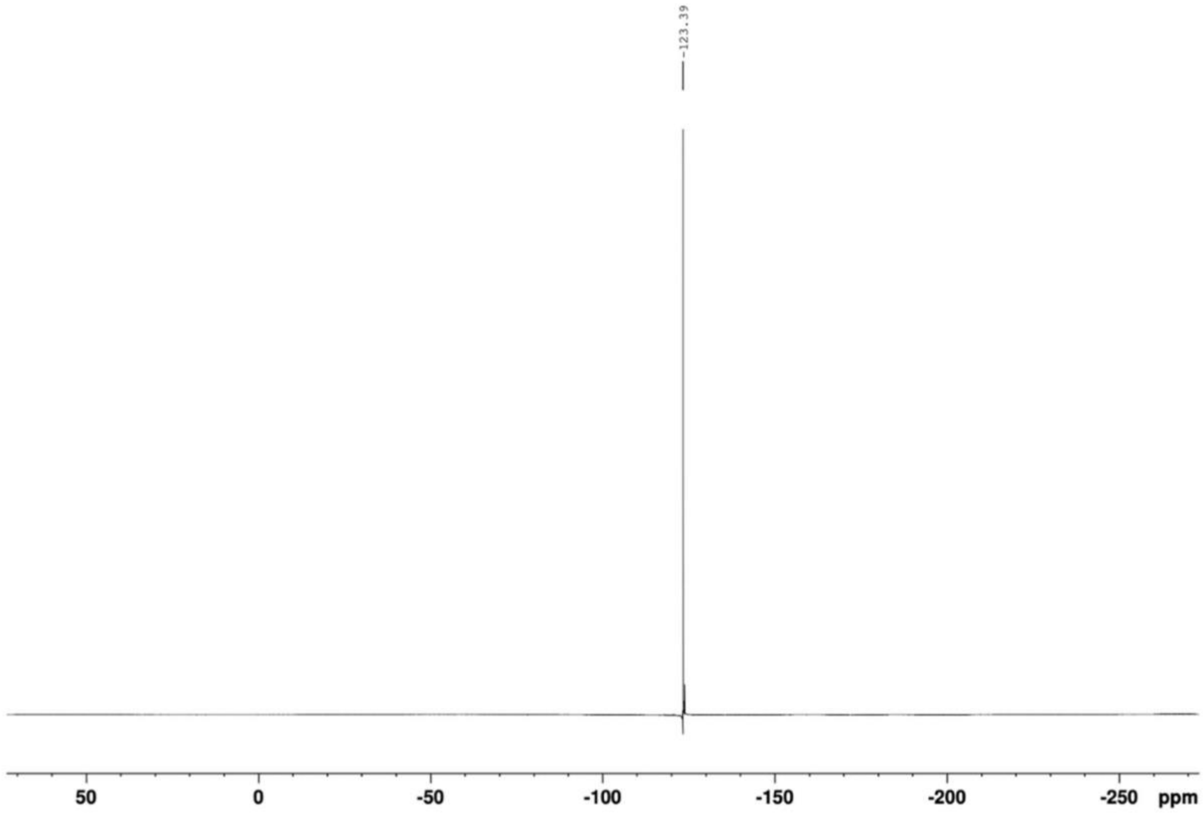
-123.39
50
0
-50
-100
-150
-200
-250
ppm

Diethyl-4-(6-chlorocyclohexa-1,2,3,5-tetraen-1-yl)-2-methyl-6-((2-((4-methyl-1-phenylpentan-3-yl)amino)ethoxy)methyl)-1,4-dihydropyridine-3,5-dicarboxylate (8d)

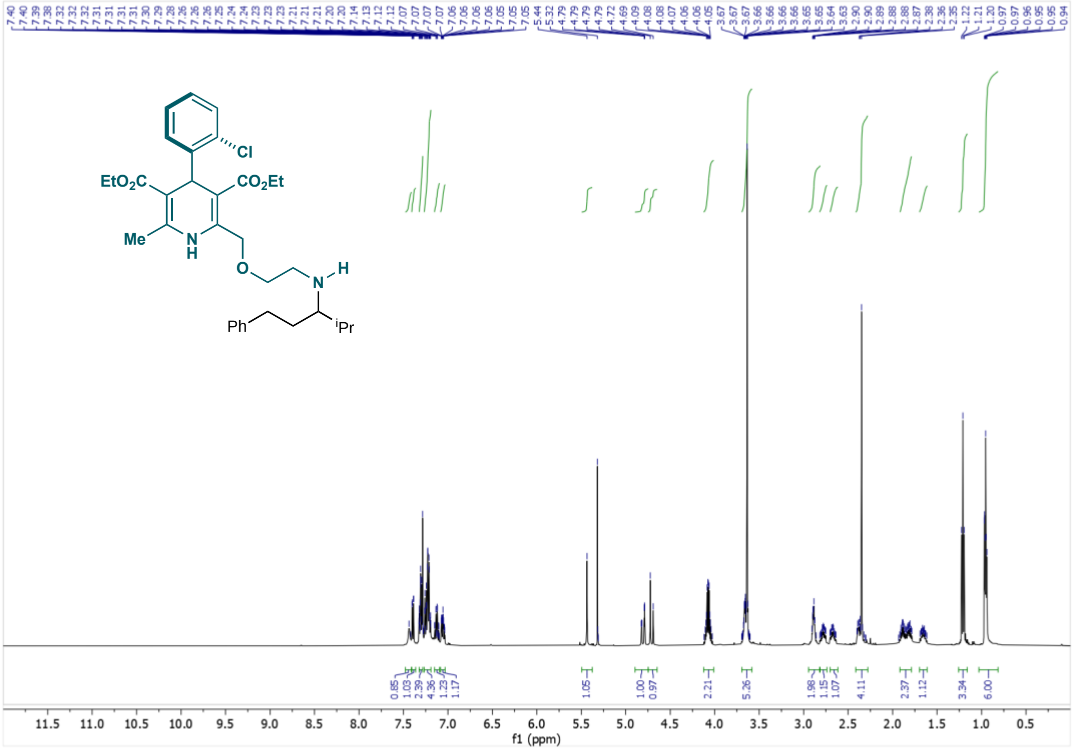

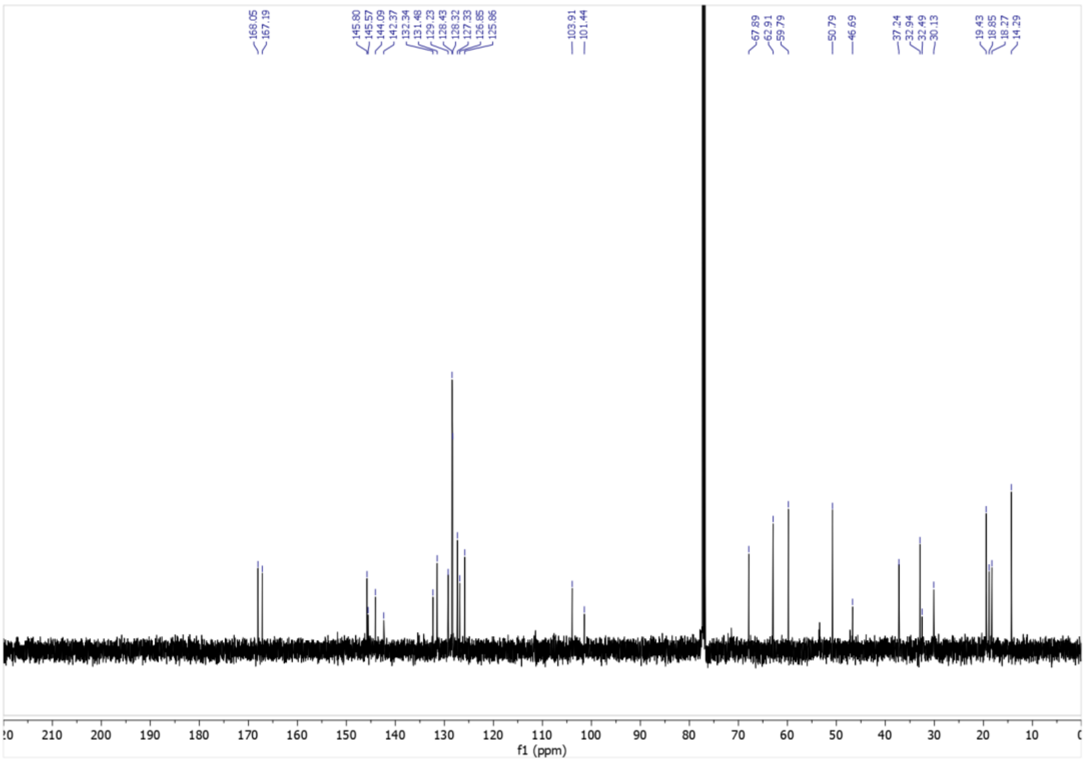

N-benzyl-N,2-dimethyltridec-12-en-3-amine (9a)

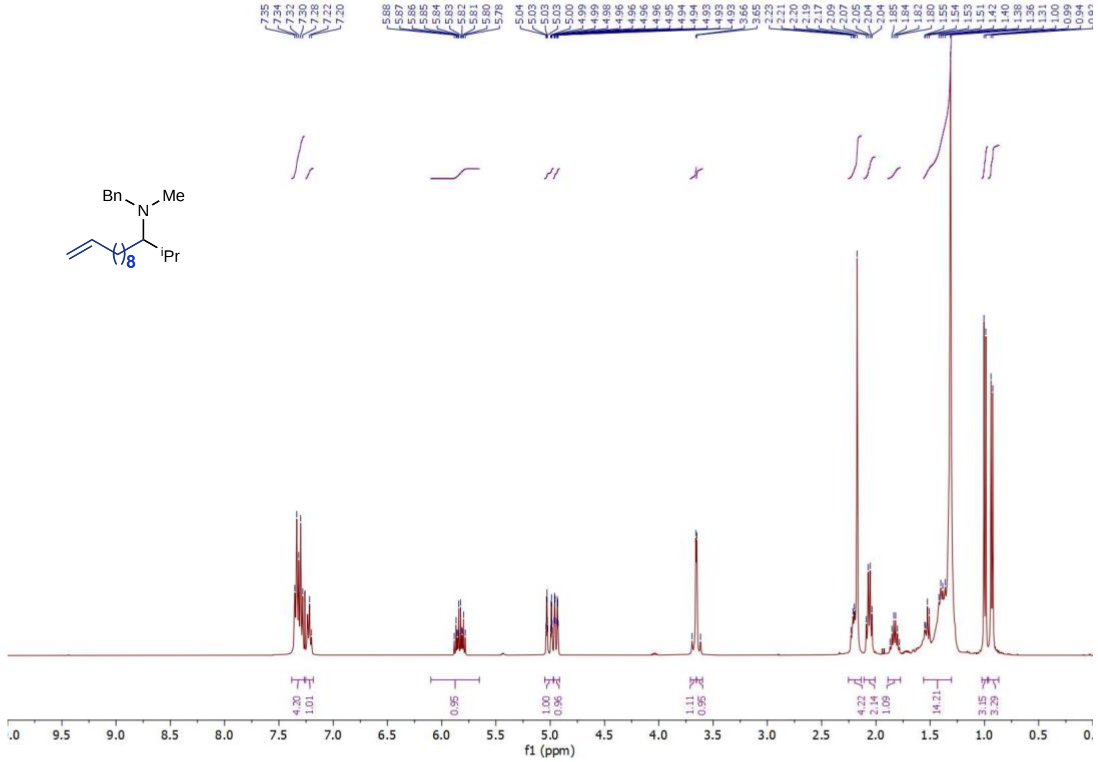

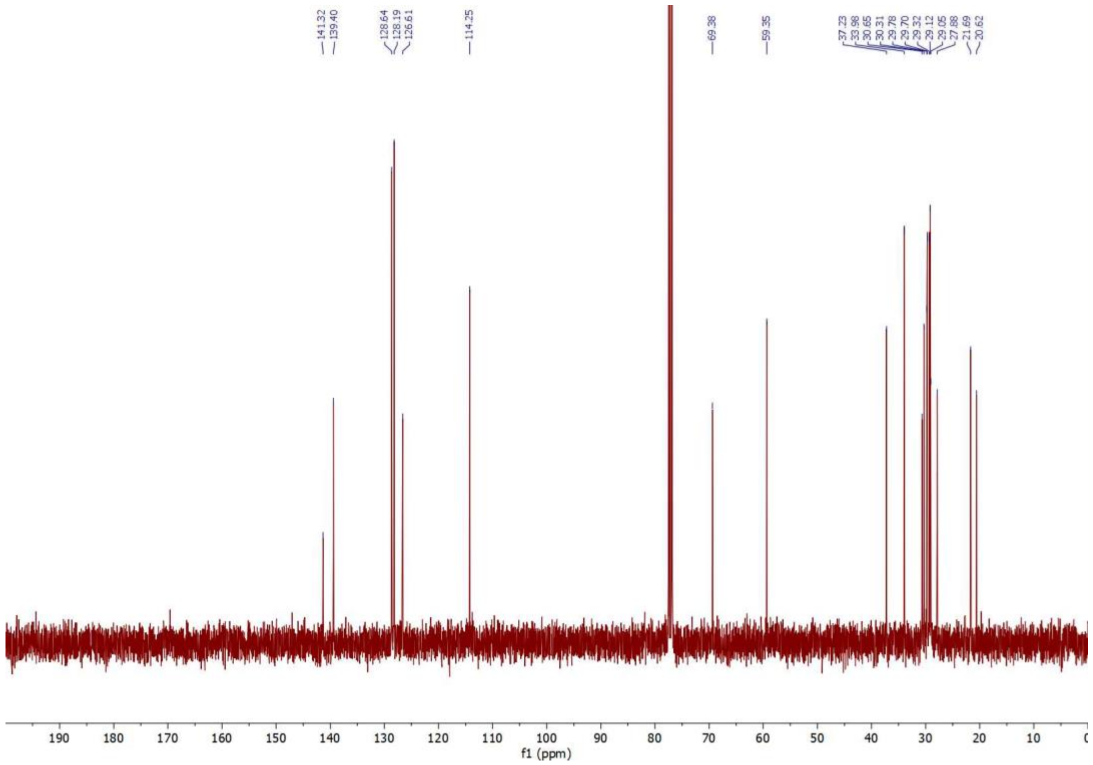

Ethyl 6-(benzyl(methyl)amino)-7-methyloctanoate (9b)

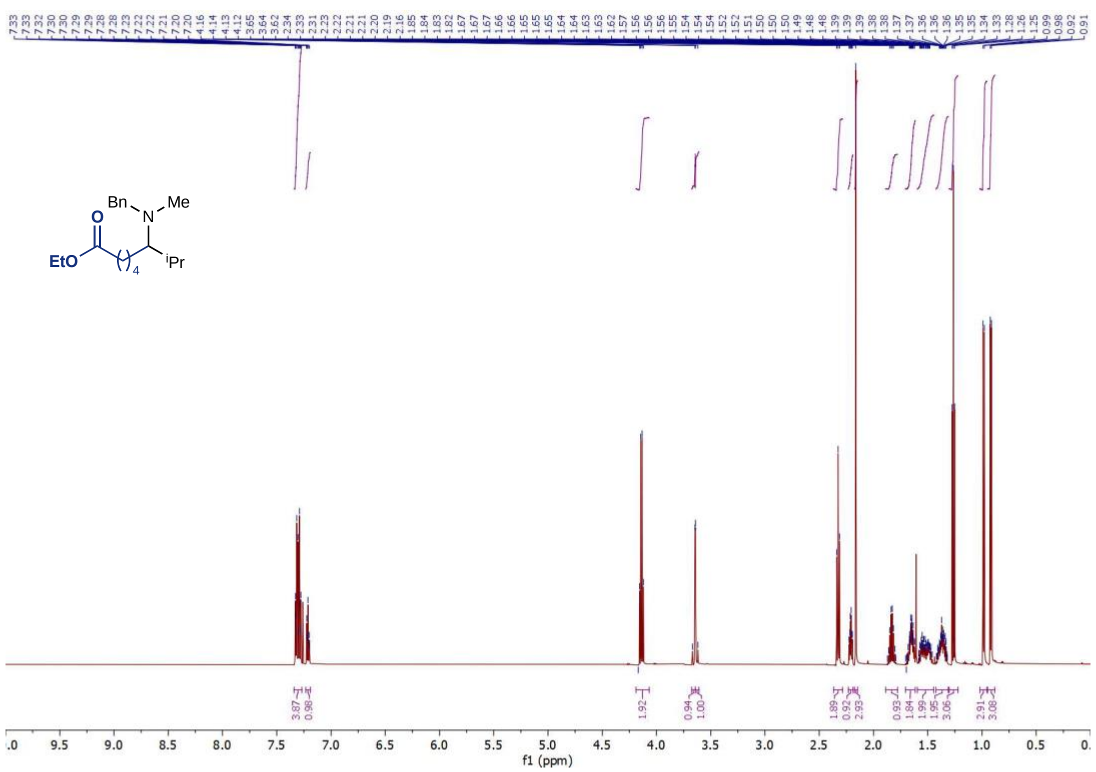

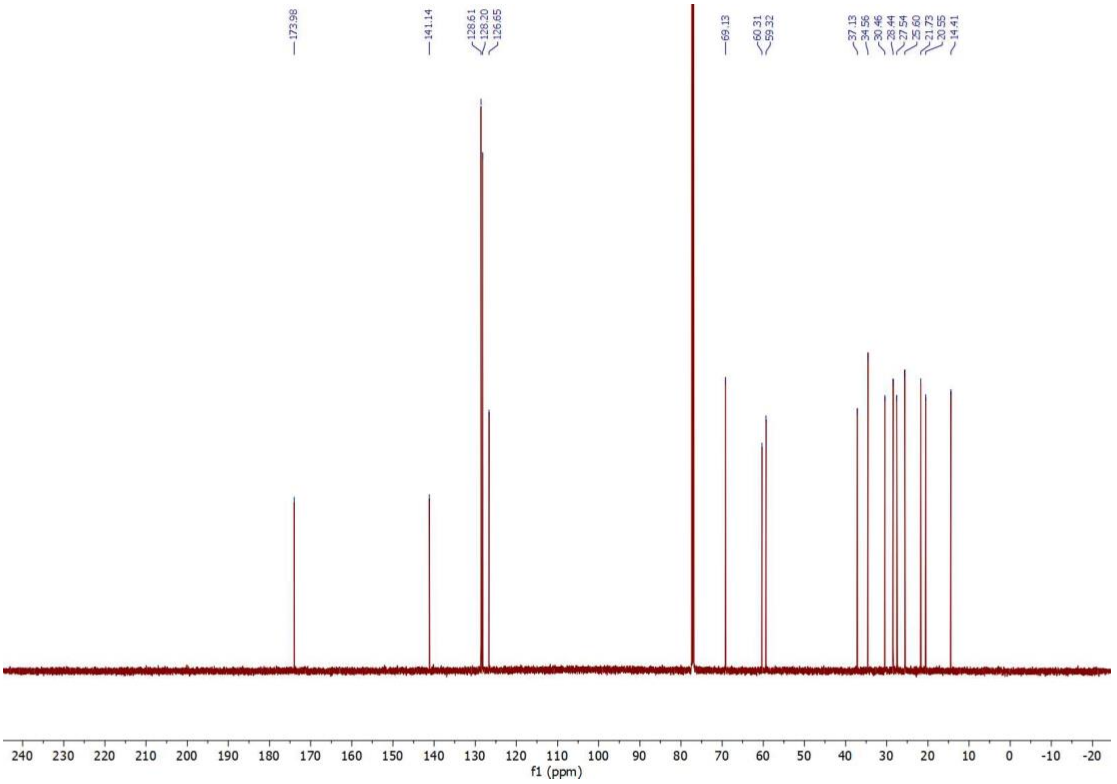

6-(1,2,3,4-tetrahydro-9H-carbazol-9-yl)hexanal (9c -precusor 1)

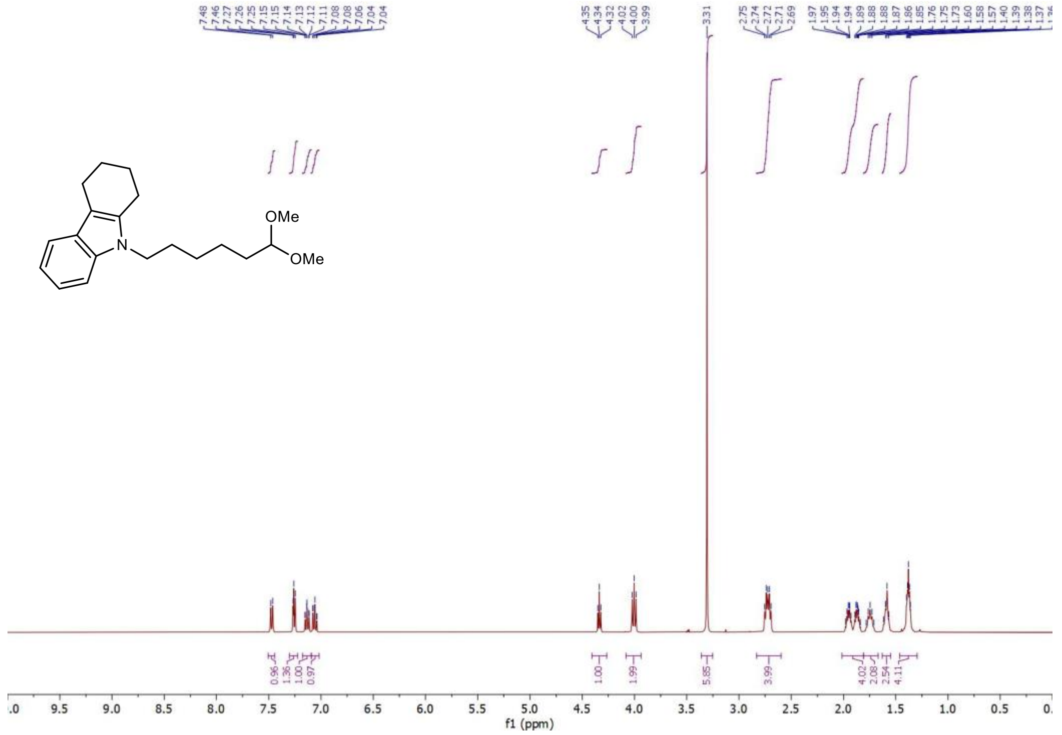

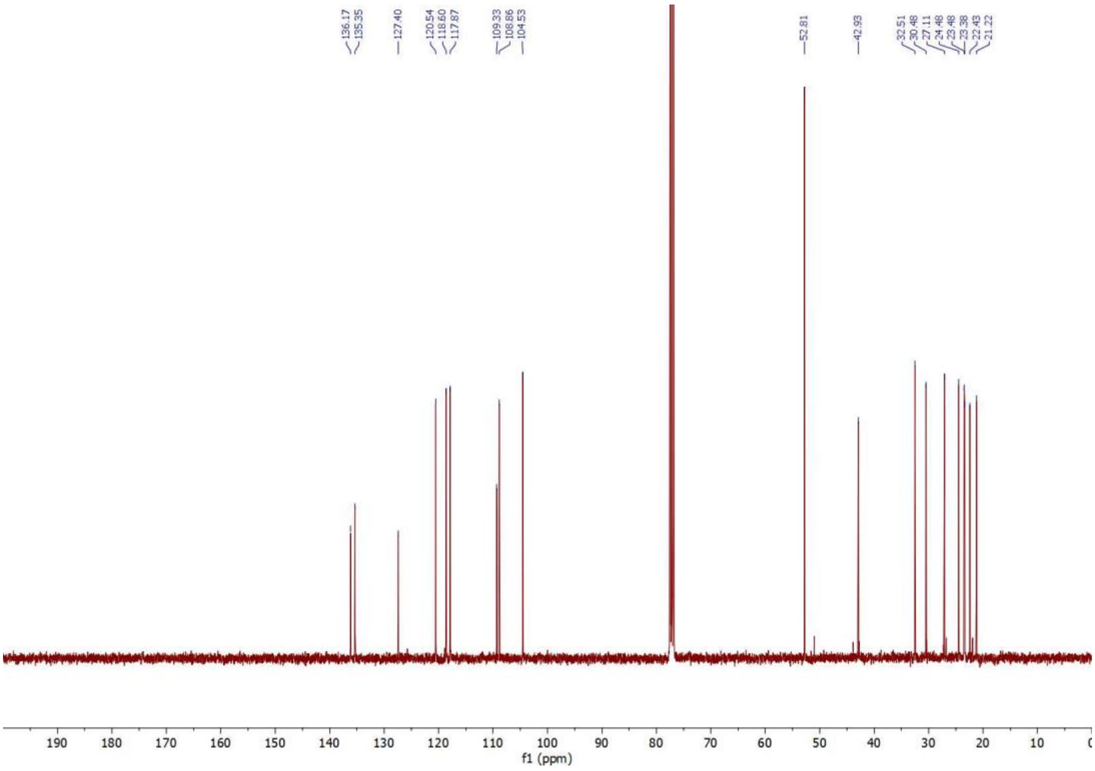

9-(6,6-dimethoxyhexyl)-2,3,4,9-tetrahydro-1H-carbazole (9c –aldehyde)

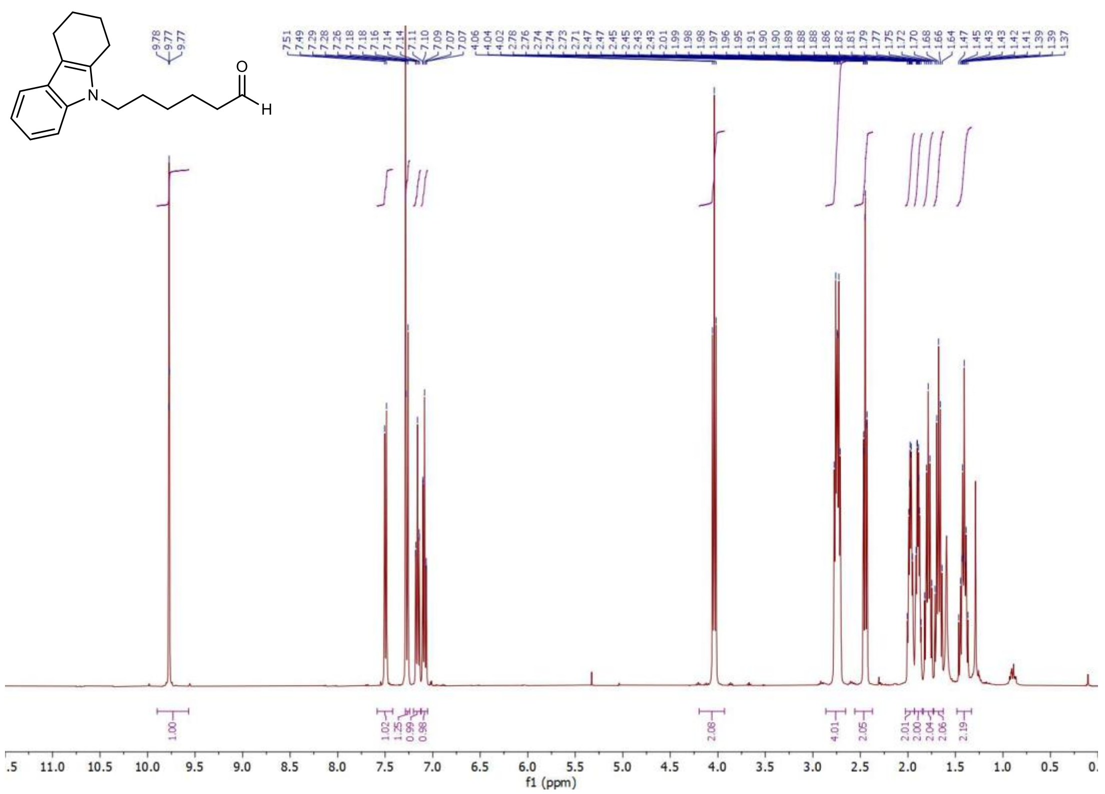

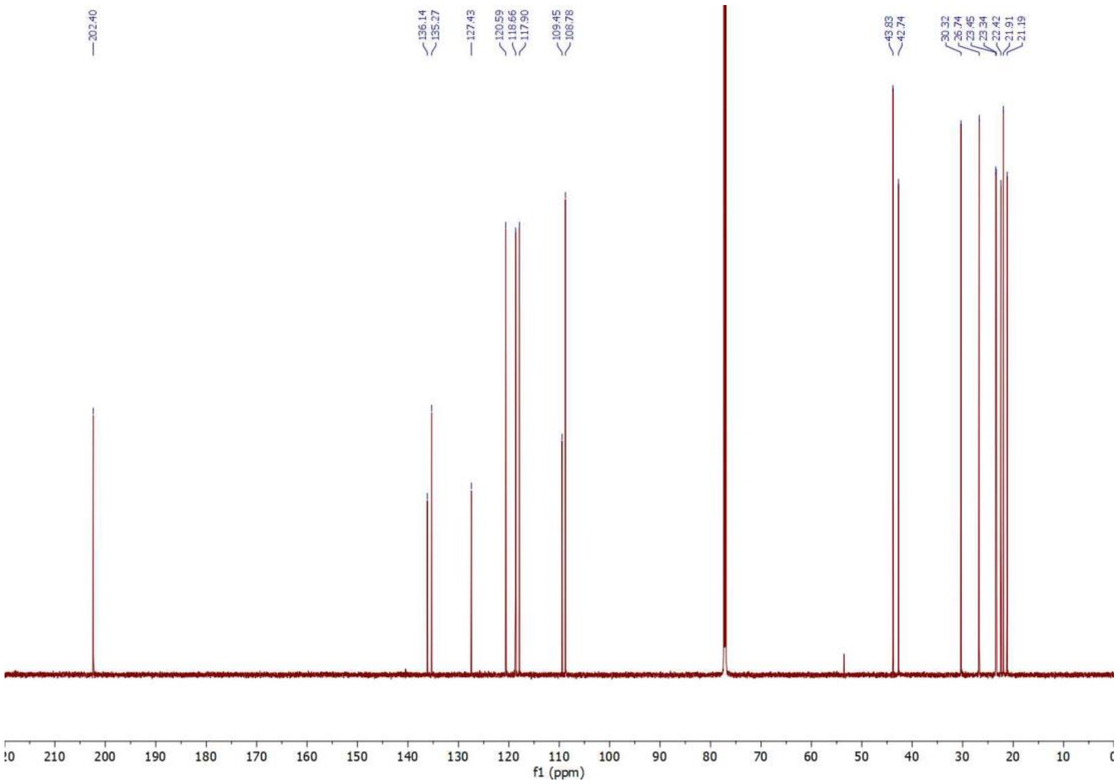

N-benzyl-N,2-dimethyl-8-(1,2, 3, 4-tetrahydro-9H-carbazol-9-yl)octan-3-amine (9c)

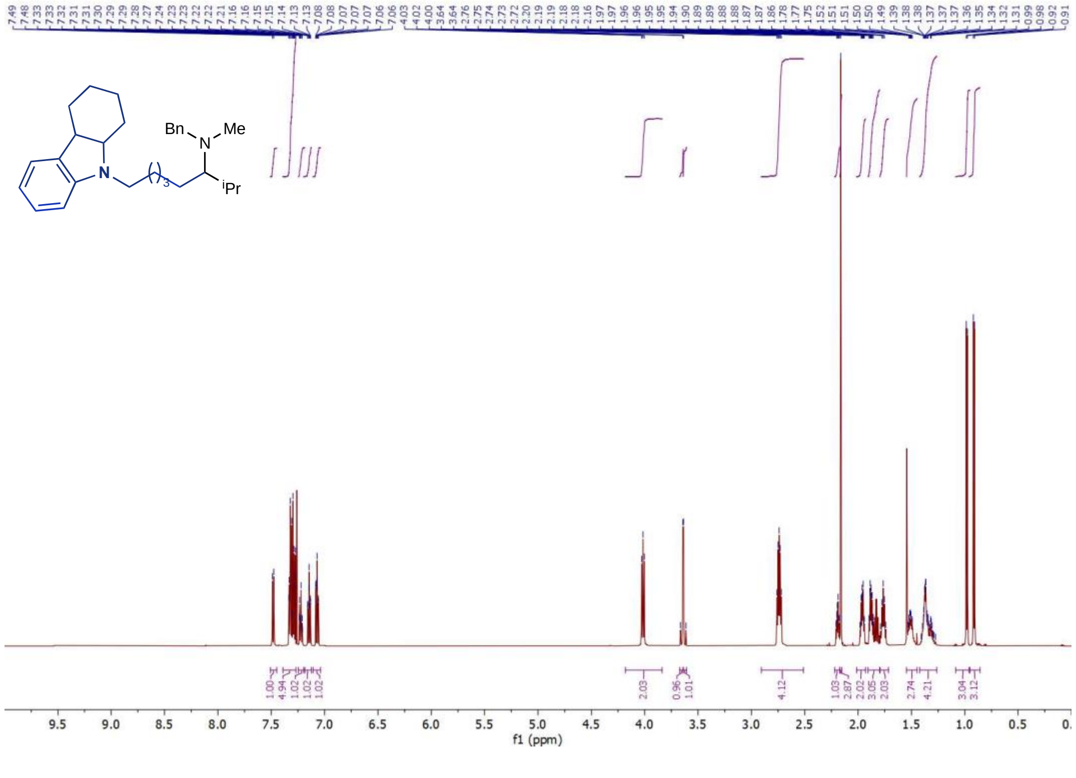

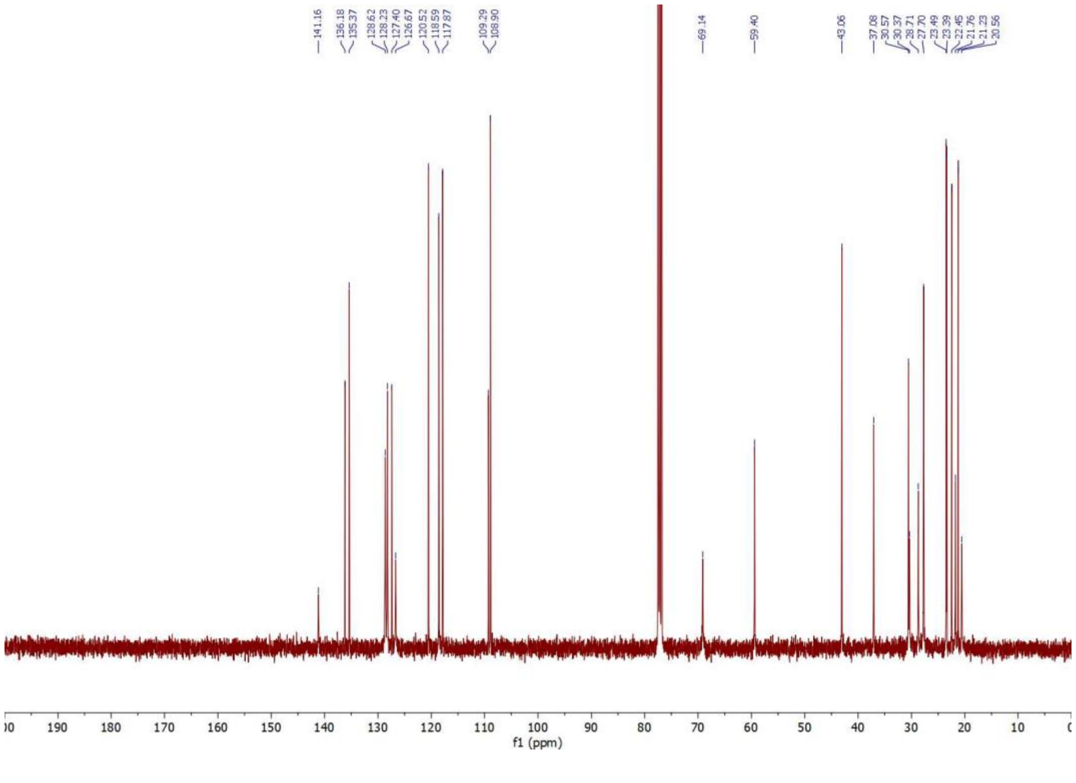

Benzyl 4-(2-(benzyl(methyl) amino)-3-methylbutyl)piperidine-1-carboxylate (9d)

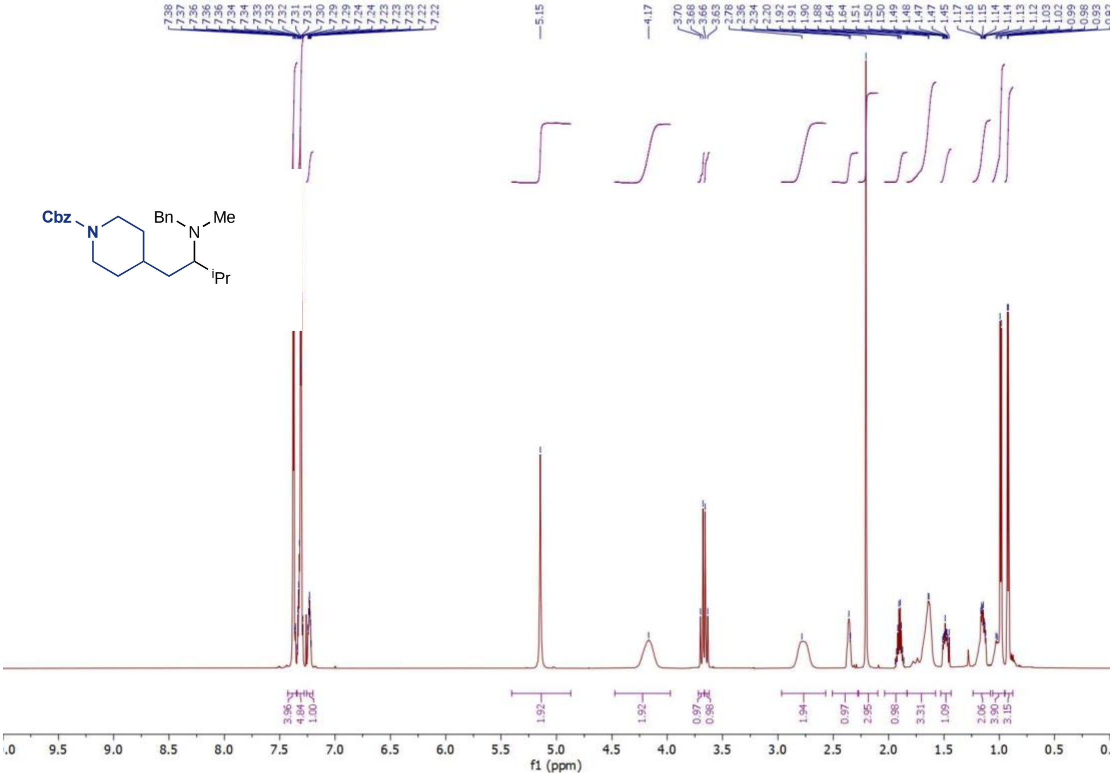

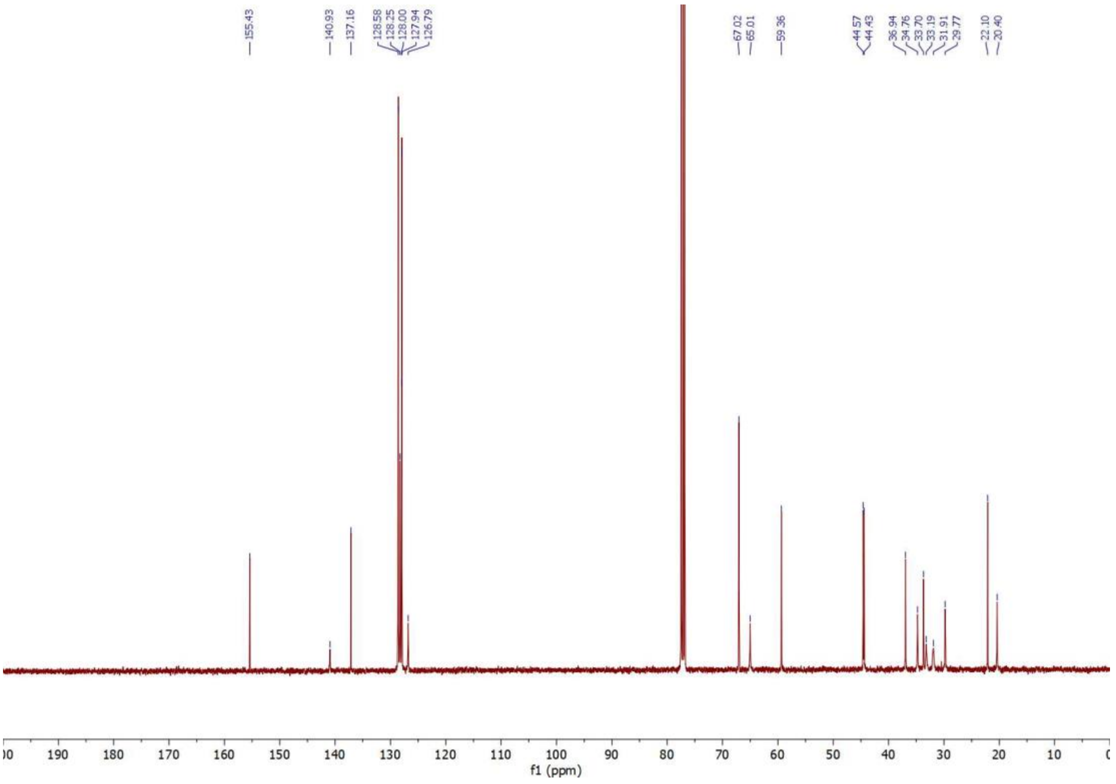

N-benzyl-N,2,5-trimethylhexan-3-amine (9e)

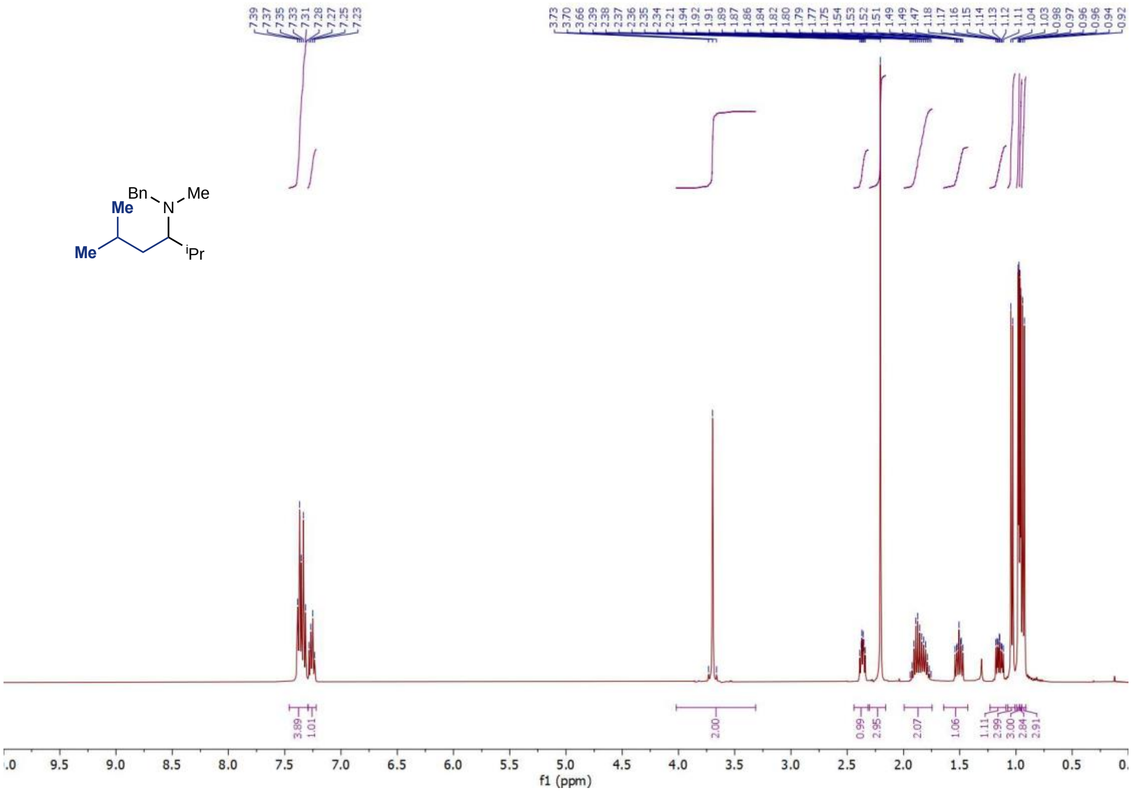

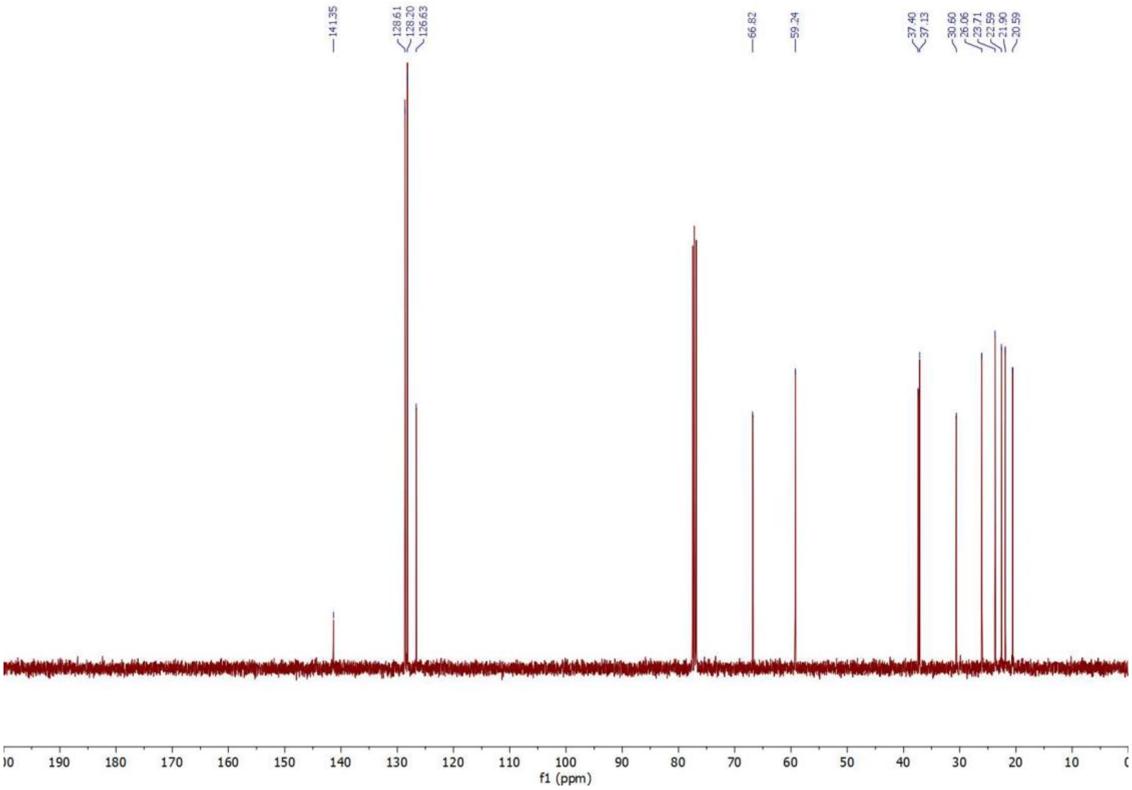

N-benzyl-N,2,5,5-tetramethylhexan-3-amine (9f)

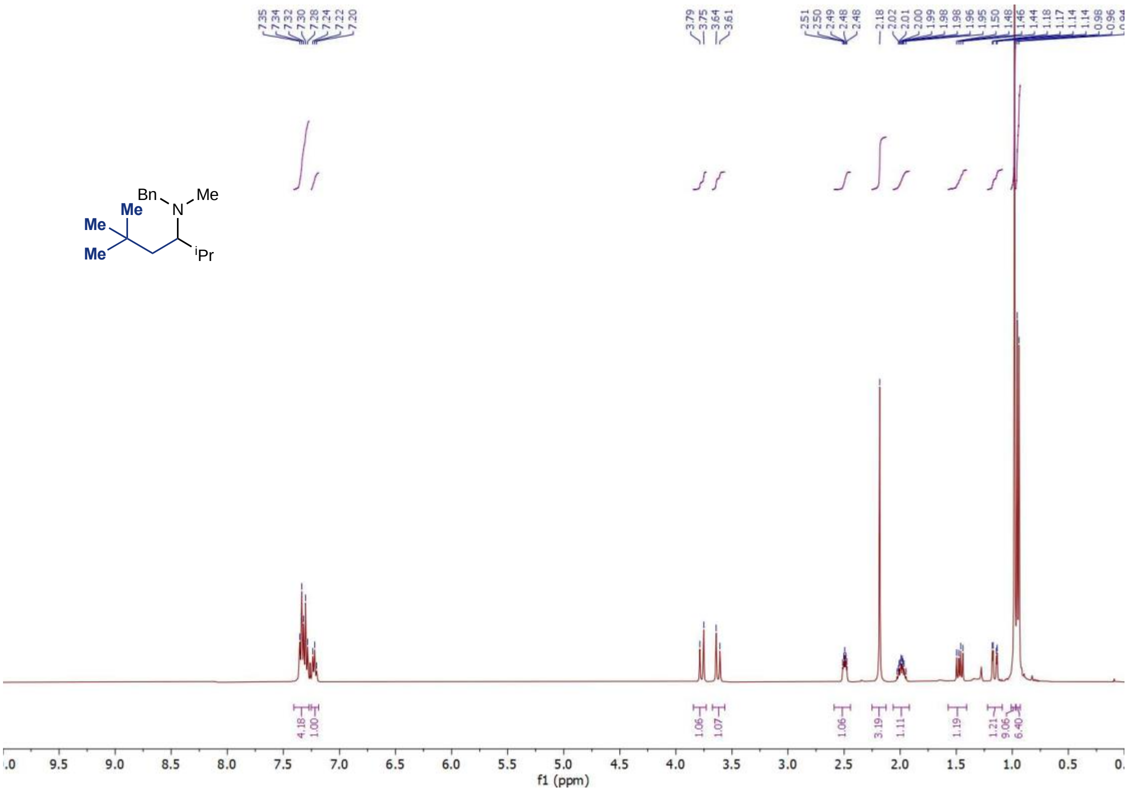

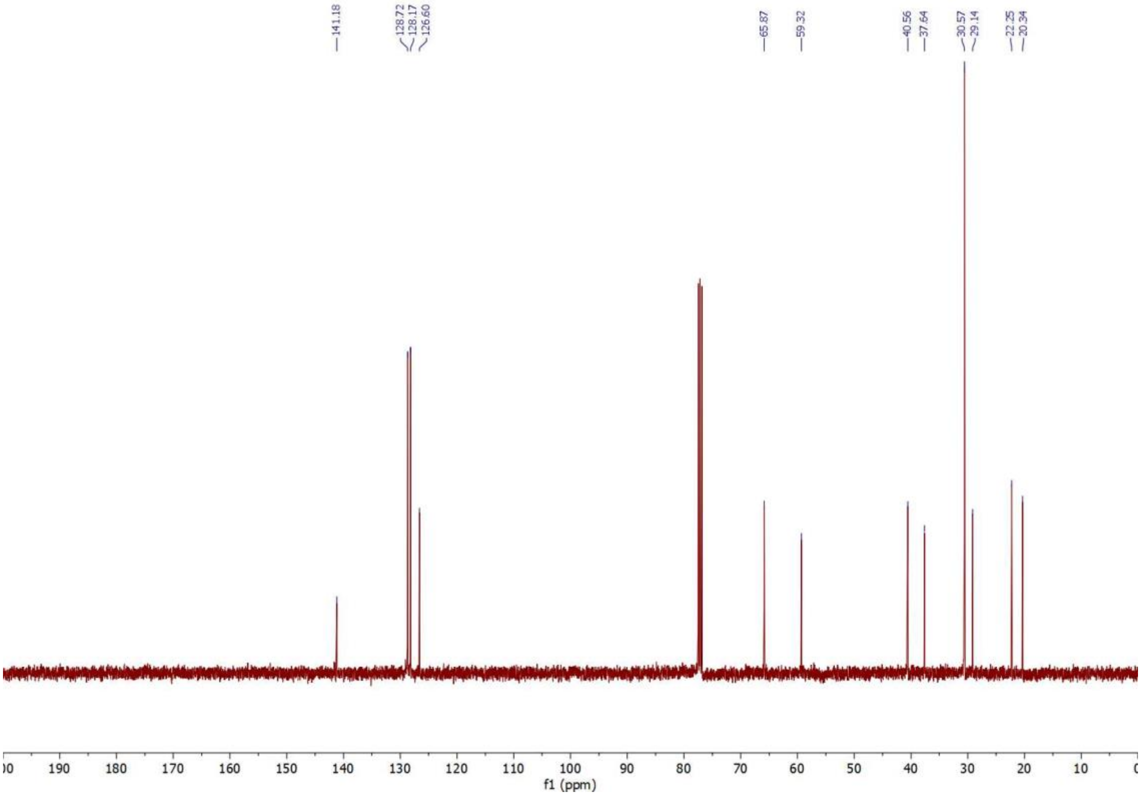

N-benzyl-1-(cyclohex-3-en-1-yl)-N,2-dimethylpropan-1-amine (9h)

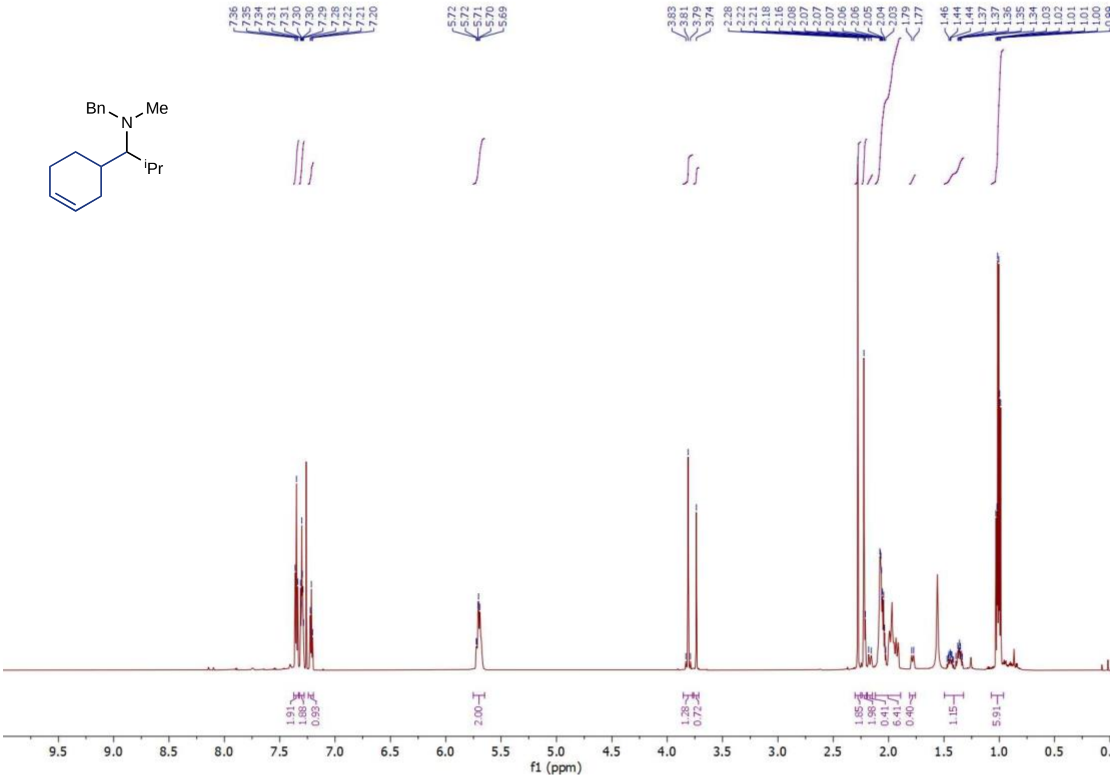

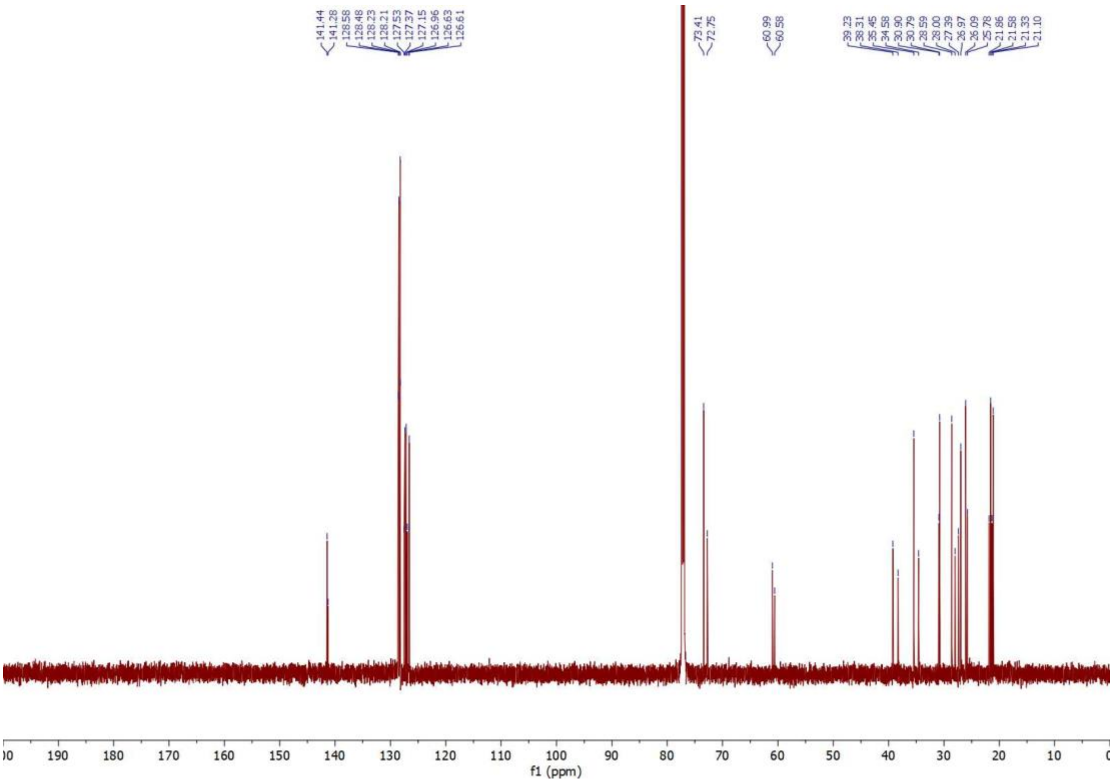

N-benzyl-N,3-dimethyl-1-phenylbutan-2-amine (9j)

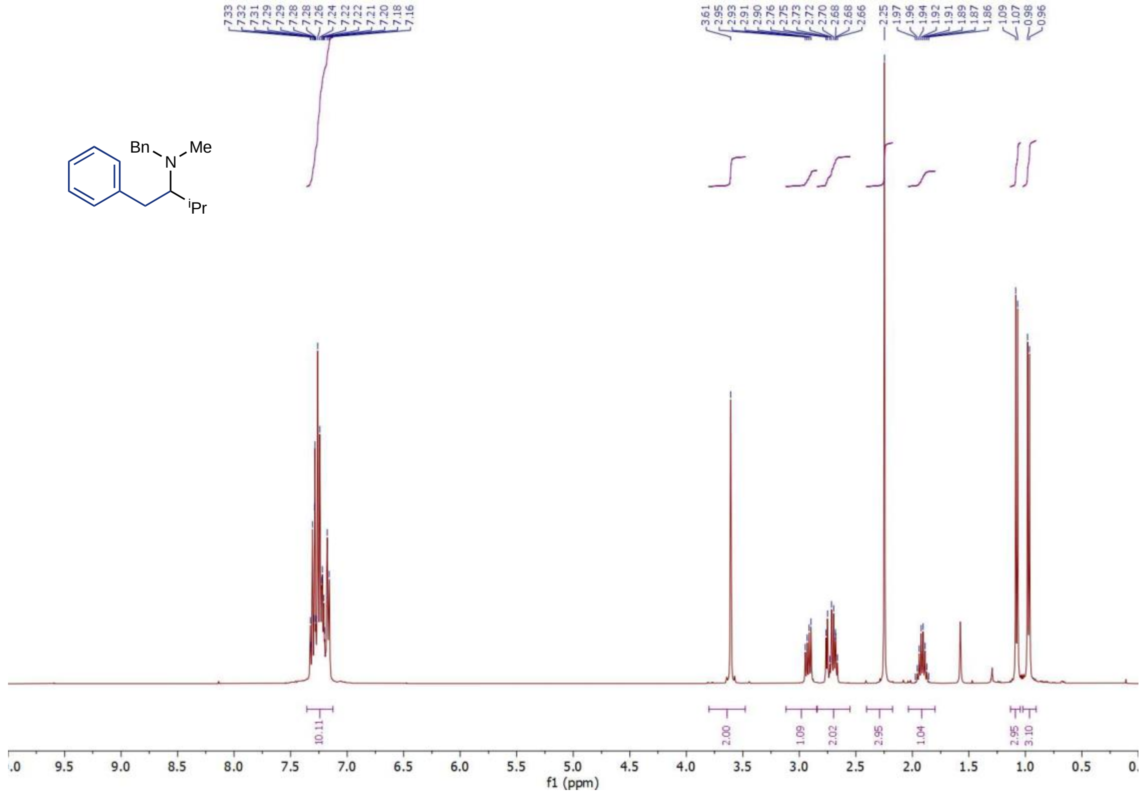

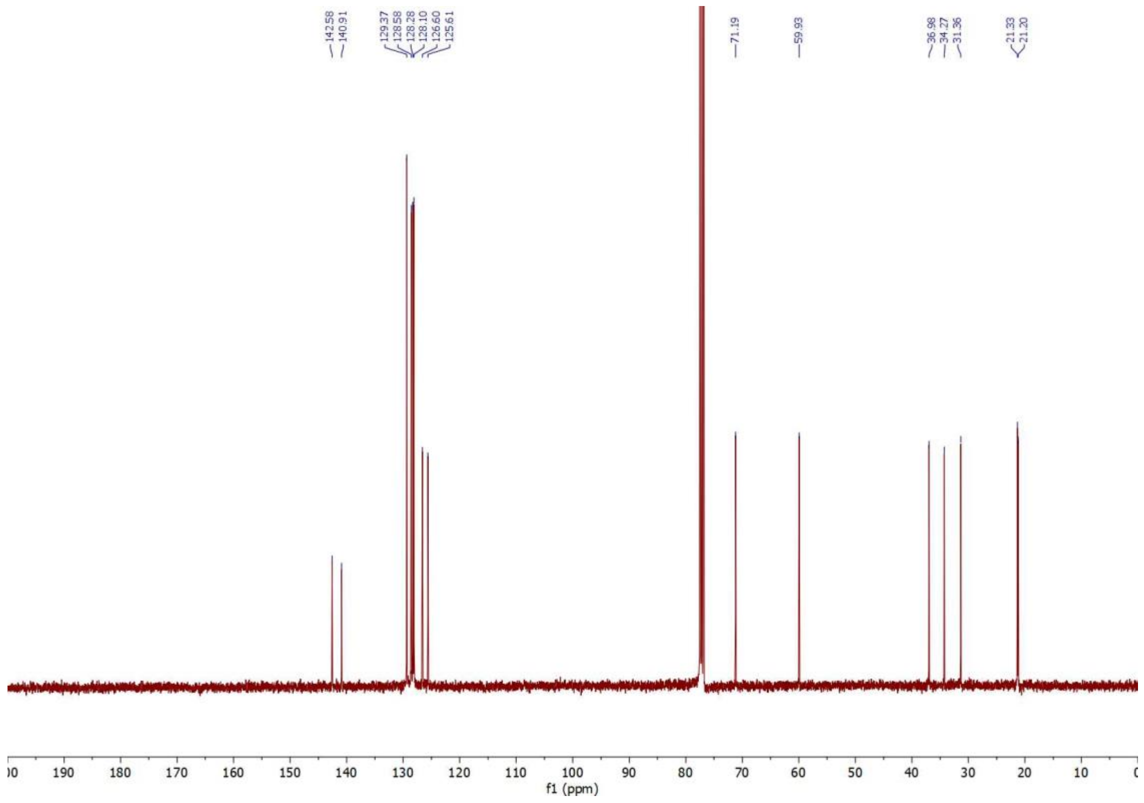

N-benzyl-1-(3,4-dimethoxyphenyl)-N,3-dimethylbutan-2-amine (9k)

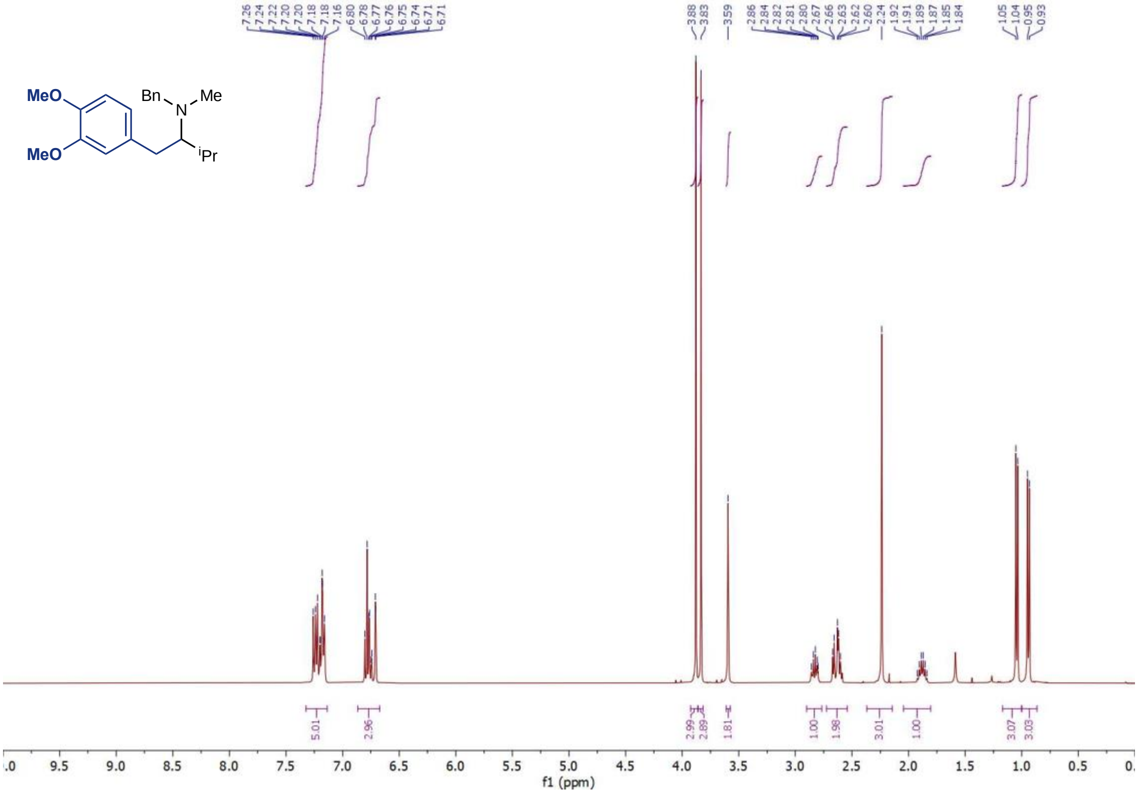

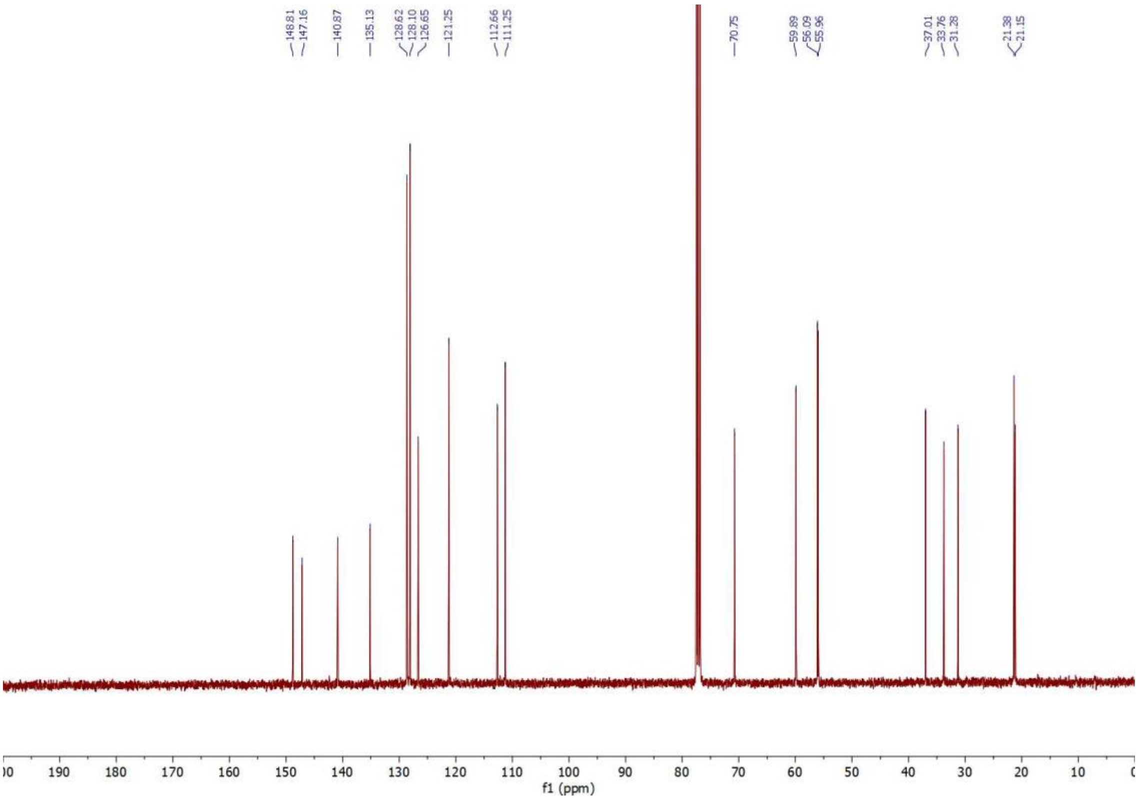

N-Benzyl-N-methyl-1-phenylpropan-3-amine (9l)

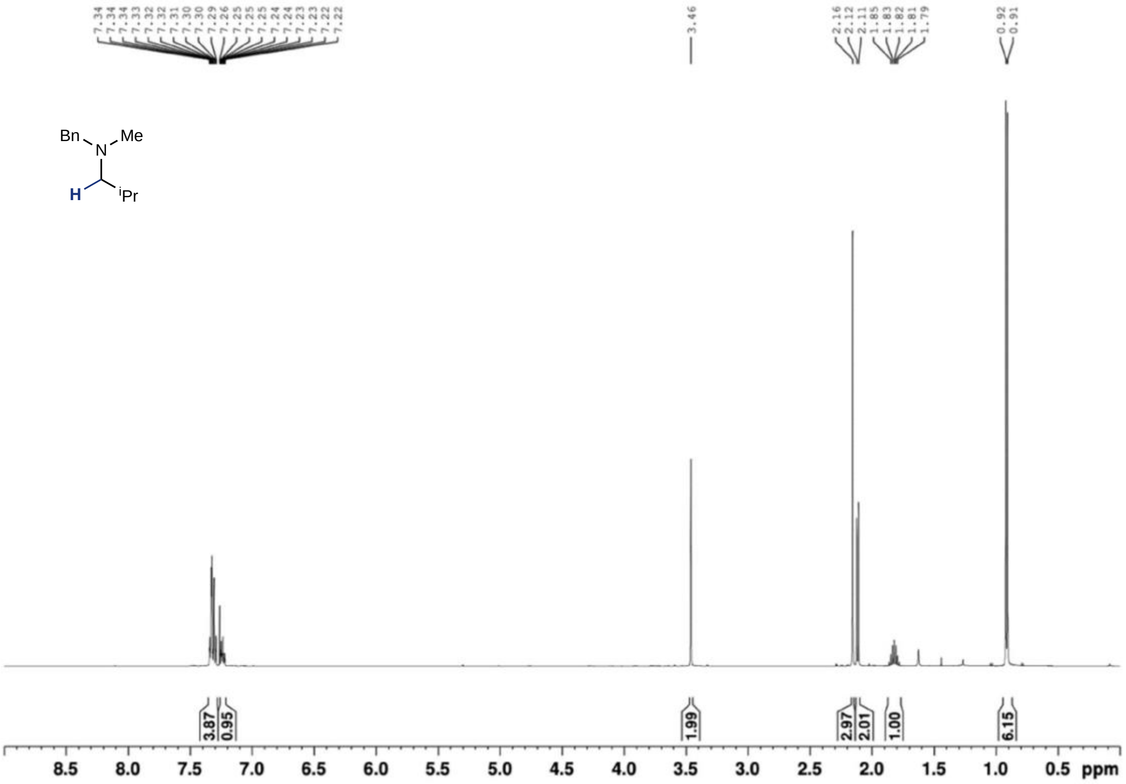

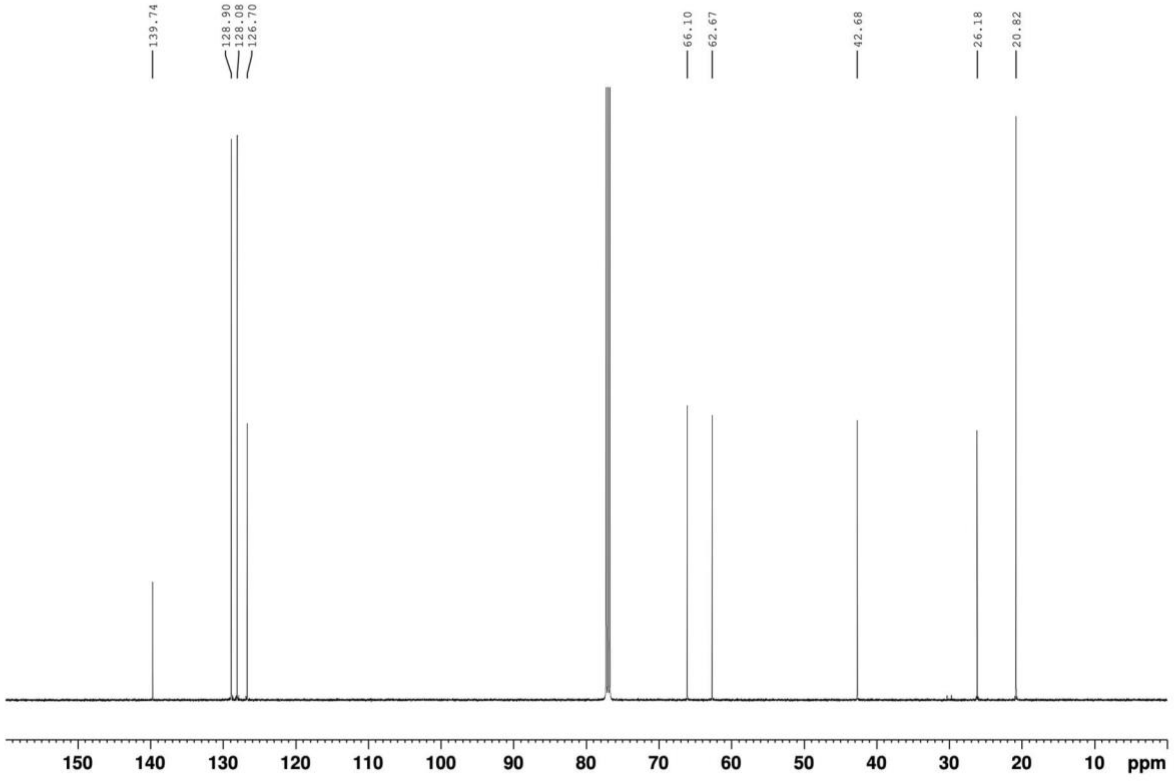

N-benzyl-1-cyclohexyl-N-methyl-3-phenylpropan-1-amine (10a)

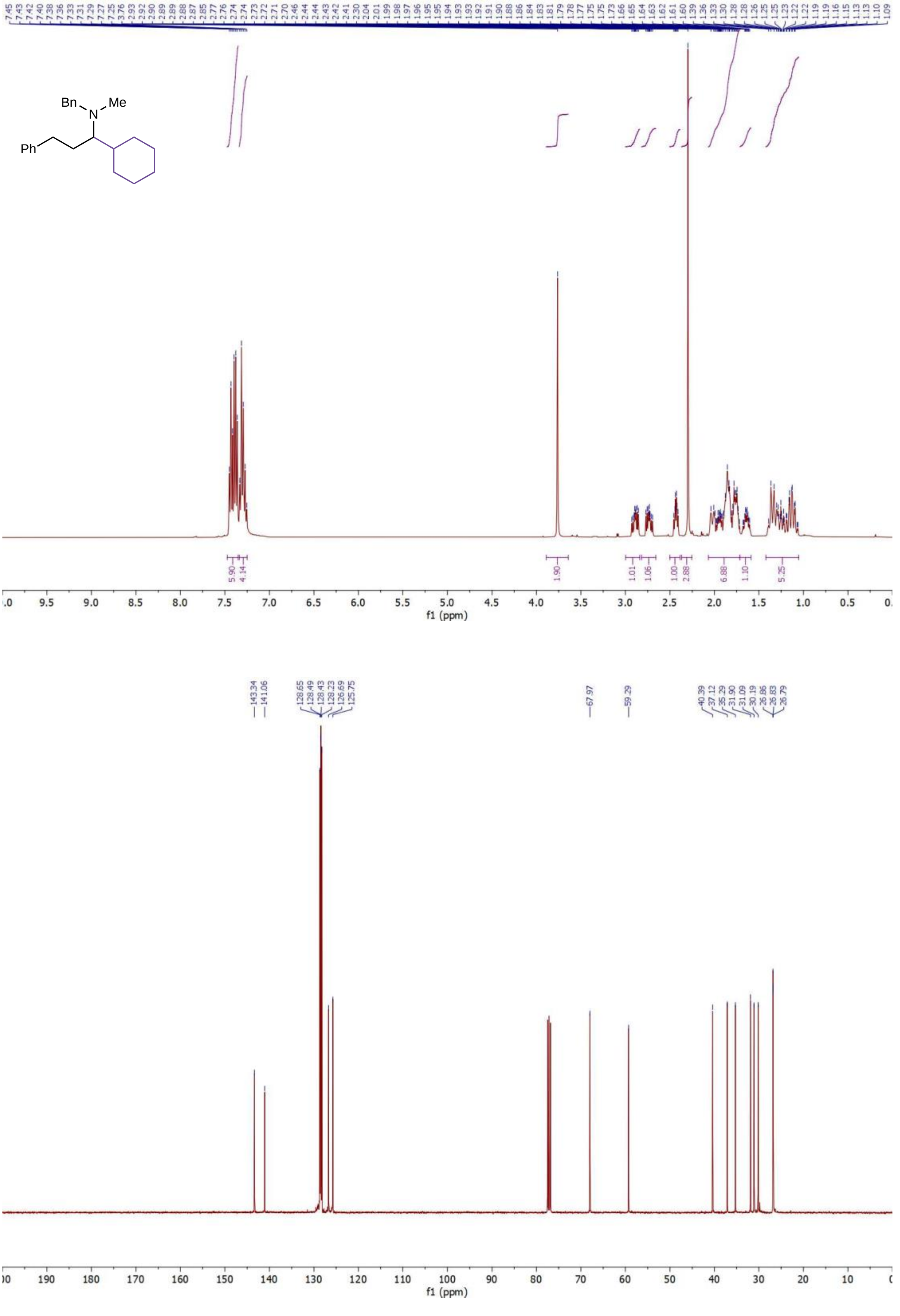

N-benzyl-N-methyl-3-phenyl-1-(tetrahydro-2H-pyran-4-yl)propan-1-amine (10b)

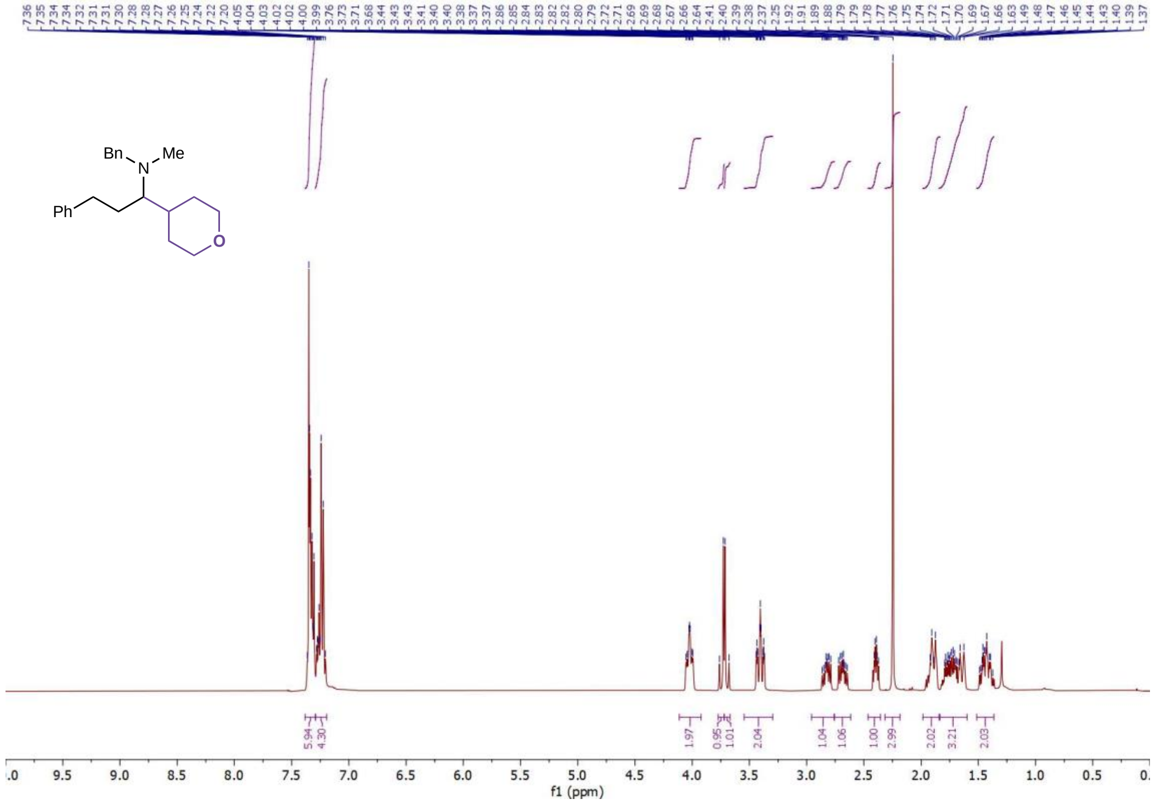

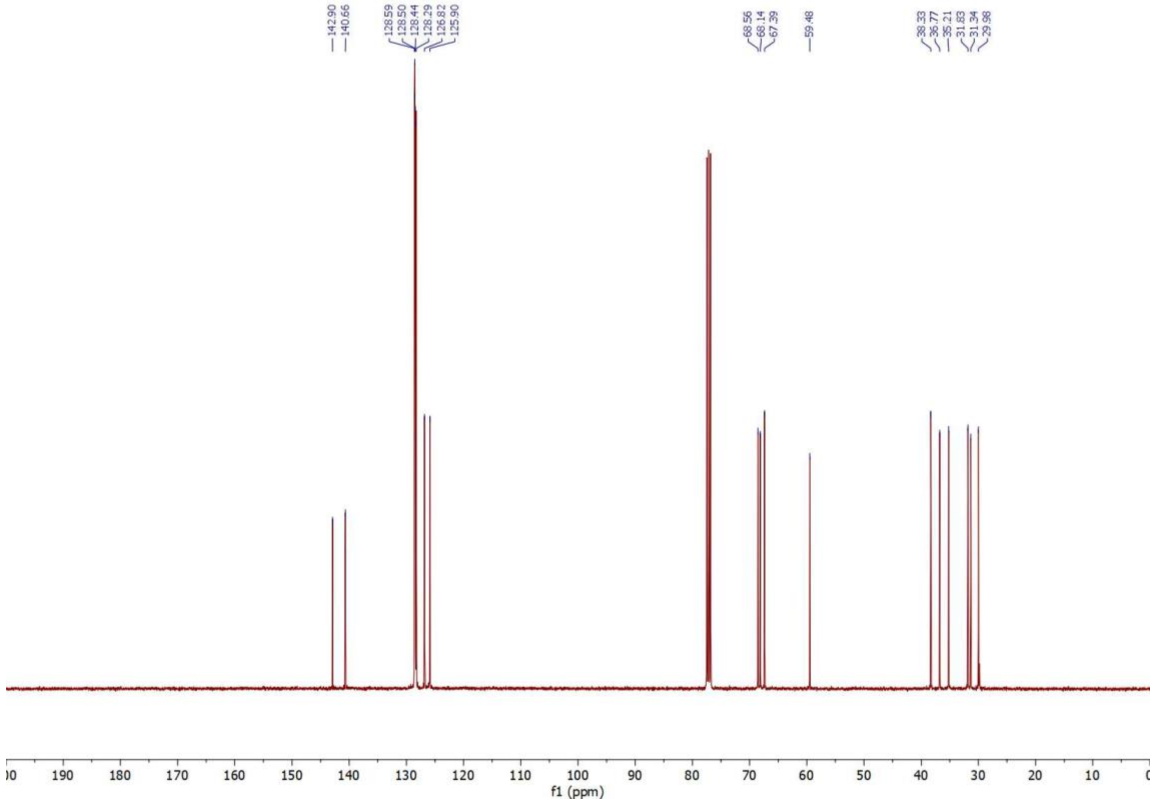

N-benzyl-1-cyclopentyl-N-methyl-3-phenylpropan-1-amine (10d)

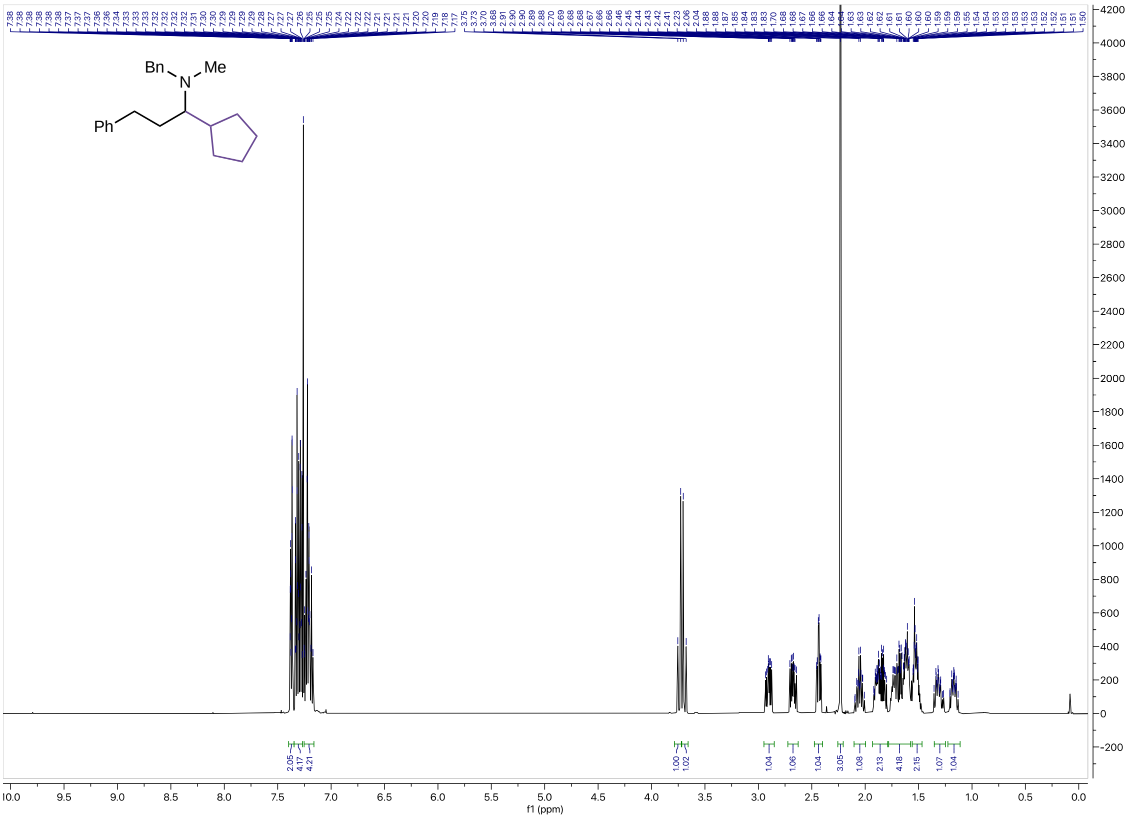

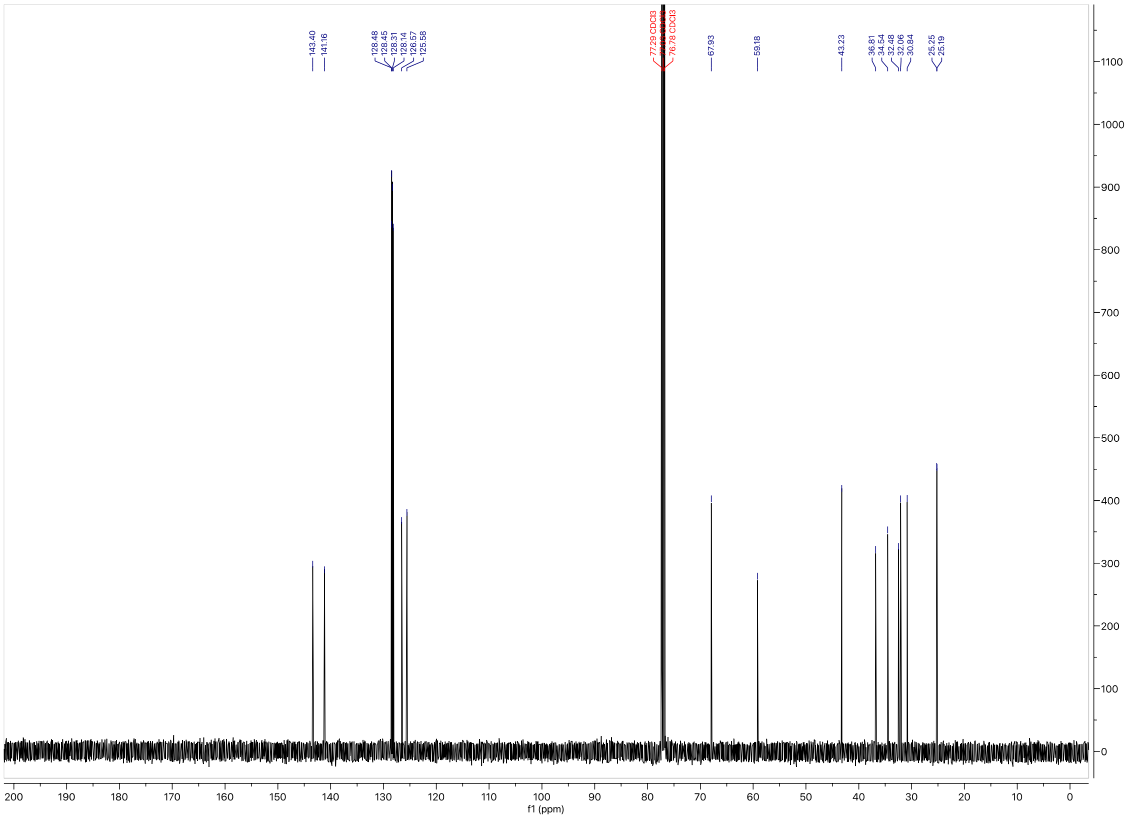

N-benzyl-N-methyl-4-methyl-1-phenylhexan-3-amine (10e)

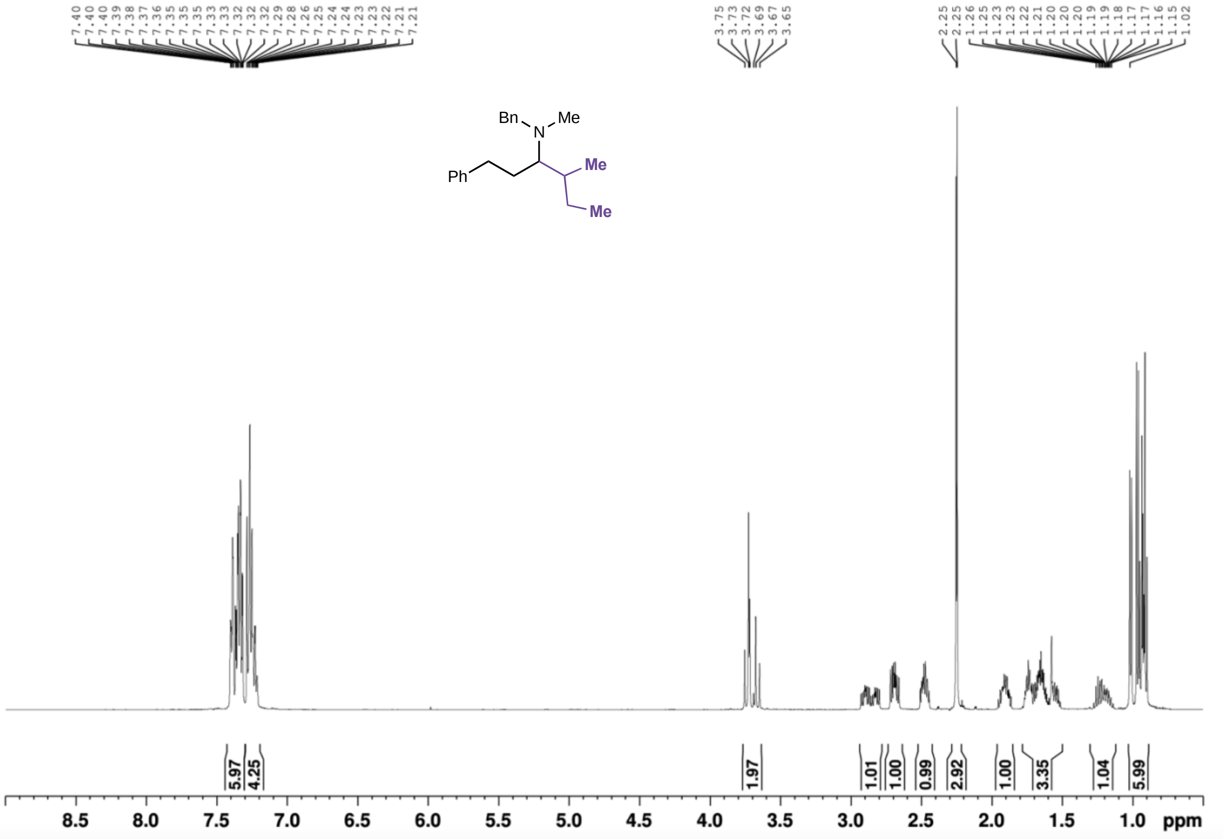

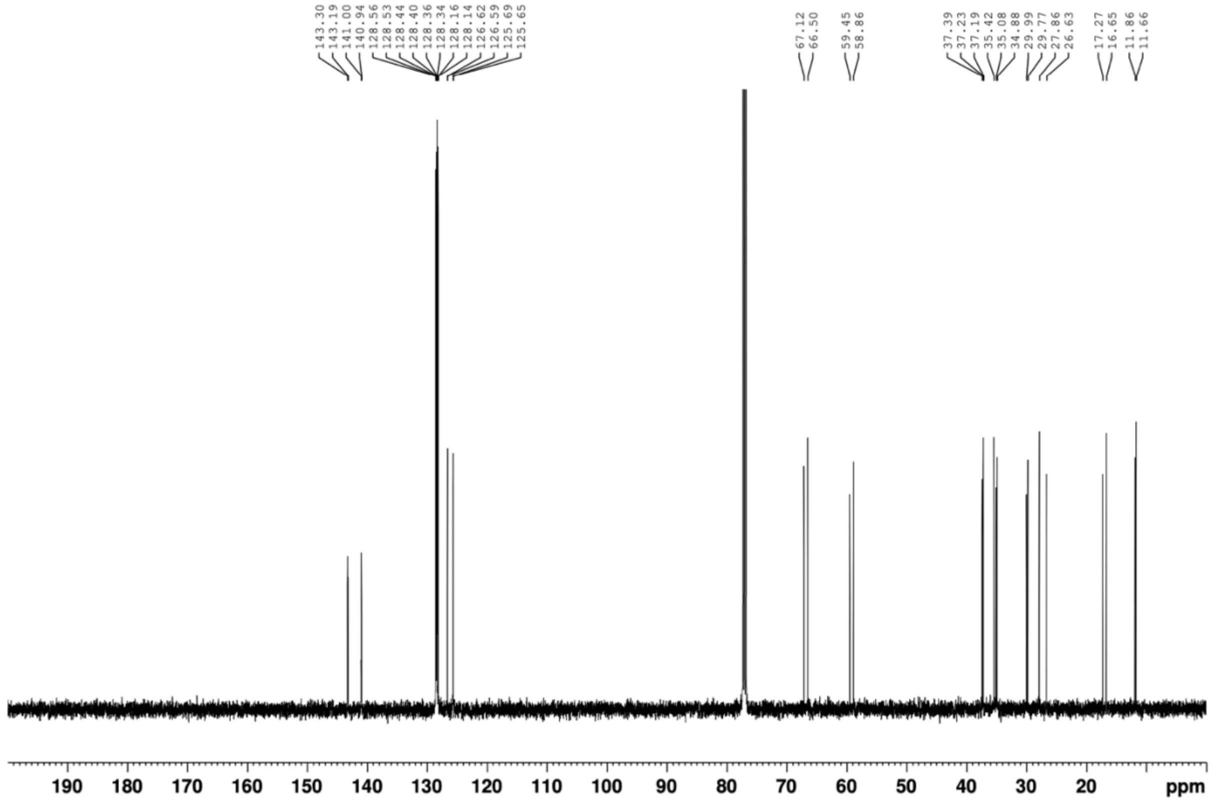

N-Benzyl-1-(4-iodophenyl)-N-methyl-4-phenylbutan-2-amine (10f)

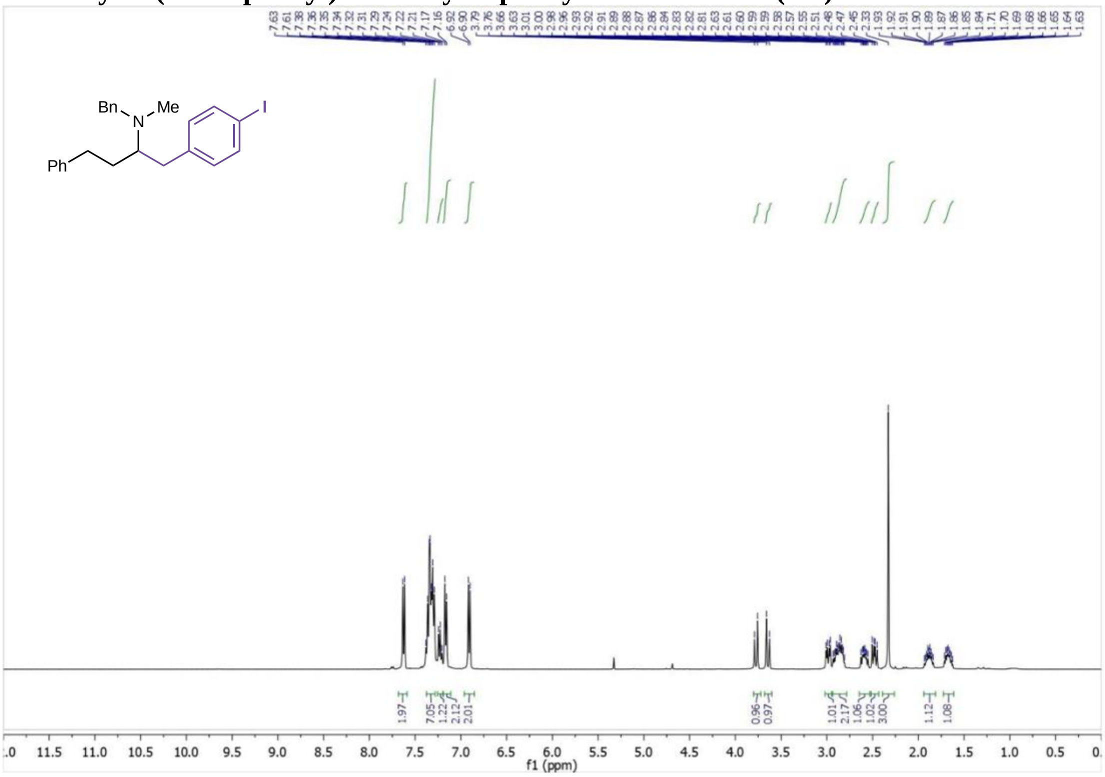

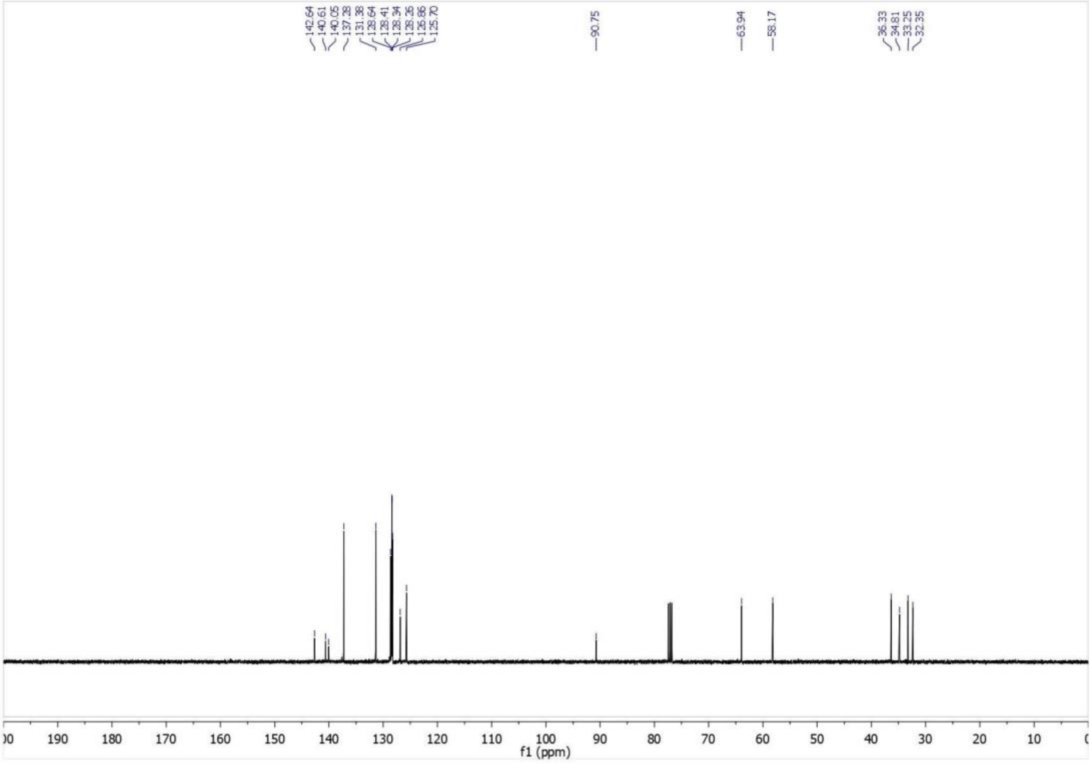

Methyl 3-(2-(benzyl(methyl)amino)-4-phenylbutyl)benzoate (10g)

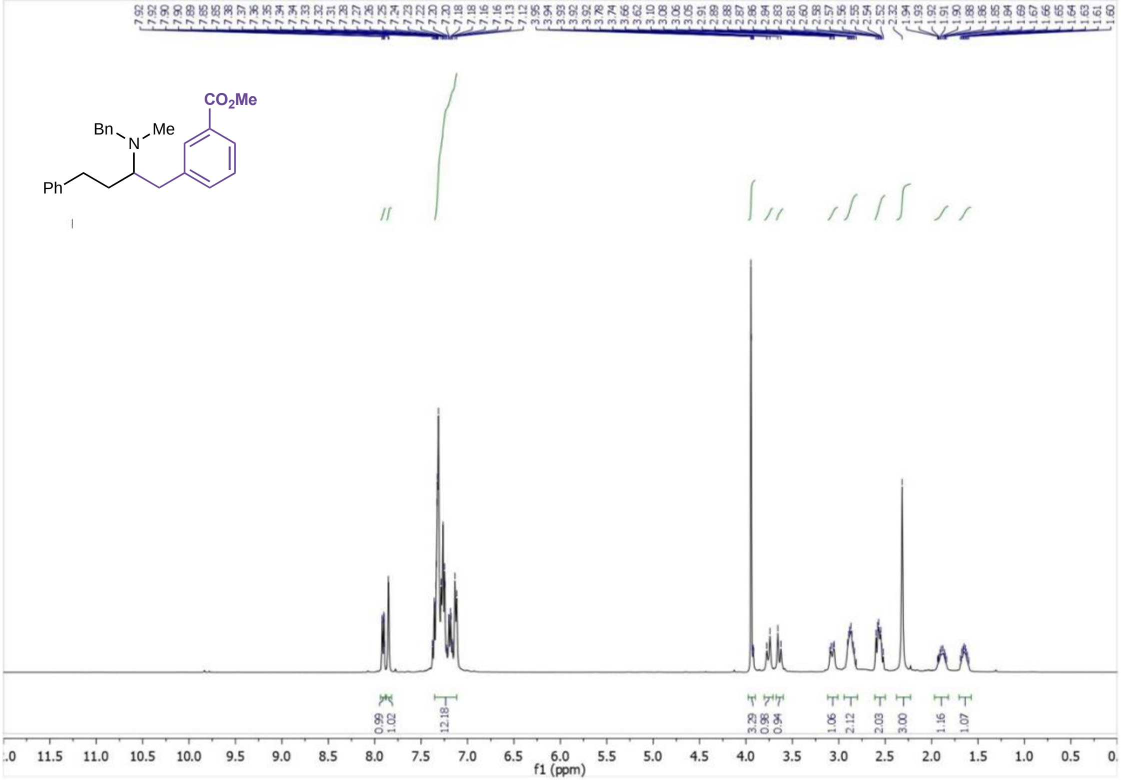

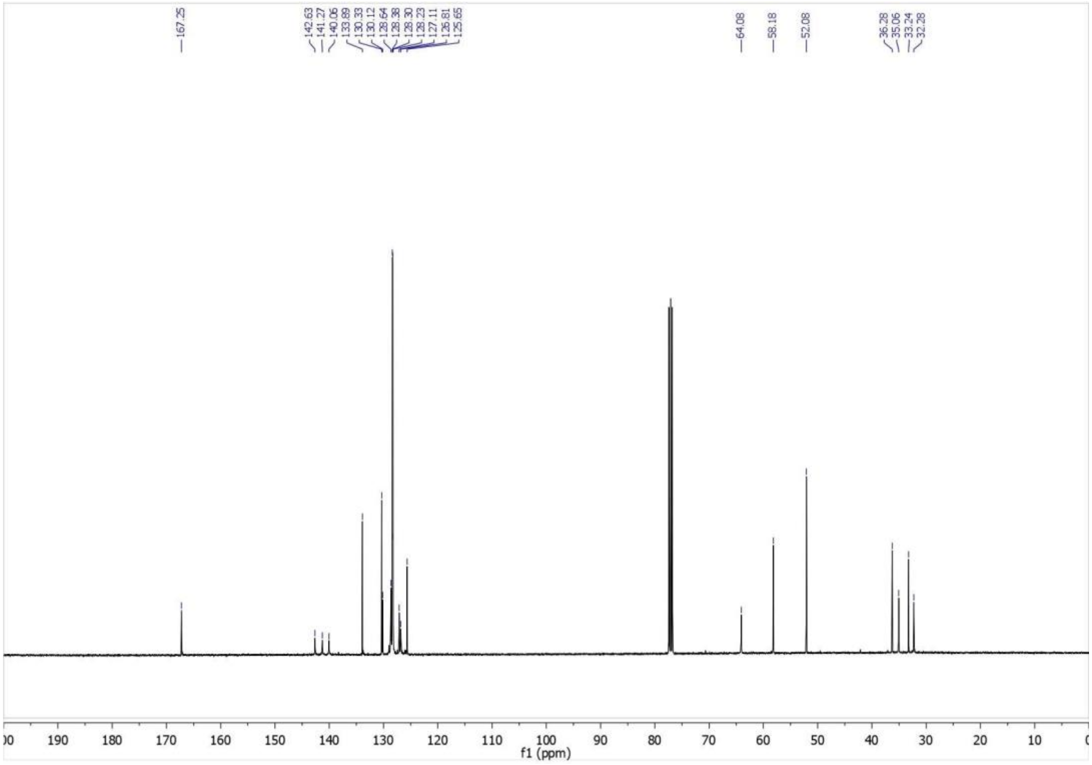

N-benzyl-N-methyl-1,4-diphenylbutan-2-amine (10h)

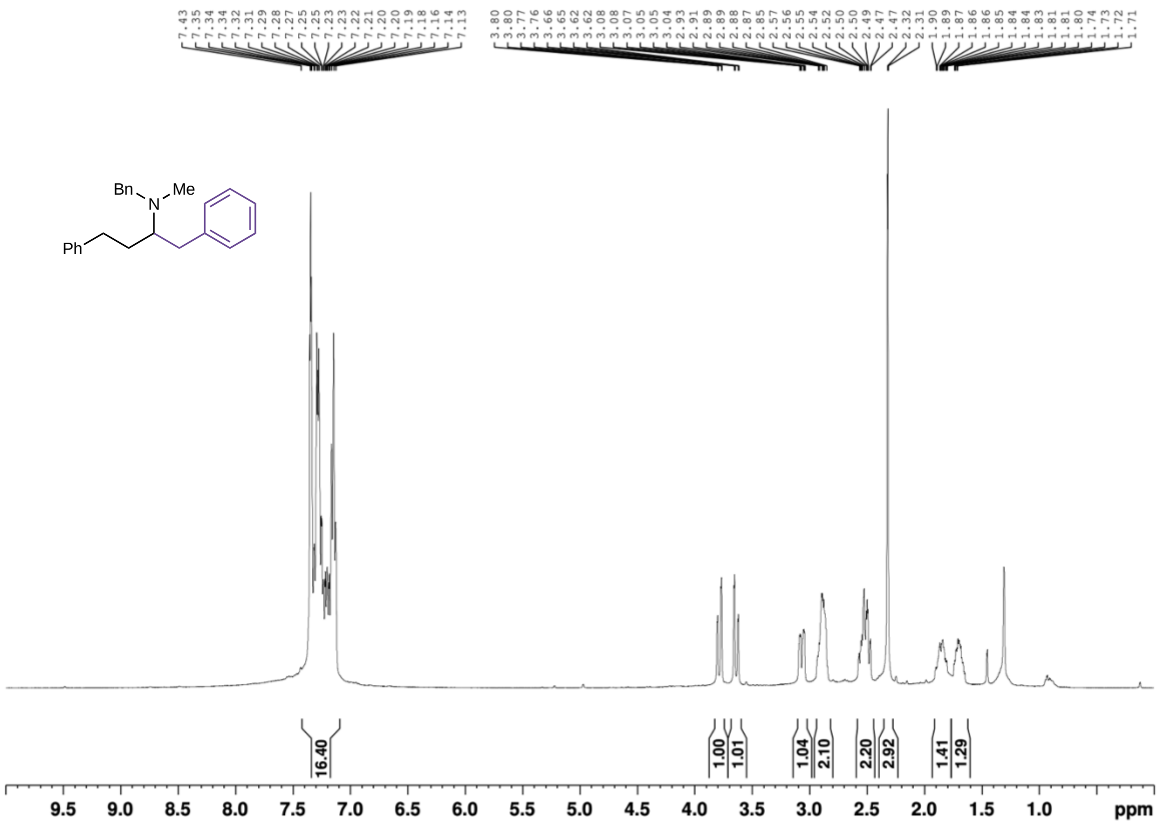

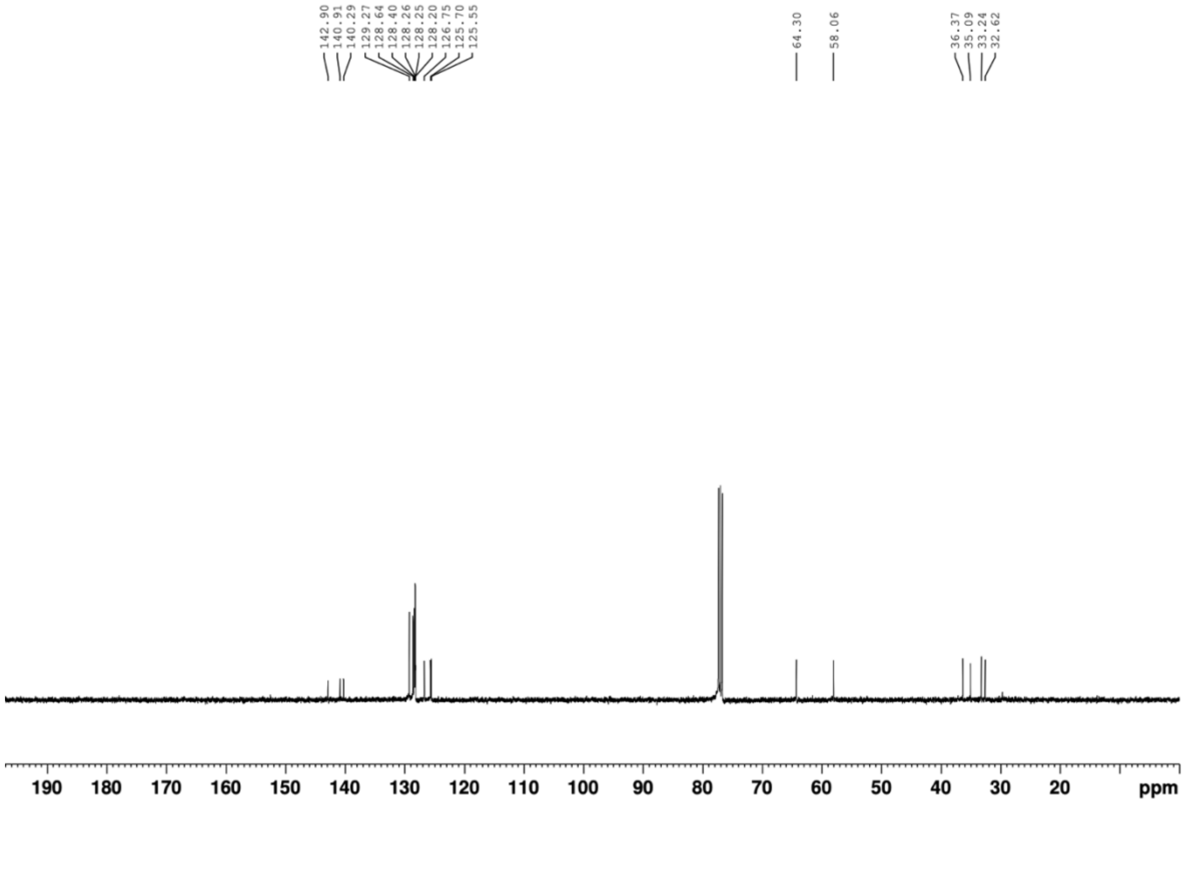

N-benzyl-1-(4-chlorophenyl)-N-methyl-4-phenylbutan-2-amine (10i)

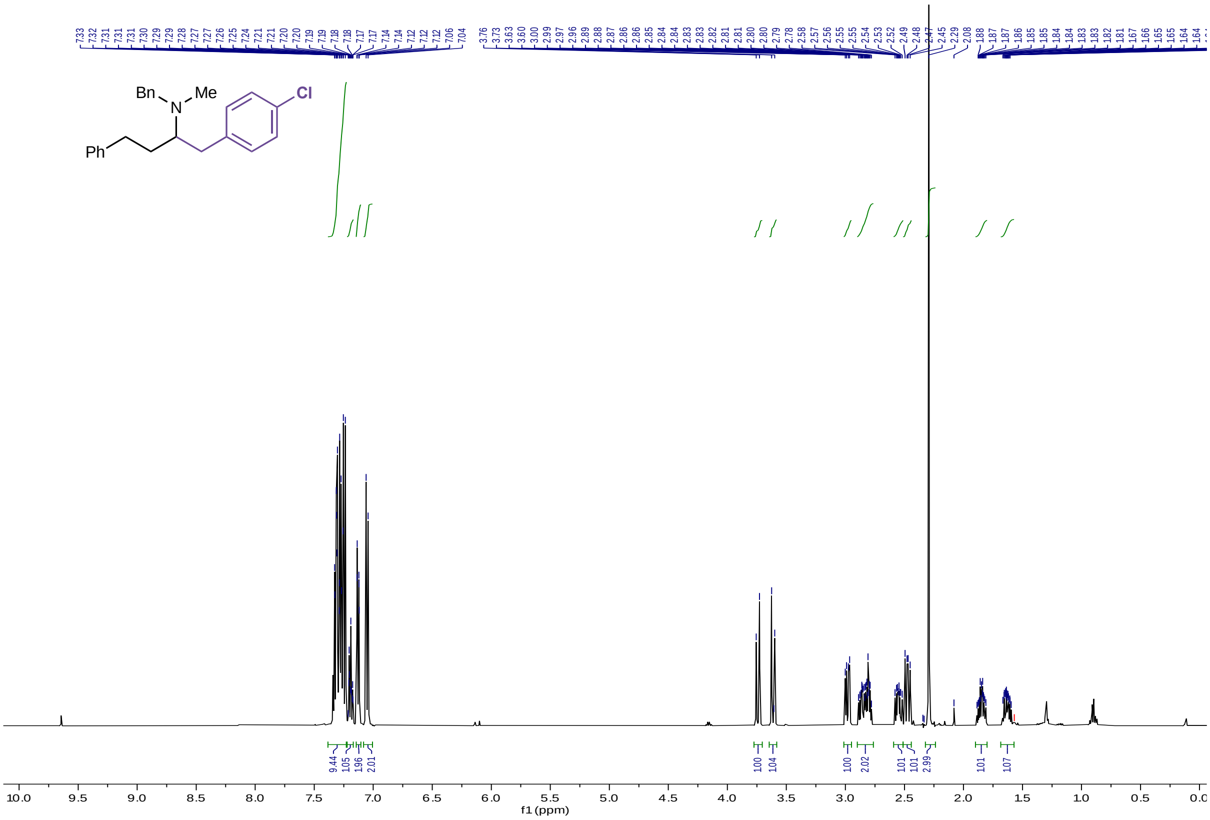

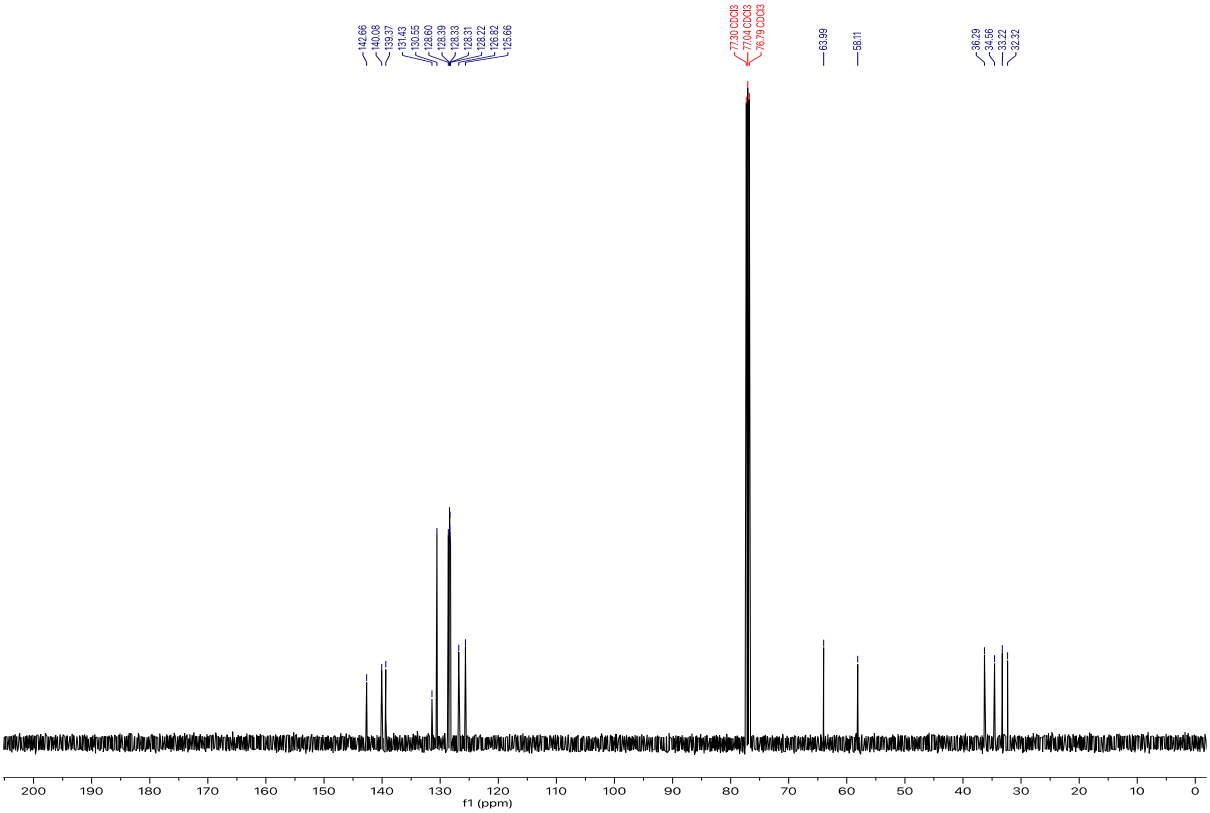

N-benzyl-N-methyl-1,1,4-triphenylbutan-2-amine (10j)

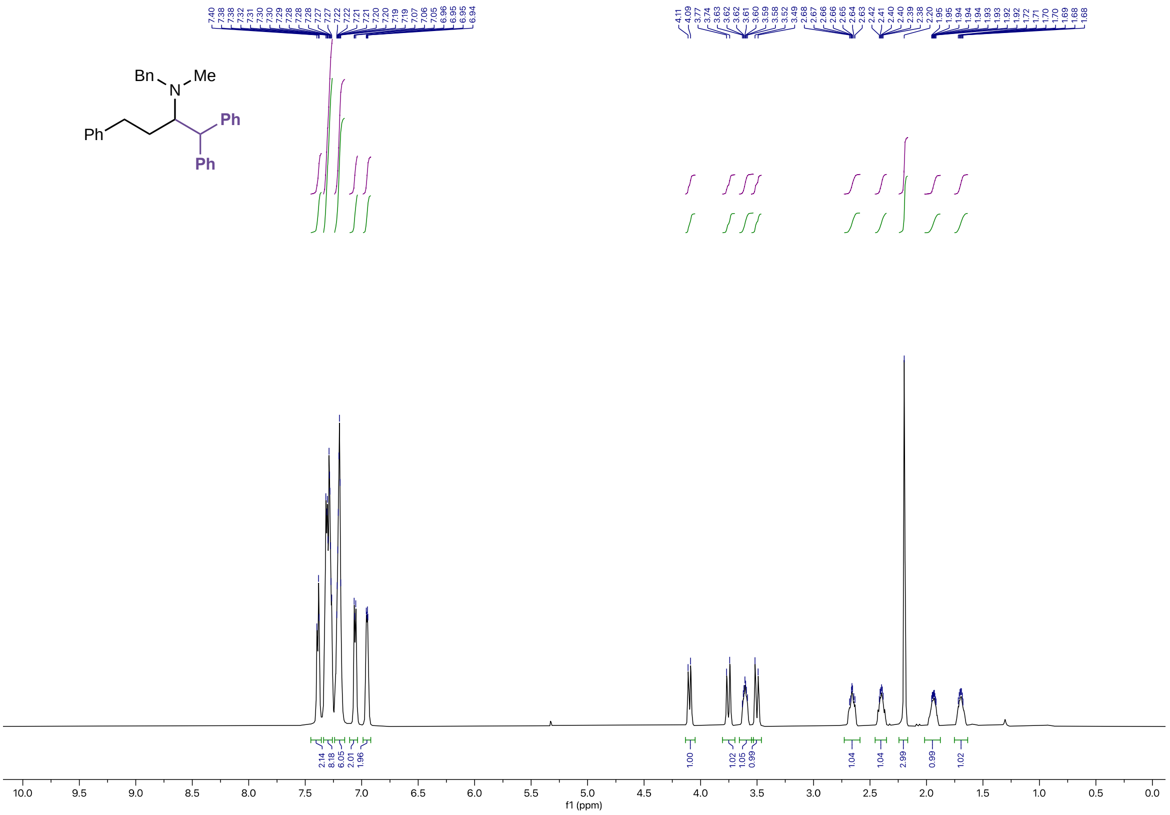

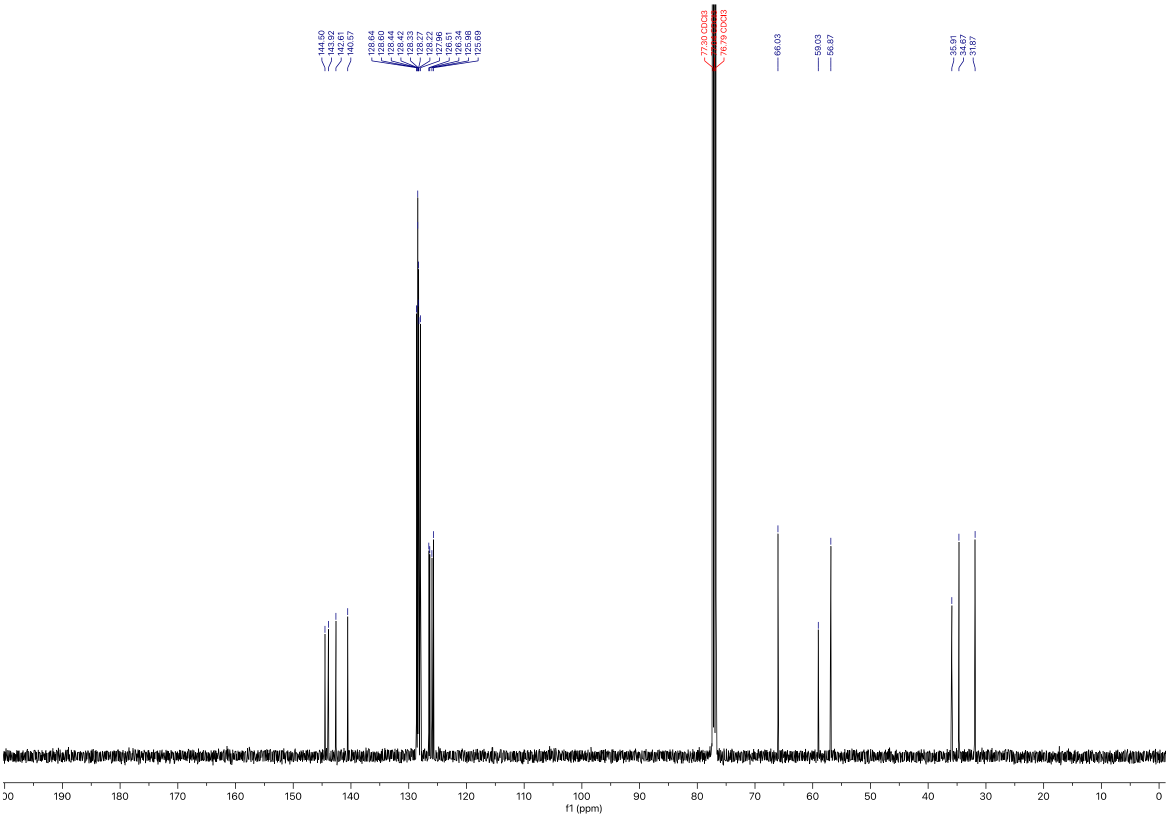

Ethyl 1-(1-(benzyl(methyl)amino)-3-phenylpropyl)cyclobutane-1-carboxylate (10k)

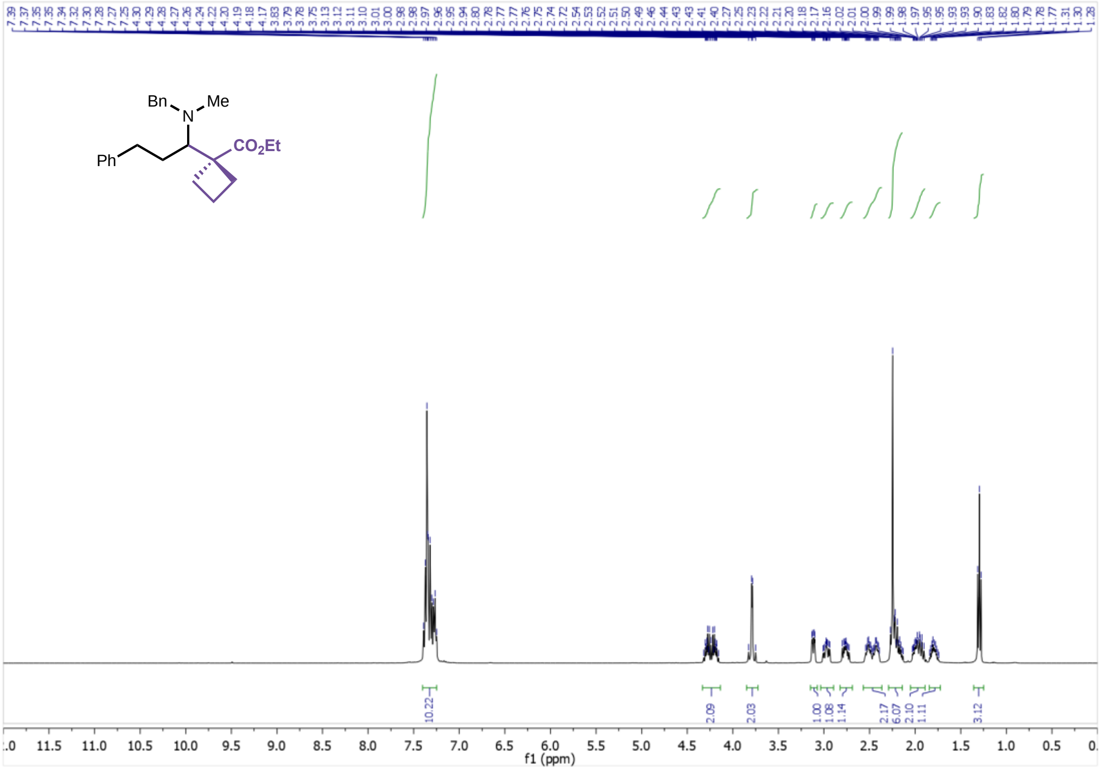

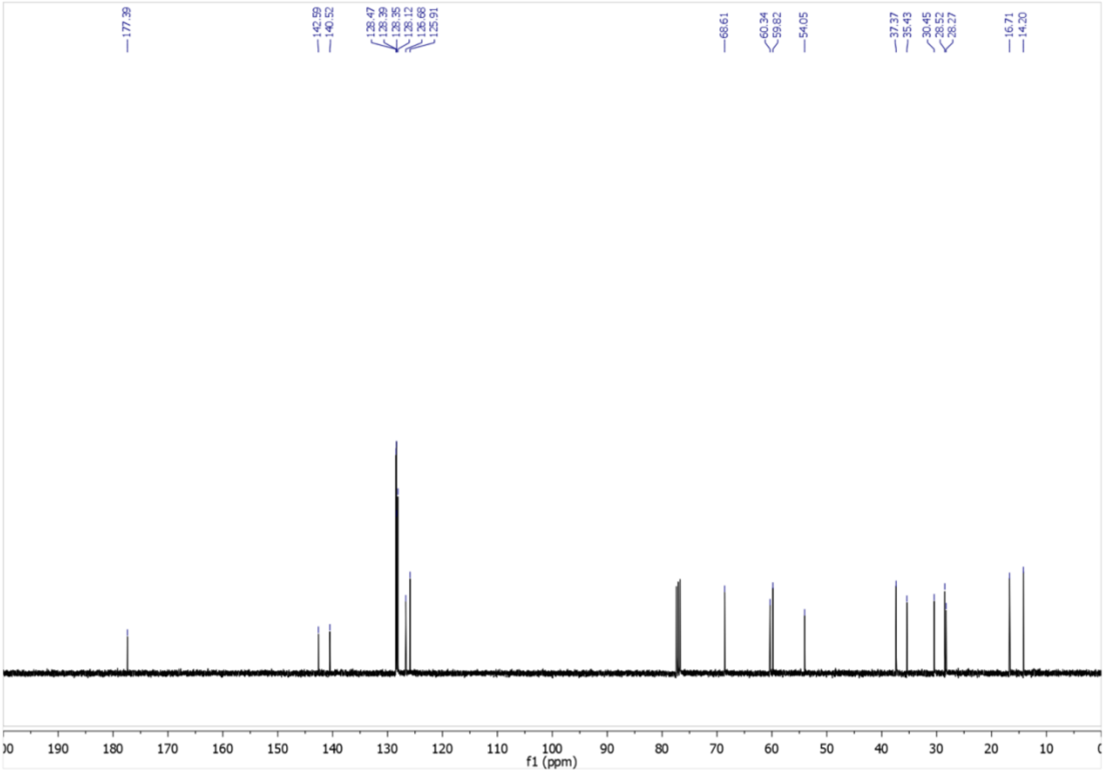

Ethyl 3-(benzyl(methyl)amino)-2,2-difluoro-5-phenylpentanoate (10l)

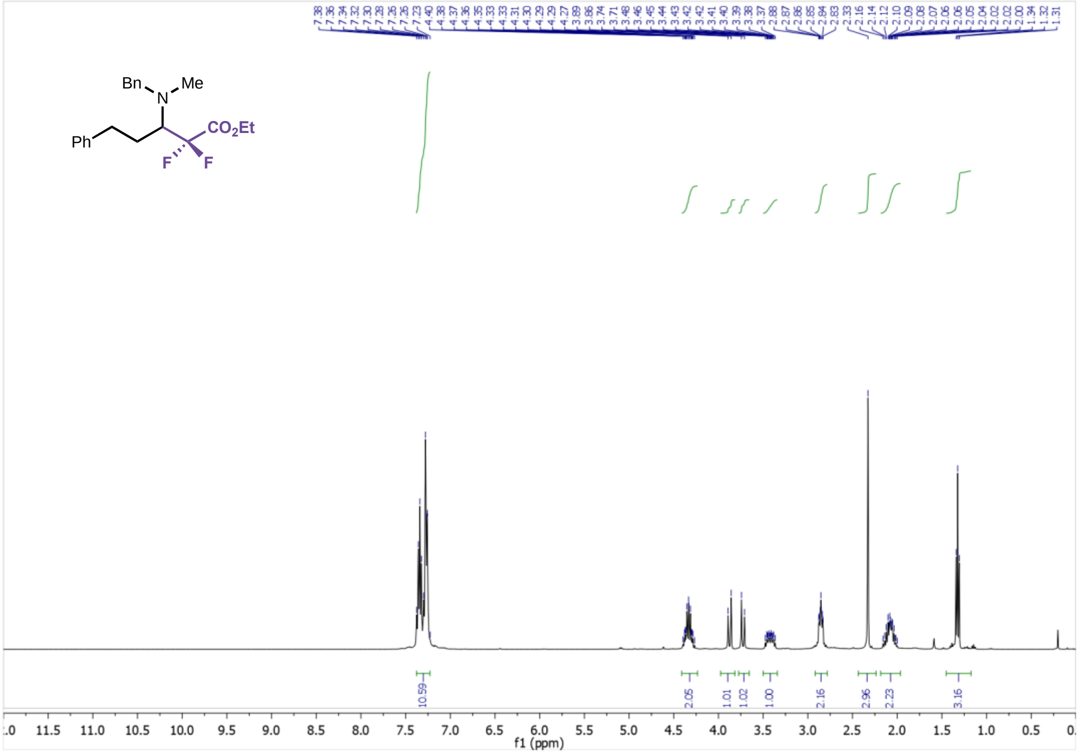

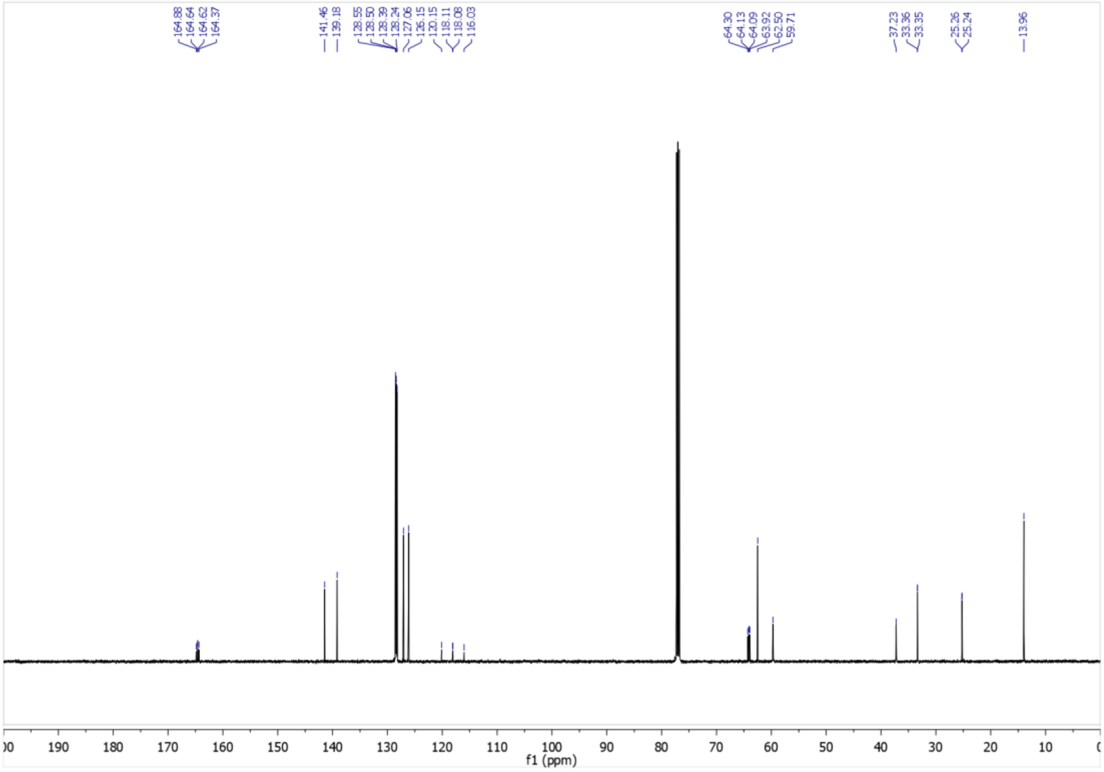

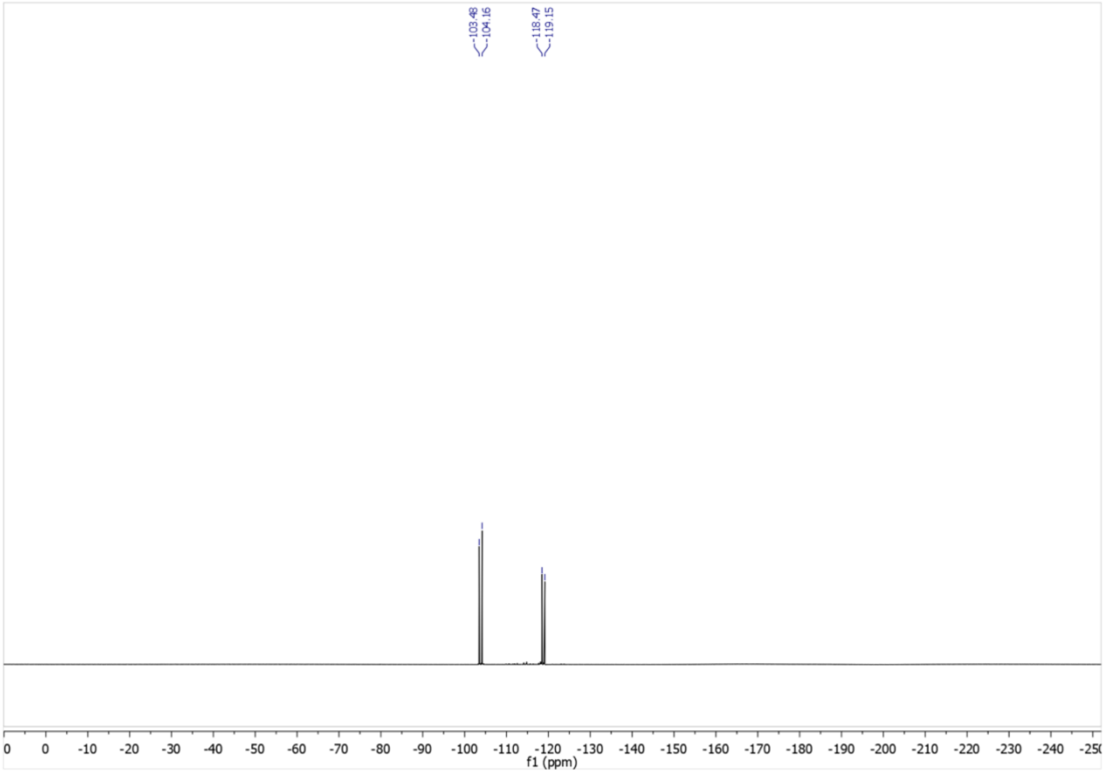
-103.48
-104.16
-118.47
-119.15
0 0 -10 -20 -30 -40 -50 -60 -70 -80 -90 -100 -110 -120 -130 -140 -150 -160 -170 -180 -190 -200 -210 -220 -230 -240 -250
f1 (ppm)

N,N-Dibenzyl-1-phenylbutan-3-amine (11a)

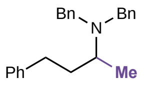

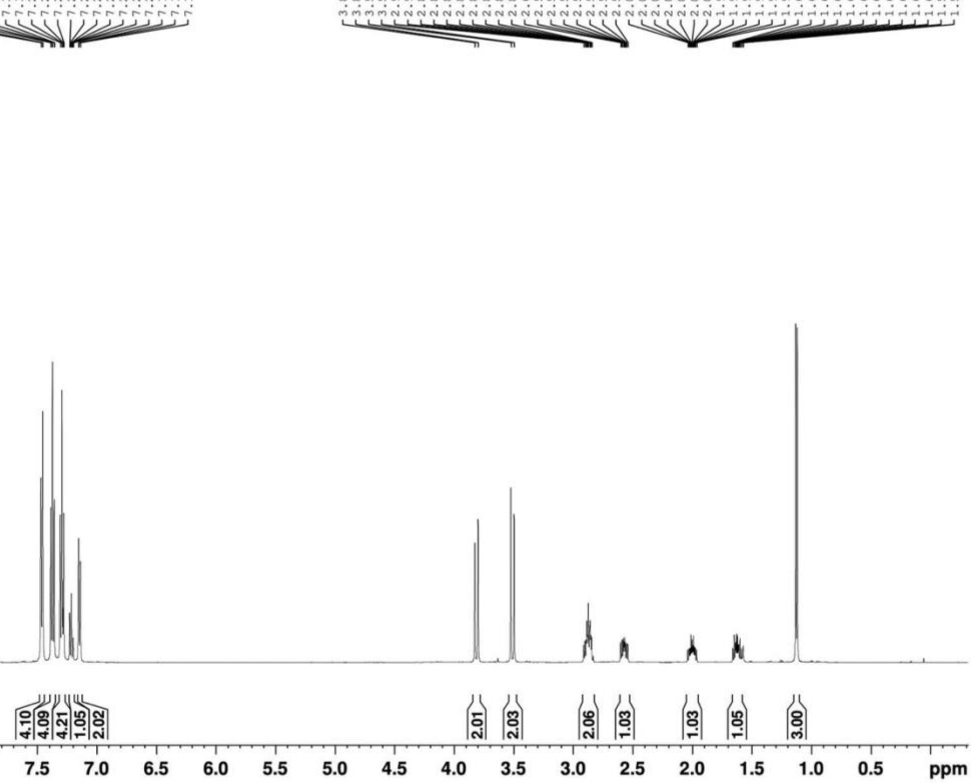

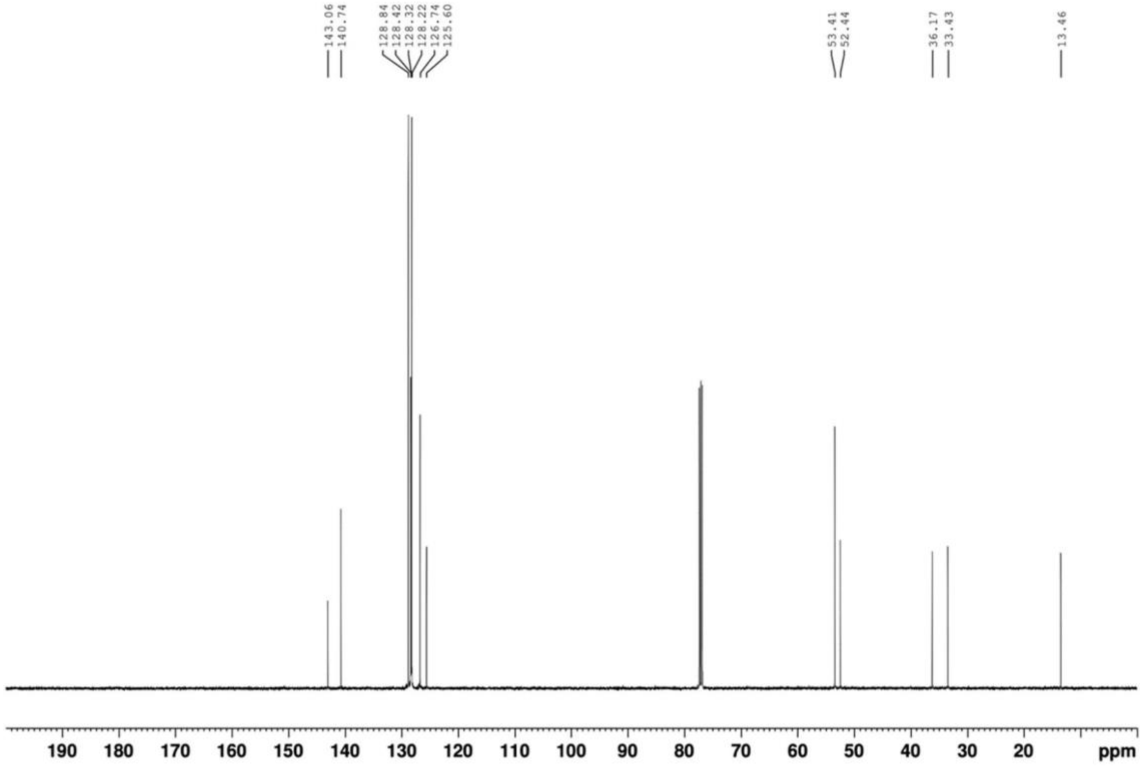

1-N-Cbz-4-(4-phenylbutan-2-yl)piperazine (11b)

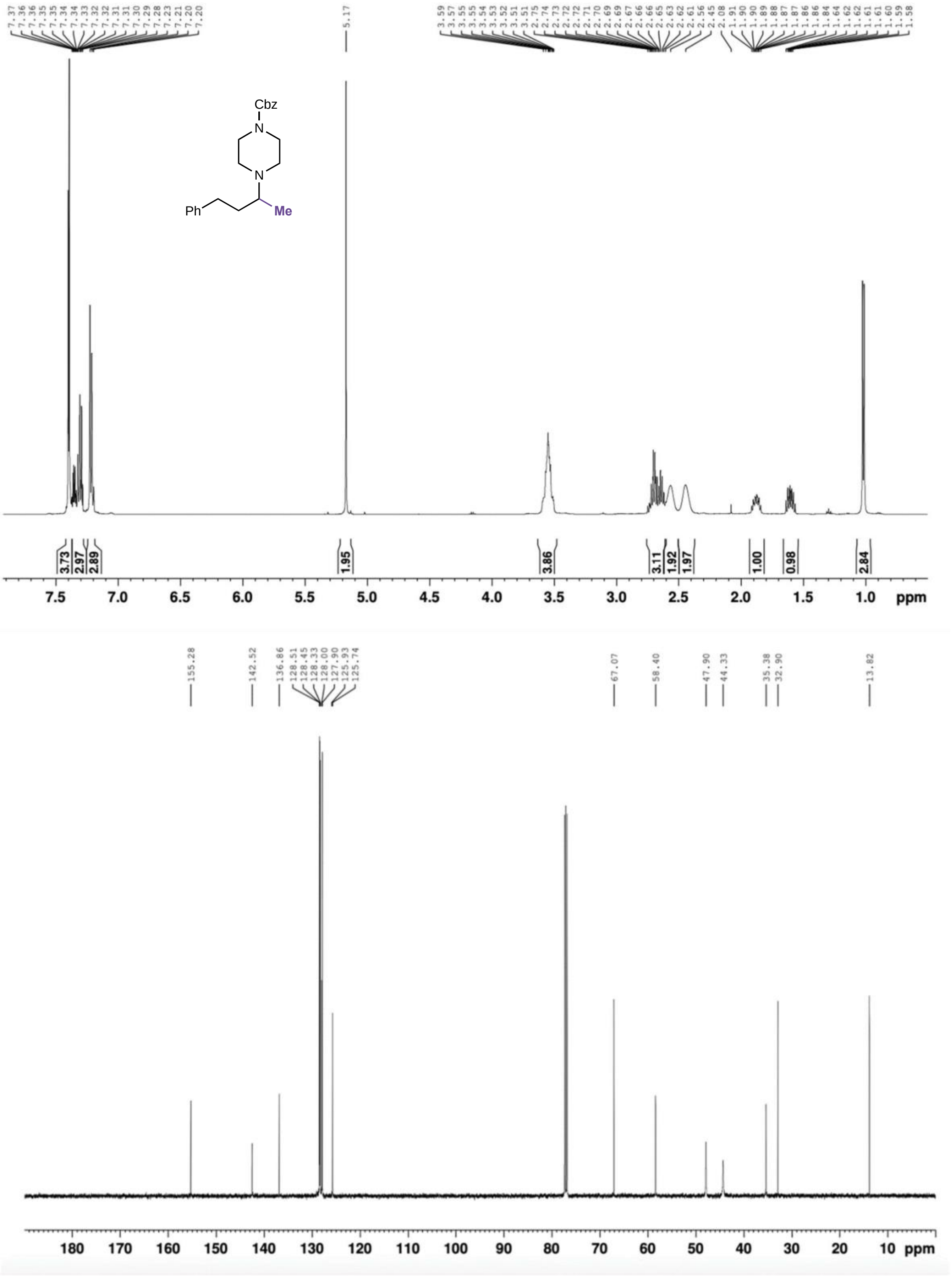

N-benzyl-1-fluoro-N-methyl-5-phenylpentan-3-amine (11d)

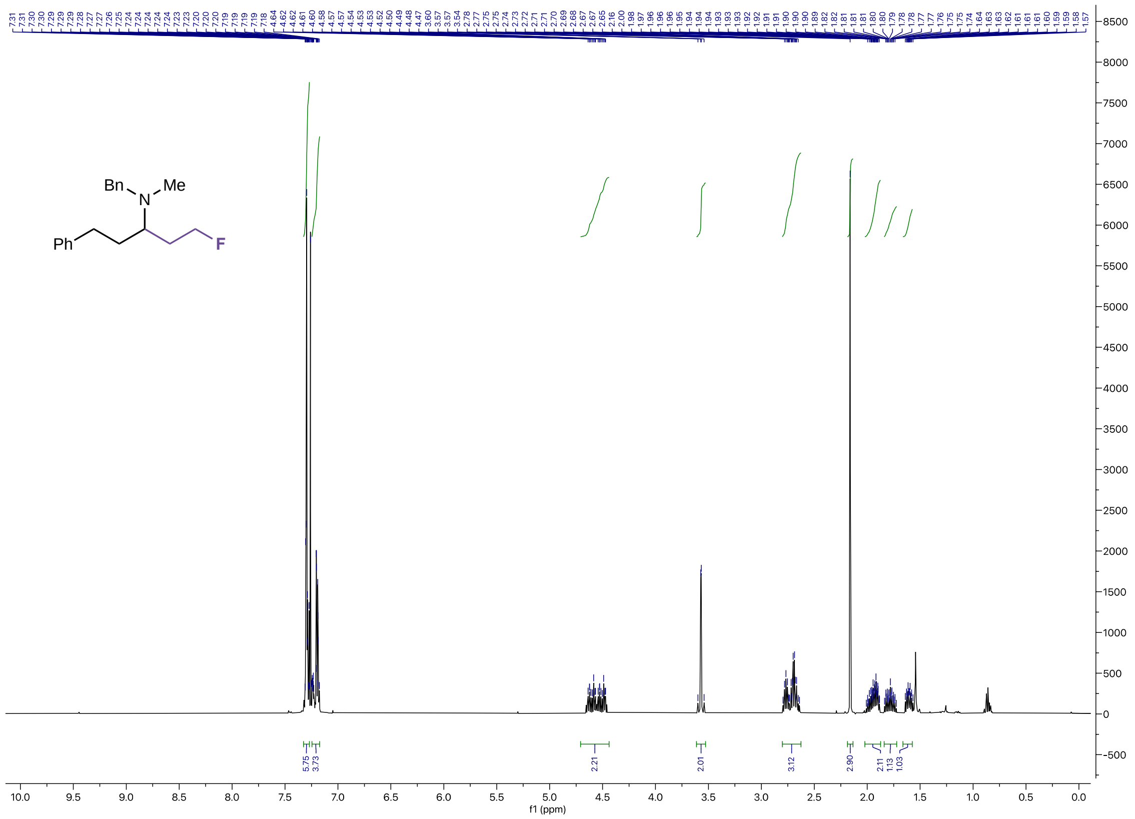

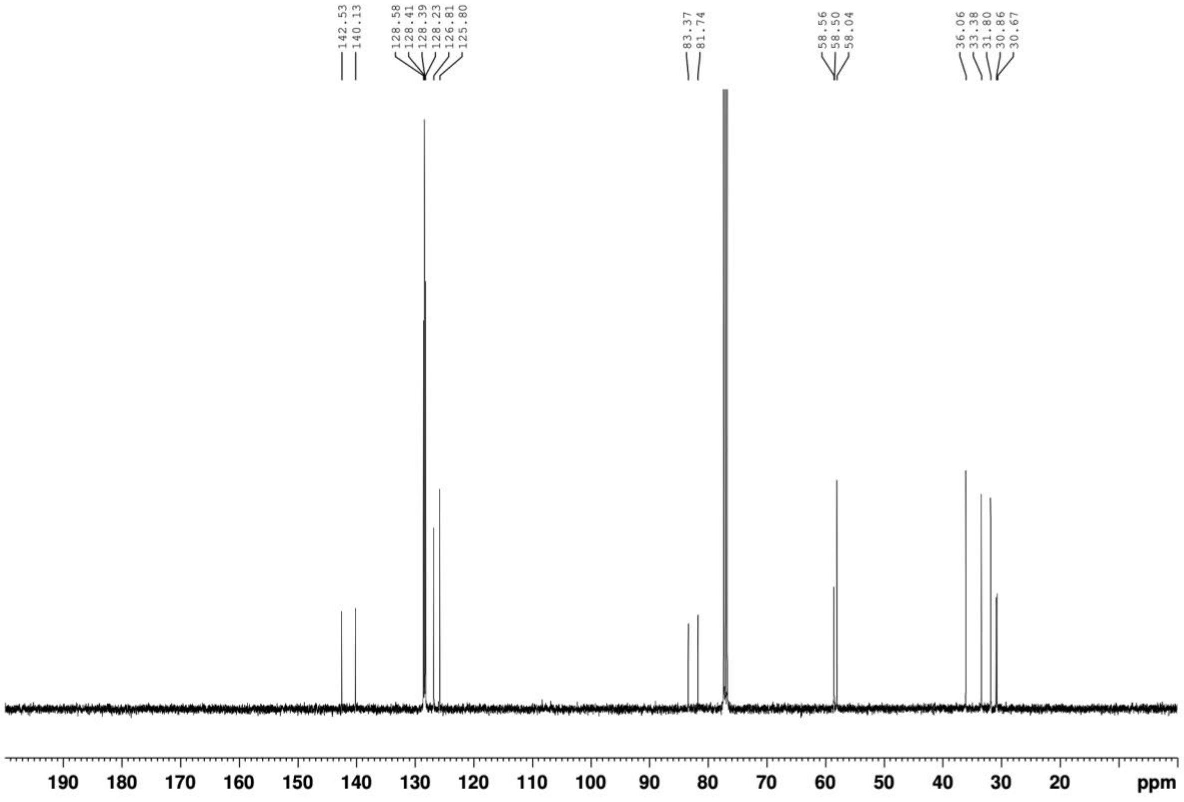

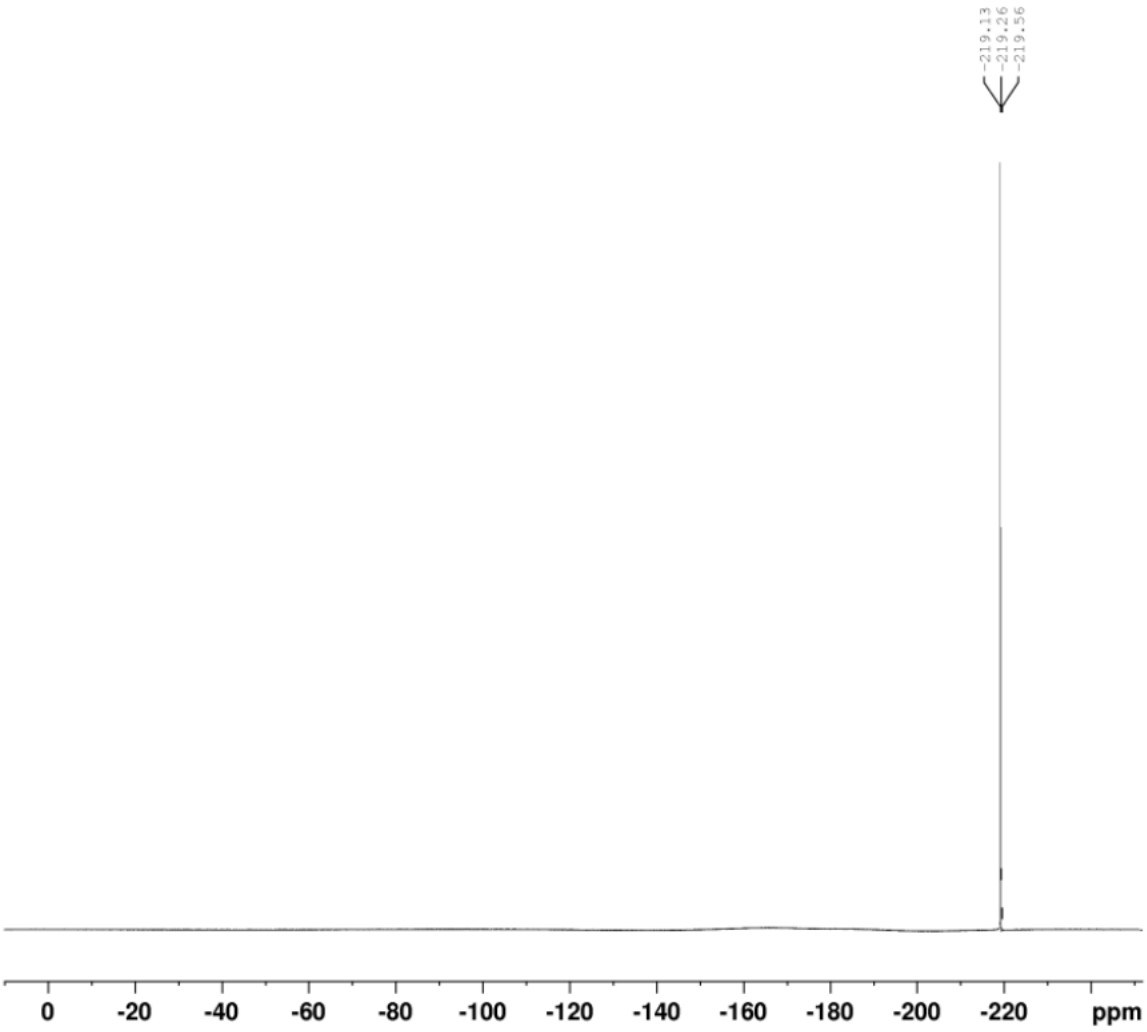

-219.13
-219.26
-219.56
0
-20
-40
-60
-80
-100
-120
-140
-160
-180
-200
-220
ppm

N-Benzyl-N-methyl-1-phenylhexan-3-amine (11e)

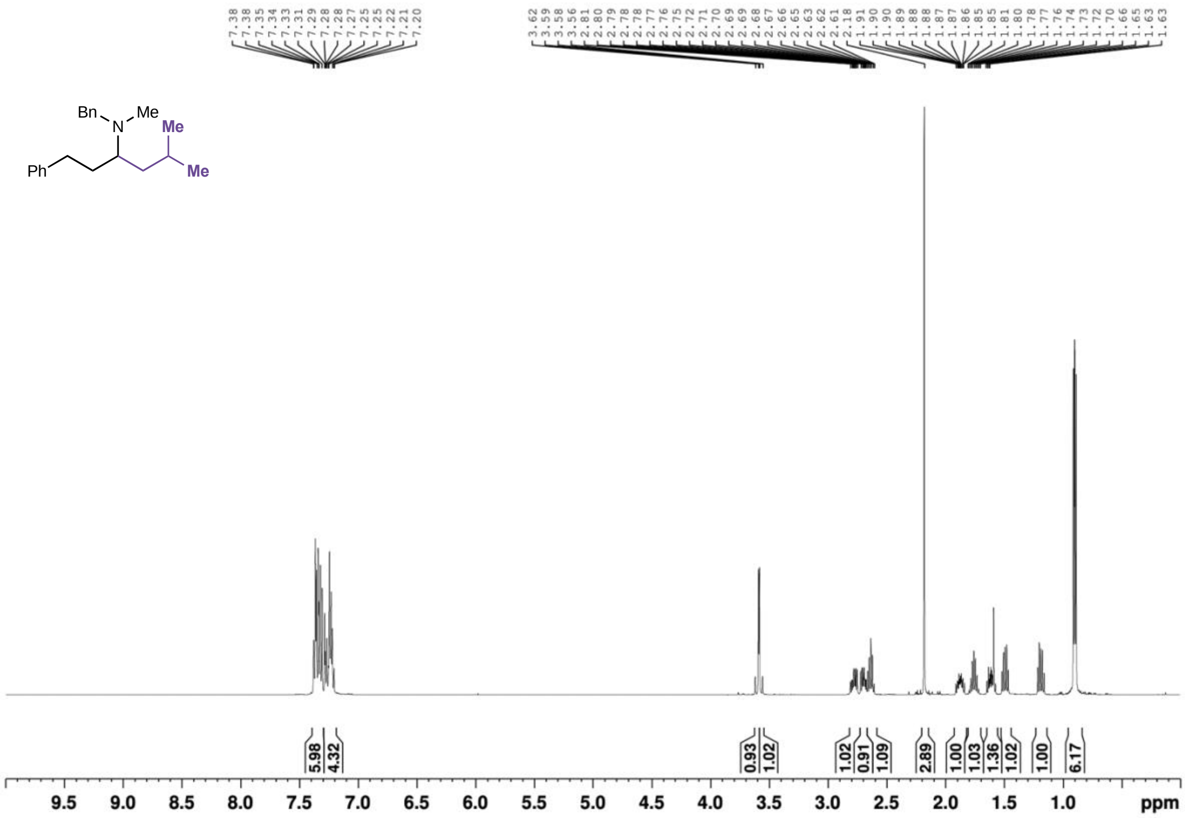

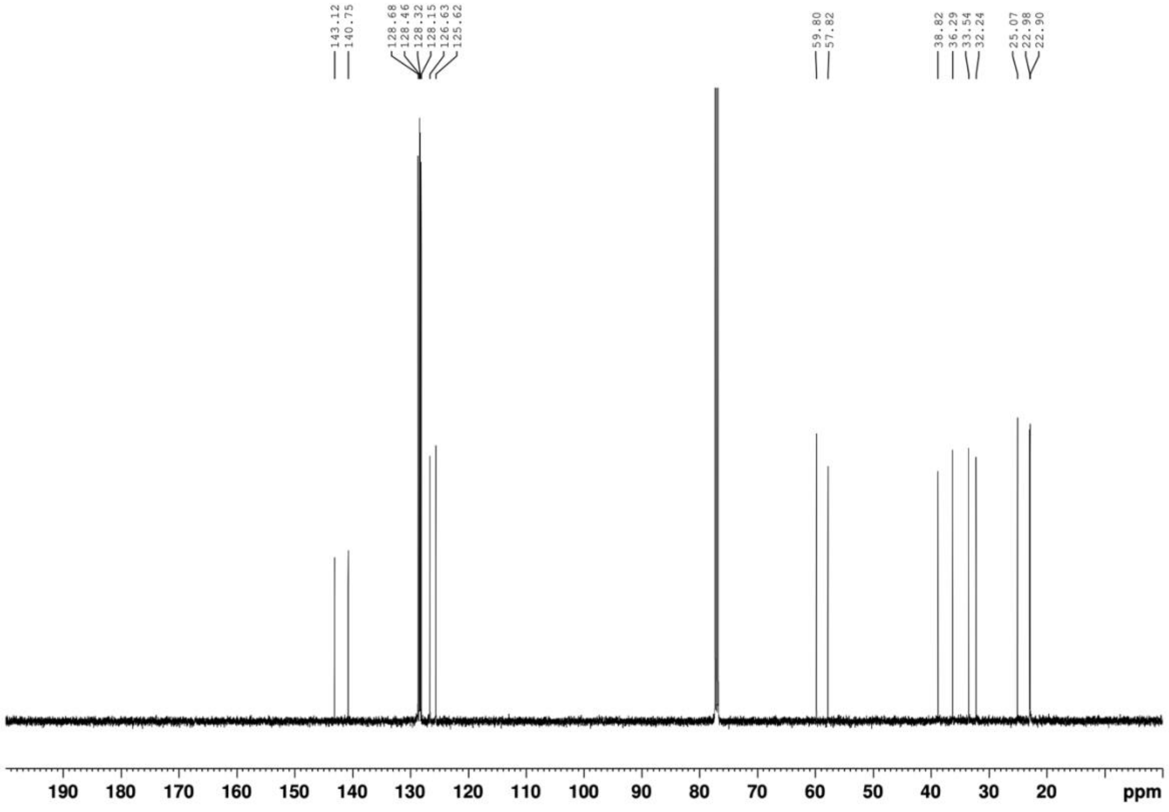

N-benzyl-N-methyl-1-phenylpentan-3-amine-4,4,5,5,5-d_5 (11f)

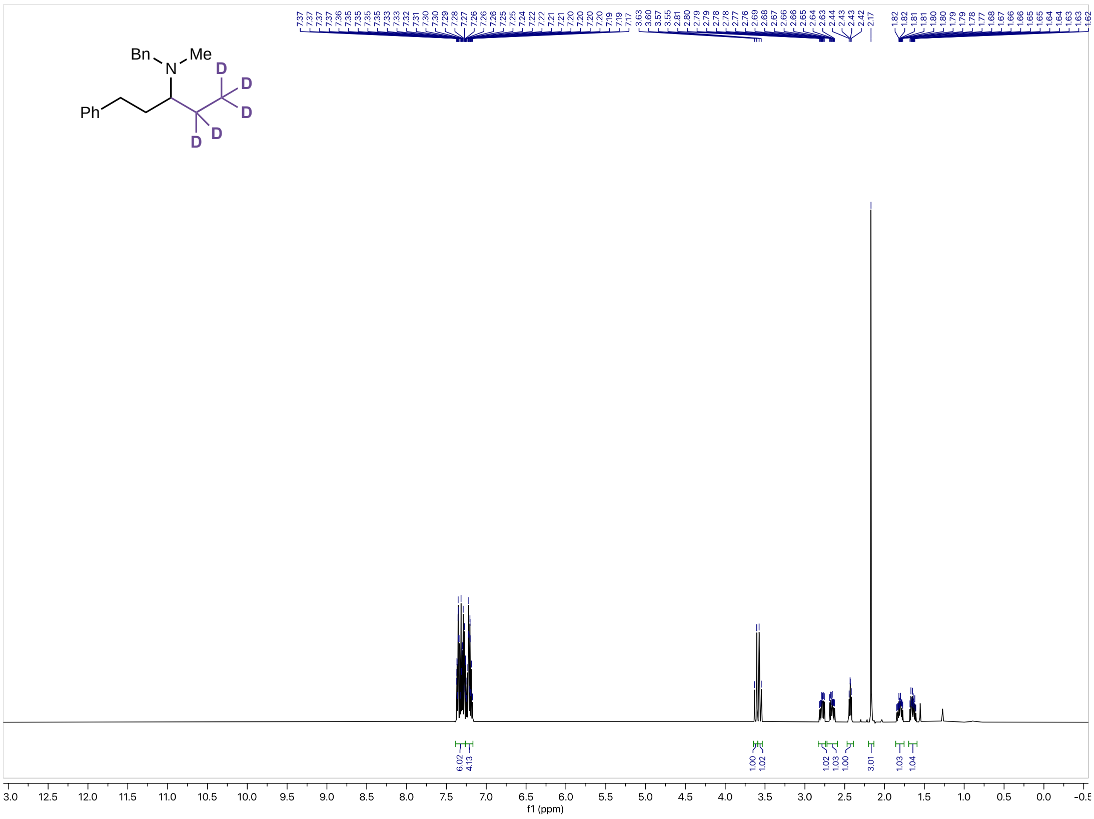

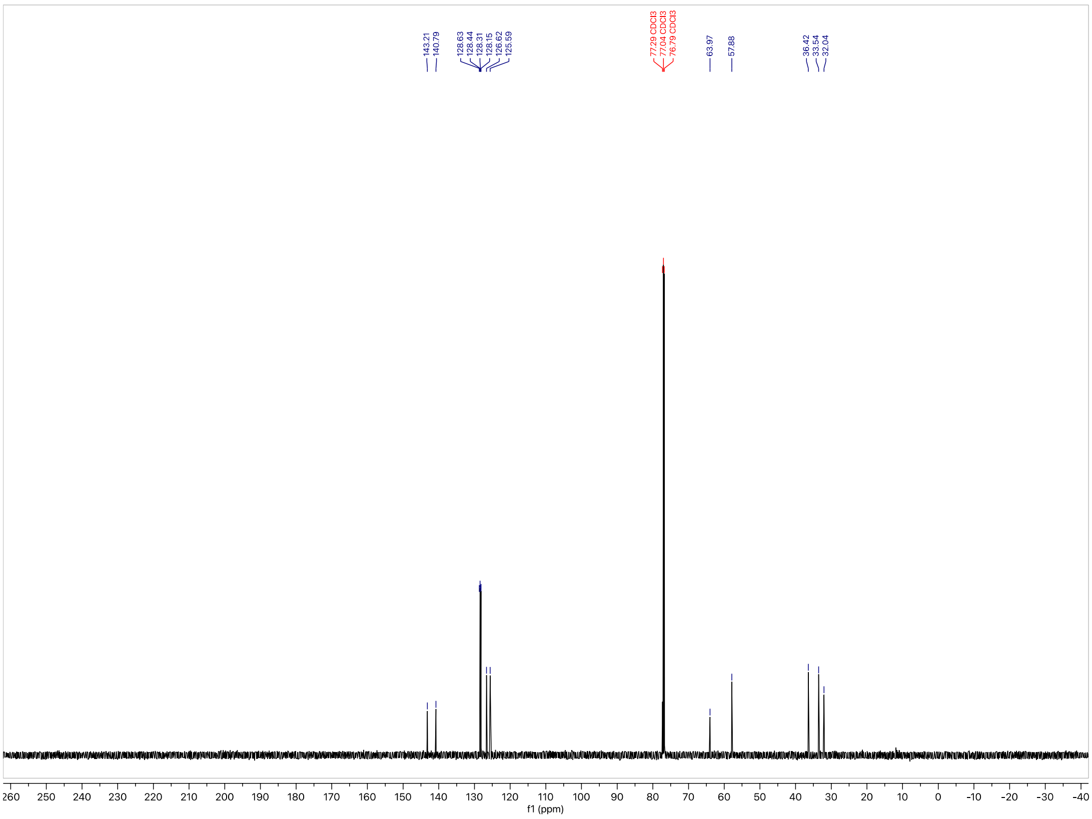

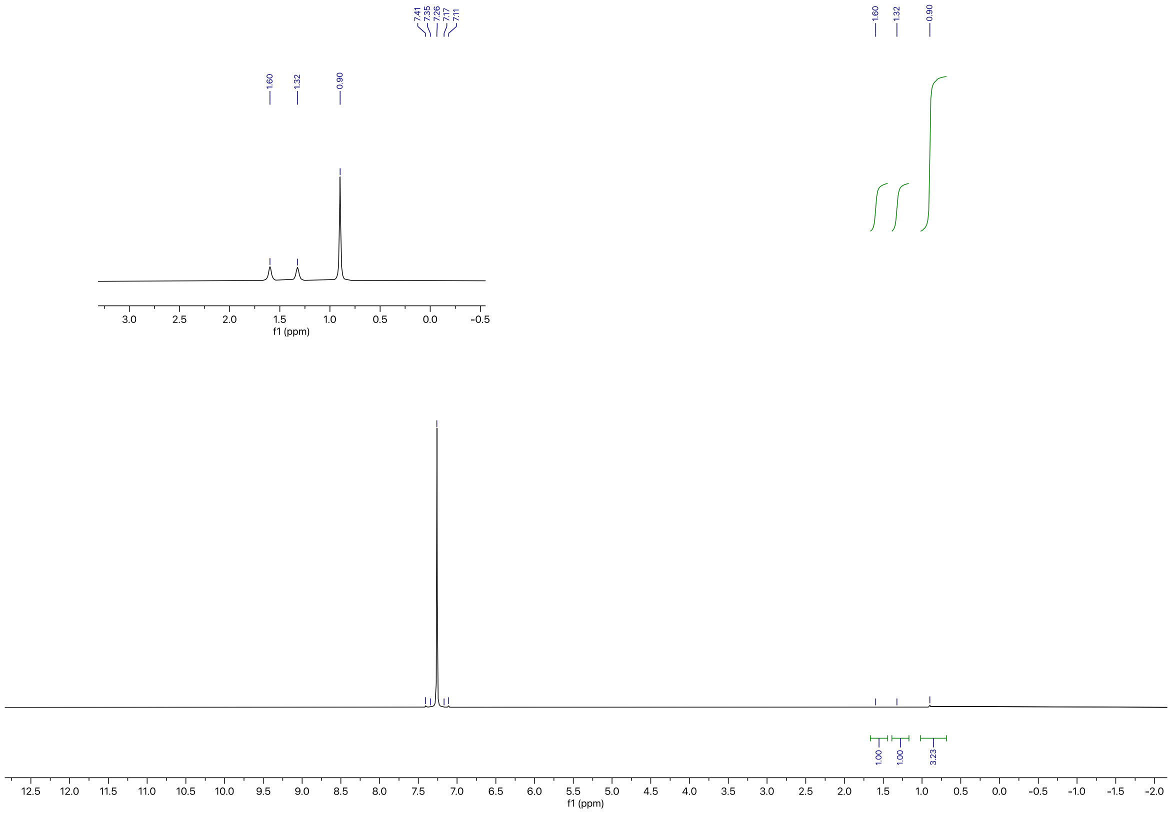

7.41
7.35
7.26
7.17
7.11
1.60
1.32
0.90
1.00
1.00
3.23
f1 (ppm)

N-benzyl-7-chloro-N-methyl-1-phenylheptan-3-amine (11g)

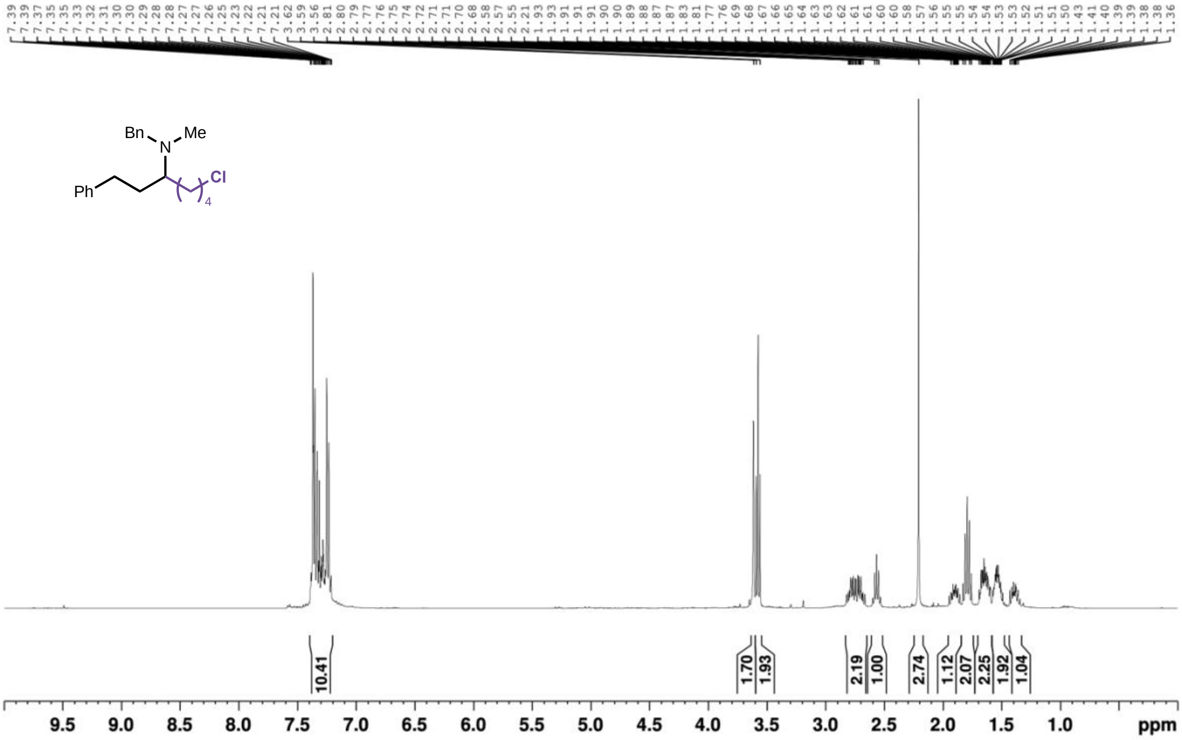

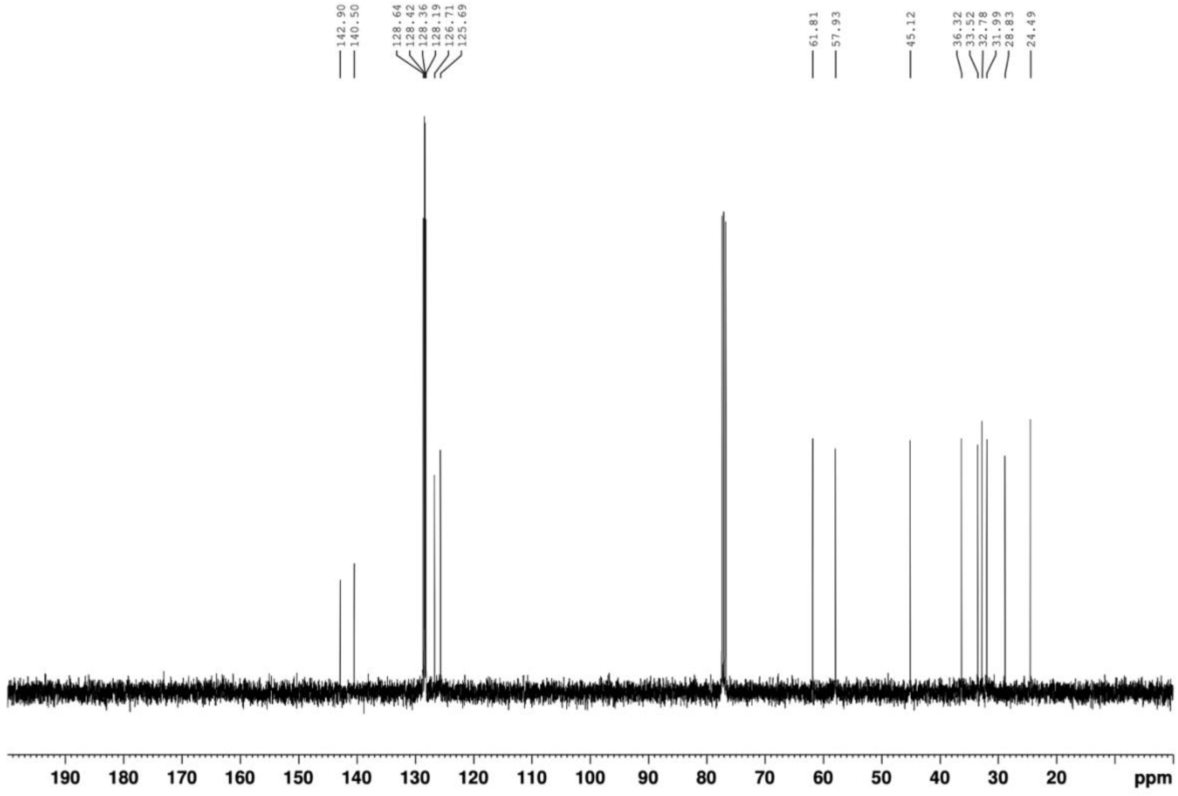

1-(1-phenylpentan-2-yl)pyrrolidine (Prolitane) (11h)

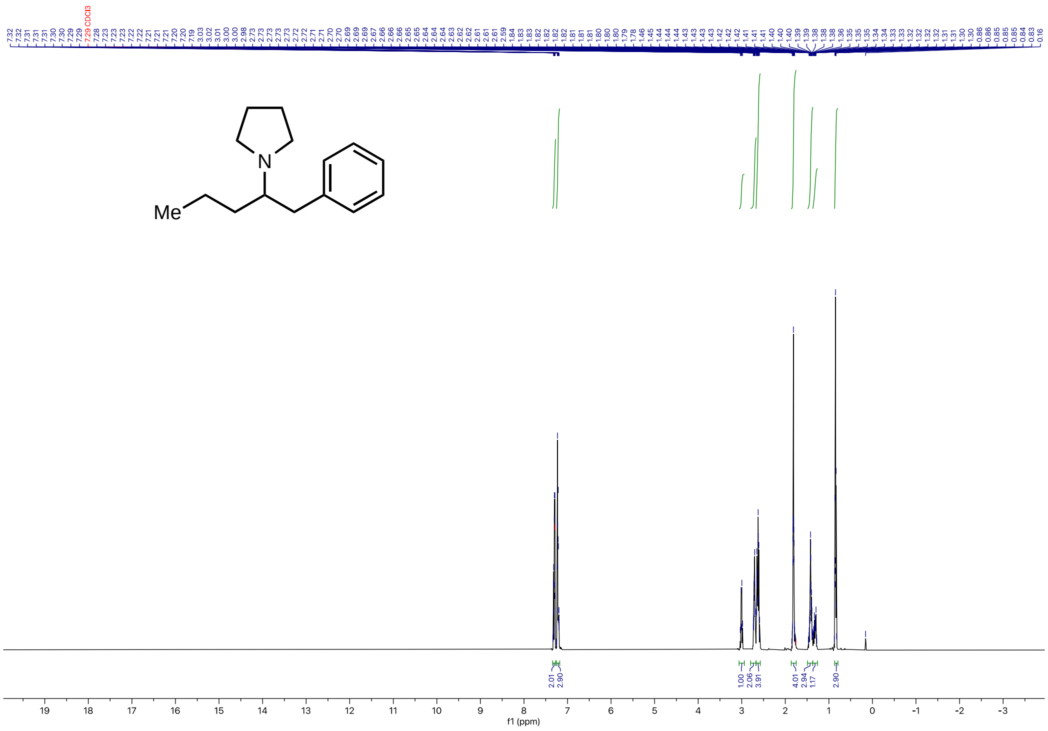

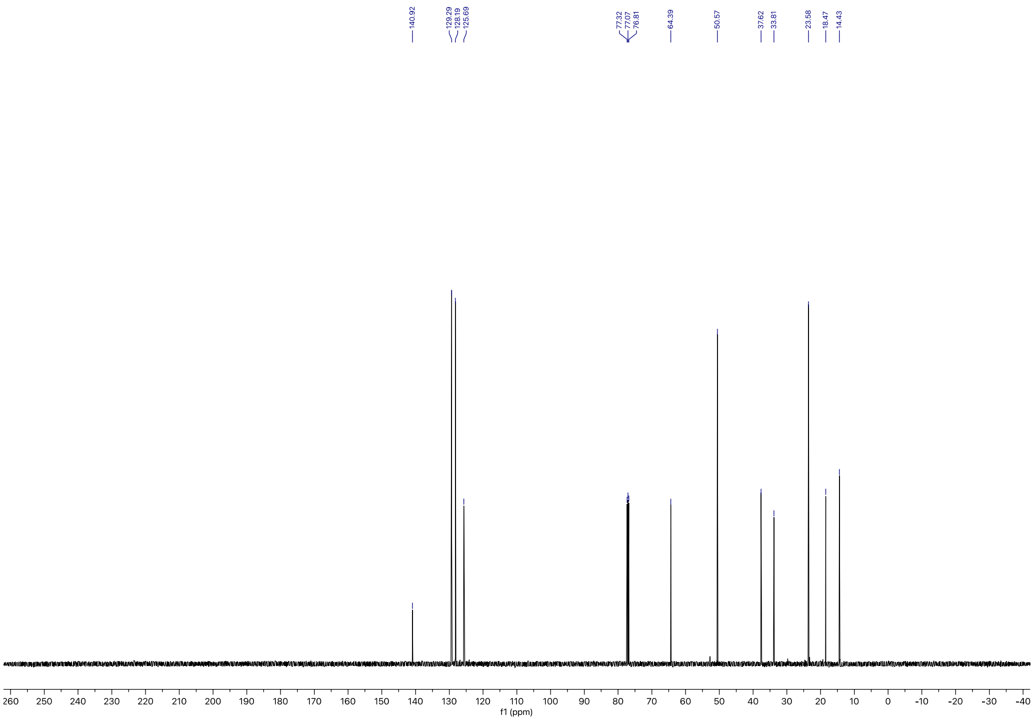

N-Benzyl-N-methyl-1-phenyl-2methylpropan-1-amine (12a)

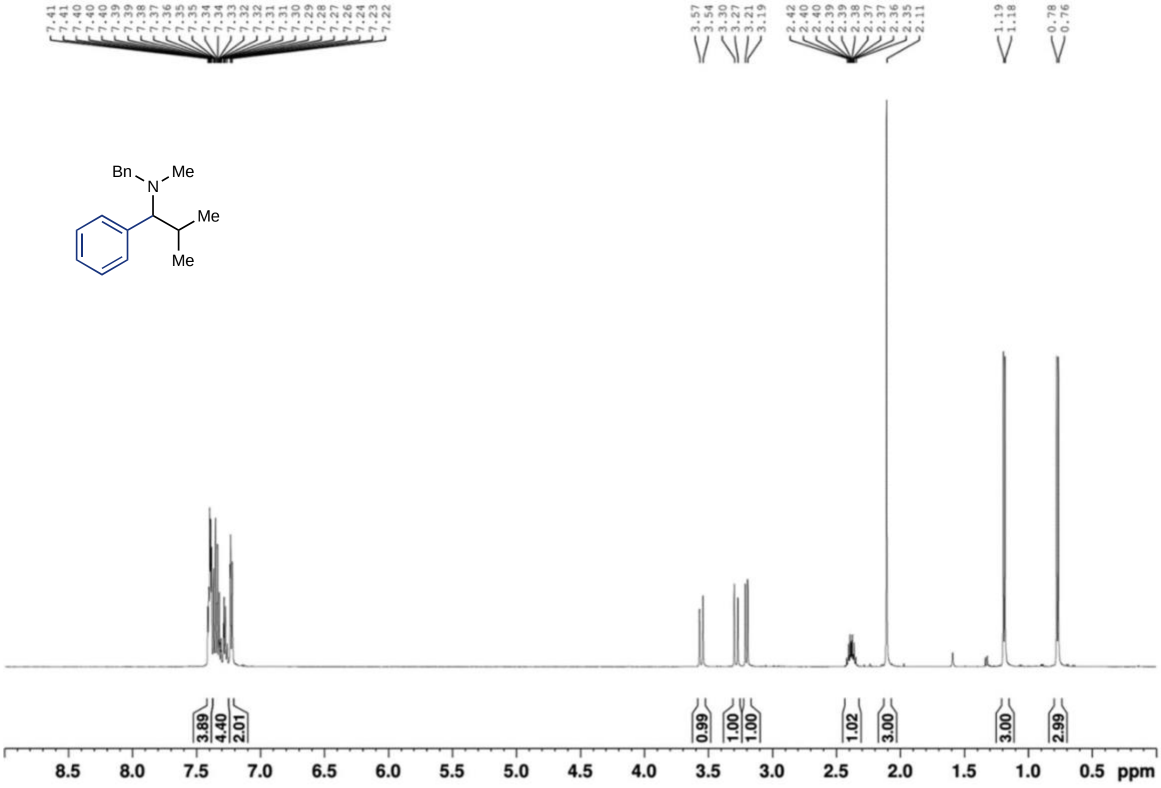

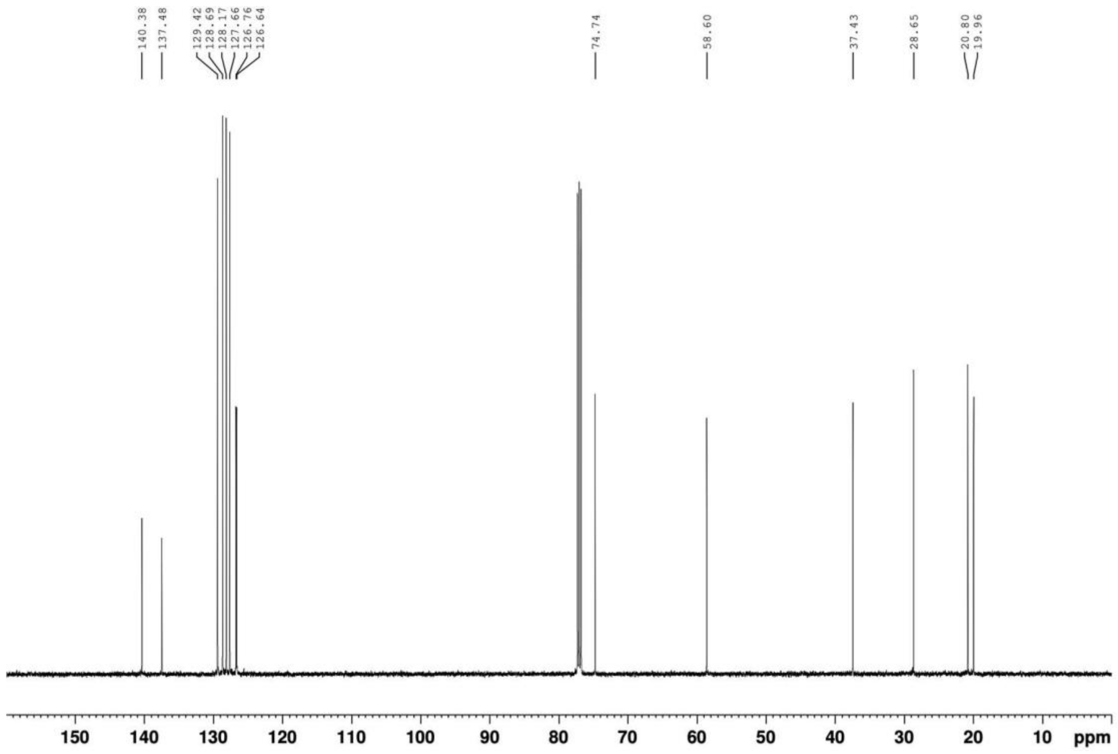

methyl 4-(1-(benzyl(methyl)amino)-2-methylpropyl)benzoate (12b)

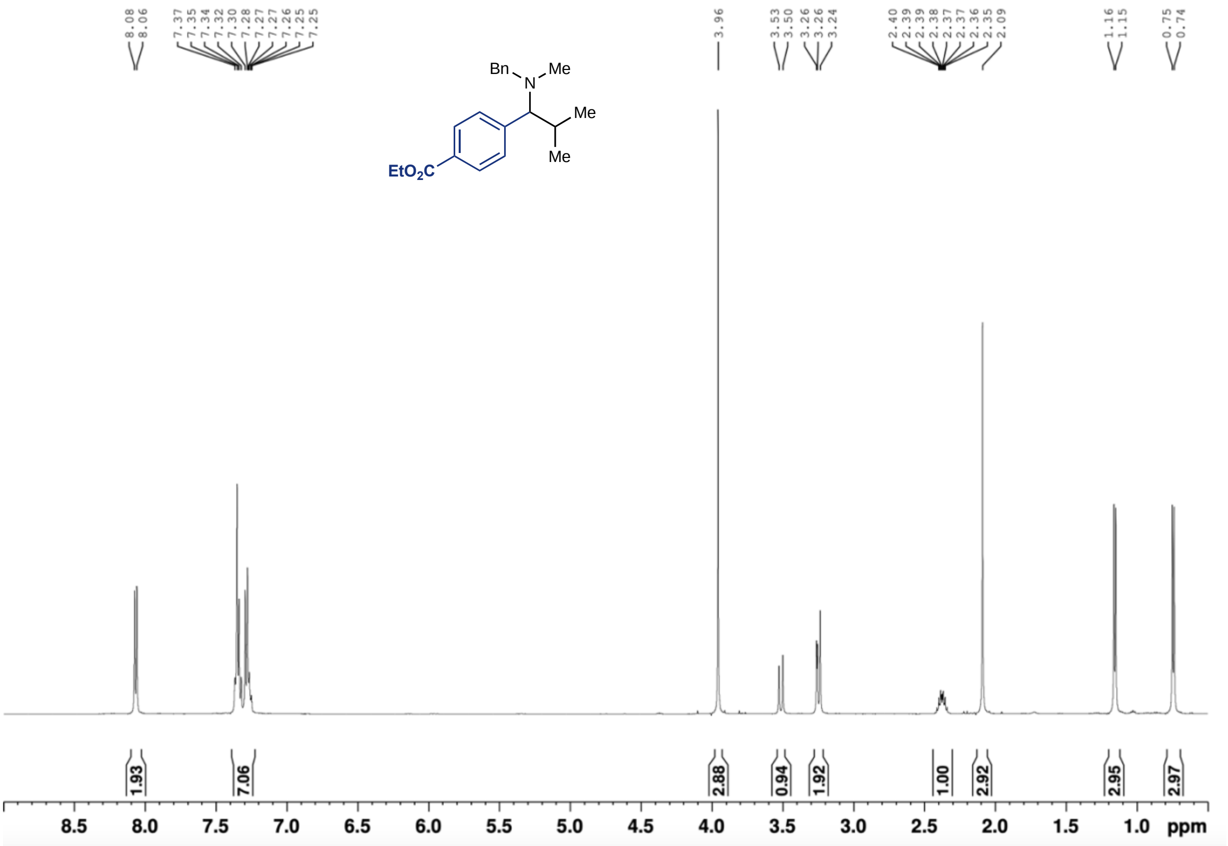

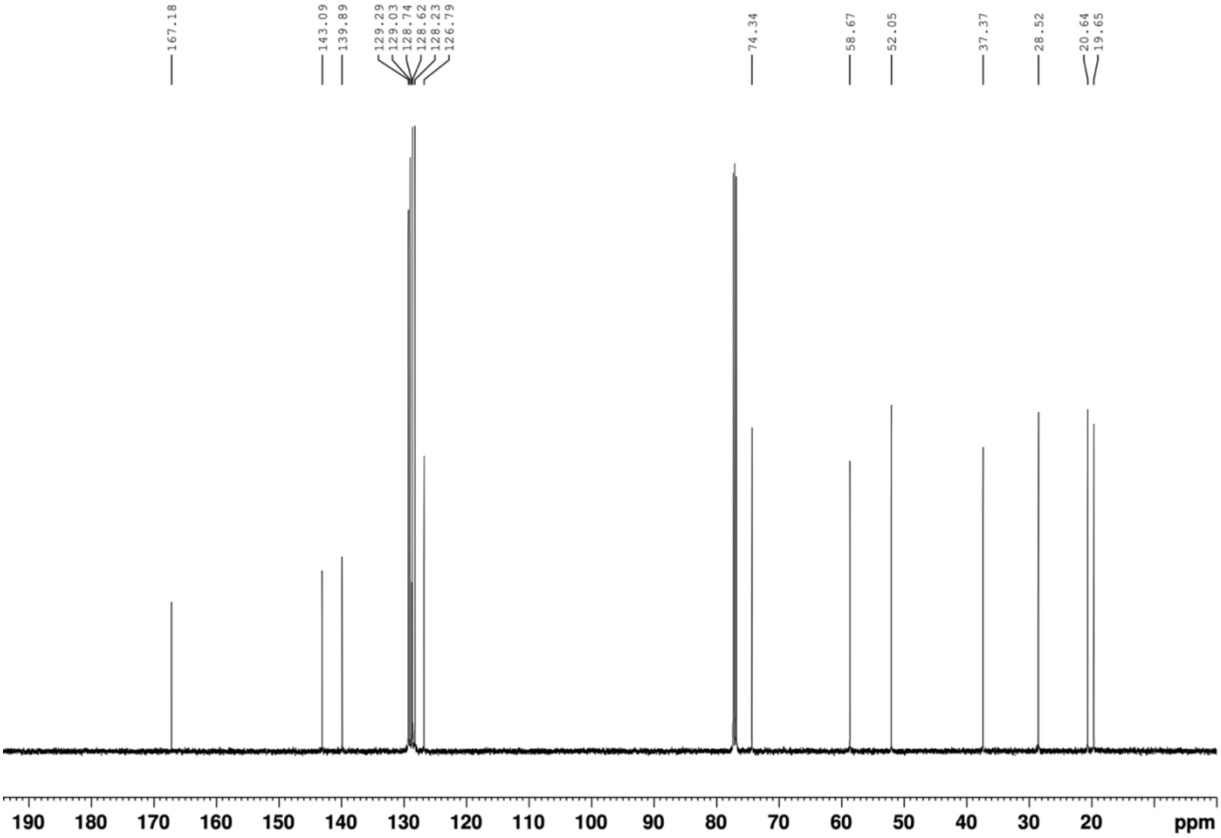

N-benzyl-1-(6-bromobenzo[d][1,3]dioxol-5-yl)-N,2-dimethylpropan-1-amine (12c)

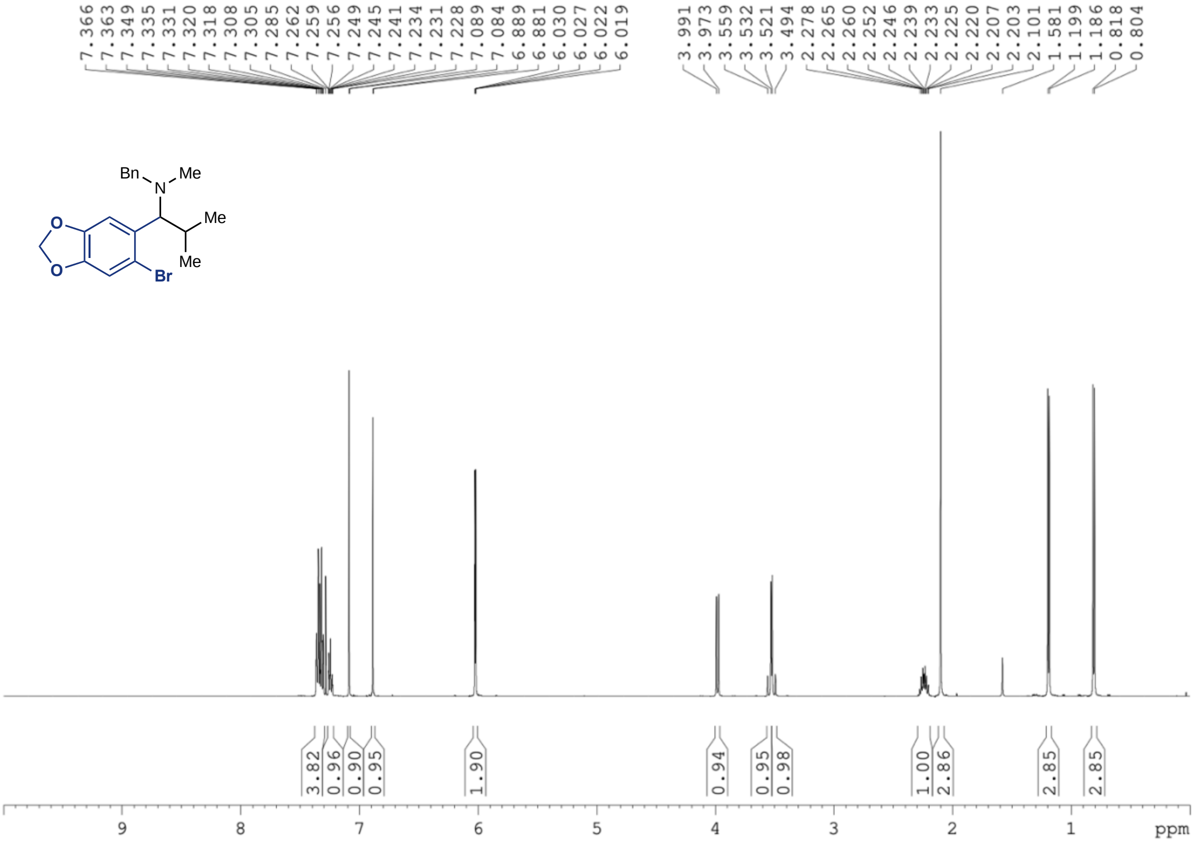

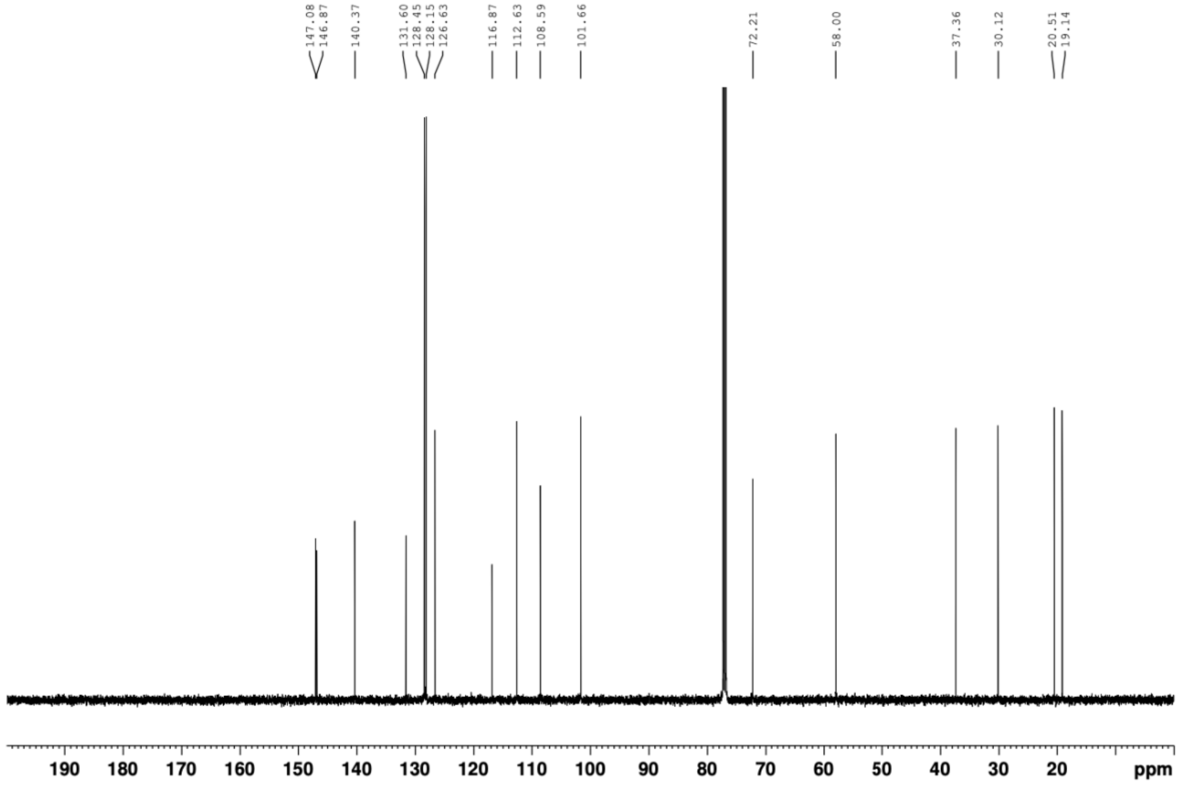

N-benzyl-N,2-dimethyl-1-(1-methyl-1H-pyrazol-4-yl)propan-1-amine (12d)

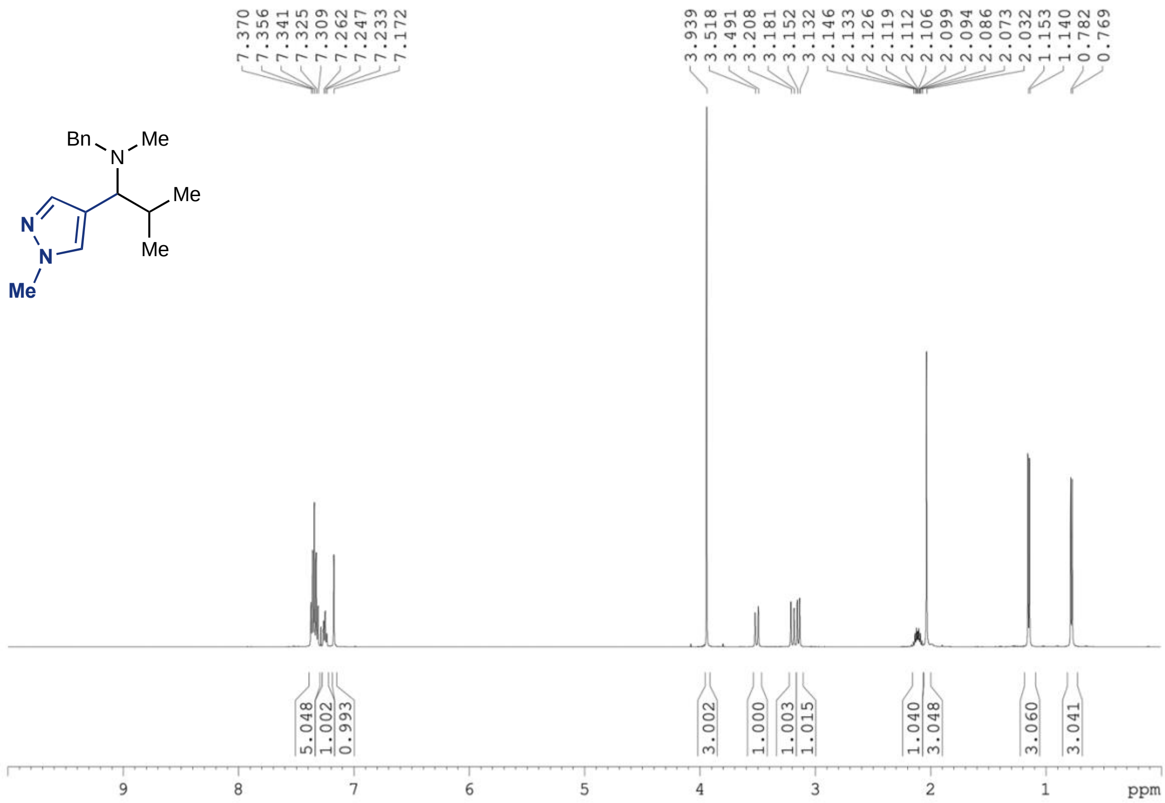

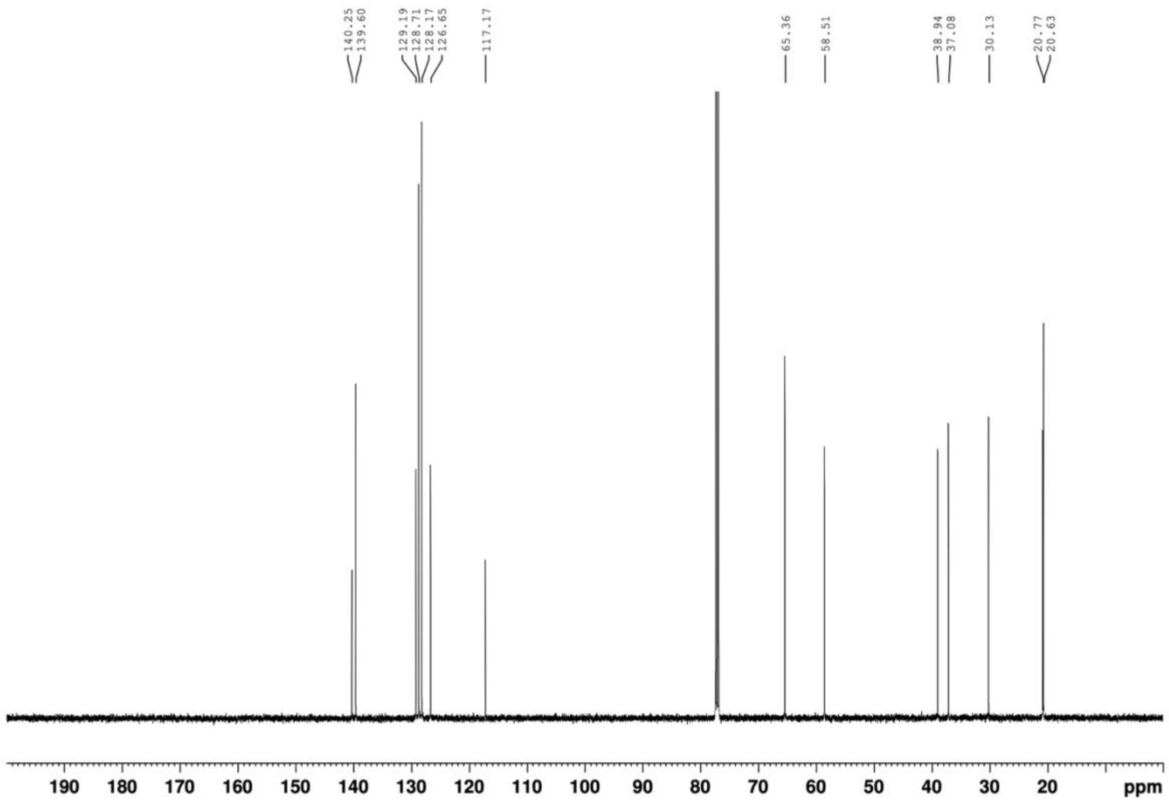

N-benzyl-1-(3,5-dimethylisoxazol-4-yl)-N,2-dimethylpropan-1-amine (12e)

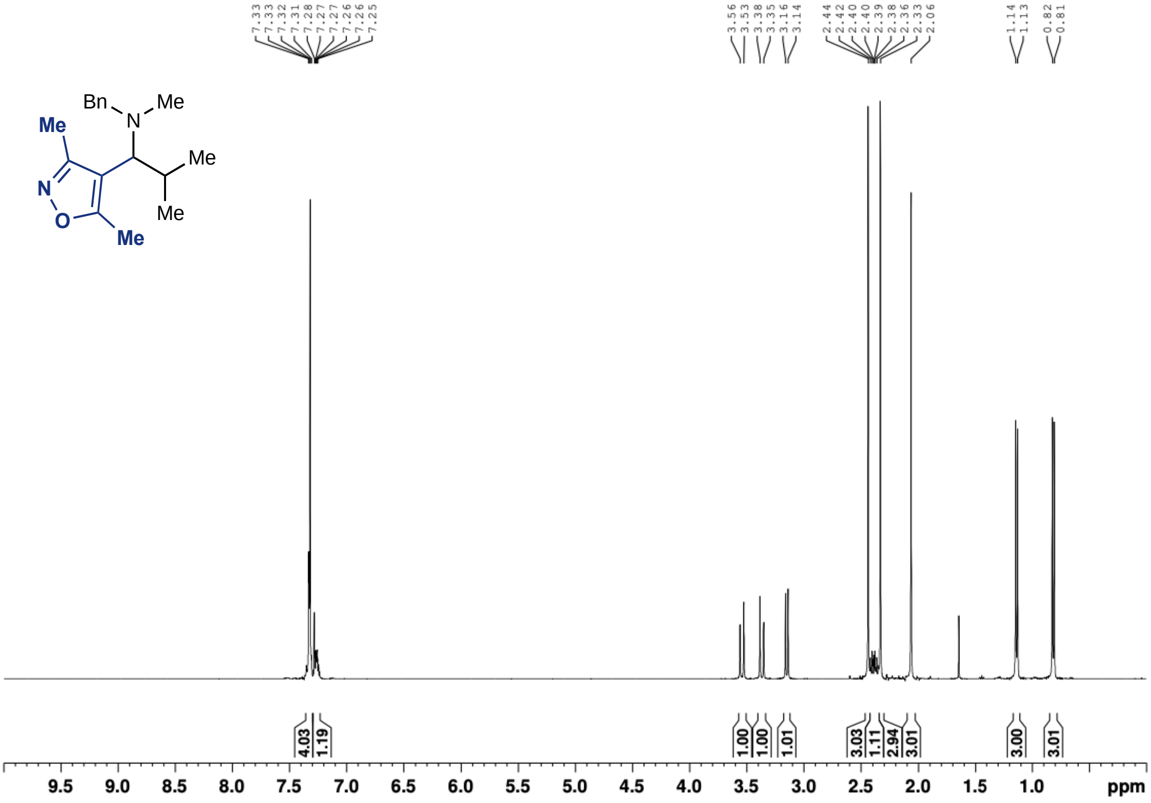

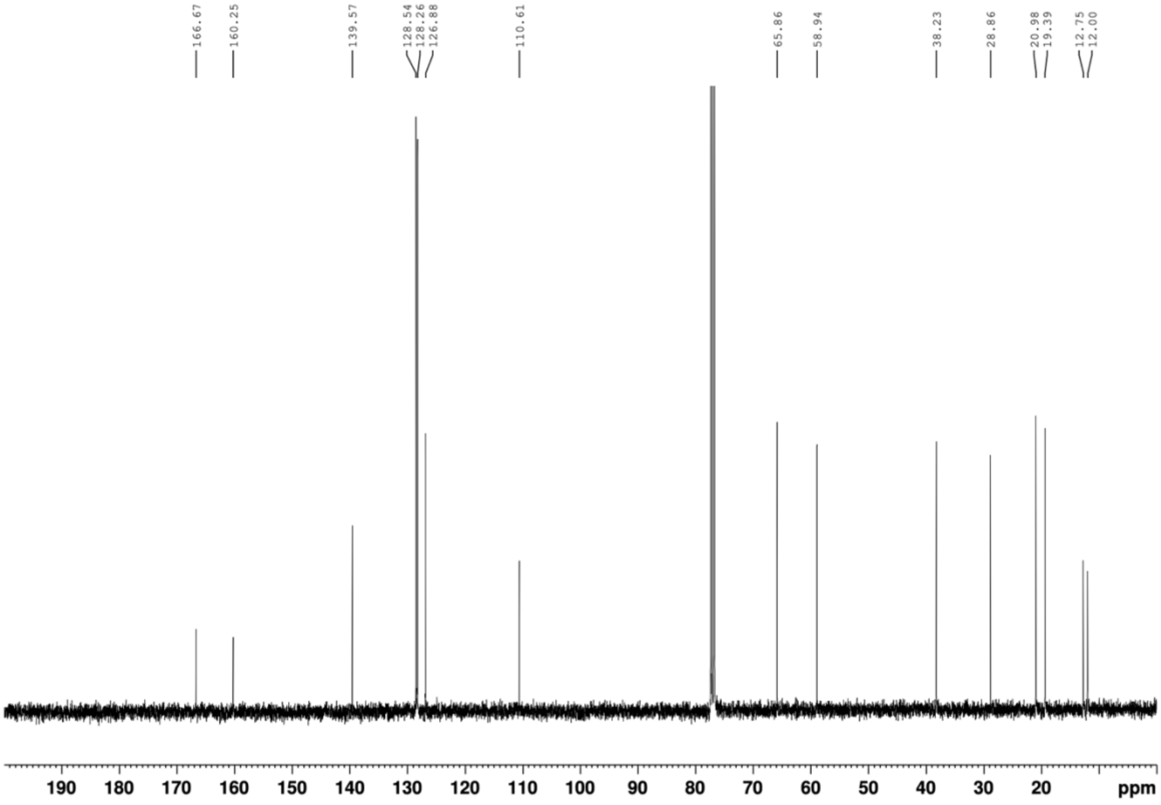

N-benzyl-1-(6-bromopyridin-2-yl)-N,2-dimethylpropan-1-amine (12f)

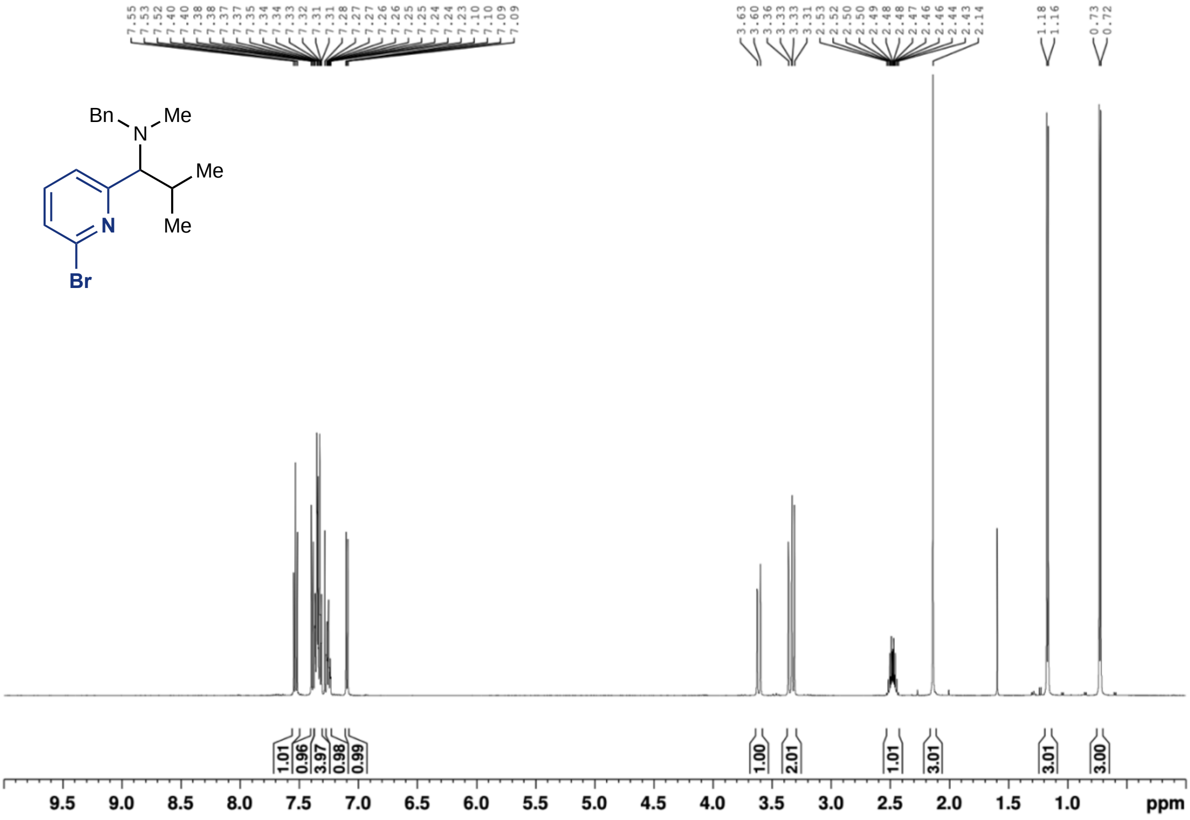

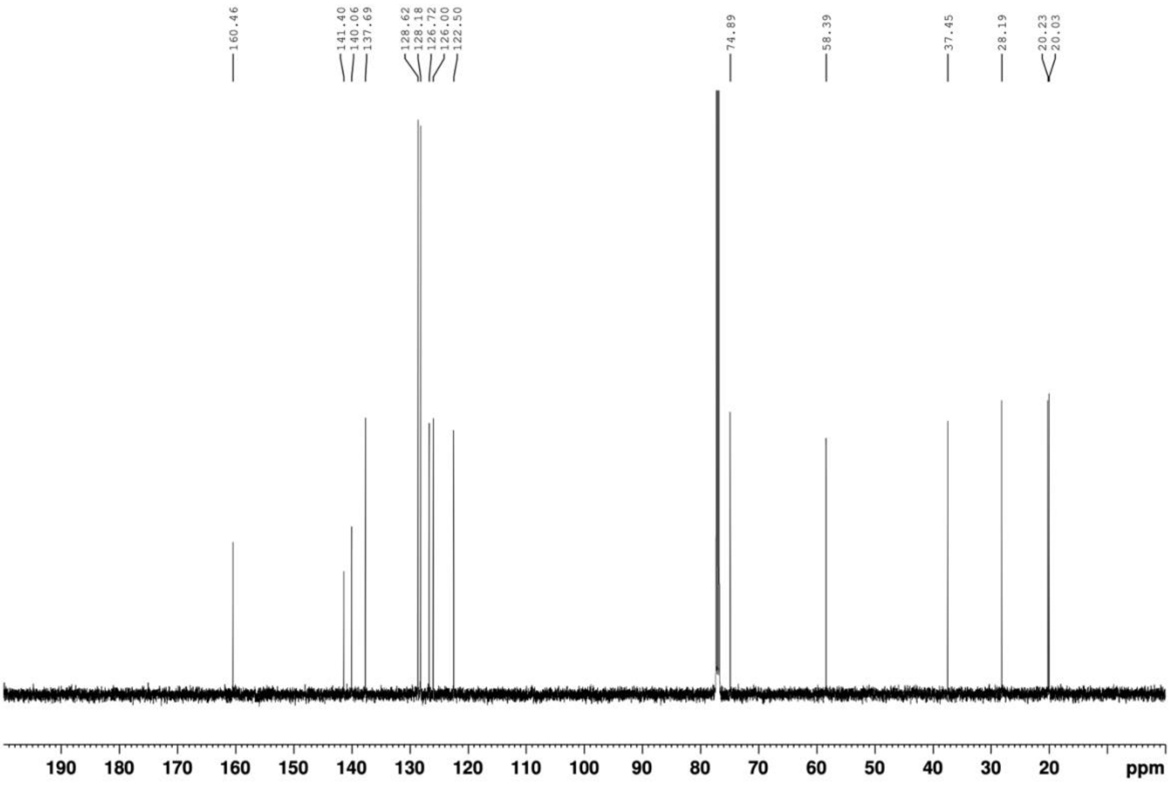

N-benzyl-1-(3-(4-bromophenyl)isoxazol-5-yl)-N,2-dimethylpropan-1-amine (12g)

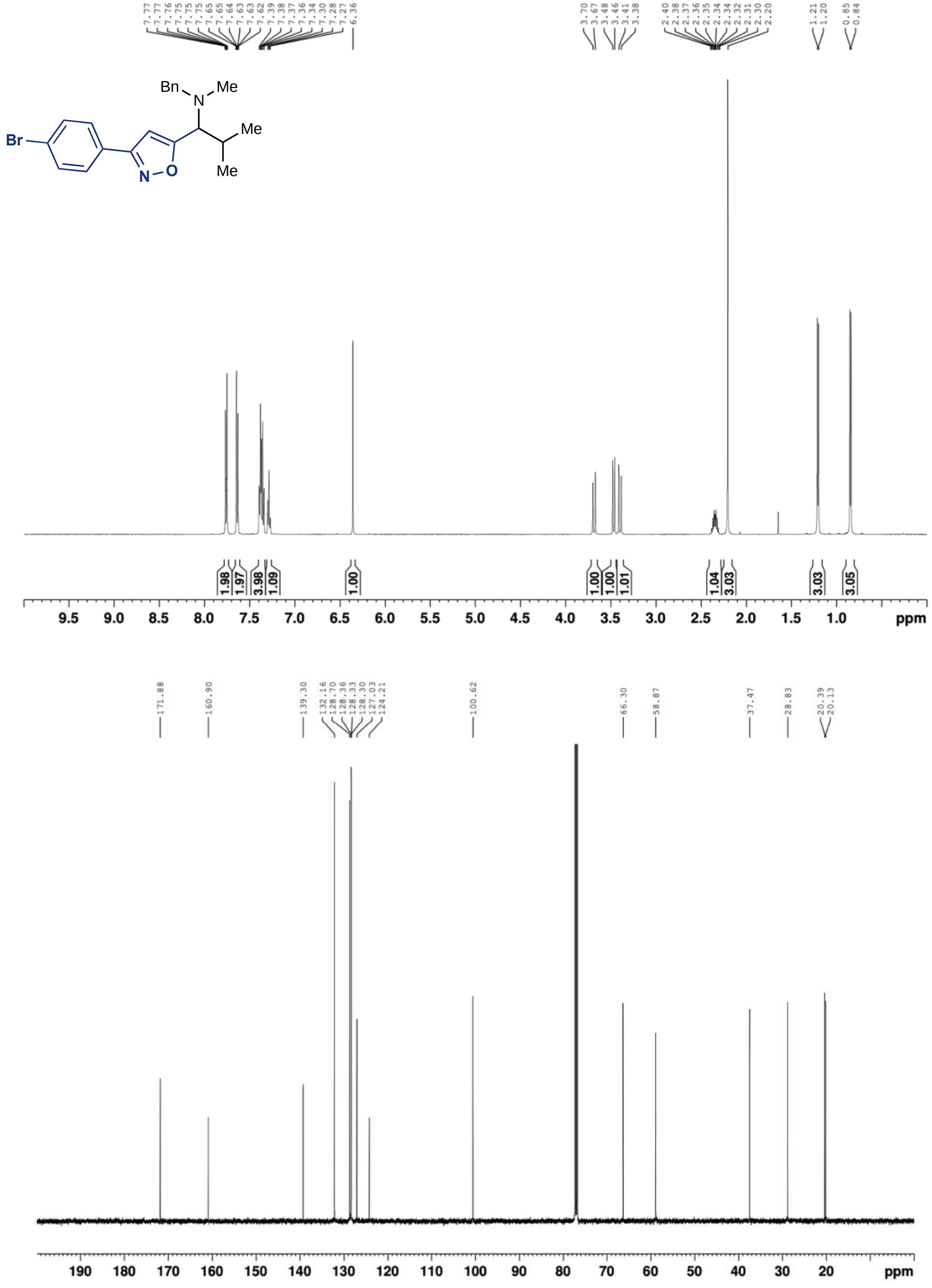

(4-(2-methyl-1-phenylpropyl)piperazin-1-yl)(naphthalen-2-yl)methanone (14a)

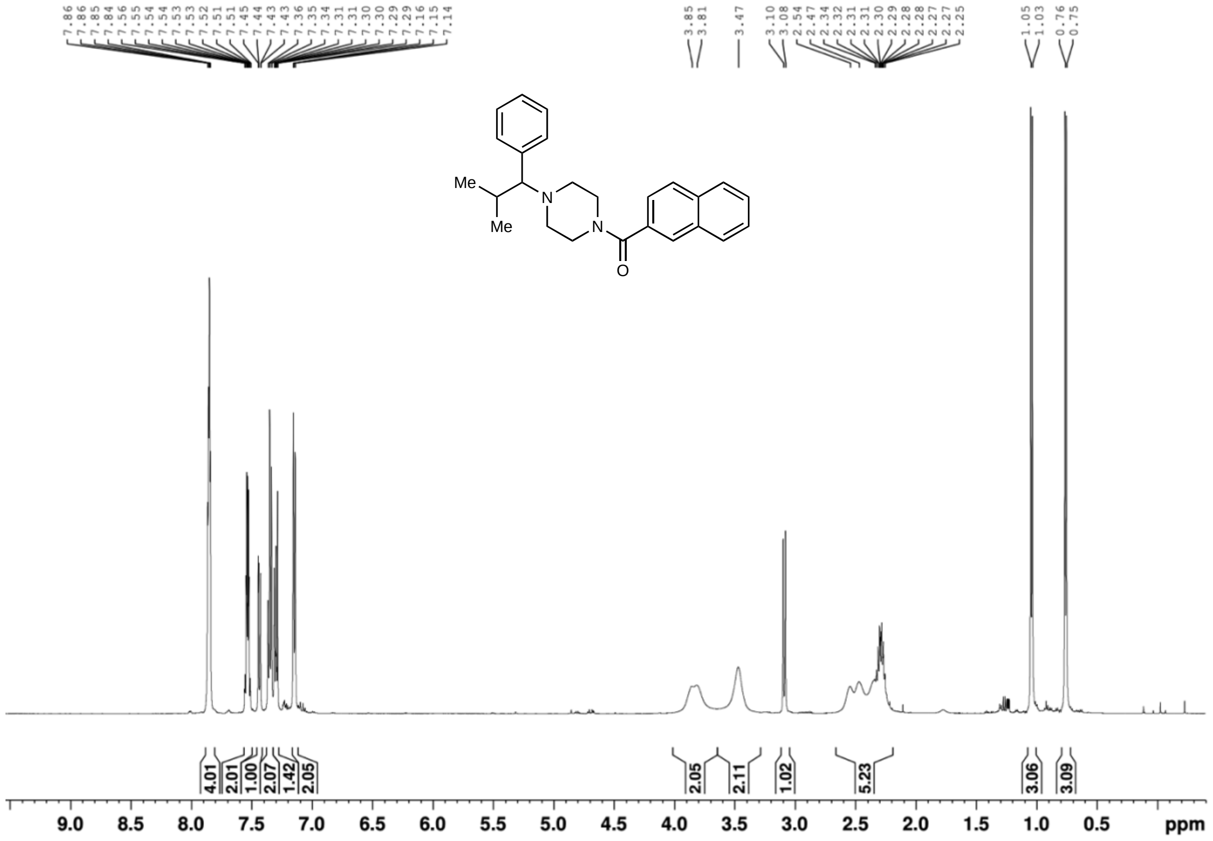

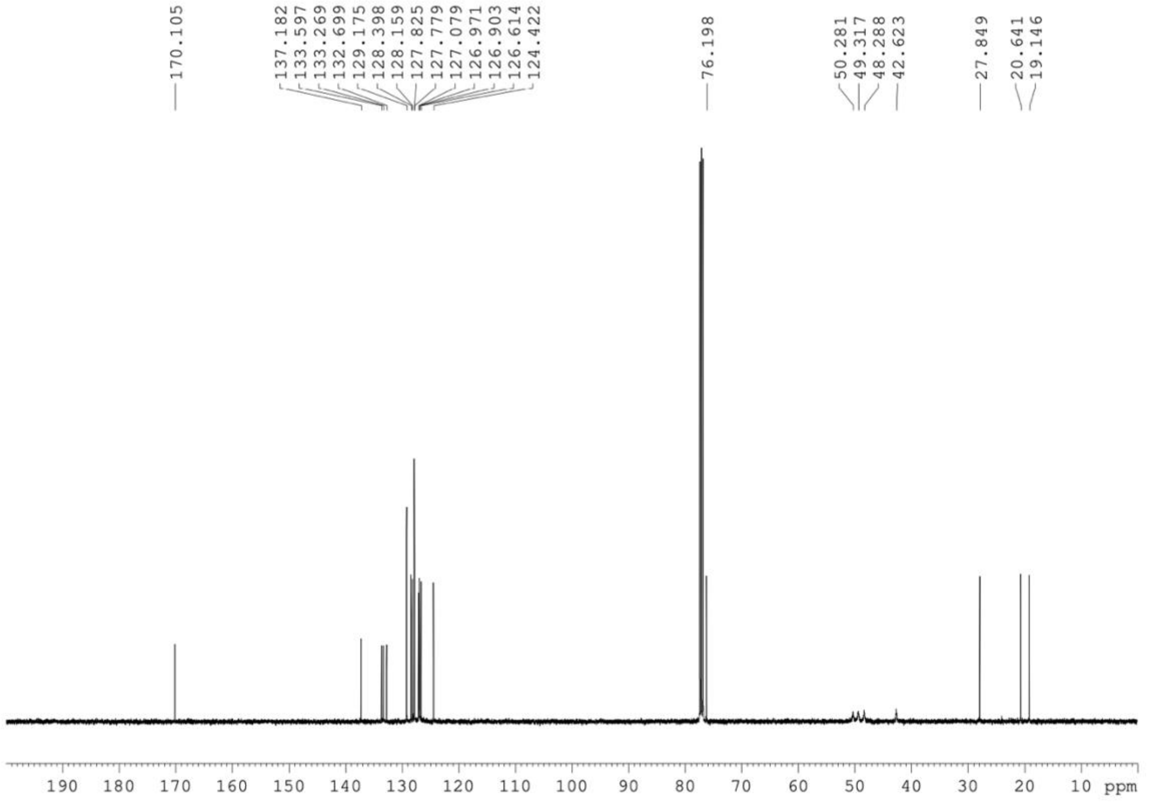

4,5,6,7-tetrachloro-1,3-dioxoisoindolin-2-yl pivalate (15c

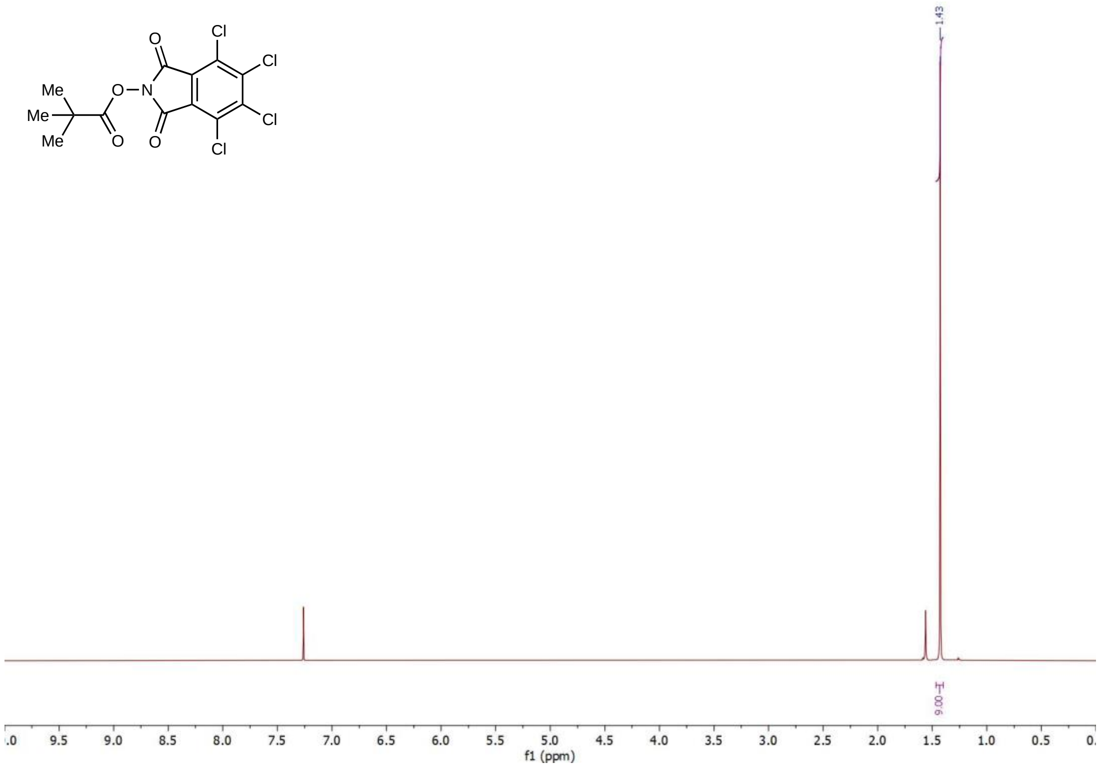

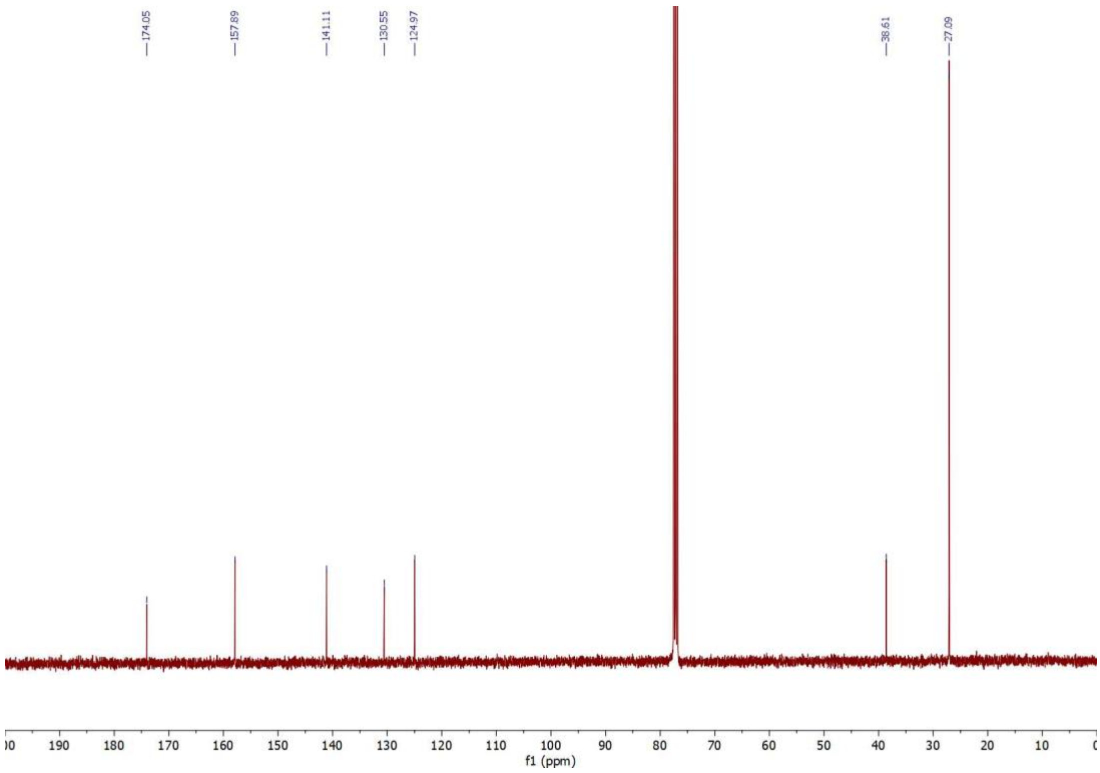

4,5,6,7-tetrachloro-1,3-dioxoisoindolin-2-yl acetate (15d)

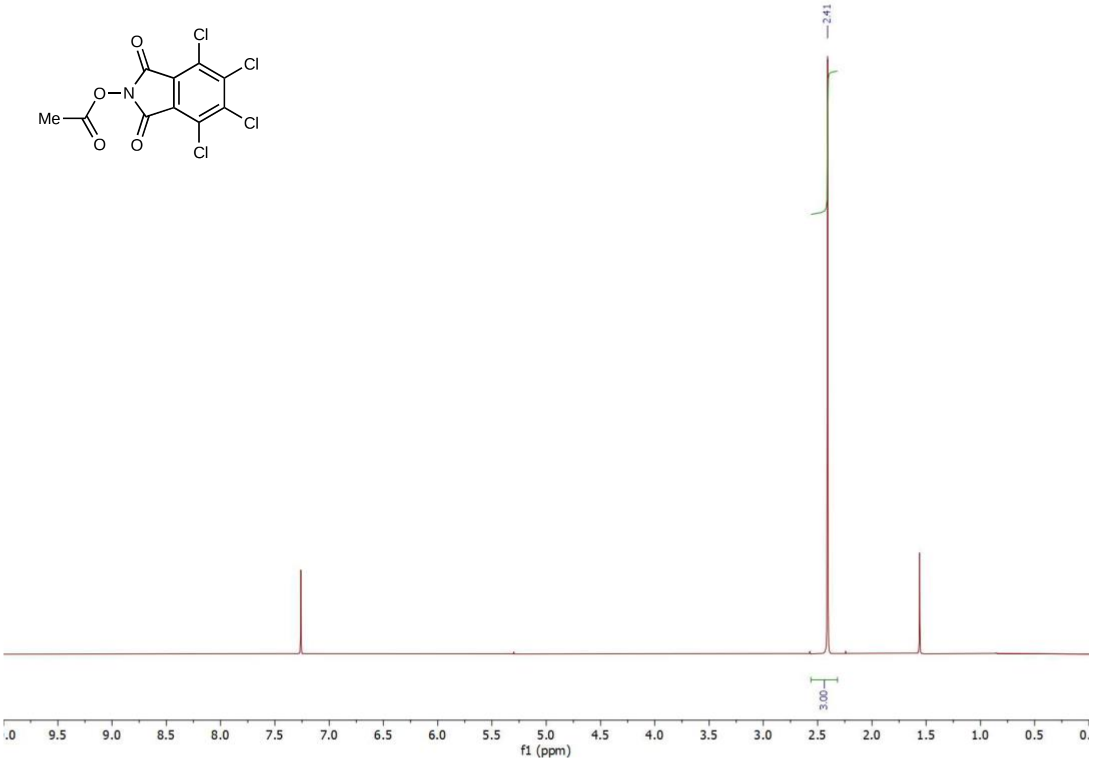

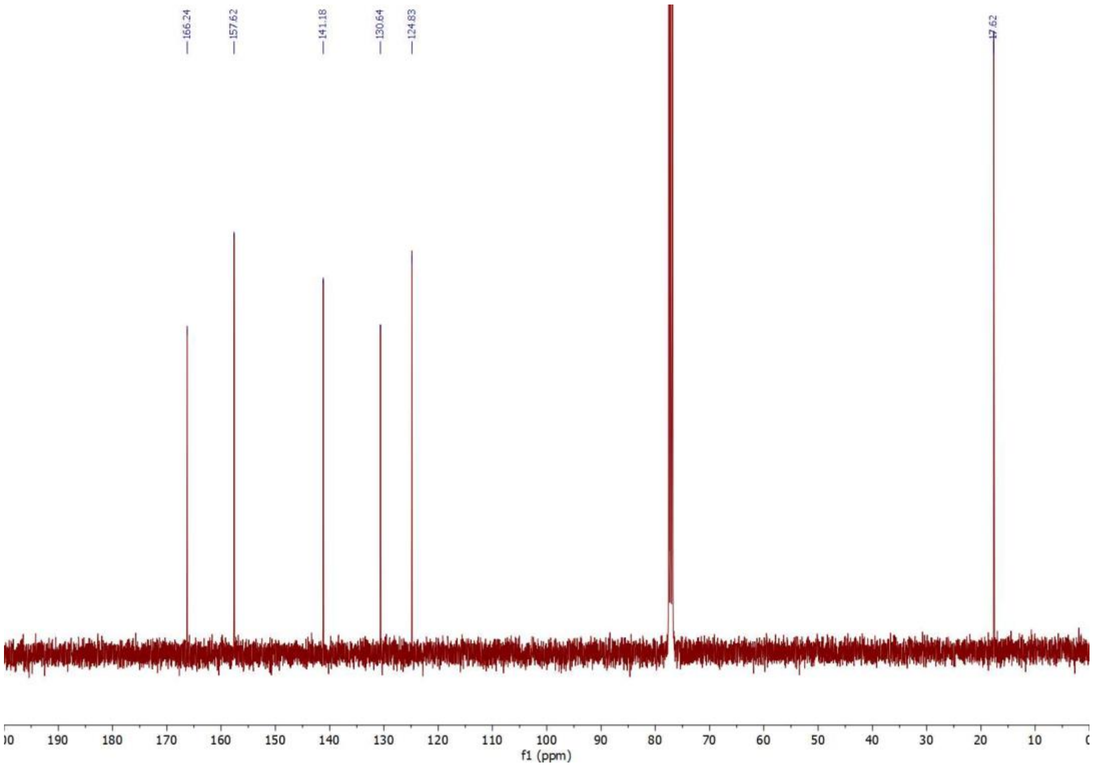

4,5,6,7-tetrachloro-1,3-dioxoisoindolin-2-yl cycloheptanecarboxylate (15i)

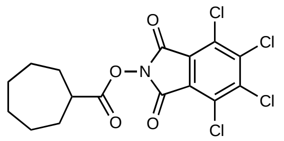

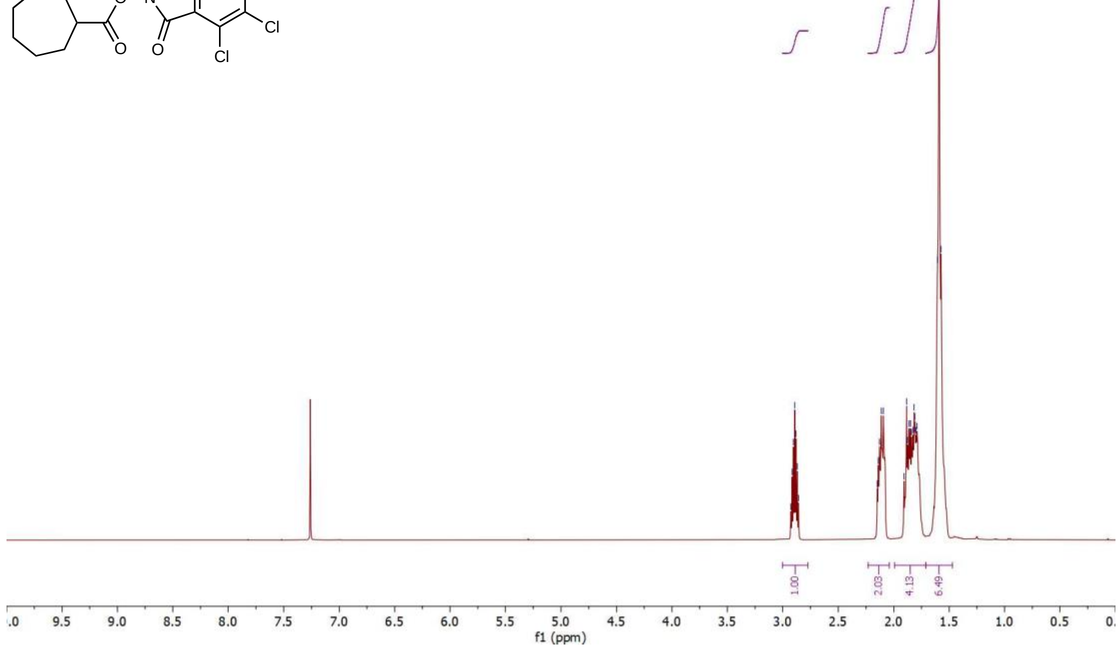

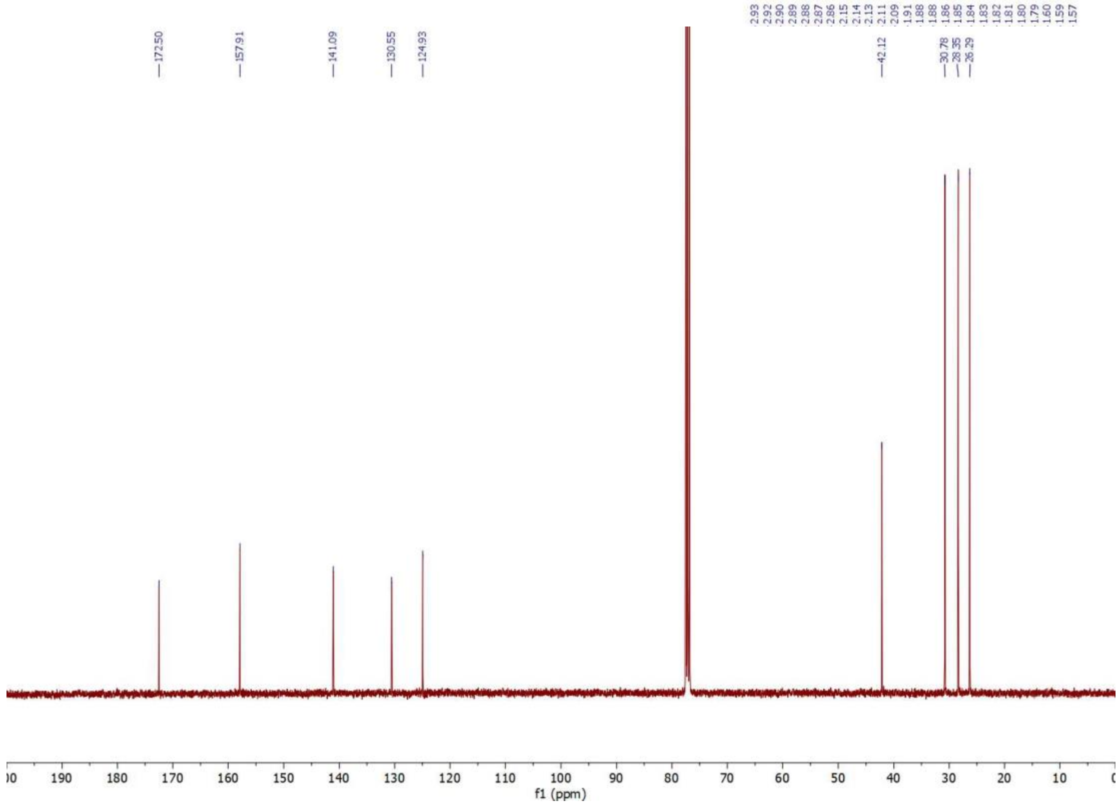

4,5,6,7-tetrachloro-1,3-dioxoisoindolin-2-yl 3-oxocyclobutane-1-carboxylate (15j)

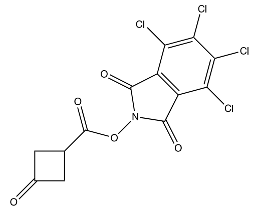

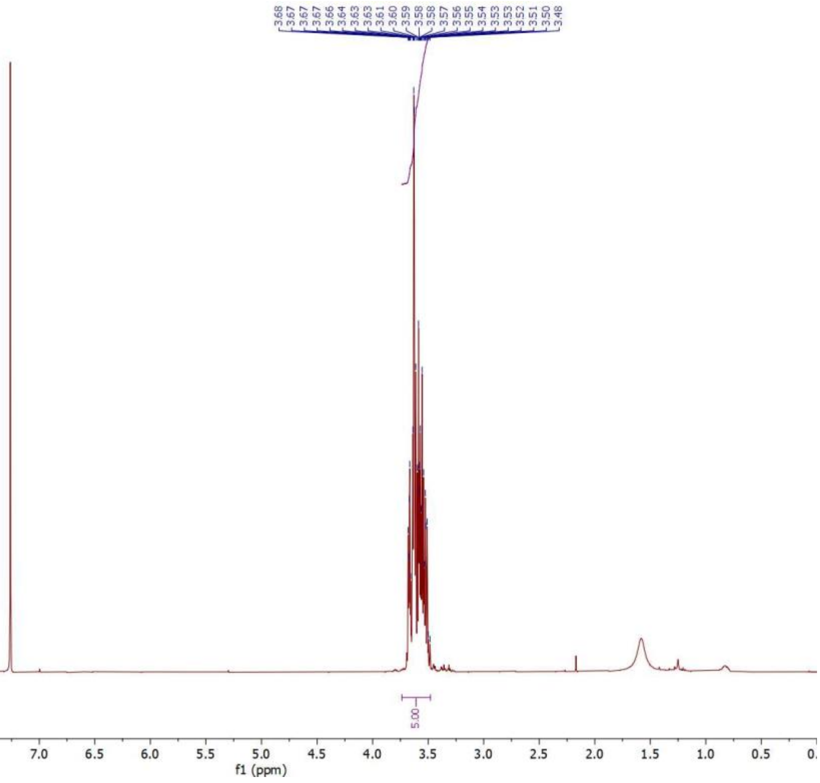

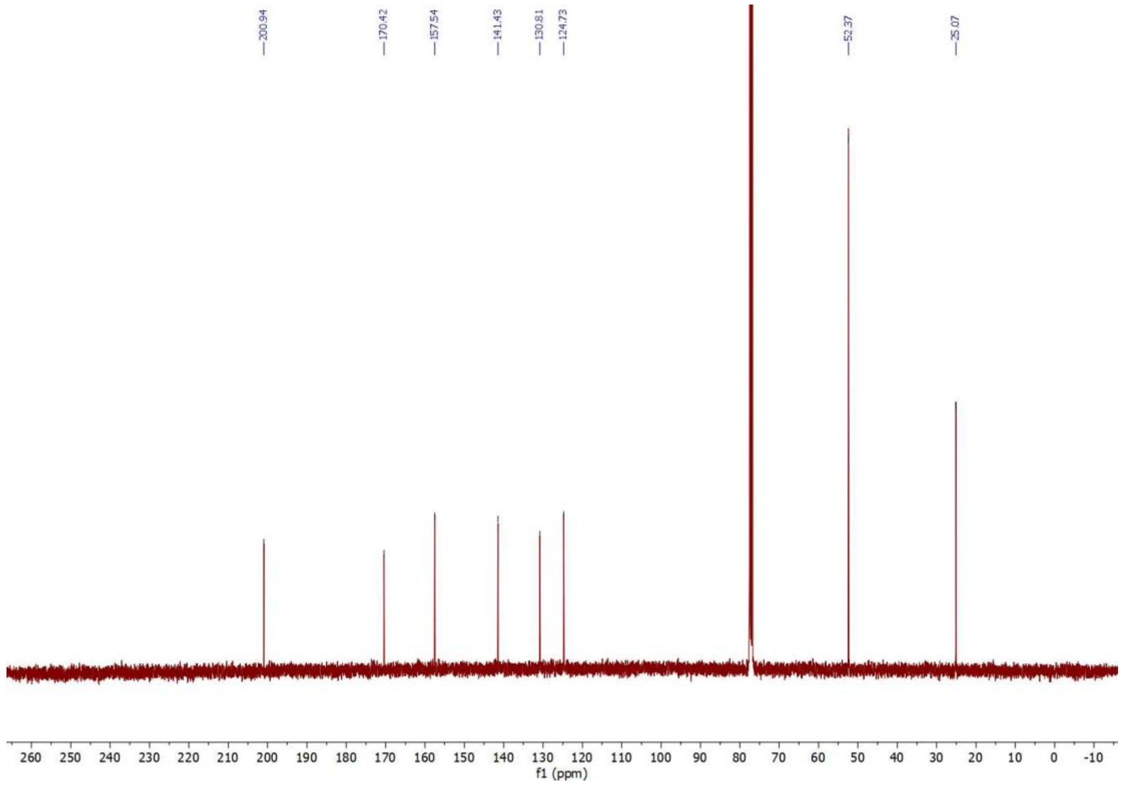

4,5,6,7-tetrachloro-1,3-dioxoisoindolin-2-yl 3,3-difluorocyclobutane-1-carboxylate (15k)

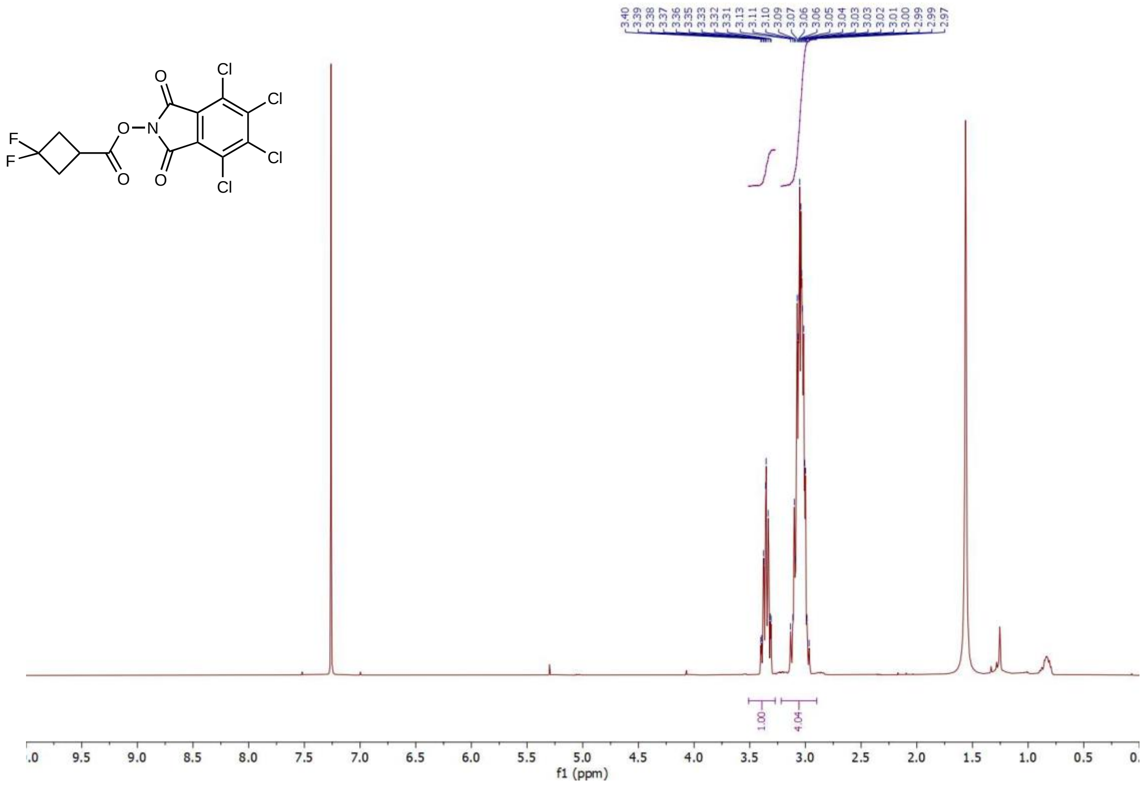

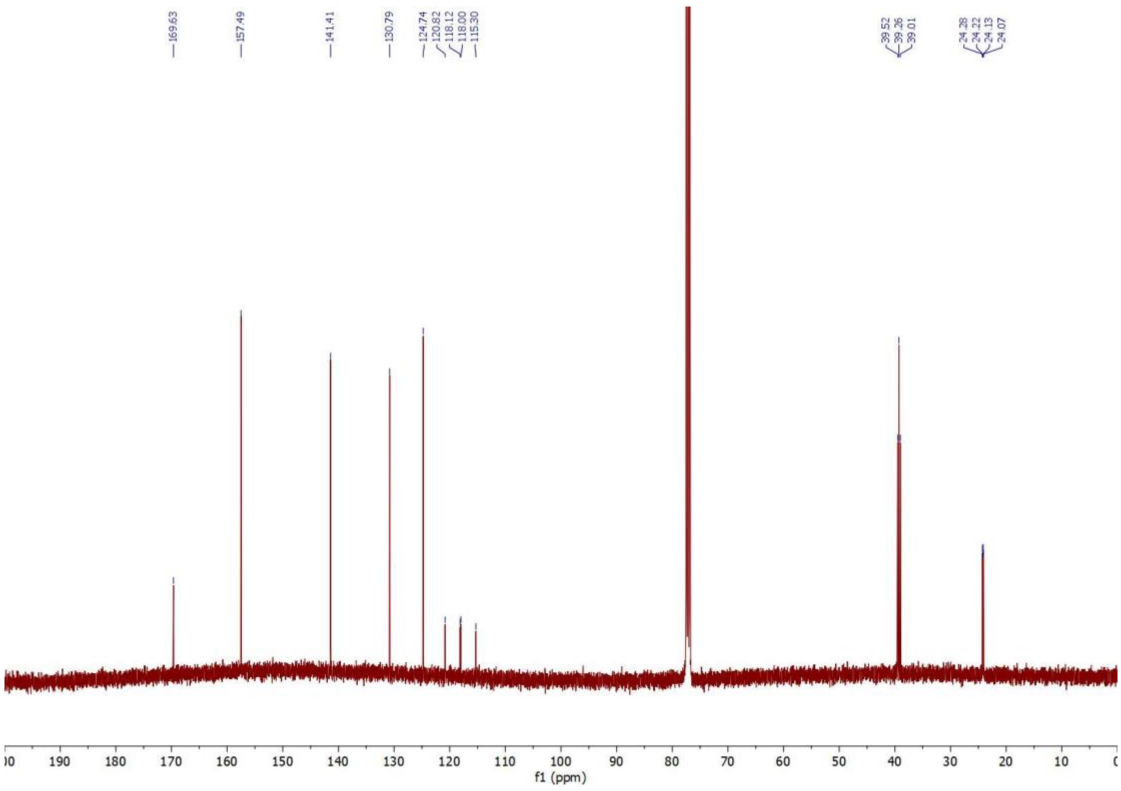

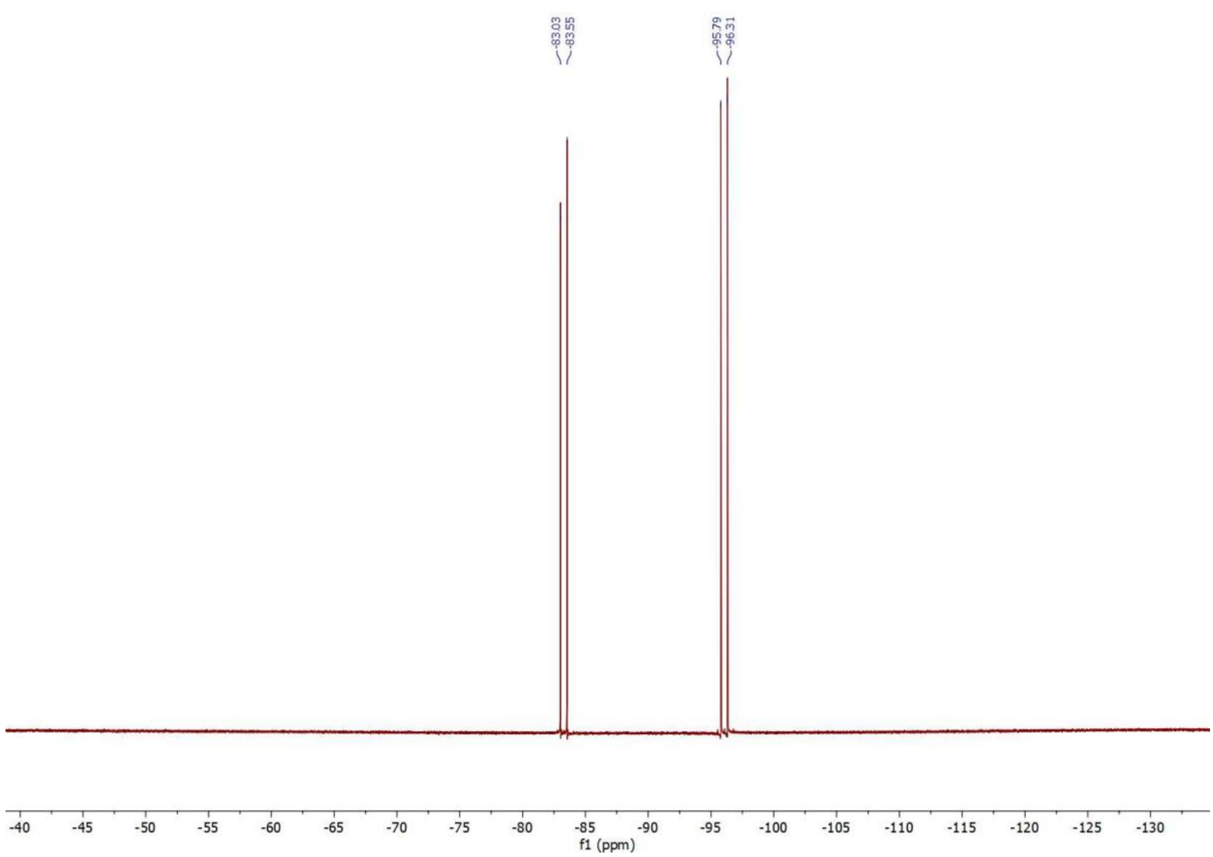
-83.03
-83.55
-95.79
-96.31
-40 -45 -50 -55 -60 -65 -70 -75 -80 -85 -90 -95 -100 -105 -110 -115 -120 -125 -130
f1 (ppm)

4,5,6,7-tetrachloro-1,3-dioxoisoindolin-2-yl 3-methoxypropanoate (15l)

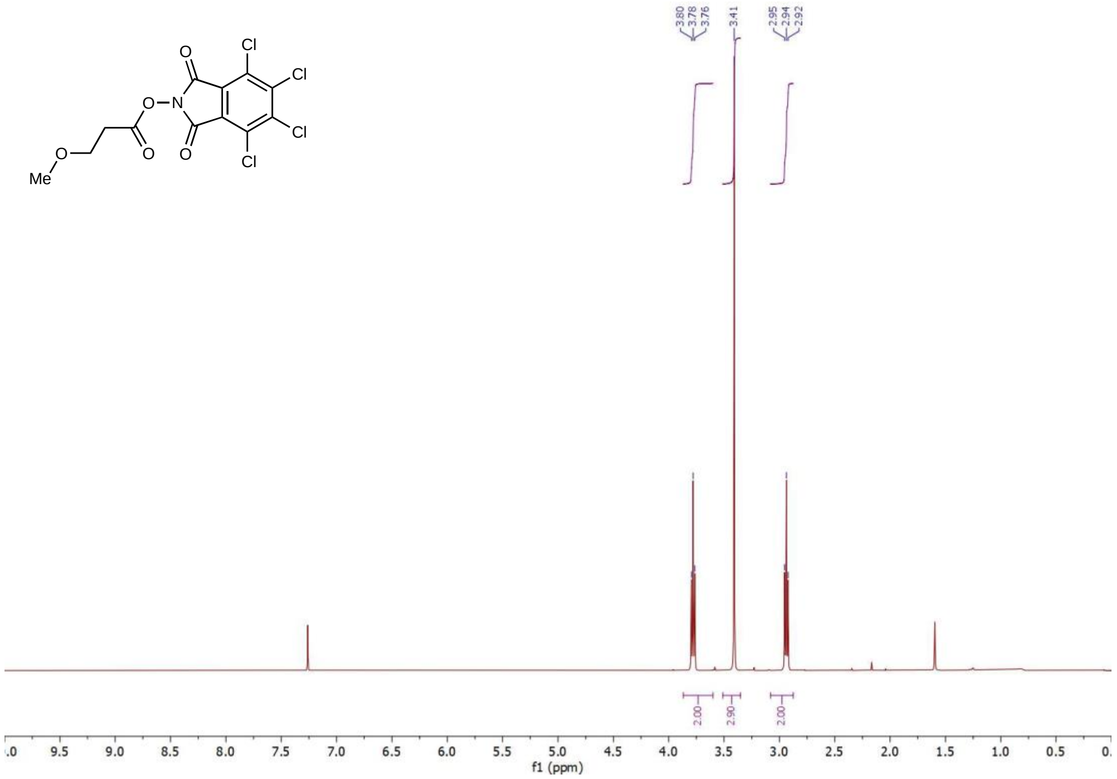

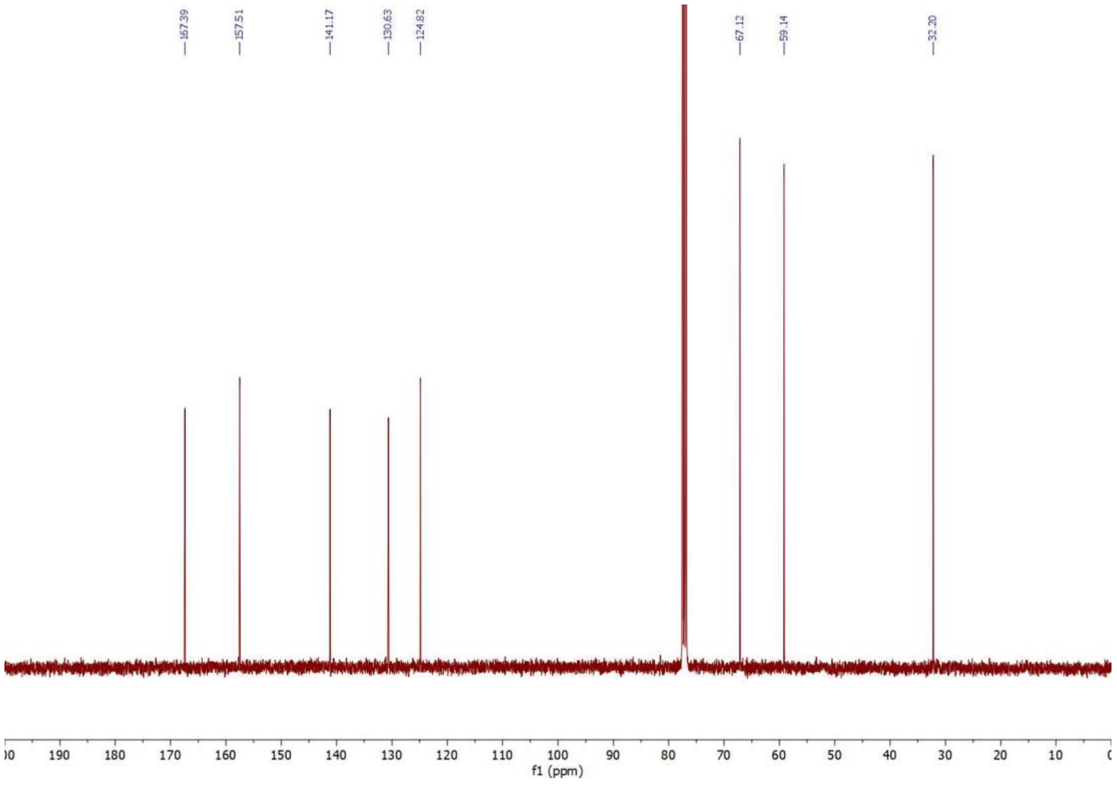

4,5,6,7-Tetrachloro-1,3-dioxoisoindolin-2-yl 2-(methylthio)acetate (15l)

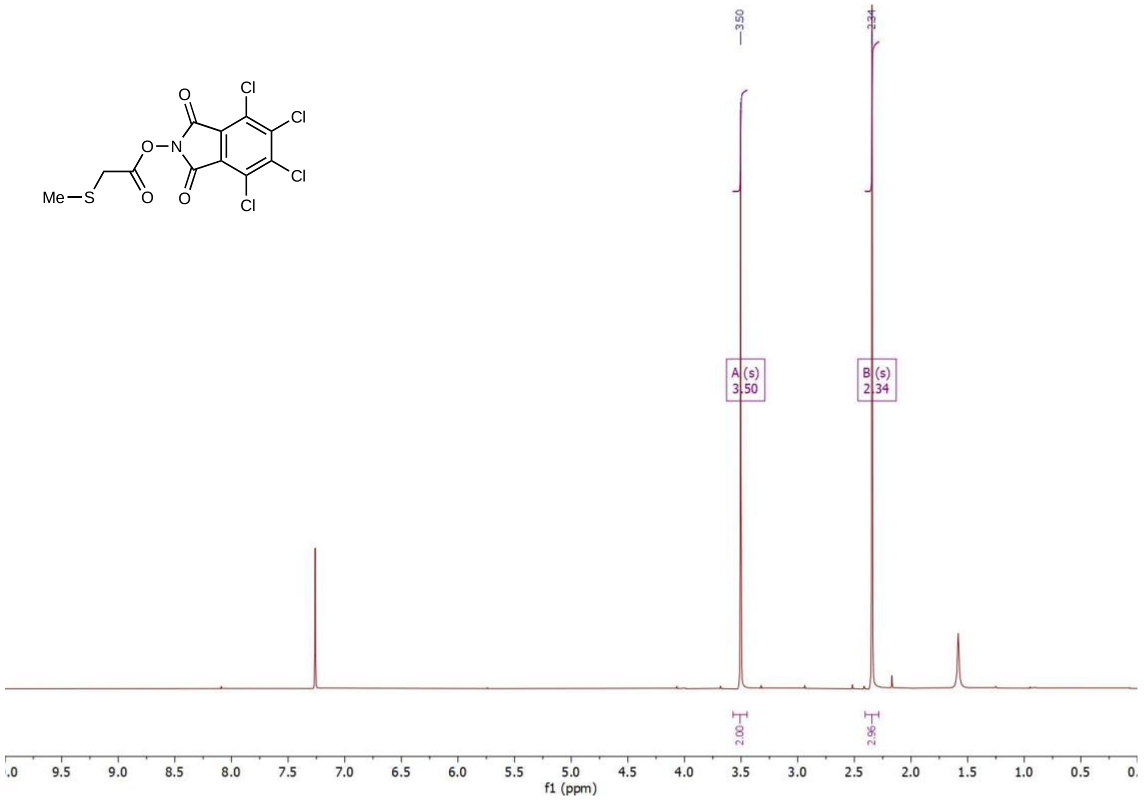

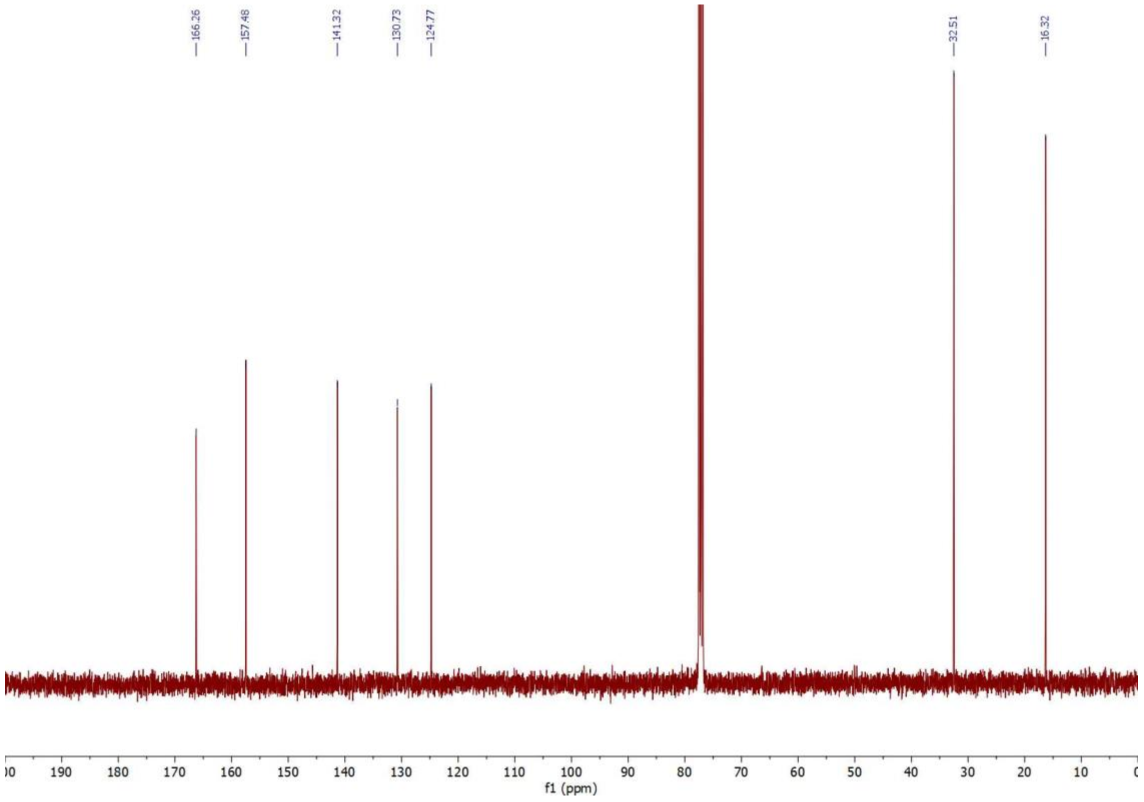

4,5,6,7-Tetrachloro-1,3-dioxoisoindolin-2-yl 2-(1,3-dioxoisoindolin-2-yl)acetate (15m)

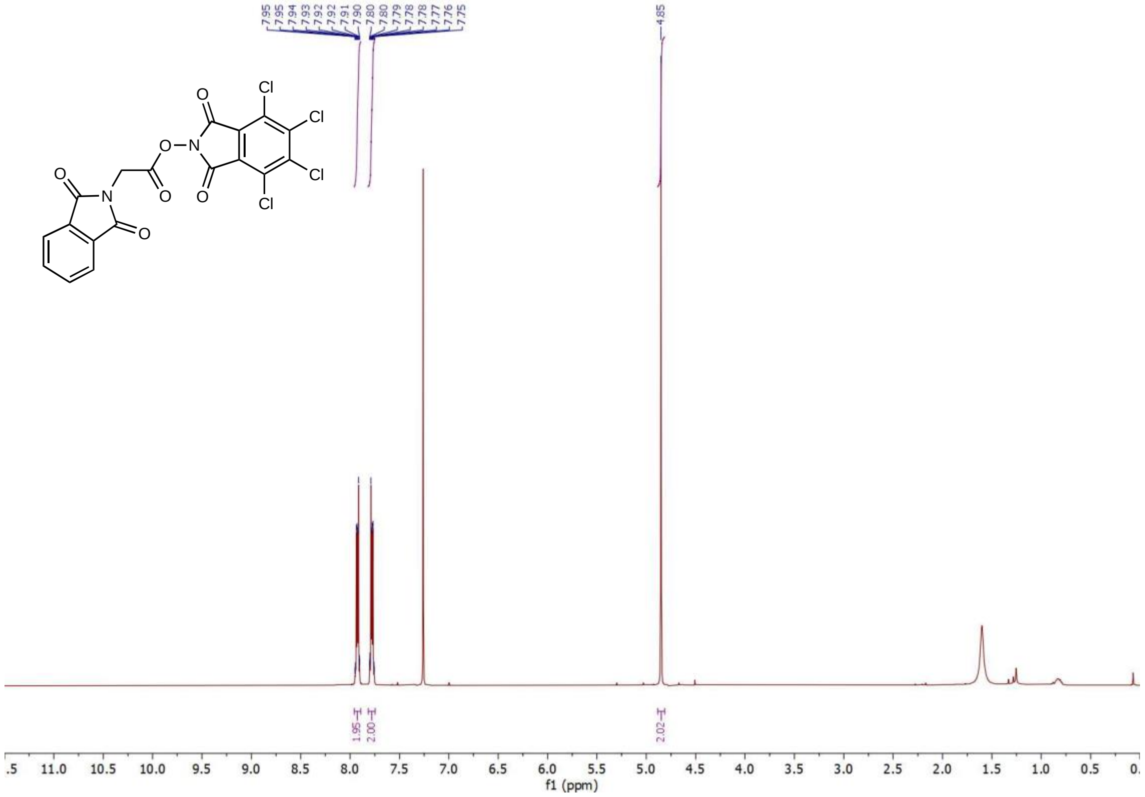

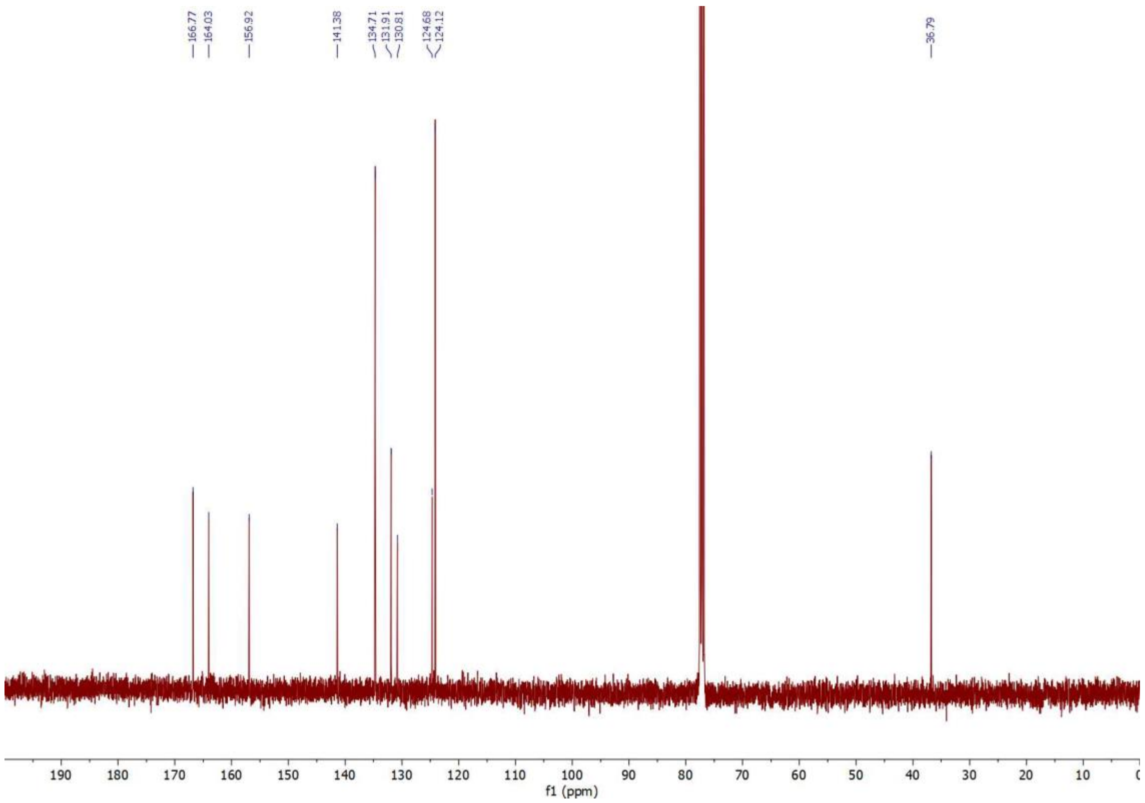

N-benzyl-N-methyl-1-phenylpentan-3-amine (16a)

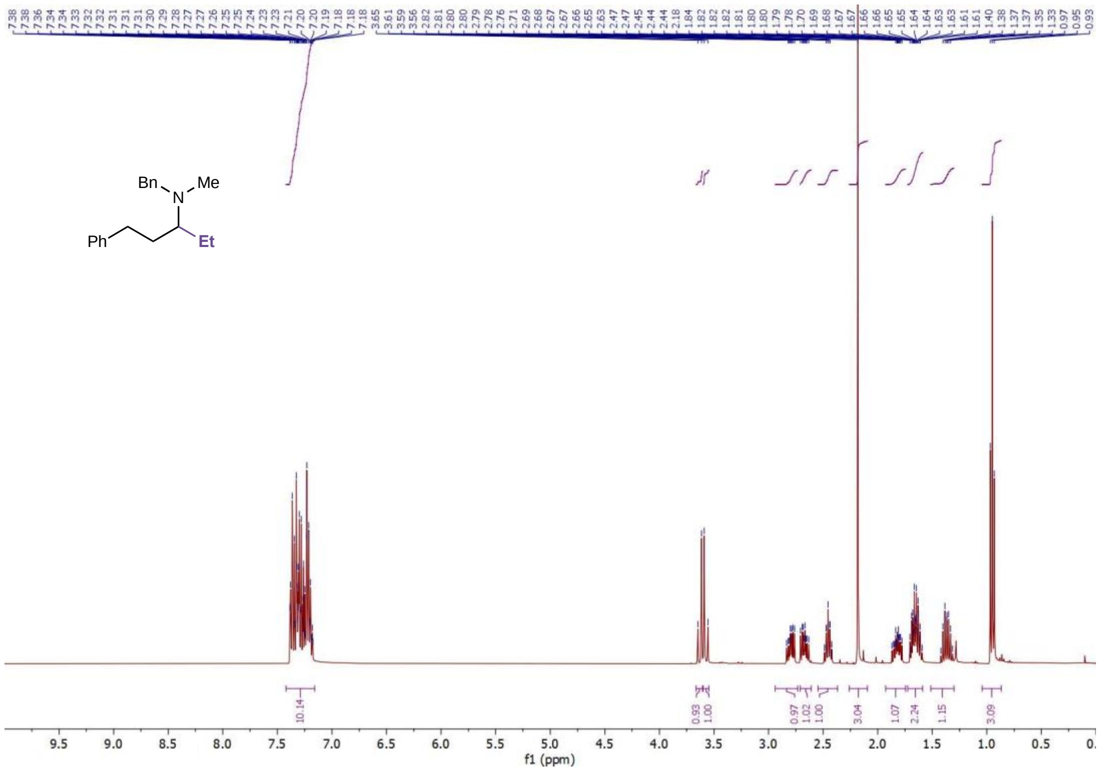

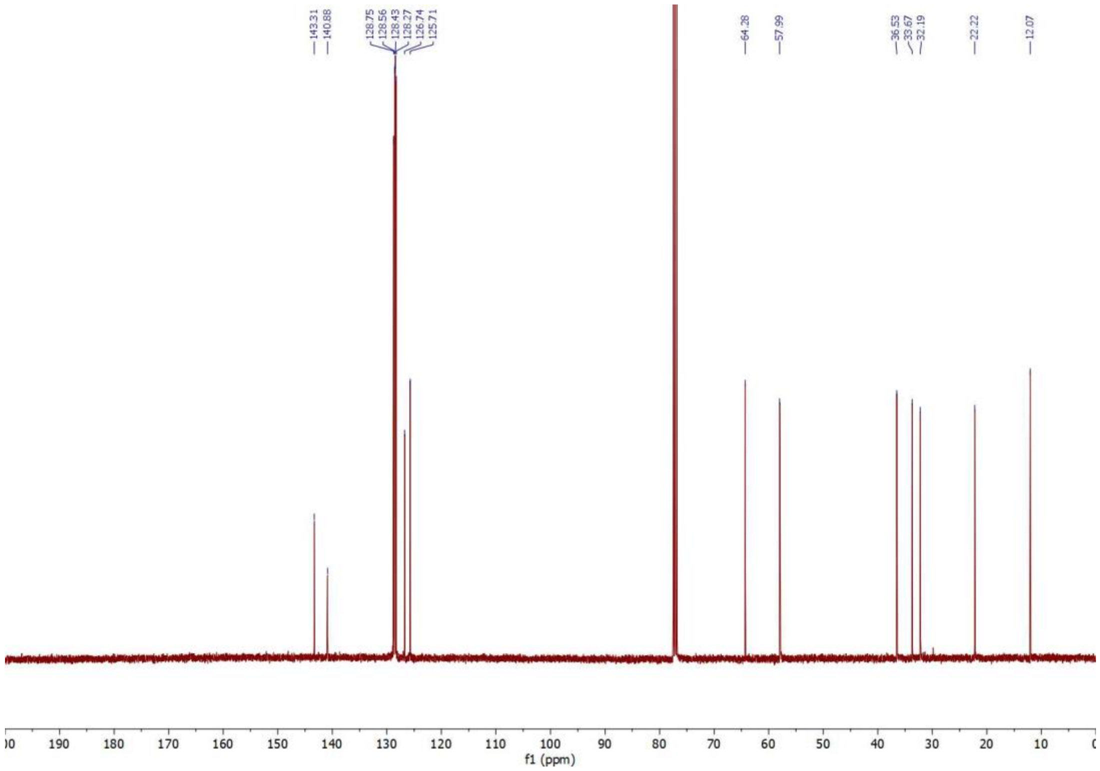

N-benzyl-N-methyl-1-(1-methylcyclopropyl)-3-phenylpropan-1-amine amine (16b)

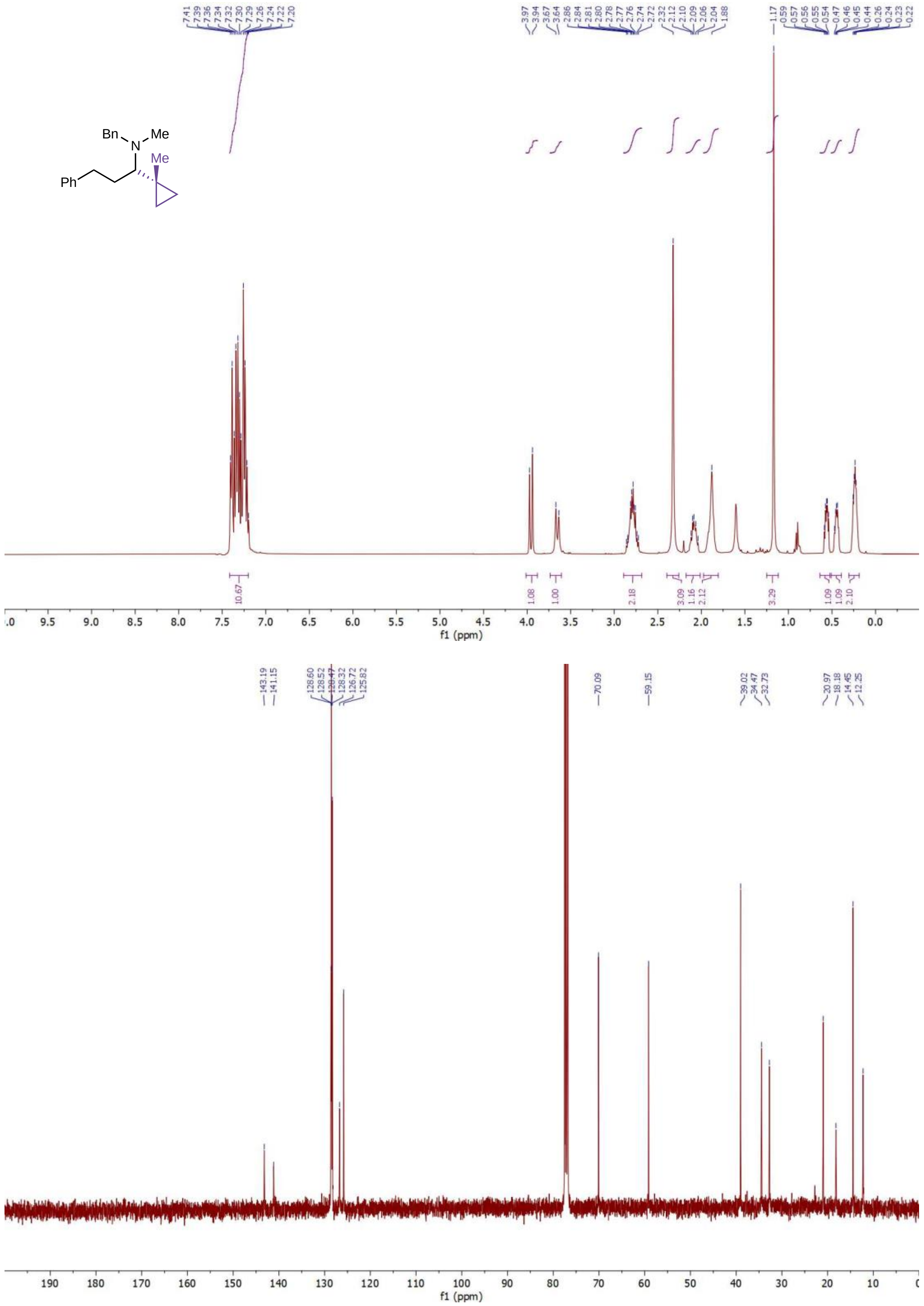

N-benzyl-1-cyclobutyl-N-methyl-3-phenylpropan-1-amine (16c)

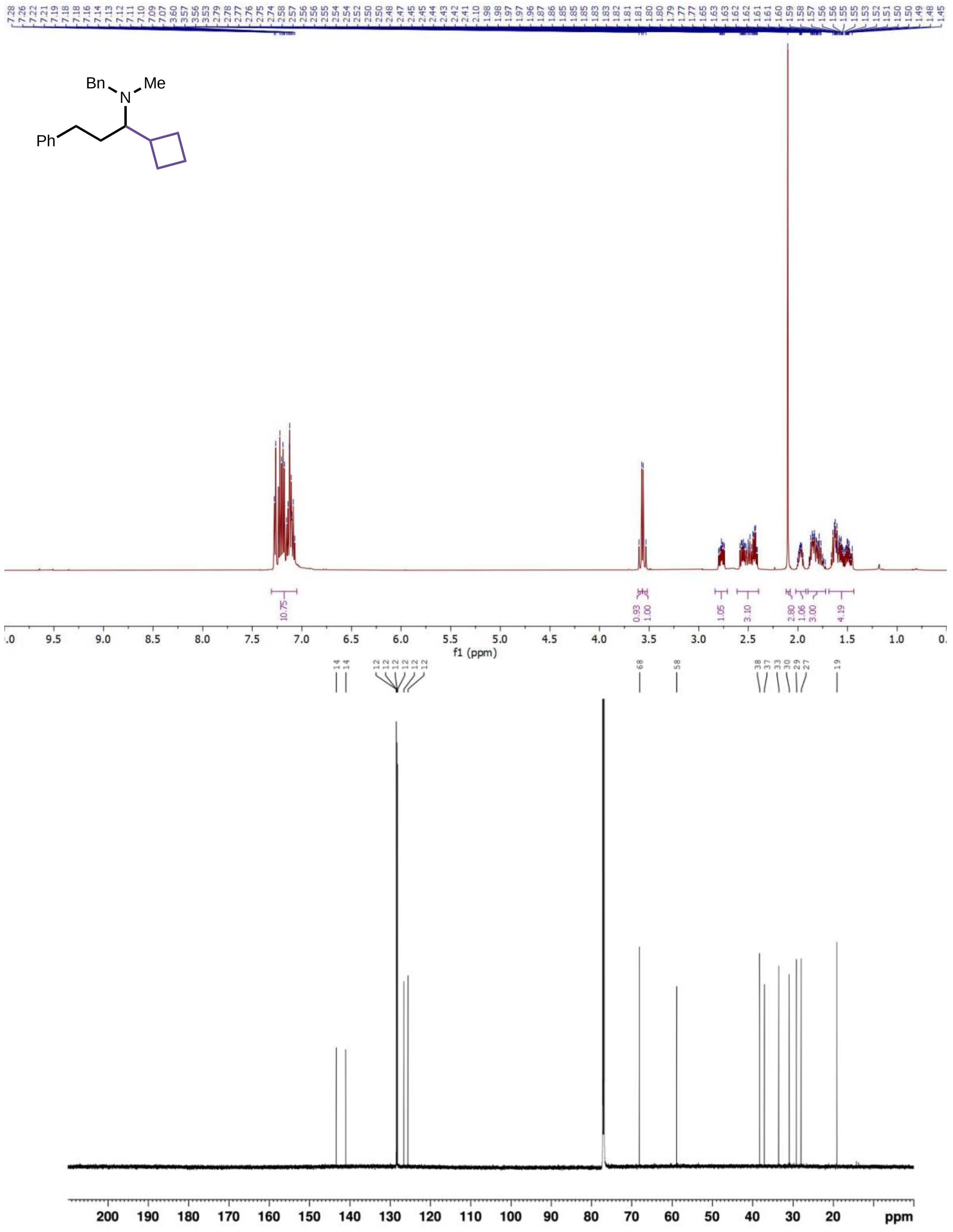

N-benzyl-N-methyl-1-(3-methyloxetan-3-yl)-3-phenylpropan-1-amine (16d)

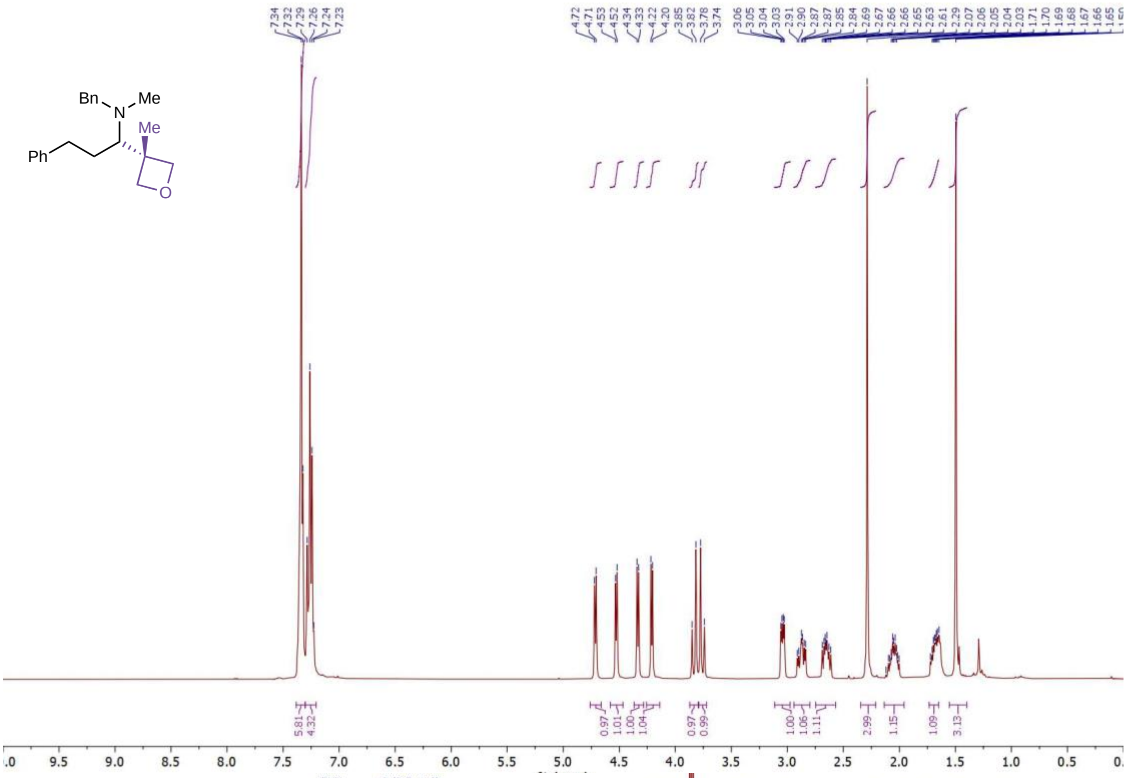

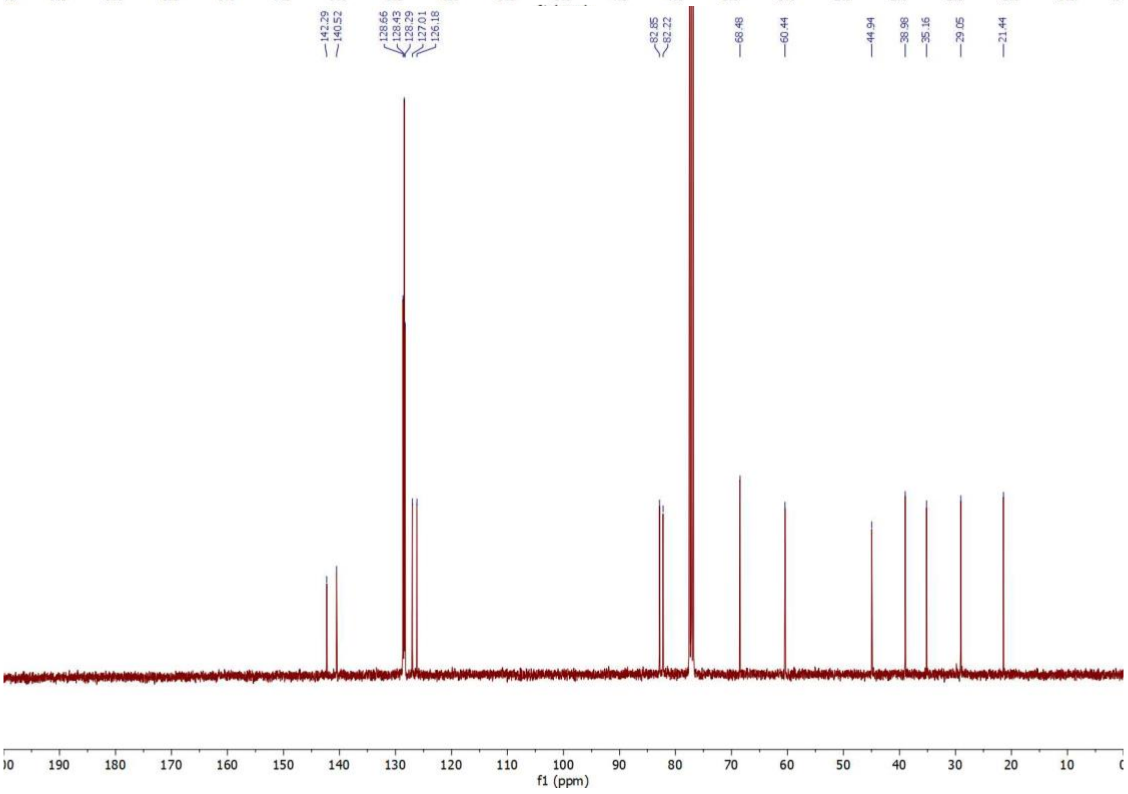

3-(1-(benzyl(methyl)amino)-3-phenylpropyl)cyclobutan-1-one (16e)

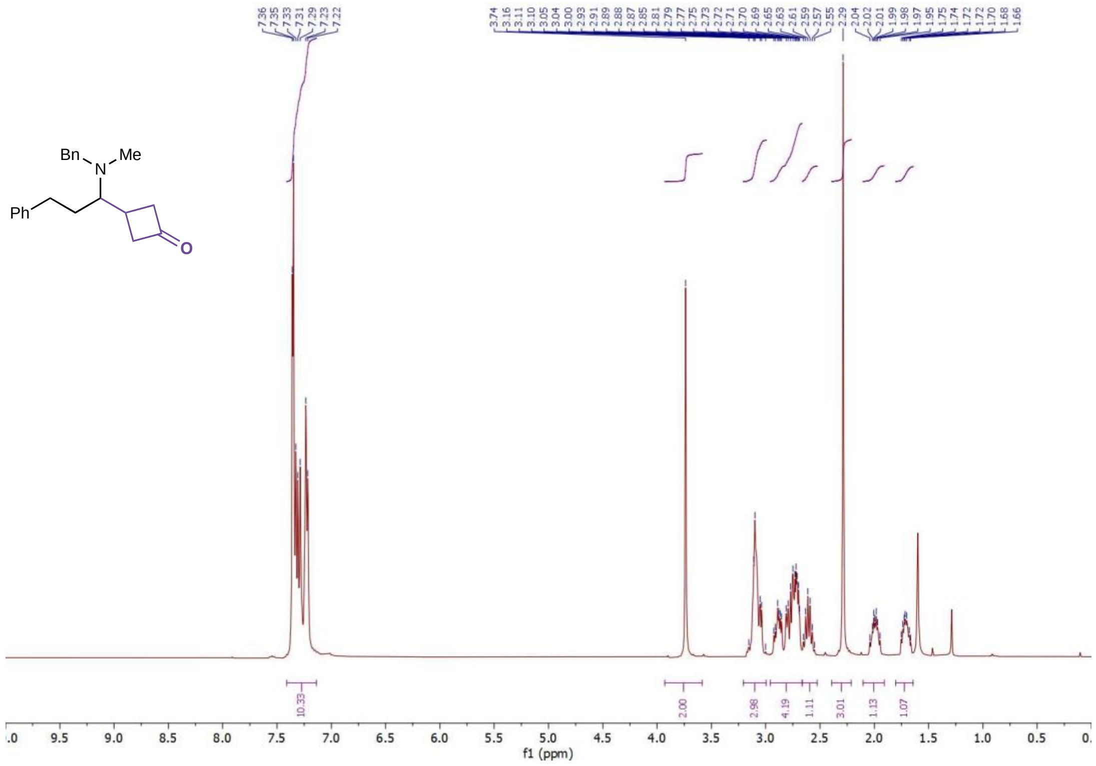

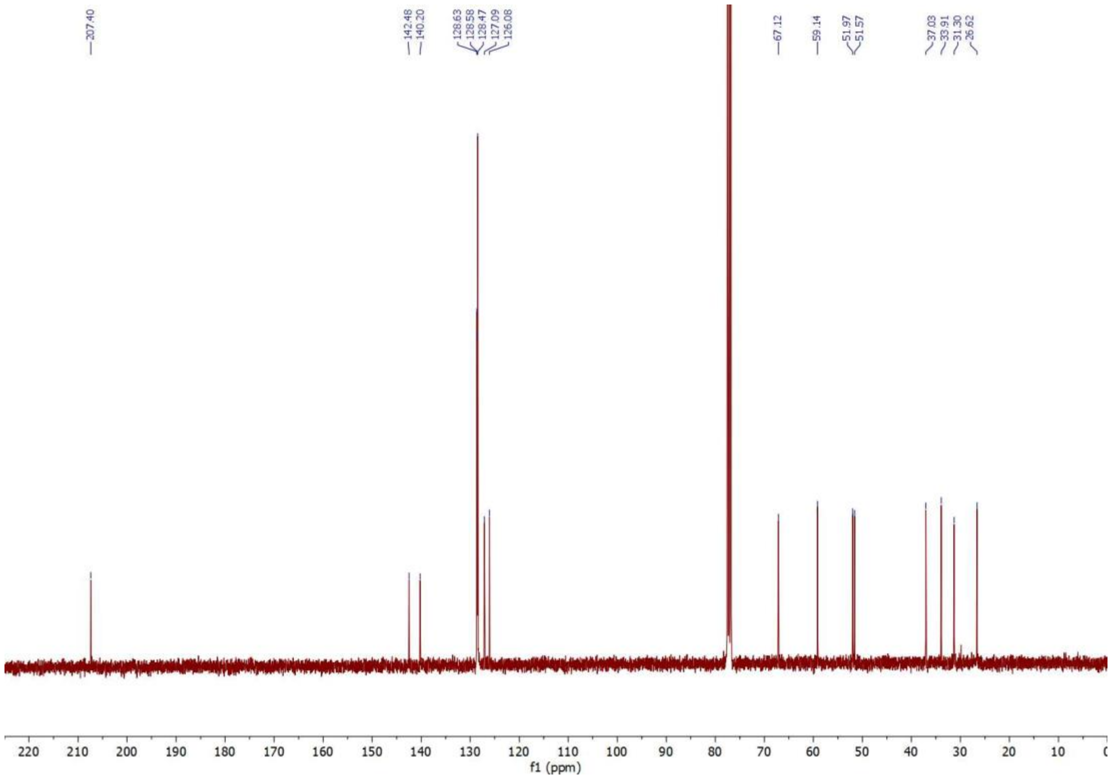

N-benzyl-1-(3,3-difluorocyclobutyl)-N-methyl-3-phenylpropan-1-amine (16f)

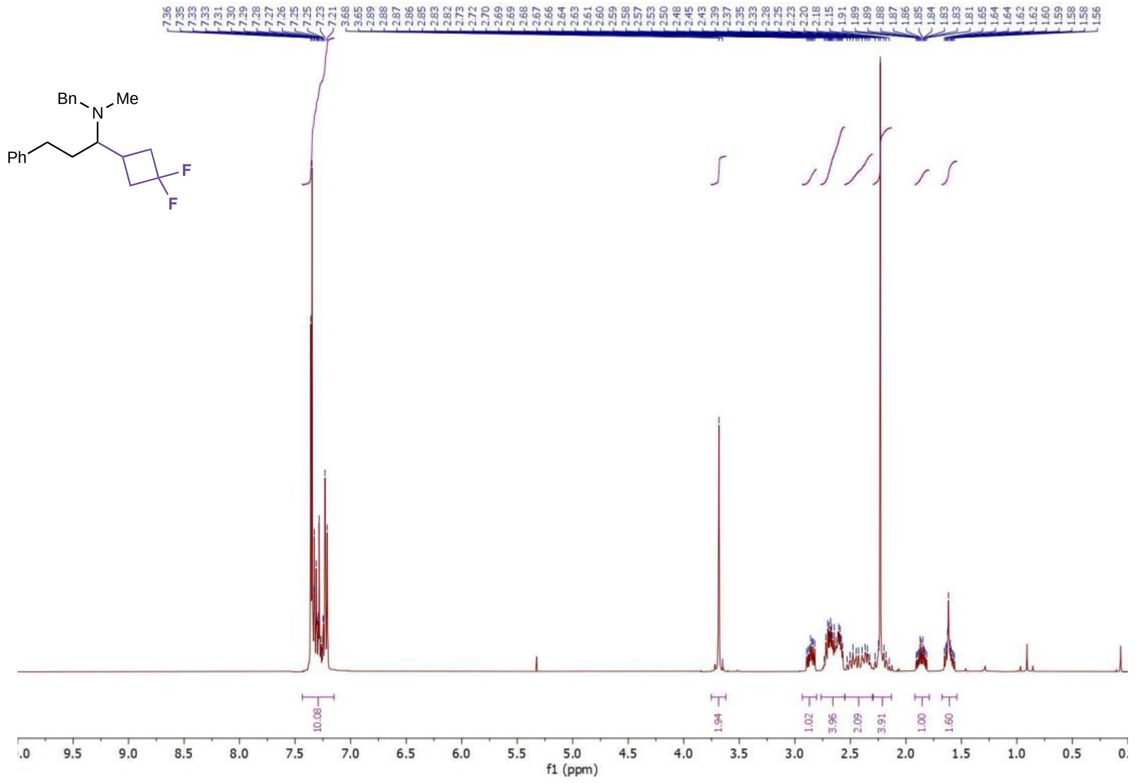

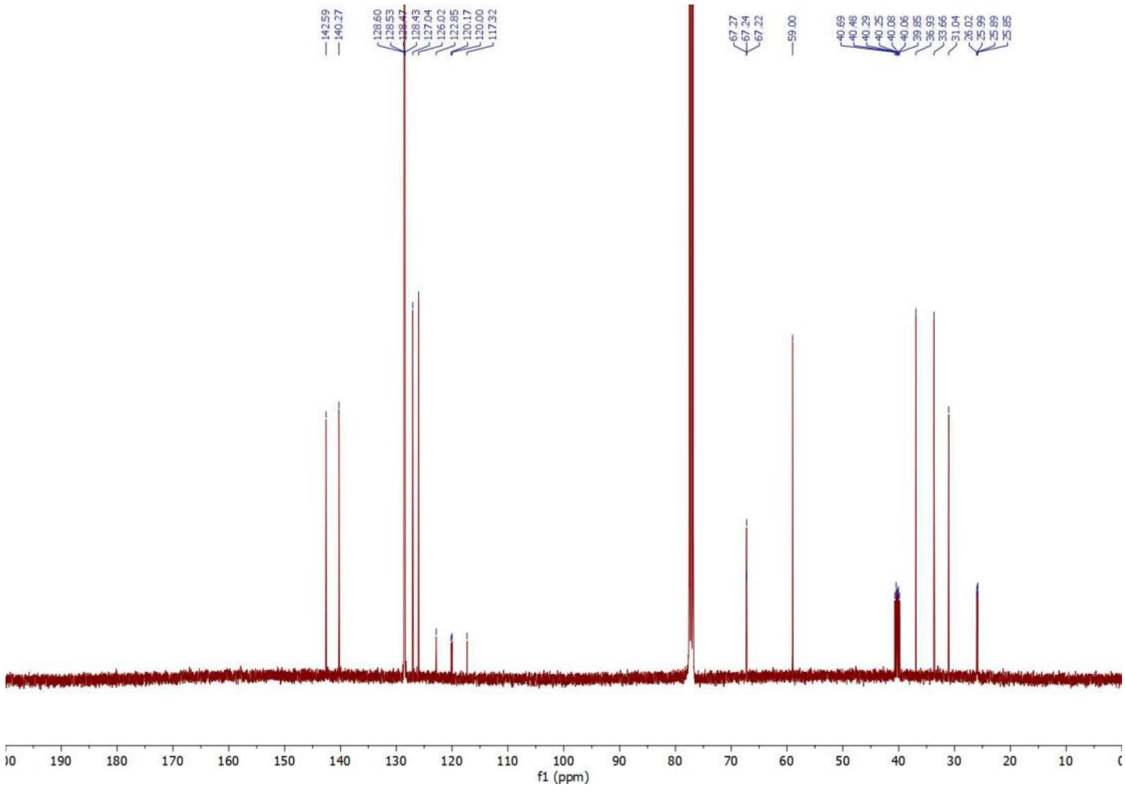

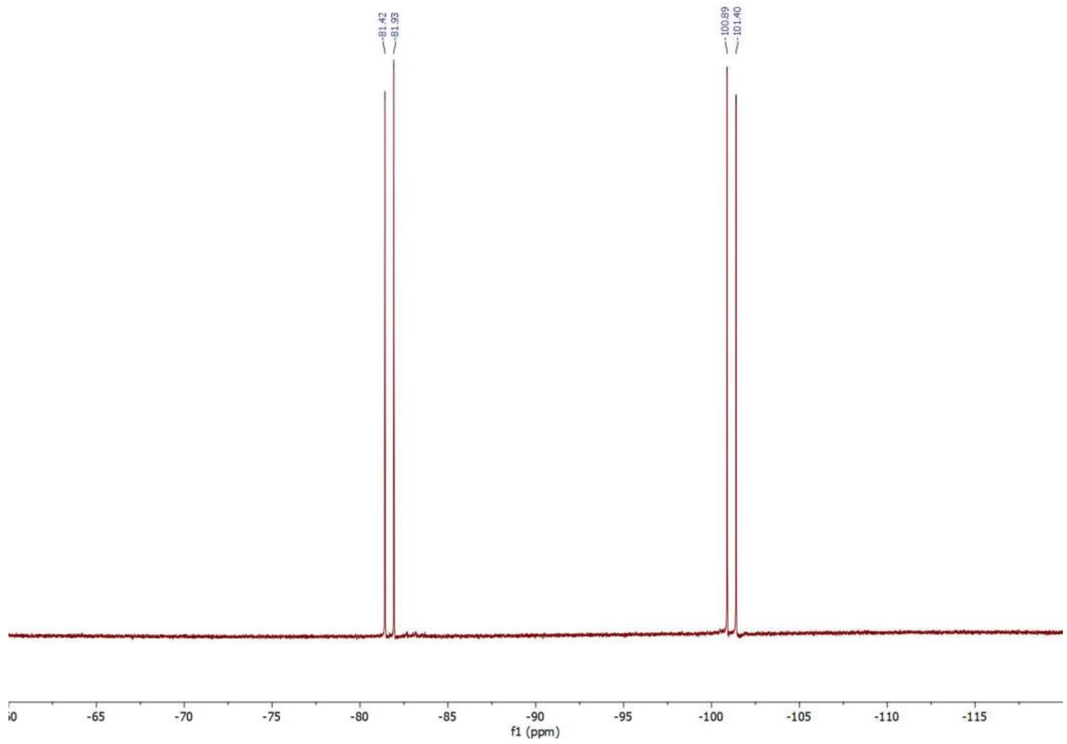

81.42
81.93
100.89
101.40
-65
-70
-75
-80
-85
-90
-95
-100
-105
-110
-115
f1 (ppm)

N-benzyl-N-methyl-1-(4-methyltetrahydro-2H-pyran-4-yl)-3-phenylpropan-1-amine (16g)

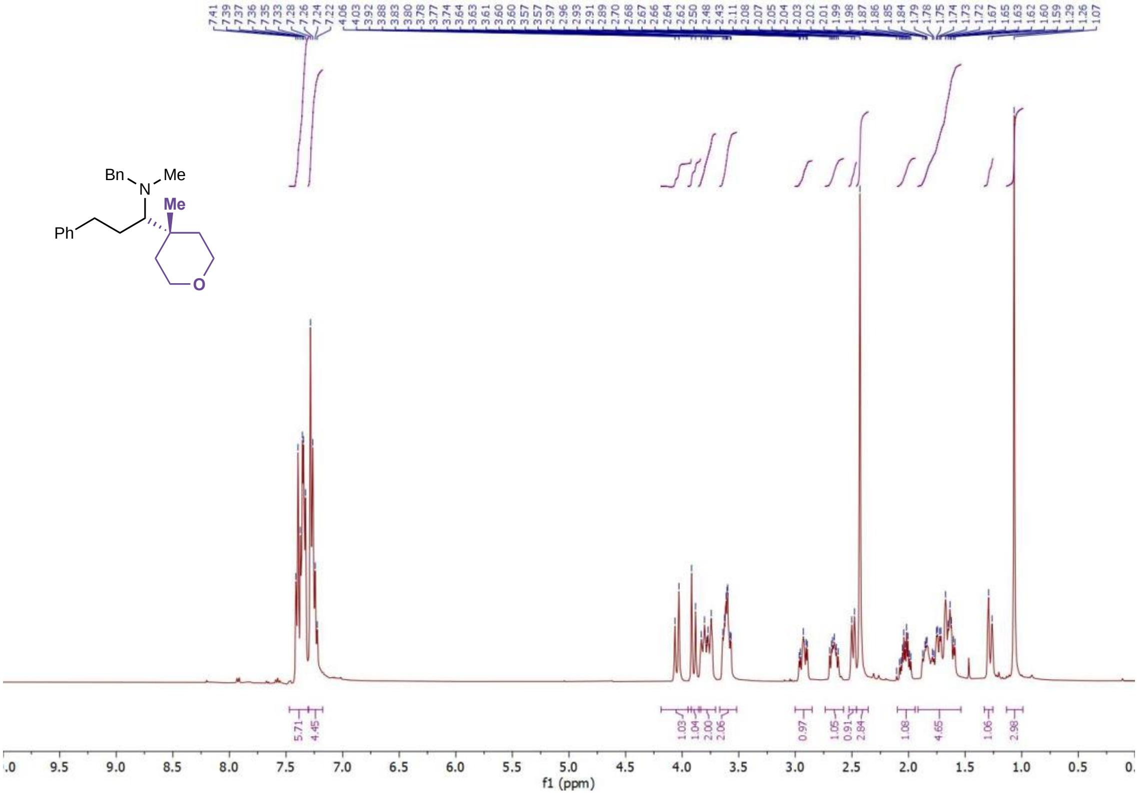

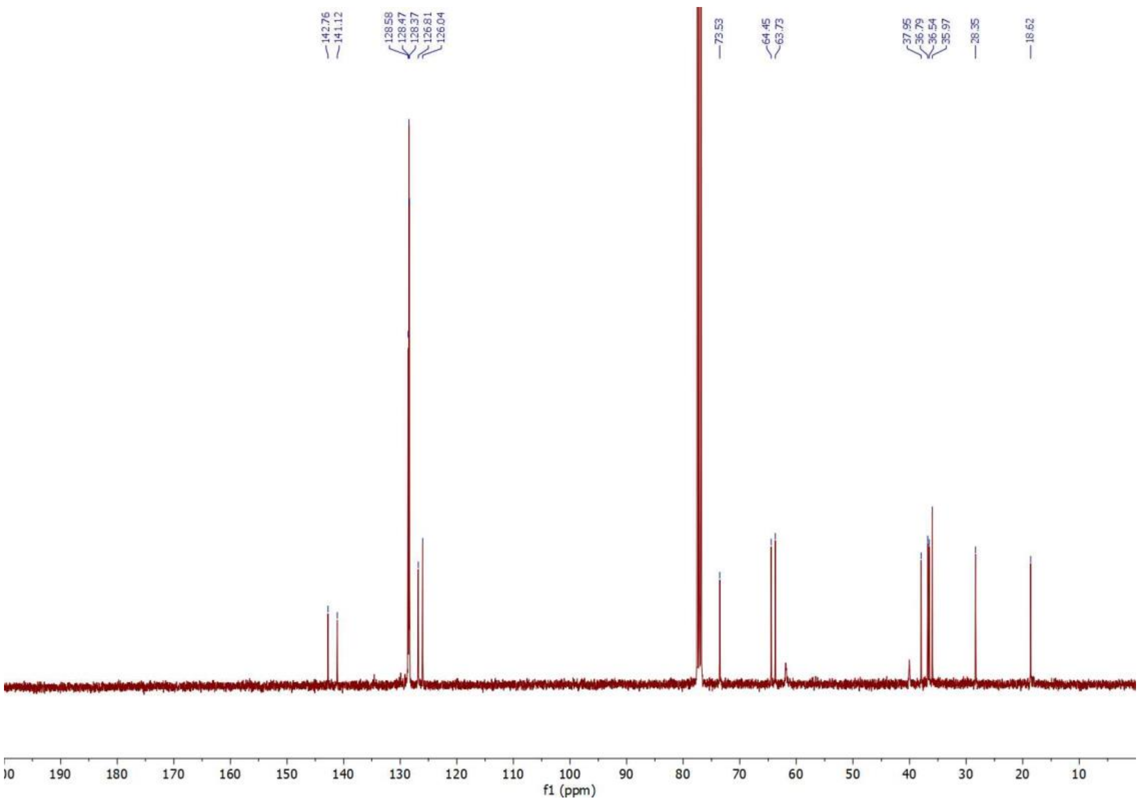

N-benzyl-1-cycloheptyl-N-methyl-3-phenylpropan-1-amine (16h)

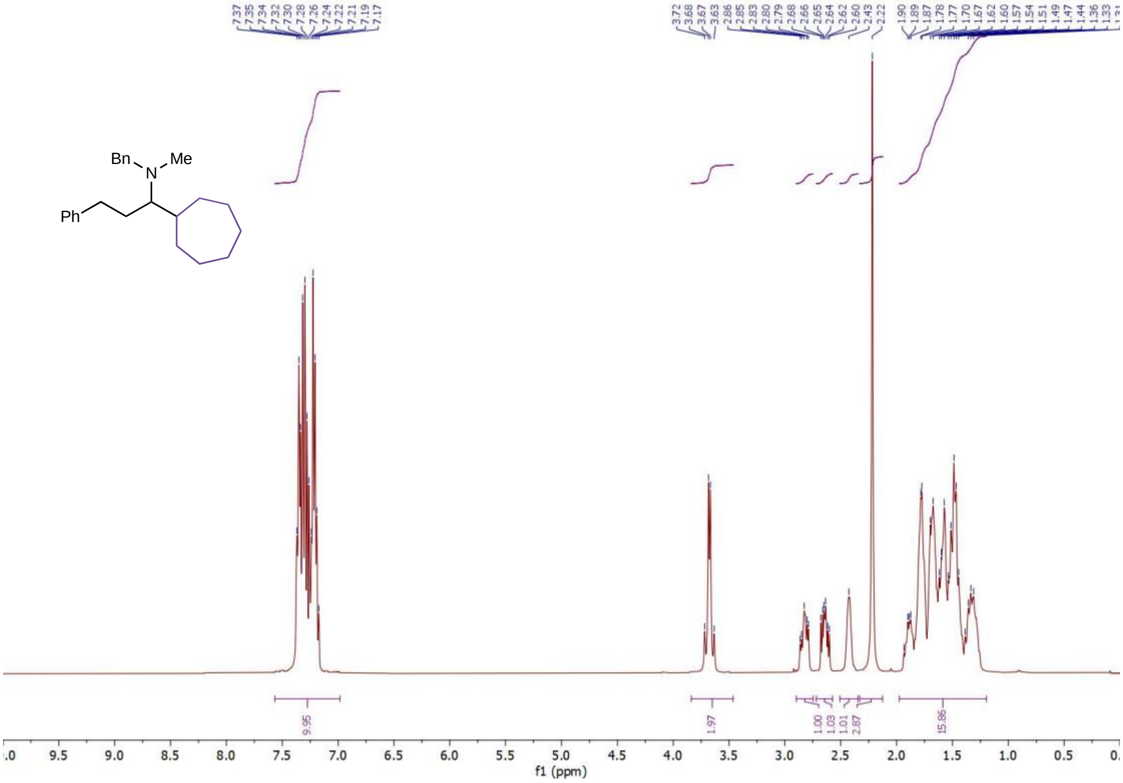

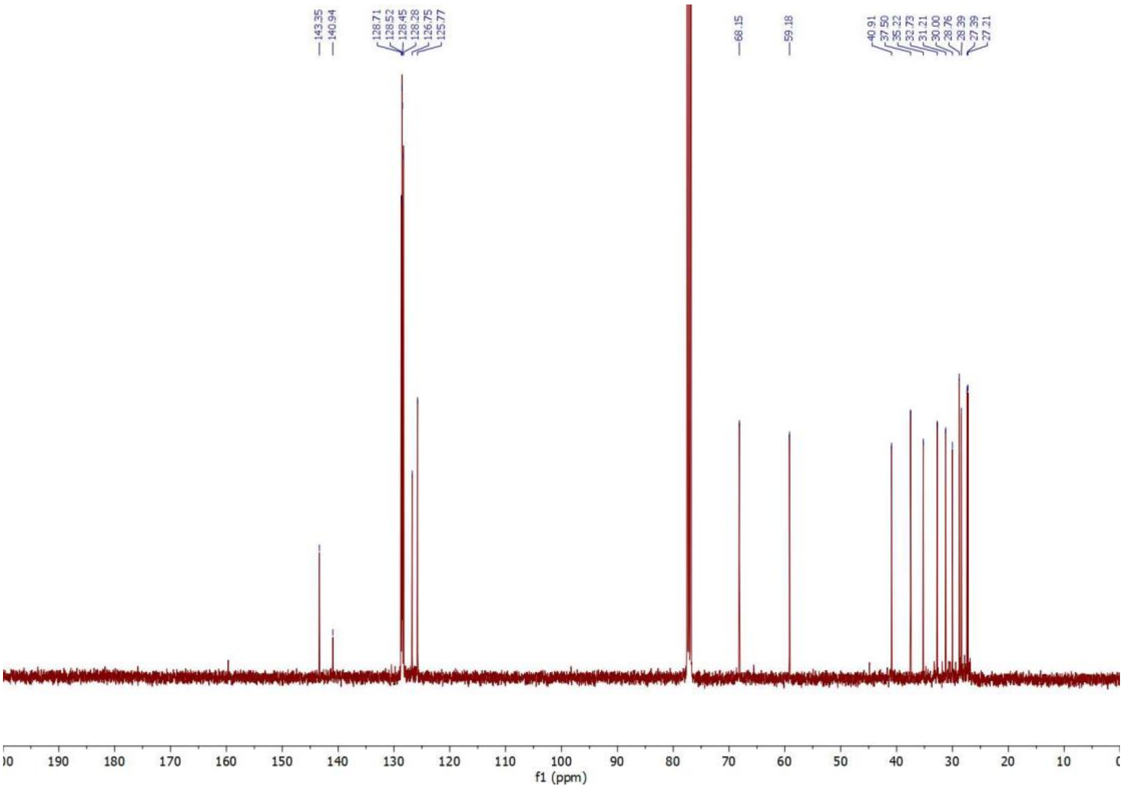

N-benzyl-1-methoxy-N-methyl-5-phenylpentan-3-amine (16i)

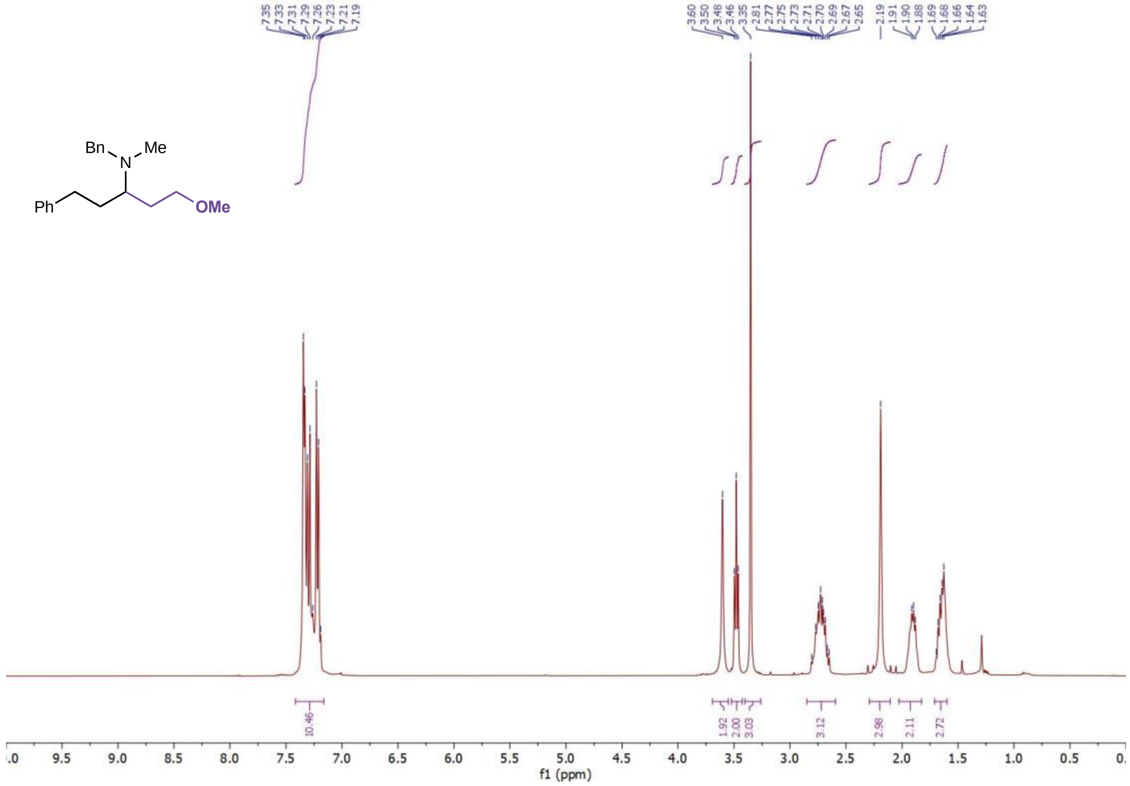

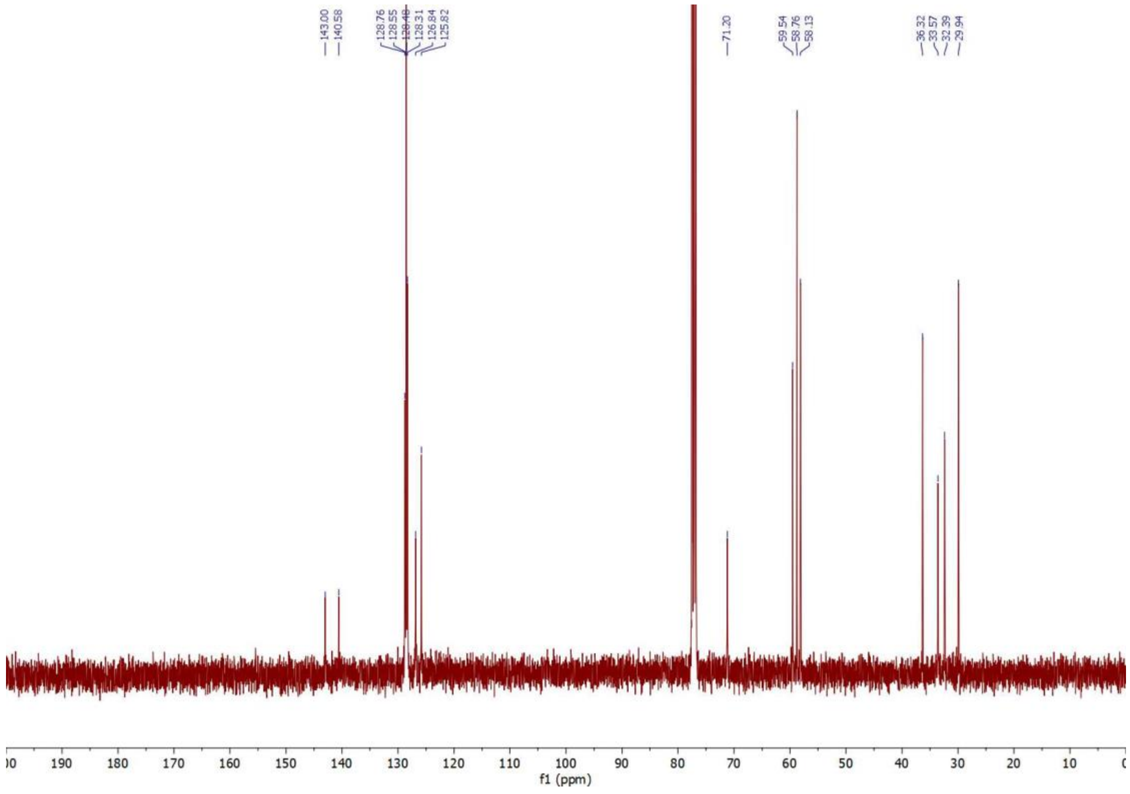

N-benzyl-1-(benzyloxy)-N-methylbutan-2-amine (16j)

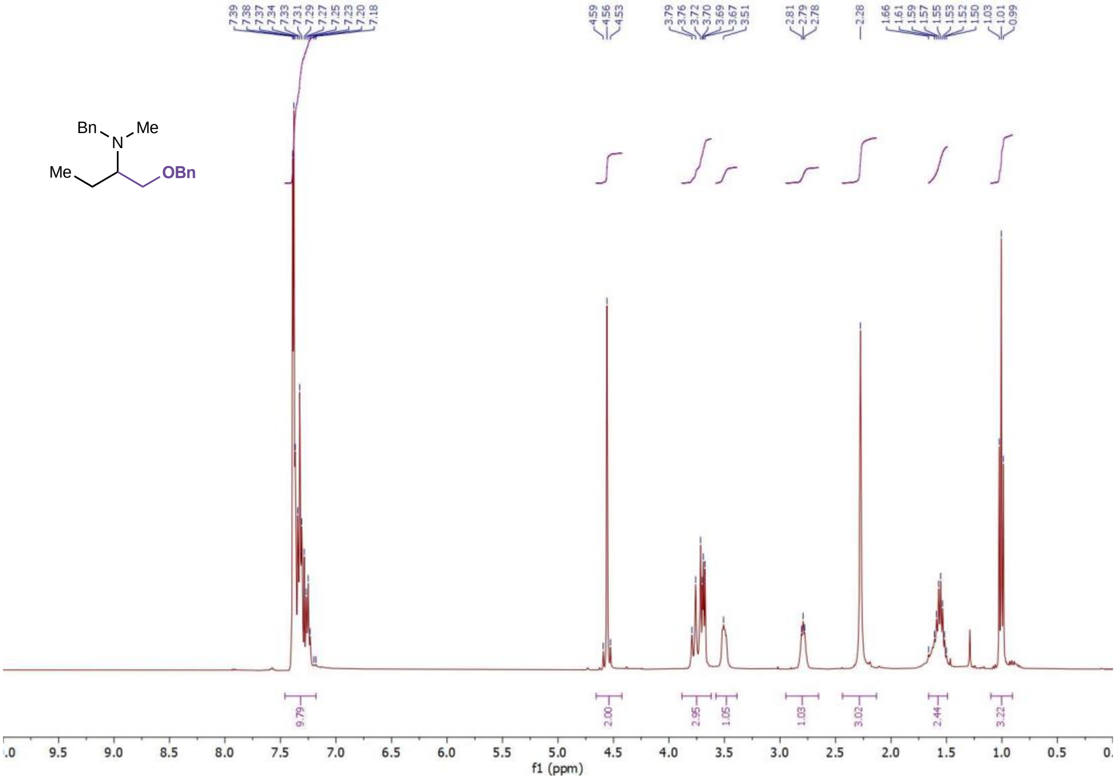

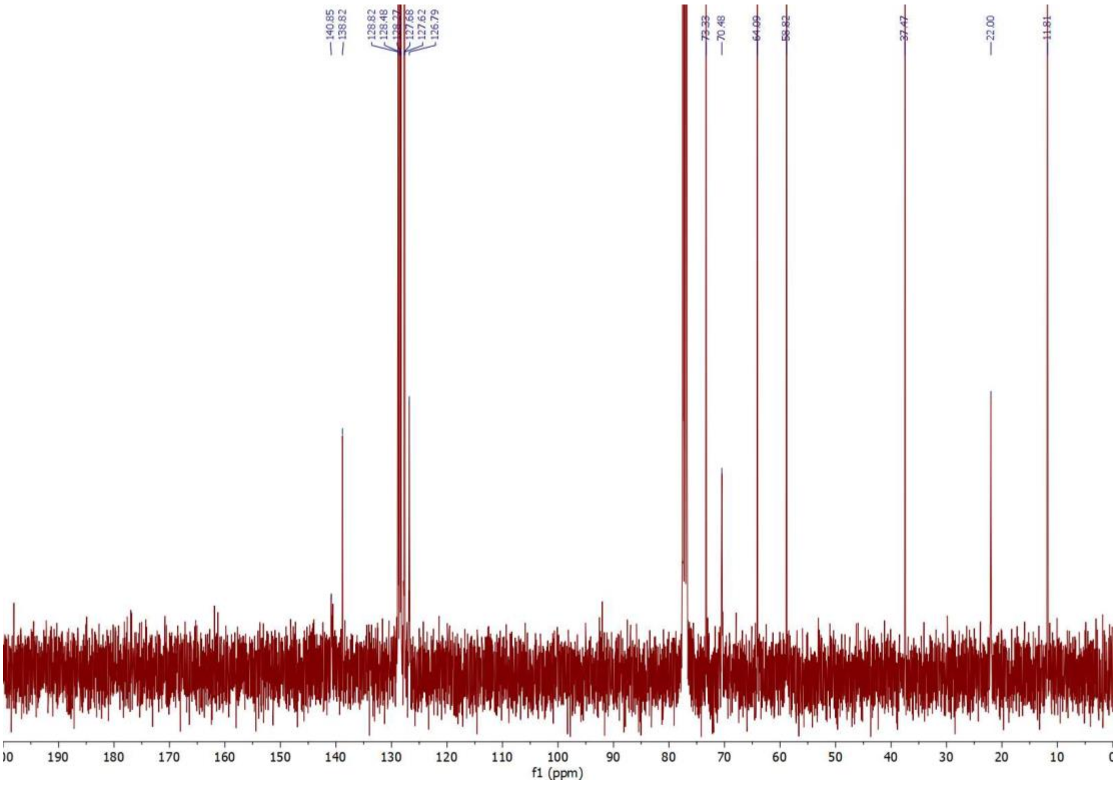

2-(2-(benzyl(methyl)amino)-4-phenylbutyl)isoindoline-1,3-dione (16k)

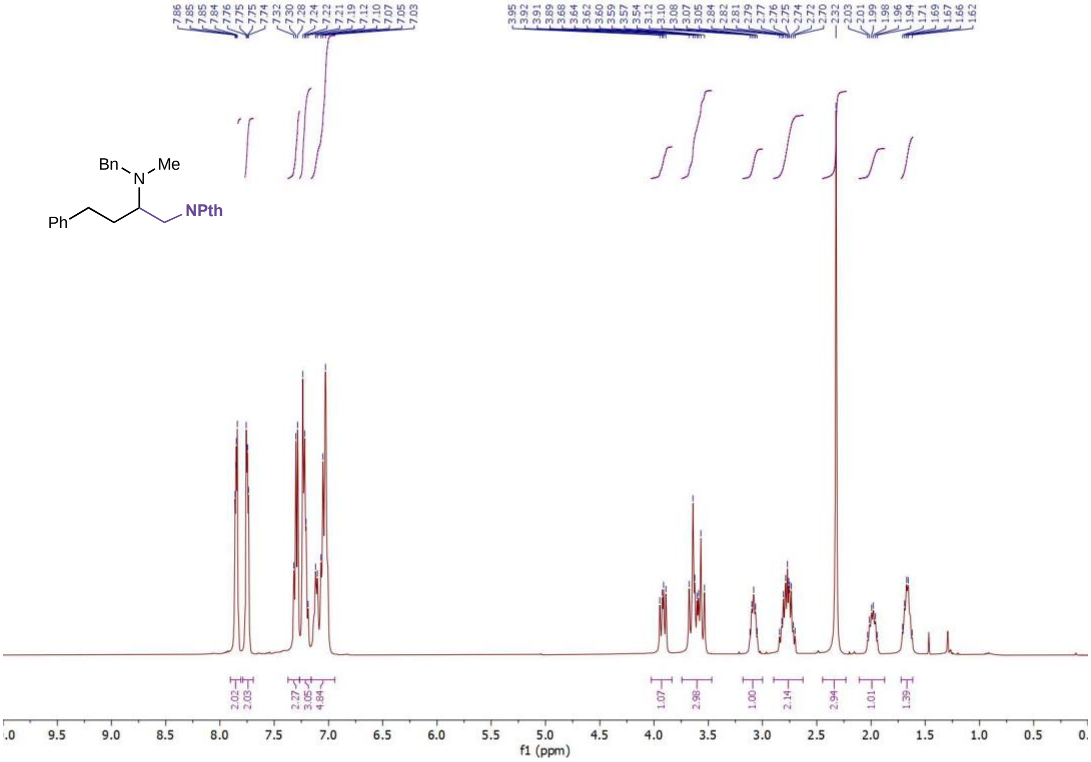

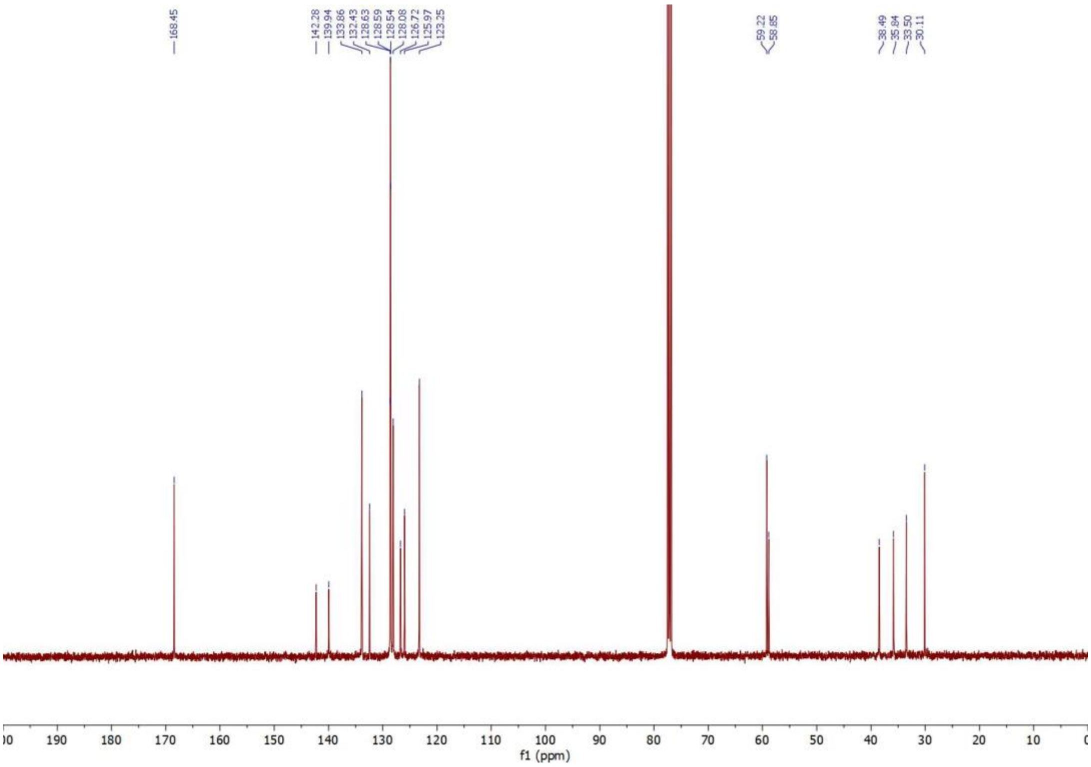

N-benzyl-N-methyl-1-(methylthio)-4-phenylbutan-2-amine (16l)

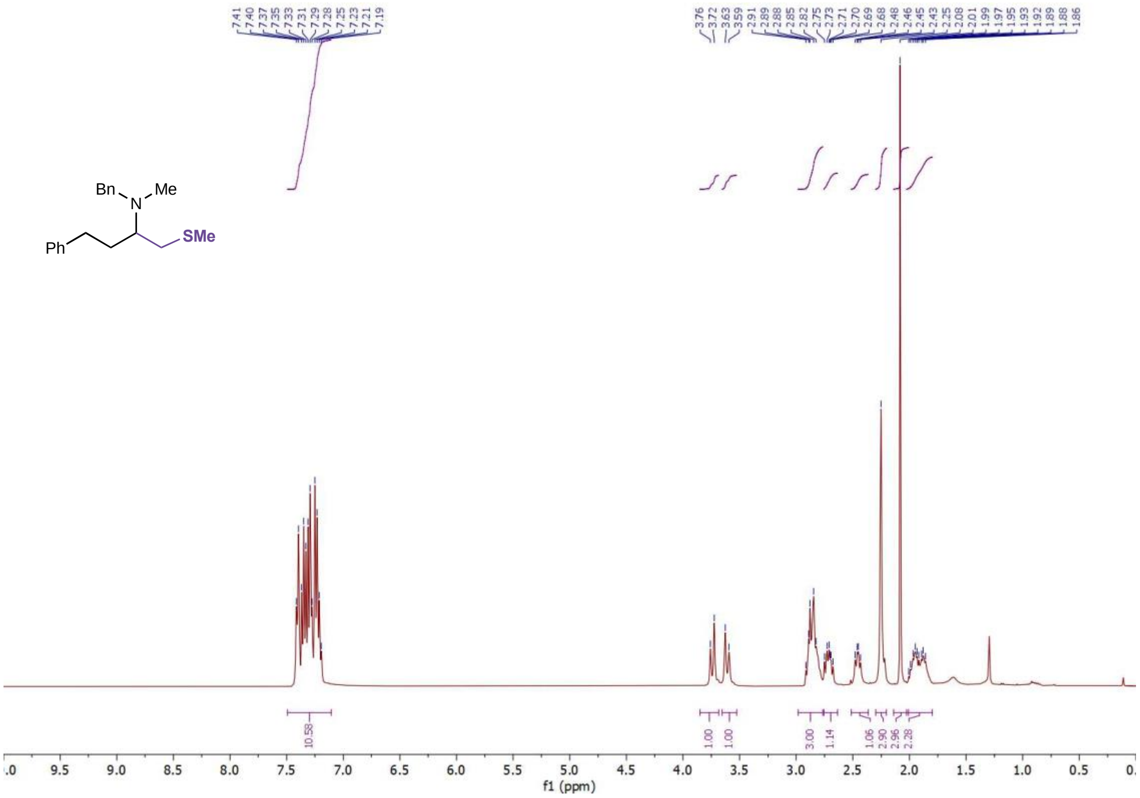

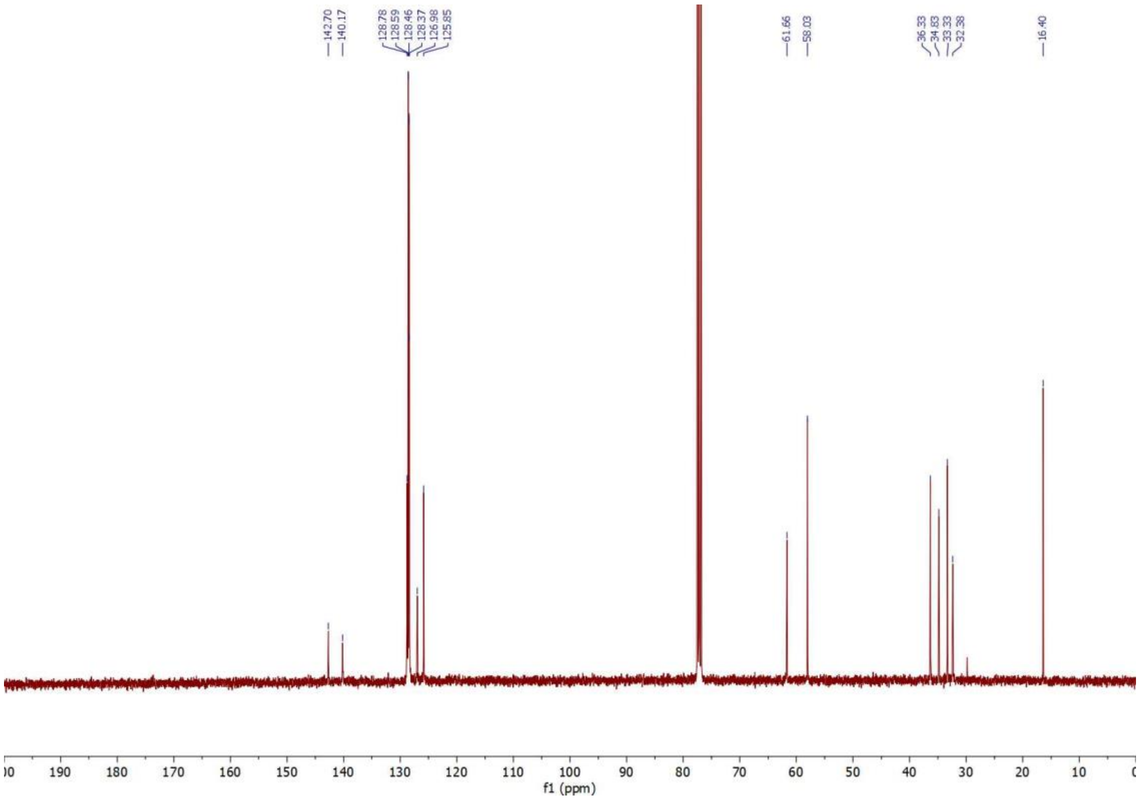

N-benzyl-N-methyl-3-phenyl-1-(tetrahydrofuran-2-yl)propan-1-amine (16m) single diastereomer

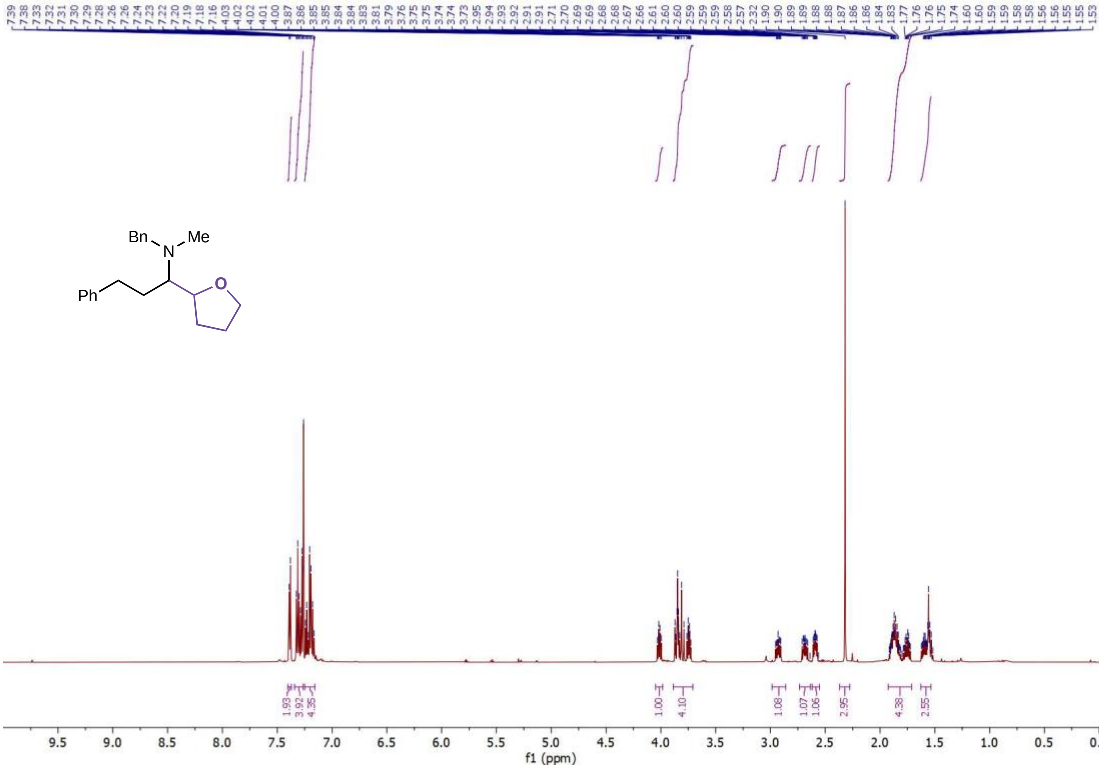

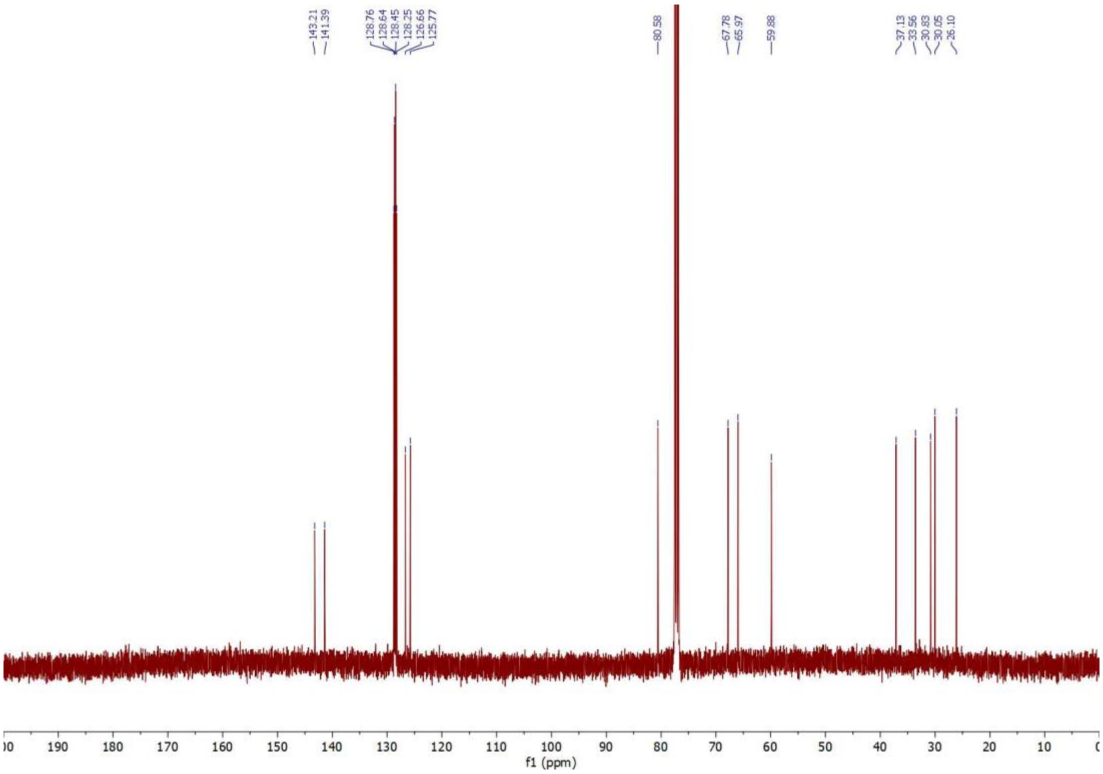

N-benzyl-4-fluoro-N,4-dimethyl-1-phenylpentan-3-amine (16n)

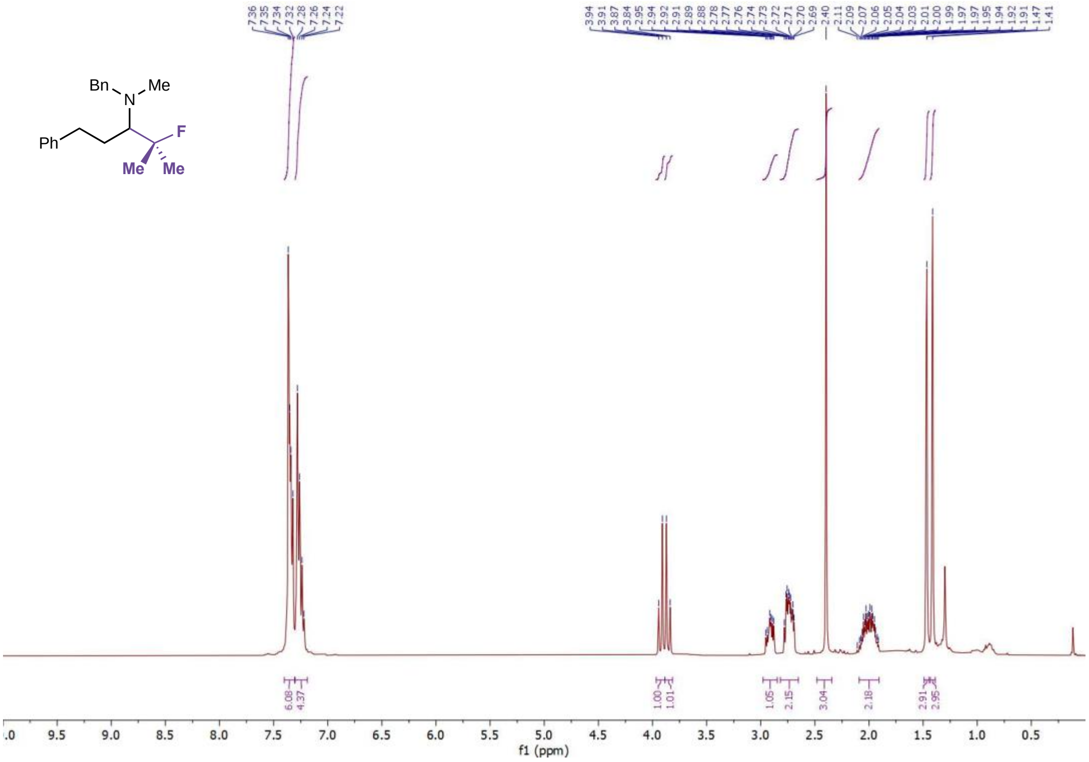

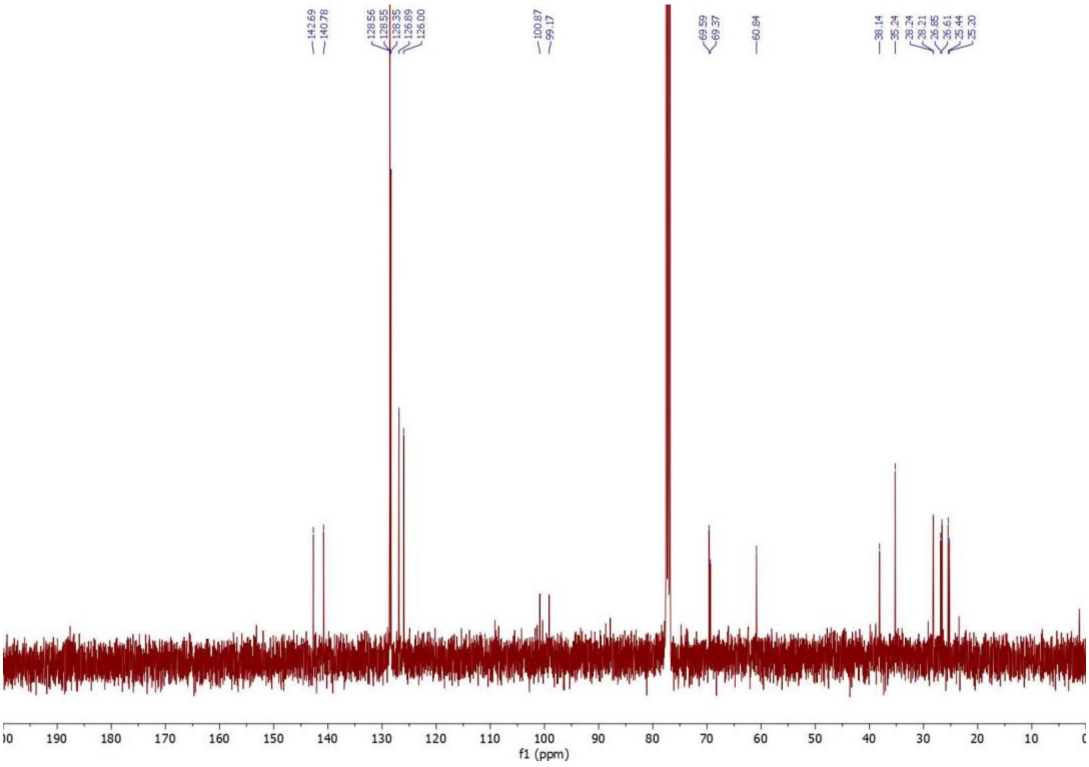

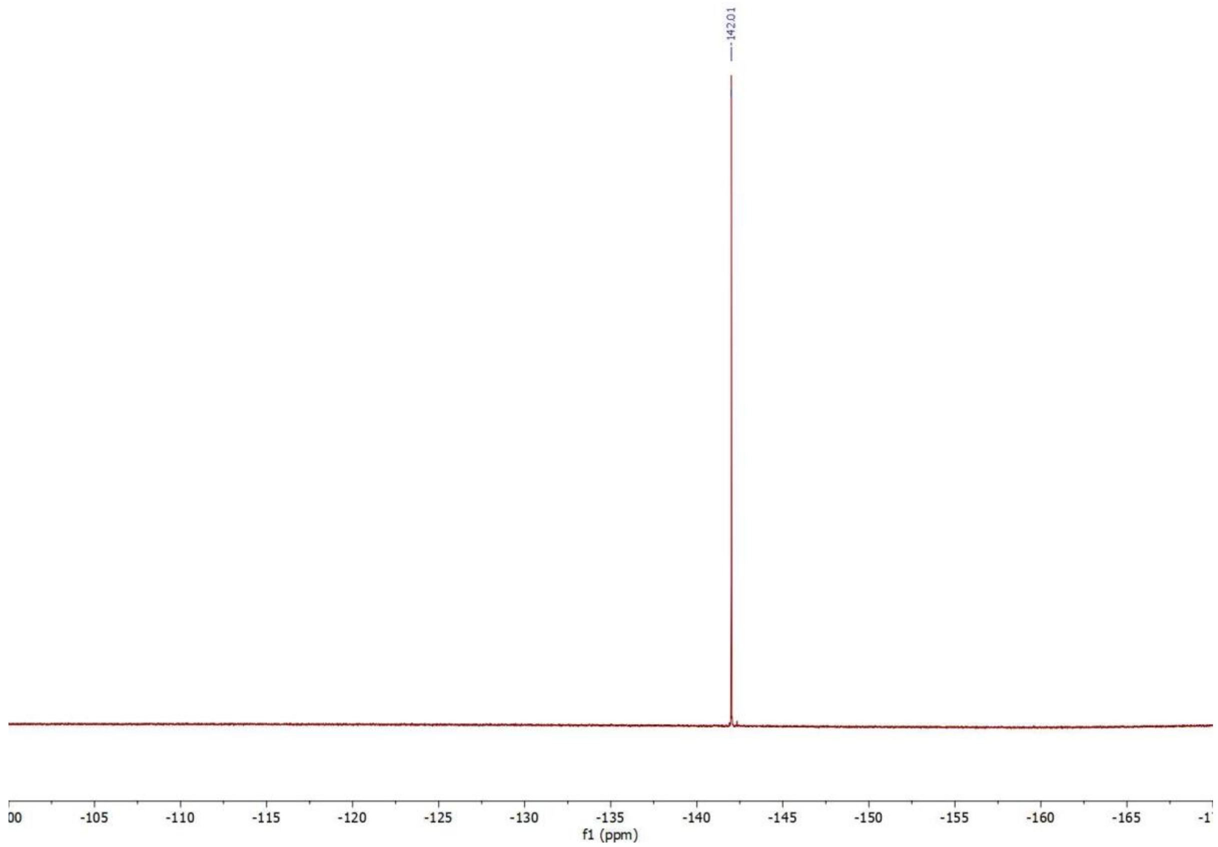
-142.01
00
-105
-110
-115
-120
-125
-130
-135
-140
-145
-150
-155
-160
-165
f1 (ppm)

N-(2,3-dimethylbutan-2-yl)-4-methoxyaniline (17a)

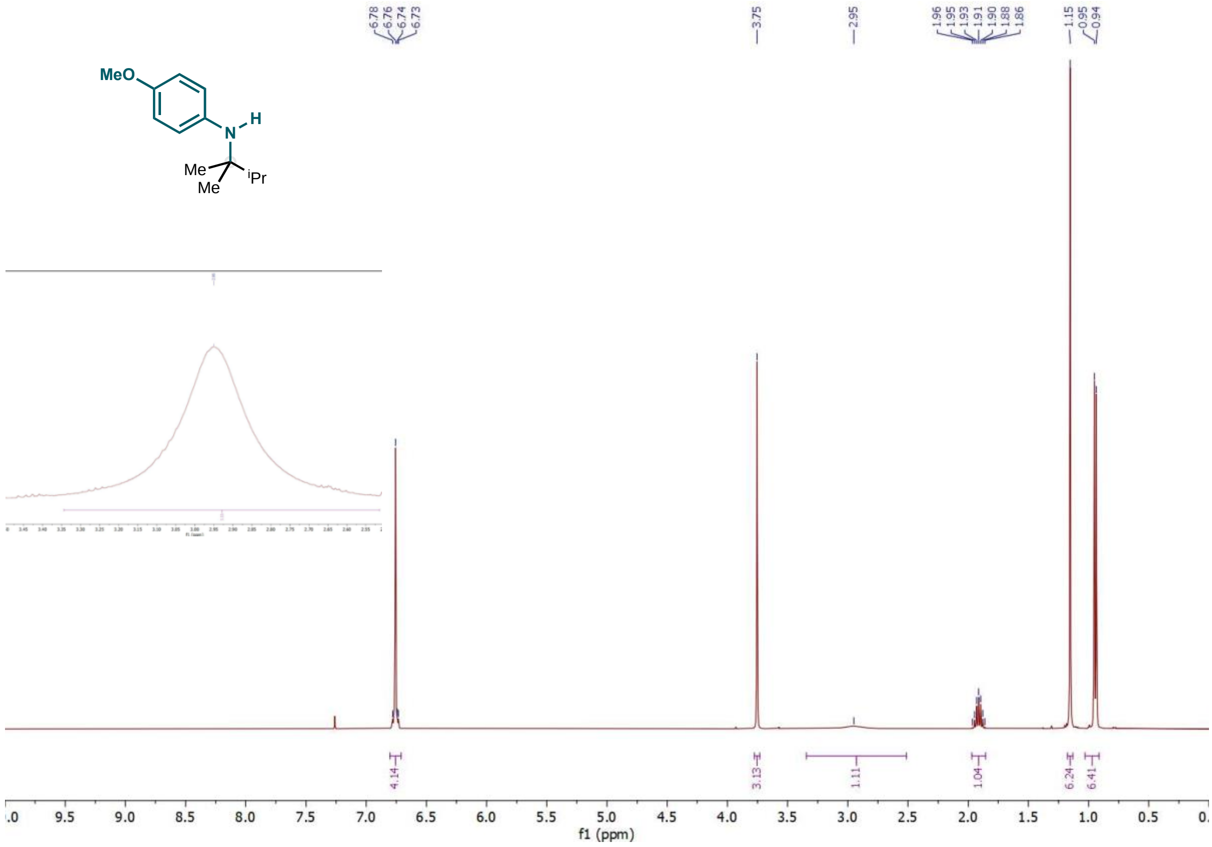

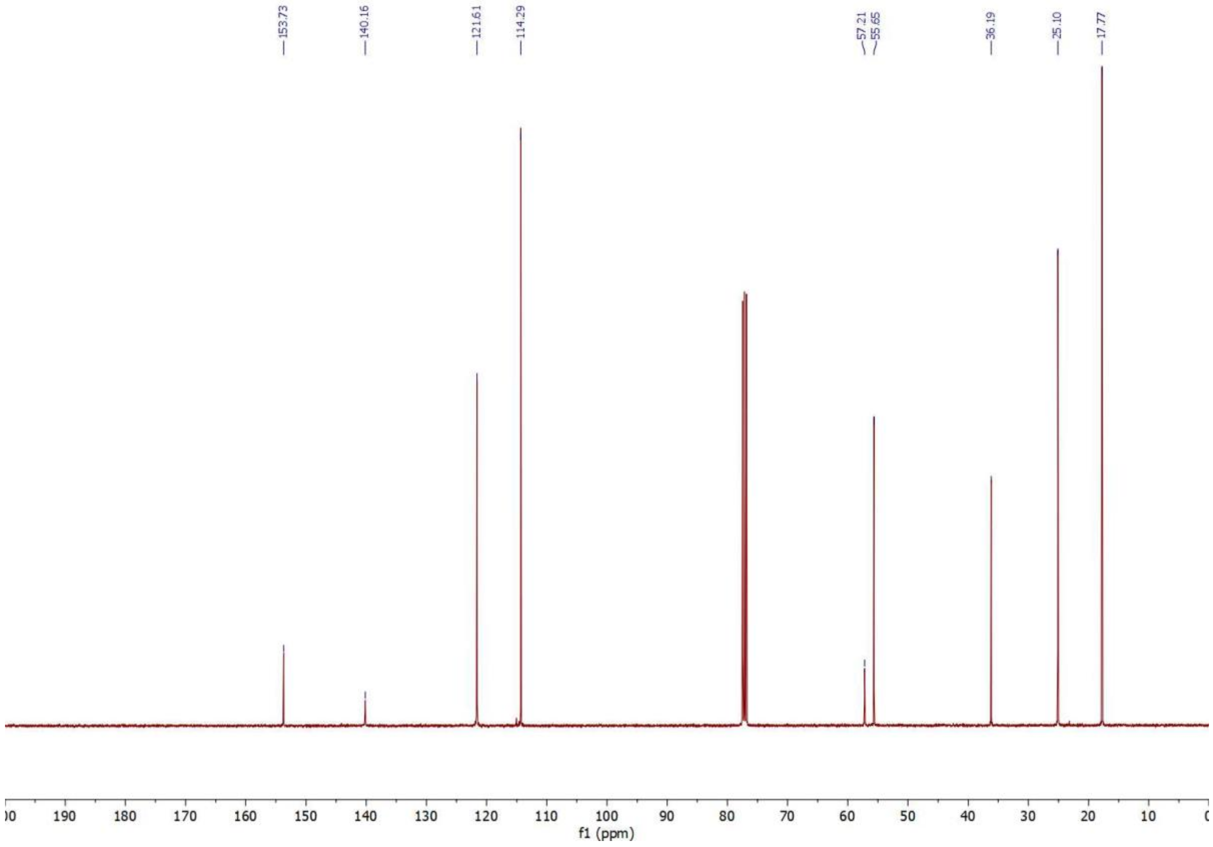

N-(2,3-dimethylbutan-2-yl)aniline (17b)

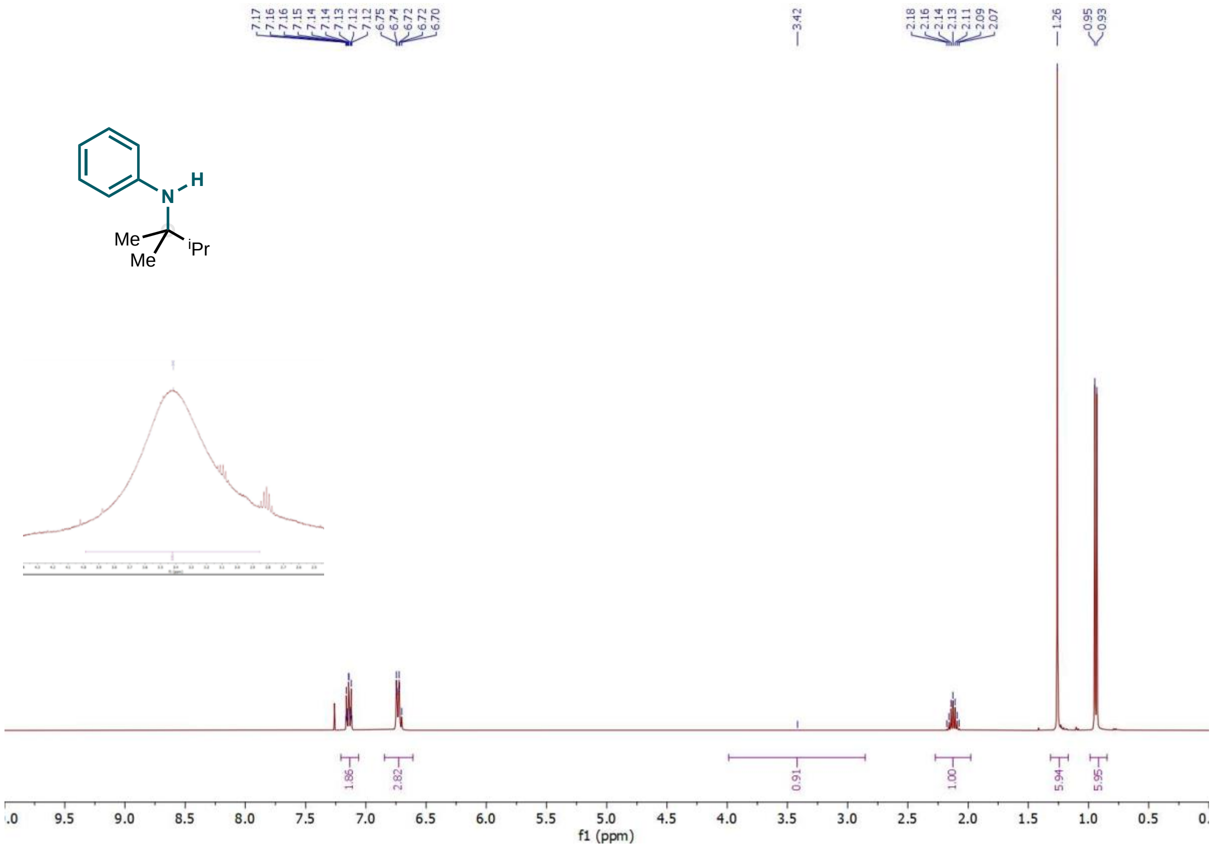

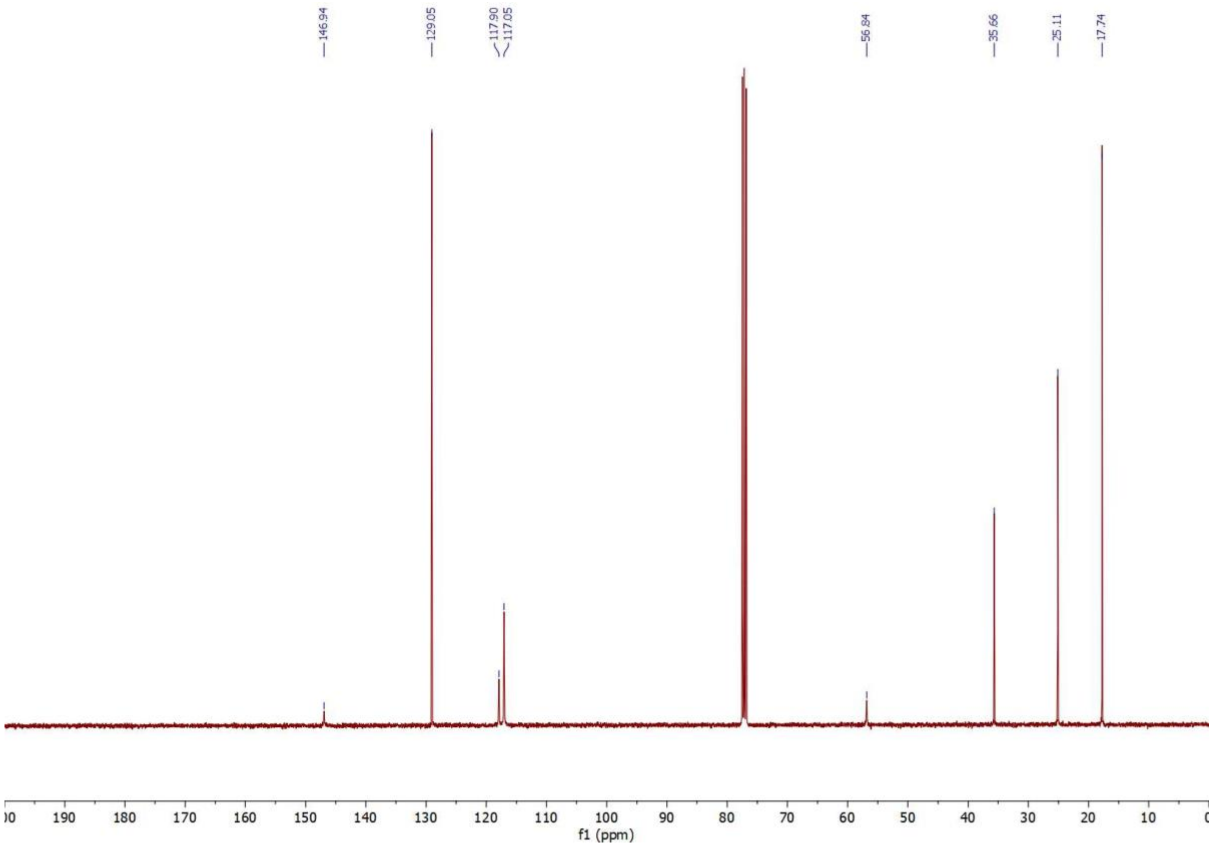

4-bromo-N-(2,3-dimethylbutan-2-yl)aniline (17c)

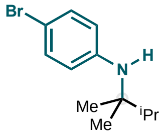

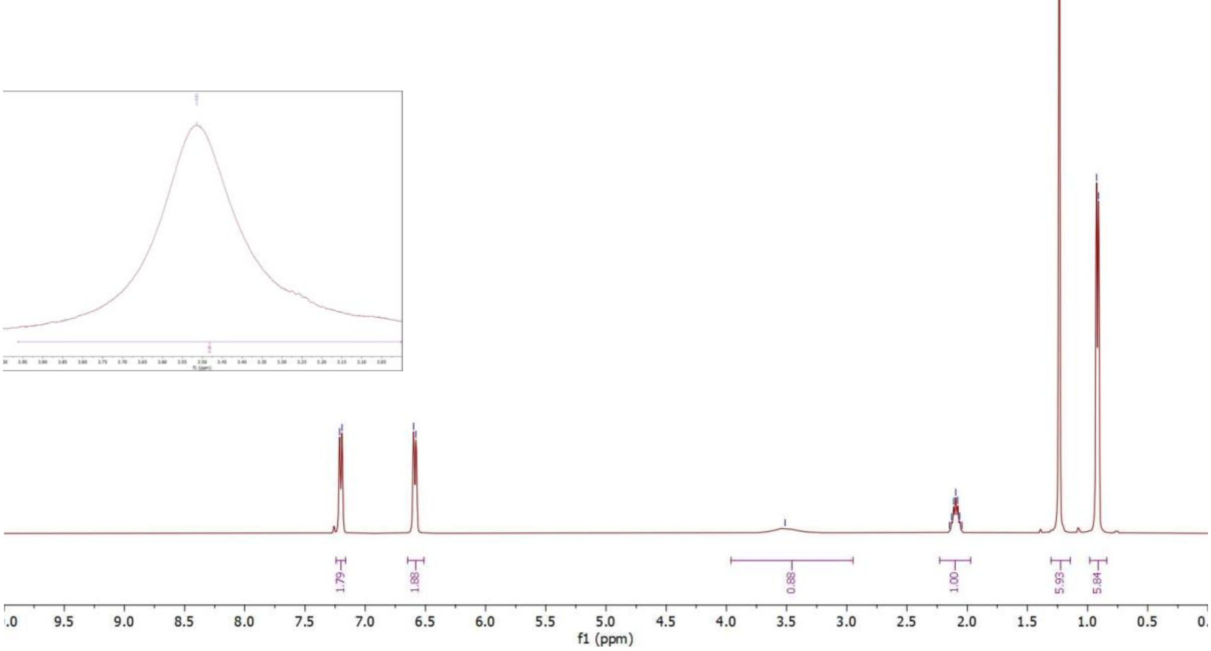

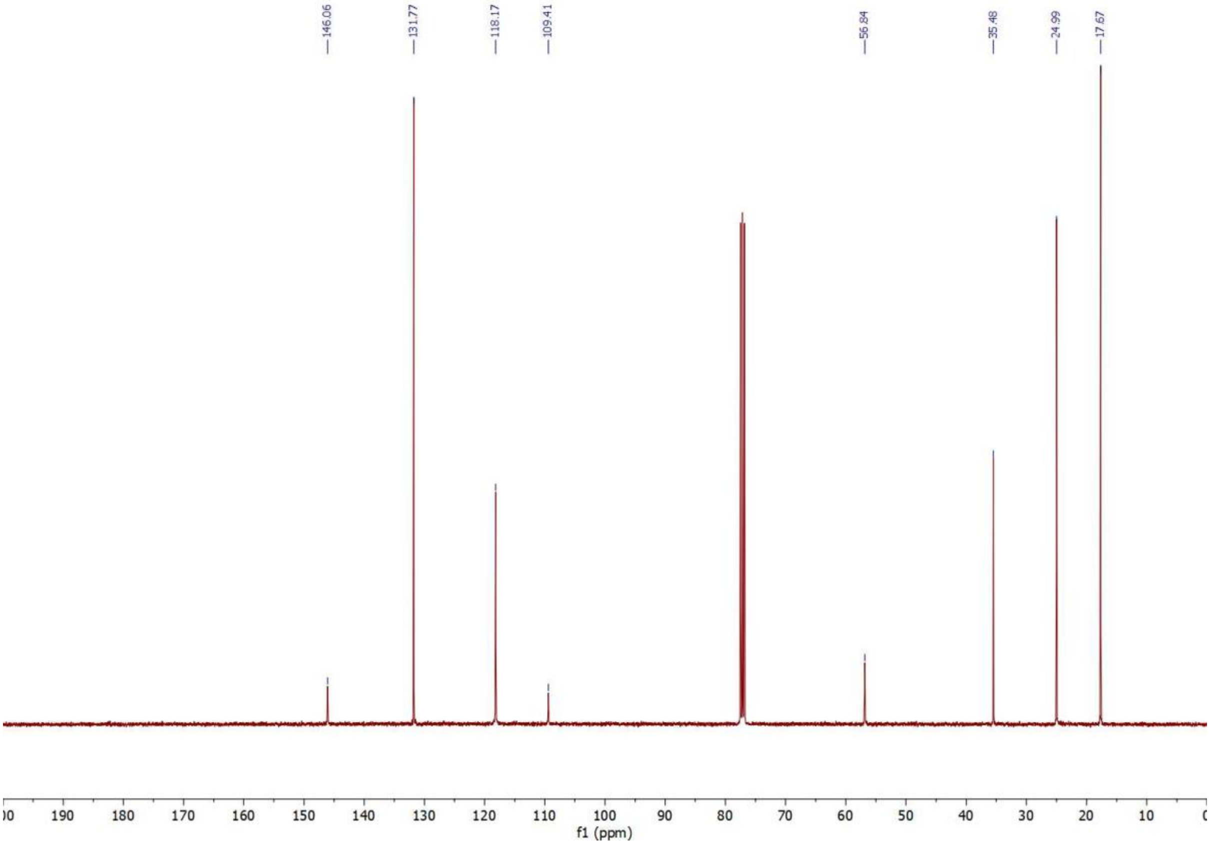

N-(2,3-dimethylbutan-2-yl)-4-iodoaniline (17d)

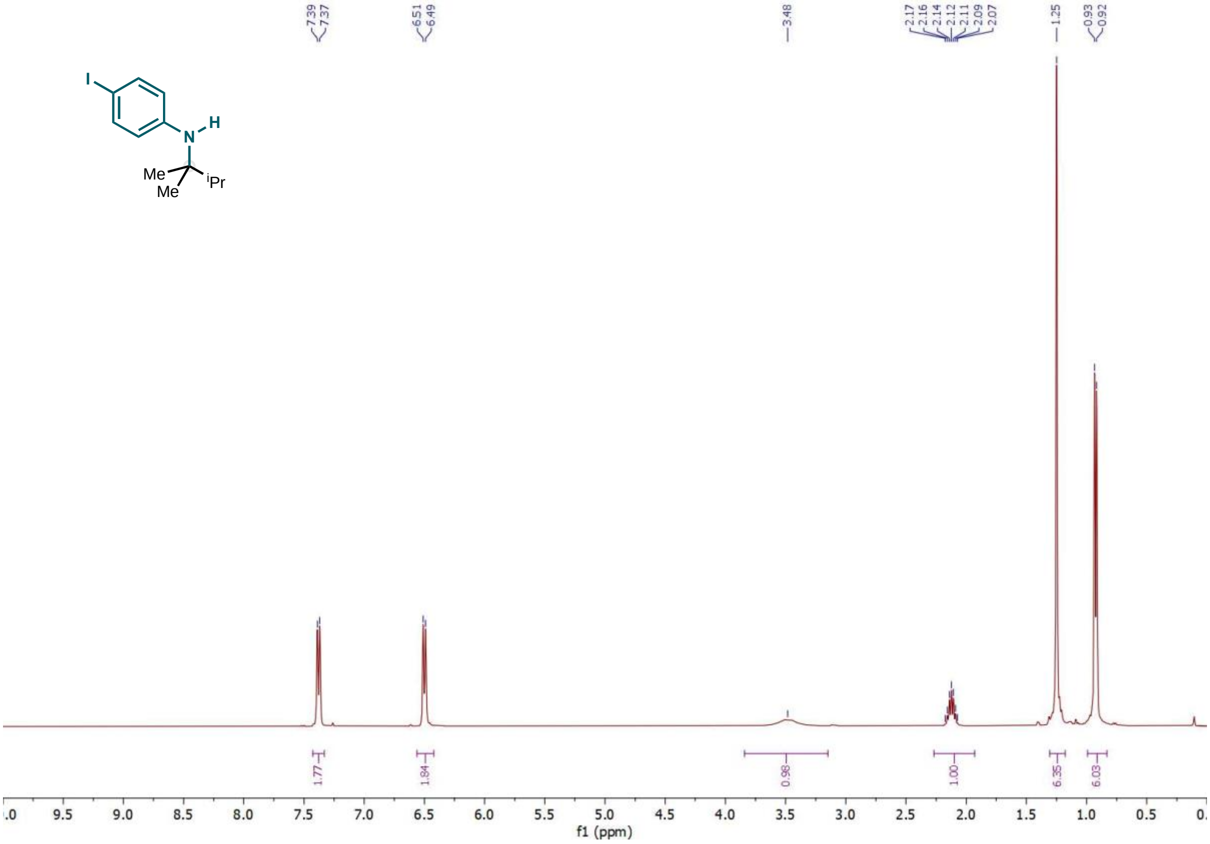

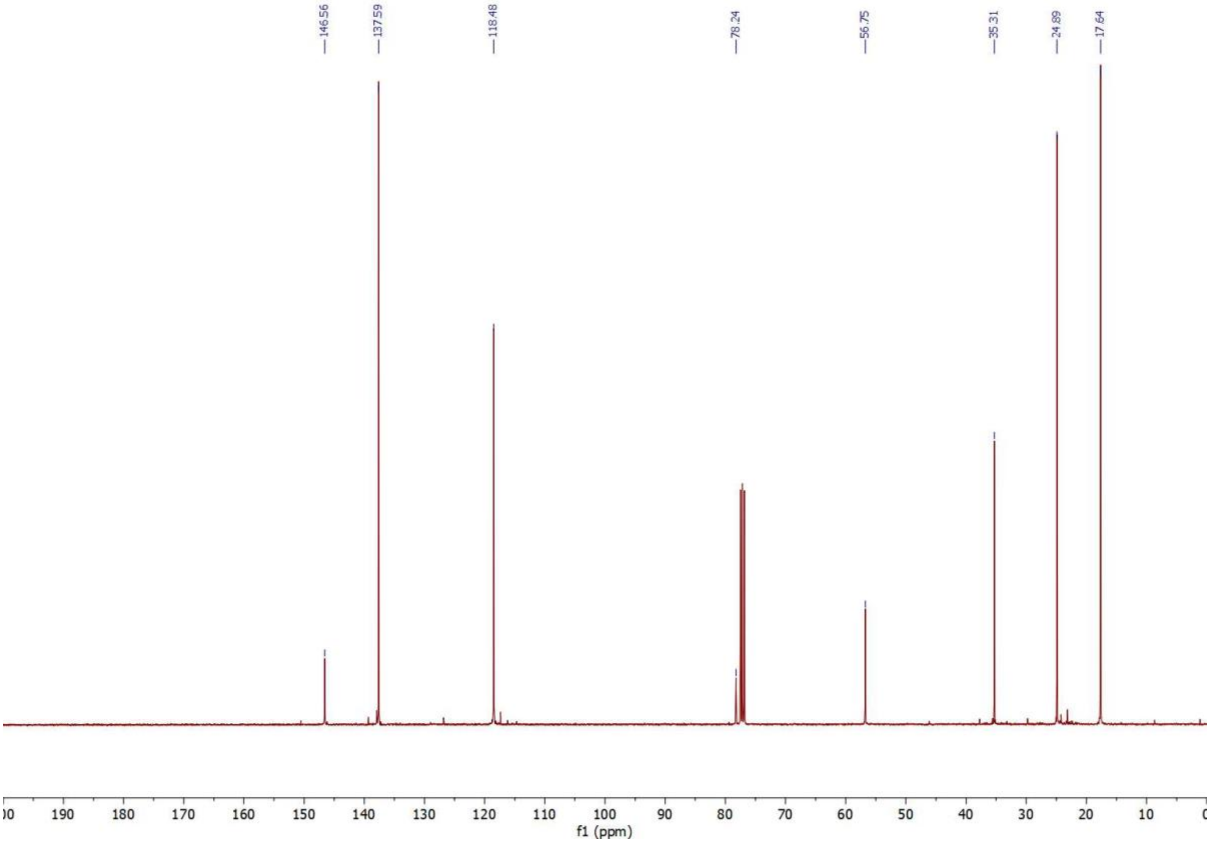

Ethyl 3-((2,3-dimethylbutan-2-yl)amino)benzoate (17e)

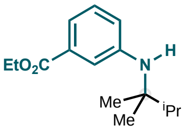

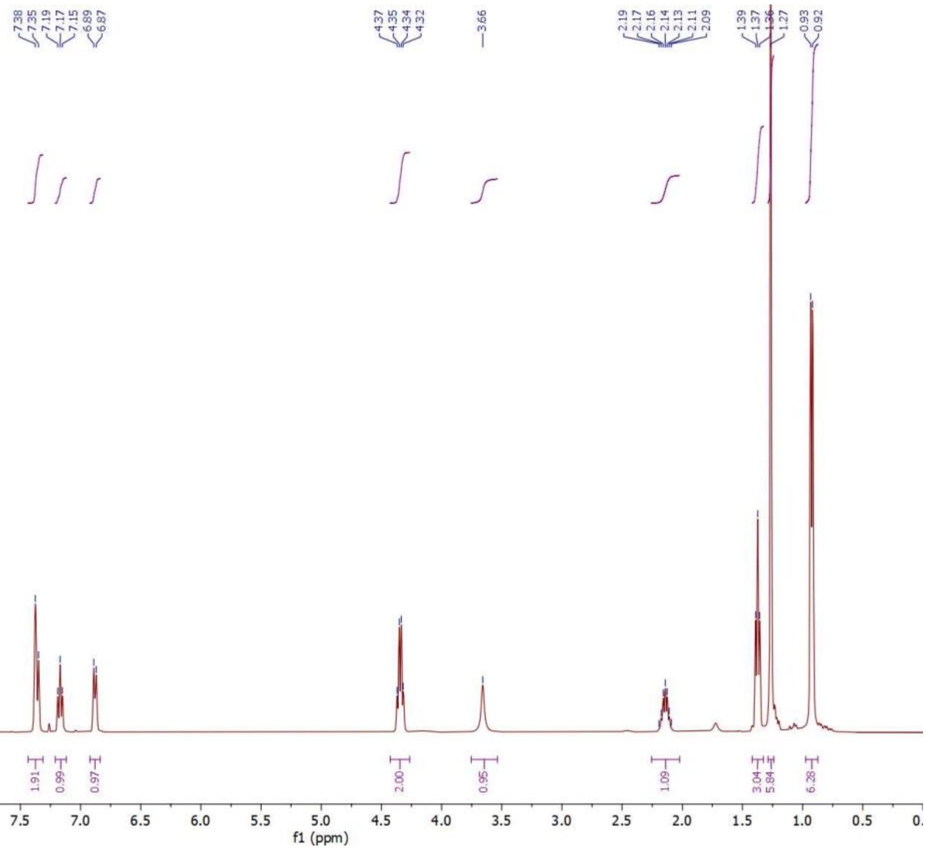

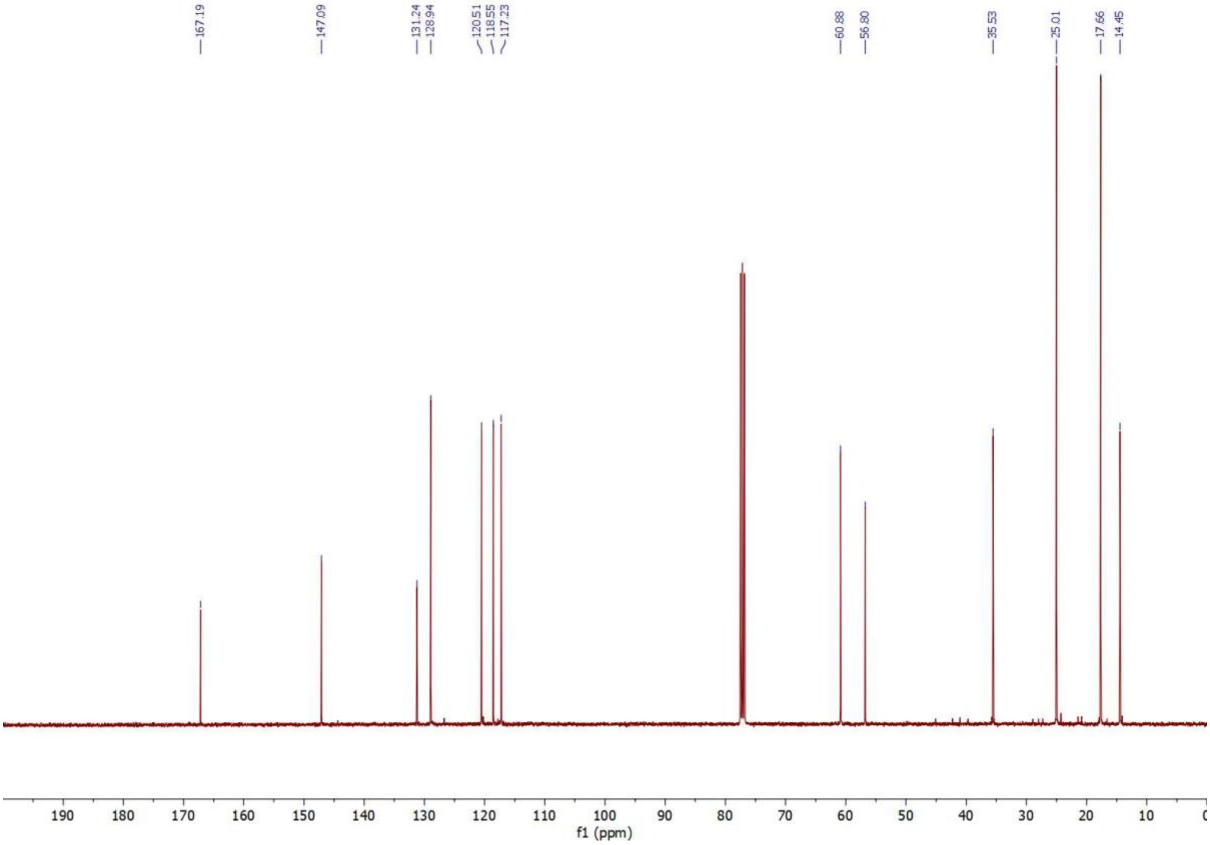

3-bromo-N-(2,3-dimethylbutan-2-yl)aniline (17f)

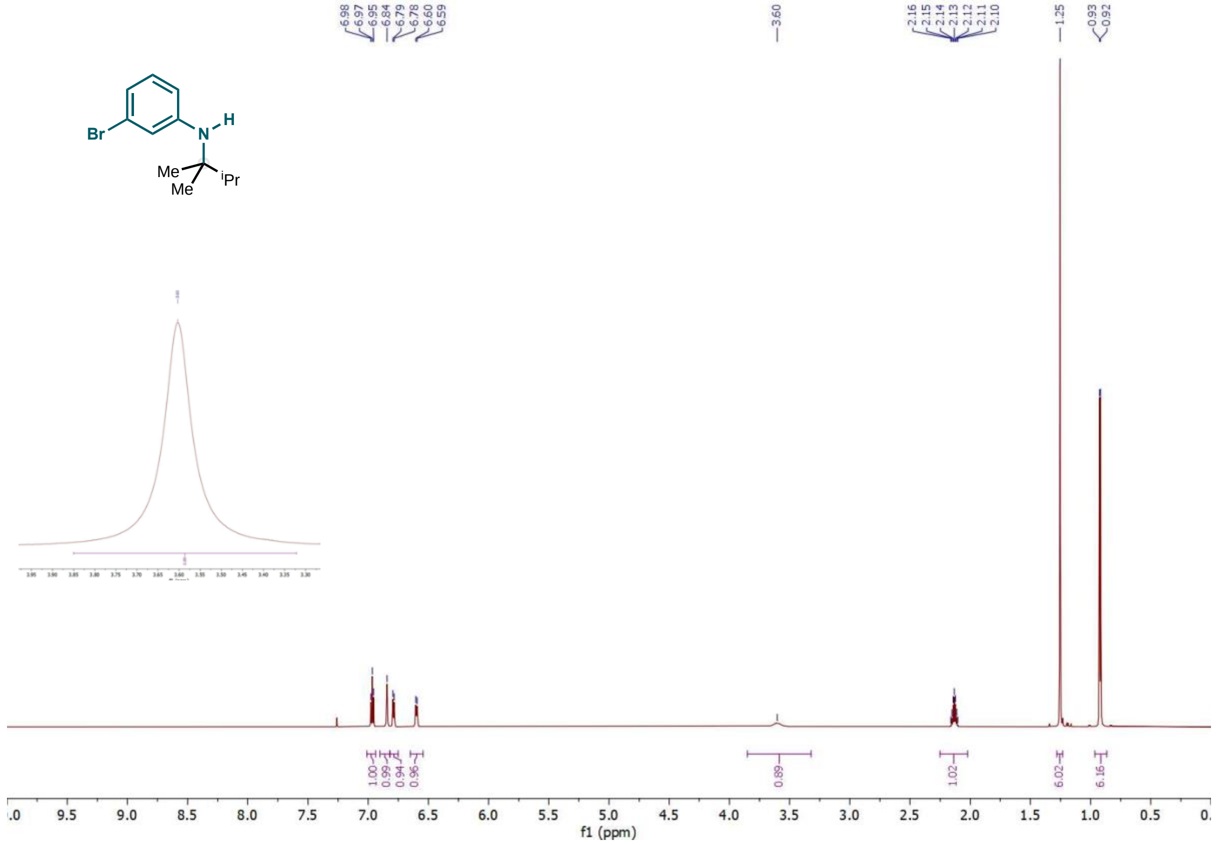

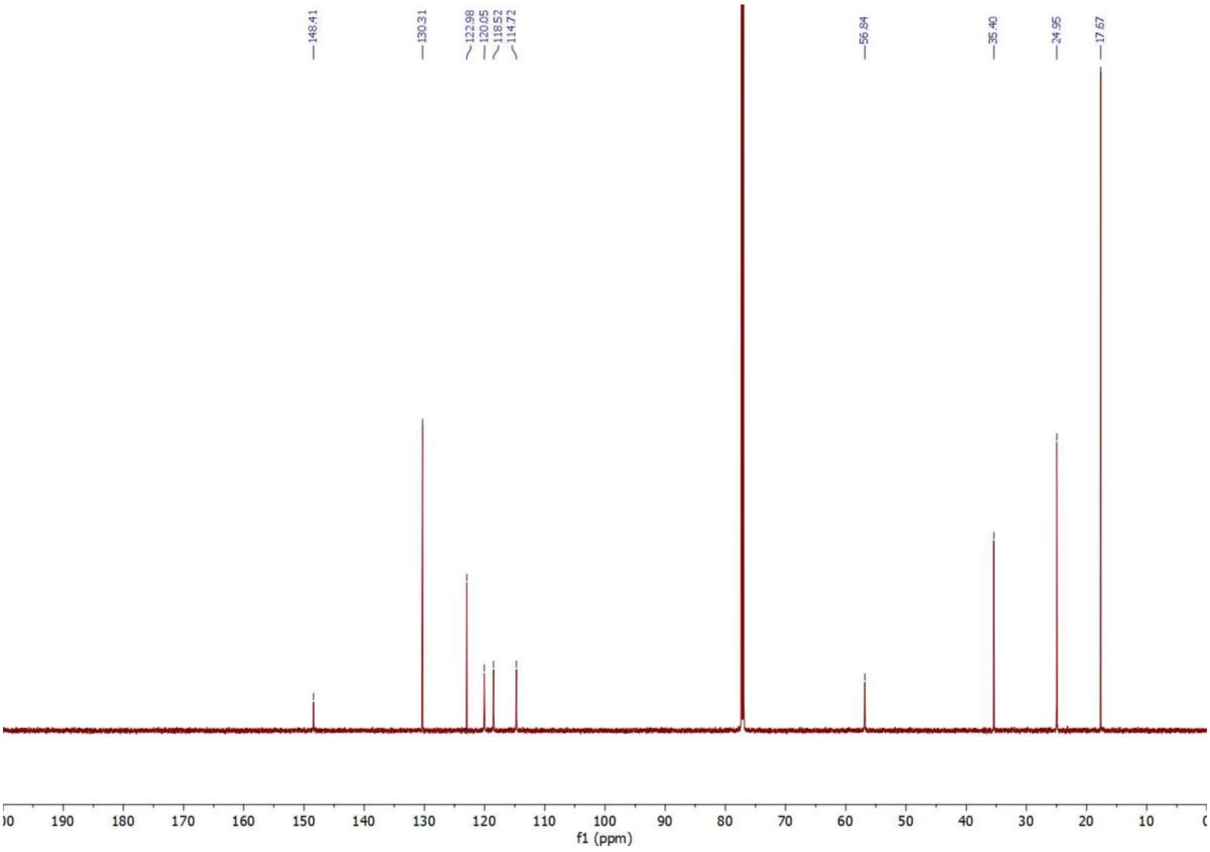

N-(2,3-dimethylbutan-2-yl)-2-methylaniline (17g)

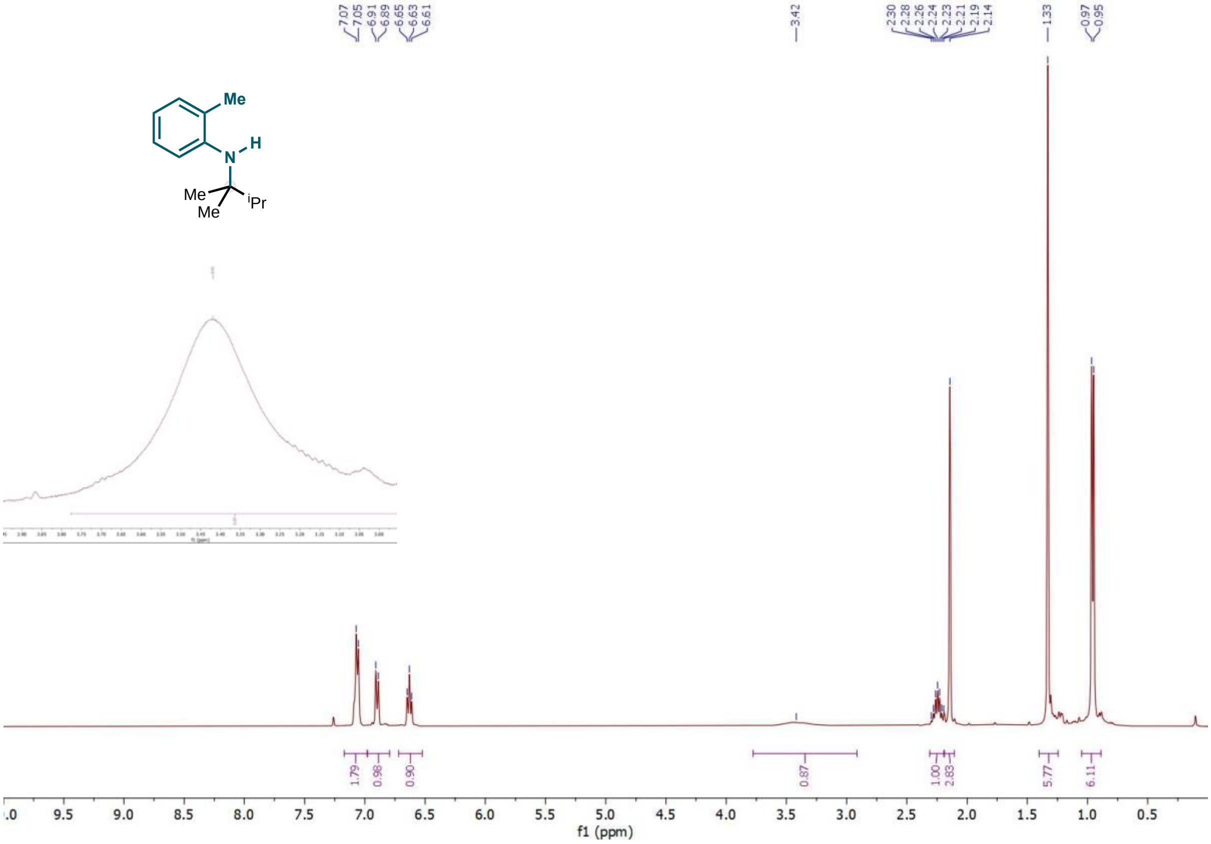

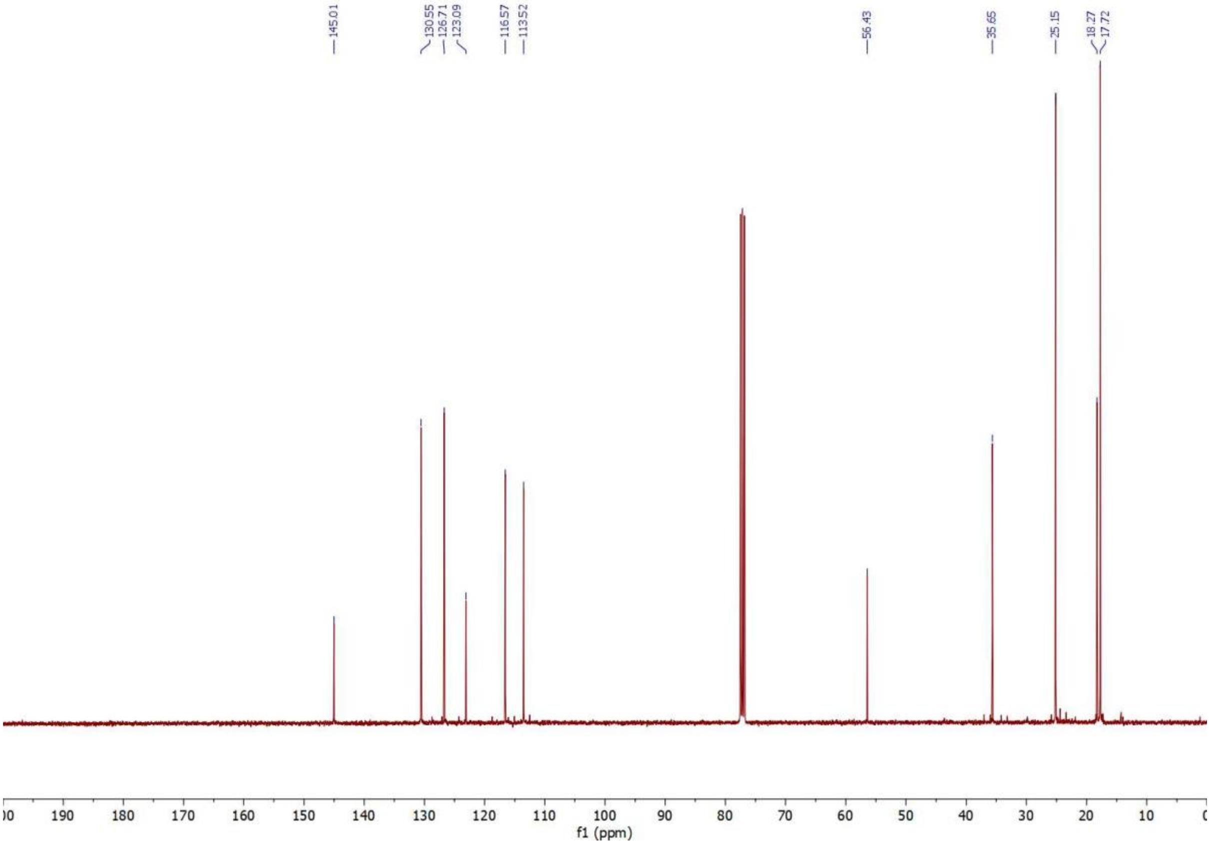

methyl 4-((2,3-dimethylbutan-2-yl)amino)-2-methoxybenzoate (17h)

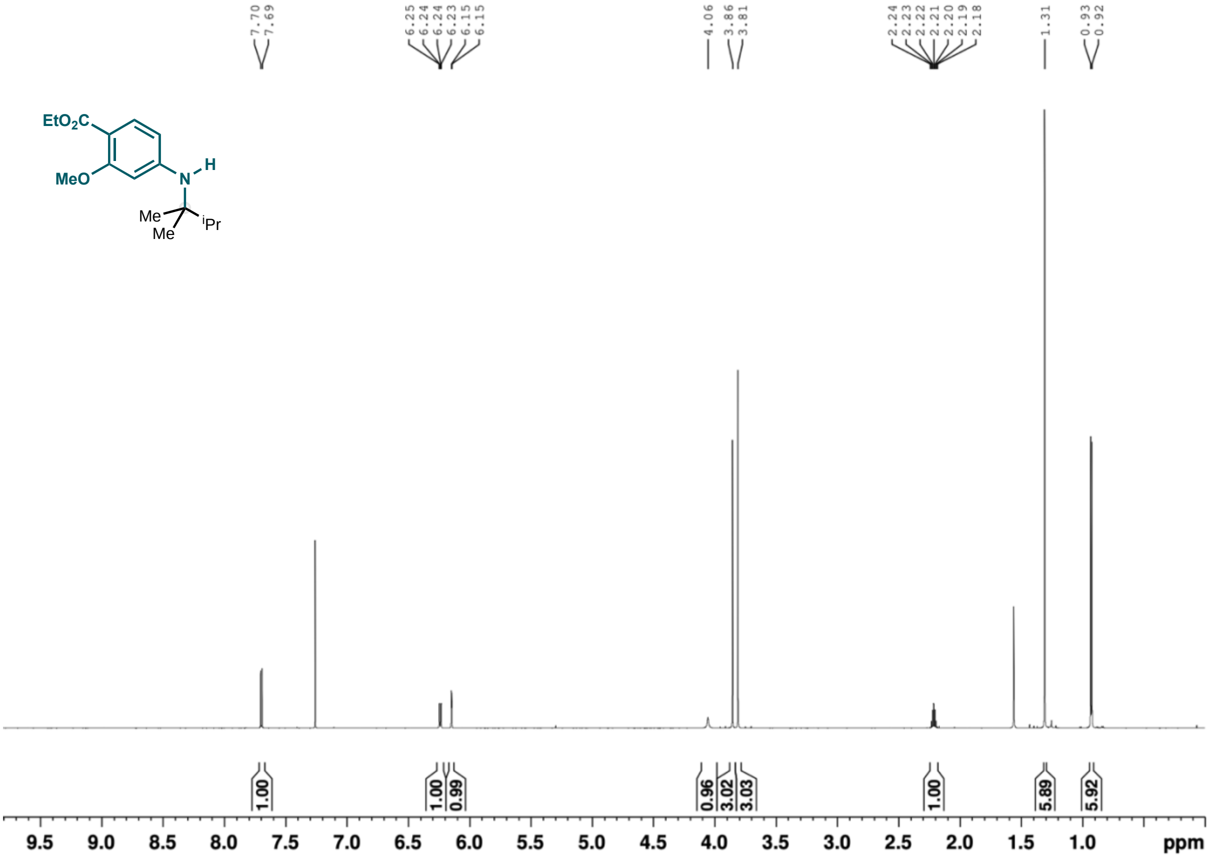

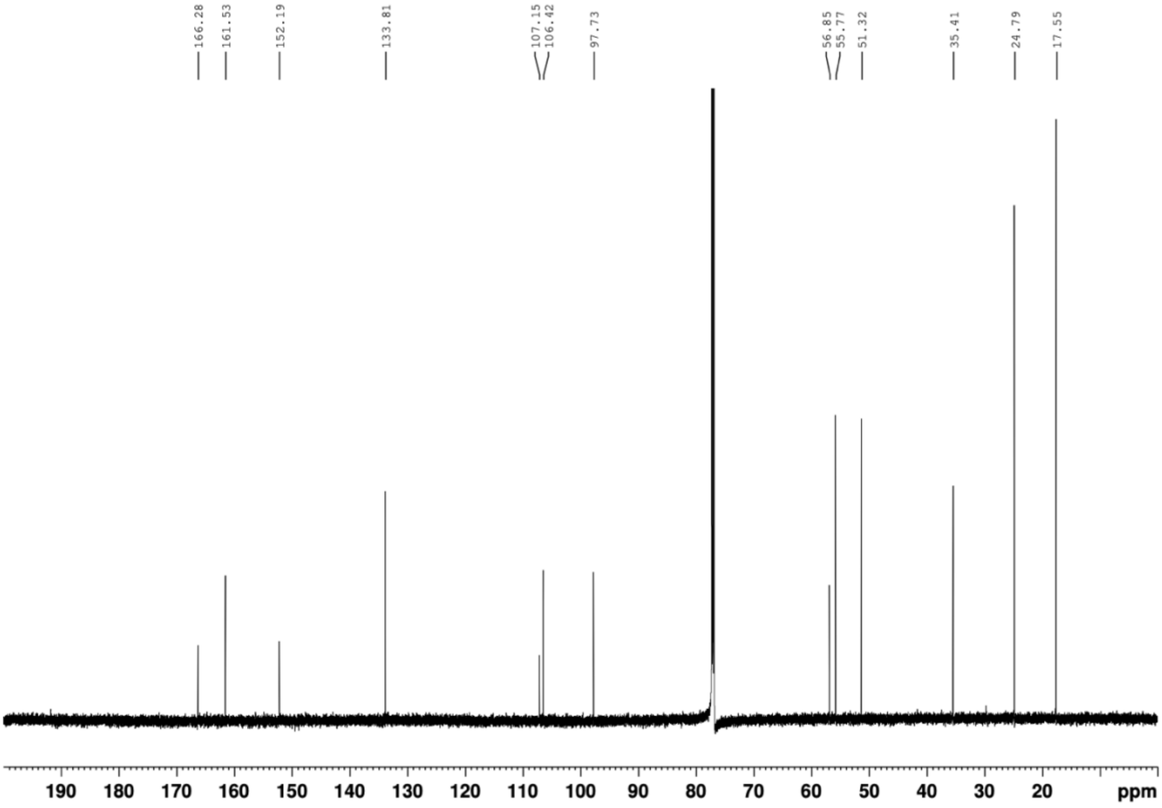

Benzyl 4-isopropyl-4-(phenethylamino)piperidine-1-carboxylate (18a)

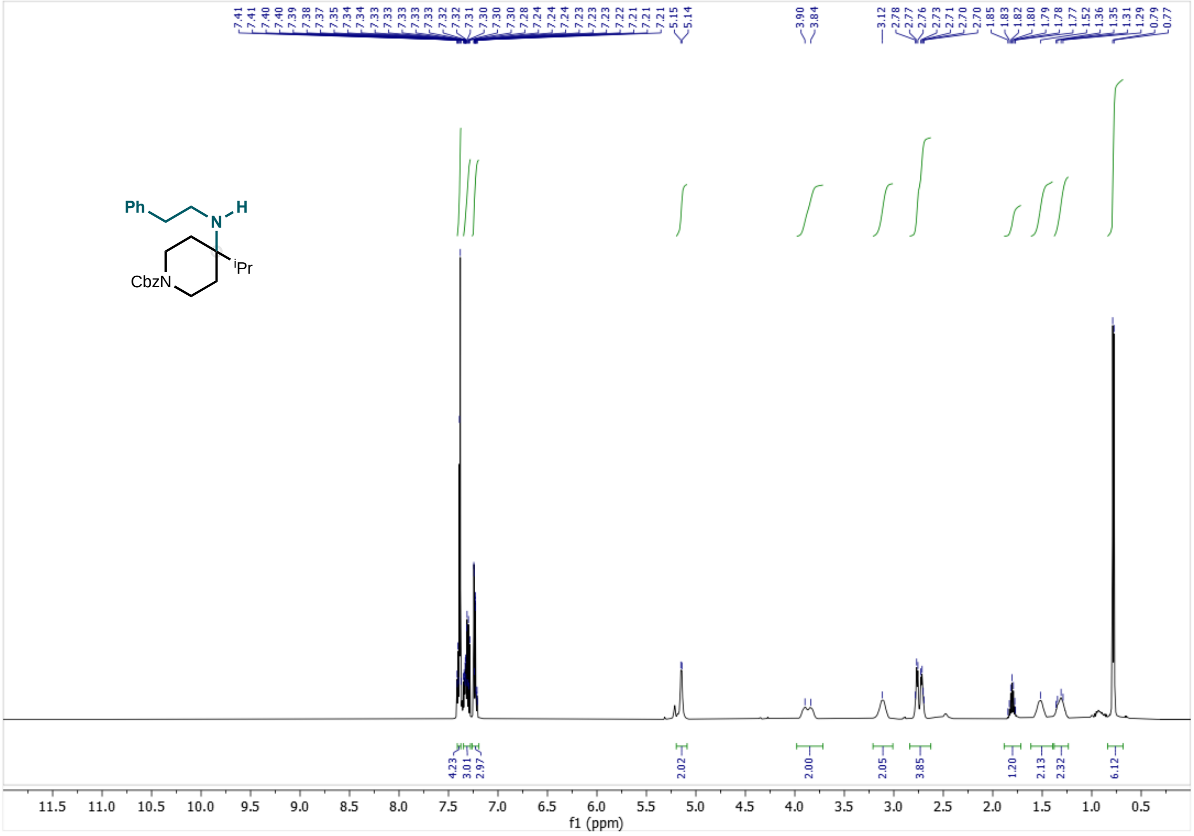

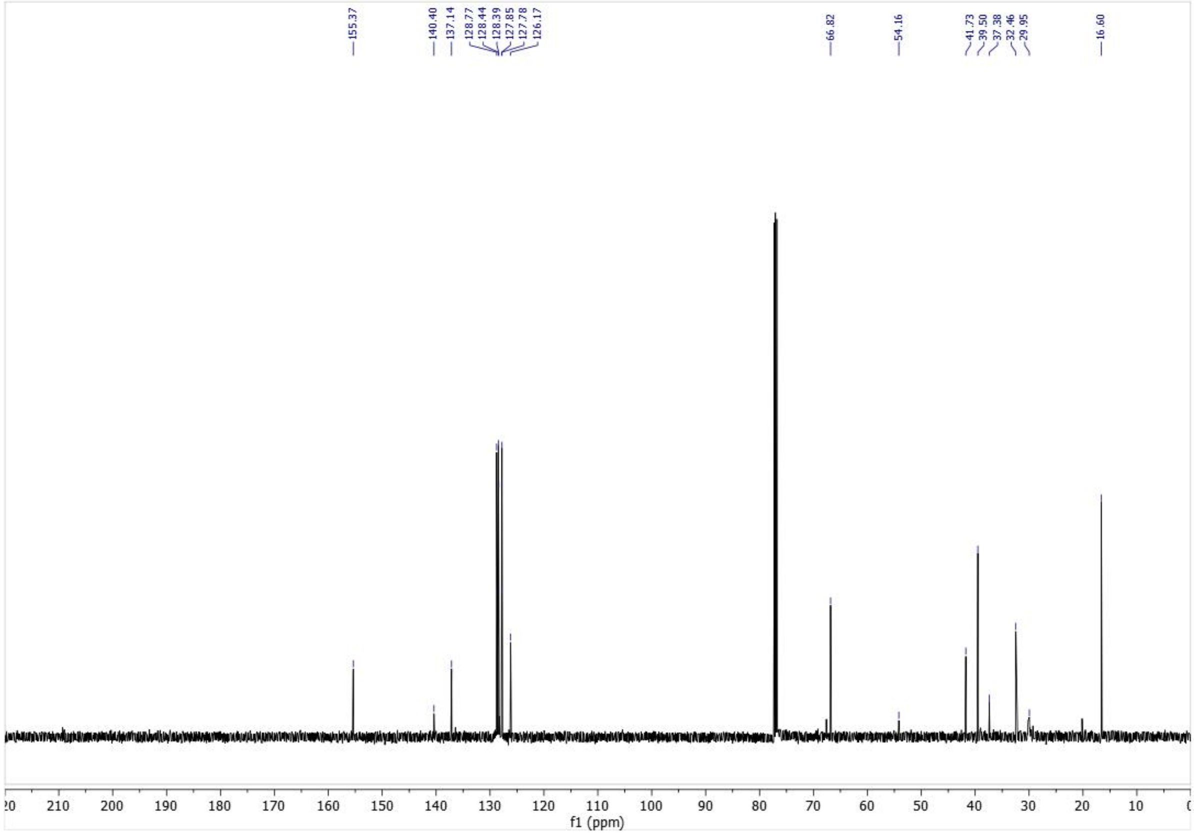

Benzyl 4-((2-fluoroethyl)amino)-4-isopropylpiperidine-1-carboxylate (18b)

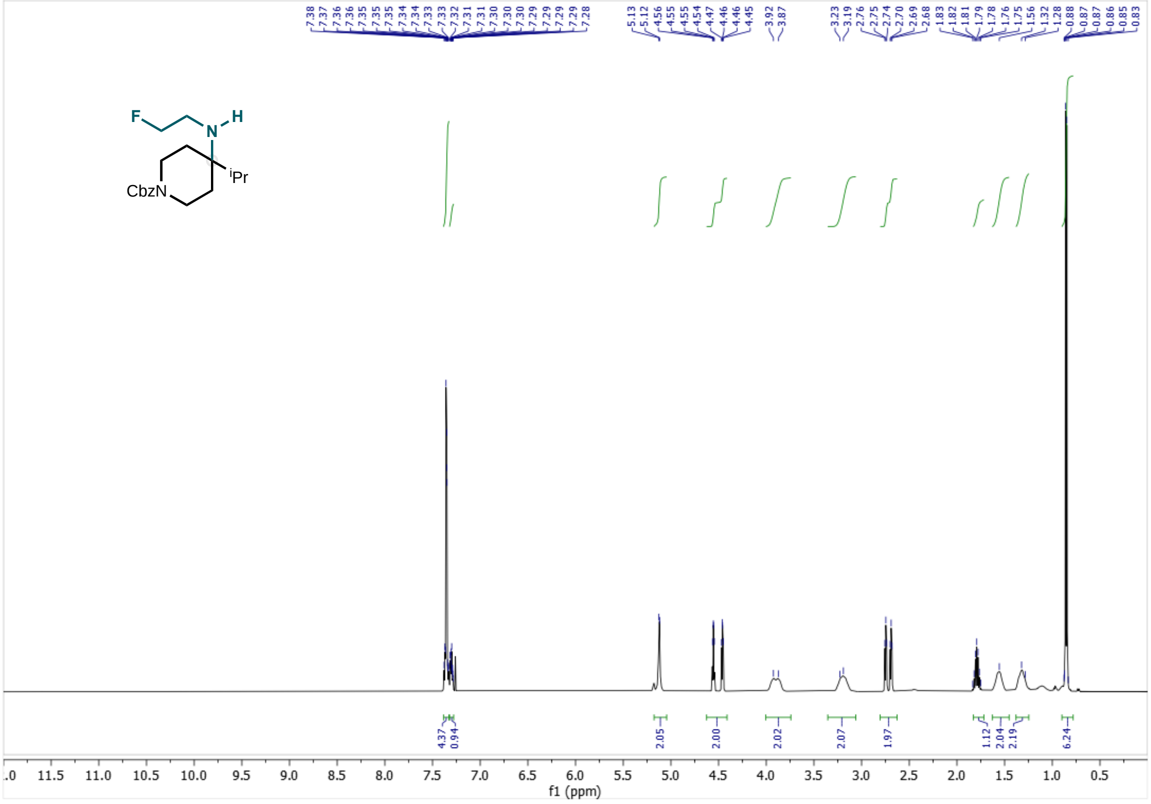

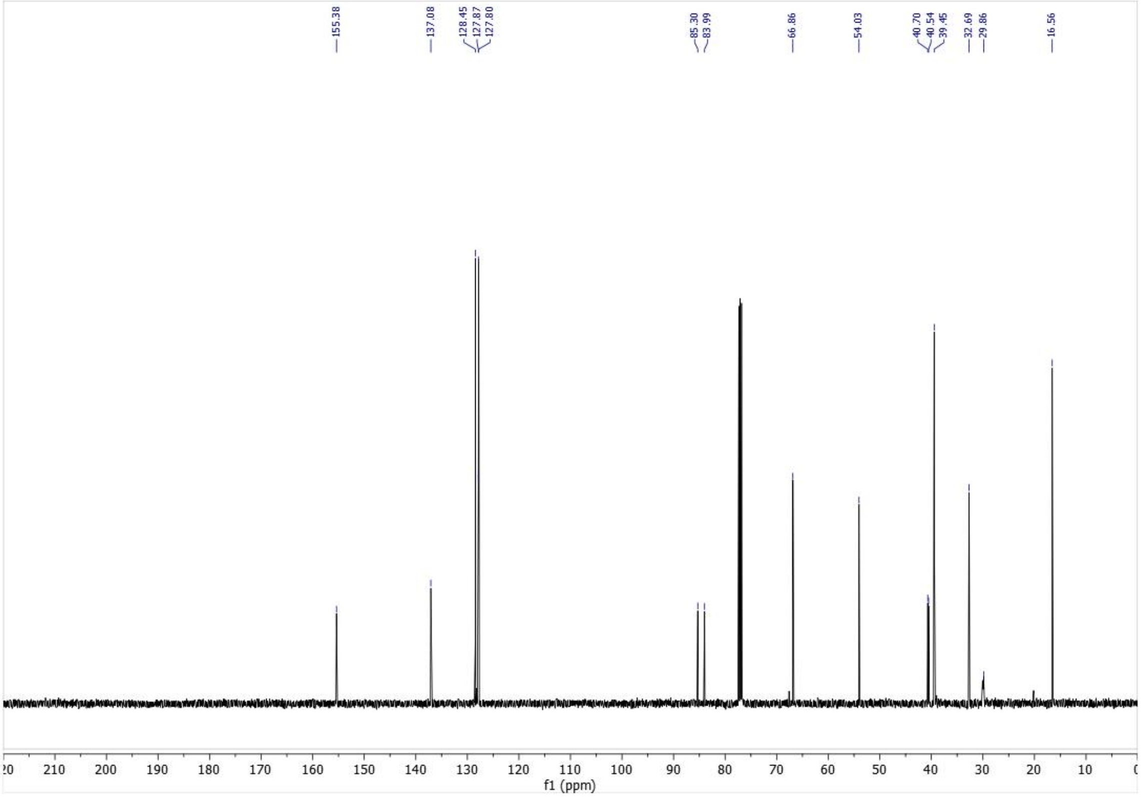

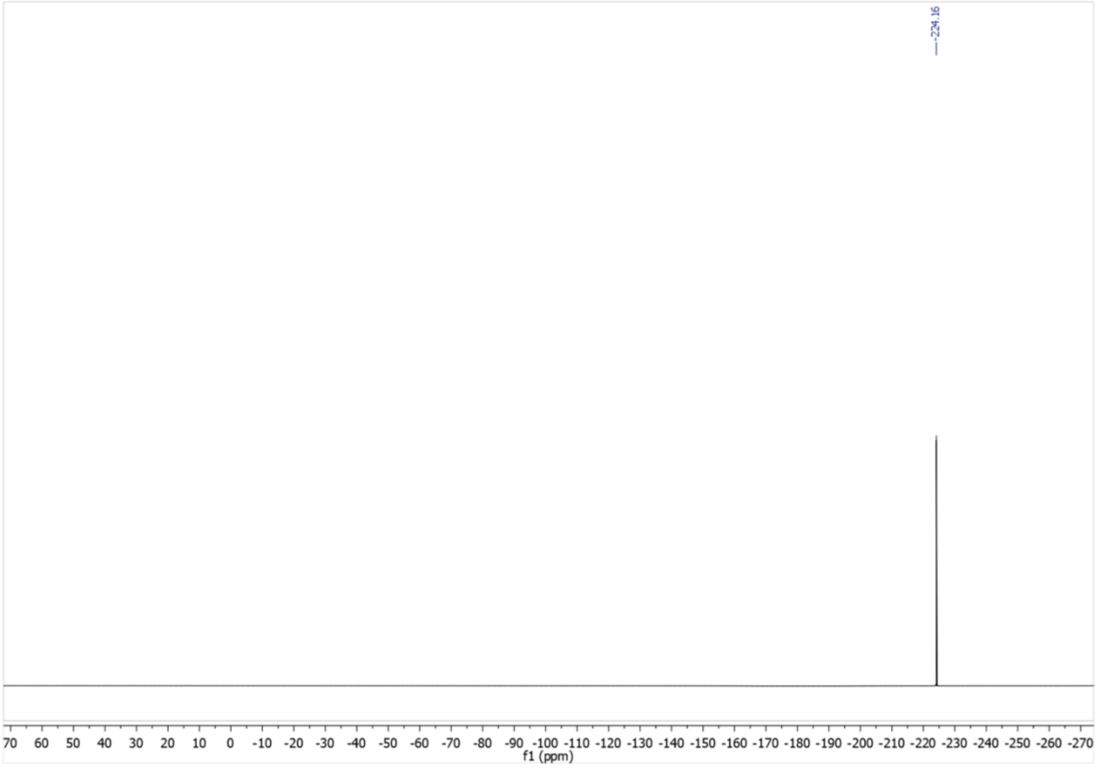
-224.16
70 60 50 40 30 20 10 0 -10 -20 -30 -40 -50 -60 -70 -80 -90 -100 -110 -120 -130 -140 -150 -160 -170 -180 -190 -200 -210 -220 -230 -240 -250 -260 -270
f1 (ppm)

Benzyl 4-((2-chloroethyl)amino)-4-isopropylpiperidine-1-carboxylate (18c)

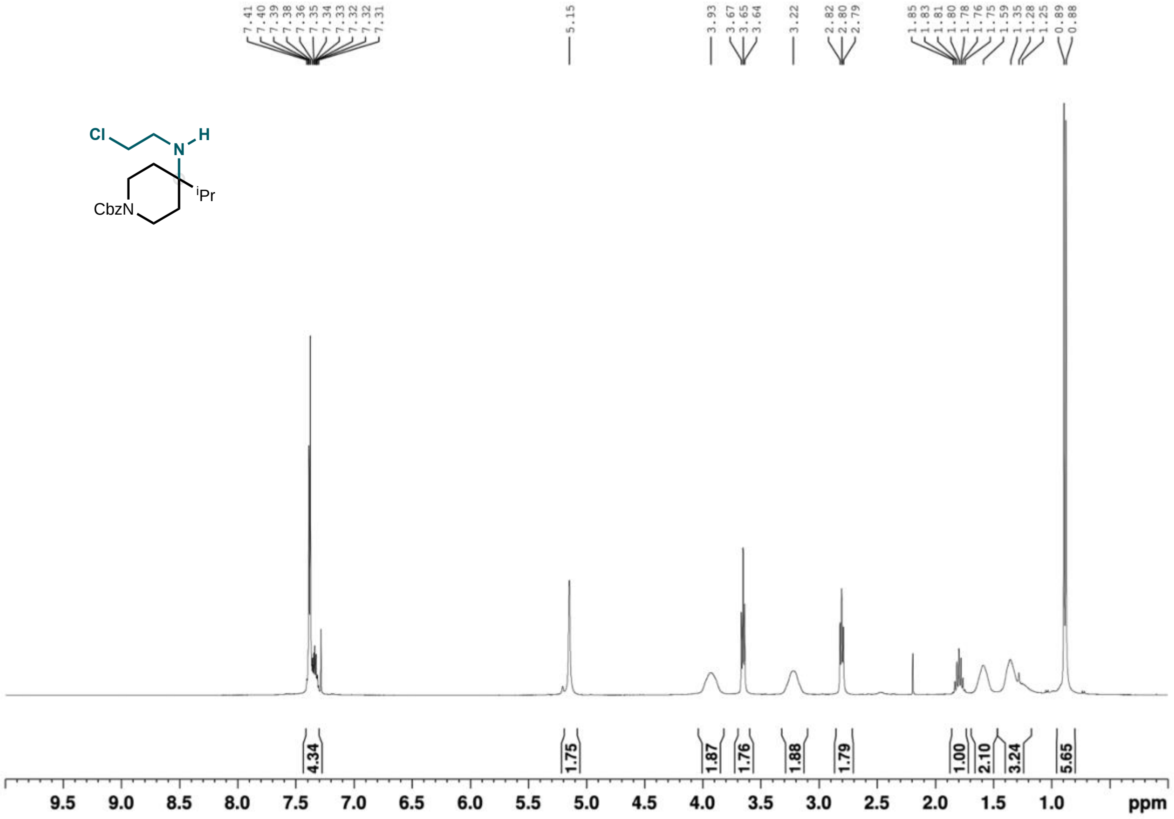

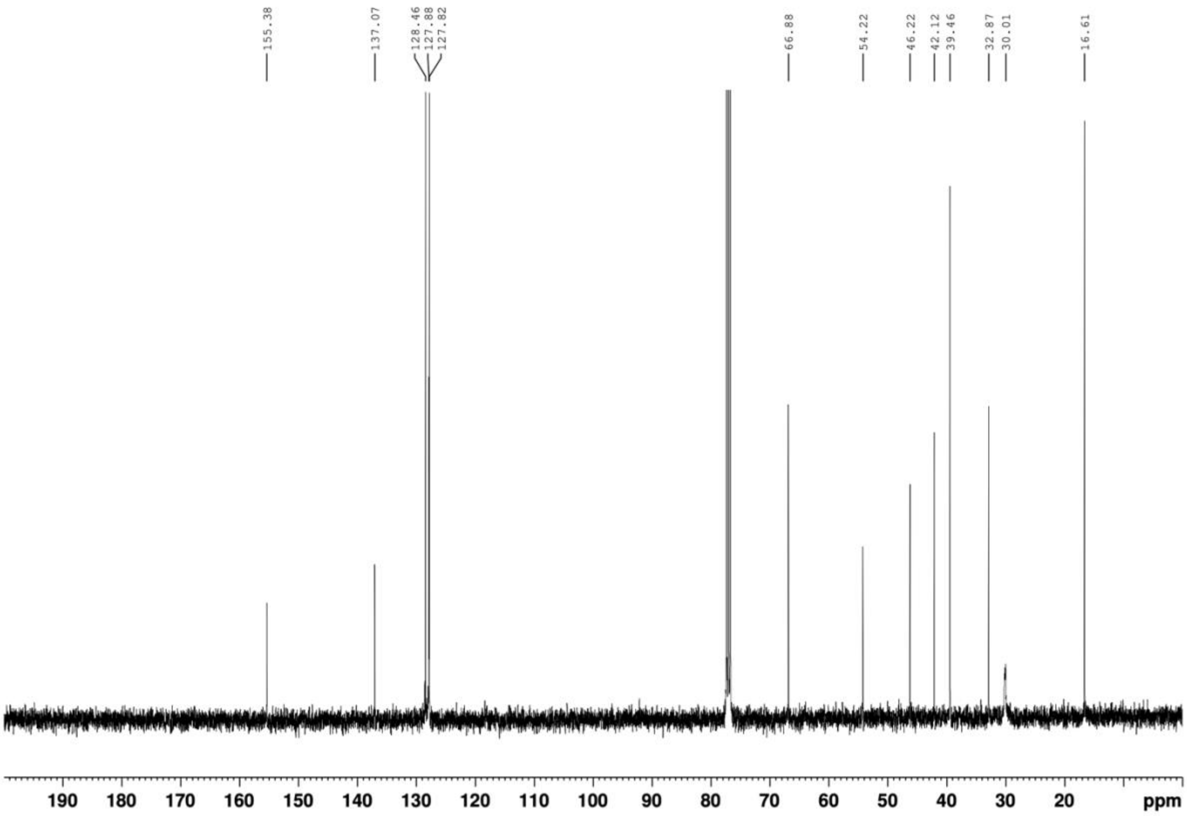

Benzyl 4-isopropyl-4-(methylamino)piperidine-1-carboxylate (18d)

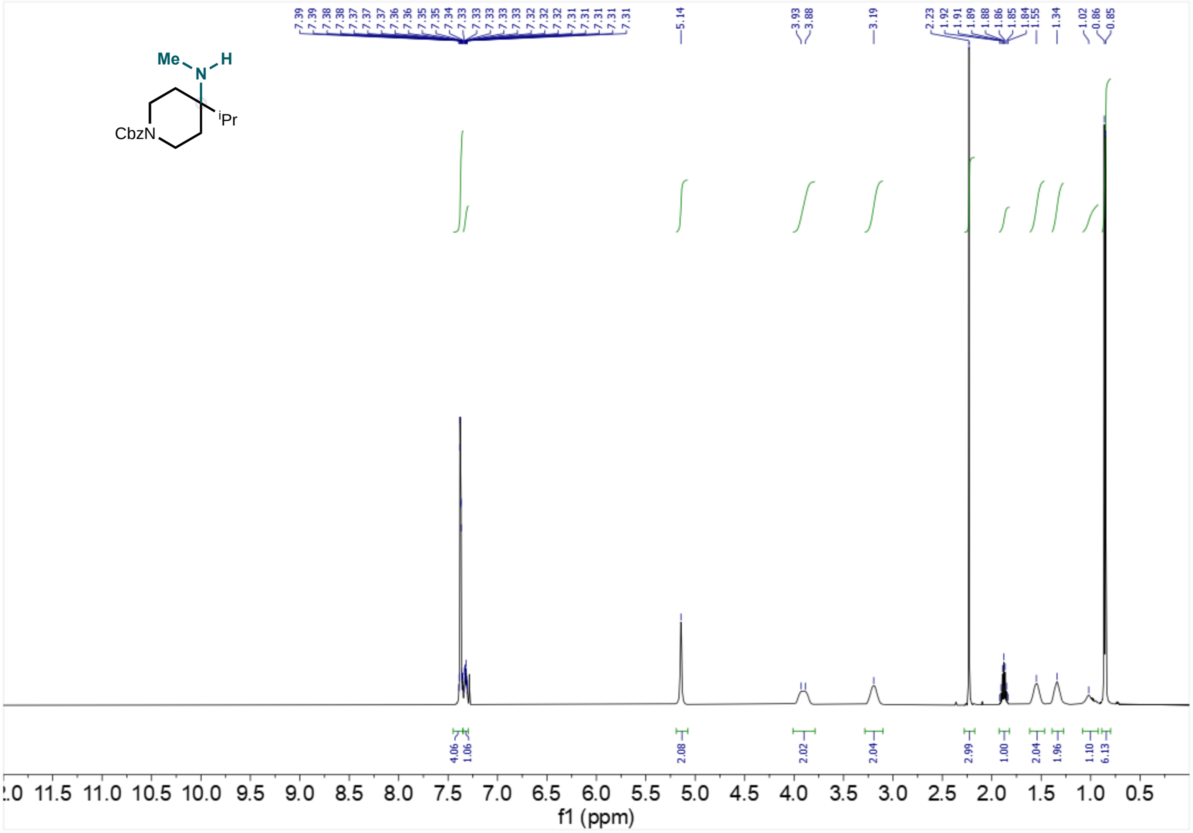

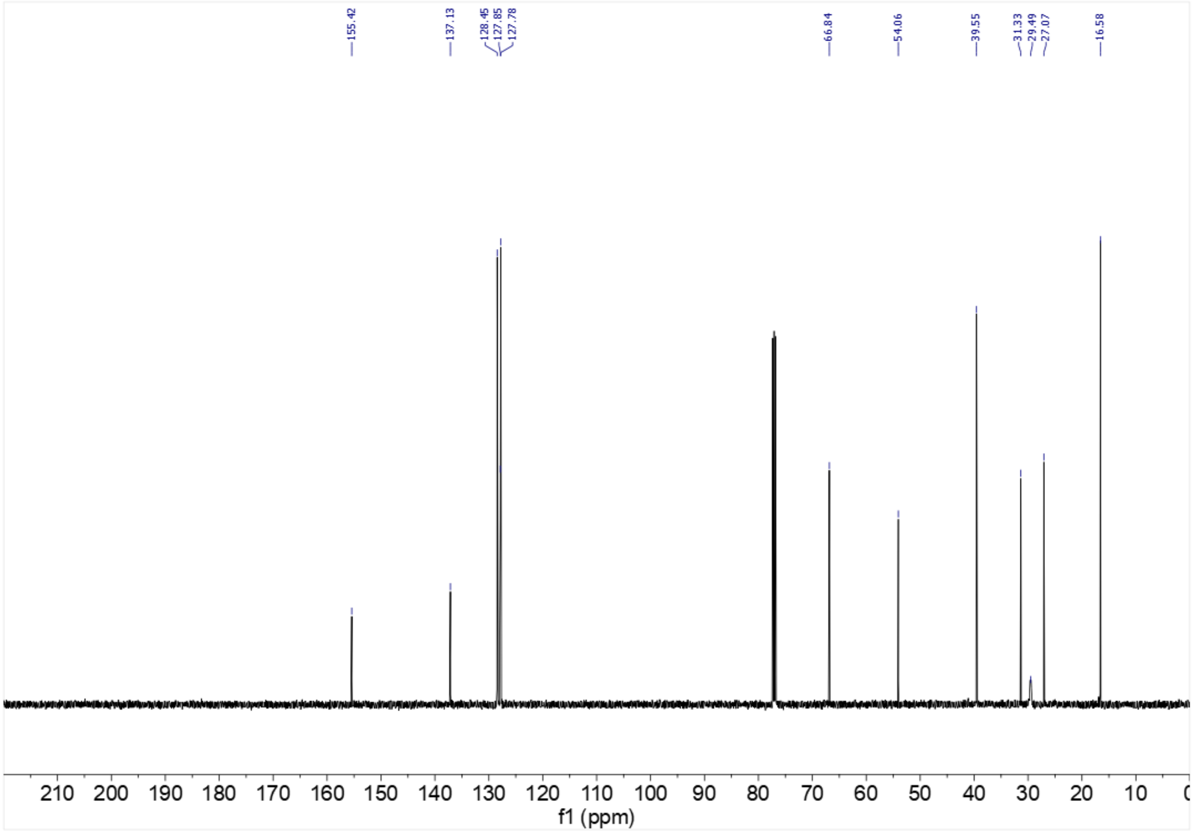

Benzyl 4-(benzylamino)-4-isopropylpiperidine-1-carboxylate (18e)

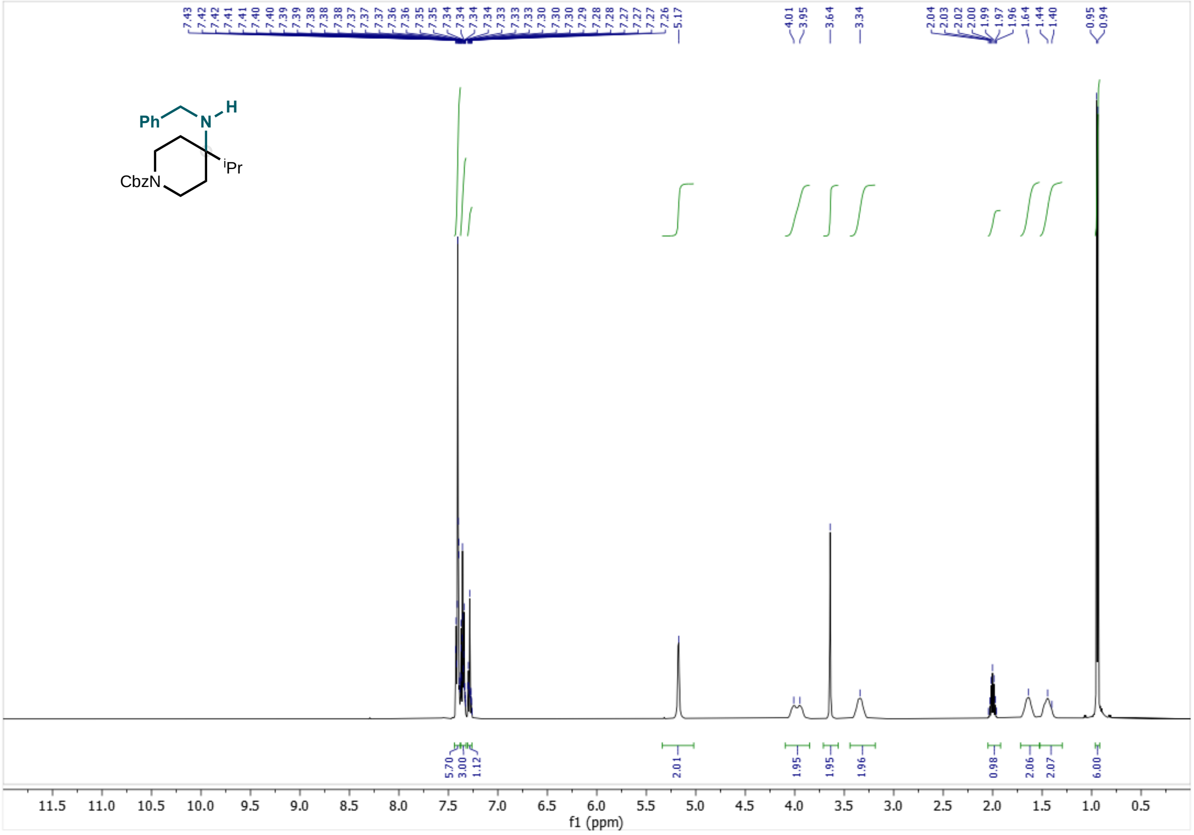

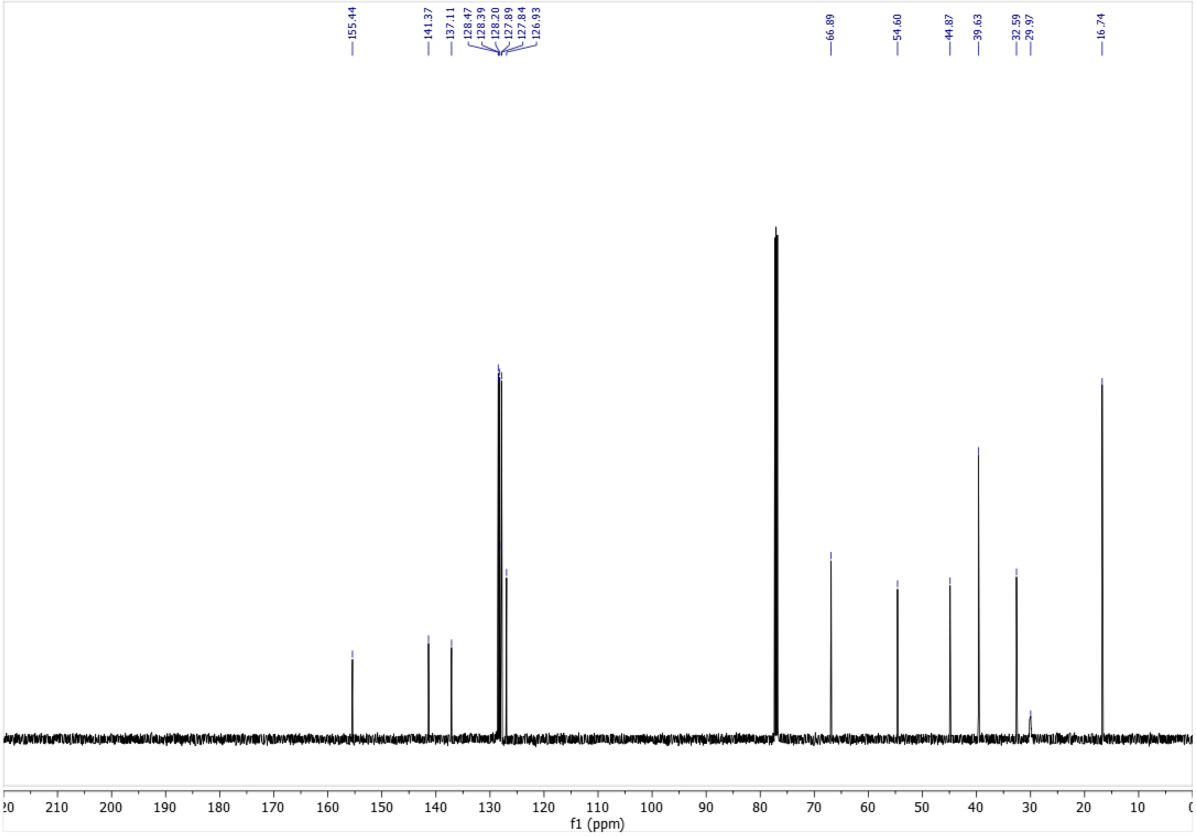

Benzyl 4-(but-3-en-1-ylamino)-4-isopropylpiperidine-1-carboxylate (18f)

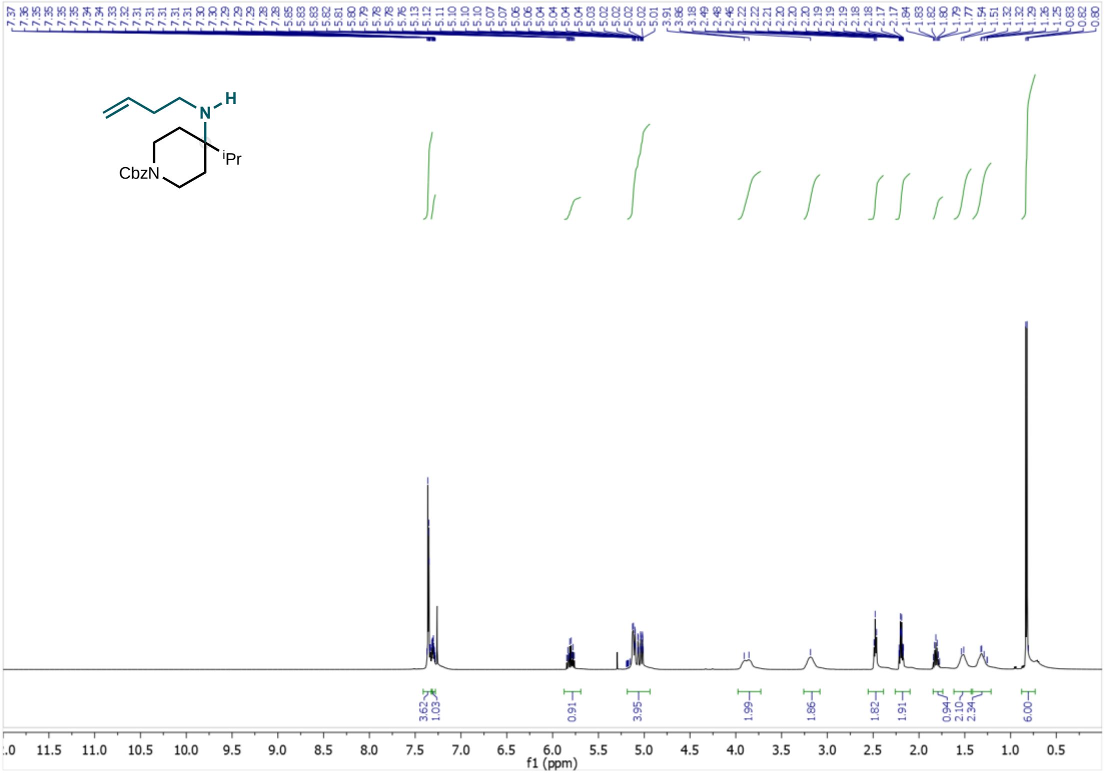

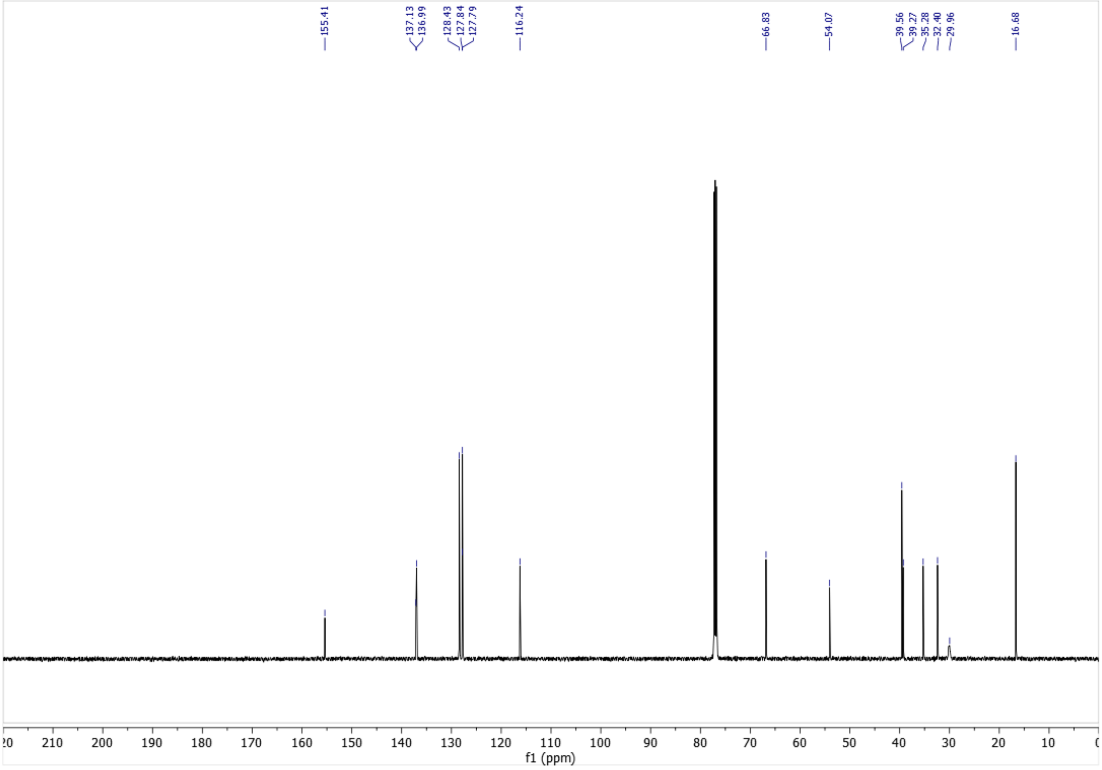

Benzyl 4-isopropyl-4-((2-(thiophen-2-yl)ethyl)amino)piperidine-1-carboxylate (18g)

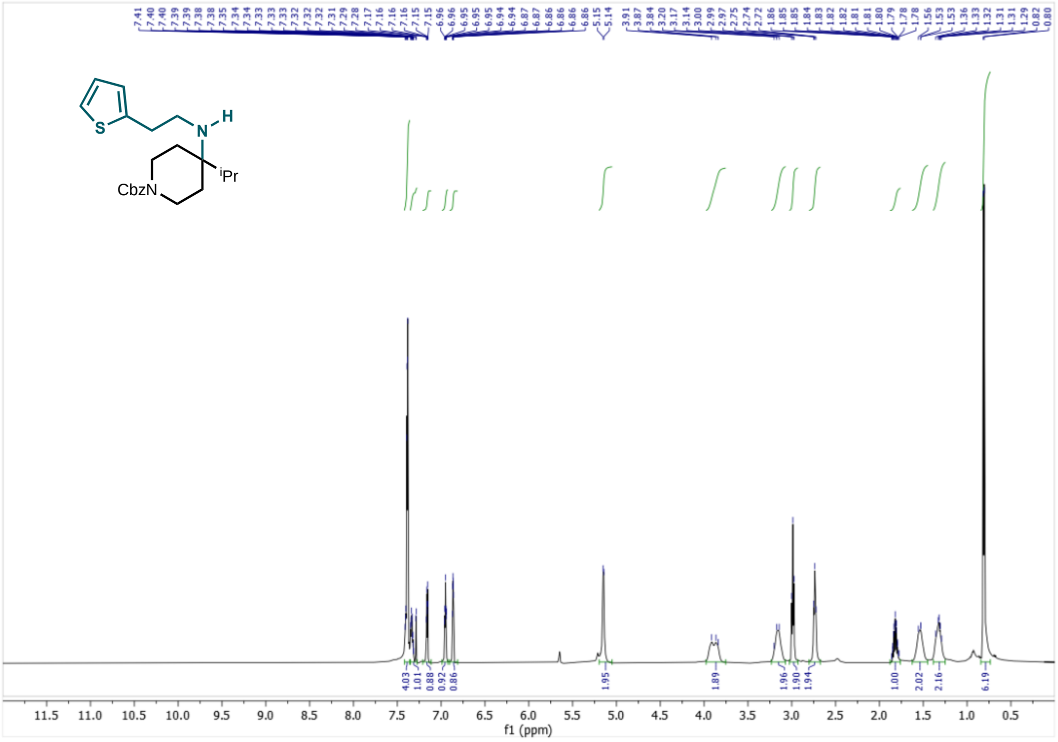

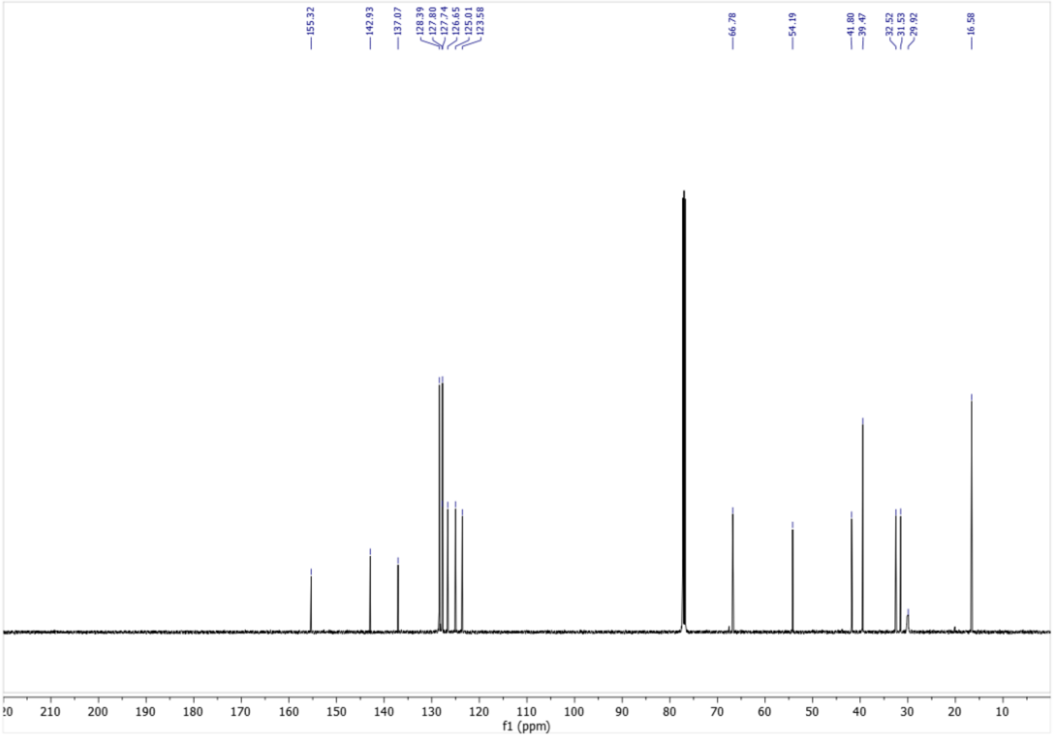

N-(1-isopropylcyclobutyl)-4-methoxyaniline (19a)

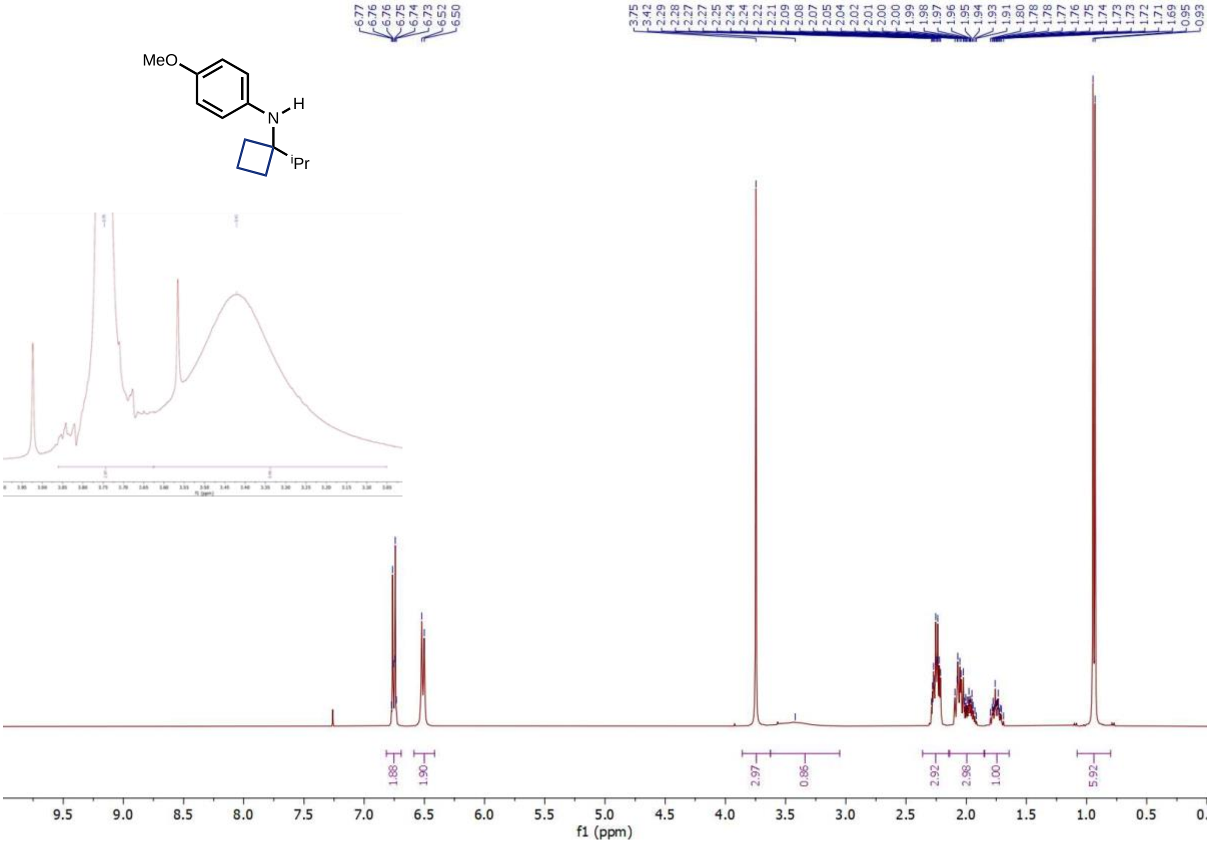

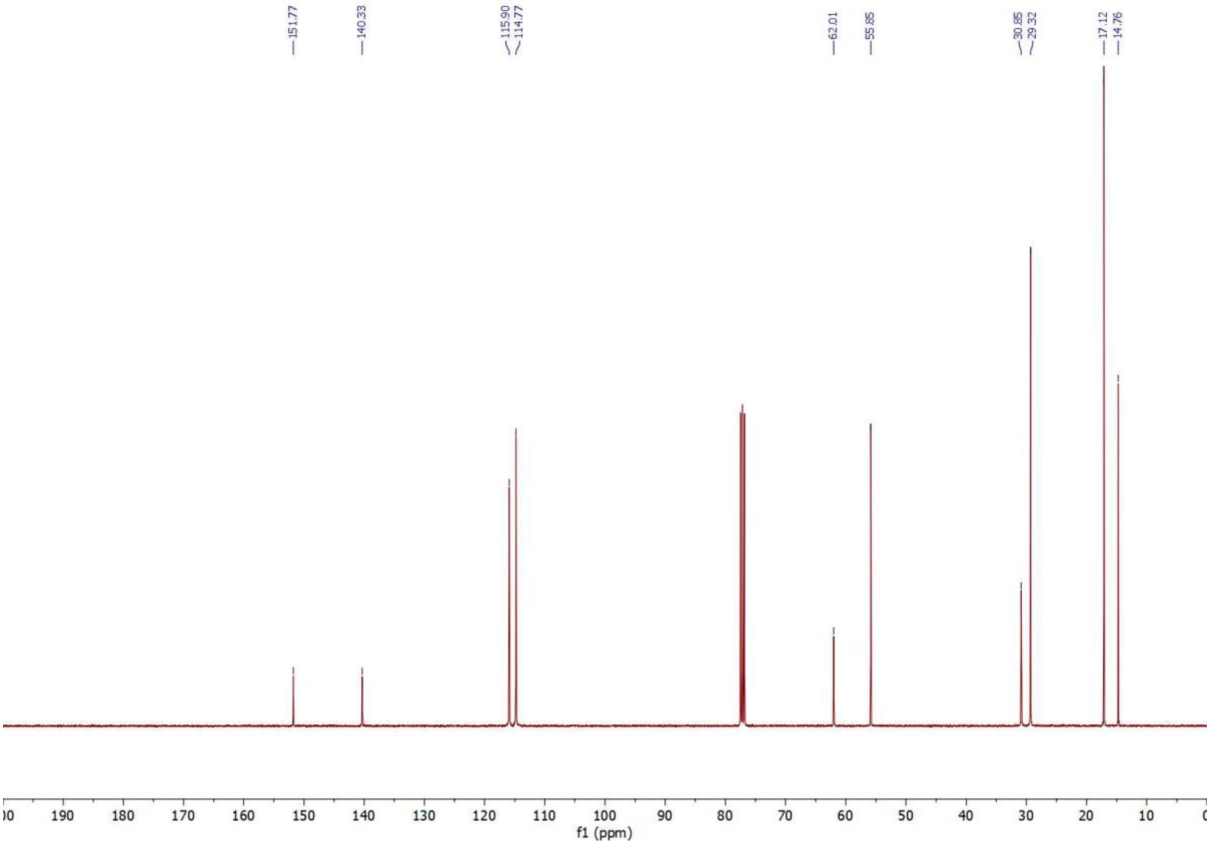

N-(1-isopropylcyclopentyl)-4-methoxyaniline (19b)

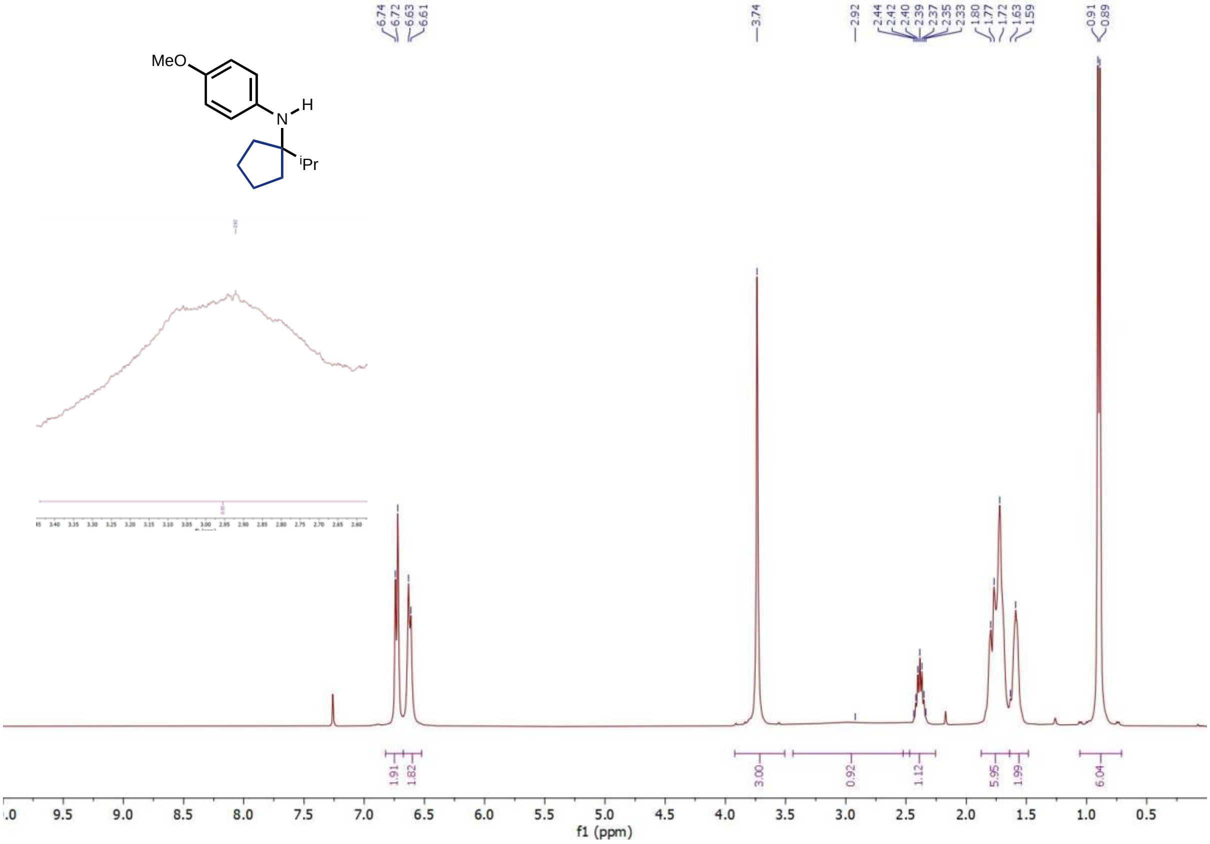

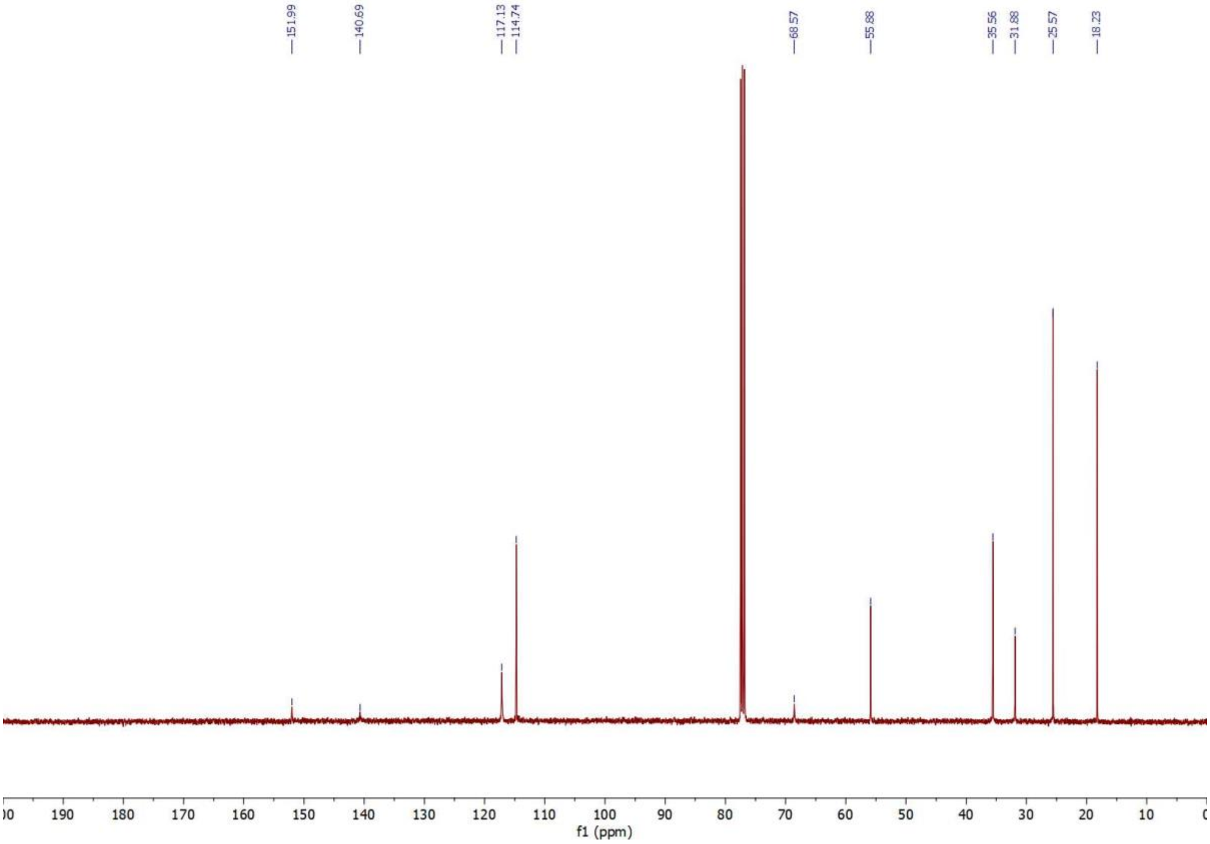

N-(1-isopropylcyclohexyl)-4-methoxyaniline (19c)

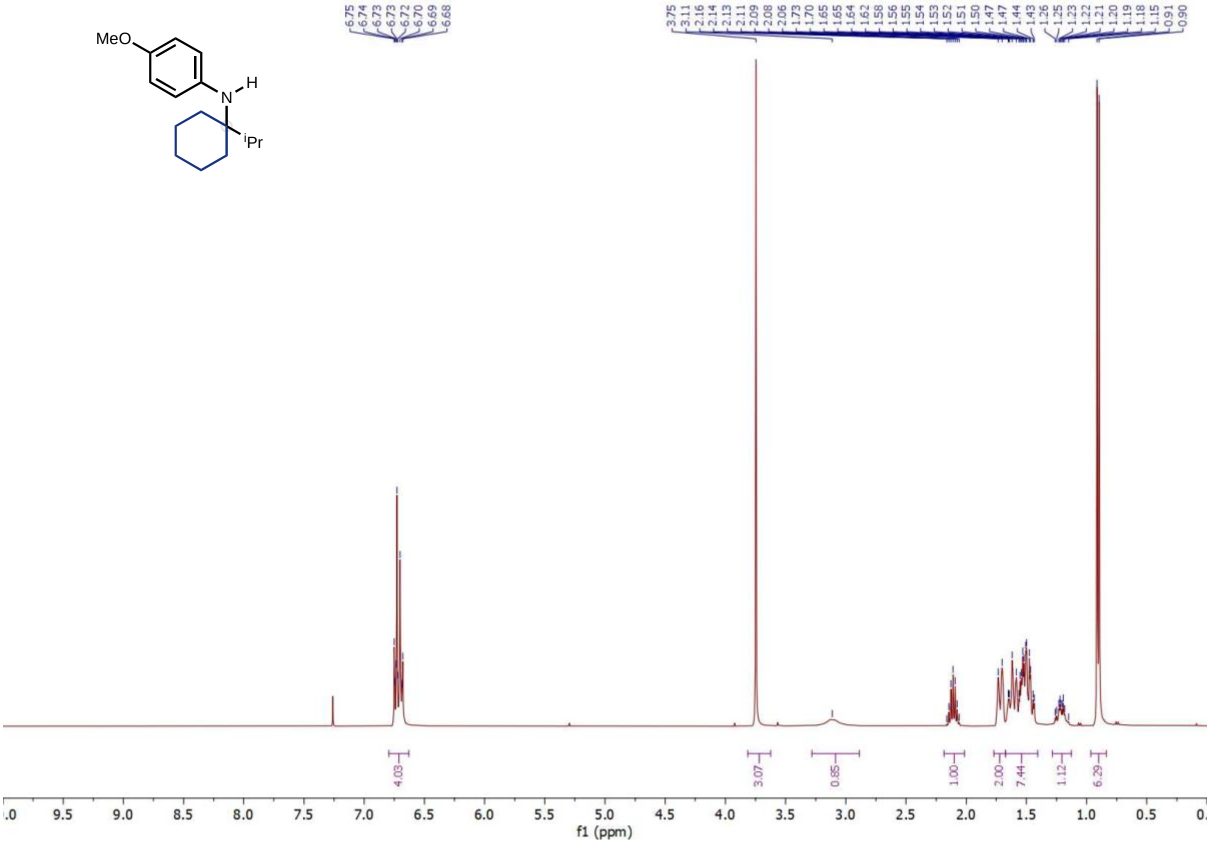

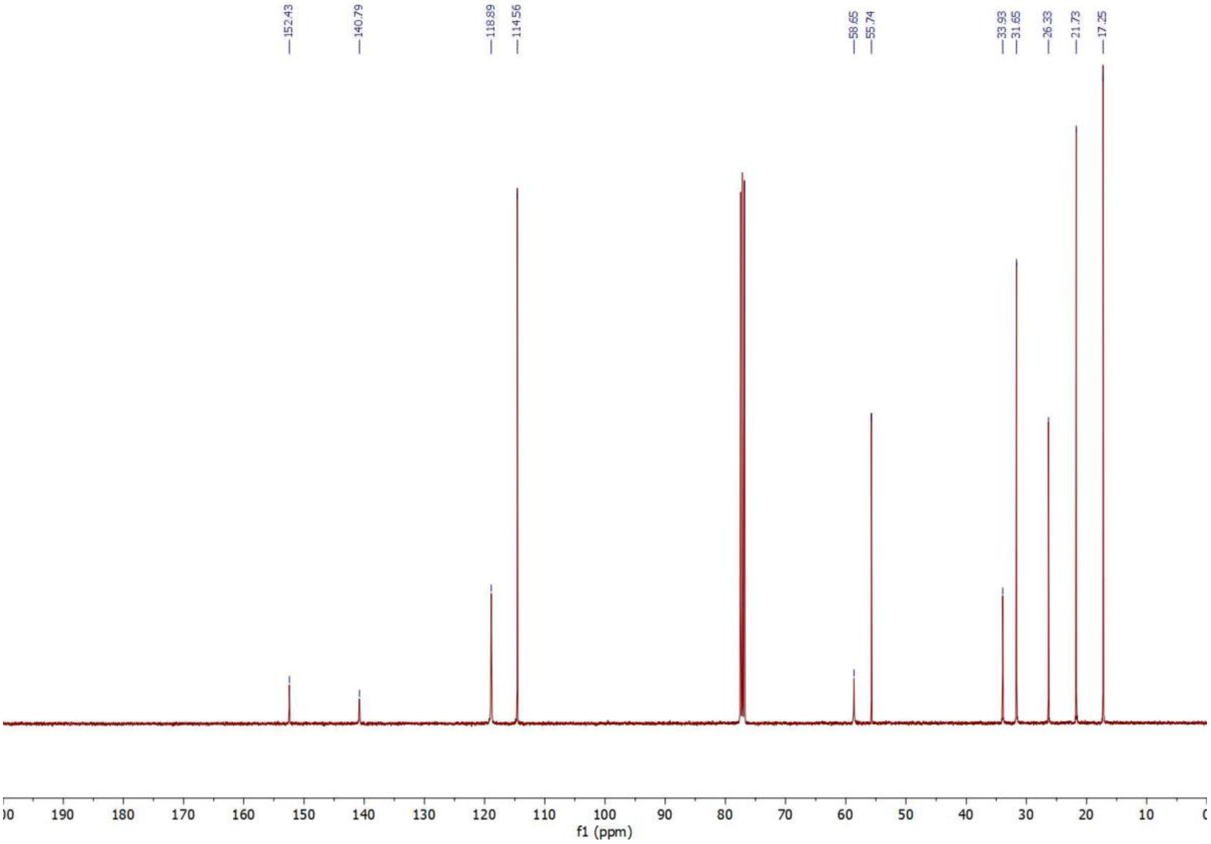

N-(1-isopropyl-4-phenylcyclohexyl)-4-methoxyaniline (19d)

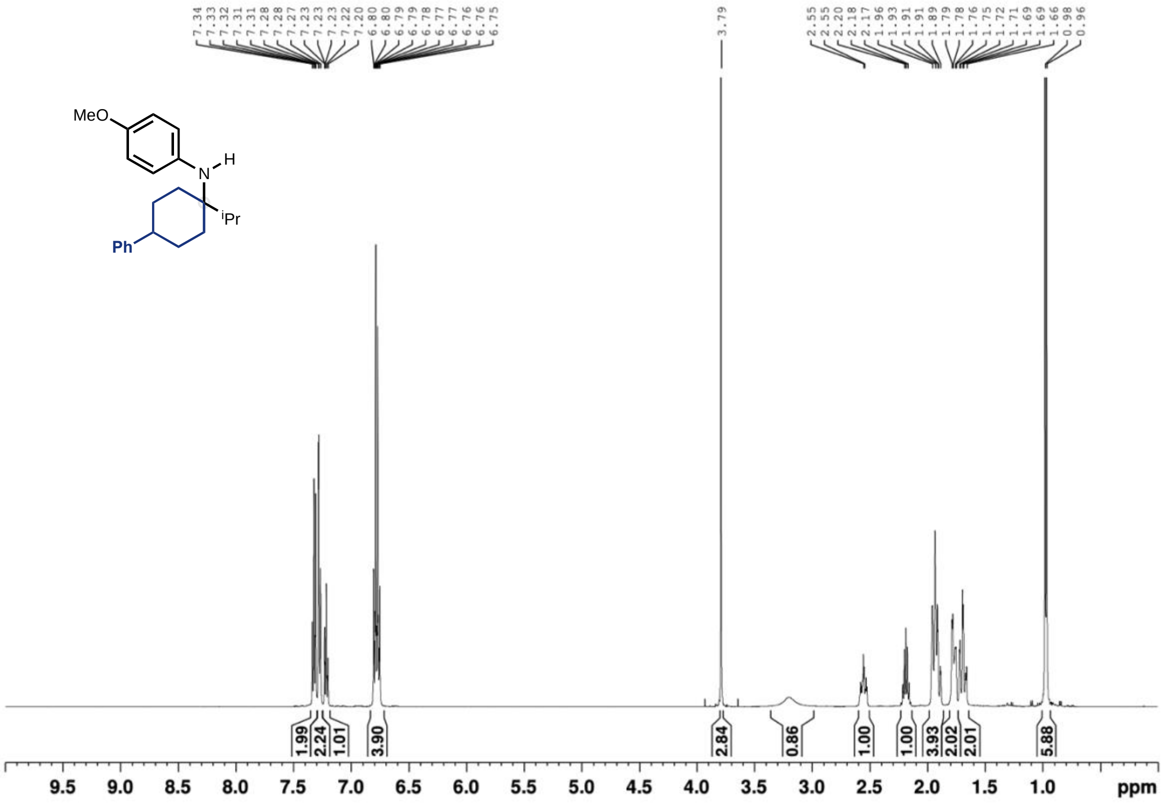

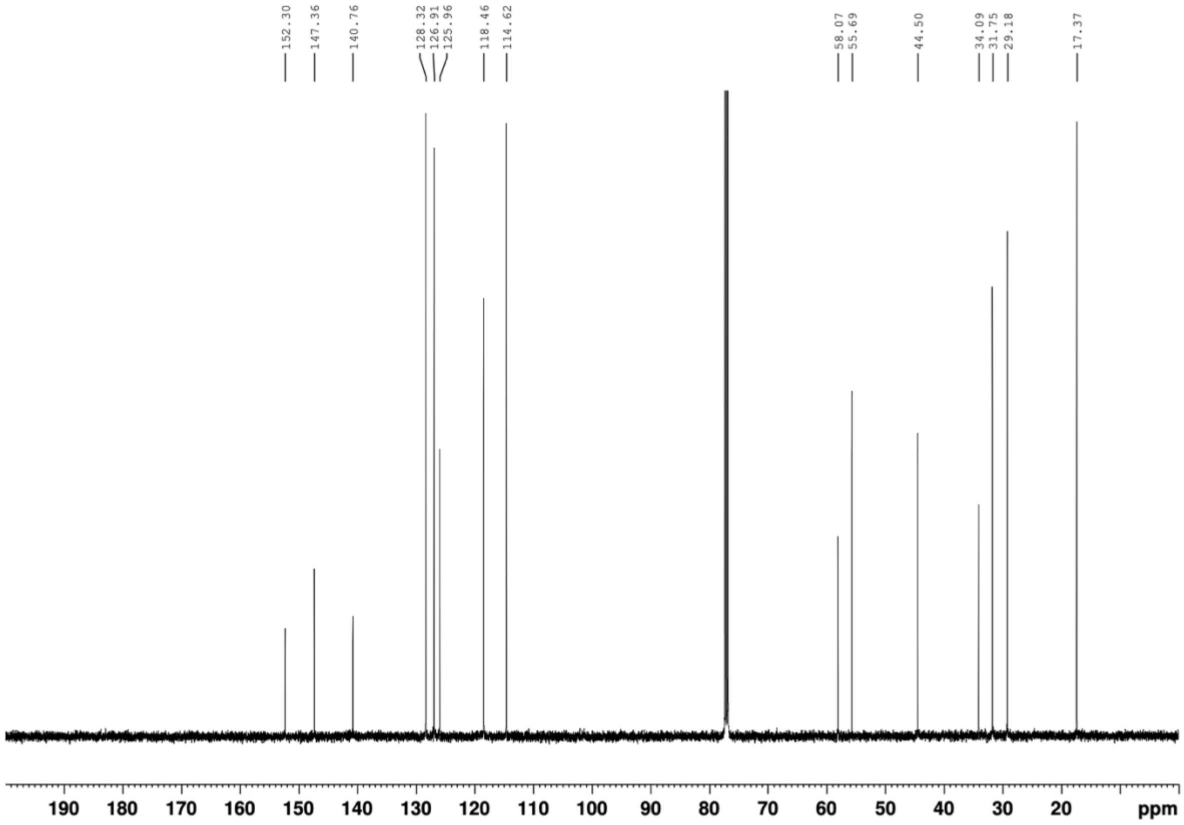

4-isopropyl-N-(4-methoxyphenyl)tetrahydro-2H-pyran-4-amine (19e)

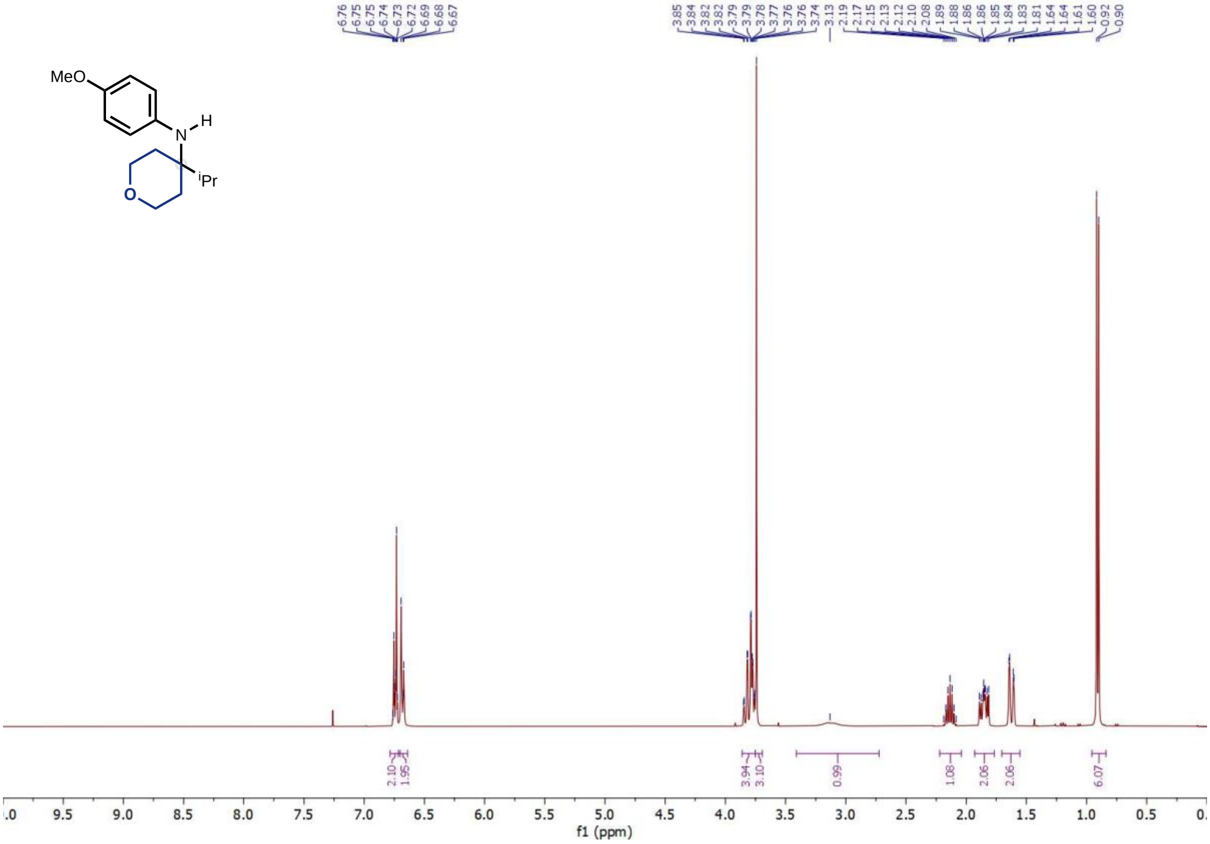

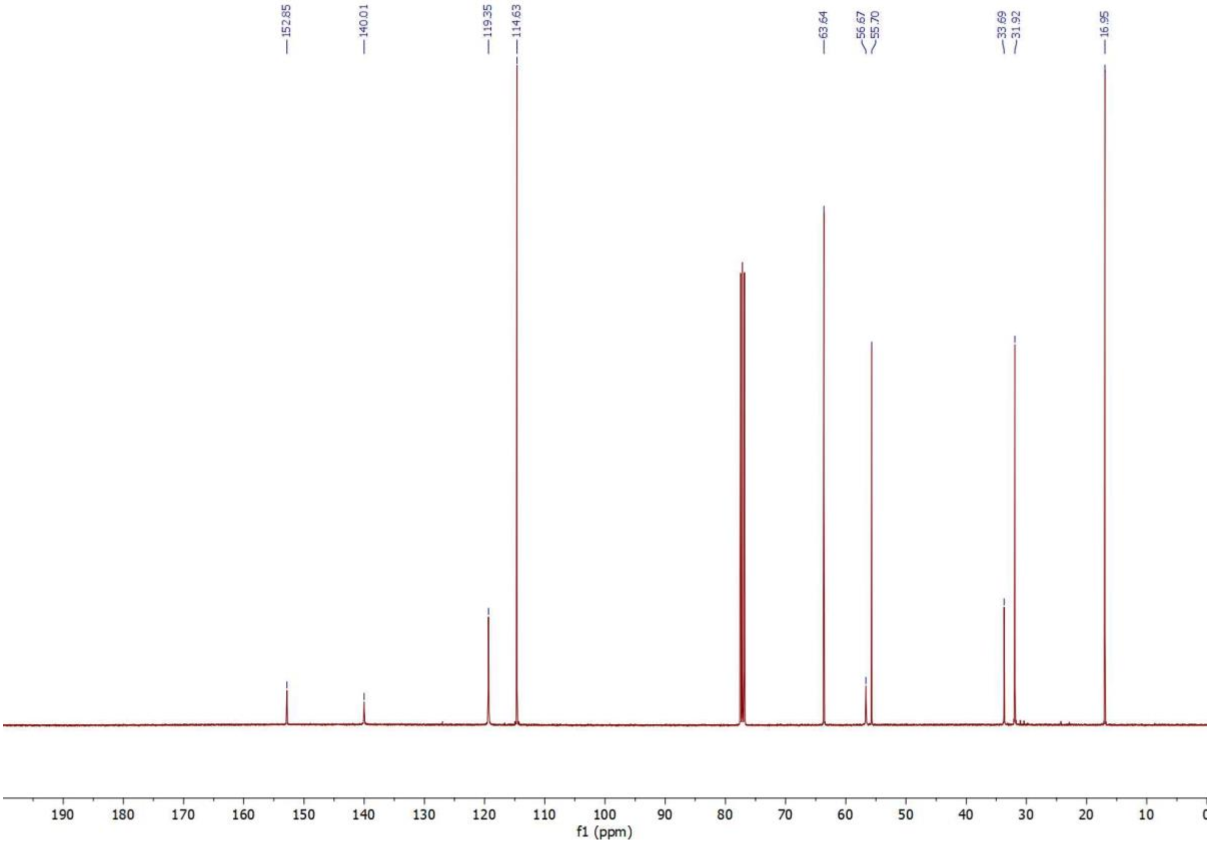

4-isopropyl-N-(4-methoxyphenyl)tetrahydro-2H-thiopyran-4-amine (19f)

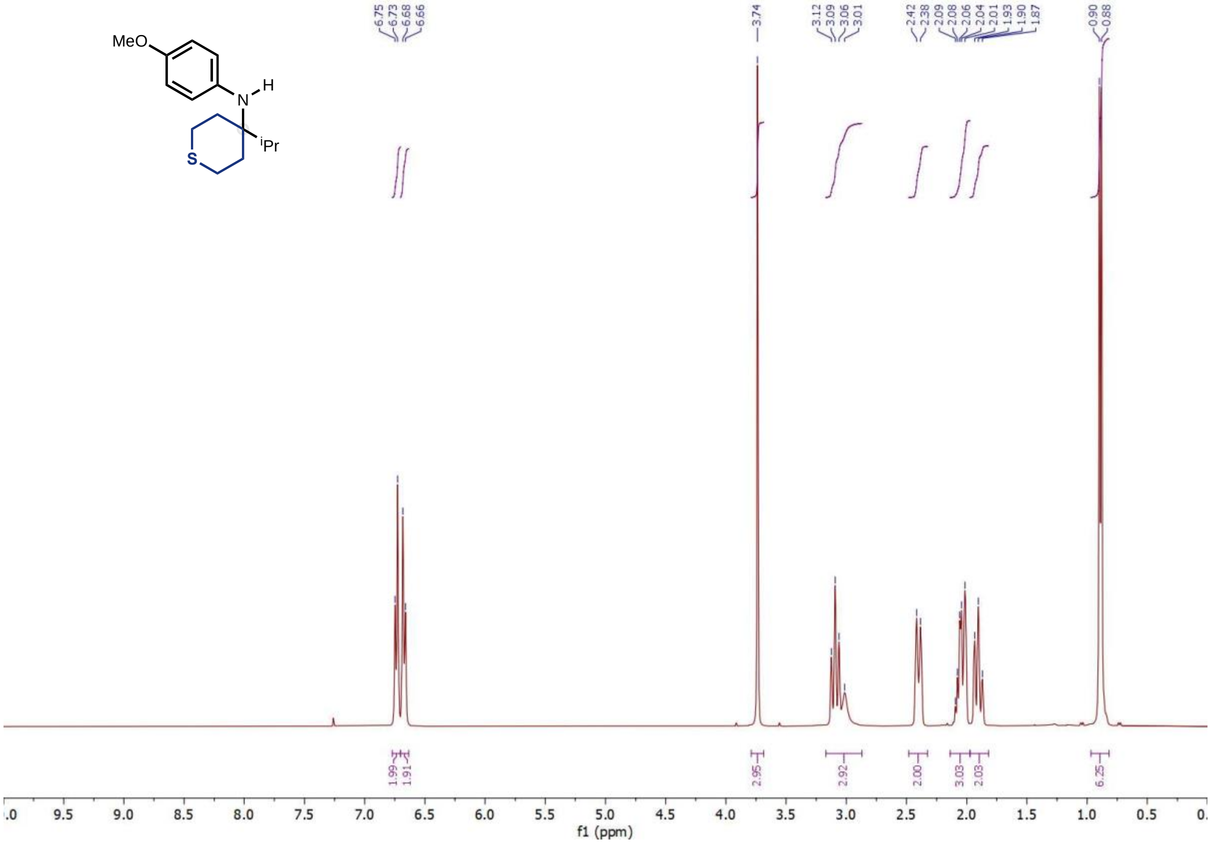

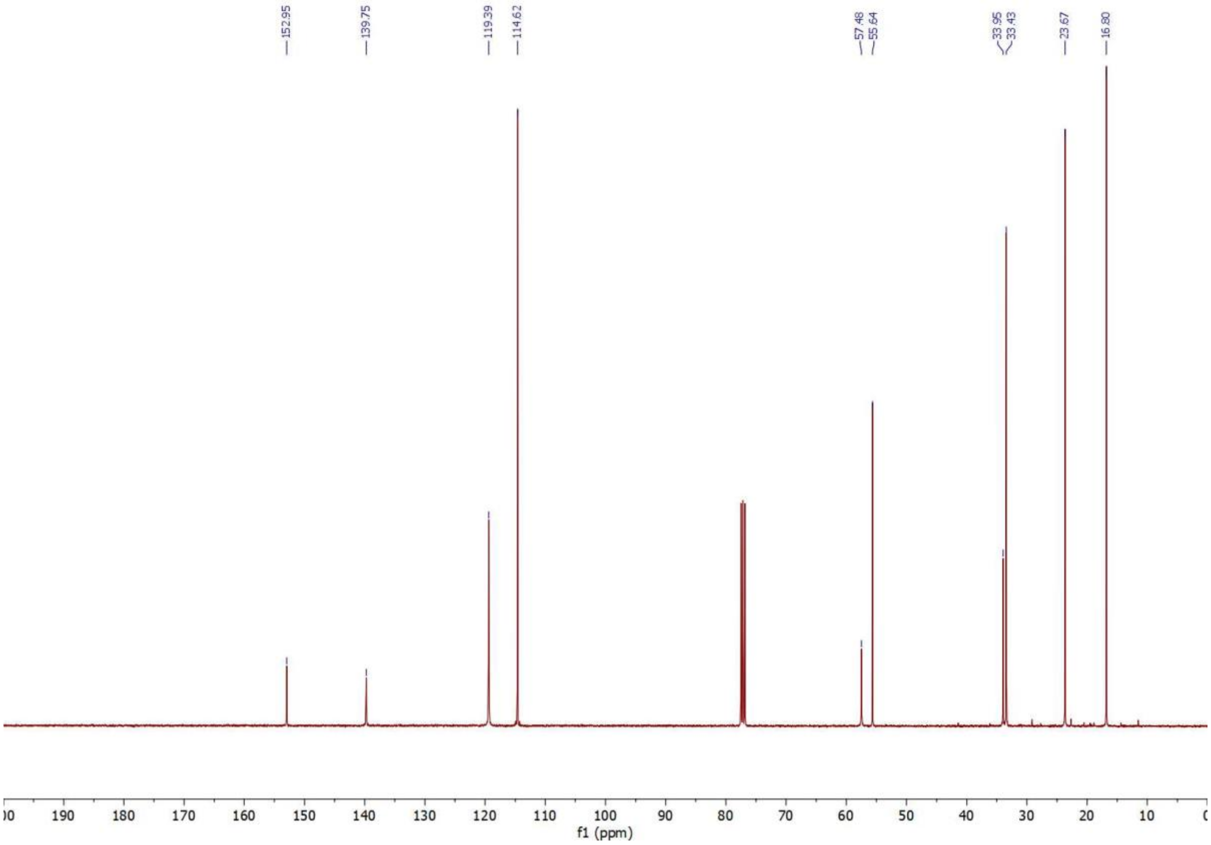

Benzyl 4-isopropyl-4-((4-methoxyphenyl)amino)piperidine-1-carboxylate (19g)

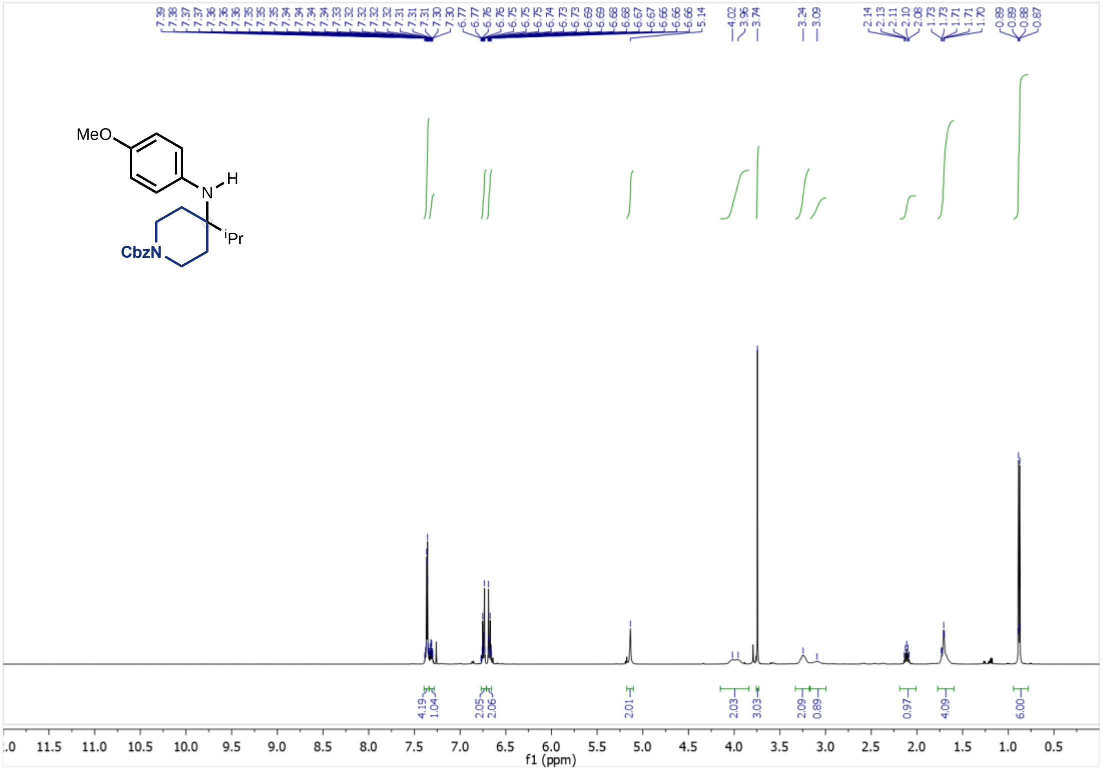

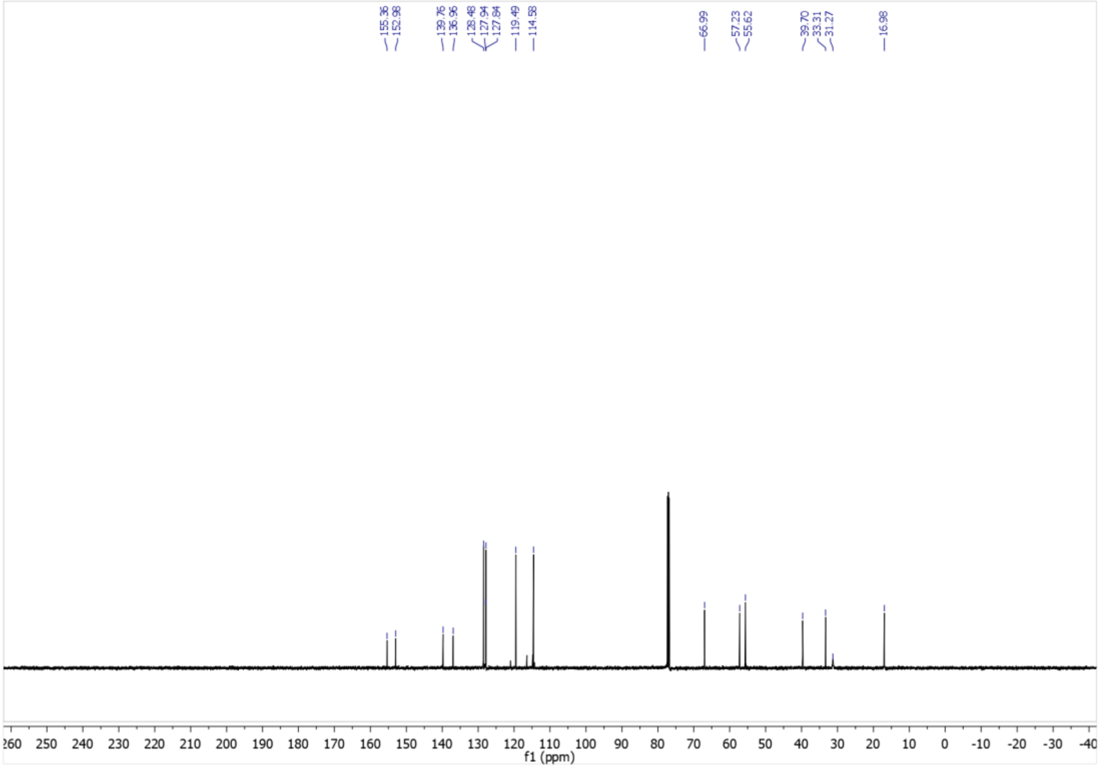

Ethyl 3-isopropyl-3-((4-methoxyphenyl)amino)cyclobutane-1-carboxylate (19h)

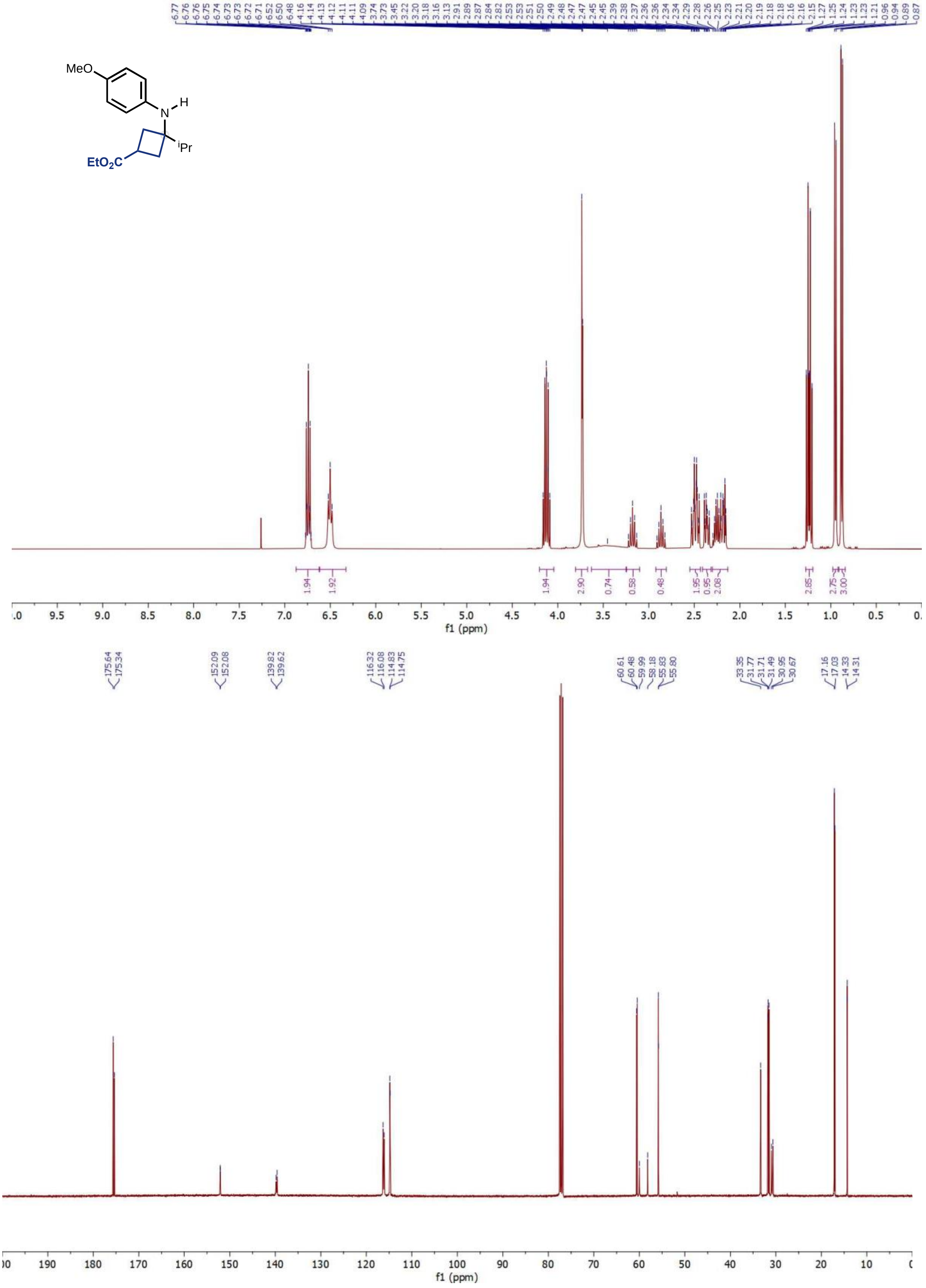

3-isopropyl-N-(4-methoxyphenyl)oxetan-3-amine (19i)

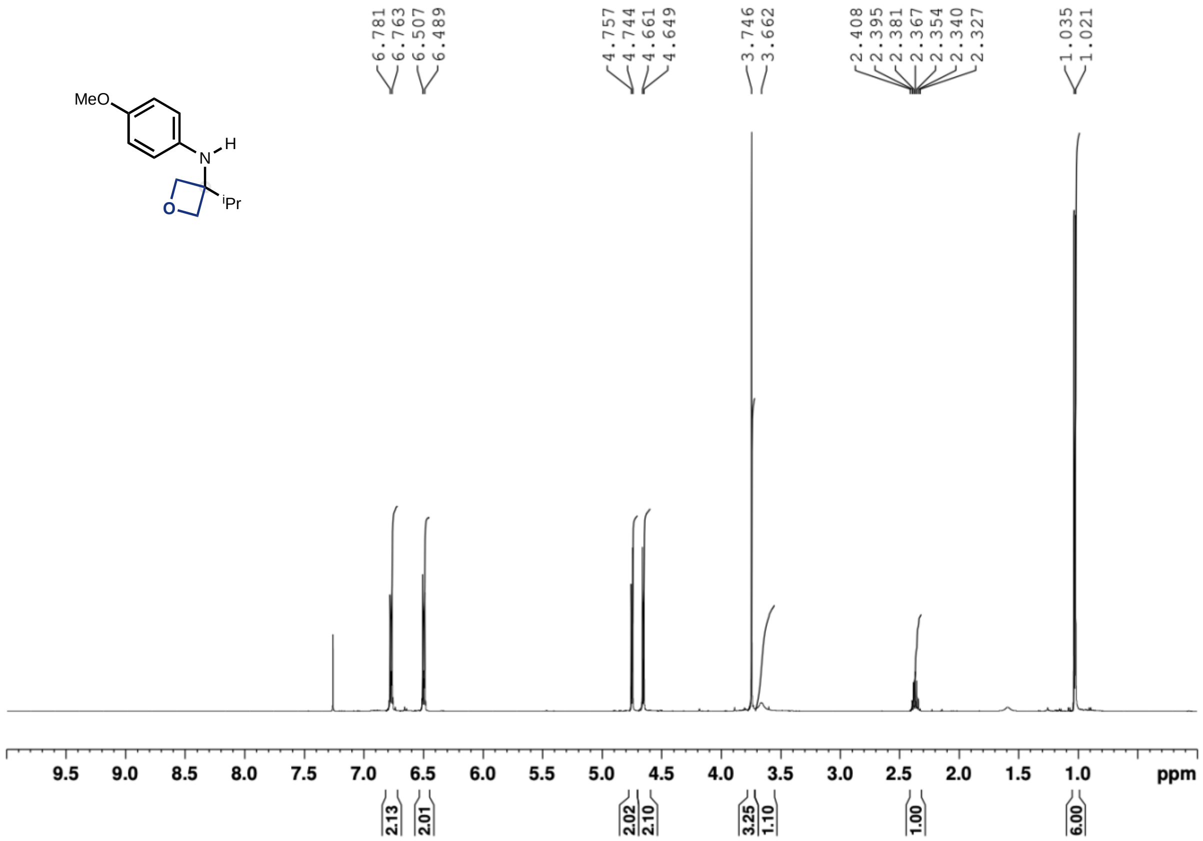

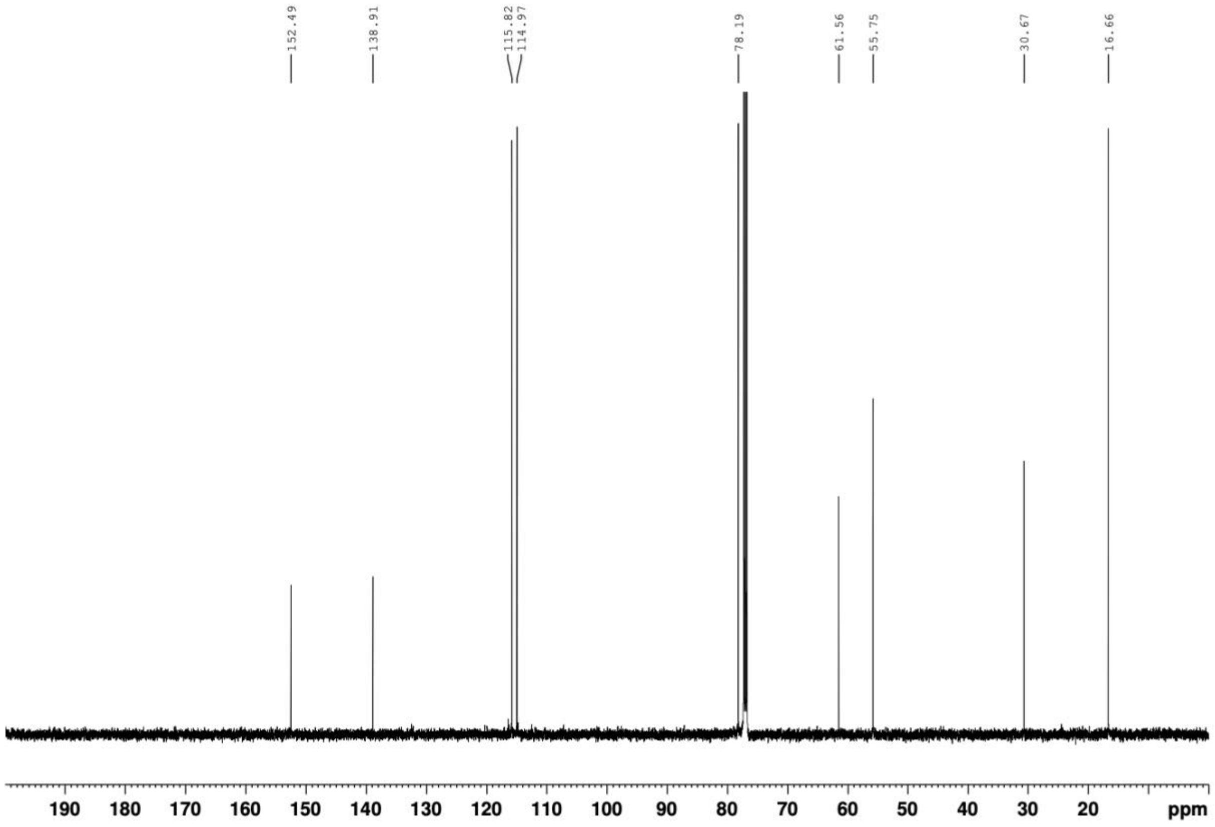

N-(2,3-dimethylhexan-3-yl)-4-methoxyaniline (20a)

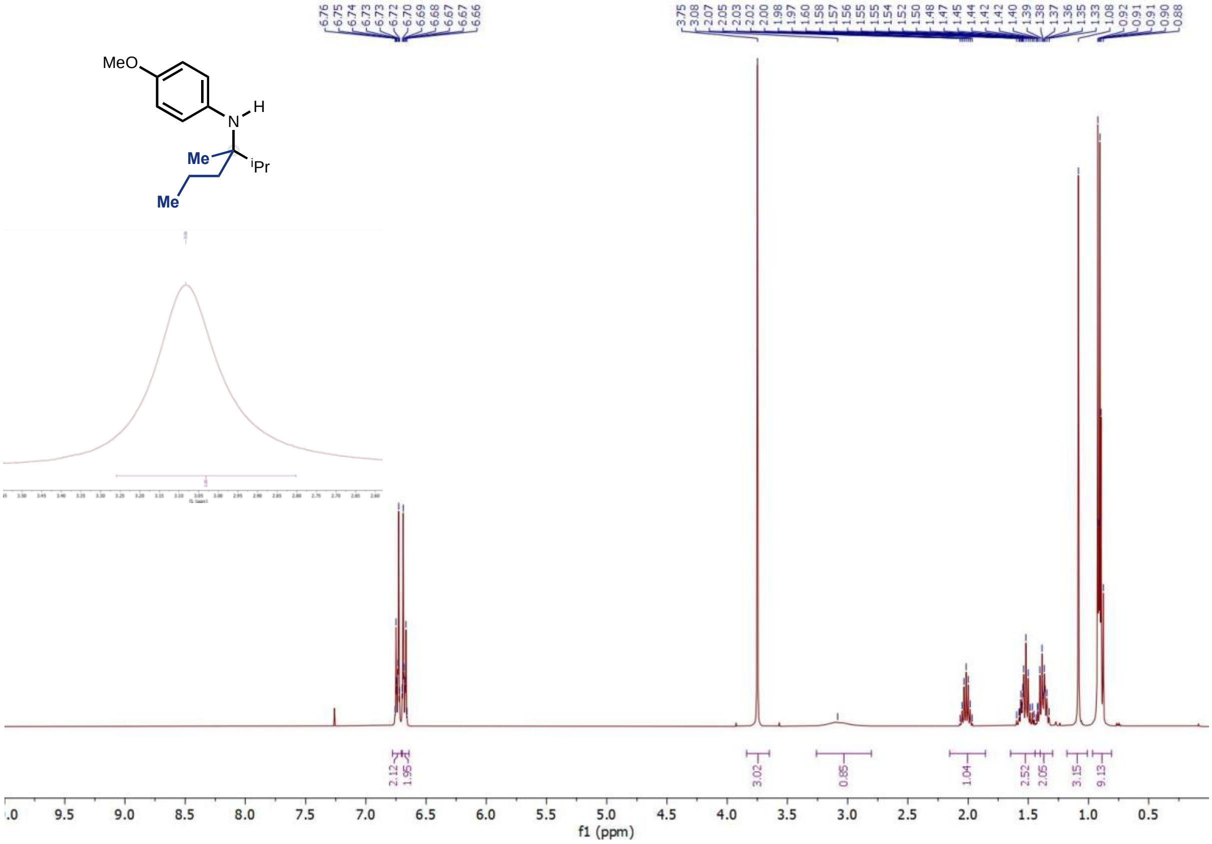

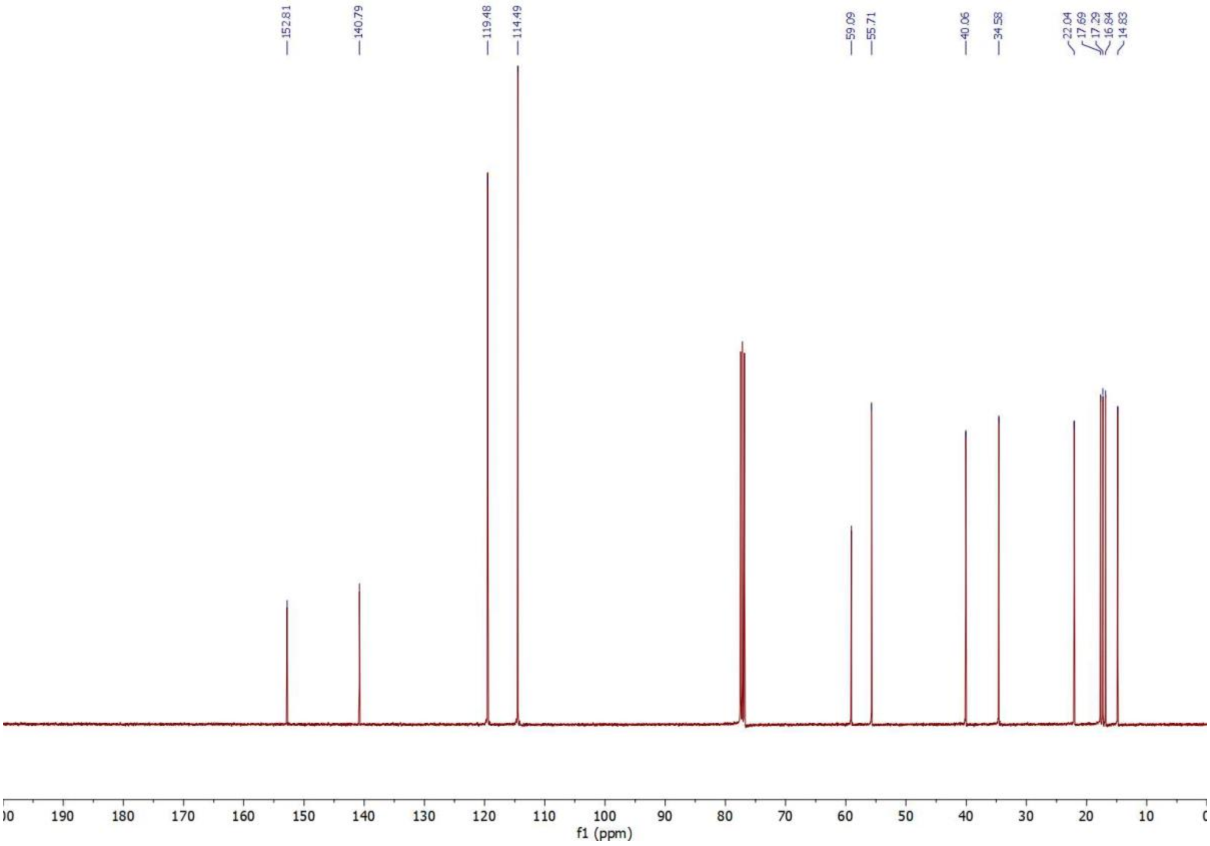

N-(3,4-dimethyl-1-phenylpentan-3-yl)-4-methoxyaniline (20b)

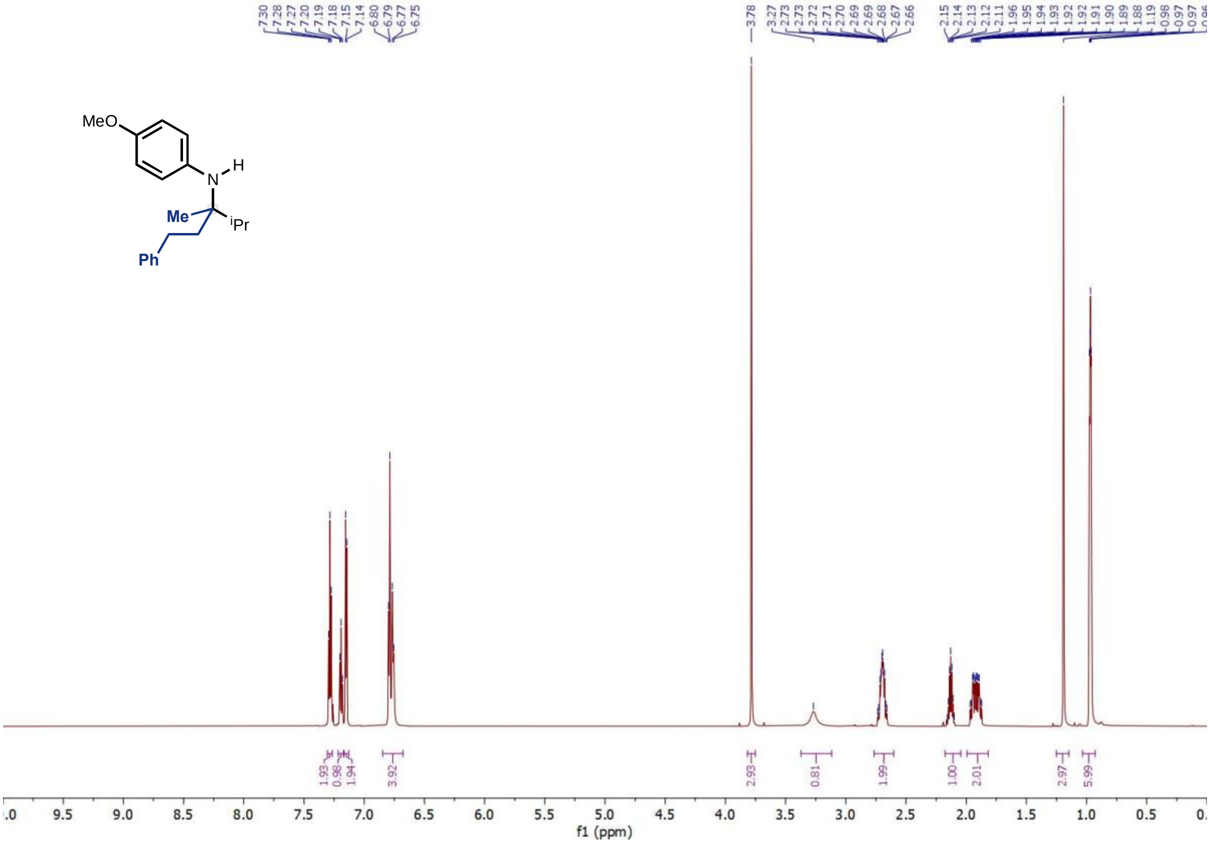

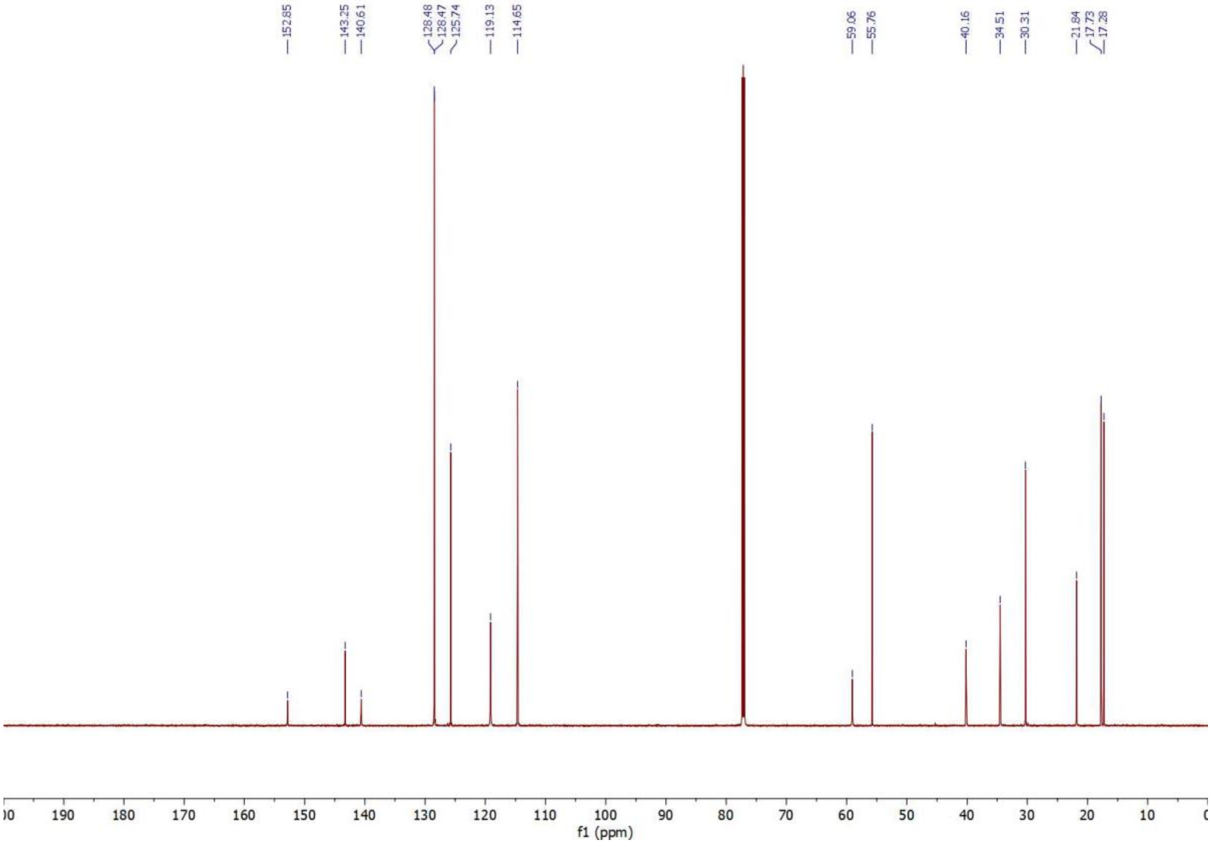

3,4-dimethyl-1-phenylpentan-3-amine

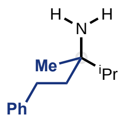

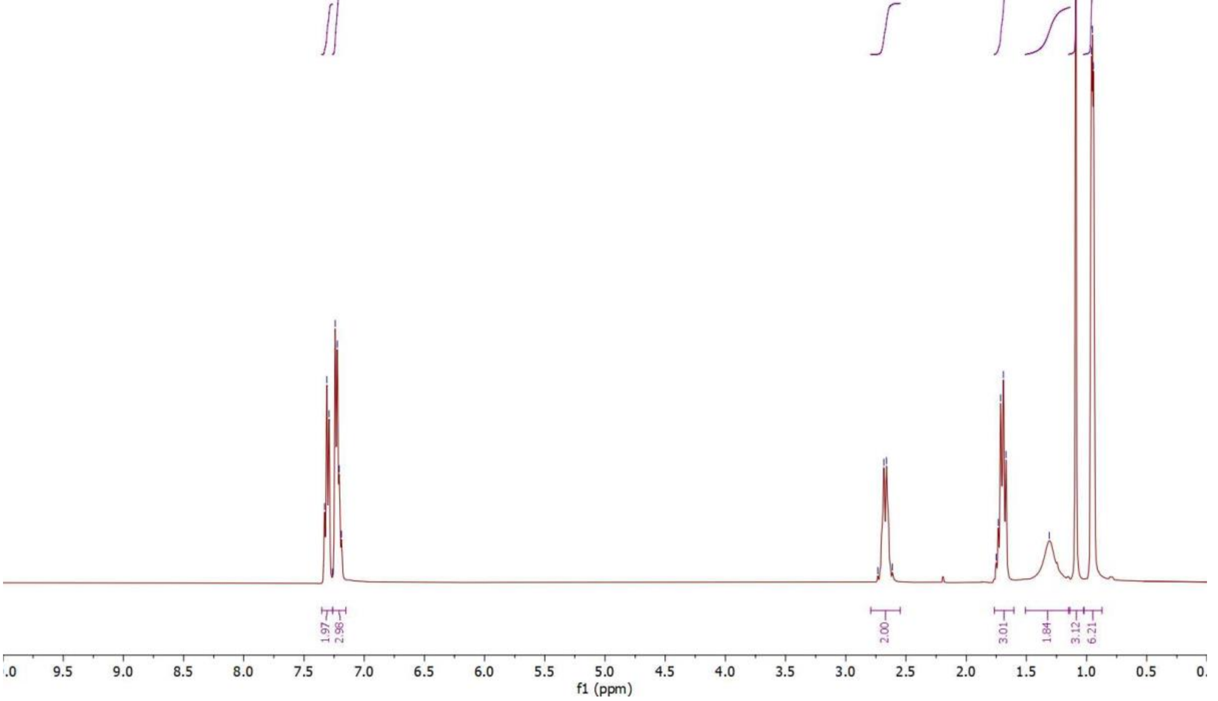

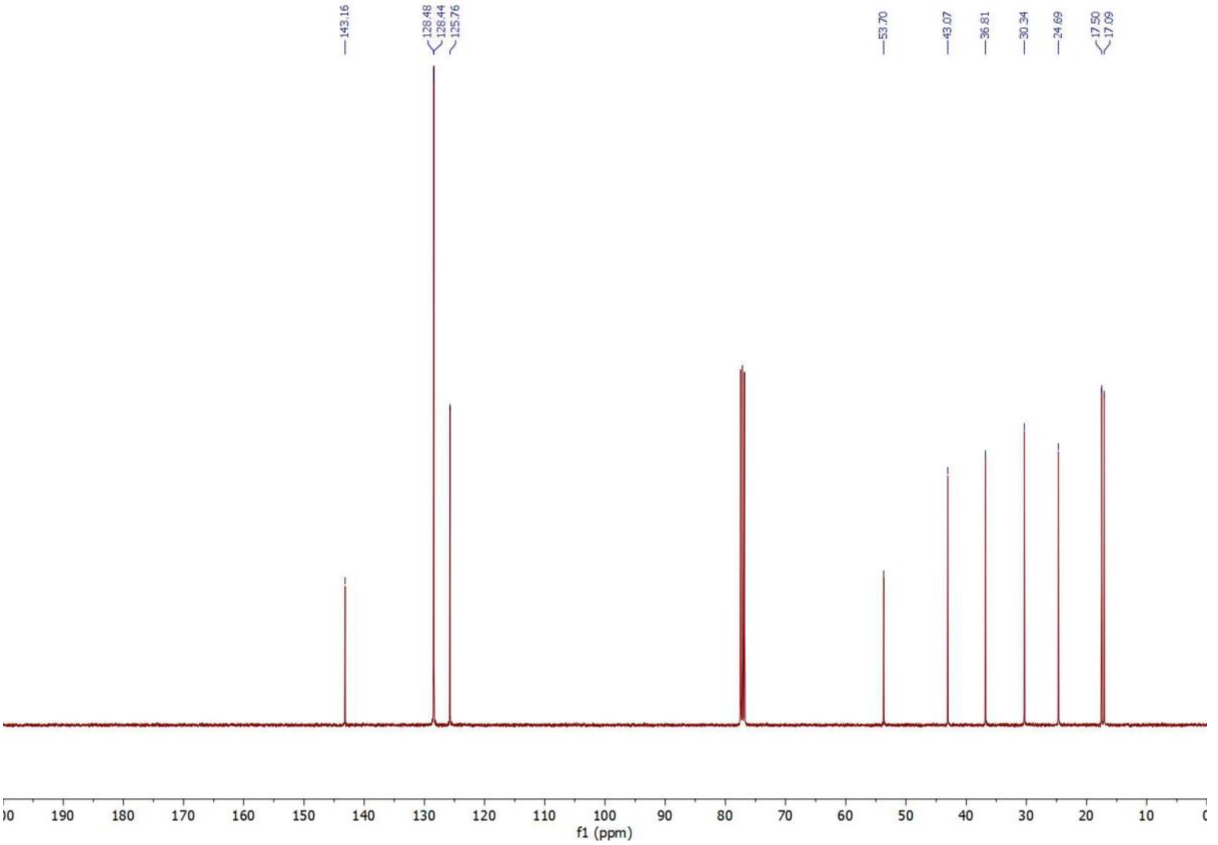

N-(2,3-dimethylhept-6-en-3-yl)-4-methoxyaniline (20c)

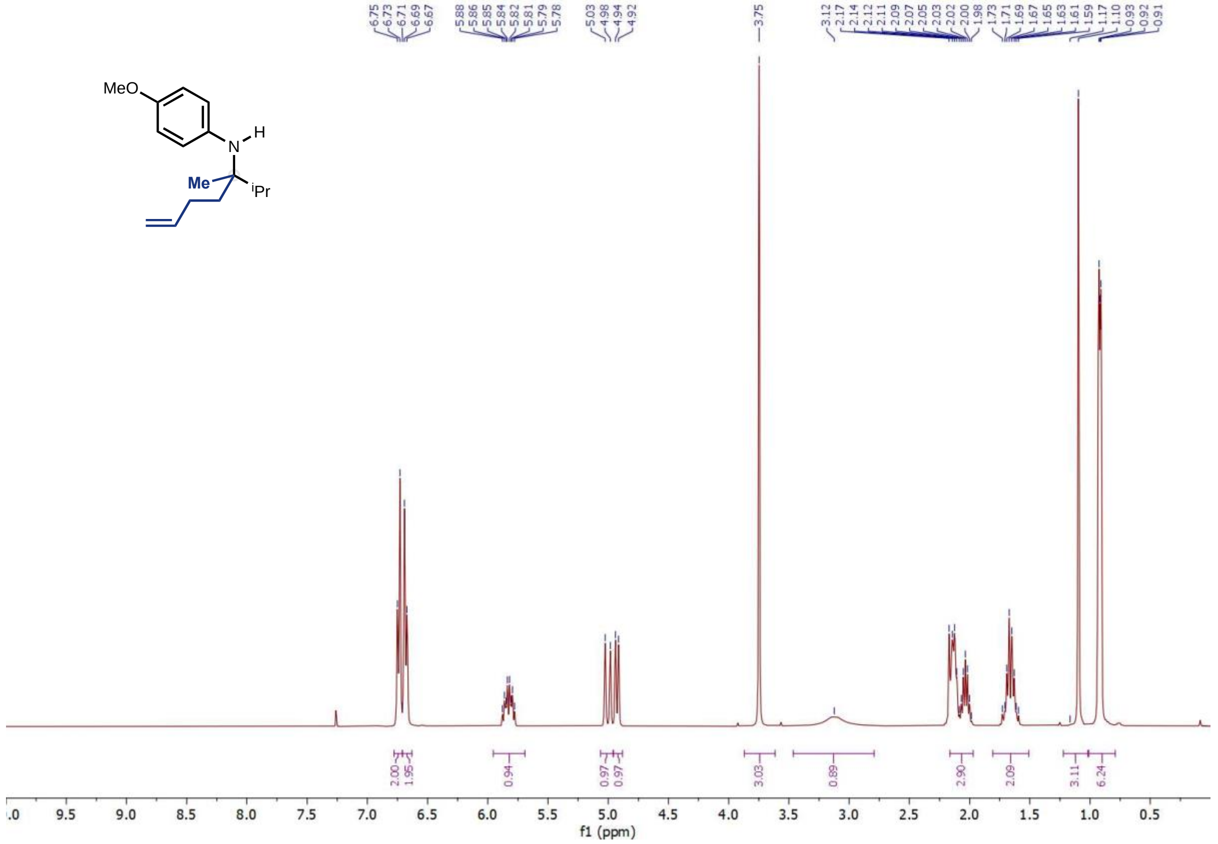

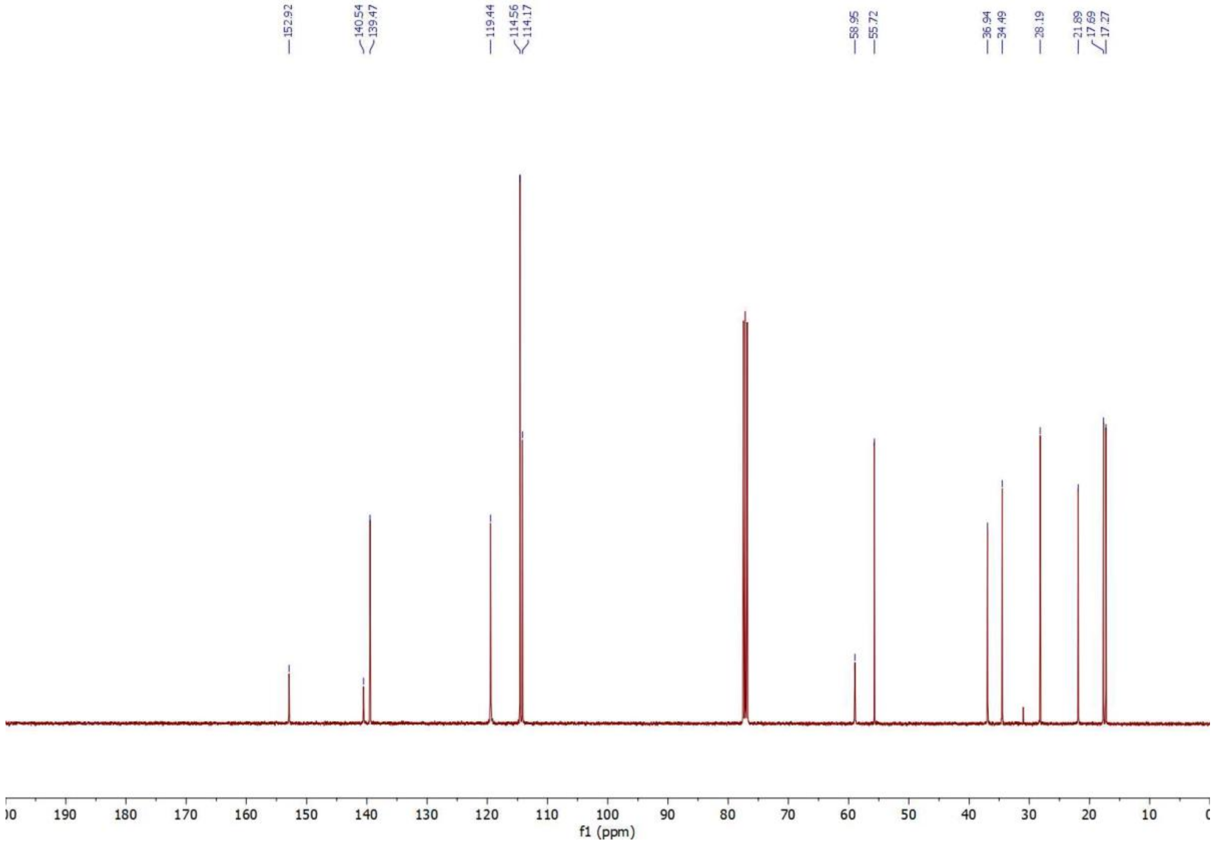

Ethyl 5-((4-methoxyphenyl)amino)-5,6-dimethylheptanoate (20d)

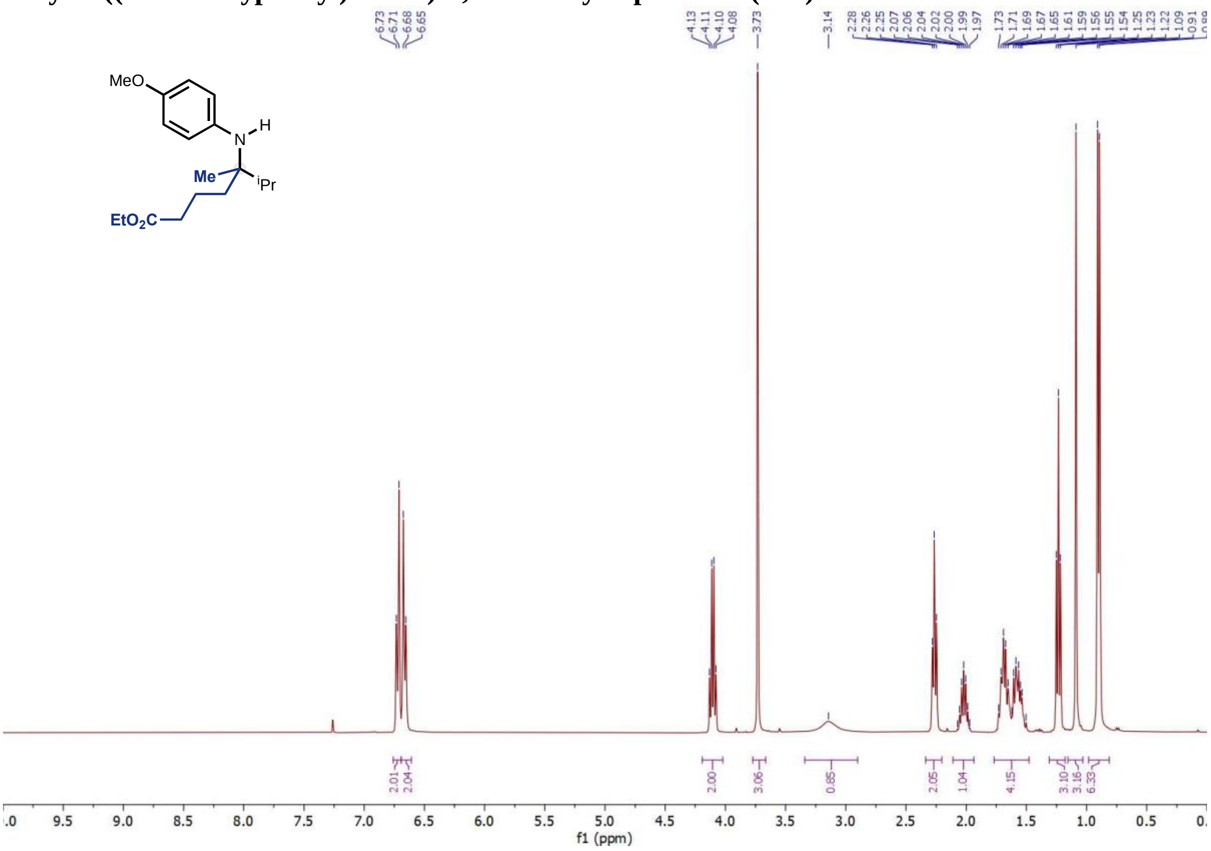

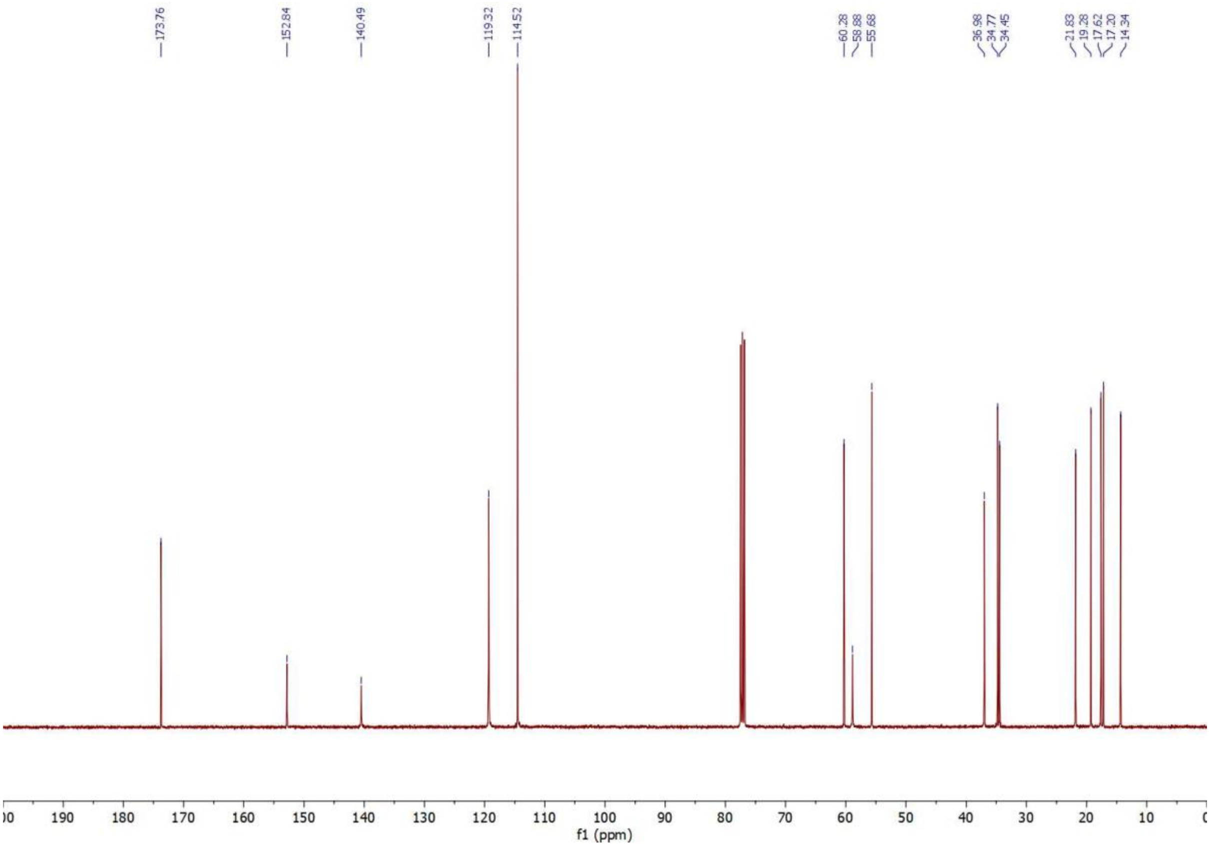

N-(4-methoxyphenyl)heptan-4-imine

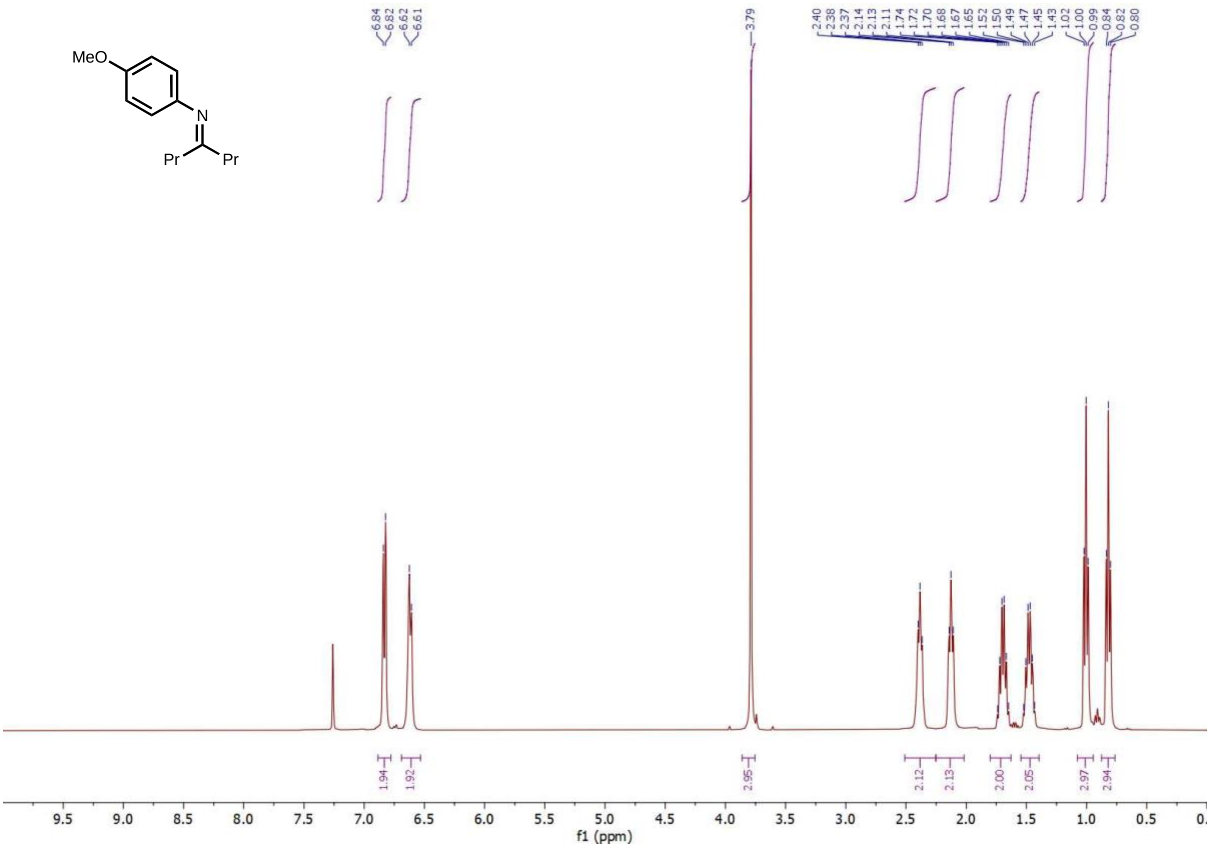

N-(4-isopropylheptan-4-yl)-4-methoxyaniline (20e)

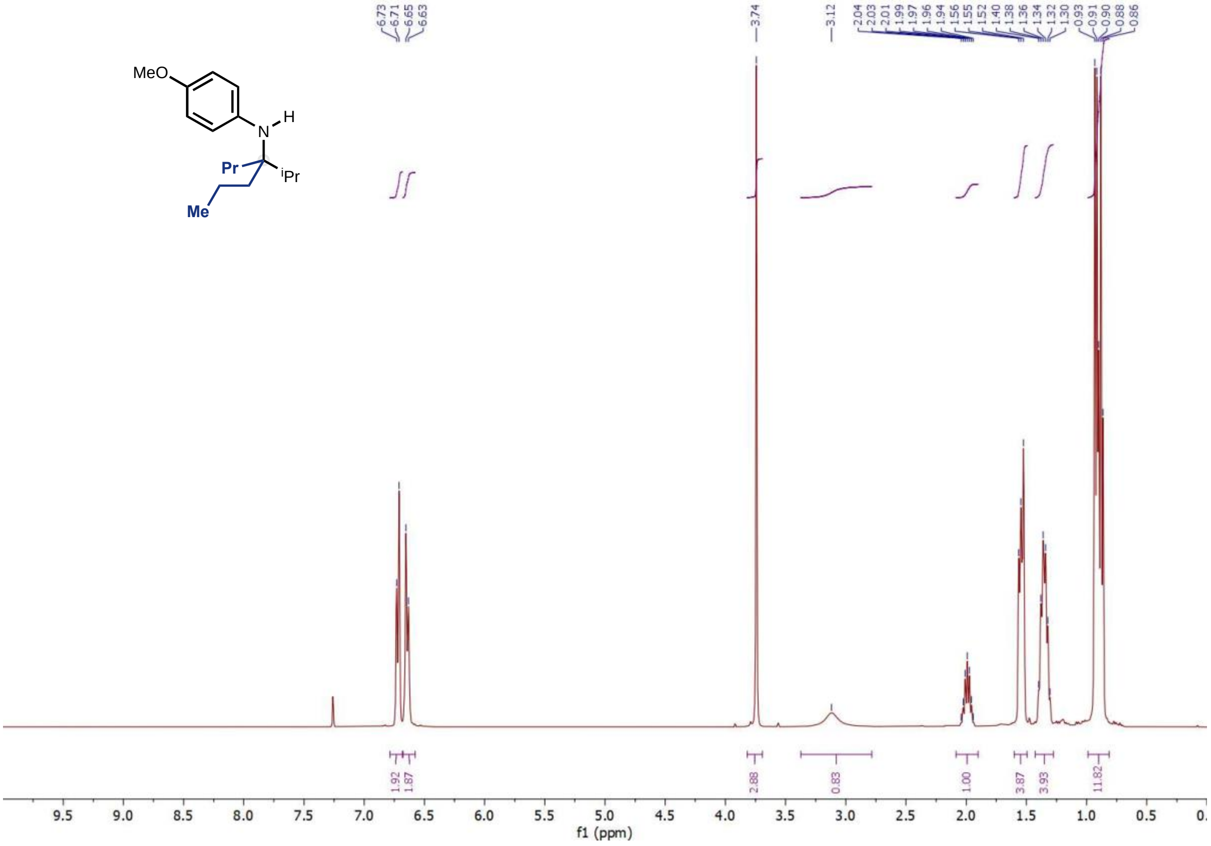

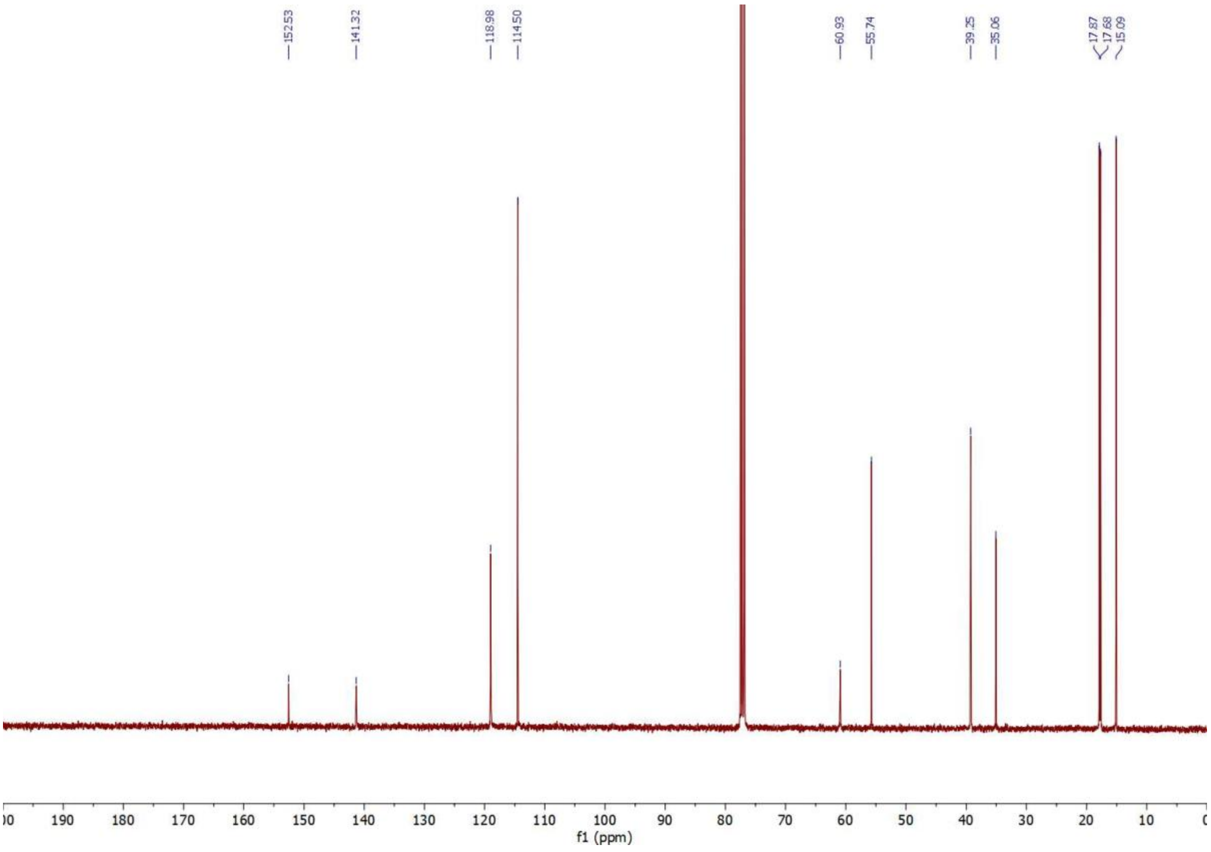

N-(2,3-dimethylpentan-2-yl)-4-methoxyaniline (21a)

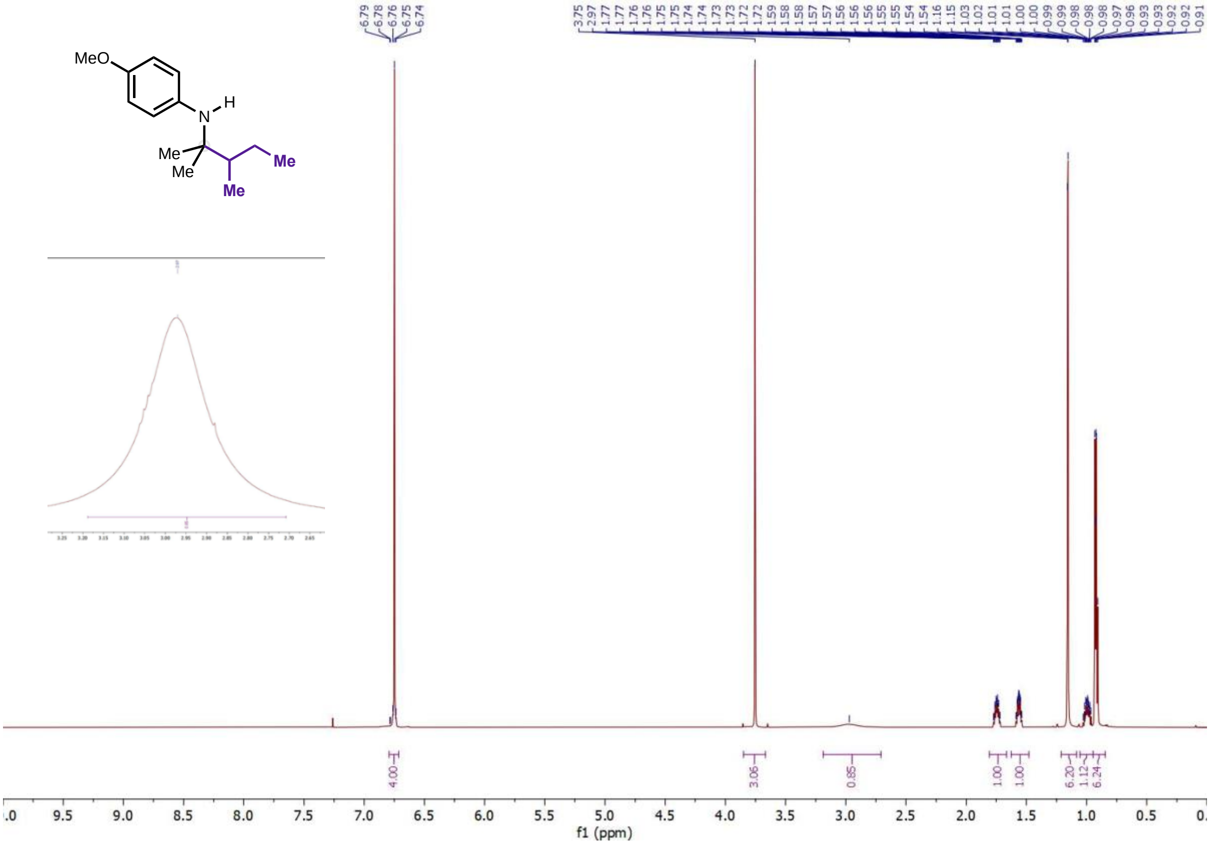

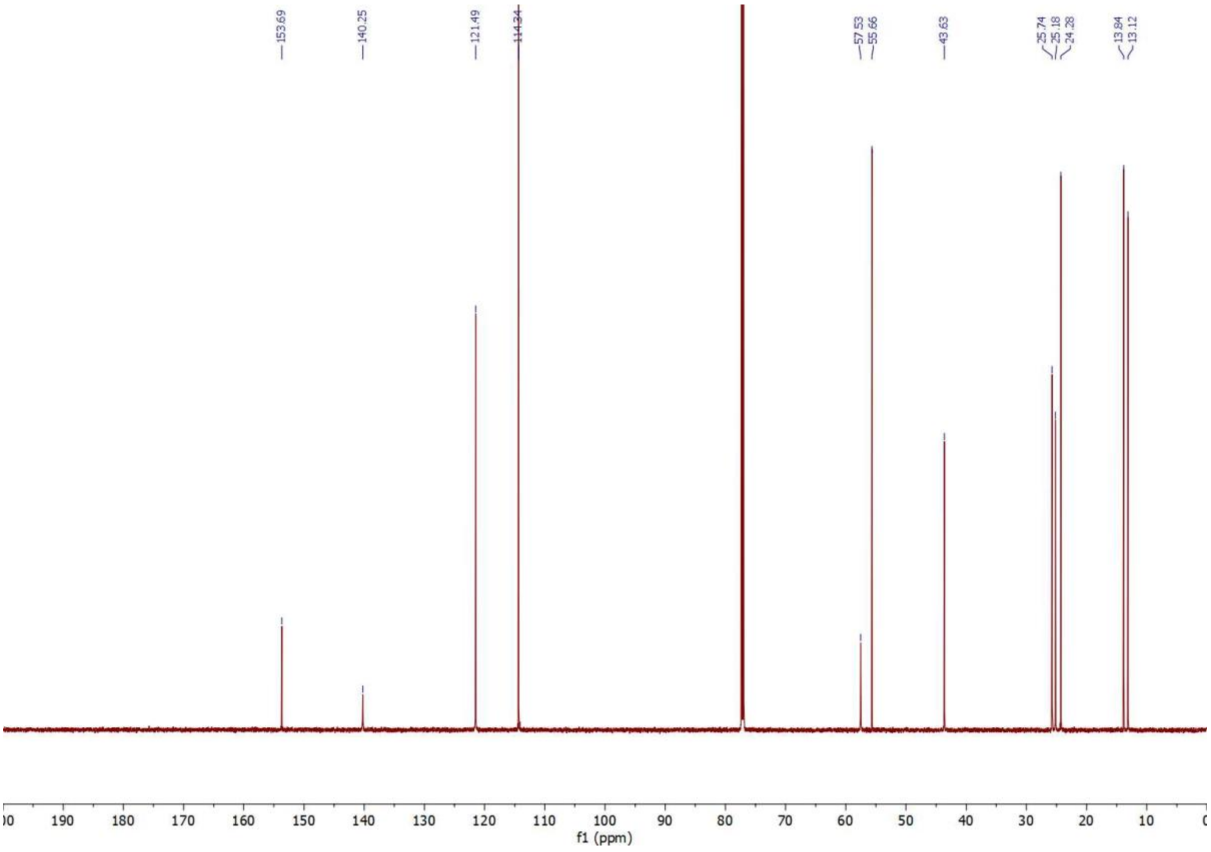

N-(2-cyclopentylpropan-2-yl)-4-methoxyaniline (21b)

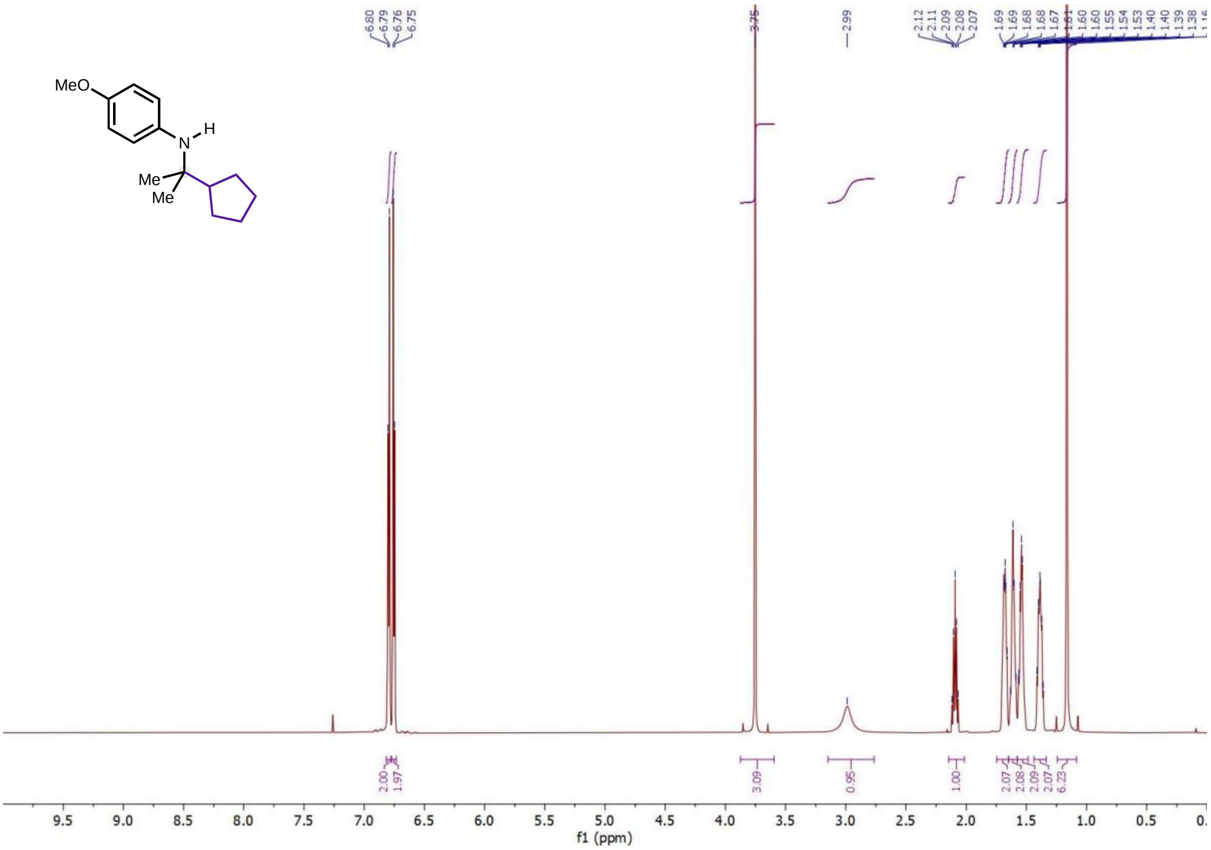

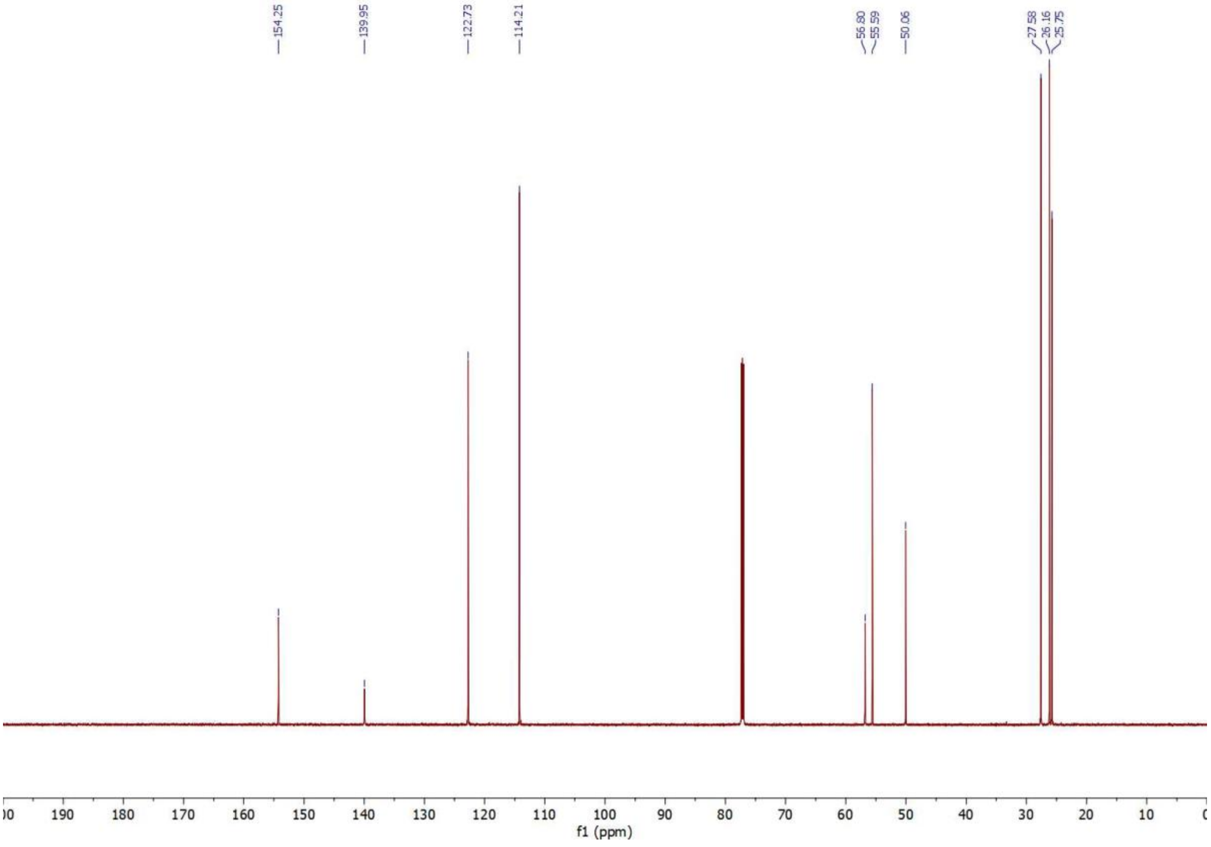

N-(2-(2,3-dihydro-1H-inden-2-yl)propan-2-yl)-4-methoxyaniline (21c)

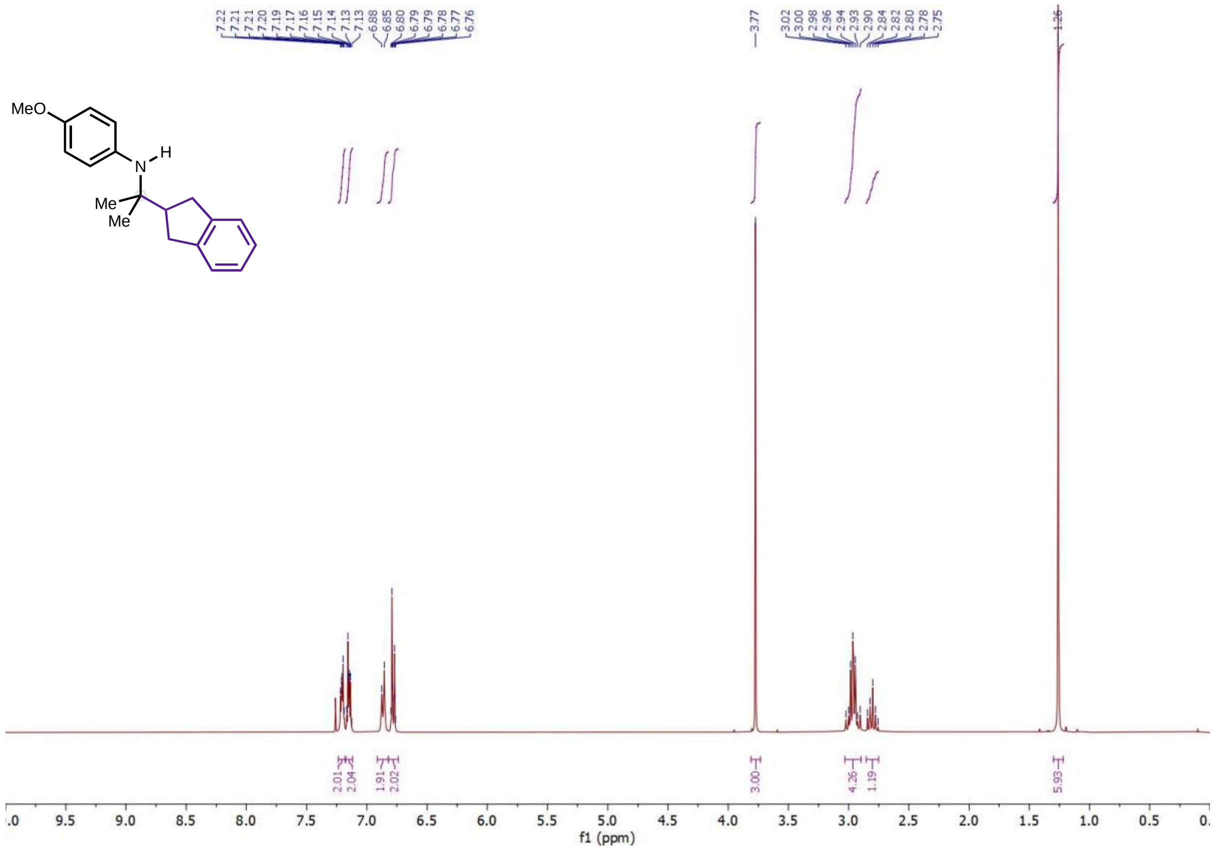

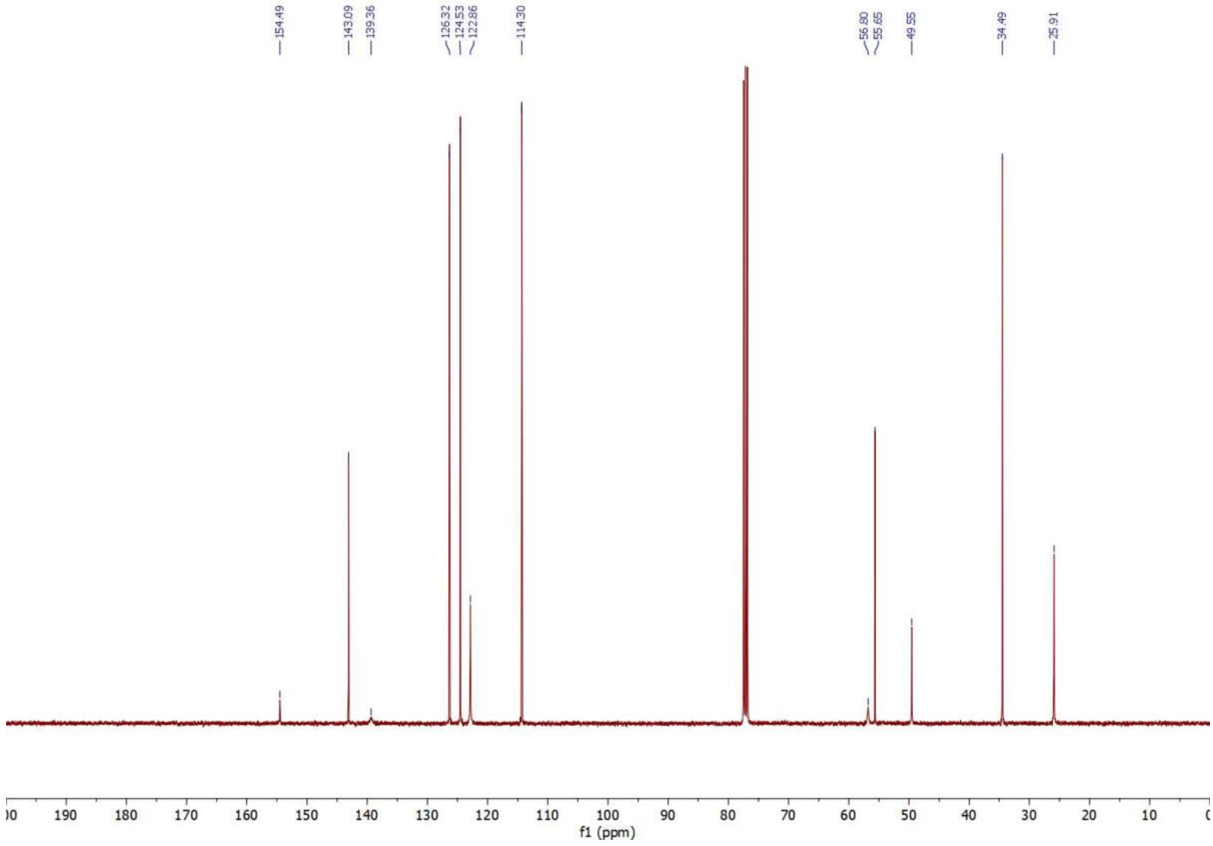

4-methoxy-N-(2-methylpentan-2-yl)aniline (21d)

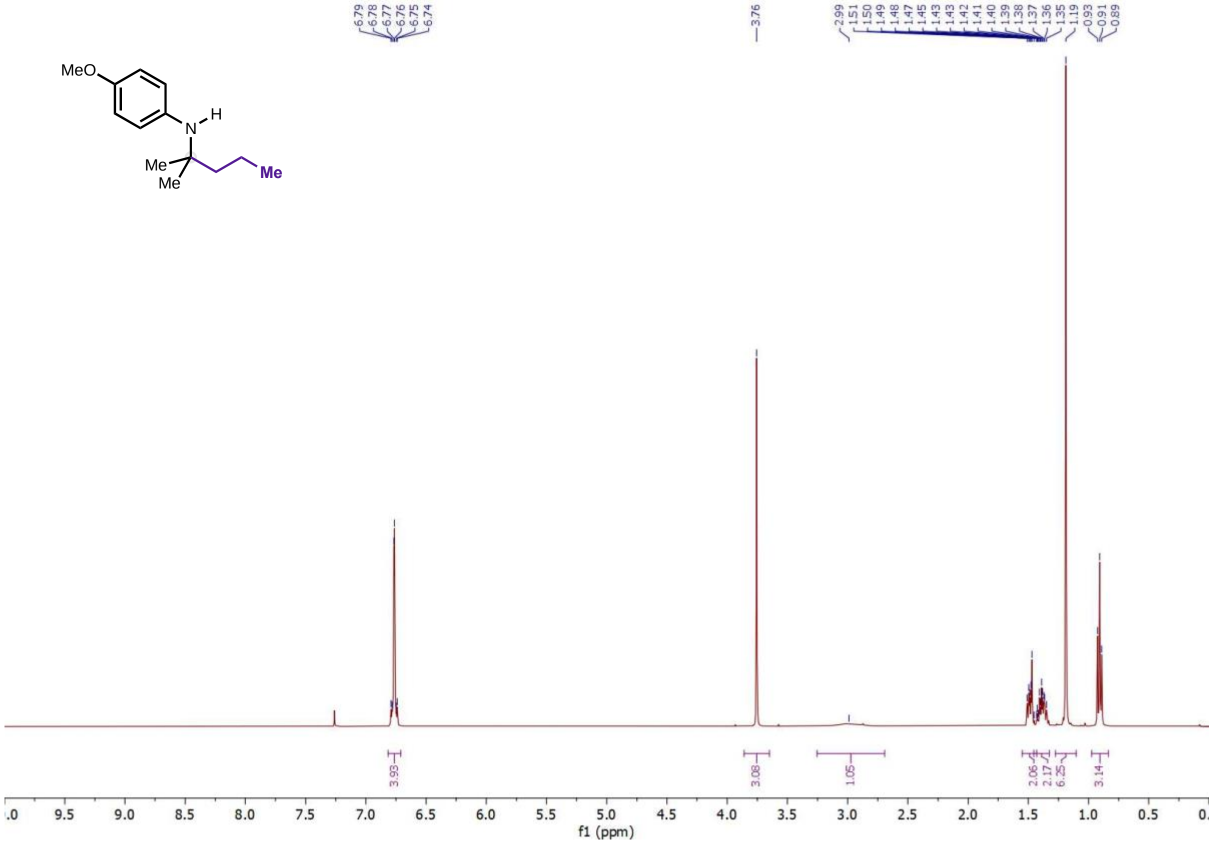

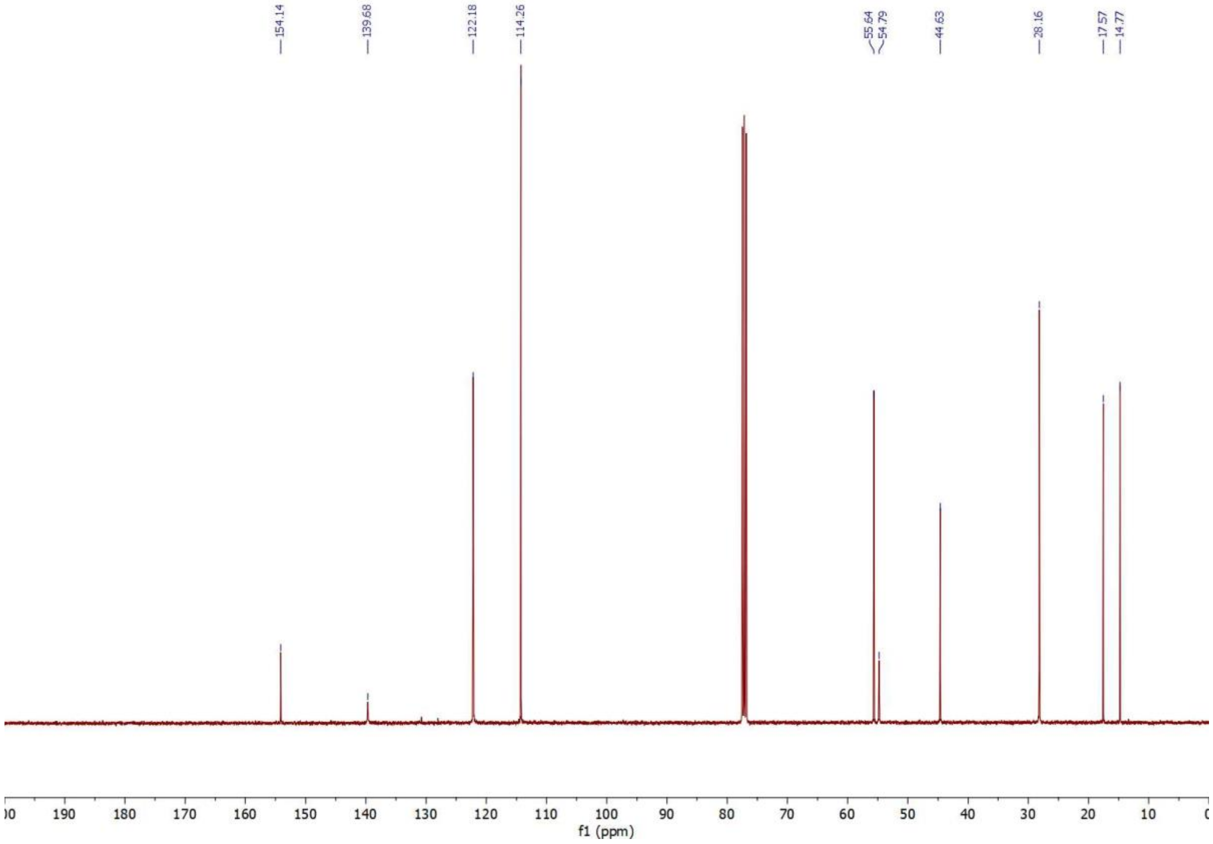

N-benzyl-N-(1-phenylpentan-3-yl)cyclopropanamine

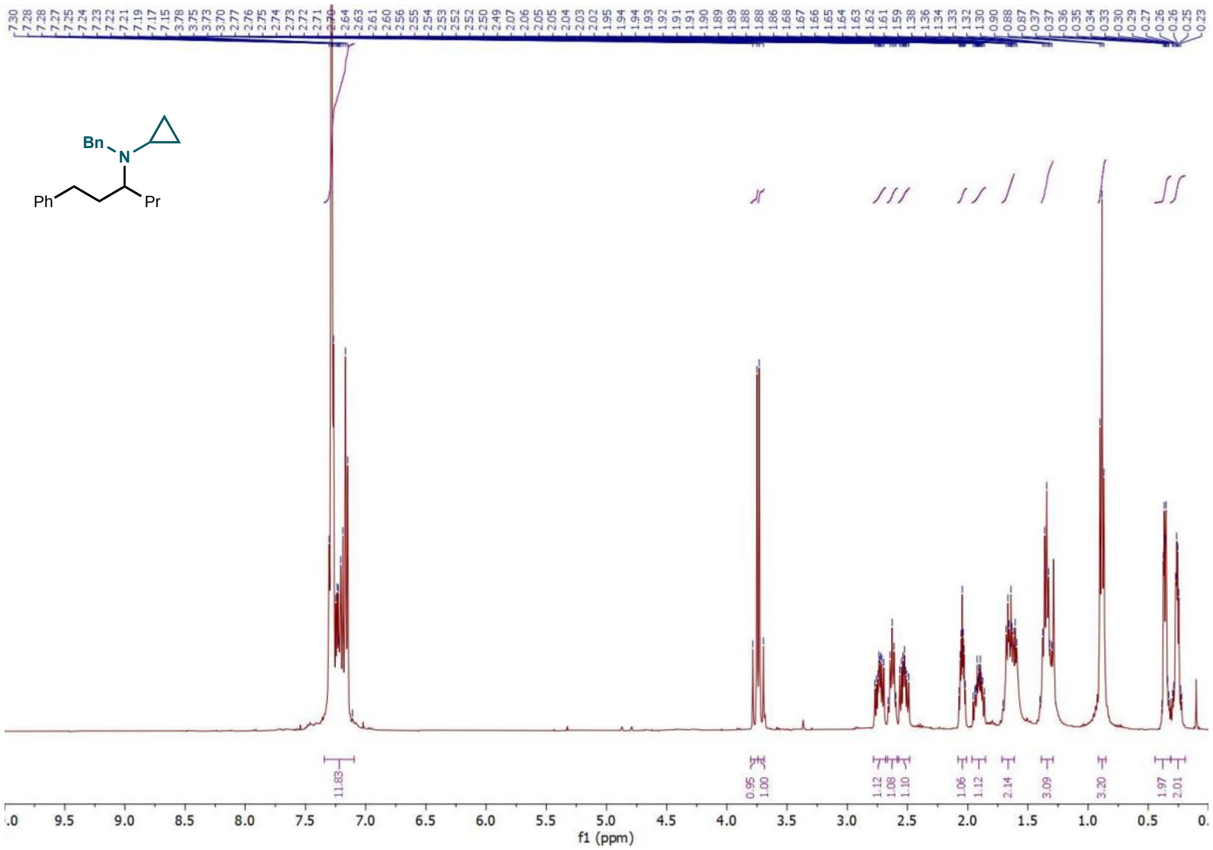

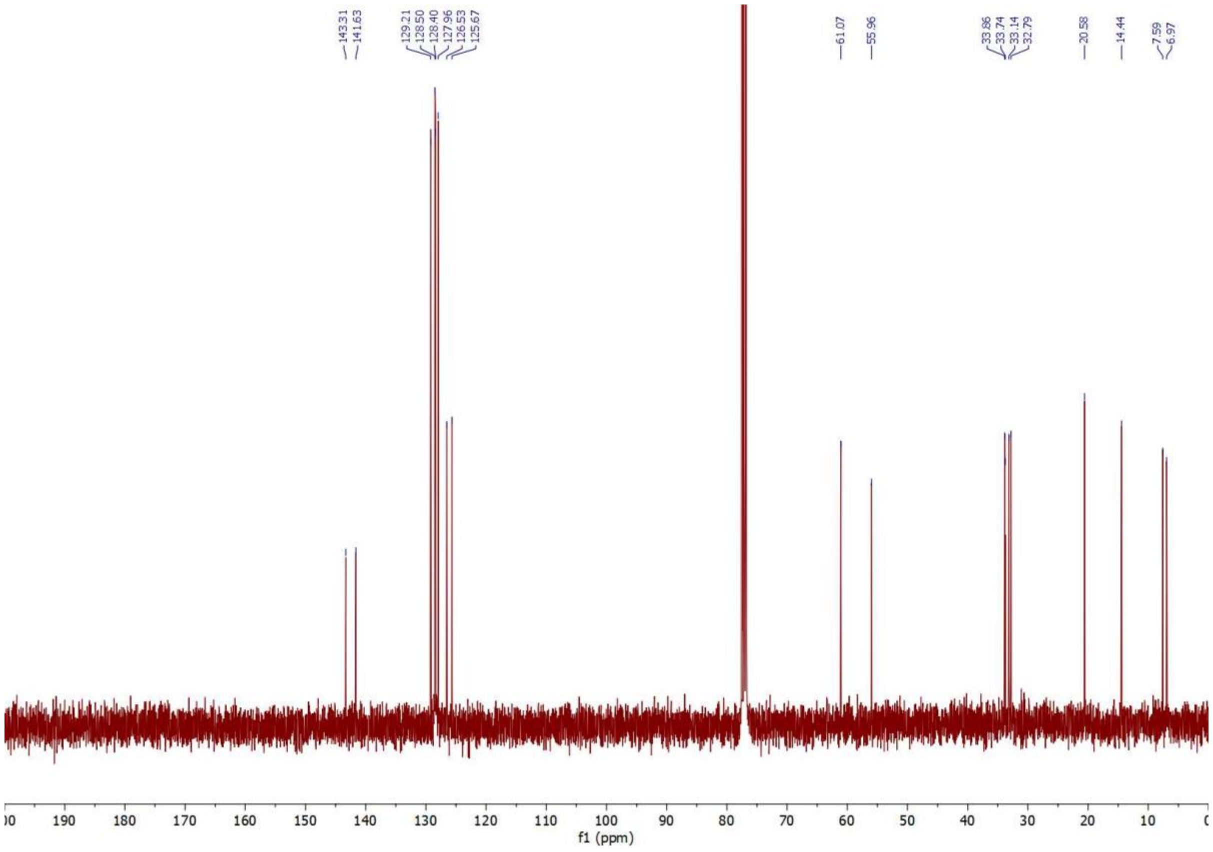

N-benzyl-1-phenylpentan-3-amine

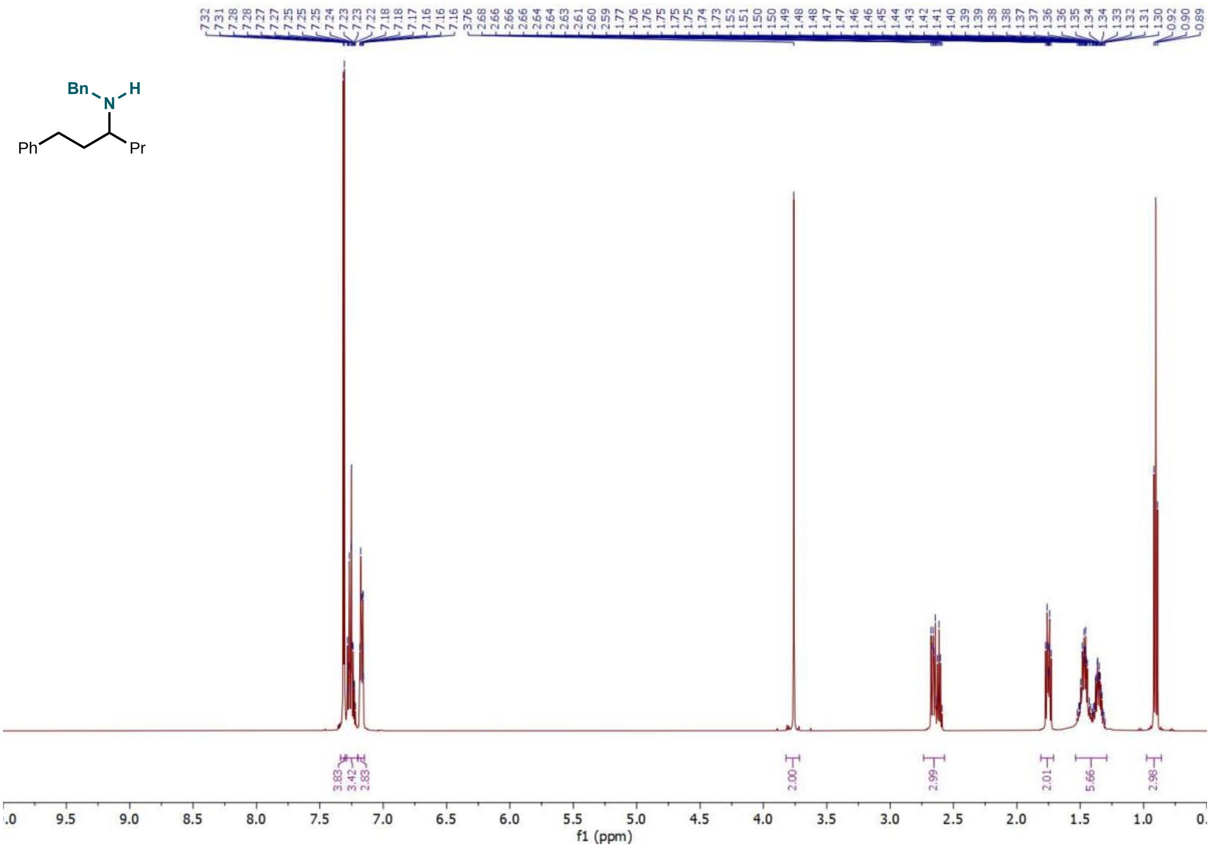

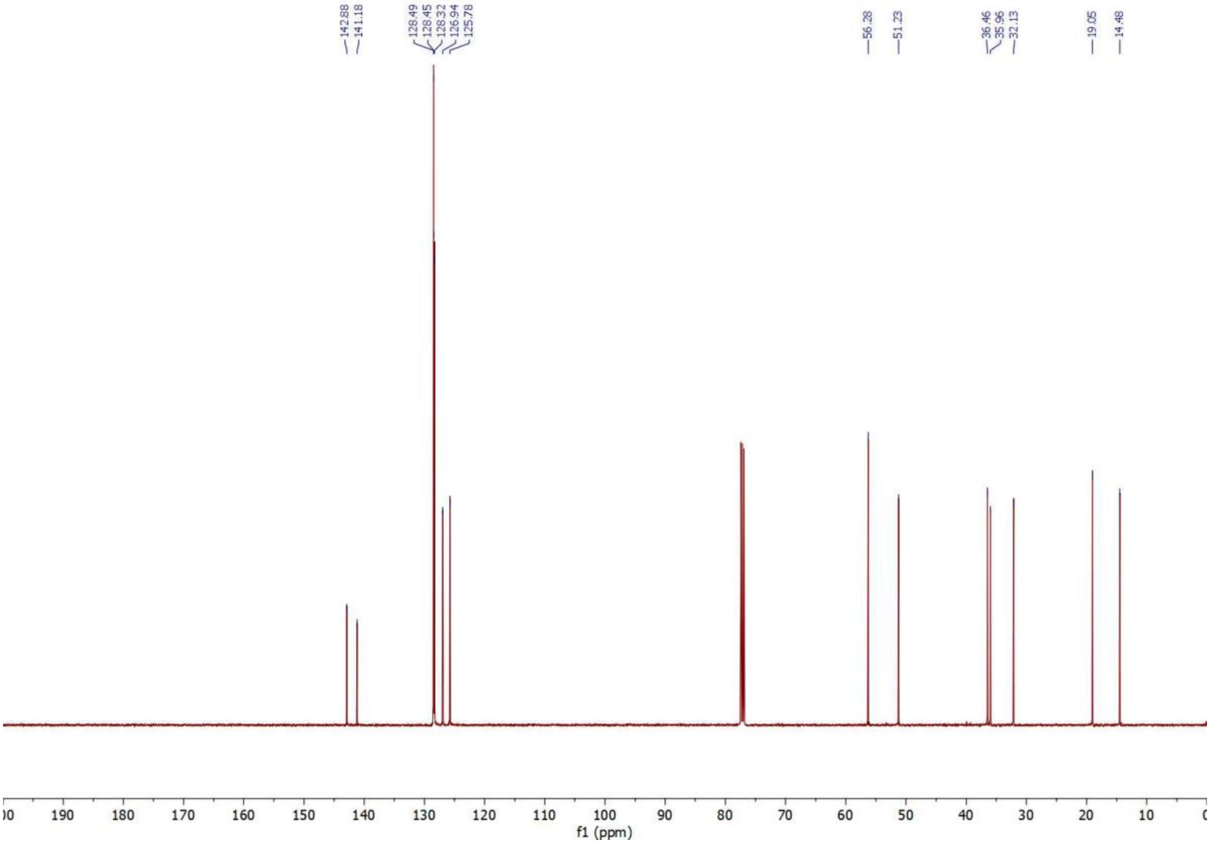

N-benzyl-N-(4-methyl-1-phenylpentan-3-yl)cyclopropanamine

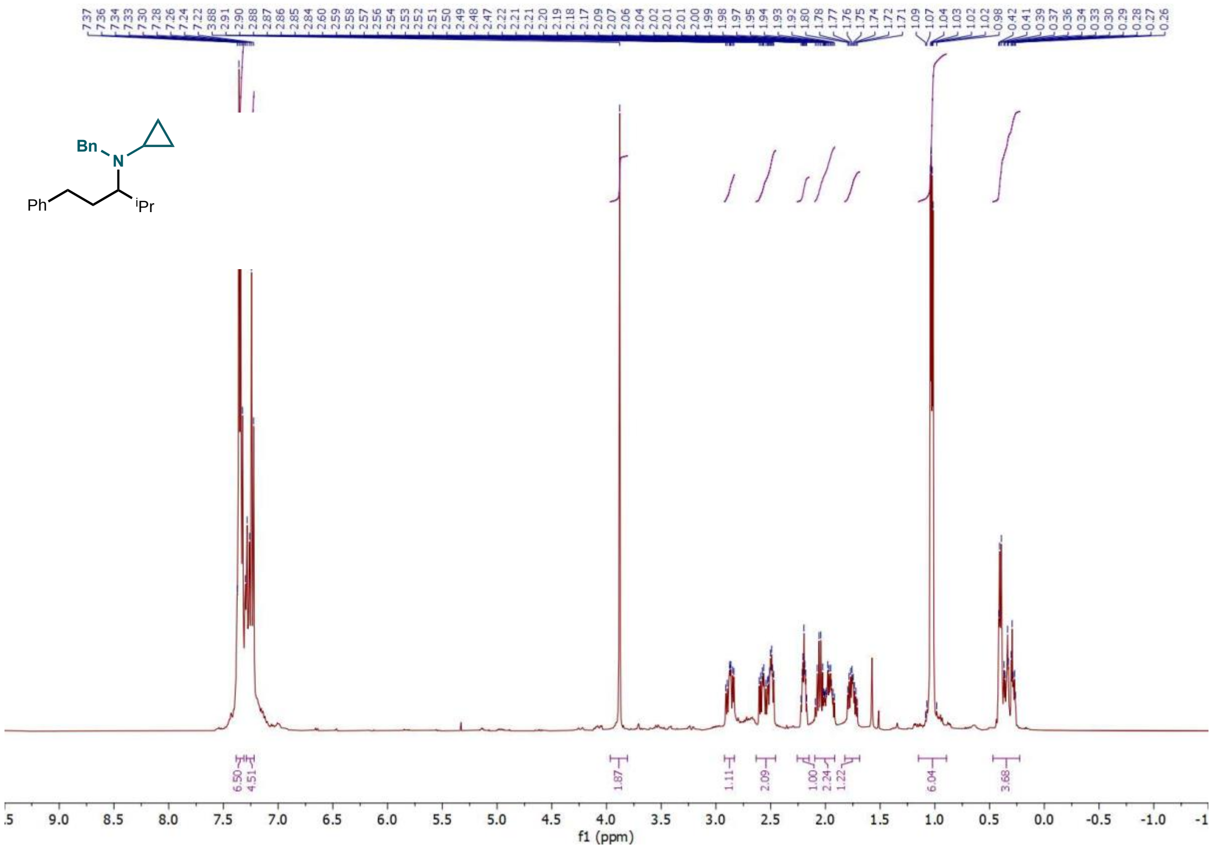

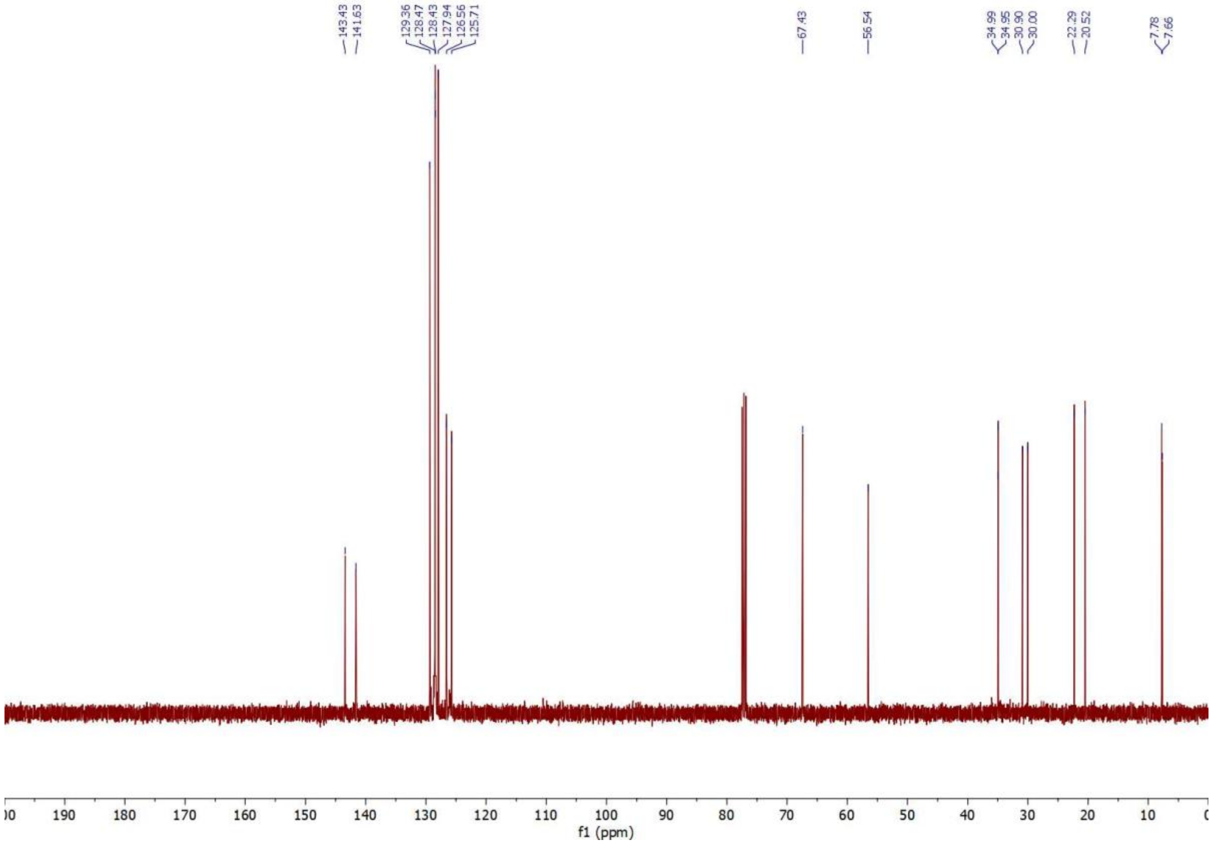

Supplement: Supplementary file 1 — ja3c14037_si_001.pdf [file ja3c14037_si_001.pdf]
